# Glycosite mapping and in situ mass spectrometry imaging of MUC2 glycopeptides via on-slide mucinase digestion

**DOI:** 10.1038/s41467-026-72853-3

**Published:** 2026-05-07

**Authors:** Sarah C. Lowery, Mikaela K. Ribi, Joann Chongsaritsinsuk, Isabella P. Tran, Grace Grimsley, Rachel Stubler, Keira E. Mahoney, Taryn M. Lucas, Georgia Charkoftaki, Alvaro Santos-Neto, Nissi Varki, Vasilis Vasiliou, Michael Angelo, Richard R. Drake, Stacy A. Malaker

**Affiliations:** 1 Department of Chemistry, Yale University, New Haven, CT USA; 2 Department of Pathology, School of Medicine, Stanford University, Stanford, CA USA; 3 Department of Pharmacology and Immunology, Medical University of South Carolina, Charleston, SC USA; 4 Department of Regenerative Medicine and Cell Biology, Medical University of South Carolina, Charleston, SC USA; 5 Department of Environmental Health Sciences, Yale School of Public Health, Yale University, New Haven, CT USA; 6 São Carlos Institute of Chemistry, University of São Paulo, São Carlos, Brazil; 7 Department of Pathology, University of California, San Diego, La Jolla, CA USA; 8 Glycobiology Research and Training Center, University of California San Diego, La Jolla, CA USA; 9 Present Address: Department of Pediatrics, Division of Critical Care and Hospital Medicine, Columbia University Irving Medical Center, New York, NY 10032 USA

**Keywords:** Glycobiology, Mass spectrometry, Tumour heterogeneity, Proteomics

## Abstract

Current analytical techniques are limited in their ability to interrogate the mechanistic relationship between aberrant mucin glycosylation and malignancy. Herein, we describe a method for mapping specific mucin glycoforms in diseased tissue, enabling correlation of the tumor glycan profile with malignant features. Following on-tissue digestion with mucinase StcE, matrix-assisted laser desorption ionization mass spectrometry imaging (MALDI-MSI) deduces the spatial distribution of mucinous O-glycopeptides that are subsequently identified using liquid chromatography coupled to mass spectrometry (LC-MS). Our coupled MS approach reveals a striking mucin 2 (MUC2) expression pattern in three colorectal mucinous adenocarcinomas, in which different glycoforms clearly stratify regions within each tumor. The LC-MS experiments obtain glycoproteomic sequence coverage of MUC2’s mucin domains in unprecedented depth, and we also present evidence for an endogenous O-acetylated GalNAc. Overall, this proof-of-concept work underscores the potential of this technique to drive research in oncology and beyond.

## Abstract (web summary)

The authors developed a dual-mass spectrometry workflow to localize and identify O-glycopeptides generated by mucinase StcE in tumor tissue, detecting glycoform-specific expression patterns in three colorectal carcinomas.

## Introduction

Protein glycosylation is the most abundant post-translational modification (PTM), predicted to occur on over 50% of the human proteome^1^, the majority of which can be classified as either N-linked or O-linked^2^. N-linked glycosylation occurs primarily on asparagine residues within an NXS/T sequon, whereby Asn is followed first by any amino acid except proline, then a serine or threonine^3^. All N-linked glycans are derived from the same chitobiose core, which is further modified by various glycosidases and glycosyltransferases in the Golgi apparatus^4^. Although numerous types of O-linked glycosylation can occur on serine and threonine residues, most research has focused on intracellular O-GlcNAc or extracellular “mucin-type” O-GalNAc glycosylation^2,5^.

The term “mucin-type” refers to the mucin family of glycoproteins, where this type of glycosylation is most abundant^6^. Mucins, the primary protein component of mucus, are characterized by dense O-GalNAc glycosylation in their proline-, threonine-, and serine-rich or “PTS” domains^7^. The human proteome includes roughly 20 canonical mucins, which can be further classified as transmembrane or secreted^8^. However, mucin domains are found in a diverse range of glycoproteins, including the T cell immunoglobulin and mucin-domain containing (TIM) family, lubricin, lacritin, and platelet glycoprotein 1bα (GP1Bα)^9–13^. The unique properties of these domains are important for their varied functions in the formation of protective mucus barriers, regulation of proteolytic cleavage, and cell surface adhesion^7,14,15^.

Many cancers feature aberrant expression of different mucins, making them attractive diagnostic and therapeutic targets^15^. Mechanistically, elevated expression of mucins on the tumor surface has been shown to physically preclude immune cell engagement while simultaneously decreasing adhesion to surrounding tissue, facilitating metastatic invasion^16–18^. The structure of the glycans themselves can also play a significant role in pathological mechanisms, as evidenced by the increased expression of tumor-associated carbohydrate antigens (TACAs) on both N- and O-glycans^19–21^. Specific O-glycan structures associated with cancer include the Tn (GalNAc or N1), sialyl-Tn (STn, Neu5Ac-GalNAc or N1A1), and Thomsen–Friedenreich (T, Gal-GalNAc or H1N1) antigens^20^. In fact, experimental tissue models have directly linked malignant phenotypes to the overexpression of truncated O-GalNAc glycans^22,23^.

However, the relationship between mucins and disease is not always well understood. For example, strong evidence exists for both protective and pathological functions of MUC2 in colorectal cancer (CRC)^24–26^. MUC2 is a secreted, gel-forming mucin that assembles into netlike assemblies that form a protective layer on intestinal epithelial cells^27^. Histopathological analyses have shown diminished or even absent MUC2 expression in colorectal tumors compared to normal tissue^25^, and MUC2 knockout mice lacking a protective intestinal mucus layer spontaneously develop colon tumors^26^. On the other hand, mucinous carcinomas of the colon often overexpress MUC2^28^. Mucinous colorectal adenocarcinoma, a distinct CRC subtype, is characterized by mucus content that exceeds 50% of the total tumor volume^29^. Approximately 10-15% of all diagnosed CRC falls into this subcategory^30^, which can be highly invasive and often responds poorly to traditional therapeutics^29,31^. Mucinous carcinomas can also form in other epithelial tissues such as the esophagus, pancreas, breast, and ovary, among others^28,32^; the prognostic significance of mucinous differentiation appears to differ by tumor tissue origin^33^.

The exact molecular mechanisms governing the development and progression of mucinous tumors are poorly understood, primarily due to the analytical challenges associated with studying them^29^. Although glycosylation is essential for their structure and function, no canonical mucin has been sequenced at the glycoproteomic level. In fact, few studies have attempted to characterize both the expression and O-glycan profile of mucins in cancerous tissue; previous attempts at mapping MUC2 O-glycosylation have relied on recombinantly expressed fragments of the full-length protein^34–36^. Liquid chromatography coupled to mass spectrometry (LC-MS) is the technique of choice for most glycoproteomic analyses, as it allows for comprehensive, untargeted detection of proteins in complex samples and can be used to localize glycans to specific amino acids^36,37^. While LC-MS can identify the various glycoforms present in a homogenized sample, such “bulk” analyses lose biologically relevant spatial information. Visualizing specific mucin glycoforms in malignant tissue could therefore improve our understanding of tumor physiology and inform therapeutic design.

Interest in spatial proteomics has exploded in recent years, driven by technological advances that afford imaging of numerous targets in tissue with ever-increasing resolution^38^. Extensive documentation of the high spatial heterogeneity within the tumor microenvironment and its relevance to disease progression has propelled cancer research in new directions^39^. While many techniques have been employed to understand the spatial distribution of mucins in various diseases, all are accompanied by significant drawbacks. Histological dyes represent a simple, relatively inexpensive approach for mucin visualization in tissue. Alcian blue (AB)-periodic acid-Schiff (PAS) staining detects acidic and neutral mucins, respectively, although polysaccharides like glycogen are also stained^40^. Catalytically inactivated mucinases, which are proteases selective for mucins, can be conjugated to reporter molecules for mucin-selective staining, but do not reveal the identities of the underlying mucins^41^. More detailed analyses of specific mucin proteins and glycan structures typically require antibody-based techniques such as immunohistochemistry (IHC)^42,43^. Similarly, recent advances in multiplexed antibody-based imaging, including technologies such as co-detection by indexing (CODEX)^44^ and multiplexed ion beam imaging by time of flight (MIBI-TOF)^45^ have allowed for simultaneous detection of multiple proteins (currently up to roughly 50) with high sensitivity and resolution. Although powerful, such approaches rely on prior knowledge of targets and the availability of well-characterized antibodies against them.

Matrix-assisted laser-desorption ionization mass spectrometry imaging (MALDI-MSI) is a technique that is commonly employed to analyze glycans within preserved biological tissues with high spatial resolution, often in the range of tens of micrometers^46^. This technique is routinely used to map different analytes, including metabolites, lipids, and glycans^47^. The latter is restricted to N-glycans, owing to the availability of the universal N-glycosidase, PNGaseF. To date, no universal O-glycosidase has been characterized, limiting spatial analyses of O-glycans to targeted methods such as lectin staining and IHC.

Mucinases, as described above, are a class of proteolytic enzymes specific for the unique bottlebrush-like structure of mucin domains. We hypothesized here that on-tissue mucinase digestion followed by MALDI-MSI could allow for visualization of mucin glycopeptide distribution. In MALDI-MSI, analytes are typically identified only by their intact precursor mass. This works well for species like N-glycans, whose modular structures limit them to a specific set of masses^46,47^. In contrast, accurate identification of mucinase-generated O-glycopeptides with MS requires tandem mass spectrometry. Given that we previously demonstrated the correlation between high-resolution MALDI imaging of N-glycans and LC-MS analysis of glycopeptides extracted from a serial tissue section in canine glioma^48^, we reasoned that a similar approach could be employed here.

Our proof-of-concept experiments on colon, esophageal, and salivary gland carcinomas demonstrate the technical feasibility of our dual-MS workflow for spatial glycoproteomic analyses. Most of the detected O-glycopeptides were modified by known cancer antigens, and many were found exclusively in benign or tumor tissue sections. Some of these tumor-associated O-glycopeptides were diffusely distributed within the carcinomas, while others localized to well-defined clusters. This work demonstrates that coupled MALDI-MSI and intact glycoproteomics can map the spatial distribution of mucinase-derived O-glycopeptides in tissue with high resolution. Furthermore, our approach enabled sequencing of tumor-associated MUC2, with nearly complete coverage across its two mucin domains. Overall, we present this workflow as a means to identify and visualize mucin O-glycopeptides in archival tissue. Future applications of this technique to large patient cohorts might reveal potential diagnostic and/or therapeutic targets for mucinous carcinomas.

## Results

### MALDI-MSI can map glycopeptides generated by on-tissue StcE digestion

**Fig. 1:**
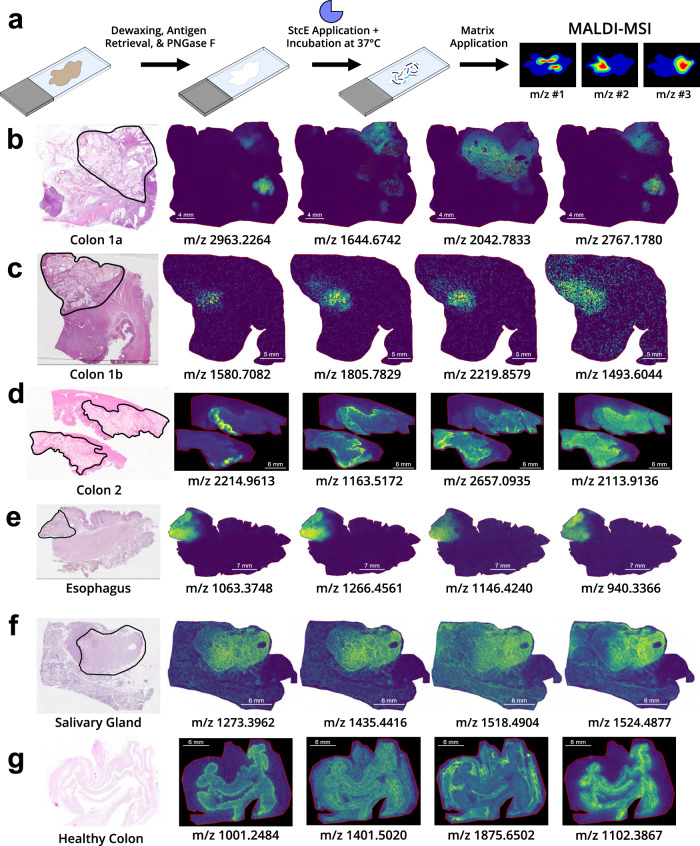
MALDI-MSI of tumor sections treated with PNGaseF and mucinase StcE. **a** FFPE tissue sections were dewaxed, antigen retrieved, and treated with PNGaseF, after which slides were subjected to on-slide digestion with StcE. Following application of CHCA matrix, samples were spatially analyzed with MALDI-MSI. Tumor regions are outlined on H&E-stained tissues and representative MALDI-MSI species are shown for **b** Colon 1a, **c** Colon 1b, **d** Colon 2, **e** Esophagus, **f** Salivary Gland, and **g** Healthy Colon samples. Images in **b**-**g** represent the results from a single preparation of each tissue. Individual MALDI images with corresponding color scales are included in the Source Data.

We analyzed one healthy colon tissue sample and five mucinous carcinomas with our coupled-MS approach: three from the colon, one from the esophagus, and one from the salivary gland. Colon tumors 1a and 1b were resected from the same patient, while all other tissues were collected from different individuals. Detailed morphological descriptions of each tumor can be found in Supplementary Figs. 1–6. Using these tissues, we developed a MALDI-MSI workflow to image StcE-generated glycopeptides (Fig. 1a). Briefly, tissue slides were de-waxed and subjected to low-pH antigen-retrieval, after which digestion with automatic sprayer-applied PNGaseF was performed on all samples. On-slide StcE digestion was followed by matrix application and MALDI-MSI data acquisition. Image resolution was in the range of 40-50 microns, except for Colon 1b, for which the pixel size was approximately 200 µm. For each cancer tissue, imaging experiments revealed numerous species that primarily localized to tumor or tumor-adjacent tissue (Fig. 1b-f). The detection of distinct expression patterns in lower-resolution Colon 1b images highlights the sensitivity of our workflow, indicating tissues can be rapidly screened for such patterns in future work. In contrast, ions detected in the Healthy Colon displayed a more limited range of spatial expression patterns, with many species localized diffusely across the tissue or within the mucosa (Fig. 1g). Nearly all ions detected in the cancerous tissues at high abundance outside of the tumor boundaries were N-glycans, although we identified multiple N-glycans within the boundaries of the salivary gland tumor (Supplementary Fig. 7), showing the compatibility of our workflow with orthogonal enzymatic treatments for multi-omic imaging.

Notably, tumor-localized ions displayed distinct expression patterns even within the diseased tissue (Fig. 1b-f). In Colons 1a, 1b, and 2, certain species localized to dense clusters, while others appeared more evenly distributed across the tumor (Fig. 1b-d). In the Healthy Colon, ions were distributed evenly across the same areas, with no discernable differences in the relative intensity of different species (Supplementary Fig. 8). Ions from the esophageal tumor were generally detected across the entire tumor region, although some were more abundant along the smooth muscle (Fig. 1e). Most species identified in the salivary gland were diffuse across the tumor, with some encroachment into the adjacent parotid tissue (Fig. 1f). Taken together, these results demonstrated that StcE could be used to generate ions in FFPE tissues for MALDI-MSI analysis.

### Optimized LC-MS sample preparation facilitates tumor (glyco)proteome characterization

All slides used for LC-MS experiments were prepared as for MALDI-MSI prior to the matrix deposition step. Details regarding workflow optimization can be found in the Supplementary Text. Briefly, tumor and tumor-adjacent regions within the same tissue section were outlined using a hydrophobic barrier pen, enabling a comparison of the (glyco)proteome between malignant and neighboring tissues. The Healthy Colon was not separated into different regions (Supplementary Fig. 9). We then used a pipette to saturate each tissue region with a concentrated StcE solution and allowed digestion to proceed for approximately 24 h prior to extraction of mucinous glycopeptides.

**Fig. 2:**
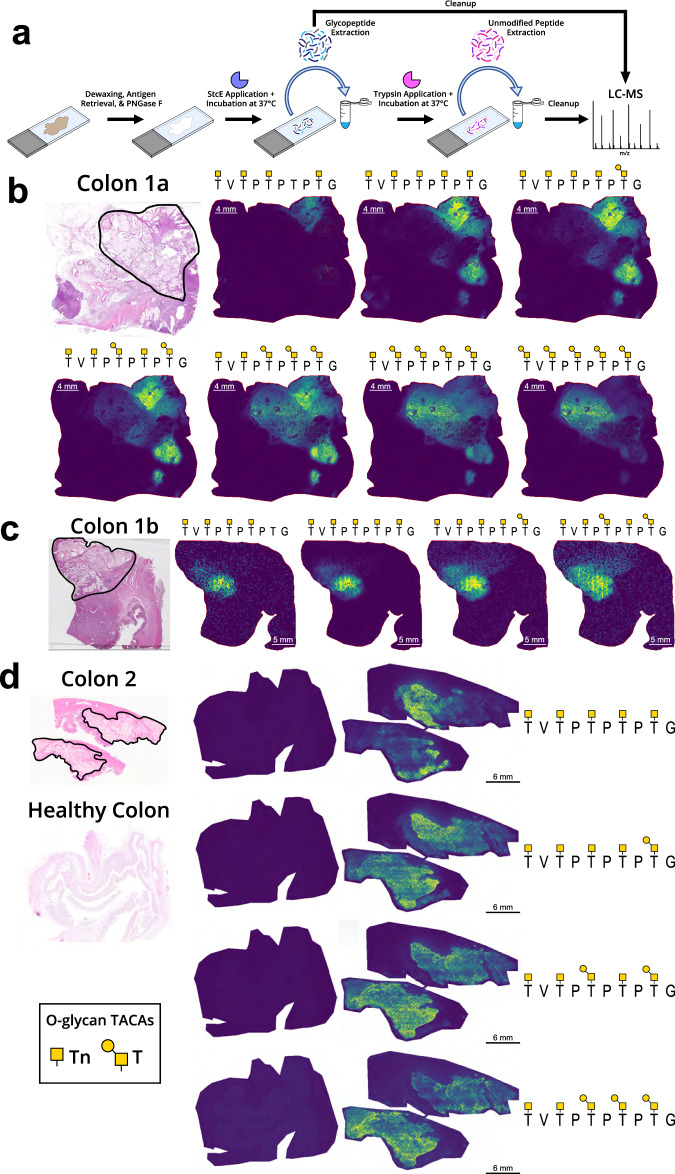
Identification of StcE-derived O-glycopeptides via LC-MS. **a** Serial tissue sections were dewaxed, antigen-retrieved, and PNGaseF-treated. Following rinse steps and on-slide digestion with sprayer-applied StcE, the tissue surface was saturated with pipette-applied StcE to maximize O-glycopeptide generation. After O-glycopeptide extraction, an on-slide trypsin digestion was performed, and unmodified peptides were collected and fractionated with a HILIC-based approach. All peptide samples were then analyzed with LC-MS. **b** The expression profile of MUC2 glycoforms of the backbone TVTPTPTPTG in Colon 1a varies based on relative amounts of the T and Tn antigens, with high levels of the former displaying a more diffuse pattern and the latter more clustered to specific areas. The sequence TVTPTPTPTG is from the second PTS domain in MUC2. **c** MUC2 glycoforms in Colon 1b exhibit a similar spatial pattern as in Colon 1a. **d** The same MUC2 glycoforms shown in (**b**) and (**c**) exhibit a similar expression pattern in Colon 2. When normalized to the same intensity as Colon 2, signal for these glycopeptides in the Healthy Colon is nearly undetectable, indicating highly elevated expression of MUC2 with truncated O-glycosylation in Colon 2. Images in **b**–**d** represent the results from a single preparation of each tissue. Individual MALDI images with corresponding color scales are included in the Source Data.

After extracting glycopeptides, we dried the slides completely before depositing trypsin and digesting for 3–4 h (Fig. 2a). Subsequent analyses of unmodified peptide fractions revealed the comprehensive proteome within each tissue (Supplementary Data 1; *vide infra*), allowing us to curate focused protein databases used to analyze glycopeptide data. This tailored database approach enhanced the sensitivity, accuracy, and speed of glycoproteomic search algorithms by reducing the size of the search space. We manually inspected MS spectra to validate all software-identified glycopeptides in the tumor LC-MS data (Supplementary Data 2). A detailed description of our data analysis workflow can be found in “Methods” (see sections titled “Unmodified Proteome Searches of PHEA-enriched Tryptic Peptides,” “Glycoproteomic Searches with O-Pair,“ and “Glycoproteomic Searches with Byonic”).

Most glycopeptides identified in the colorectal and esophageal tumors were derived from MUC2 (Supplementary Data 2). Overall, we observed a high degree of overlap in the glycopeptides identified in Colons 1a, 1b, and 2. We expected Colons 1a and 1b to be similar in composition as they were resected from the same patient. However, we were surprised by their overlap with Colon 2, which was from a second patient. Nearly 90% of the glycopeptides identified in Colon 2 were shared with Colons 1a and 1b, a result largely driven by the overlap of MUC2 glycoforms, which composed nearly all (approximately 97.1%) of the reported identifications in Colon 2. Furthermore, Colon 2 was prepared by a different lab and analyzed at a much later date than the other colorectal tumors, underscoring the high similarity between these samples as well as the robustness of our workflow.

### Integration of MALDI-MSI and LC-MS data enables visualization of MUC2 glycoforms

Parallel MALDI-MSI experiments revealed visually striking expression patterns of MUC2 glycoforms in the colorectal tissues, particularly those with the backbone sequence TVTPTPTPTG (Fig. 2b-d). These sequences were decorated with a combination of Tn and T antigens, whose structures are defined as a single GalNAc or Galβ1-3GalNAc, respectively. In Colon 1a, the Tn-only glycoform clustered to two regions within the tumor, adjacent to fibrotic or non-mucinous malignant colon tissue. Glycoforms with varied backbone sequences decorated with both T and Tn antigens were more diffusely distributed across the mucinous tumor area, and T-only glycoforms localized closer to the tumor edge opposite that of the Tn-only clusters, adjacent to adipose tissue (Fig. 2b). We observed the same glycoform-specific patterning in Colons 1b and 2, but not in the Healthy Colon tissue (Fig. 2c, d, Supplementary Fig. 10). Importantly, glycopeptides with different backbone sequences shared the same glycosylation-dependent localization in the carcinoma tissues, with T and Tn antigen distribution discerning between different tumor regions (Supplementary Figs. 11, 12, 13, and 14). Notably, StcE-derived O-glycopeptides were only identified within tumor boundaries in Colons 1a, 1b, and 2. This result suggests that our workflow might aid the identification of tumor-specific biomarkers if applied to a sufficiently large set of patient-derived samples. We also detected tumor-associated species in these same samples with MALDI-MSI that could not be identified with LC-MS data, although the total number of spectra in this category was relatively low (Supplementary Fig. 15).

Because data for Colon 2 and the Healthy Colon were acquired on the same instrument, we compared the relative abundance of MUC2 glycoforms in these tissues by normalizing the MS intensity. Although MUC2 glycopeptides with truncated glycosylation were detected in the Healthy Colon, signal for these ions was negligible when directly compared to their abundance in Colon 2 (Fig. 2d). This disparity suggests that truncated, non-sialylated O-glycoforms of MUC2 were overexpressed in the colorectal tumors compared to healthy tissue. In the future, integrating our results into multi-omic analyses of expanded patient cohorts could potentially link specific, overexpressed MUC2 O-glycoforms to carcinogenic processes in mucinous colorectal tumors. O-glycopeptide epitopes might then serve as diagnostic or prognostic biomarkers and possibly therapeutic targets. However, we should note that we cannot fully rule out the possibility that a portion of the detected signal corresponds to immature, Golgi-associated MUC2.

### StcE facilitates extensive O-glycosite mapping of PTS regions in tumor-associated MUC2

**Fig. 3:**
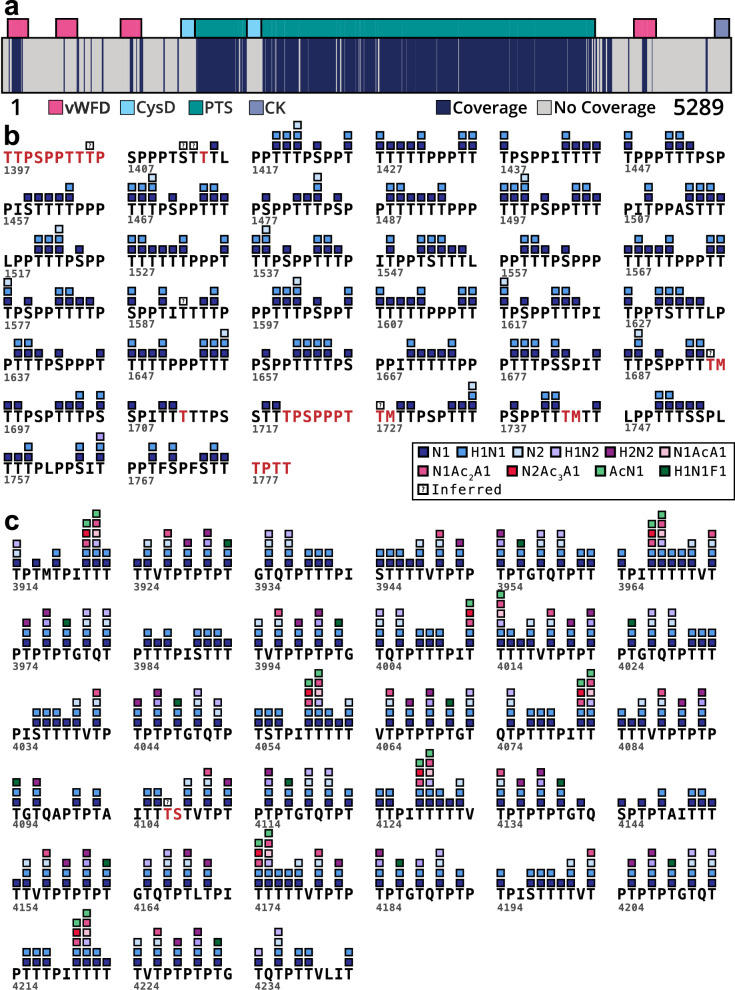
LC-MS analyses of Colon 1a, Colon 1b, Colon 2, the Healthy Colon, and the esophageal tumor yielded high sequence coverage of MUC2 and revealed domain-dependent glycosylation patterns. **a** Nearly complete coverage of MUC2’s mucin domains was obtained in LC-MS analyses, while proteomic coverage outside of the PTS regions was more limited. **b** Truncated O-glycans were confidently localized to residues in the first PTS domain, shown here in its entirety. **c** More varied O-glycan structures were localized to residues in the second PTS domain, a portion of which is shown here. “Inferred” indicates glycosites identified based on the cleavage preference of mucinase StcE. Red text indicates residues for which we did not obtain sequence coverage.

To calculate MUC2 sequence coverage, we compiled all backbone sequences from identified glycopeptides across all tumor samples (and the Healthy Colon), as well as any unmodified peptides from tryptic digests. Because bottom-up proteomic methods cannot determine the exact origin of perfect repeat sequences from the same protein, our calculation assumed repeat glycopeptides were derived from all possible locations. Using these assumptions, we obtained 57.3% sequence coverage (3030/5289 residues) across the full MUC2 sequence, and 14.7% coverage (365/2477 residues) outside of the mucin domains. The limited proteomic coverage outside of the mucin domains likely stemmed from the high number of disulfide-bonded cysteines combined with the lack of reduction and alkylation steps in our workflow. On the other hand, we obtained 94.8% coverage (2665/2812 residues) over its two PTS domains (Fig. 3a), a particularly exciting result as no canonical mucin has yet been characterized at the glycoproteomic level. We envision that the availability of detailed glycan maps will enable in-depth investigation into the effects of glycosylation on the higher order structure and biophysical properties of mucins, particularly in the context of disease^10,49–51^. Glycoproteomic sequencing of MUC2 extracted from an expanded patient cohort may also reveal disease-associated glycoforms that could serve as sensitive diagnostic or prognostic biomarkers.

Most of the MUC2 glycopeptides identified in the colorectal tumors were decorated with a combination of T and Tn antigens, although we detected evidence indicating the presence of core 2, fucosylated, and sialylated O-glycans that could not be localized to specific Ser or Thr residues (Supplementary Fig. 16a and Supplementary Data 2). We detected evidence for a single sialylated O-glycopeptide in the first PTS domain, and more complex fucosylated and acetylated structures in PTS 2, although we were unable to pinpoint the exact glycosites harboring these structures (Supplementary Fig. 16c-d). In the first, shorter PTS domain, we confidently localized only Tn and T antigen structures, with the exception of one core 2 structure mapped to a single site (Fig. 3b). We localized these and additional, more extended O-glycan structures on peptide sequences from the second PTS domain (Fig. 3c). We suspect that protein structural differences between the two PTS domains could explain this disparity in glycosylation. Mucin domains are enriched in proline; in fact, mucins are proposed to have evolved from secreted proline-rich proteins in different animal lineages^52^. Most of the prolines in PTS 1 are found in clusters of two or three, while virtually all of the prolines in PTS 2 are separated by at least one other amino acid. The frequency and distribution of proline within the backbone can have an enormous impact on protein tertiary structure and flexibility^53^. The distinct proline distribution patterns in PTS 1 and PTS 2, therefore, suggest that these domains adopt divergent structures. If so, these conformational differences might influence glycosyltransferase accessibility and activity, ultimately leading to disparate glycosylation profiles between the two PTS domains. Previous work by Nason et al. observed mostly core 1 O-glycans on recombinant PTS 1 constructs, compared to a mix of core 1 and 2 structures on sequences from PTS 2 and other mucins, an interesting finding that also indicates possible structural variation along the MUC2 backbone^34^.

Additionally, some backbone sequences we attributed to MUC2 are technically present in other proteins. We considered StcE’s known cleavage preferences, total proteome data (from unmodified peptides), and cellular compartment annotations to make assignments in these cases. For example, because our MS acquisition method cannot distinguish Leu from Ile, the sequence TPITT could, in theory, be TPLTT. Both of these sequences map to numerous proteins; however, many of these can be excluded from consideration as they are intracellular proteins and thus highly unlikely to be glycosylated with O-GalNAc glycans. TPLTT is repeated three times in the canonical MUC16 sequence and twice in MUC17, but only in MUC17 is the TPLTT sequence flanked by residues consistent with StcE’s cleavage motif. MUC17 was not identified in the unmodified tryptic data, however. On the other hand, MUC2 is highly abundant in tryptic data and the sequence TPITT is repeated 69 times in the canonical MUC2 sequence flanked by residues corresponding to StcE cleavage. Taken together, we felt confident assigning MUC2-derived TPITT as the identity of the glycopeptide.

### Gastrointestinal tumors lack hyper-sialylation typically associated with carcinomas

In all tumor samples and the Healthy Colon, we identified glycopeptides decorated with a variety of O-glycan structures (Supplementary Figs. 17a-e, 18). In the colorectal and esophageal tumor data, the distribution of identified glycoforms largely resembled that of MUC2 (Supplementary Fig. 19a-d). Neu5Ac-containing glycans were not particularly abundant in the colon and esophageal tumors, evidenced by ion chromatograms for protonated Neu5Ac and its water loss at *m/z* 292.1027 and 274.0921, respectively (Supplementary Fig. 20). This result was surprising, as cancer is typically associated with hyper-sialylation of N- and O-glycans^20^. We did detect some sialyl T-containing MUC2 glycopeptides in the colorectal tumors with LC-MS and MALDI-MSI, although at a much lower intensity than non-sialylated versions of the same species (Supplementary Fig. 21 and Supplementary Data 2). Some data have suggested that StcE does not readily cleave mucins heavily decorated with STn^34^, however, the scarcity of sialyl-T and other Neu5Ac-containing structures in the MUC2 data suggests that StcE’s cleavage restrictions did not skew our glycoproteomic results.

Sialic acids are prone to in-source fragmentation (ISF) in both electrospray ionization (ESI) and MALDI^54^, which might have contributed to poor detection of sialylated glycopeptides. However, because the Neu5Ac fingerprint ion signal was robust in the salivary gland tumor LC-MS data (Supplementary Fig. 20), we concluded that the unexpectedly low sialic acid content in other samples was likely not a technical artifact. Regardless, future studies may benefit from an on-slide derivatization step during sample preparation, which can reduce sialic acid lability during ionization and even distinguish its different glycosidic linkages^55^.

**Fig. 4:**
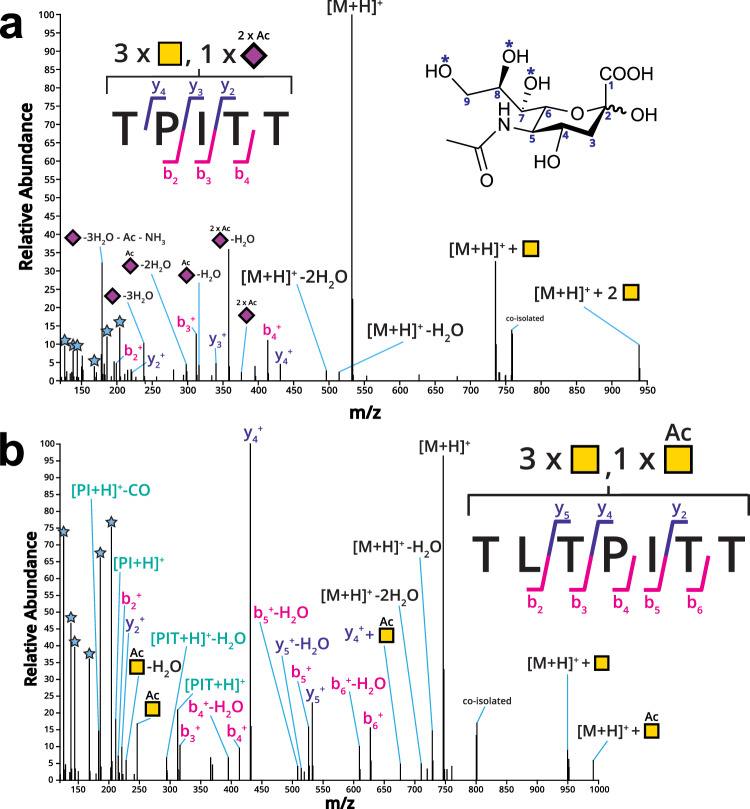
Spectral evidence for acetylated glycans on MUC2 sequences. **a** Representative higher-energy collision dissociation (HCD) spectrum for a glycoform of TPITT containing di-O-acetylated Neu5Ac at *m/z* 758.8309 (*z* = 2, MS1 error = +1.87 ppm). Fingerprint ions for Ac_2_Neu5Ac are observed in the low-mass region, along with HexNAc fingerprint ions denoted by blue stars. [M + H]^+^ represents the mass of the naked peptide backbone. The inset shows the structure and numbered carbons of Neu5Ac, with asterisks indicating the most common sites of O-acetylation on the hydroxyl moieties of C7, C8, and/or C9. This specific spectrum is from Colon 1b, region 2 (tumor-adjacent tissue) data. **b** HCD spectrum for the sequence TLTPITT containing a putative AcGalNAc O-glycan at *m/z* 800.8820 (z = 2, MS1 error = +1.87 ppm). Fragments corresponding to the lone AcGalNAc as well as the y_4_^+^ and peptide backbone ions with AcGalNAc are observed. Internal fragments containing Pro, Ile, and Thr (from the sequence TPI or PIT) were also detected in the low-mass region. Corresponding *m/z* values and respective errors for acetylated species in (**a**) and (**b**) are listed in Supplementary Tables 1 and 2. All fragment *m/z* values and relative intensities within each spectrum are provided in the Source Data.

We explored possible modifications on Neu5Ac that could mask its presence in our data. Neu5Ac is often mono-, di-, or tri-acetylated in colon-derived glycans, and previous studies have linked loss of Neu5Ac acetylation with the development of colorectal cancer^56^. O-glycopeptides decorated with di- and tri-acetylated Neu5Ac on more extended core structures were highly abundant in the Healthy Colon tissue (Supplementary Figs. 18, 19f, 22). Unlike the Healthy Colon tissue, LC-MS analyses of Colons 1a/b and 2 revealed only low levels of mono- and di-O-acetylated Neu5Ac-containing glycans, consistent with downregulation of this modification with disease progression (Supplementary Figs. 23 and 24). The Ac_2_Neu5Ac fingerprint ion signal (*m/z* 358.1131 and 376.1237) exceeded that of AcNeu5Ac (*m/z* 316.1026 and 334.1132) across Colon 1b and in the tumor-adjacent region of Colon 1a, with the opposite pattern observed in the tumor region (Supplementary Figs. 23–25). Glycans containing AcNeu5Ac and Ac_2_Neu5Ac could be localized on peptides from the second PTS domain of MUC2 (Fig. 4a, Supplementary Figs. 26a, b, Supplementary Table 1, and Supplementary Data 2). We were unable to detect glycopeptides decorated with AcNeu5Ac or Ac_2_Neu5Ac in the tumor tissues via MALDI-MSI, likely due to in-source fragmentation or possibly lower sensitivity. Possible biological explanations for the relative paucity of sialylated O-glycans in the gastrointestinal tumors should be investigated in greater detail.

Previous work characterizing human MUC2-derived O-glycans determined that many glycans with the same composition (minus Neu5Ac O-acetylation) were core 3 (Supplementary Fig. 18)^57^. On the other hand, assays using StcE to digest mucin tandem repeat reporters with defined O-glycans found that core three structures effectively blocked proteolysis^34^. In this same study, though, efficient cleavage of core 3-decorated mucin reporters was achieved at higher concentrations (approximately 1 µg/mL)^34^. Given that we saturated each tissue surface with much more concentrated StcE (approximately 50 µg/mL), it seems likely that any core 3-decorated mucins would be cleaved under these conditions. Future work should integrate in-depth glycomic analyses on the same samples for confident structural assignments.

### Glycoproteomic analyses of colorectal tumors reveal O-acetylated GalNAc on MUC2

Spectral evidence from Colons 1a and 1b suggested the presence of an additional acetyl moiety on GalNAc (Fig. 4b, Supplementary Figs. 27a, b, and Supplementary Table 2). This included fragments in HCD spectra corresponding to the HexNAc fingerprint ions at *m/z* 204.0867 and *m/z* 186.0761 shifted by the mass of an acetyl group (+42.0106 Da), resulting in glycan fragments with theoretical *m/z* 246.0973 and 228.0867 (Fig. 4b, Supplementary Fig. 27a, and Supplementary Table 2). For all glycopeptides identified with this modification, the naked peptide backbone was detected at the expected *m/z* in HCD spectra. This observation ruled out potential acetylation of the peptide itself, instead supporting acetylation on one of the glycans. Importantly, we detected LC-MS evidence for “AcGalNAc” only in the colorectal tumor regions. MALDI-MSI results for Colon 1a included a heat map for an *m/z* corresponding to the mass of TVTPTPTPTG decorated with four Tn antigens and one AcGalNAc, the presence of which was identified by LC-MS (Supplementary Fig. 28a and Supplementary Data 2). Although the MALDI-MSI intensity of this ion was weak, its distribution overlapped with that of Tn-only MUC2 glycopeptides in the tumor region (Supplementary Figs. S28a-b). While the amine nitrogen in GalNAc could theoretically accommodate a second acetyl group, we suspect the additional acetylation is O-linked through one of the hydroxyl moieties, similar to sialic acid acetylation. Although others have speculated about its existence, to our knowledge, experimental evidence for endogenous O-acetylated GalNAc in O-glycans has not been reported in the literature. Additional studies should thoroughly assess whether AcGalNAc is a true biological phenomenon or a technical artifact.

### LC-MS identifies additional mucins in colon samples

Our LC-MS analyses revealed the presence of additional canonical mucins beyond MUC2, including MUC1, MUC5AC, MUC12, and MUC18 (Supplementary Data 1 and 2). Overexpression of MUC1 is estimated to occur in over half of all cancers^15^, and we detected unmodified tryptic peptides from this transmembrane mucin in Colons 1a and 1b as well as the esophageal tumor (Supplementary Data 1). However, we did not identify any glycopeptides from MUC1. StcE cleaves mucins at T/S*XT/S” motifs, in which a glycosylated Ser or Thr is followed by any amino acid (X) and then another Ser or Thr^58^. Cleavage occurs C-terminally to the “X” residue, and StcE can accommodate glycosylation at any position within the motif. Although MUC1 contains very few putative StcE cleavage sites in its tandem repeats, StcE-sensitive sequences can be found outside this region. Because there are only single copies of the potential glycopeptides produced by StcE, they are less likely to produce sufficient signal for detection by LC-MS. Therefore, we suspect the lack of MUC1 glycopeptides in Colons 1a and 1b stems from their relatively low abundance compared to MUC2 in these samples.

Even though we were unable to detect MUC1 via LC-MS in Colon 2 and the Healthy Colon, immunohistological staining of MUC1 in these samples revealed weak expression in both tissues (Supplementary Fig. 29a, b). In contrast, staining with the catalytically inactive StcE^E447D^, which binds any mucin domain-containing proteins in tissue, revealed an inverse expression pattern (Supplementary Fig. 29a, b). While MUC1 staining was strongest at the outer edges of the mucosa, StcE^E447D^ staining was detected throughout the mucosa and submucosa (Supplementary Fig. 29a, b). These data suggest StcE used to prepare LC-MS samples could not favorably bind to MUC1, in contrast to previous work demonstrating StcE’s activity on MUC1^41,58^. This observation might be explained by suboptimal binding conditions in a complex tissue or that our current antigen retrieval step does not adequately expose the MUC1 backbone for binding and cleavage by StcE.

MUC5AC, like MUC2, is a secreted, gel-forming mucin that is often aberrantly expressed in colorectal cancer^24,25^. Normally, MUC5AC is only found in the respiratory and upper digestive tract, and its presence in colorectal cancer is associated with better patient outcomes^24^. We identified both unmodified tryptic peptides and glycopeptides from MUC5AC in Colon 2, but not in Colons 1a or 1b. As with MUC2, the O-glycans we detected on MUC5AC glycopeptides were primarily truncated structures like the T and Tn antigens. Future work should investigate how MUC5AC glycosylation regulates its function and contribution to colon cancer.

The transmembrane mucin MUC12 was identified using unmodified tryptic peptides and one glycopeptide in Colons 1a and 1b (Supplementary Data 1). While expression of MUC12 is normally high in the gastrointestinal tract, particularly in the colon, its downregulation is associated with colorectal cancer and ulcerative colitis^59,60^, which could help explain why we were only able to validate a single glycopeptide from this transmembrane mucin (Supplementary Data 2). MUC18, also known as melanoma cell adhesion molecule (MCAM) or CD146, was identified in all colon samples as well as the esophageal and salivary gland tissues using only unmodified tryptic peptides. This transmembrane mucin is relatively small and contains few putative StcE sites, potentially explaining the lack of glycopeptides in our results.

### LC-MS allows for the detection of Lewis antigen glycoforms in the esophageal tumor

Despite its origination in a completely different location within the gastrointestinal tract, the esophageal tumor displayed significant glycoproteomic overlap with the colorectal cancers, consistent with the “intestinalization” phenotype often observed in the progression from healthy tissue to cancerous lesions^61^. Many of the MUC2 glycopeptides decorated with a combination of the T and Tn antigens identified in Colons 1a, 1b, and 2 were similarly detected in the esophageal tumor. Our results also revealed MUC5AC glycopeptides shared between the esophageal tumor and Colon 2. This tumor was notably smaller than those in the colorectal and salivary gland tissues, and as a result, the amount of recovered material and subsequent LC-MS HexNAc signal was lower in the esophageal samples compared to the others (Supplementary Fig. 8a). Many glycopeptide species were too weak for MS2 selection and were identified by precursor mass and retention time (Supplementary Data 2). Despite strong HexNAc ion signal in the esophageal tumor (Supplementary Fig. 8a), relatively few spectra could be confidently assigned to glycopeptides from specific proteins using search algorithms. By manually extracting and analyzing spectra with intense HexNAc fingerprint ions, we identified multiple dipeptides with the sequence TI/TL or IT/LT. These dipeptides were highly abundant and decorated with a variety of structures, including H2N2F1 and H2N3F2 (Supplementary Fig. 30a, b). The latter of these may correspond to either the Lewis b or Lewis y blood group antigens, which are indistinguishable with our MS/MS acquisition method. While these species were most likely cleaved from MUC2 or MUC5AC, we cannot confidently confirm this due to the lack of unique peptide sequences. Additionally, much of the HexNAc ion signal could be attributed to free N-glycans released during PNGaseF treatment (Supplementary Fig. 31a, b). Numerous tumor-associated ions were detected in the esophageal tissue by MALDI-MSI, but we were unable to identify any of these species in the associated LC-MS data (Supplementary Fig. 32). Conversely, although many MUC2 O-glycopeptides were identified in the esophageal tumor LC-MS data, we were unable to detect any by MALDI-MSI.

We identified a single unmodified peptide from MUC5B, a secreted, gel-forming airway mucin (Supplementary Data 1). The backbone sequence of MUC5B contains numerous Ser and Thr within TXT motifs, so the relative paucity of MUC5B glycopeptides in the esophageal data was surprising. Like MUC2, this secreted mucin assembles into complex, higher-order structures that may render the protein backbone inaccessible to StcE^62^.

### Versican dominates the glycoproteome of the salivary gland tumor

In the salivary gland tumor, nearly all identified glycopeptides originated from the core protein of versican, a mucinous chondroitin sulfate proteoglycan (Supplementary Fig. 19d and Supplementary Data 2). Versican glycopeptides were also detected in other samples, albeit very few at low abundances (Supplementary Data 2). Various cancers are associated with versican overexpression^63^, and cleavage of the core protein by tissue-resident and tumor-associated proteases may contribute to tissue invasion and metastasis^64^. Core 1 and core 2 structures with and without sialylation were localized on versican glycopeptides, although most were decorated with only T antigens (Supplementary Fig. 19e). Surprisingly, very few versican glycopeptides displayed the Tn antigen, in stark contrast to MUC2 results in the other tumors (Supplementary Figs. S19a-b). Interestingly, many versican peptide sequences corresponded to non-StcE proteolytic cleavage at the N-terminus (*n* = 25, 9.8%), C-terminus (*n* = 110, 43.3%), or both (*n* = 9, 3.5%) (Supplementary Data 2). We detected only low levels of non-specific cleavage in other samples, suggesting degradation of versican by endogenous enzymes prior to tissue preservation^65^. Unmodified peptide searches identified multiple proteases and peptidases in this tissue (Supplementary Table 3). Whether O-glycosylation influences versican proteolysis and tumor ECM remodeling should be explored in greater detail.

## Discussion

In summary, we developed a dual-MS workflow integrating LC-MS and MALDI-MSI data to spatially analyze StcE-derived O-glycopeptides in FFPE tissue. Importantly, MALDI-MSI data were acquired in two different labs on three different instruments with varied resolution, demonstrating the robustness of this technique for imaging intact O-glycopeptides. Our results revealed glycoform-dependent expression of MUC2 glycopeptides in three different mucinous colorectal tumors from two different patients. Specifically, we observed striking differences in glycoform localization depending on the abundance of T and Tn antigens, with the former diffusely expressed across the tumor mass while glycopeptides decorated with the latter were more punctate in their distribution. These truncated O-glycan structures are strongly associated with numerous malignant processes, and their relative paucity in healthy tissue has generated strong interest in their use as potential biomarkers or therapeutic targets^23^. In fact, previous work using an in vitro skin model showed that cancer cells with O-glycosylation limited to Tn and STn antigens exhibited a significantly more invasive phenotype than cells decorated with wild-type O-glycosylation^22^. Interestingly, the O-glycopeptides extracted from the colorectal and esophageal tumors were largely unsialylated, an unexpected result as cancer is typically associated with hyper-sialylation. We detected abundant sialylation in the healthy colon and salivary gland tumor, ruling out technical limitations as the cause of this observation. Future work should investigate the prevalence and potential role of hypo-sialylation in mucinous carcinomas.

The high sequence coverage we obtained in LC-MS analyses enabled the construction of an in-depth O-glycan map of endogenous, full-length MUC2. Although most of the localized O-glycans were the Tn or T antigen, we also identified core 2 structures, mono- and di-acetylated STn-containing glycans, and O-acetylated GalNAc. Previous attempts to characterize MUC2 at the glycoproteomic level relied on recombinantly expressed fragments of the full-length protein, in some cases from cell lines engineered to produce more homogenous O-glycosylation to facilitate sequencing efforts^34,35^. In contrast, our glycoproteomic sample preparation enabled direct analysis of endogenous, patient-derived MUC2. This aspect is a significant advantage as many immortalized cell lines do not always fully recapitulate more complex glycosylation patterns observed in vivo^66^. As a result, our data is more relevant to understanding specific disease processes in humans.

While this work represents a significant milestone in mucin glycobiology, we emphasize that in-depth characterization of full-length MUC2 and other canonical mucins is unlikely to become routine in the near future due to persistent technical obstacles. The software used in this work provided a helpful starting point in our analyses, but the lack of search algorithms that can accurately and reliably localize multiple O-glycan structures on a given peptide remains the most significant bottleneck in such work. Manual assessment of all glycopeptide identifications in the LC-MS data was an intensive, months-long effort. The presence of numerous possible O-glycosites, evidence for multiple glycoforms in the same or additional chromatographic peaks, and isobaric sequences from the PTS domains of MUC2 are just a few examples of mucin-related challenges we overcame in this work. This work was feasible partly due to the predominance of truncated O-glycans; a dataset dominated by extended O-glycan structures would have been substantially more complex and most likely intractable.

Matching LC-MS identifications to MALDI-MSI data was another hurdle requiring significant trial and error on our part. Although vendor software facilitated the extraction of ion intensity maps for a given list of precursor masses (see “Assignment of Identified Glycopeptides to MALDI-MSI Spectra” in “Methods”), many of the exported images were simply noise. This necessitated visual inspection of all extracted images (approximately 300–400) to remove low-quality data. However, the detection of glycoform-specific expression patterns was a serendipitous result of this process, underscoring the importance of manually reviewing raw data to enhance the value of more sophisticated computational approaches.

Although our results demonstrate the potential of our glycopeptide imaging workflow for studying the tumor microenvironment, important limitations of this approach should be considered in future efforts. The small number of analyzed tissues was suitable for this proof-of-concept work, but sufficiently large sample sizes are critical for evaluating the biological and clinical significance of glycopeptide expression patterns. One of the major technical limitations of this approach was the relatively low sensitivity of MALDI-MSI compared to LC-MS. Another drawback of our approach is that isobaric MUC2 glycopeptides detected by LC-MS could not be distinguished by MALDI-MSI, as MS2 spectra were not acquired with these instruments. For example, LC-MS analyses identified glycoforms of TTPSPPTI, TPSPPITT, TPITPPTS, and TVTPTPTP, all of which have the same theoretical monoisotopic mass. For these cases, we have indicated all possible backbone sequences when assigning glycopeptides to MALDI-MSI data (Supplementary Figs. 8 and 17).

Lastly, when employing glycoproteomic search algorithms to analyze our LC-MS data, we used O-glycan databases with structures frequently observed in cancer. As such, it is possible that we failed to identify glycopeptides decorated with less common structures or modifications. The previously unreported O-acetylated GalNAc structure we detected was found through manual de novo glycopeptide sequencing of spectra that had been incorrectly assigned to other glycopeptides by software. Future work would ideally include a separate glycomics analysis of each tissue to address this limitation. Glycomic data could be used to generate a tailored glycan database to be used in glycoproteomic searches, similar to how we constructed custom protein databases by analyzing unmodified tryptic peptides. Additionally, future work should assess the performance of our workflow when applied to unfixed or fresh-frozen tissue. Samples prepared with these preservation techniques are generally considered superior to FFPE tissue in retaining protein and PTM integrity, thus, we would expect to observe an improved depth of analysis in tissues that were not fixed.

Overall, we present a powerful method to image and sequence cancer-associated mucin glycopeptides in situ. We also hope to assess the relative frequency of the distinct Tn to T antigen expression patterns detected in the colon samples by significantly expanding our patient cohort. Further interrogation of glycopeptide expression patterns in numerous clinical specimens from different disease stages may provide useful insights and drive biological hypotheses in cancer research. We anticipate that this workflow can be applied to many cancers, tissues, and/or pathologies and will prove useful to the broader glycobiology, cancer biology, and mass spectrometry fields.

## Methods

### Tissue acquisition, fixation, and sectioning

All archival tissue samples were obtained in compliance with relevant ethical and legal regulations. A total of four formalin-fixed, paraffin-embedded (FFPE) carcinoma tissue blocks (Colon 1a, Colon 1b, Esophagus, and Salivary Gland) were obtained from the Medical University of South Carolina’s Hollings Cancer Center Biorepository. Two additional FFPE tissues (Colon 2 and Healthy Colon) were obtained through Stanford University. No identifiable patient information was provided to the investigators, and the use of all tissues was approved by the Institutional Review Board at the Medical University of South Carolina (Colons 1a and 1b, Esophageal, and Salivary Gland tissues; protocol 17669) or Stanford University (Colon 2 and Healthy Colon; protocol IRB-46646). Colon 1a and Colon 1b were derived from the same donor, while the Esophagus and Salivary Gland samples were obtained from two additional patients. A pathologist annotated the boundaries of the tumor and adjacent non-malignant regions for each. Tissues were sectioned onto ITO conductive slides at thickness of 5 µm for further sample preparation. Due to the destructive nature of the sample preparation and analysis, these specific tissues are no longer available for additional studies.

### Tissue preparation for MS experiments

His-tagged mucinase StcE was expressed and purified as previously described^58^ using a pET28 plasmid kindly provided by the Bertozzi laboratory. All solvents for slide preparation were purchased from Fisher Scientific. Tissue slides were prepared as described previously^67^. Slides were heated, tissue side-up, at 60 °C for 1 h. After dewaxing and rehydration of tissue slides, antigen retrieval was performed in citraconic anhydride buffer, pH 3, for 30 min in a decloaking chamber set to 95 °C. Following a water wash, slides were dried in a desiccator. A total of 15 passes of 0.1 µg/µL PNGaseF PRIME (N-Zymes Scientific, Doylestown, PA) was applied to slides for N-glycan release using an M5 Sprayer (HTX Technologies, Chapel Hill, NC) with the following parameters: 25 µL/min flow rate, 1200 mm/min velocity, 3-mm offset, 10 psi, and 45 °C. Deglycosylation in prewarmed humidity chambers proceeded for 2 h at 37 °C, after which tissues were H&E stained and scanned. Cover slips were removed, then washed sequentially in 10 mM Tris pH 8.5 and citraconic anhydride pH 3 buffer, then water to remove stain, and dried completely in a desiccator. The M5 sprayer then applied mucinase StcE using the same parameters as above. StcE digestion in the same prewarmed humidity chambers proceeded overnight at 37 °C and slides were dried in a desiccator prior to further preparation for MALDI-MSI or LC-MS analyses.

### MALDI-MSI matrix application

A matrix of 7 mg/mL CHCA in 50%ACN/0.1% TFA was applied to slides with the M5 sprayer at 10 psi and 79 °C using a flow rate of 100 µL/min, velocity of 1300 mm/min, and a 2.5-mm offset. Slides were stored in a desiccator until MALDI-MSI acquisition.

### MALDI-MSI data acquisition and processing

Colon 1a, the esophageal tumor, and the salivary gland tumor were analyzed with a timsTOF fleX MALDI-QTOF mass spectrometer (Bruker Corporation, Billerica, MA). The following parameters were used: mass range *m/z* 700–4000 (Colon 1a) or *m/z* 500–3000 (Esophagus and Salivary Gland), positive ion mode, 20–25 µm laser spots, 300 shots per pixel, and 40-µm raster. Colon 2 and the Healthy Colon tissue were analyzed with a timsTOF fleX MALDI-2 mass spectrometer (Bruker Corporation, Billerica, MA) using the following parameters: mass range m/z 500-3000, positive ion mode, 20–25 µm laser spots, 300 shots per pixel, and 50-µm resolution. Colon 1b was analyzed with a SolariX dual-source 7T MALDI-FTICR mass spectrometer (Bruker). Settings were as follows: mass range *m/z* 689–2500, positive ion mode, 20–25 µm laser spots, 300 shots per pixel, and 40-µm raster. Raw imaging data were processed in SCiLS Lab version 2024b Pro (Bruker Corporation, Billerica, MA), and O-glycopeptide spectra were normalized to total ion count.

### Additional mucinase digestion and glycopeptide extraction for LC-MS

LC-MS grade water (Thermo Scientific, 51140), acetonitrile (ACN) (Honeywell, LC015), methanol (MeOH) (Fisher Scientific, A456), and formic acid (FA) (Thermo Scientific, 85178) were used unless otherwise noted. Slide humidity chambers were prepared by placing dish sponges saturated with Milli-Q water (MicroPure UV/UF, Thermo Scientific, 50132370) in the bottom of airtight plastic containers, then pre-warming at 37 °C for at least 1 h. The tumor and adjacent tumor-adjacent tissue regions were outlined using a hydrophobic barrier pen (Sigma Aldrich, Z377821 or Z672548), then dried for approximately 5 min. A 0.05 mg/mL solution of StcE in fresh, 50 mM ammonium bicarbonate (AmBic) (Honeywell Fluka, 40867) was deposited via micropipette to completely cover each outlined region. Slides were placed in pre-warmed humidity chambers and incubated at 37 °C for 13–23 h.

After removing slides from the humidity chambers, the remaining solution from each region was collected via pipette into separate 1.5 mL Eppendorf tubes, taking care not to touch tissue surfaces with pipette tips. Glycopeptides were collected into the same tubes after extraction with three different solvents: 0.1% FA in water, 4:1 ACN:0.1% FA in water, and 7:3 MeOH:0.1% FA in water^48^. Using the same volume as StcE digestion, each solvent (in the order listed) was pipetted up and down ten times on the surface of the slide. This process was performed twice for each of the solvents, for a total of six extractions.

After glycopeptide extraction, slides were allowed to dry in a chemical fume hood. A 0.01 mg/mL solution of sequencing-grade trypsin (Promega, V5113) in 50 mM AmBic was applied to slides via micropipette, using the same volumes as the mucinase digestion. Slides were placed in humidity chambers and incubated at 37 °C for 4-5 hours before removing tryptic peptides using the extraction method described above. All samples were taken to dryness in a vacuum centrifuge and stored at −20 °C until further use.

### Glycopeptide sample preparation for LC-MS

Glycopeptide samples were resuspended in 100 µL of 50 mM ammonium bicarbonate, after which the approximate protein concentration was measured by NanoDrop One Microvolume UV-Vis Spectrophotometer (Thermo-Fisher). Samples were acidified with 1 μL of neat FA. Desalting was performed using 10 mg Strata-X 33 µm polymeric reversed phase SPE columns (Phenomenex, 8B-S100-AAK). Each column was activated using 1 mL of ACN, equilibrated with 1 mL of 0.1% FA, pre-eluted with 1 mL of 0.1% FA in 40% ACN, and then re-equilibrated with 1 mL of 0.1% FA. Samples were added to the column and de-salted using two 150-µL rinses of 0.1% FA. The columns were transferred to clean 1.5 mL tubes, and peptides were eluted with two 150 µL additions of 0.1% FA in 40% ACN. Samples were taken to dryness in a vacuum centrifuge (LabConco), resuspended in 7–10 µL of 0.1% FA, and transferred to HPLC vials for LC-MS analysis.

### Tryptic peptide sample fractionation and preparation for LC-MS

Tryptic peptide samples were resuspended in 100 μL of 50 mM ammonium bicarbonate, after which the approximate protein concentration was obtained via NanoDrop One. Samples were acidified by adding 1 μL of neat FA, desalted as above, and taken to dryness.

To obtain deeper proteome coverage, peptides were fractionated with hydrophilic interaction chromatography (HILIC)-based solid-phase extraction (SPE) using 12-μm, 300-Å PolyHYDROXYETHYL Aspartamide™ (PHEA) (PolyLC®, BMHY12-03). We constructed PHEA-packed SPE columns based on the stop-and-go extraction tips (StageTips) design^68^. Briefly, a 16-gauge blunt-tip needle (Cadence Science, 7938) was used to punch glass filter membranes from a larger disk (Whatman, grade GF/F, 987468). Using a 20-mL plastic syringe (Henke Sass Wolf, Henke-Ject® Luer Lock, 4200.X00V0), the glass membranes were then plugged into the ends of 200-μL micropipette tips, one for each sample. The plugged tips were then placed in 1.5-mL tubes equipped with centrifuge adapters (GL Sciences, 5010-21514).

A small scoop of PHEA resin was resuspended in 0.5 mL of 200 mM ammonium formate (AF) (Alfa Aesar, 14517) in a 1.5-mL tube, and equal amounts of slurry were transferred to each packed pipette tip. PHEA resin was packed into beds of approximately 1 cm in length by centrifugation at 350-400 rcf. All subsequent centrifugation steps used the same speed. Packed tips were rinsed with 100-uL volumes of the following solvents: 1% FA, 20 mM AF in 90% ACN, 200 mM AF, pure water, 20 mM AF in 90% ACN, and a final wash with 20 mM AF in 90% ACN. Dried peptide samples were resuspended in 200 mM AF to a final concentration of 1 mg/mL. A 10-μL aliquot was transferred to a tube containing 90 μL of neat ACN, producing 10-μg peptide aliquots in 20 mM AF in 90% ACN.

Samples were applied to packed tips, then rinsed with two 100-μL volumes of 20 mM AF in 90% ACN. The packed tips were transferred to clean tubes, and less hydrophilic peptides were eluted with two 100-μL volumes of 40 mM AF in 80% ACN (“Fraction 1”). The packed tips were transferred again to clean tubes, and more hydrophilic peptides were eluted with 150 μL, then 100 μL of 1% FA (‘Fraction 2’). This final elution was performed at 200 rcf. Both eluted fractions were taken to dryness, after which samples were resuspended in 7–10 µL of 0.1% FA in water and transferred to HPLC vials for LC-MS analysis.

### LC-MS data acquisition

Samples were analyzed by online nanoflow liquid chromatography-tandem mass spectrometry using an Orbitrap Eclipse Tribrid mass spectrometer (Thermo Fisher Scientific) coupled to a Dionex UltiMate 3000 HPLC (Thermo Fisher Scientific). A volume of 6 µL for tumor glycopeptide samples or 1–6 µL for PHEA-enriched fractions was injected onto an Acclaim PepMap 100 column packed with 2 cm of 5 µm C18 material (Thermo Fisher, 164564) using 0.1% FA in water (solvent A). Peptides were then separated on a 15 cm PepMap RSLC EASY-Spray C18 column packed with 2 µm of C18 material (Thermo Fisher, ES904) using a gradient from 0-35% solvent B (0.1% FA with 80% ACN) in 60 min.

For glycopeptide and PHEA-enriched Fraction 2 samples (as well as Fraction 1 for Colon 1b and the Salivary Gland), full scan MS1 spectra were collected at a resolution of 60,000, an automatic gain control (AGC) target of 3e5, and a mass range from *m/z* 400-1500 or *m/z* 300-1500. Charge states 2–6 or 2–7 were selected for fragmentation. MS2s were generated at top speed for 3 seconds. Higher-energy collisional dissociation (HCD) was performed on all selected precursor masses with the following parameters: isolation window of 2 *m/z*, stepped collision fragmentation at 25–30–35% or 25-30-40% normalized collision energy (nCE), Orbitrap detection (resolution of 7500), maximum inject time of 75 ms, and a standard AGC target. Dynamic exclusion was enabled with a repeat count of 2, repeat duration of 8 s, and exclusion duration of 8 s.

Detection of at least three HexNAc or Neu5Ac fingerprint ions (*m/z* 126.055, 138.055, 144.07, 168.065, 186.076, 204.086, 274.092, 292.103, and 316.102) present at ±10 ppm and greater than 5% relative intensity triggered additional fragmentation on precursors below *m/z* 850 (with an intensity of at least 5e4) using electron transfer dissociation (ETD), and fragments were detected in the ion trap at a normal scan rate. For precursors between *m/z* 850-1300 (with an intensity over 5e5), detection of fingerprint ions triggered ETD with supplemental activation (EThcD) at 15% nCE, which was analyzed in the Orbitrap with a resolution of 7500. Calibrated charge-dependent reaction times were used for ETD and EThcD fragmentation, with 200 ms maximum injection time and custom injection targets. Dynamic exclusion was enabled with a repeat count of 2, repeat duration of 10 s, and exclusion duration of 10 s.

For PHEA-enriched Fraction 1 samples of Colon 1a and the Esophagus, full scan MS1 spectra were collected at a resolution of 60,000, an automatic gain control (AGC) target of 3e5, and a mass range from *m/z* 400-1500 or 300-1500. Charge states 2–6 were selected for fragmentation. MS2s were generated at top speed for 3 seconds. Higher-energy collisional dissociation (HCD) was performed on all selected precursor masses with the following parameters: isolation window of 2 *m/z*, stepped collision fragmentation at 27–29–31% or 25–30–35% normalized collision energy (nCE), Orbitrap detection (resolution of 15,000), maximum inject time of 100 ms, and a standard AGC target. Dynamic exclusion was enabled with a repeat count of 2, repeat duration of 7 s, and exclusion duration of 8 s. No electron-based MS2 spectra were collected for these samples.

### Unmodified proteome searches of PHEA-enriched tryptic peptides

Tryptic peptide *.raw files were searched against the human proteome with Byonic (Protein Metrics)^69^. Mass tolerances of 10 ppm, 20 ppm, and 0.3 Da were used for MS1, FTMS MS2, and ITMS MS2, respectively. Met oxidation was selected as a variable modification. The search was set to “fully specific” with proteolytic cleavage C-terminal to R and K, allowing 2 missed cleavages. The protein-level FDR was set to 1%.

### Glycoproteomic searches with O-Pair

Glycopeptide *.raw files for each tumor region were searched using O-Pair with MetaMorpheus (v1.0.2 or v1.0.3)^70^, using a manually curated protein database containing mucins of interest and StcE. The dissociation type was set to HCD, and EThcD was selected for child scan dissociation. Precursor and product ion mass tolerances were set to 10 ppm and 20 ppm, respectively. “Non-specific” protease cleavage was selected, and peptide length was set to 5–25 amino acids. A maximum of 6 O-glycans was allowed, using the “OGlycan.gdb” database. All glycopeptide identifications assigned Level 1 or 1b were validated by manual inspection of raw spectra using QualBrowser in XCalibur (v4.1.31.9) and added to the final reporting template (Supplementary Data 2).

### Glycoproteomic searches with Byonic

Glycopeptide *.raw files were also searched with Byonic (v4.5.2) to improve glycoproteomic coverage of mucins previously identified only by unmodified peptides. Separate searches were performed using focused databases created from searches of the two unmodified peptide fractions for each sample. “Fully specific” protease cleavage N-terminal to Ser and Thr residues was selected, allowing for a maximum of 6 missed cleavages. Precursor and product ion mass tolerances were set to 10 ppm and 20 ppm/0.35 Da (FTMS/ITMS), respectively. A custom O-glycan database that included the 6 most common O-glycans, glycan compositions manually identified in the Healthy Colon data, and mono-/di-/tri-acetylated versions of each sialylated O-glycans was used in each search. Glycopeptide identifications not found in original O-Pair searches were validated by manual inspection of raw spectra using QualBrowser in XCalibur (v4.1.31.9) and added to the final reporting template (Supplementary Data 2).

### Assignment of identified glycopeptides to MALDI-MSI spectra

Subsequently, putative LC-MS *m/z* values corresponding to doubly and triply protonated precursor ions were calculated for each *m/z* detected by MALDI-MSI. Raw LC-MS data were then searched manually for the possible precursor masses. If one of the calculated *m/z* values was detected, the O-Pair output file was checked for any identifications with the *m/z* detected. If there were no O-Pair identifications for a detected *m/z*, the corresponding HCD and ETD/EThcD MS2 spectra were used for manual peptide sequencing and glycan localization. Sequenced peptides were then matched to specific proteins using NCBI’s BLAST algorithm^71^.

### H&E staining, MUC1 immunohistochemistry, and StcE^E447D^ staining

Tissues were sectioned (4 μm in thickness) from tissue blocks. Slides were baked at 70 °C for 1 h followed by deparaffinization and rehydration with washes in xylene (3 times), 100% ethanol (twice), 95% ethanol (twice), 80% ethanol (once), 70% ethanol (once) and ddH_2_O with a Leica ST4020 Linear Stainer (Leica Biosystems). Tissues next underwent antigen retrieval, which was carried out by submerging sides in 3-in-1 Target Retrieval solution (pH 9, Dako Agilent) and incubating them at 97 °C for 40 min in a Lab Vision PT Module (Thermo Fisher Scientific). After cooling to room temperature, slides were washed in 1× PBS IHC washer buffer with Tween 20 (Cell Marque) with 0.1% (w/v) BSA (Thermo Fisher). Next, all tissue samples underwent blocking with BLOXALL Endogenous Blocking Solution, Peroxidase and Alkaline Phosphatase (Vector Laboratories); Avidin/Biotin Blocking Kit (Vector Laboratories) was used for biotinylated reagents. Tissue samples were then washed with wash buffer and blocked for 1 h at room temperature with 1× TBS IHC wash buffer with Tween 20 and 3% (v/v) normal donkey serum (Sigma-Aldrich), 0.1% (v/v) cold fish skin gelatin (Sigma-Aldrich), 0.1% (v/v) Triton X-100 and 0.05% (v/v) sodium azide. The tissues were then stained overnight with Anti-MUC1 (Abcam ab218998) at 0.25 µg/mL or biotinylated StcE^E447D^ at 1 µg/mL in 1× TBS IHC wash buffer with Tween 20 and 3% (v/v) normal donkey serum (Sigma-Aldrich) overnight at 4 °C in a Sequenza staining rack (Thermo Fisher Scientific). After overnight incubation, slides were washed with 2 mL of wash buffer. For slides with biotinylated StcE^E447D^, HRP Streptavidin (Vector Laboratories, SA-5704-100) was used and for anti-MUC1 stain, HRP Goat Anti-Rabbit (Vector Laboratories, SKU MP-7451-15) was used according to manufacturer instructions. These were incubated with the tissues for 1 h at 4 °C. After staining, slides were washed twice for 5 min in wash buffer and revealed with DAB (Vector Laboratories) for 40 s and counterstained with Hematoxylin. Slides were imaged with an Aperio AT2 digital pathology scanner (Leica Biosystems).

### Reporting summary

Further information on research design is available in the Nature Portfolio Reporting Summary linked to this article.

## Supplementary information

Supplementary Information

Description of Additional Supplementary Files

Supplementary Data 1

Supplementary Data 2

Reporting Summary

Transparent Peer Review file

## Source data

Source Data 1

Source Data 2

## Data Availability

The LC-MS proteomics raw data and search outputs have been deposited to the ProteomeXchange Consortium via the PRIDE^72^ partner repository with the dataset identifier PXD055865. Unless otherwise stated, all data supporting the results of this study can be found in the article, supplementary, and source data files. Source data are provided with this paper.
