# Supplementary material for: Glycosite mapping and in situ mass spectrometry imaging of MUC2 glycopeptides via on-slide mucinase digestion
Source: Nat Commun. 2026 May 7;17:6125. doi: 10.1038/s41467-026-72853-3 (PMC13357551; doi:10.1038/s41467-026-72853-3)
Supplement: Supplementary file 1 — Supplementary Information [file 41467_2026_72853_MOESM1_ESM.pdf]

Supplementary Figures, Tables, & Text

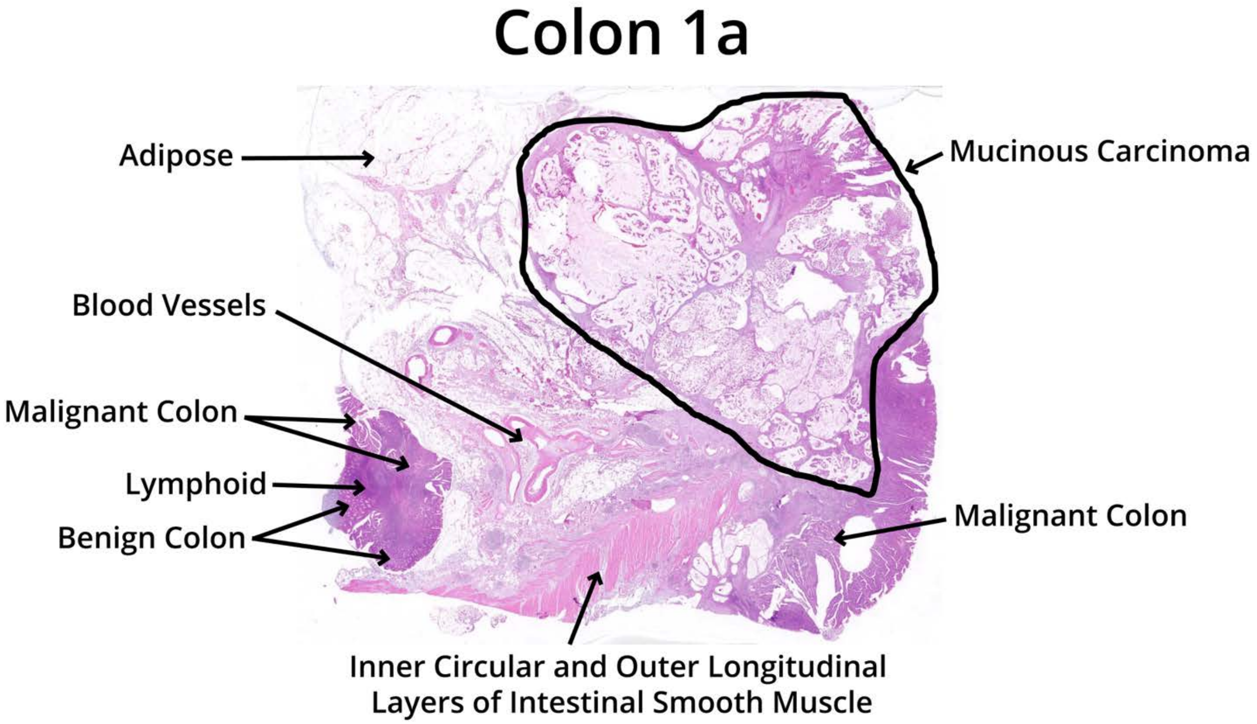

Supplementary Fig. 1. H&E stain of Colon 1a, with tissue features annotated and tumor region encircled. Tumor infiltration into muscle tissue is observed, therefore this tumor is classified as Stage II.[1]

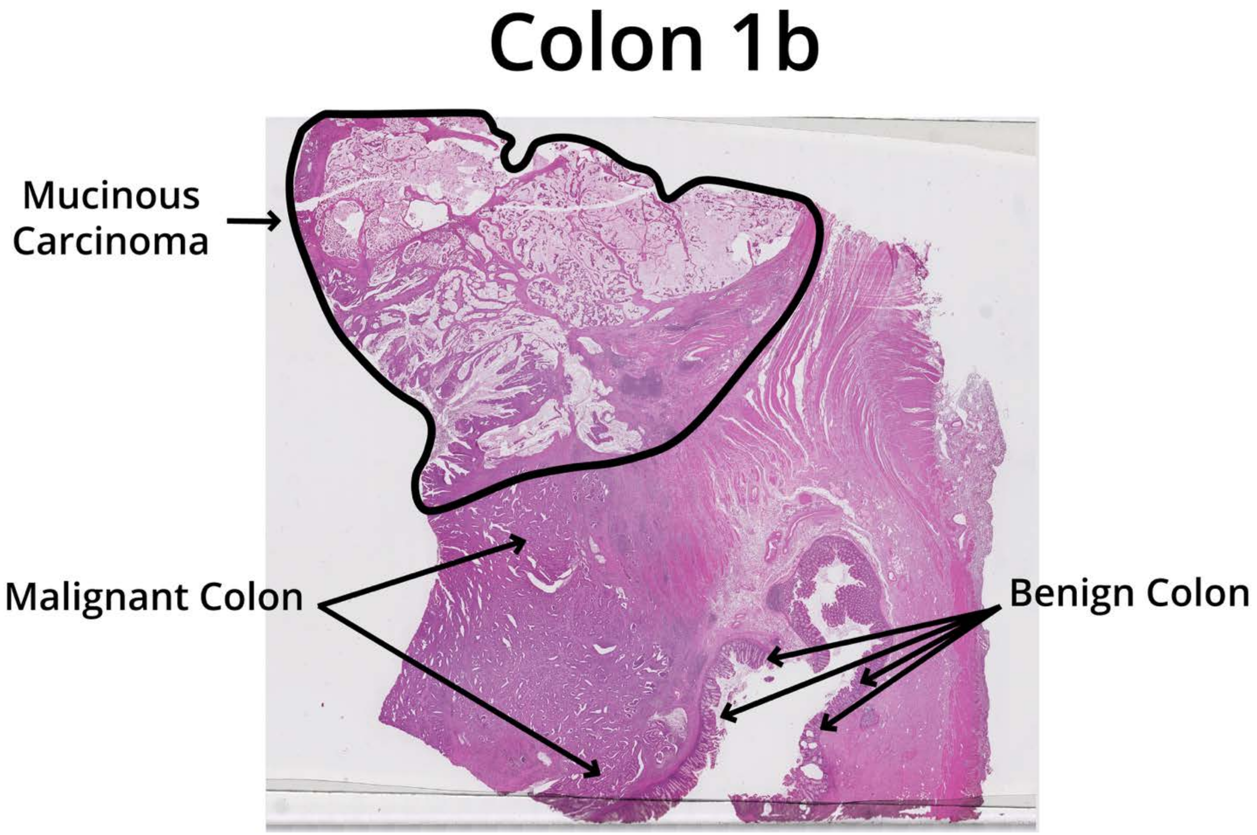

Supplementary Fig. 2. H&E stain of Colon 1b, with tissue features annotated and tumor region encircled. This tumor was resected from the same patient as Colon 1a. Tumor infiltration into muscle tissue is observed, therefore this tumor is classified as Stage II.[1]

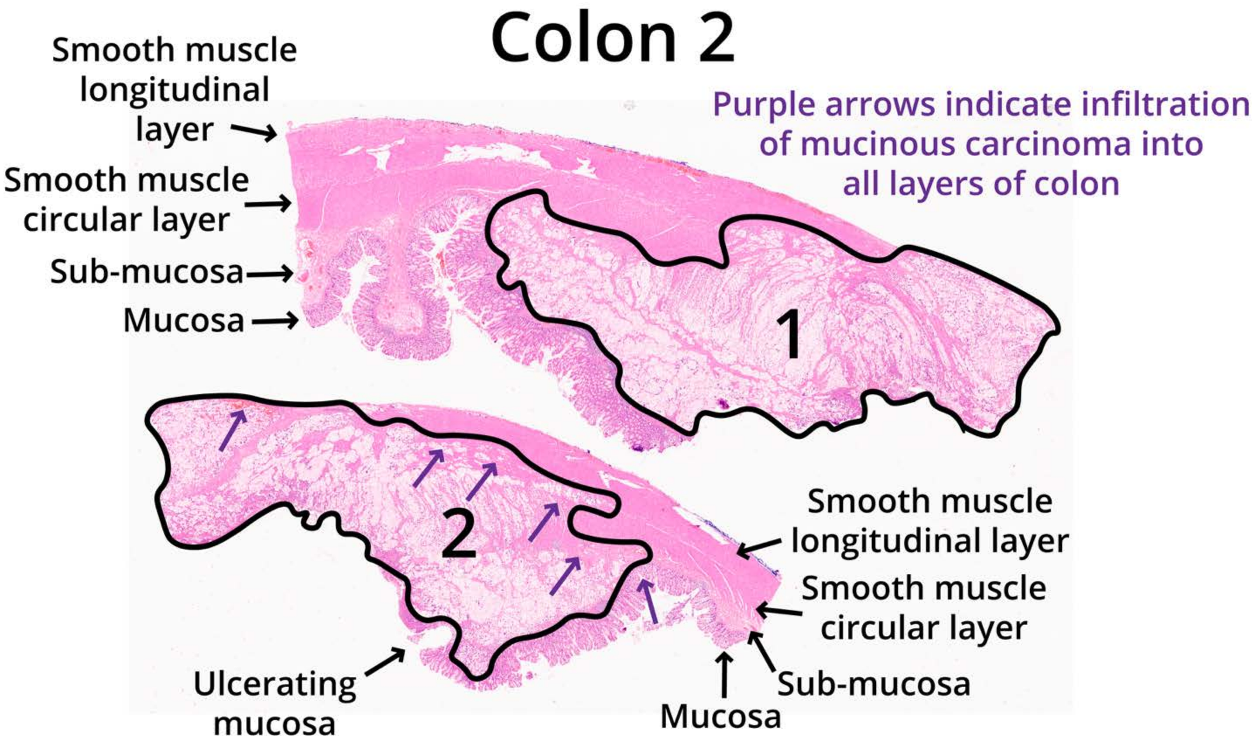

Supplementary Fig. 3. H&E stain of Colon 2, with tissue features annotated and tumor regions encircled.

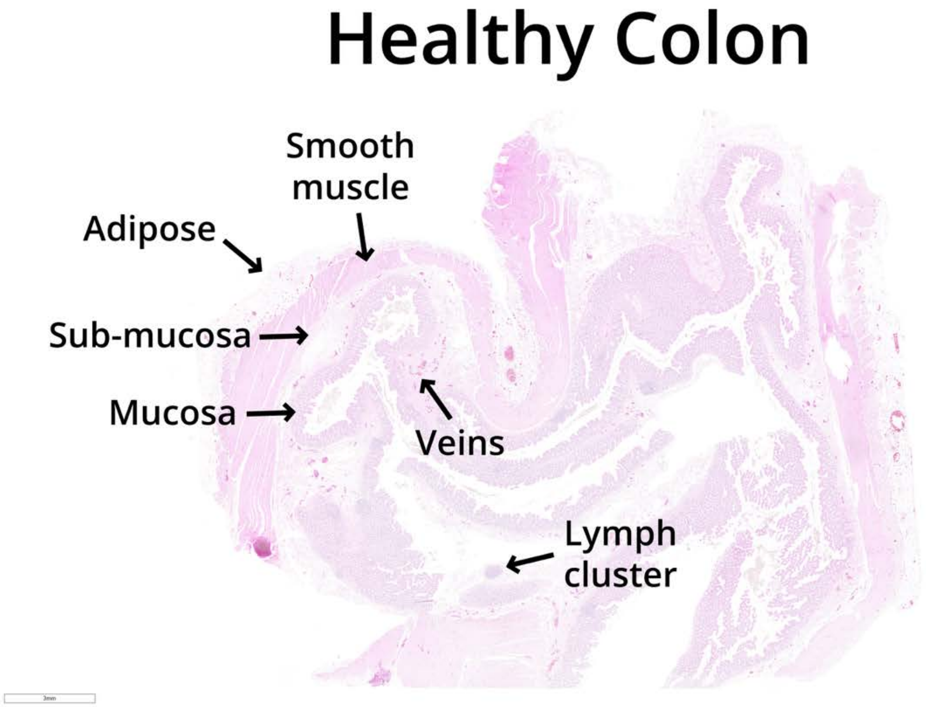

Supplementary Fig. 4. H&E stain of the Healthy Colon with tissue features annotated.

Esophagus

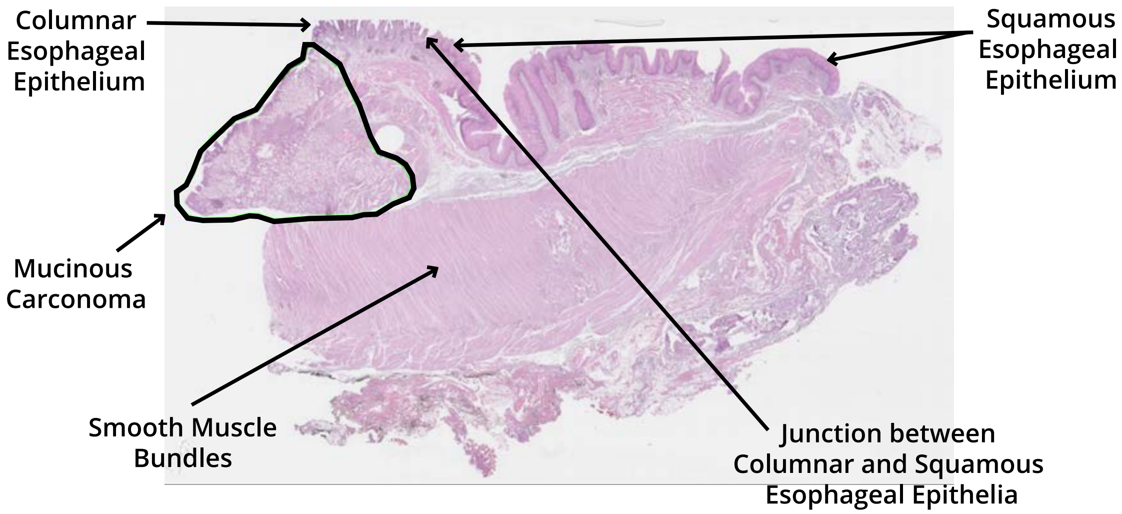

Supplementary Fig. 5. H&E stain of the esophageal sample, with tissue features annotated and tumor region encircled. The mucinous tumor is adjacent to the junction of the columnar and squamous epithelia, a hotspot for the development of carcinomas.[2] This sample may be a metastasis rather than a primary tumor, as the malignant tissue appears to infiltrate from below and does not connect to the surface epithelium.

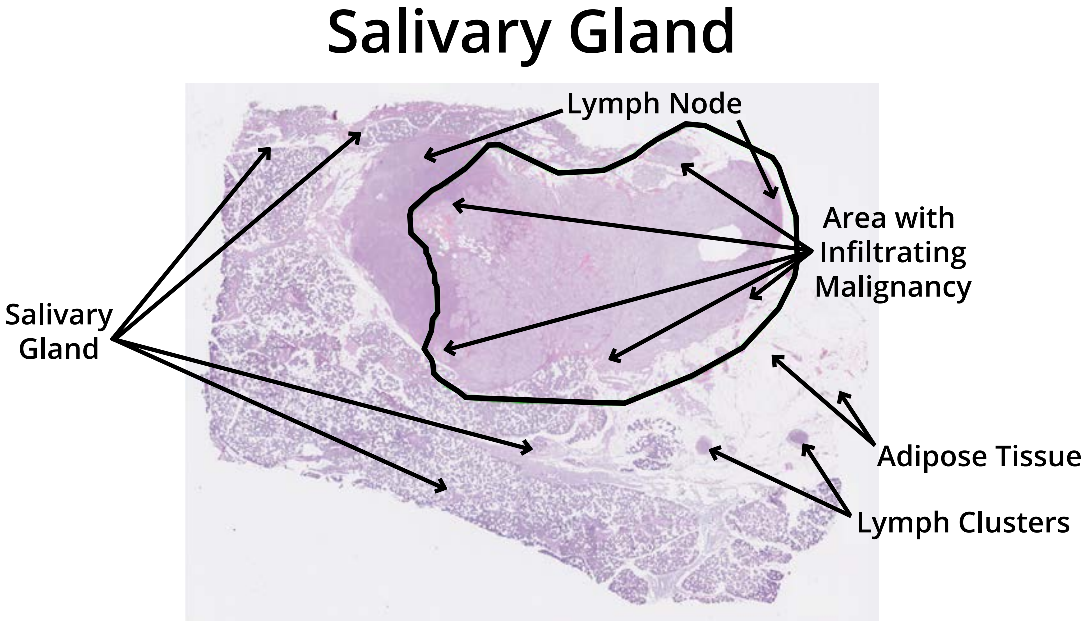

Supplementary Fig. 6. H&E stain of the salivary gland sample, with tissue features annotated and tumor region encircled. This carcinoma might not represent a primary tumor, as salivary gland tumors are rarely mucinous.[3,4] The malignant region in this sample resides in the lymph node adjacent to parotid tissue, also supporting the characterization of this sample as a metastasis.

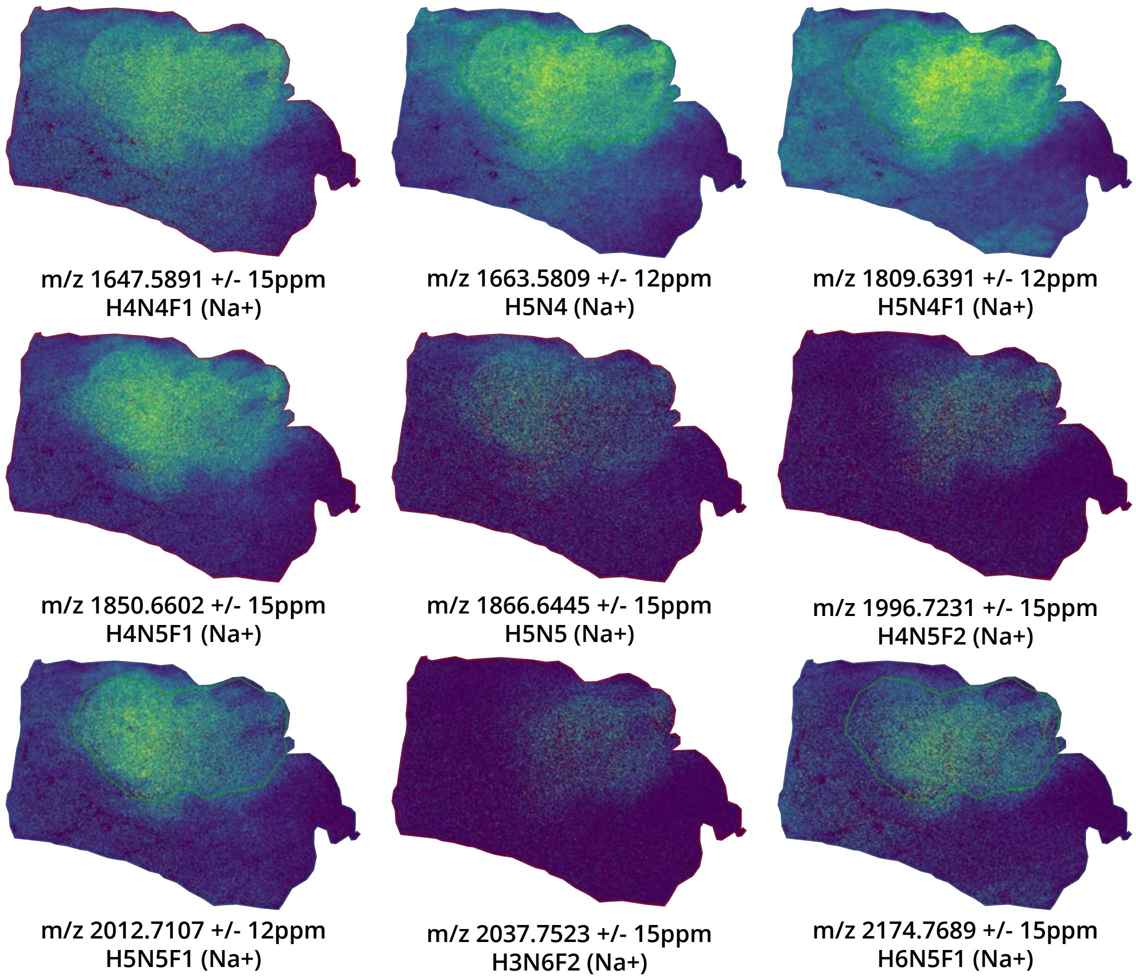

Supplementary Fig. 7. MALDI-MSI images of N-glycans detected within the boundaries of the salivary gland tumor treated with PNGaseF and mucinase StcE. α-cyano-4-hydroxycinnaminic acid (CHCA) matrix was applied via automatic sprayer prior to acquisition with a timsTOF fleX MALDI-QTOF mass spectrometer (Bruker). Images were generated with SCiLS Lab 2024b Pro (Bruker) by manually selecting *m/z* values within the mass window indicated. All identified species correspond to complex-type N-glycans, and all depicted masses correspond to mono-sodiated precursor ions. Individual images with scale bars and color scales have been uploaded in the Source Data.

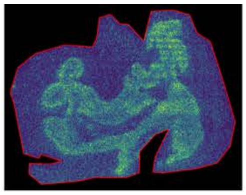
m/z 951.4011 +/- 39.2 ppm
TTV + N3 (Na+)

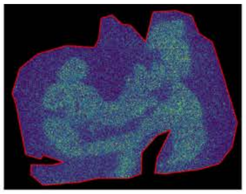
m/z 1051.3808 +/- 39.2 ppm
TQ + N2Ac$_2$A1 (Na+)

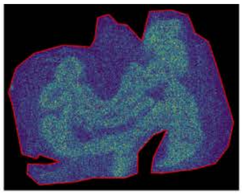
m/z 1093.3919 +/- 39.2 ppm
TQ + N2Ac$_3$A1 (Na+)

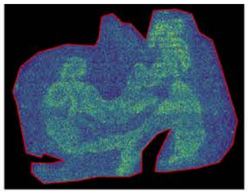
m/z 1142.5435 +/- 39.2 ppm
TAPIAEL + N2 (Na+)

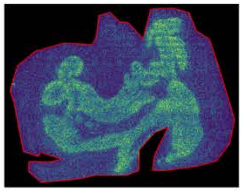
m/z 1149.5015 +/- 39.2 ppm
TPIST or TPTSI + N3 (Na+)

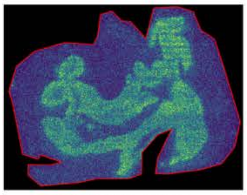
m/z 1154.4803 +/- 39.2ppm
TTV + N4 (Na+)

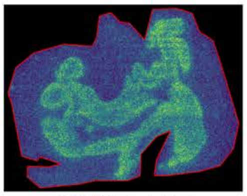
m/z 1163.5172 +/- 39.2ppm
TPITT + N3 (Na+)

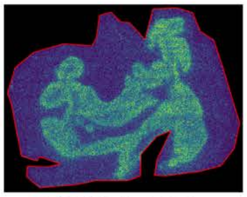
m/z 1280.5232 +/- 39.2ppm
TQTP + N4 (Na+)

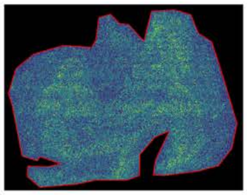
m/z 1286.6223 +/- 39.2ppm
TELDVLLP + H1N1 (Na+)

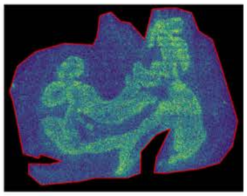
m/z 1304.5963 +/- 39.2ppm
TAPIAEL + H1N2 (Na+)

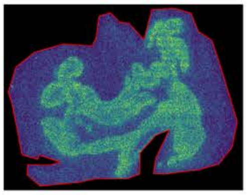
m/z 1316.5331 +/- 39.2ppm
TTV + H1N4 (Na+)

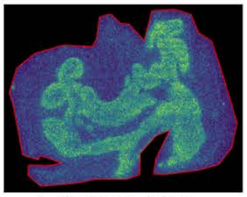
m/z 1325.5700 +/- 39.2ppm
TPITT + H1N3 (Na+)

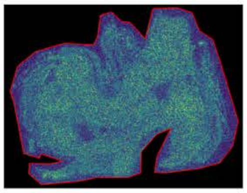
m/z 1359.4915 +/- 39.2ppm
TQ + H1N2Ac$_2$A1F1 (Na+)

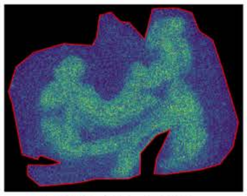
m/z 1389.5019 +/- 39.2ppm
TT + H1N3Ac$_2$A1 (Na+)

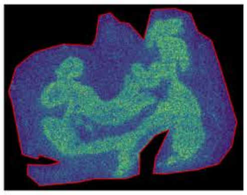
m/z 1399.5549 +/- 39.2ppm
SAFPWEEF + H1N1 (Na+)

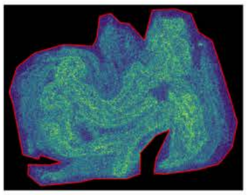
m/z 1401.5020 +/- 39.2ppm
TQ + H1N2Ac$_3$A1F1 (Na+)

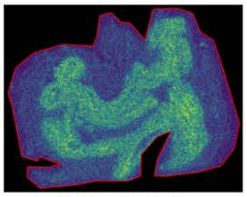
m/z 1401.5383 +/- 39.2ppm
TK + H1N2Ac$_3$A1F1 (Na+)

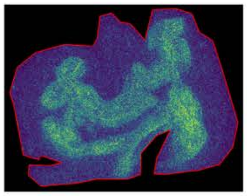
m/z 1416.5128 +/- 39.2ppm
TQ + H1N3Ac$_2$A1 (Na+)

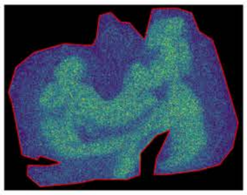
m/z 1431.5124 +/- 39.2ppm
TT + H1N3Ac$_3$A1 (Na+)

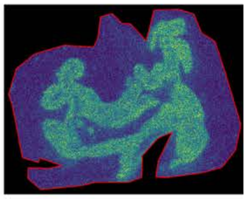
m/z 1442.5760 +/- 39.2ppm
TQTP + H1N4 (Na+)

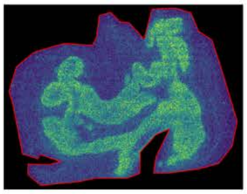
m/z 1453.6284 +/- 39.2ppm
TPISTT + N4 (Na+)

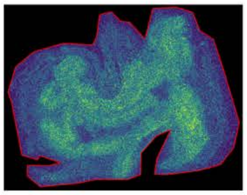
m/z 1458.5233 +/- 39.2ppm
TQ + H1N3Ac$_3$A1 (Na+)

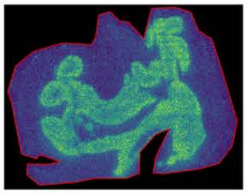
m/z 1482.6186 +/- 39.2ppm
TQTPTT + N4 (Na+)

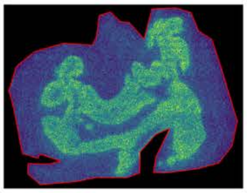
m/z 1528.6492 +/- 39.2 ppm
TPITT + H1N4 (Na+)

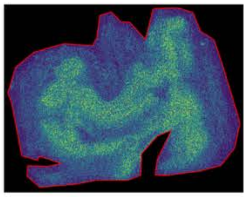
m/z 1532.5600 +/- 39.2ppm
TTT + H1N3Ac$_3$A1 (Na+)

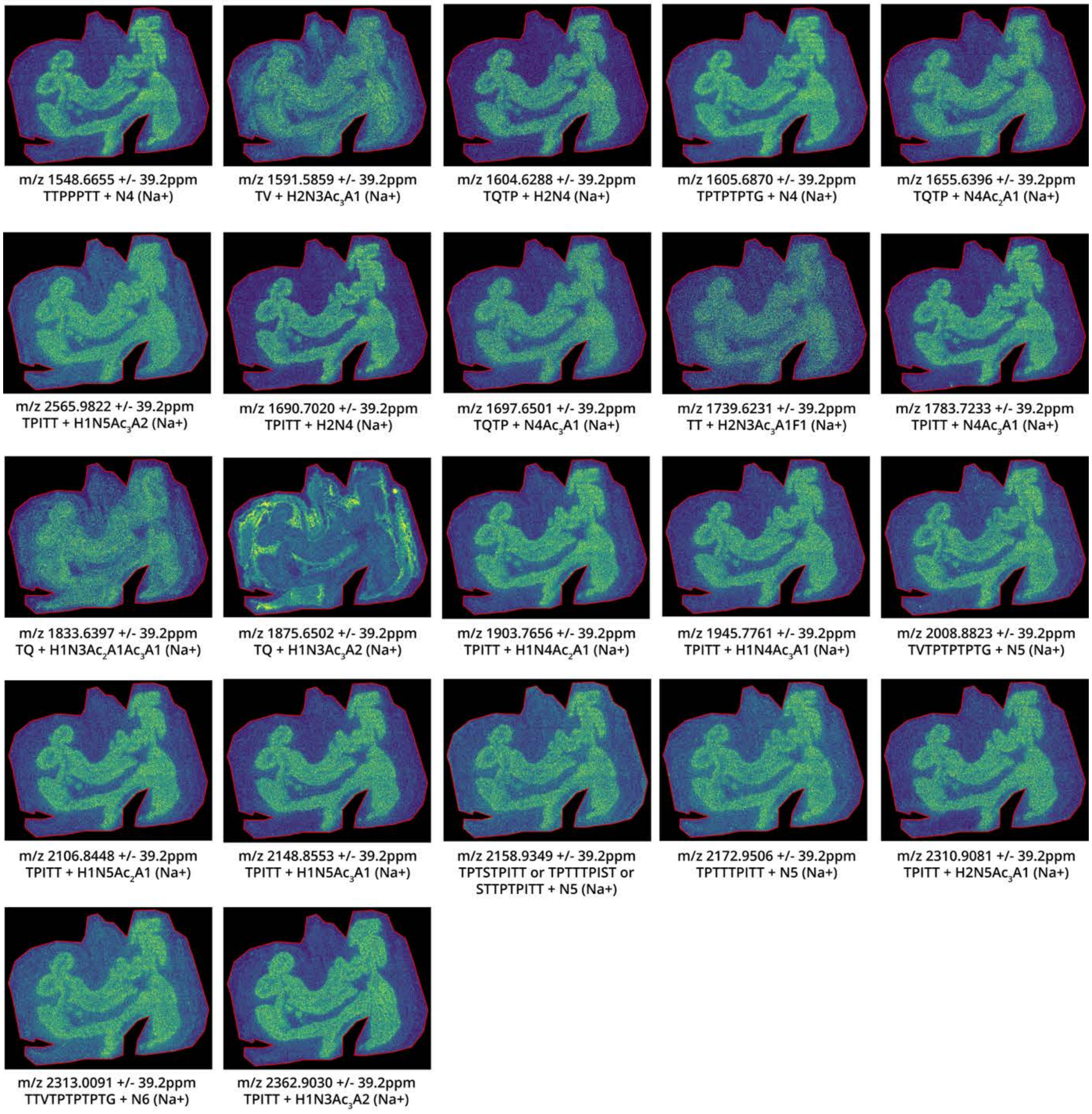

Supplementary Fig. 8. Glycopeptides detected in MALDI-MSI and identified via LC-MS analysis of the Healthy Colon tissue display diffuse, indistinct localizations. Images were acquired with a timsTOF fleX MALDI-QTOF (Bruker), and each *m/z* value was manually extracted using SCiLS Lab version 2024b Pro (Bruker). All *m/z* values shown correspond to the mono-sodiated precursor mass of the associated glycoform. Species were identified using LC-MS data collected for the Healthy Colon treated with StcE. Individual images with scale bars and color scales have been uploaded in the Source Data.

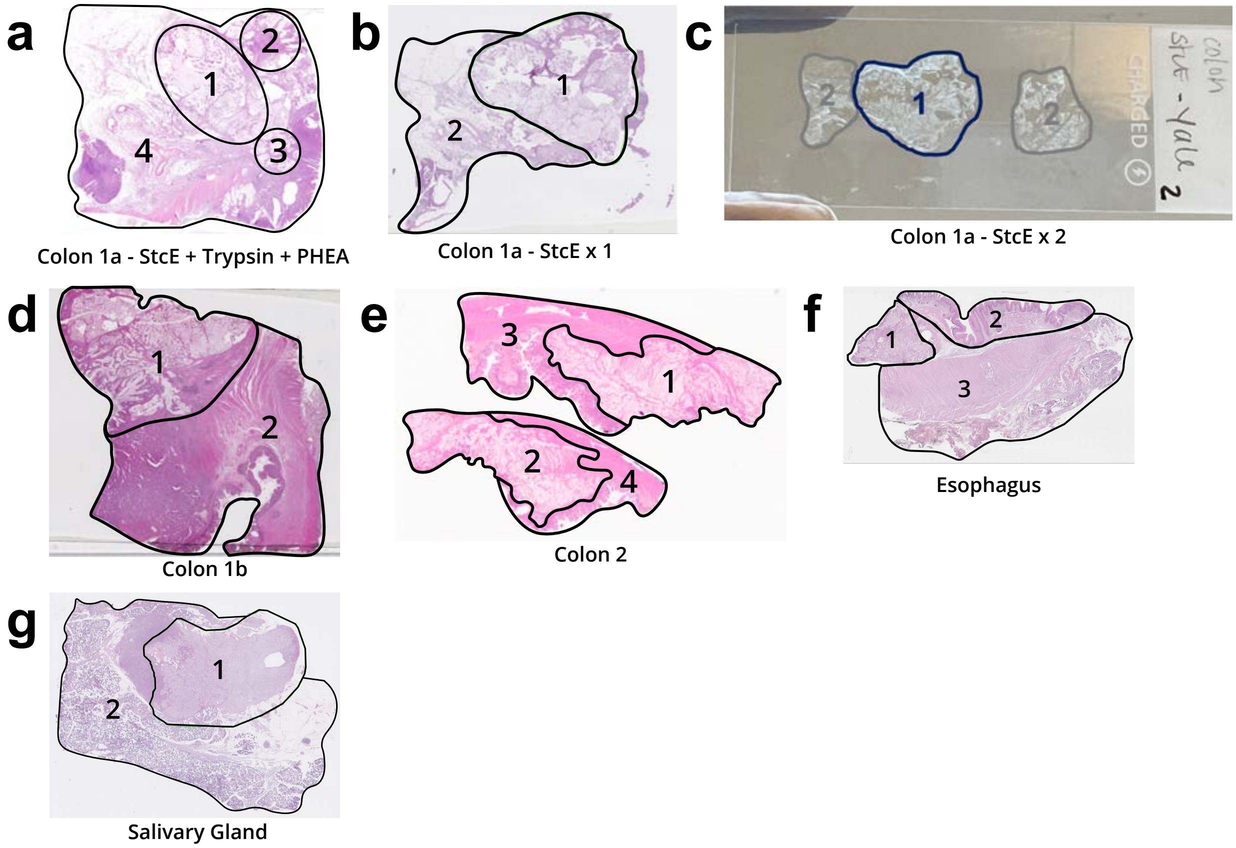

Supplementary Fig. 9. Tumor and tumor-adjacent regions were outlined with a hydrophobic barrier pen for LC-MS experiments. (A) Based on intratumor expression patterns observed in MALDI-MSI, initial experiments with Colon 1a divided the tumor into Regions 1, 2, and 3, while the rest of the tissue was designated Region 4. (B) A different tissue section of Colon 1a used for a StcE-only experiment. Regions 1 and 2 correspond to the tumor and tumor-adjacent regions, respectively. (C) A third section of Colon 1a used for double-StcE experiments. Regions 1 and 2 correspond to the tumor and tumor-adjacent regions, respectively. The separate tumor-adjacent regions were combined into a single sample for this particular tissue. (D) Colon 1b was separated into Regions 1 (tumor) and 2 (tumor-adjacent). (E) The two tumor areas of Colon 2 were designated Regions 1 and 2, while the tumor-adjacent tissue was separated into Regions 3 & 4. (F) In the esophageal sample, the tumor was designated Region 1. Because we anticipated high mucin content in the villous tumor-adjacent area, we separated Region 2 from the rest of the tumor-adjacent Region 3. (G) The salivary gland sample was separated into Regions 1 (tumor) and 2 (tumor-adjacent). Note: the entirety of the healthy colon tissue was treated as a single sample.

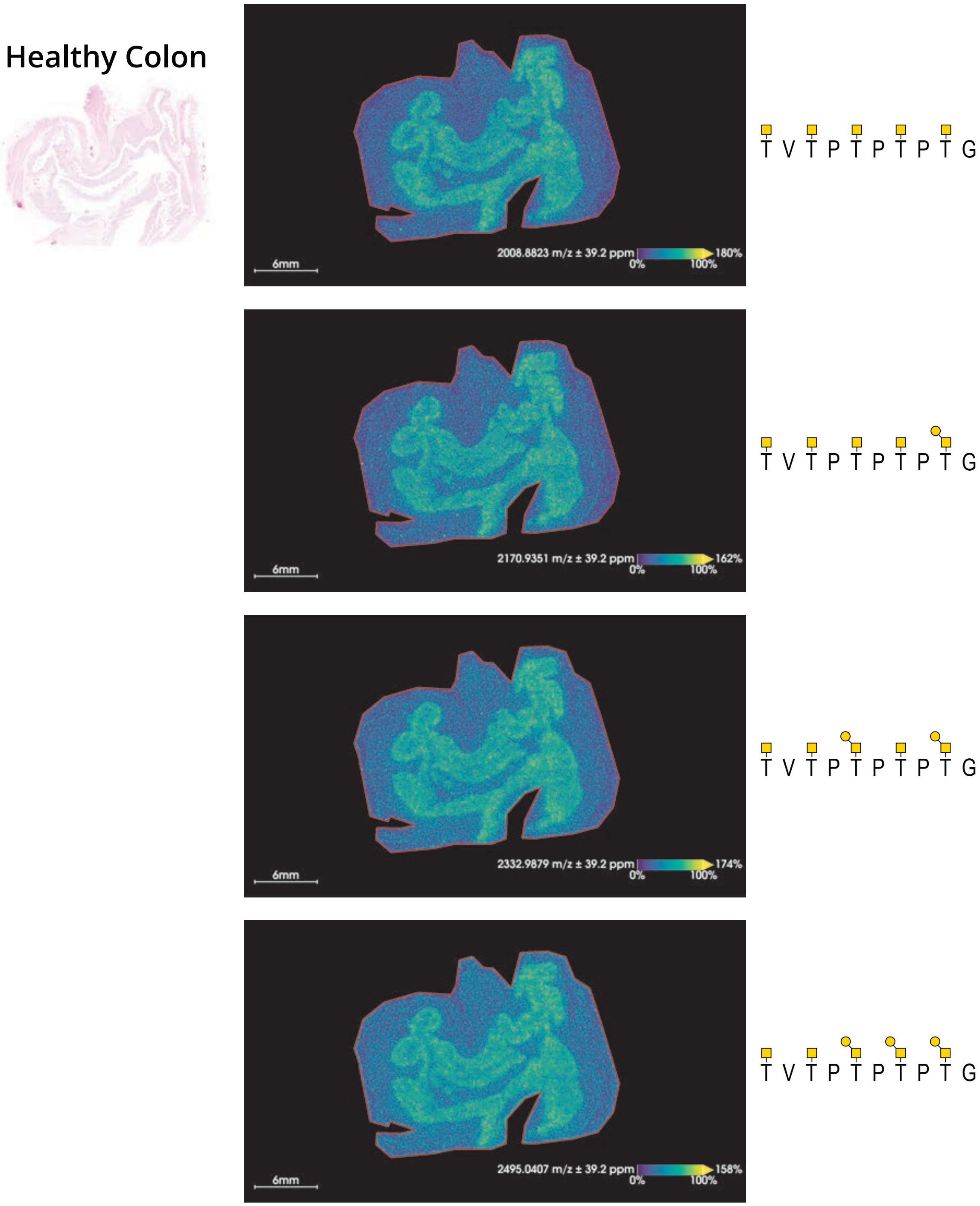

Supplementary Fig. 10. MUC2 glycopeptides associated with the tumor regions of Colons 1a/b & 2 do not demonstrate glycoform-specific expression in the Healthy Colon. Images were acquired with a timsTOF fleX MALDI-2 mass spectrometer (Bruker), and each *m/z* value was manually extracted using SCiLS Lab version 2024b Pro (Bruker). All *m/z* values shown correspond to the mono-sodiated precursor mass of the associated glycoform. Species were identified using LC-MS data collected for the Healthy Colon. Individual images with scale bars and color scales have been uploaded in the Source Data.

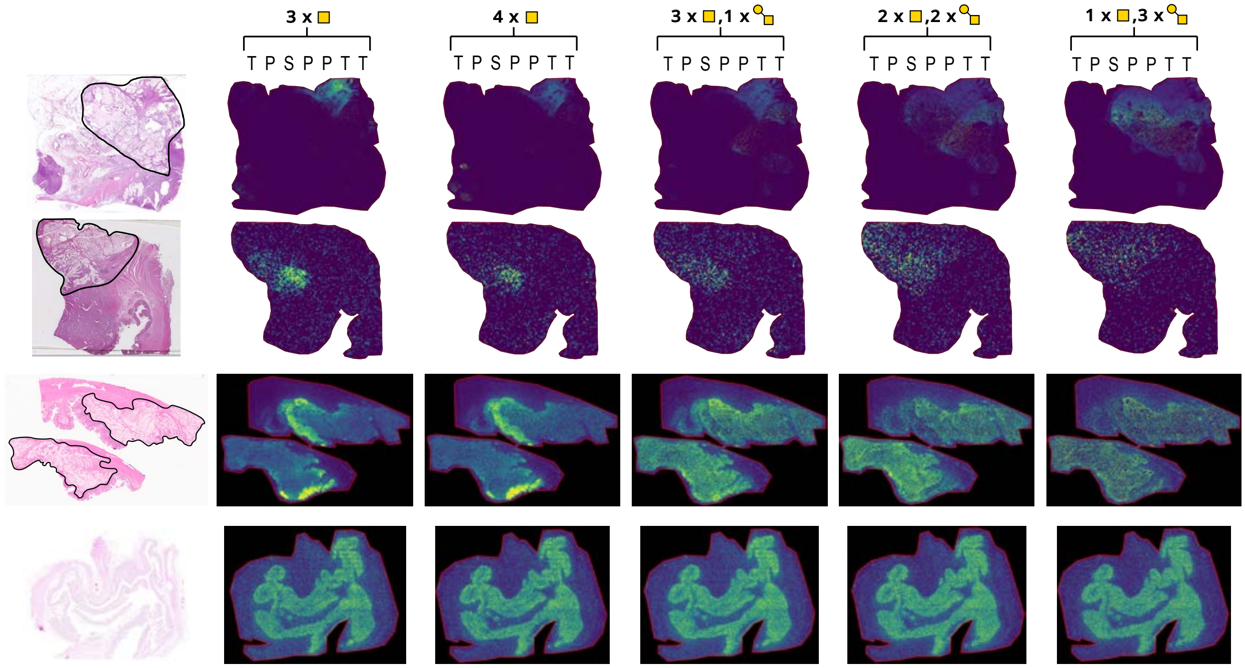

Supplementary Fig. 11. TPSPPTT glycoforms with three or four glycosites exhibit similar spatial distributions to glycoforms with only three glycosites shown in Figures 2b-c. MALDI-MSI data for Colon 1a (top row) was acquired with a timsTOF fleX MALDI-QTOF mass spectrometer (Bruker). Images for Colon 1b (second row) were acquired with a Solarix dual-source 7T MALDI-FTICR mass spectrometer (Bruker). Colon 2 & Healthy Colon (third and fourth rows, respectively) were analyzed with a timsTOF fleX MALDI-2 mass spectrometer (Bruker). Each *m/z* value was manually extracted using SCiLS Lab version 2024b Pro (Bruker) and manually inspected for tumor localization prior to image export. All *m/z* values shown correspond to the mono-sodiated precursor mass of the associated glycoform. Species were identified using LC-MS data collected for each tissue. The H3N4 glycoform was not identified via LC-MS in Healthy Colon but is inferred based on precursor mass in MALDI-MSI and the presence of multiple other TPSPPTT glycoforms in the LC-MS data for this samples. Note that in this figure, Colon 2 and the Healthy Colon are not normalized to the same level to facilitate visualization of glycoform distribution in the latter. Individual images with scale bars and color scales have been uploaded in the Source Data.

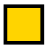 **Only**

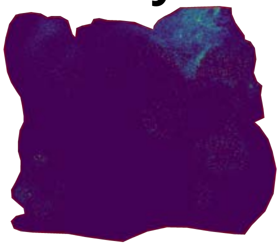

m/z 1163.5183 +/- 15ppm
TPITT + N3 (Na+)

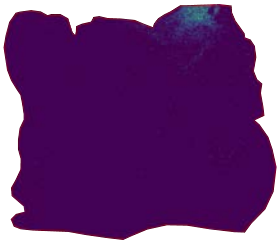

m/z 1279.5424 +/- 15ppm
TQTPTT + N3 (Na+)

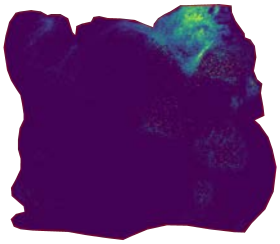

m/z 1482.6216 +/- 15ppm
TQTPTT + N4 (Na+)

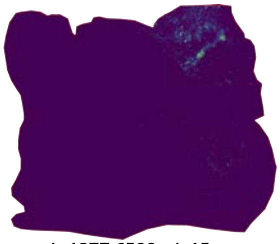

m/z 1377.6528 +/- 15ppm
TLTPITT + N3 (Na+)

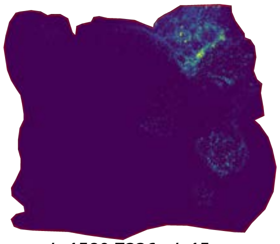

m/z 1580.7336 +/- 15ppm
TLTPITT + N4 (Na+)

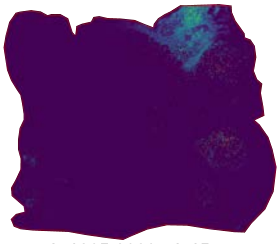

m/z 1805.8029 +/- 15ppm
TVTPTPTPTG + N4 (Na+)

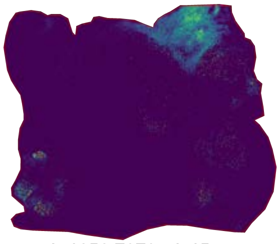

m/z 1852.7979 +/- 15ppm
TTTPPPTT + N5 (Na+)

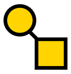 **x 1**

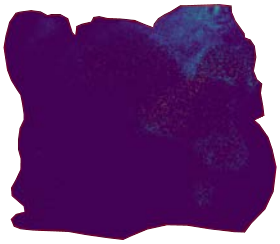

m/z 1325.5690 +/- 15ppm
TPITT + H1N3 (Na+)

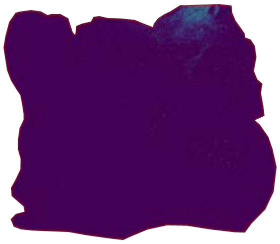

m/z 1441.5960 +/- 15ppm
TQTPTT + H1N3 (Na+)

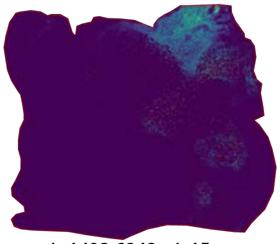

m/z 1493.6248 +/- 15ppm
TPSPPTT + H1N3 (Na+)

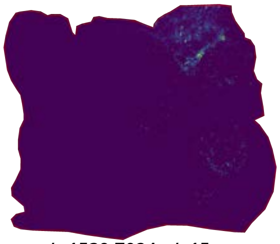

m/z 1539.7024 +/- 15ppm
TLTPITT + H1N3 (Na+)

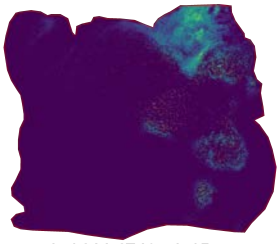

m/z 1644.6742 +/- 15ppm
TQTPTT + H1N4 (Na+)

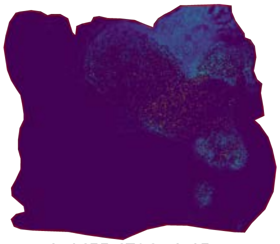

m/z 1655.6796 +/- 15ppm
TPSPPTT + H2N3 (Na+)

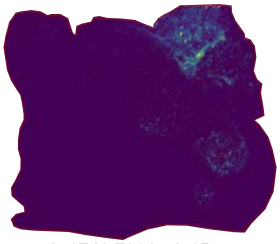

m/z 1742.7821 +/- 15ppm
TLTPITT + H1N4 (Na+)

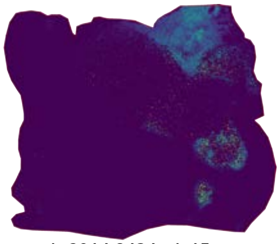

m/z 2014.8434 +/- 15ppm
TTTPPPPTT + H1N5 (Na+)

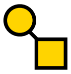 **x 2**

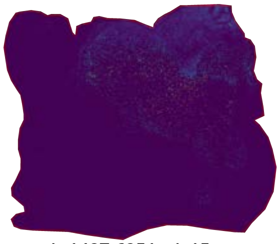

m/z 1487.6251 +/- 15ppm
TPITT + H2N3 (Na+)

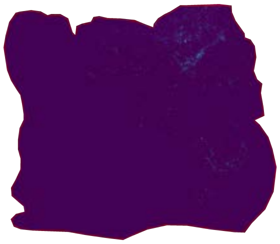

m/z 1701.7614 +/- 15ppm
TLTPITT + H2N3 (Na+)

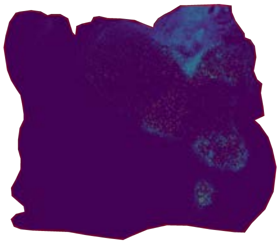

m/z 1806.7267 +/- 15ppm
TQTPTT + H2N4 (Na+)

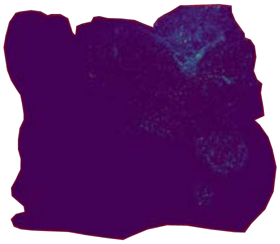

m/z 1904.8377 +/- 15ppm
TLTPITT + H2N4 (Na+)

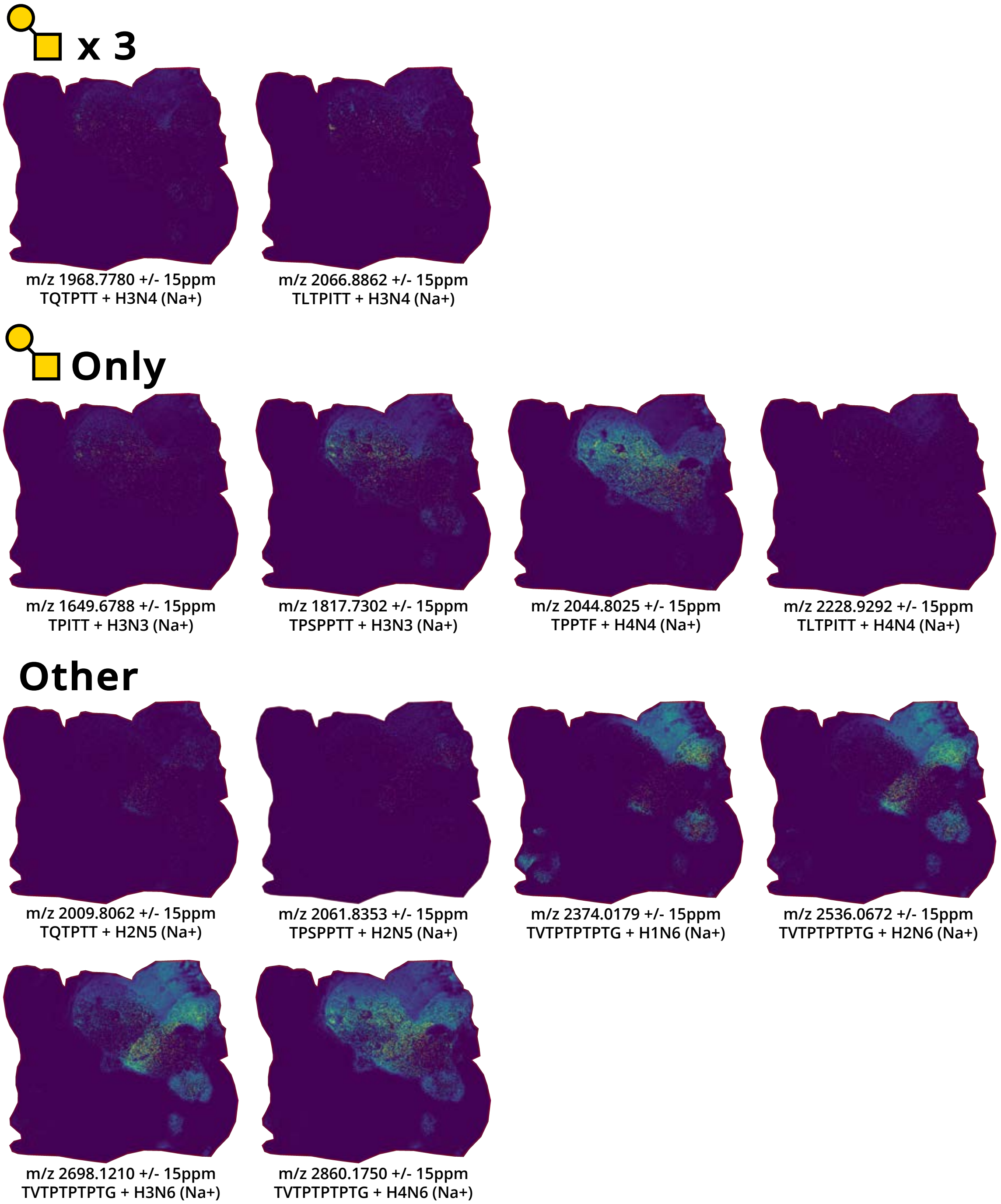

Supplementary Fig. 12. Additional glycopeptides detected by MALDI-MSI in the tumor region of PNGaseF- and StcE-treated Colon 1a that were subsequently identified with LC-MS data. Images were acquired with a timsTOF fleX MALDI-QTOF (Bruker). Each *m/z* value was manually extracted using SCiLS Lab version 2024b Pro (Bruker) and manually inspected for tumor localization prior to image export. All *m/z* values shown correspond to the mono-sodiated precursor mass of the associated glycoform. Species were identified using LC-MS data collected for the tumor region of Colon 1a prepared with the double-StcE workflow. Individual images with scale bars and color scales have been uploaded in the Source Data.

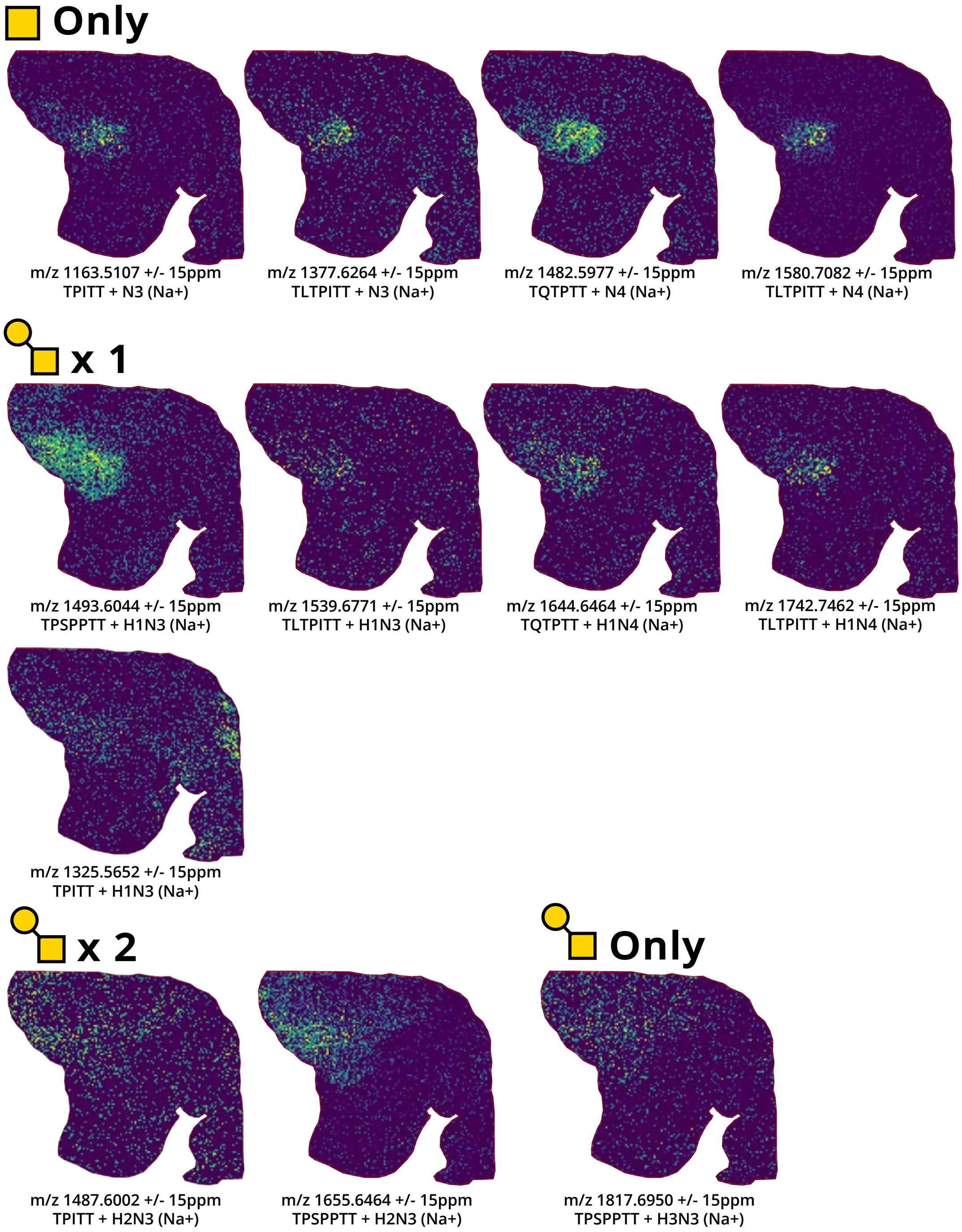

Supplementary Fig. 13. Additional glycopeptides detected by MALDI-MSI in the tumor region of PNGaseF- and StcE-treated Colon 1b that were subsequently identified by LC-MS. Images were acquired with a Solarix dual-source 7T MALDI-FTICR mass spectrometer (Bruker). Each *m/z* value was manually extracted using SCiLS Lab version 2024b Pro (Bruker) and manually inspected for tumor localization prior to image export. The *m/z* values shown correspond to mono-sodiated precursor masses for the associated glycoform. Species were identified using LC-MS data collected for the tumor region of Colon 1b prepared with the double-StcE workflow. Individual images with scale bars and color scales have been uploaded in the Source Data.

Only

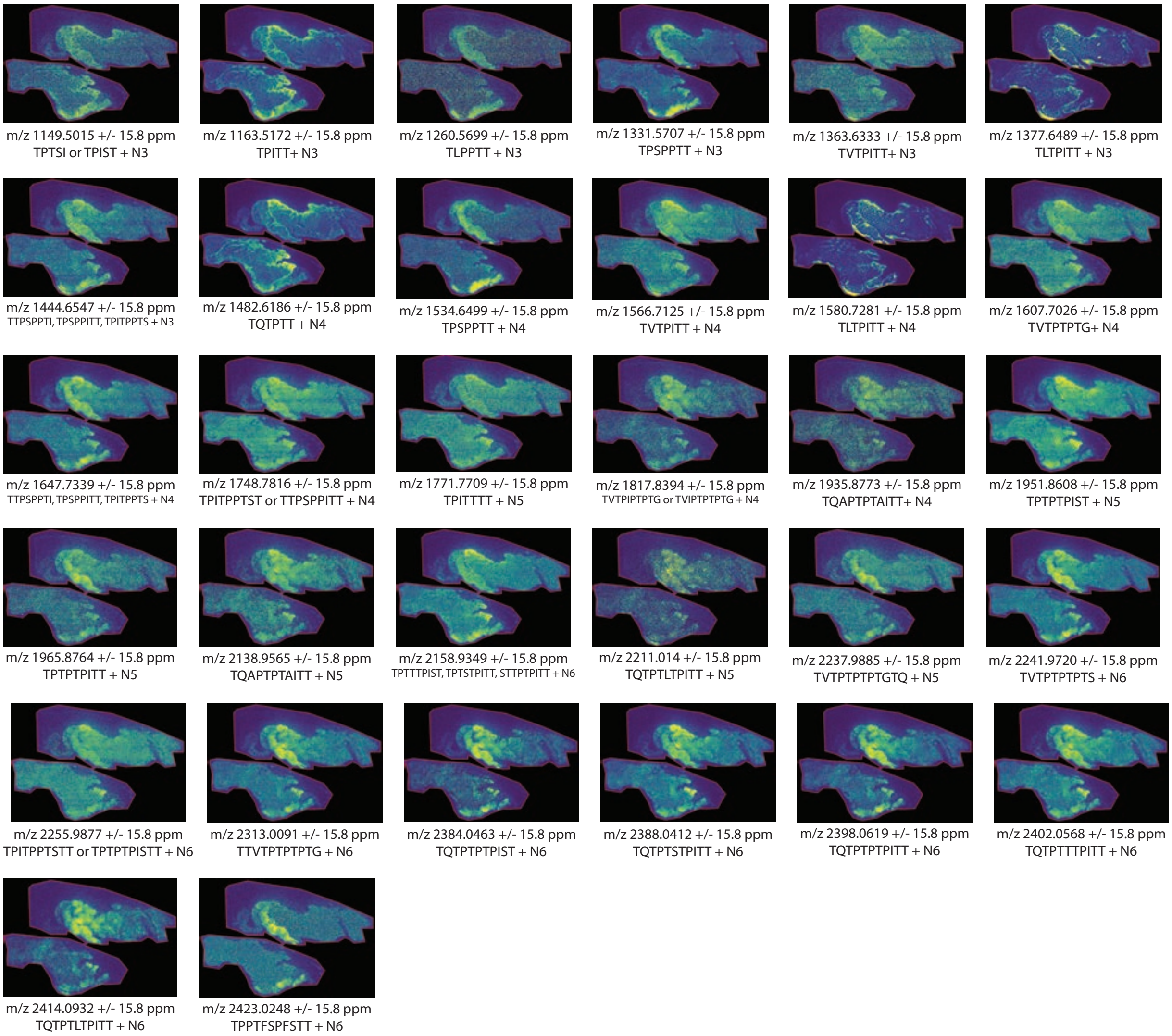
m/z 1149.5015 +/- 15.8 ppm
TPTSI or TPIST + N3
m/z 1163.5172 +/- 15.8 ppm
TPITT+ N3
m/z 1260.5699 +/- 15.8 ppm
TLPPTT + N3
m/z 1331.5707 +/- 15.8 ppm
TPSPPTT + N3
m/z 1363.6333 +/- 15.8 ppm
TVTPITT+ N3
m/z 1377.6489 +/- 15.8 ppm
TLTPITT + N3
m/z 1444.6547 +/- 15.8 ppm
TTPSPPTI, TPSPPITT, TPITPPTS + N3
m/z 1482.6186 +/- 15.8 ppm
TQTPTT + N4
m/z 1534.6499 +/- 15.8 ppm
TPSPPTT + N4
m/z 1566.7125 +/- 15.8 ppm
TVTPITT + N4
m/z 1580.7281 +/- 15.8 ppm
TLTPITT + N4
m/z 1607.7026 +/- 15.8 ppm
TVTPTPTG+ N4
m/z 1647.7339 +/- 15.8 ppm
TTPSPPTI, TPSPPITT, TPITPPTS + N4
m/z 1748.7816 +/- 15.8 ppm
TPITPPTST or TTPSPPITT + N4
m/z 1771.7709 +/- 15.8 ppm
TPITTTT + N5
m/z 1817.8394 +/- 15.8 ppm
TVTPIPTPTG or TVIPTPTPTG + N4
m/z 1935.8773 +/- 15.8 ppm
TQAPTPTAITT+ N4
m/z 1951.8608 +/- 15.8 ppm
TPTPTPIST + N5
m/z 1965.8764 +/- 15.8 ppm
TPTPTPITT + N5
m/z 2138.9565 +/- 15.8 ppm
TQAPTPTAITT + N5
m/z 2158.9349 +/- 15.8 ppm
TPTTTPIST, TPTSTPITT, STTPTPITT + N6
m/z 2211.014 +/- 15.8 ppm
TQTPTLTPITT + N5
m/z 2237.9885 +/- 15.8 ppm
TVTPTPTPTGTQ + N5
m/z 2241.9720 +/- 15.8 ppm
TVTPTPTPTS + N6
m/z 2255.9877 +/- 15.8 ppm
TPITPPTSTT or TPTPTPISTT + N6
m/z 2313.0091 +/- 15.8 ppm
TTVTPTPTPTG + N6
m/z 2384.0463 +/- 15.8 ppm
TQTPTPTPIST + N6
m/z 2388.0412 +/- 15.8 ppm
TQTPTSTPITT + N6
m/z 2398.0619 +/- 15.8 ppm
TQTPTPTPITT + N6
m/z 2402.0568 +/- 15.8 ppm
TQTPTTTPITT + N6
m/z 2414.0932 +/- 15.8 ppm
TQTPTLTPITT + N6
m/z 2423.0248 +/- 15.8 ppm
TPPTFSPFSTT + N6

x 1

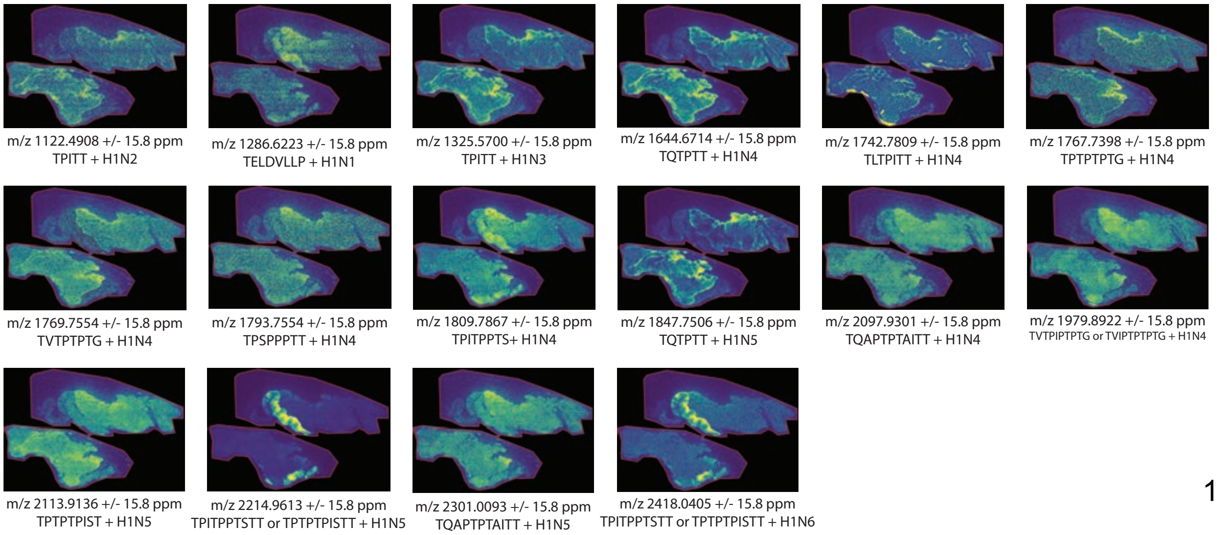
m/z 1122.4908 +/- 15.8 ppm
TPITT + H1N2
m/z 1286.6223 +/- 15.8 ppm
TELDVLLP + H1N1
m/z 1325.5700 +/- 15.8 ppm
TPITT + H1N3
m/z 1644.6714 +/- 15.8 ppm
TQTPTT + H1N4
m/z 1742.7809 +/- 15.8 ppm
TLTPITT + H1N4
m/z 1767.7398 +/- 15.8 ppm
TPTPTPTG + H1N4
m/z 1769.7554 +/- 15.8 ppm
TVTPTPTG + H1N4
m/z 1793.7554 +/- 15.8 ppm
TPSPPPTT + H1N4
m/z 1809.7867 +/- 15.8 ppm
TPITPPTS+ H1N4
m/z 1847.7506 +/- 15.8 ppm
TQTPTT + H1N5
m/z 2097.9301 +/- 15.8 ppm
TQAPTPTAITT + H1N4
m/z 1979.8922 +/- 15.8 ppm
TVTPIPTPTG or TVIPTPTPTG + H1N4
m/z 2113.9136 +/- 15.8 ppm
TPTPTPIST + H1N5
m/z 2214.9613 +/- 15.8 ppm
TPITPPTSTT or TPTPTPISTT + H1N5
m/z 2301.0093 +/- 15.8 ppm
TQAPTPTAITT + H1N5
m/z 2418.0405 +/- 15.8 ppm
TPITPPTSTT or TPTPTPISTT + H1N6

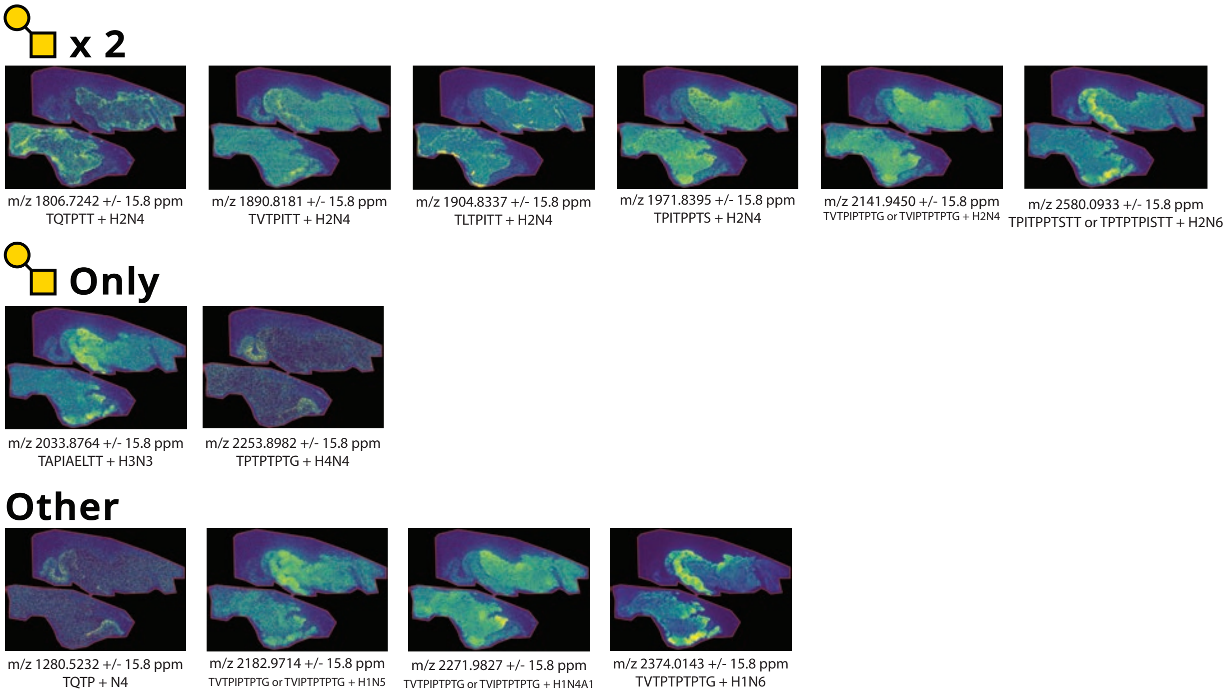

Supplementary Fig. 14. Additional glycopeptides detected by MALDI-MSI in the tumor region of StcE-treated Colon 2 that were subsequently identified by LC-MS. Images were acquired with a timsTOF fleX MALDI-2 mass spectrometer (Bruker). Each *m/z* value was manually extracted using SCiLS Lab version 2024b Pro (Bruker) and manually inspected for tumor localization prior to image export. The *m/z* values shown correspond to mono-sodiated precursor masses for the associated glycoform. Species were identified using LC-MS data collected for both of the tumor regions of Colon 2. Individual images with scale bars and color scales have been uploaded in the Source Data.

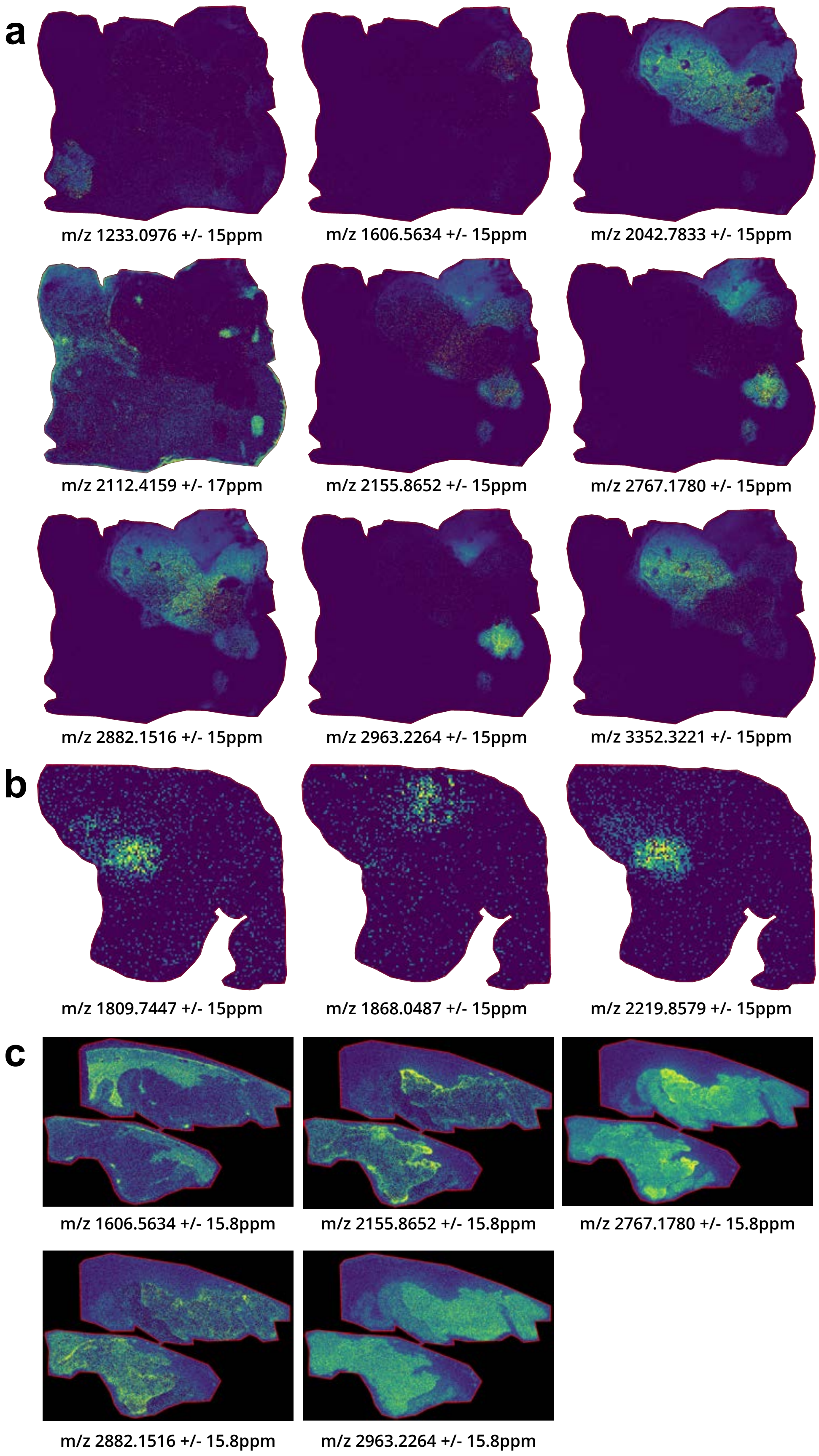
a
m/z 1233.0976 +/- 15ppm
m/z 1606.5634 +/- 15ppm
m/z 2042.7833 +/- 15ppm
m/z 2112.4159 +/- 17ppm
m/z 2155.8652 +/- 15ppm
m/z 2767.1780 +/- 15ppm
m/z 2882.1516 +/- 15ppm
m/z 2963.2264 +/- 15ppm
m/z 3352.3221 +/- 15ppm
b
m/z 1809.7447 +/- 15ppm
m/z 1868.0487 +/- 15ppm
m/z 2219.8579 +/- 15ppm
c
m/z 1606.5634 +/- 15.8ppm
m/z 2155.8652 +/- 15.8ppm
m/z 2767.1780 +/- 15.8ppm
m/z 2882.1516 +/- 15.8ppm
m/z 2963.2264 +/- 15.8ppm

Supplementary Fig. 15. Tumor-associated ions detected in PNGaseF- and StcE-treated colorectal tumors by MALDI-MSI that were not identified with LC-MS data. Each *m/z* value was manually extracted in SCiLS Lab version 2024b Pro (Bruker) and assessed for tumor localization prior to image export. (A) Nine unidentifiable species were detected at a strong intensity in Colon 1a. Images were acquired with a timsTOF fleX MALDI OR MALDI-2 QTOF mass spectrometer. (B) Three unidentifiable species detected at a strong intensity in Colon 1b. (C) Five unidentifiable species were detected at a strong intensity in Colon 2. Images were acquired with a Solarix dual-source 7T MALDI-FTICR mass spectrometer (Bruker) or timsTOF fleX MALDI-2 mass spectrometer (Bruker). Individual images with scale bars and color scales have been uploaded in the Source Data.

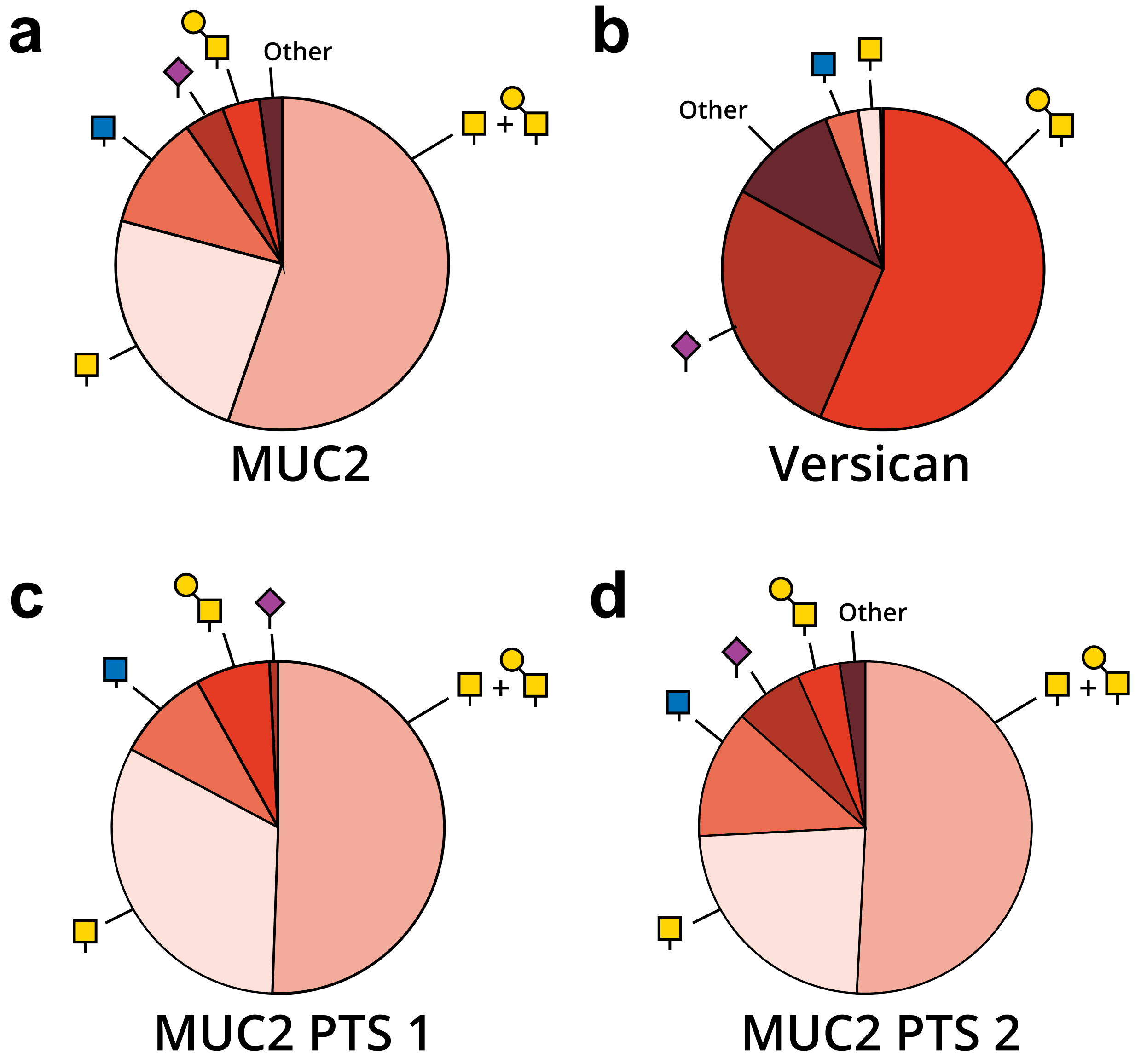

Supplementary Fig. 16. Pie charts showing distribution of identified glycoforms. All glycoforms recorded for each protein or protein domain were binned into one of six categories (Tn-only, Tn + T, T-only, GlcNAc-containing, sialylated, and other), and the total number of sequences in each category were summed. (A) MUC2 glycopeptides (N=698) can be categorized into Tn+T (365, 52.3%), Tn-only (173, 24.8%), GlcNAc-containing (81, 11.6%), sialylated (35, 5.0%), T-only (32, 4.6%), and other (12, 1.7%) species. (B) Versican glycopeptides (N=251) can be categorized into T-only (142, 56.6%), sialylated (67, 26.7%), other (28, 11.2%), GlcNAc-containing (8, 3.2%), and Tn-containing (6, 2.4%) species. (C) MUC2 PTS1 glycopeptides (N=174) can be categorized into Tn+T (88, 50.6%), Tn-only (56, 32.2%), GlcNAc-containing (16, 9.2%), T-only (13, 7.4%), and sialylated (1, 0.6%) species. (D) MUC2 PTS 2 glycopeptides (N=515) can be categorized into Tn+T (263, 51.1%), Tn-only (120, 23.3%), GlcNAc-containing (64, 12.4%), sialylated (34, 6.6%), T-only (22, 4.3%), and other (12, 2.3%) species. Data used to construct these charts can be found in Supplementary Data 2.

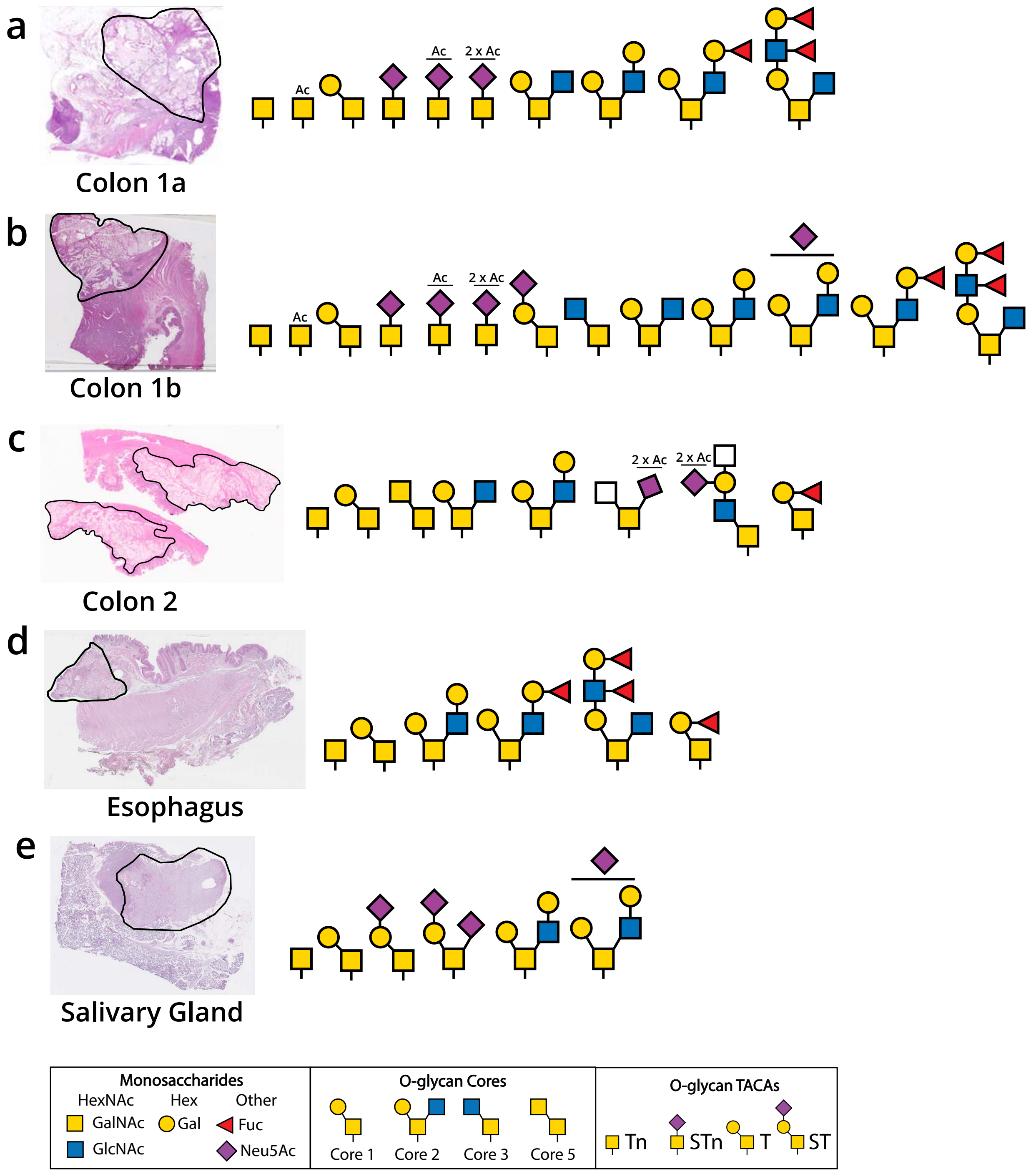

Supplementary Fig. 17. Glycan structures confidently localized in each tumor region (outlined in black) via LC-MS/MS. (A) From left to right: N1 (Tn), AcN1, H1N1 (T), N1A1 (sialyl-Tn), N1AcA1, $N1Ac_2A1$, H1N2, H2N2, H2N2F1, and H2N3F2 could be localized to specific sites on Colon 1a glycopeptides. (B) From left to right: N1 (Tn), AcN1, H1N1 (T), N1A1 (sialyl-Tn), N1AcA1, $N1Ac_2A1$, H1N1A1 (sialyl T), N2, H1N2, H2N2, H2N2A1, H2N2F1, and H2N3F2 could be

localized to specific sites on Colon 1b glycopeptides. (C) From left to right: N1 (Tn), H1N1 (T), N2, H1N2, H2N2, N2Ac$_2$A1, H2N3Ac$_2$A1, and H1N1F1 could be localized to specific sites on Colon 2 glycopeptides. White squares indicate ambiguous HexNAc. (D) From left to right: N1 (Tn), H1N1 (T), H2N2, H2N2F1, H2N3F2, and H1N1F1 could be localized to specific sites on Esophagus glycopeptides. (E) From left to right: N1 (Tn), H1N1 (T), H1N1A1 (sialyl T), H1N1A2 (di-sialyl T), H2N2, and H2N2A1 could be localized to specific sites on Salivary Gland glycopeptides.

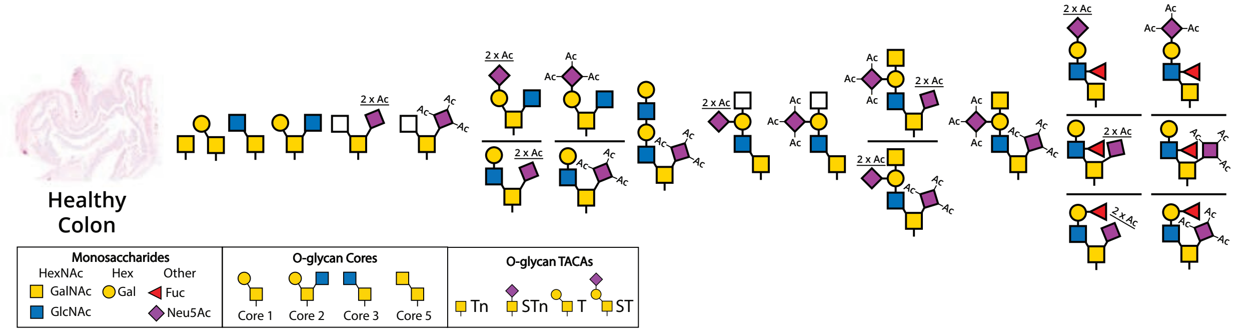

Supplementary Fig. 18. Glycan structures confidently localized on glycopeptides extracted from the Healthy Colon via LC-MS/MS. White squares indicate ambiguous HexNAcs. Vertically stacked glycans separated by lines indicate possible structural isomers based on monosaccharide composition. Ac, Acetyl. Proposed glycan structures are based on a previously published glycomic characterization of human intestinal MUC2-derived O-glycans.[5]

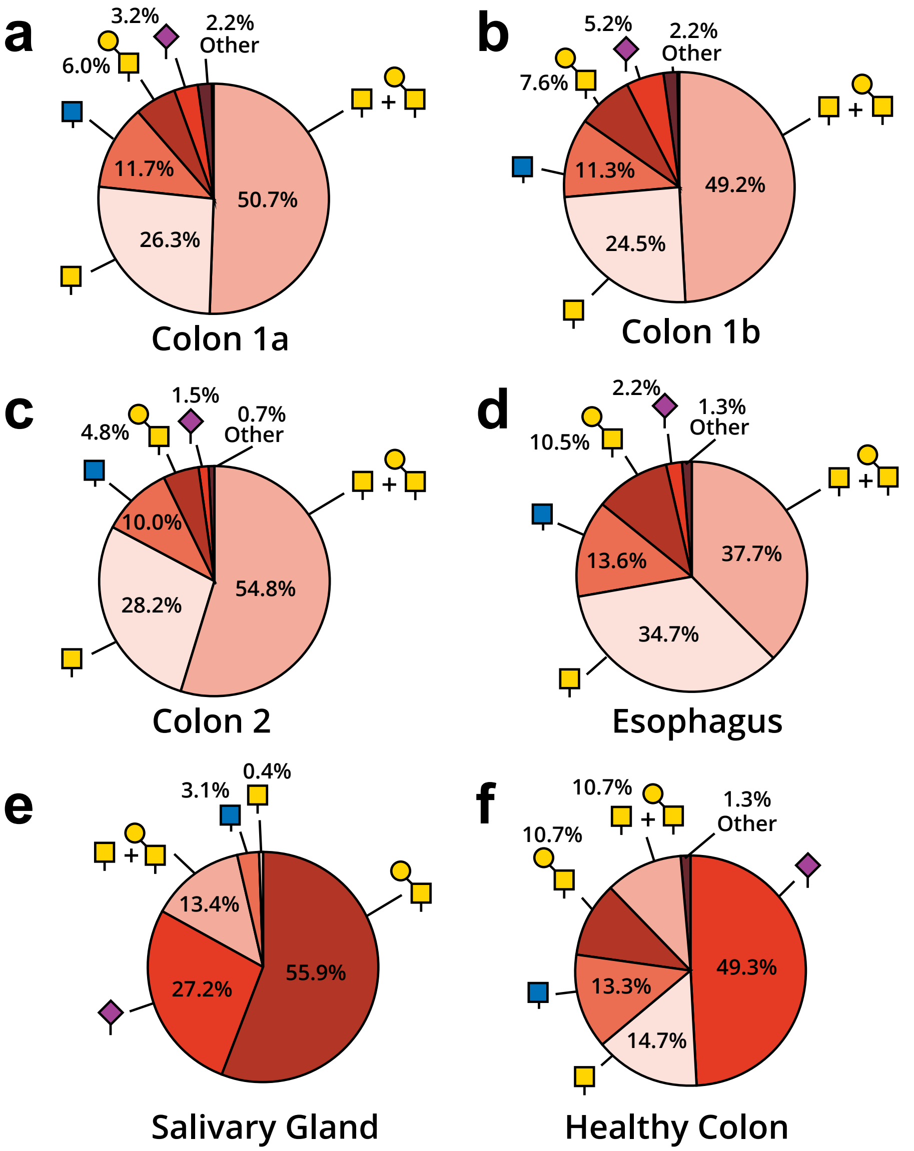

Supplementary Fig. 19. Pie charts showing distribution of glycoforms identified in each tumor region. (A) Colon 1a glycopeptides (N=635) can be categorized into Tn+T (322, 50.7%), Tn-only (167, 26.3%), GlcNAc-containing (74, 11.7%), T-only (38, 6.0%), sialylated (20, 3.2%), and other (14, 2.2%) species. (B) Colon 1b glycopeptides (N=636) can be categorized into Tn+T (313, 49.2%), Tn-only (156, 24.5%), GlcNAc-containing (72, 11.3%), T-only (48, 7.6%), sialylated (33, 5.2%), and other (14, 2.2%) species. (C) Colon 2 glycopeptides (N=588) can be categorized into Tn+T (322, 54.8%), Tn-only (166, 28.2%), GlcNAc-containing (59, 10.0%), T-only (28, 4.8%), sialylated (9, 1.5%), and other (4, 0.7%) species. (D) Esophagus glycopeptides (N=228) can be categorized into Tn+T (86, 37.7%), Tn-only (79, 34.7%), GlcNAc-containing (31, 13.6%), T-only (24, 10.5%), sialylated (5, 2.2%), and other (3, 1.3%) species. (E) Salivary gland glycopeptides (N=254) can be categorized into T-only (142, 55.9%), sialylated (69, 27.2%), Tn+T (34, 13.4%), GlcNAc-containing (8, 3.1%), and Tn-only (1, 0.4%) species. (F) Healthy Colon glycopeptides (N=75) can be categorized into sialylated (37, 49.3%), Tn-only (11, 14.7%), GlcNAc-containing (10, 13.3%), Tn+T (8, 10.7%), T-only (8, 10.7%), and other (1, 1.3%) species. Data used to construct these charts can be found in Supplementary Data 2.

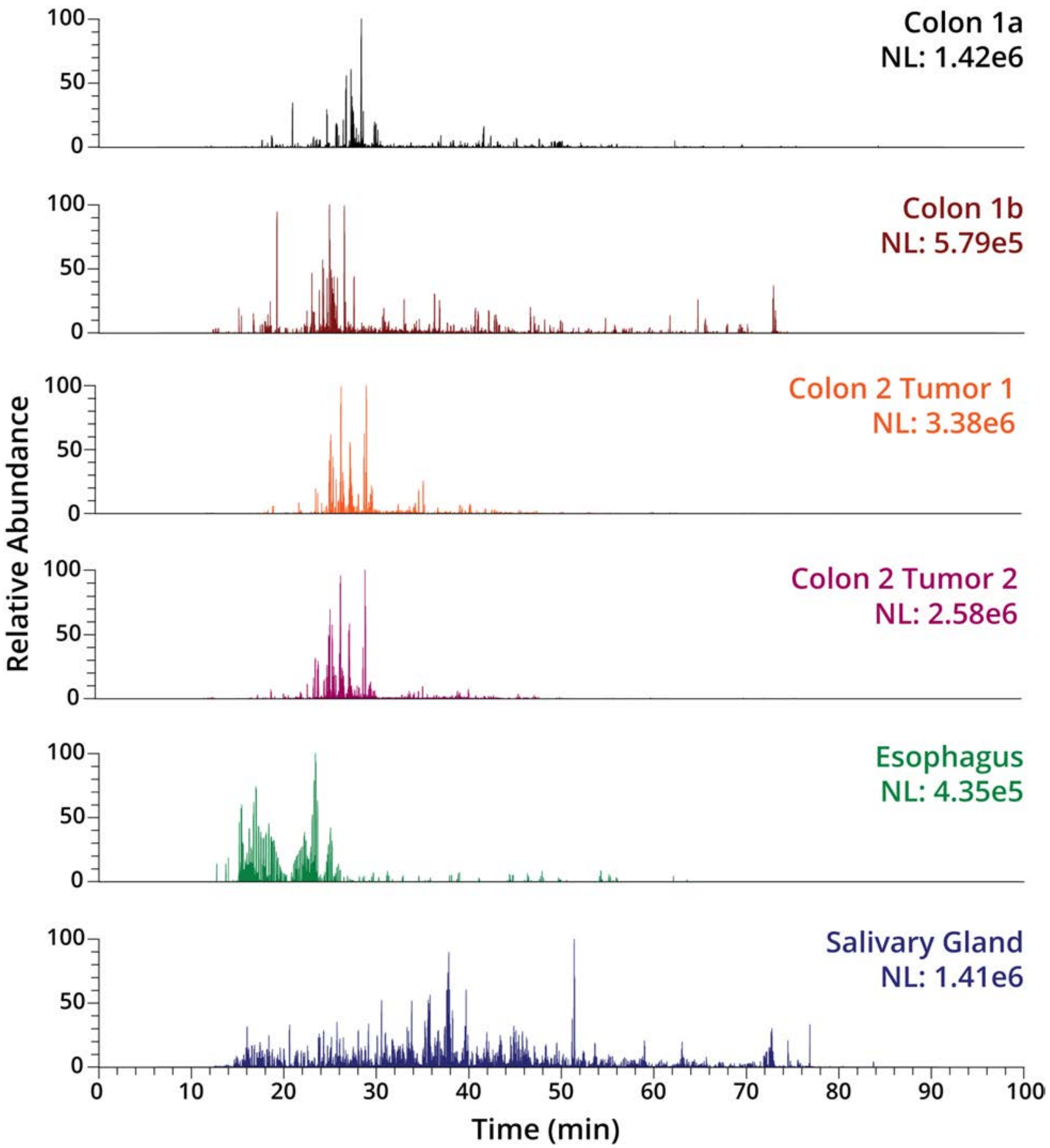

Supplementary Fig. 20. Combined traces of Neu5Ac fingerprint ions at *m/z* 292.1027 and 274.0921 in the tumor region of Colon 1a (black), Colon 1b (red), Colon 2 (orange & fuchsia), esophagus (green), and salivary gland (blue). All samples were prepared with the double-StcE workflow. NL, normalized level. Chromatograms were extracted from LC-MS data that have been made available in the PRIDE proteomics repository under the identifier PXD055865.

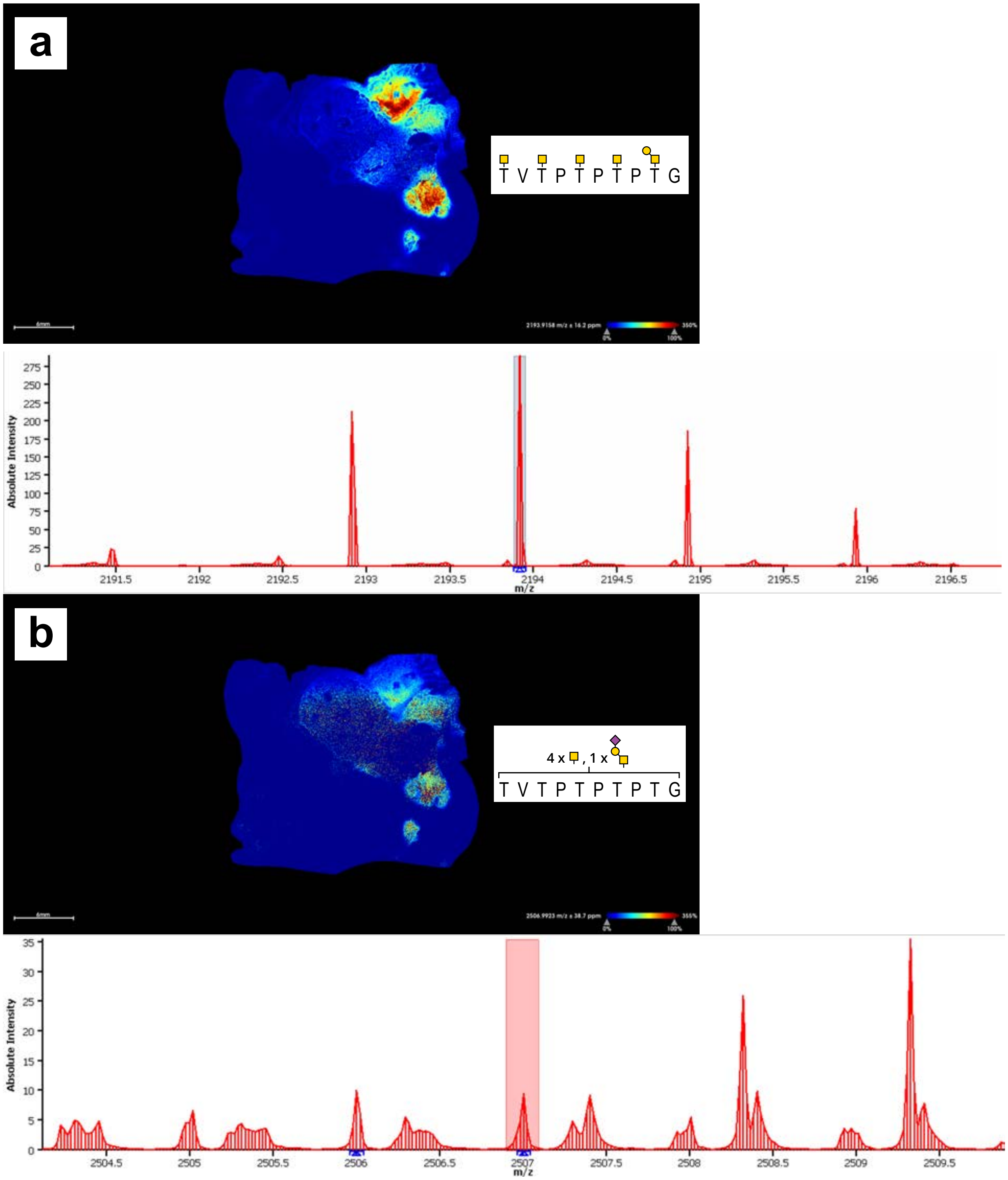

Supplementary Fig. 21. Sialylated MUC2 glycoforms were less intense than their non-sialylated counterparts in Colon 1a MALDI-MSI experiments. (A) Heat map of glycopeptide with backbone sequence TVTPTPTPTG from the second PTS domain of MUC2 decorated with one T and four Tn antigen structures. Note that this intensity map corresponds to the isotopic peak with a single C13 atom (bottom row), which is more intense than the monoisotopic precursor mass. (B) Heat map of glycopeptide with backbone sequence TVTPTPTPTG from the second PTS domain of

MUC2 decorated with one ST and four Tn antigen structures. For consistency with Panel A, the C13 peak of this species was also used to generate the intensity map, although the monoisotopic precursor mass is at roughly the same intensity (bottom row). MALDI-MSI data was acquired with a timsTOF fleX MALDI-QTOF mass spectrometer (Bruker). Ion images were manually extracted in SCiLS Lab version 2024b (Bruker). Images and MS1 spectra were extracted from MALDI-MSI data that have been made available in the PRIDE proteomics repository under the identifier PXD055865.

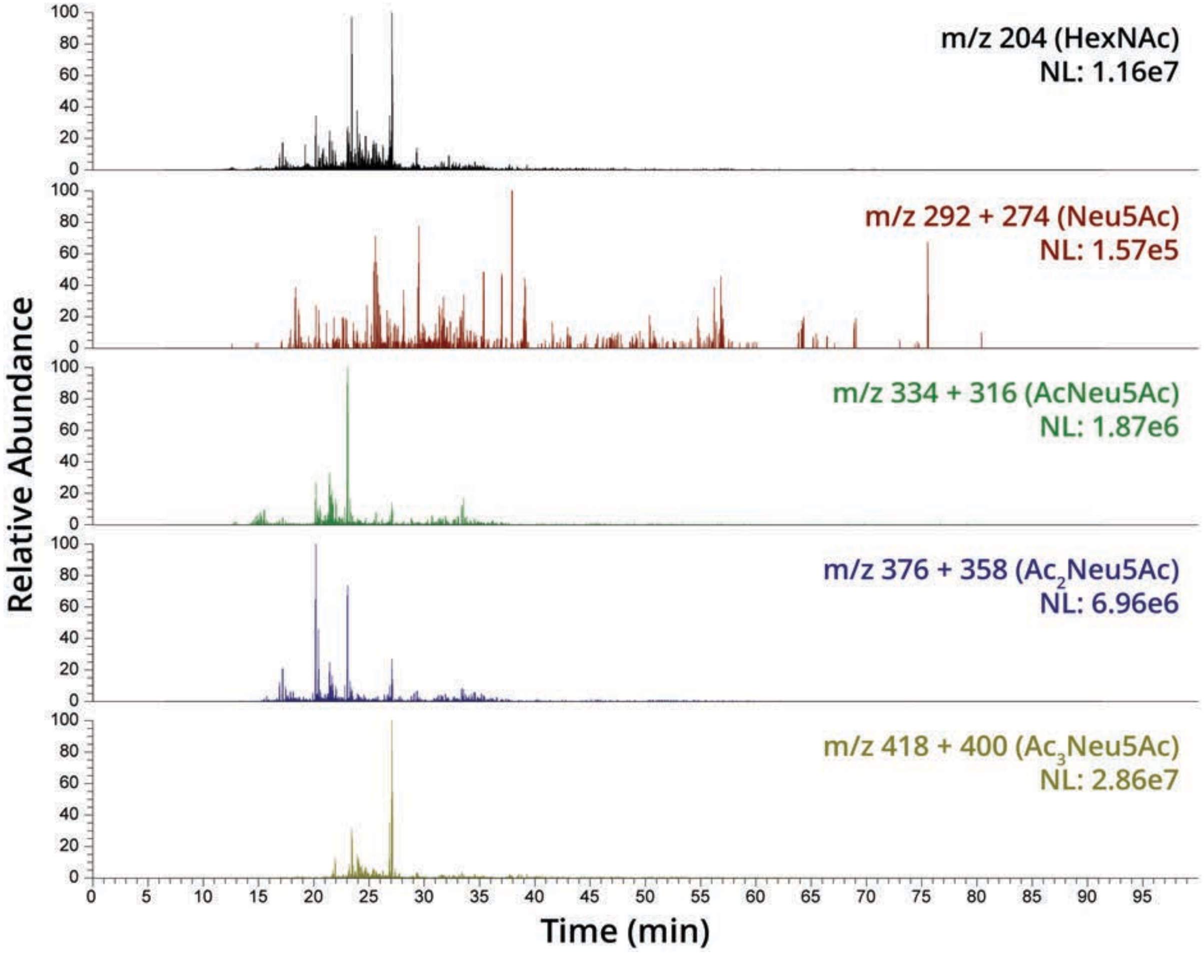

Supplementary Fig. 22. Chromatograms of AcNeu5Ac, Ac_2Neu5Ac, and Ac_3Neu5Ac fingerprint ions compared to overall HexNAc and Neu5Ac signal in the Healthy Colon sample. Chromatograms were extracted from LC-MS data that have been made available in the PRIDE proteomics repository under the identifier PXD055865.

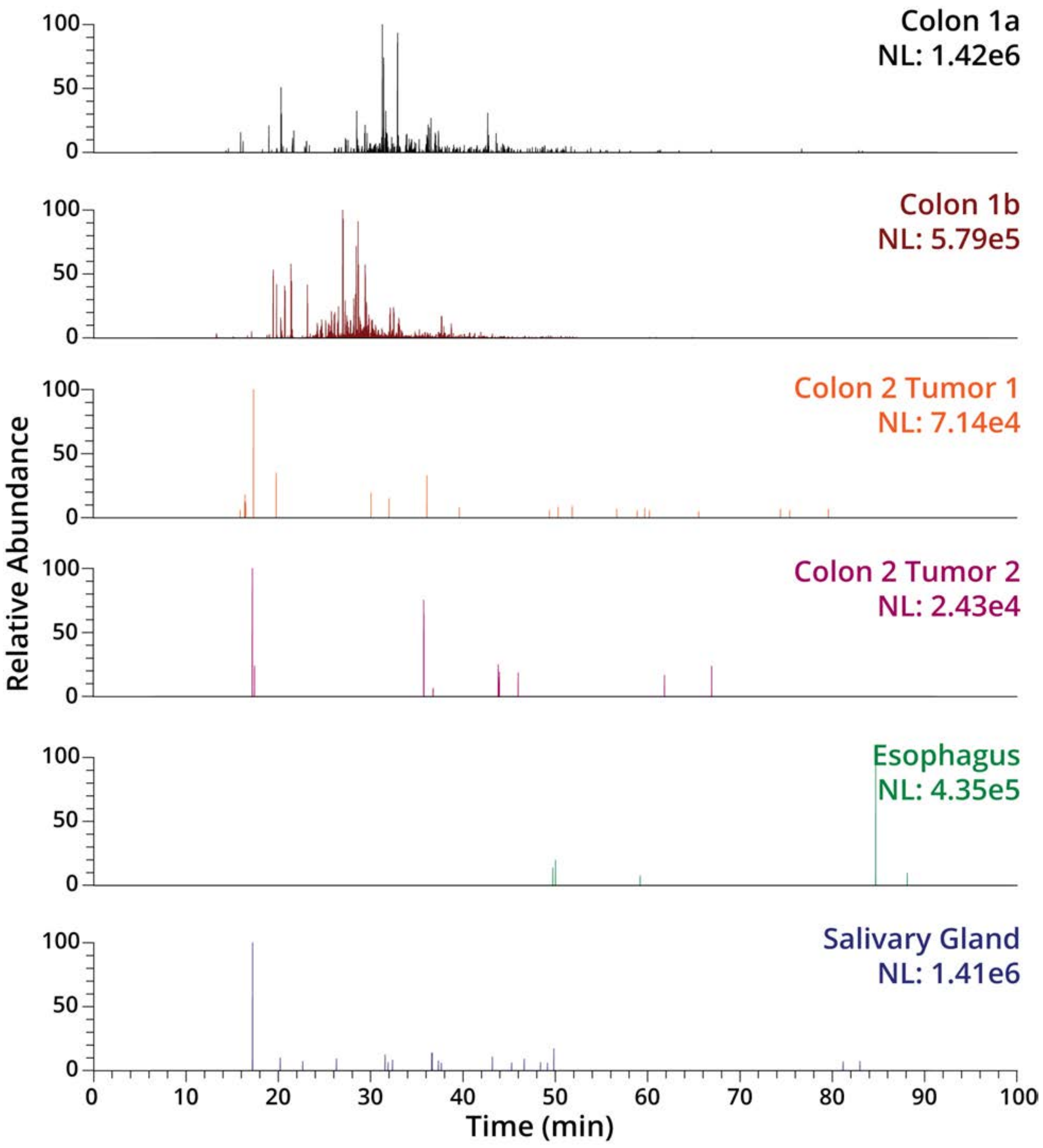

Supplementary Fig. 23. Combined traces of AcNeu5Ac fingerprint ions at *m/z* 334.1132 and 316.1026 for the tumor regions of Colon 1a (black), Colon 1b (red), Colon 2 (orange and fuchsia), esophagus (green), and salivary gland (blue). All samples shown were prepared with the double-StcE workflow. NL, normalized level. Chromatograms were extracted from LC-MS data that have been made available in the PRIDE proteomics repository under the identifier PXD055865.

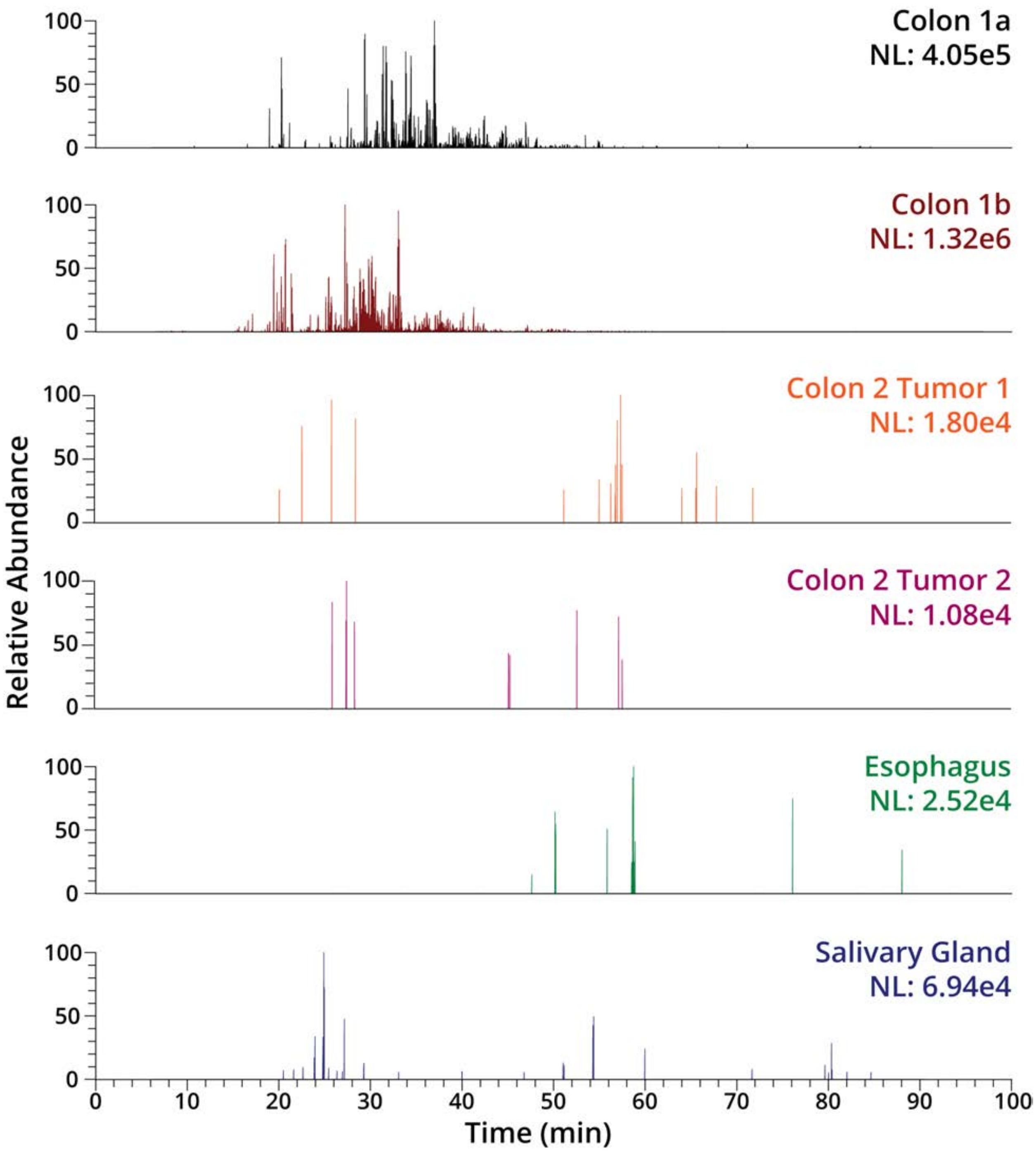

Supplementary Fig. 24. Combined traces of Ac_2Neu5Ac fingerprint ions at *m/z* 376.1237 and 358.1131 for the tumor regions of Colon 1a (black), Colon 1b (red), Colon 2 (orange and fuchsia), esophagus (green), and salivary gland (blue). All samples shown were prepared with the double-StcE workflow. NL, normalized level. Chromatograms were extracted from LC-MS data that have been made available in the PRIDE proteomics repository under the identifier PXD055865.

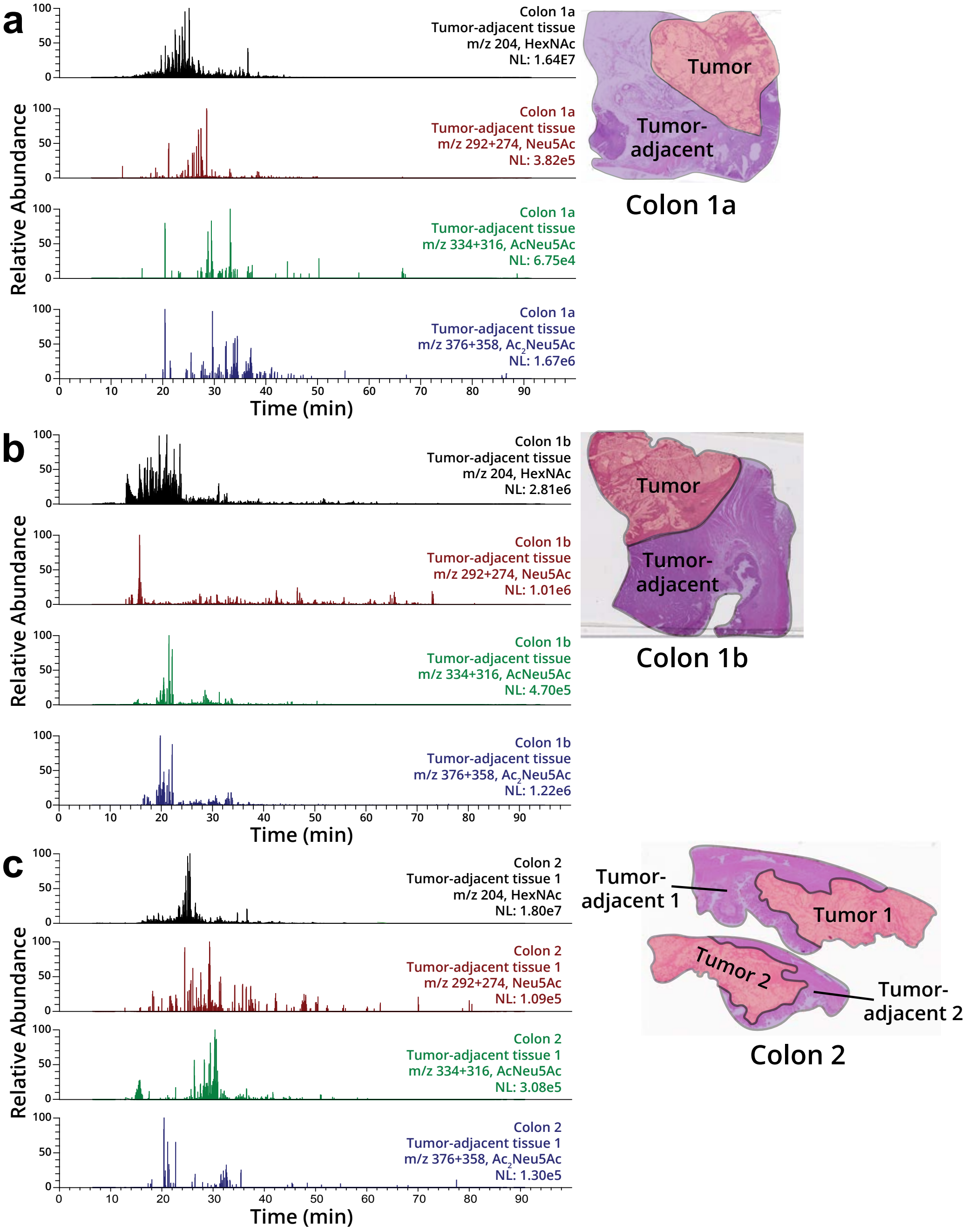

Supplementary Fig. 25. Traces of AcNeu5Ac and Ac_2Neu5Ac fingerprint ions in tumor-adjacent regions. (A) Ion traces for HexNAc (black), Neu5Ac (red), AcNeu5Ac (green), and Ac_2Neu5Ac (blue) for the tumor-adjacent region of Colon 1a prepared with the double-StcE workflow. (B) Ion traces for HexNAc (black), Neu5Ac (red), AcNeu5Ac (green), and Ac_2Neu5Ac (blue) for the tumor-adjacent region of Colon 1b prepared with the double-StcE workflow. (C) Ion traces for HexNAc (black), Neu5Ac (red), AcNeu5Ac (green), and Ac_2Neu5Ac (blue) for tumor-adjacent region 1 of Colon 2 prepared with the double-StcE workflow. NL, normalized level. Chromatograms were extracted from LC-MS data that have been made available in the PRIDE proteomics repository under the identifier PXD055865.

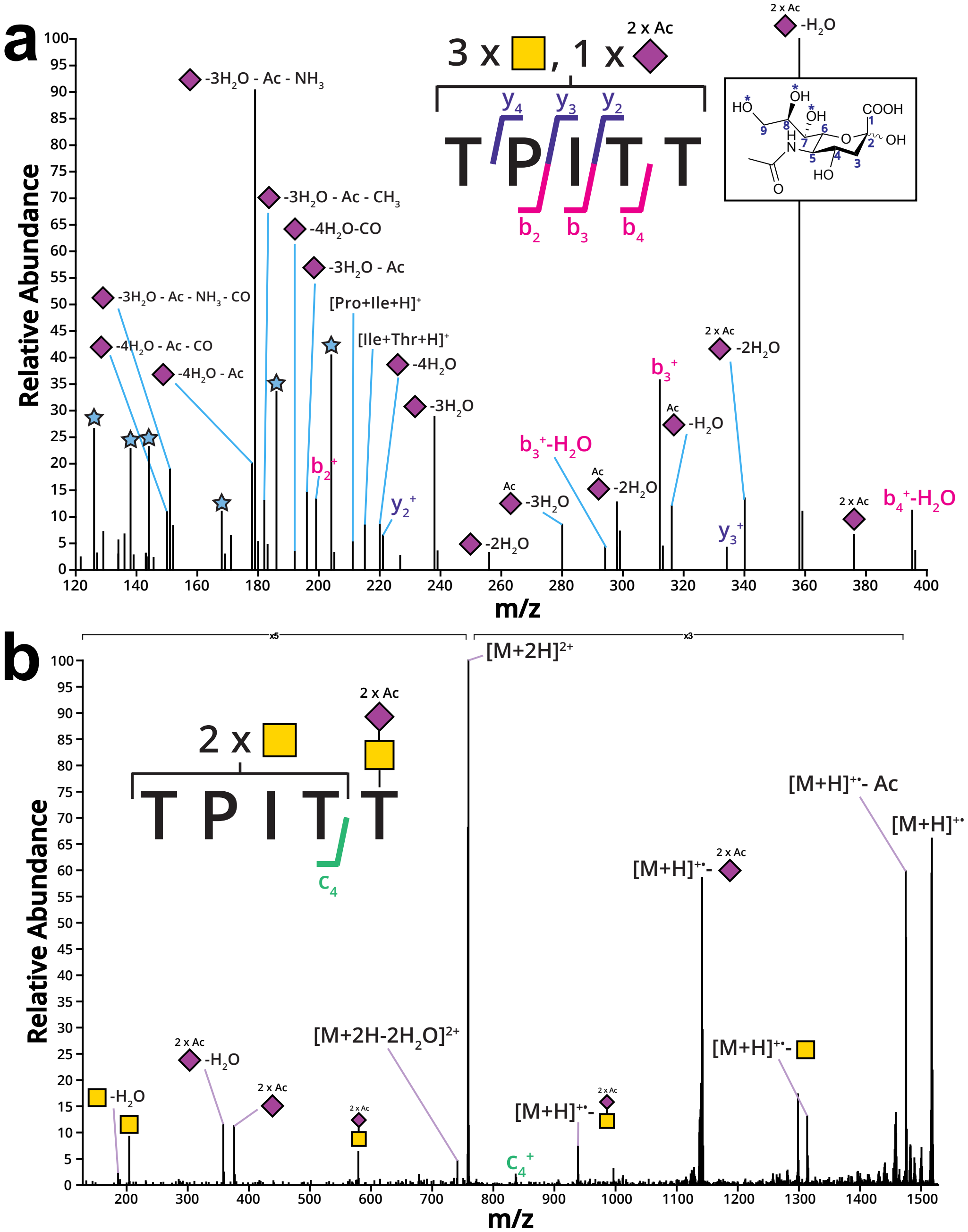

Supplementary Fig. 26. Additional MS2 evidence for Ac_2Neu5Ac-containing glycopeptide. These spectra are from the non-tumor region data for Colon 1b prepared with the double-StcE workflow and are representative of other spectra for Ac_2Neu5Ac species. (A) Low-mass region (*m/z* 120-400) of the higher-energy collision dissociation (HCD) spectrum shown in Figure 4A for the MUC2 glycopeptide with sequence TPITT, demonstrating the rich fragmentation of the Ac_2Neu5Ac moiety. Blue stars represent HexNAc fingerprint ions. (B) Electron-transfer dissociation (ETD) spectrum for the same precursor ion. Theoretical and experimental masses along with calculated error for each fragment is provided in Supplementary Table 1. All fragment *m/z* values and corresponding relative intensities for each spectrum are provided in the Source Data.

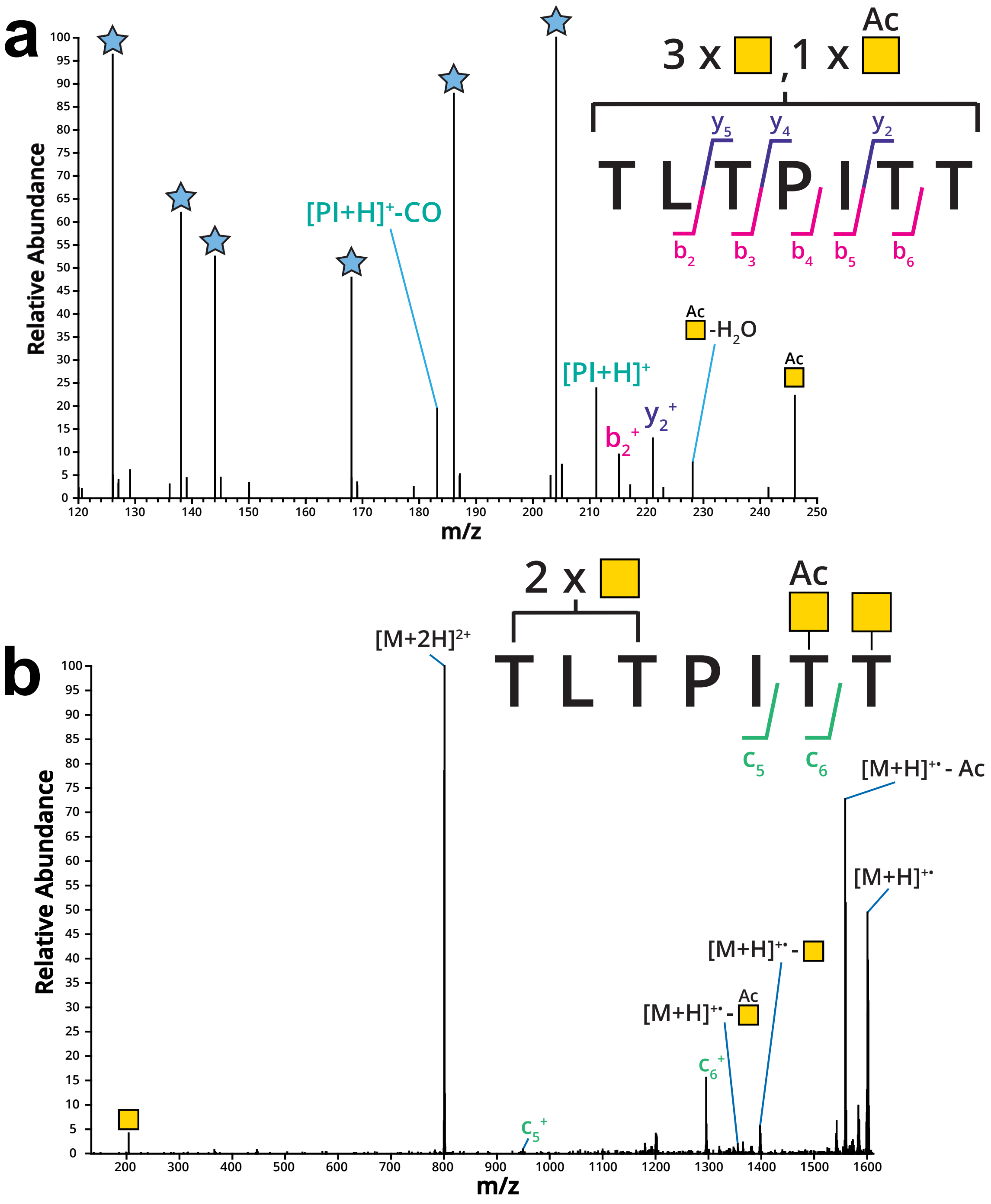

Supplementary Fig. 27. Additional MS2 evidence for glycopeptide decorated with acetylated GalNAc. (A) Low-mass region of HCD spectrum shown in Figure 4B for the MUC2 glycopeptide with sequence TLTPITT, showing fragmentation of a putative AcGalNAc-containing glycopeptide. Fingerprint ions for the intact AcGalNAc structure as well as its water loss were detected. (B) ETD spectrum for the same precursor. Spectra are from tumor region LC-MS data for Colon 1b prepared with the double-StcE workflow. Theoretical and experimental masses along with calculated error for each fragment is provided in Supplementary Table 2. All fragment *m/z* values and corresponding relative intensities from each spectrum can be found in the Source Data.

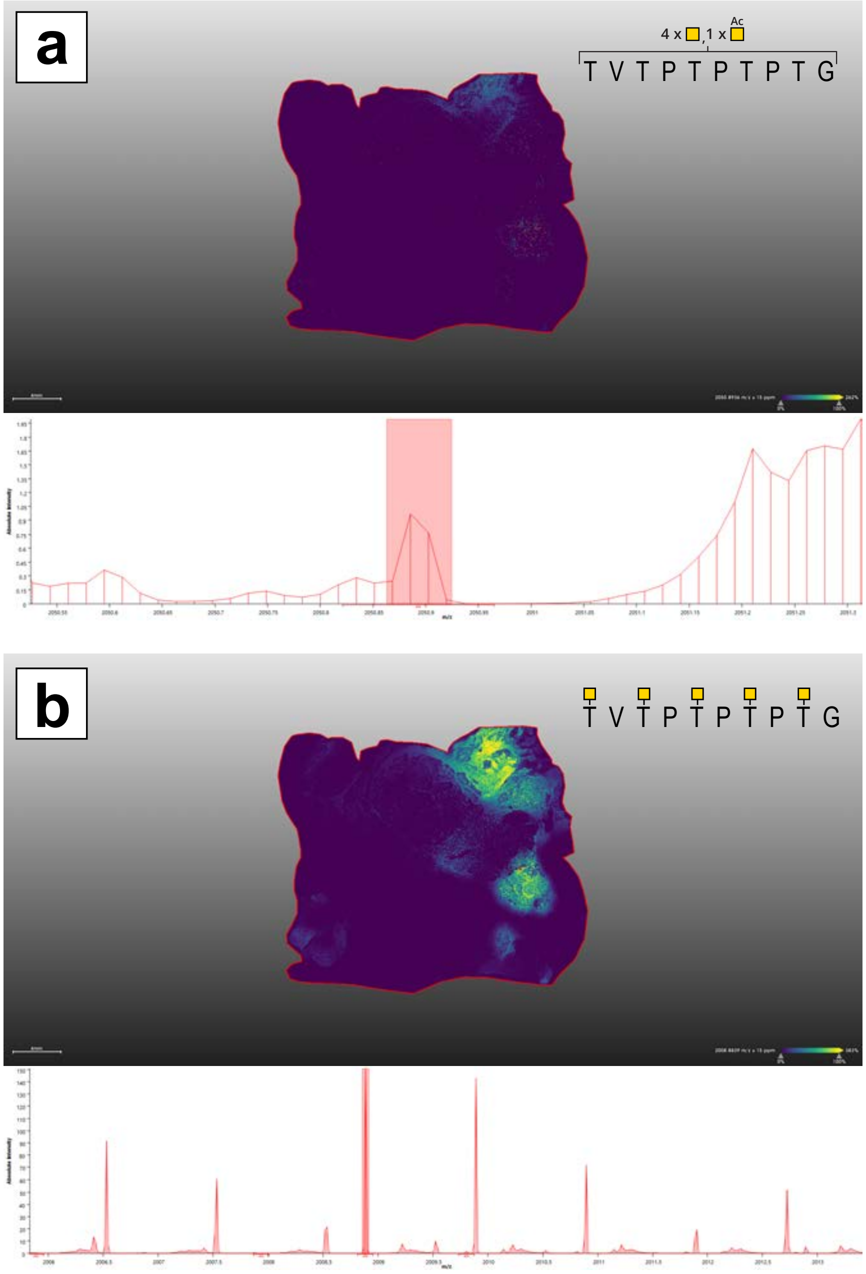

Supplementary Fig. 28. A MUC2 glycoform with AcGalNAc overlaps with Tn-only glycoform expression in MALDI-MSI of Colon 1a. This tissue section was treated with PNGaseF and StcE prior to data acquisition with a timsTOF fleX MALDI-QTOF mass spectrometer (Bruker). Precursor ions detected in the tumor region of double-StcE-treated Colon 1a with LC-MS were converted to mono-sodiated *m/z* values, which were manually extracted in MALDI-MSI data using SCiLS Lab version 2024b Pro (Bruker). (A) Heat map for the mass corresponding to the glycopeptide TVTPTPTPTG containing four Tn antigens and one AcGalNAc structure, which had a very low overall intensity (bottom row). This spatial distribution overlapped with the highest expression of the Tn-only glycoform shown in (B), which had a much higher overall abundance (bottom row). Images and MS1 spectra were extracted from MALDI-MSI data that have been made available in the PRIDE proteomics repository under the identifier PXD055865.

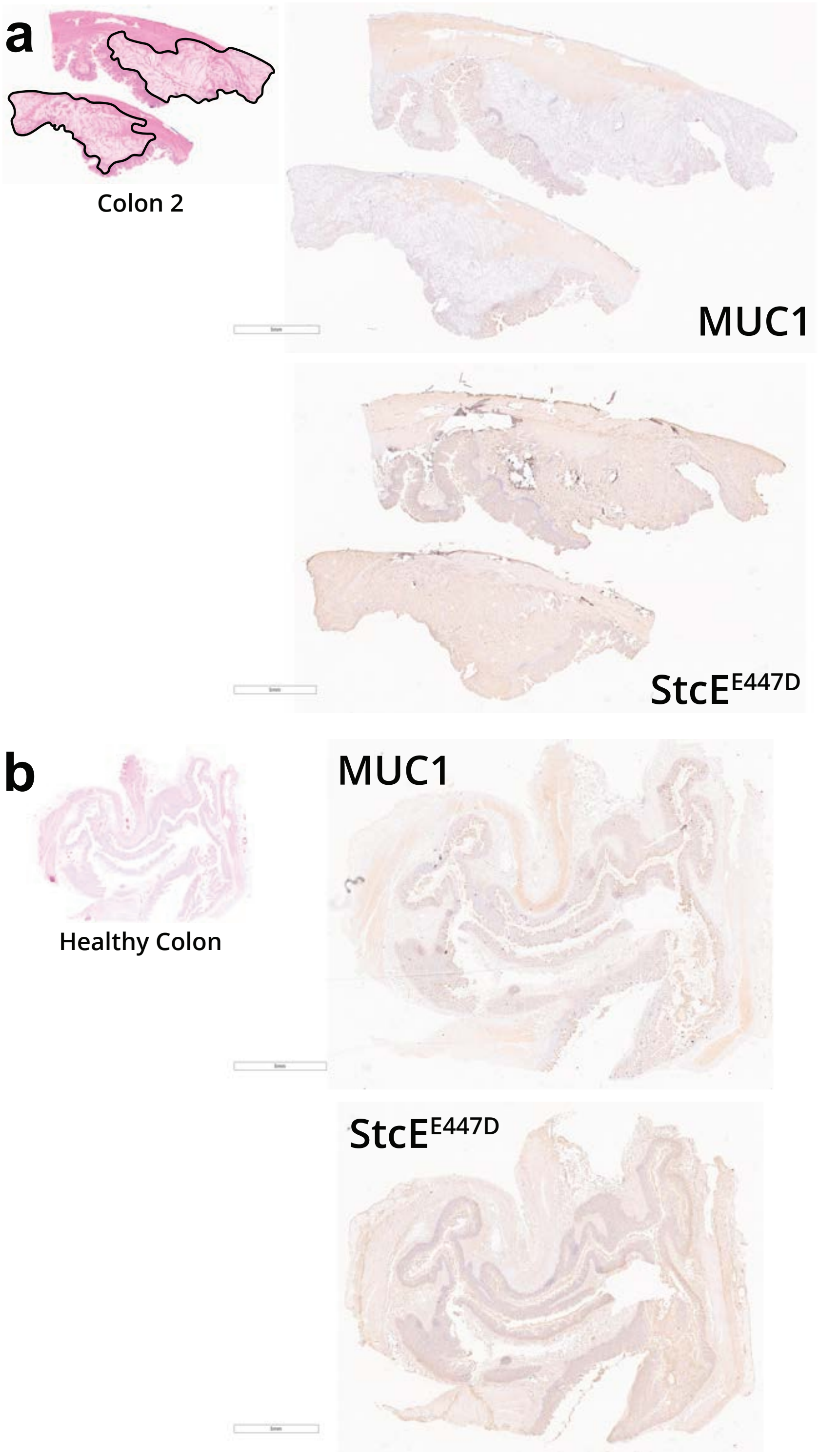

Supplementary Fig. 29. Anti-MUC1 immunohistochemistry and staining with catalytically inactive StcE^{E447D} of serial sections from (A) Colon 2 and (B) the Healthy Colon with 5-mm scale bars. Results are representative of a single experiment. High-resolution raw image files have been uploaded with the Source Data.

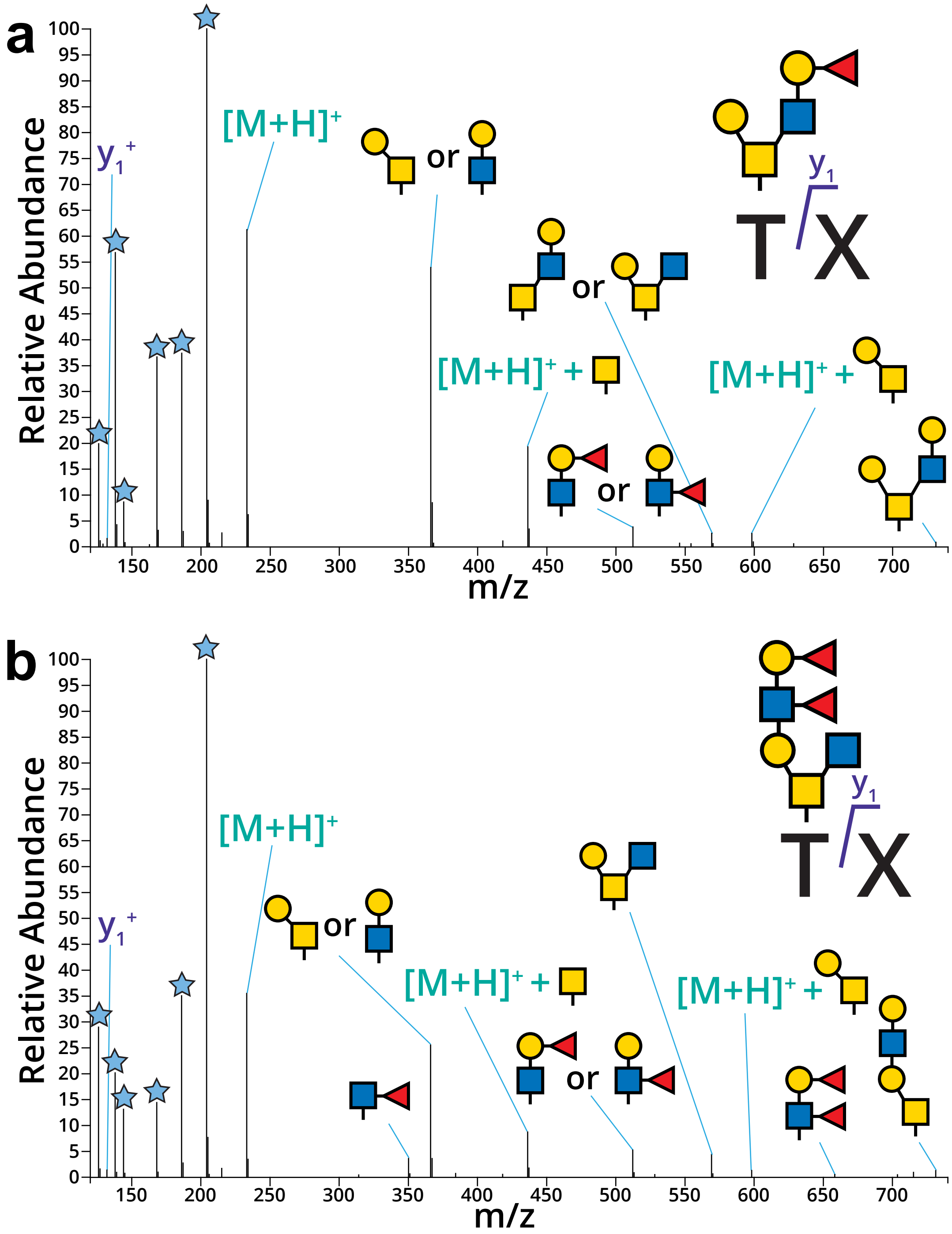

Supplementary Fig. 30. O-Glycosylated dipeptides were detected by LC-MS in the tumor region of the esophageal tissue prepared with the double-StcE workflow. (A) HCD spectrum for the ion at a retention time of 18.22 minutes with *m/z* 555.2397, corresponding to the +2 precursor of dipeptide TL or TI most likely decorated with a mono-fucosylated core 2 O-glycan. (B) HCD spectrum for the ion at a retention time of 19.41 minutes with *m/z* 729.8077, corresponding to the +2 precursor of dipeptide TL or TI most likely decorated with a core 2 O-glycan likely extended with a Le^{b} or Le^{y} motif. Blue stars in low-mass region correspond to HexNAc fingerprint ions. All fragment *m/z* values and corresponding relative intensities from each spectrum are provided in the Source Data.

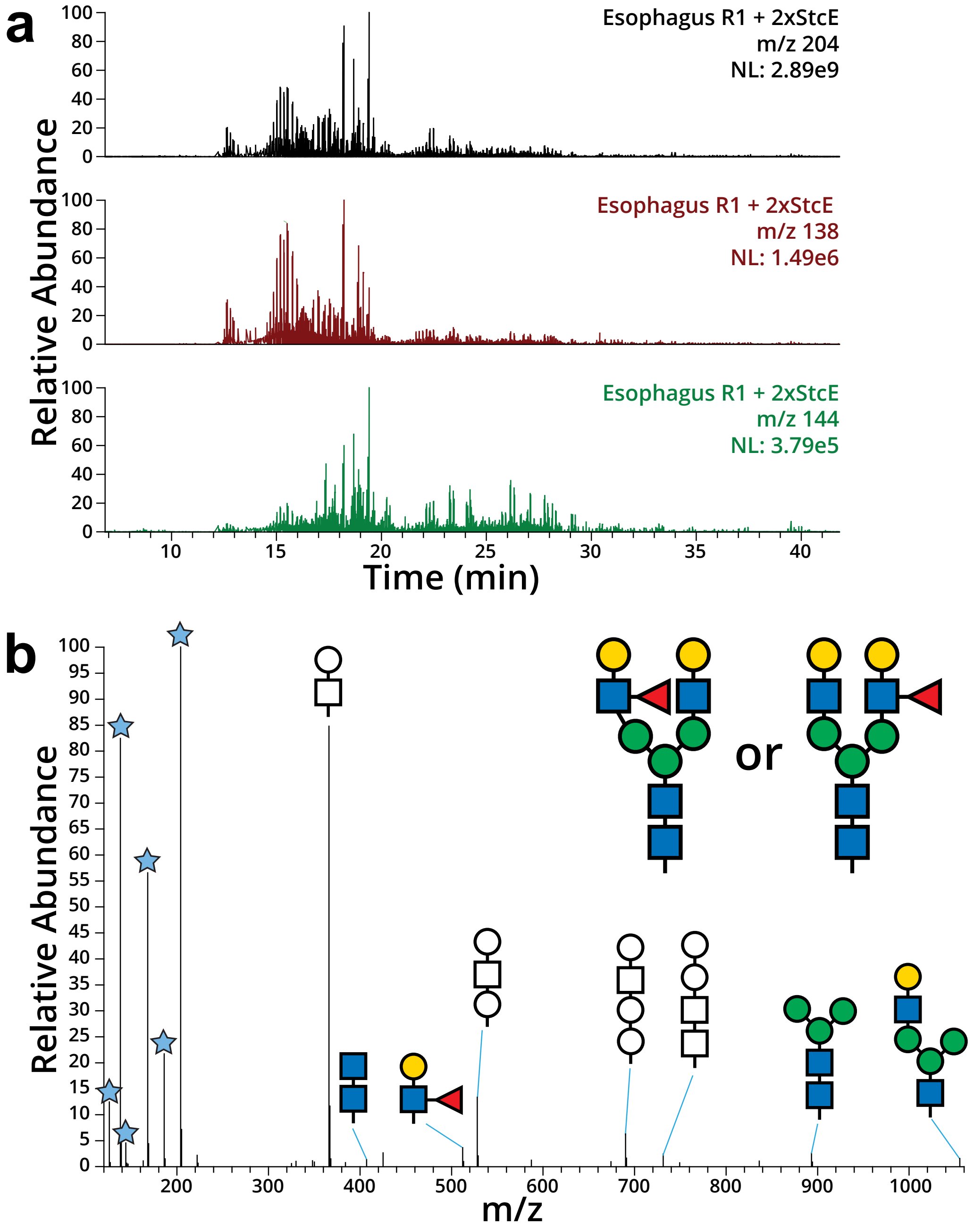

Supplementary Fig. 31. Free N-glycans were relatively abundant in the LC-MS data for the double-StcE-treated esophageal tumor region. (A) Traces of the HexNAc marker ions at *m/z* 204.0867 (black), 138.055 (red), and 144.0655 (green). (B) HCD spectrum for the ion at a retention time of 15.19 minutes and with *m/z* 894.3327, corresponding to the +2 precursor for the N-glycan with composition H5N4F1. Uncolored shapes indicate glycan fragments that cannot be attributed to a specific portion of the N-glycan structure. Blue stars in the low-mass region correspond to HexNAc fingerprint ions. The chromatogram depicted in (A) was extracted from LC-MS data that have been made available in the PRIDE proteomics repository under the identifier PXD055865. All fragment m/z values and corresponding relative intensities from the spectrum shown in (B) have been provided in the Source Data.

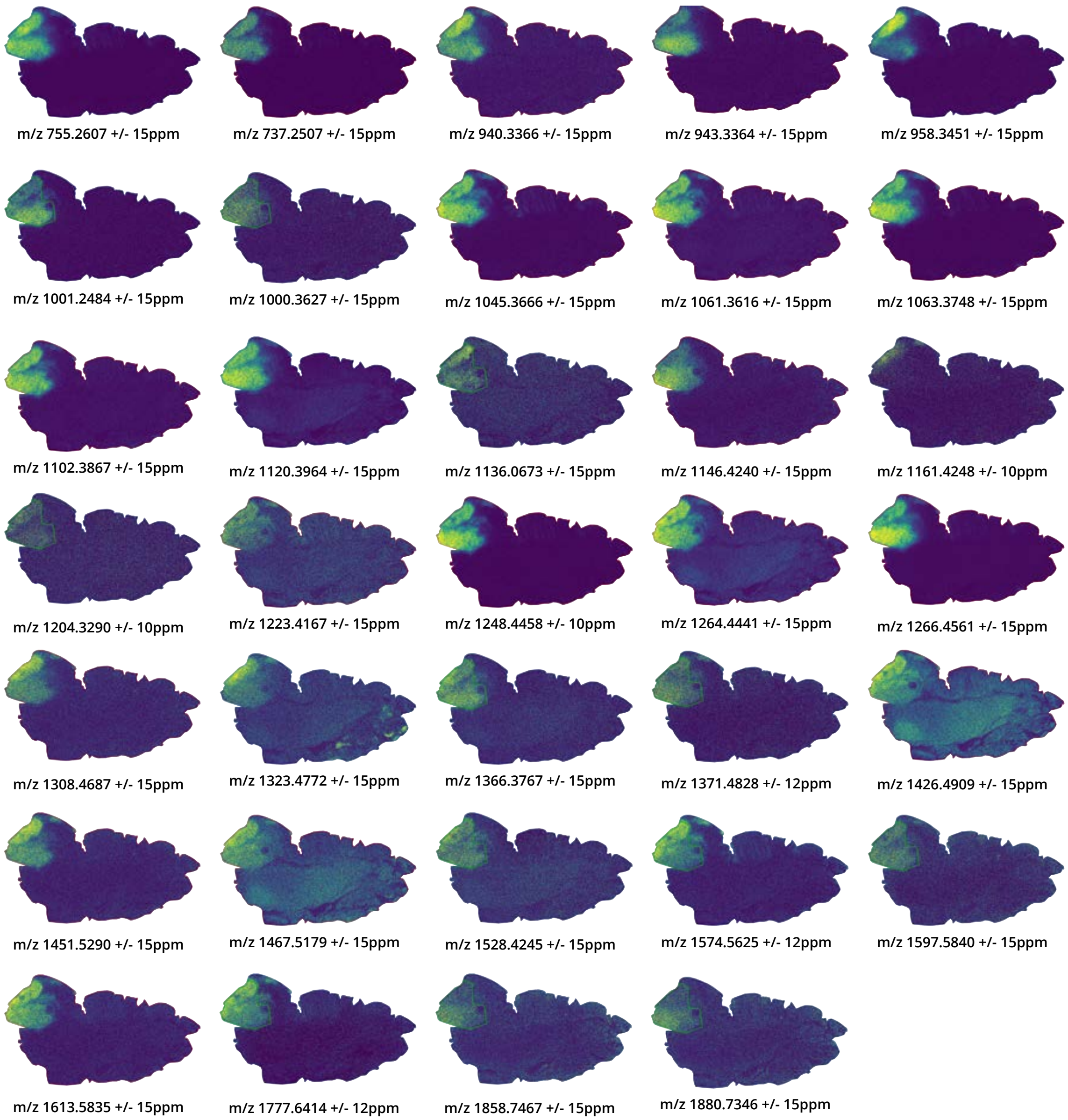

Supplementary Fig. 32. Tumor-associated ions detected by MALDI-MSI of the PNGaseF- and StcE-treated esophageal tissue. Raw data was acquired with a timsTOF fleX MALDI-QTOF mass spectrometer (Bruker). Each *m/z* value was manually extracted in SCiLS Lab version 2024b Pro (Bruker) and inspected for tumor localization prior to image export. The depicted species could not be identified with corresponding LC-MS data. Images were extracted from MALDI-MSI data that have been made available in the PRIDE proteomics repository under the identifier PXD055865. Individual images with scale bars and color scales have been uploaded in the Source Data.

Supplementary Table 1. Theoretical monoisotopic masses and subsequently detected exact masses of glycan fingerprint ions following higher energy collisional dissociation (HCD) of the sequence TPITT + N3Ac$_2$A1 shown in Figure 4a and Supplementary Figs. 29a-b.

Glycan Composition	Chemical Formula	Calculated Monoisotopic Mass	Detected Exact Mass	Error (ppm)
HexNAc-$C_2H_6O_3$	$[C_6H_8NO_2]^+$	126.0550	126.0543	-5.74
HexNAc-CH_6O_3	$[C_7H_8NO_2]^+$	138.0550	138.0544	-4.48
HexNAc-$C_2H_4O_2$	$[C_6H_{10}NO_3]^+$	144.0655	144.0652	-2.18
HexNAc-2H_2O	$[C_8H_{10}NO_3]^+$	168.0655	168.0647	-4.96
HexNAc-H_2O	$[C_8H_{12}NO_4]^+$	186.0761	186.0759	-1.25
HexNAc	$[C_8H_{14}NO_5]^+$	204.0867	204.0865	-0.74
Neu5Ac-4H_2O-Ac-CO	$[C_8H_8NO_2]^+$	150.0551	150.0534	-11.22
Neu5Ac-3H_2O-Ac-NH_3-CO	$[C_8H_7O_3]^+$	151.0392	151.0386	-4.38
Neu5Ac-4H_2O-Ac	$[C_9H_8NO_3]^+$	178.0500	178.0494	-3.61
Neu5Ac-3H_2O-Ac-NH_3	$[C_9H_7O_4]^+$	179.0341	179.0334	-3.84
Neu5Ac-3H_2O-Ac-CH_3	$[C_8H_7NO_4]^+$	182.0449	182.0444	-2.75
Neu5Ac-4H_2O-CO	$[C_{10}H_{10}NO_3]^+$	192.0657	192.0643	-7.13
Neu5Ac-3H_2O-Ac	$[C_9H_{10}NO_4]^+$	196.0605	196.0603	-1.34
Neu5Ac-4H_2O	$[C_{11}H_{10}NO_4]^+$	220.0606	220.0613	3.36
Neu5Ac-3H_2O	$[C_{11}H_{12}NO_5]^+$	238.0711	238.0704	-2.86
Neu5Ac-2H_2O	$[C_{11}H_{14}NO_6]^+$	256.0816	256.0798	-7.02
Neu5Ac-H_2O	$[C_{11}H_{16}NO_7]^+$	274.0921	Not Detected	n/a
Neu5Ac	$[C_{11}H_{18}NO_8]^+$	292.1027	Not Detected	n/a
AcNeu5Ac-3H_2O	$[C_{13}H_{14}NO_6]^+$	280.0818	280.0815	-0.79
AcNeu5Ac-2H_2O	$[C_{13}H_{16}NO_7]^+$	298.0923	298.0914	-3.00
AcNeu5Ac-H_2O	$[C_{13}H_{18}NO_8]^+$	316.1028	316.1023	-1.49
AcNeu5Ac	$[C_{13}H_{20}NO_9]^+$	334.1133	Not Detected	n/a
Ac$_2$Neu5Ac-2H_2O	$[C_{15}H_{18}NO_8]^+$	340.1028	340.1055	7.76
Ac$_2$Neu5Ac-H_2O	$[C_{15}H_{20}NO_9]^+$	358.1133	358.1131	-0.73
Ac$_2$Neu5Ac	$[C_{15}H_{22}NO_{10}]^+$	376.1238	376.1210	-7.60

Supplementary Table 2. Theoretical monoisotopic masses and subsequently detected exact masses of glycan fingerprint ions following higher energy collisional dissociation (HCD) of the sequence TLTPITT + N3AcN1 shown in Figure 4b and Supplementary Fig. 30a-b.

Glycan Composition	Chemical Formula	Calculated Monoisotopic Mass	Detected Exact Mass	Error (ppm)
HexNAc-$C_2H_6O_3$	$[C_6H_8NO_2]^+$	126.0550	126.0545	-4.36
HexNAc-CH_6O_3	$[C_7H_8NO_2]^+$	138.0550	138.0545	-3.37
HexNAc-$C_2H_4O_2$	$[C_6H_{10}NO_3]^+$	144.0655	144.0651	-2.60
HexNAc-2H_2O	$[C_8H_{10}NO_3]^+$	168.0655	168.0652	-1.60
HexNAc-H_2O	$[C_8H_{12}NO_4]^+$	186.0761	186.0761	-0.19
HexNAc	$[C_8H_{14}NO_5]^+$	204.0867	204.0868	0.30
AcHexNAc-H_2O	$[C_{10}H_{14}NO_5]^+$	228.0868	228.0883	6.61
AcHexNAc	$[C_{10}H_{16}NO_6]^+$	246.0973	246.0975	0.97

Supplementary Table 3. Extracellular proteases and peptidases identified in tumor regions of cancer tissues or in Healthy Colon sample using unmodified peptide data. C1a, Colon 1a; C1b, Colon 1b; C2, Colon 2; E, Esophagus; SG, Salivary Gland; HC, Healthy Colon.

UniProt ID	Description	C1a	C1b	C2	E	SG	HC
P61626	Lysozyme C	+	+	+	+	+	+
P07339	Cathepsin D	+	+	+	+	+	+
P08311	Cathepsin G	+	+	+	+	+	+
P15088	Mast cell carboxypeptidase A	-	-	+	+	+	+
P14780	Matrix metalloproteinase-9	-	+	+	+	+	-
Q9H4A4	Aminopeptidase B	-	+	+	-	+	+
P15144	Aminopeptidase N	-	-	-	-	+	-
P20231	Tryptase beta-2	+	+	-	+	-	+
P16444	Dipeptidase 1	+	+	-	-	-	-
P17655	Calpain-2 catalytic subunit	+	+	+	-	-	+
P04632	Calpain small subunit 1	+	+	+	-	-	+
P25774	Cathepsin S	+	-	+	-	-	+
P12955	Xaa-Pro dipeptidase	+	-	+	-	-	+
P23946	Chymase	-	-	-	+	-	+
P24158	Myeloblastin	-	+	+	-	-	-
Q96IY4	Carboxypeptidase B2	-	+	+	-	-	+
P08217	Chymotrypsin-like elastase family member 2A	-	+	-	-	-	-
Q92542	Nicastrin	-	+	+	-	-	+
Q9H3G5	Probable serine carboxypeptidase CPVL	-	-	-	+	-	-
Q9UHL4	Dipeptidyl peptidase 2	-	-	+	+	-	-
Q9NZ08	Endoplasmic reticulum aminopeptidase 1	+	+	+	+	+	+
Q9ULA0	Aspartyl aminopeptidase	+	+	+	+	+	+
Q9NQW7	Xaa-Pro aminopeptidase 1	-	+	+	-	-	+
O14773	Tripeptidyl-peptidase 1	+	+	+	+	+	+
Q96KP4	Cytosolic non-specific dipeptidase	+	+	+	+	+	+
P36776	Lon protease homolog, mitochondrial	-	+	+	+	-	+
Q15661	Tryptase alpha/beta-1	+	+	-	+	-	+
Q9NY33	Dipeptidyl peptidase 3	+	+	+	-	+	+
O75439	Mitochondrial-processing peptidase subunit beta	-	+	-	-	-	+
P55210	Caspase-7	-	-	+	-	-	+
P28838	Cytosol aminopeptidase	+	+	+	+	+	+
P29466	Caspase-1	-	-	-	-	-	+
P61009	Signal peptidase complex subunit 3	-	-	+	+	-	+
P67812	Signal peptidase complex catalytic subunit SEC11A	+	-	+	-	-	+

P29144	Tripeptidyl-peptidase 2	-	-	-	-	-	+
P55786	Puromycin-sensitive aminopeptidase	+	+	+	+	+	+
P09093	Chymotrypsin-like elastase family member 3A	-	-	+	-	-	+
P50281	Matrix metalloproteinase-14	-	-	+	-	-	-

Supplementary Text & Associated Figures

LC-MS Workflow Optimization

Initially, we treated slides with StcE via automatic sprayer, followed by on-slide trypsin digestion, after which we extracted glycopeptides and unmodified peptides using a series of aqueous and organic solvents. After de-salting, we performed a solid phase hydrophilic interaction chromatography (HILIC)-based glycopeptide enrichment using polyhydroxyethyl aspartamide (PHEA) as described in the Materials and Methods. However, search algorithms performed particularly poorly when analyzing enriched glycopeptide data, likely due to the enormous search space needed to accommodate the entire human proteome, non-specific proteolytic cleavage, multiple possible glycosites, and numerous glycan structures. Many of the glycopeptide backbone sequences identified in these searches were incorrect, requiring manual sequencing, a time-consuming approach that is untenable for large datasets.

Thus, we tested extraction of StcE-derived glycopeptides from each tissue prior to trypsin digestion to reduce sample complexity. Following a de-salting step, we acquired glycoproteomic data via LC-MS. When intact N- and O-glycopeptides are fragmented by collision-based techniques, the labile glycosidic bonds are broken and their functional groups are further dissociated from the hexose ring, producing characteristic oxonium ions in the low-mass region. These fragments are collectively referred to as the "HexNAc fingerprint," which is always present in HexNAc-containing glycopeptide MS2 spectra following beam-type collision-induced dissociation.[6] Unfortunately, we observed very weak glycopeptide signal, indicated by the intensity of the HexNAc diagnostic ion at *m/z* 204.0867 across the LC-MS gradient (Supplementary Figs. 33a-c). Searching this data also produced suboptimal results, with the low number of identifications likely stemming from weak MS2 spectra.

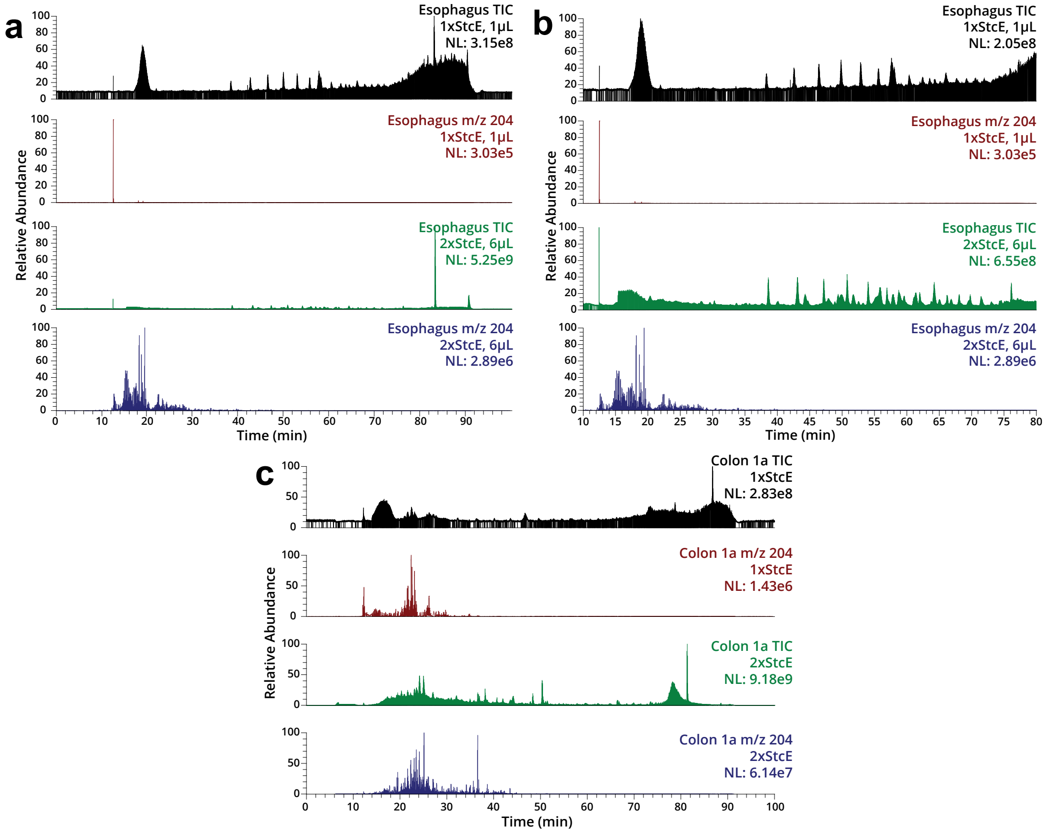

Supplementary Fig. 33. Glycopeptide signal comparison for one or two StcE treatments. (A) Total ion chromatograms (TICs) and traces of HexNAc fingerprint ion at *m/z* 204.0867 for the esophageal tumor digested with StcE once (black, red) or twice (green, blue). (B) Zoom-in on the same TICs and HexNAc traces from 10-80 minutes. (C) TICs and traces of HexNAc fingerprint ion at *m/z* 204.0867 for Colon 1a tumor digested with StcE once (black, red) or twice (green, blue).

We determined that saturating the tissue surface with StcE prior to extraction improved glycopeptide recovery and detection in downstream LC-MS based on the more intense *m/z* 204.0867 trace in these samples. The difference was particularly notable for samples recovered from the mucinous tumor regions (Supplementary Figs. 33a-c and 34a-b). We compared glycopeptide recovery using this “double-StcE” and “single-StcE”-trypsin-PHEA workflows, the latter of which was used in our first experiment. Although the HexNAc traces using each method had similar profiles, ion intensity was stronger in the double-StcE glycopeptide sample (Supplementary Figs. 35a-c). This difference might stem from minor sample loss during PHEA enrichment or from glycopeptide suppression by more readily ionized tryptic peptides during LC-MS acquisition.

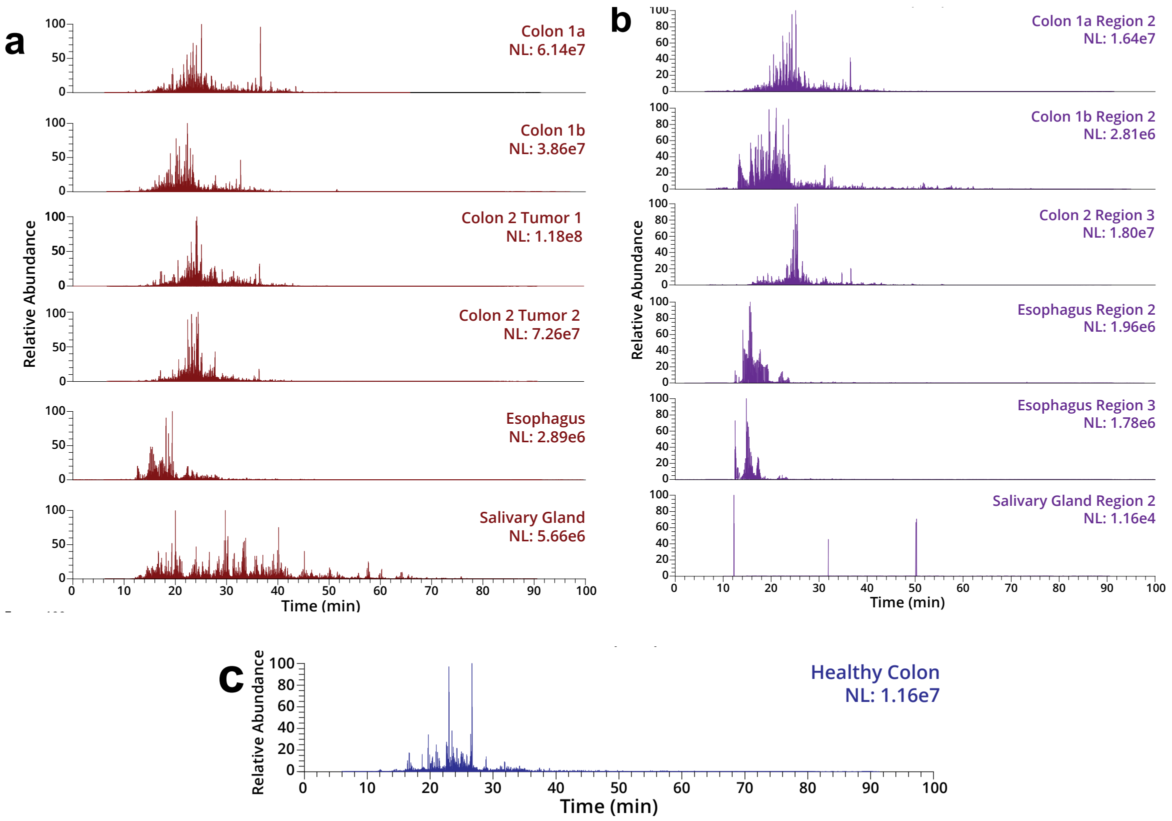

Supplementary Fig. 34. Saturating the tissue surface with StcE improved glycopeptide detection by LC-MS. The double-StcE approach maximized glycopeptide recovery from (A) the mucinous tumors, based on extracted ion chromatograms (XICs) corresponding to the HexNAc fingerprint ion (*m/z* 204.0876). (B) Strong HexNAc signal was also detected in the tumor-adjacent regions for the Colon and Esophageal samples prepared with the double-StcE workflow. (C) Intense HexNAc signal was detected in the healthy colon tissue sample. Regions are numbered according to Figure S5a-f. NL, normalized level.

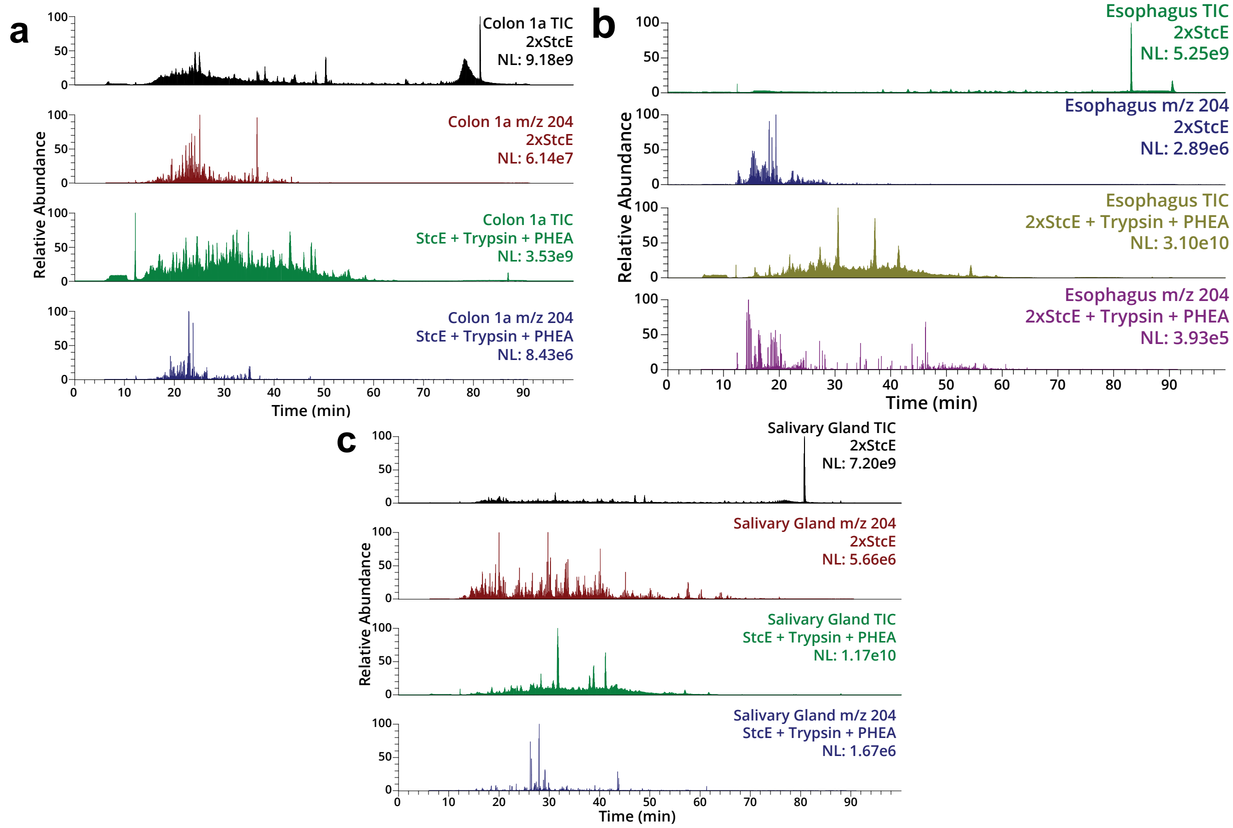

Supplementary Fig. 35. The workflow employing a double-StcE digestion had improved glycopeptide recovery from tumor regions compared to the StcE-trypsin-PHEA method. (A) Total ion chromatograms (TICs) and HexNAc ion traces from Colon 1a tumor region. (B) TICs and HexNAc ion traces from esophageal tumor region. (C) TICs and HexNAc ion traces from salivary gland tumor region.

As a result, we proceeded with the double-StcE digestion, opting to collect mucinous glycopeptides as a separate sample prior to trypsin digestion. We then extracted the unmodified peptides from each tissue and employed the PHEA fractionation workflow, with the goal of improving detection of non-mucinous O-glycopeptides from tryptic digests. However, we observed only negligible HexNAc signal in the tryptic peptide runs. Because StcE specifically targets densely glycosylated mucins, future work should test whether on-slide digestion with a less restricted O-glycoprotease, like the *Pseudomonas aeruginosa* immunomodulating protease (IMPa),[7] enhances O-glycoproteomic coverage of non-mucin glycoproteins. Cleavage proximal to O-glycosites by IMPa and similar enzymes could produce smaller glycopeptides more likely to be ionized in LC-MS, therefore improving their identification. However, it's possible that unmodified tryptic peptides could still suppress their detection during MS acquisition, a potential challenge that may or may not be alleviated via downstream enrichment techniques like the PHEA-based approached described in this work.

Assignment of MUC2 glycoforms in MALDI-MSI using LC-MS identifications

We linked MALDI-MSI heat maps to LC-MS glycopeptide identifications by manually matching ions between raw data files. Because the MALDI and electrospray ionization (ESI) mechanisms tend to produce different charge states of precursor ions,[49] all *m/z* values detected in LC-MS analyses were first converted to *m/z* values likely to be found in MALDI-MSI data, or vice versa. All species identified in our MSI experiments were singly charged and adducted to one or two sodium ions, consistent with the tendency of MALDI to generate singly charged precursor ions.[50,51]

On the other hand, ESI of mucinase-derived O-glycopeptides primarily produced protonated precursor ions that were doubly or triply charged (Supplementary Data 2).

The sensitivity of LC-MS experiments superseded that of MALDI-MSI, as evidenced by the high number of identified O-glycopeptides lacking MALDI heat maps (Supplementary Data 2). This is partially due to the acquisition parameters used in MALDI-MSI experiments, including variations in the scan range. The maximum *m/z* values used in our experiments were 4000 (Colon 1a), 2500 (Colon 1b), and 3000 (Colon 2, Healthy Colon, Esophagus & Salivary Gland). A narrower MS scan range can enhance detection of ions within the *m/z* limits but is also likely to reduce the number of species identified. For example, the singly charged, mono-sodiated precursor masses for 11.3% of the O-glycopeptides identified via LC-MS (71/630) exceeded the upper scan limit of *m/z* 2500 in Colon 1b (Supplementary Data 2). The disparity in MS sensitivity might also be related to possible differences in glycopeptide ionization efficiency for ESI and MALDI sources. Intact glycopeptides can suffer from poor ionization efficiency compared to their unmodified counterparts,[36] an effect that might be more pronounced with MALDI compared to ESI. Further optimization of matrix composition and other components of sample preparation might improve O-glycopeptide ionization and detection by MALDI-MSI.

Supplemental References

(1) Akkoca, A. N.; Yanık, S.; Özdemir, Z. T.; Cihan, F. G.; Sayar, S.; Cincin, T. G.; Çam, A.; Özer, C. TNM and Modified Dukes Staging along with the Demographic Characteristics of Patients with Colorectal Carcinoma. *Int J Clin Exp* **2014**, *7* (9), 2828–2835.

(2) Zhang, Y. Epidemiology of Esophageal Cancer. *WJG* **2013**, *19* (34), 5598. https://doi.org/10.3748/wjg.v19.i34.5598.

(3) Yakirevich, E.; Sabo, E.; Klorin, G.; Alos, L.; Cardesa, A.; Ellis, G. L.; Shumway, B. S.; Gnepp, D. R. Primary Mucin-producing Tumours of the Salivary Glands: A Clinicopathological and Morphometric Study. *Histopathology* **2010**, *57* (3), 395–409. https://doi.org/10.1111/j.1365-2559.2010.03639.x.

(4) Rooper, L. M.; Argyris, P. P.; Thompson, L. D. R.; Gagan, J.; Westra, W. H.; Jordan, R. C.; Koutlas, I. G.; Bishop, J. A. Salivary Mucinous Adenocarcinoma Is a Histologically Diverse Single Entity With Recurrent AKT1 E17K Mutations: Clinicopathologic and Molecular Characterization With Proposal for a Unified Classification. *American Journal of Surgical Pathology* **2021**, *45* (10), 1337–1347. https://doi.org/10.1097/PAS.0000000000001688.

(5) Holmén Larsson, J. M.; Karlsson, H.; Sjövall, H.; Hansson, G. C. A Complex, but Uniform O-Glycosylation of the Human MUC2 Mucin from Colonic Biopsies Analyzed by nanoLC/MSn. *Glycobiology* **2009**, *19* (7), 756–766. https://doi.org/10.1093/glycob/cwp048.

(6) Bagdonaite, I.; Malaker, S. A.; Polasky, D. A.; Riley, N. M.; Schjoldager, K.; Vakhrushev, S. Y.; Halim, A.; Aoki-Kinoshita, K. F.; Nesvizhskii, A. I.; Bertozzi, C. R.; Wandall, H. H.; Parker, B. L.; Thaysen-Andersen, M.; Scott, N. E. Glycoproteomics. *Nat Rev Methods Primers* **2022**, *2* (1), 48. https://doi.org/10.1038/s43586-022-00128-4.

(7) Vainauskas, S.; Guntz, H.; McLeod, E.; McClung, C.; Ruse, C.; Shi, X.; Taron, C. H. A Broad-Specificity *O* -Glycoprotease That Enables Improved Analysis of Glycoproteins and Glycopeptides Containing Intact Complex *O* -Glycans. *Anal. Chem.* **2022**, *94* (2), 1060–1069. https://doi.org/10.1021/acs.analchem.1c04055.
