# Supplementary material for: Glycosite mapping and in situ mass spectrometry imaging of MUC2 glycopeptides via on-slide mucinase digestion
Source: Nat Commun. 2026 May 7;17:6125. doi: 10.1038/s41467-026-72853-3 (PMC13357551; doi:10.1038/s41467-026-72853-3)
Supplement: Supplementary file 2 — Description of Additional Supplementary Files [file 41467_2026_72853_MOESM2_ESM.pdf]

Description of Additional Supplementary Files

File Name: Supplementary Data 1
Description: Unfiltered global proteomics results obtained using trypsin-treated samples. (*.xlsx)

File Name: Supplementary Data 2
Description: Manually verified glycopeptides identified via LC-MS. (*.xlsx)
