# Supplementary material for: Glycosite mapping and in situ mass spectrometry imaging of MUC2 glycopeptides via on-slide mucinase digestion
Source: Nat Commun. 2026 May 7;17:6125. doi: 10.1038/s41467-026-72853-3 (PMC13357551; doi:10.1038/s41467-026-72853-3)
Supplement: Supplementary file 5 — Reporting Summary [file 41467_2026_72853_MOESM5_ESM.pdf]

nature portfolio

Corresponding author(s): Stacy A. Malaker

Last updated by author(s): 3/29/2026

Reporting Summary

Nature Portfolio wishes to improve the reproducibility of the work that we publish. This form provides structure for consistency and transparency in reporting. For further information on Nature Portfolio policies, see our Editorial Policies and the Editorial Policy Checklist.

Statistics

For all statistical analyses, confirm that the following items are present in the figure legend, table legend, main text, or Methods section.

n/a	Confirmed	
☐	☒	The exact sample size (*n*) for each experimental group/condition, given as a discrete number and unit of measurement
☒	☐	A statement on whether measurements were taken from distinct samples or whether the same sample was measured repeatedly
☒	☐	The statistical test(s) used AND whether they are one- or two-sided<br>*Only common tests should be described solely by name; describe more complex techniques in the Methods section.*
☒	☐	A description of all covariates tested
☒	☐	A description of any assumptions or corrections, such as tests of normality and adjustment for multiple comparisons
☒	☐	A full description of the statistical parameters including central tendency (e.g. means) or other basic estimates (e.g. regression coefficient) AND variation (e.g. standard deviation) or associated estimates of uncertainty (e.g. confidence intervals)
☒	☐	For null hypothesis testing, the test statistic (e.g. *F*, *t*, *r*) with confidence intervals, effect sizes, degrees of freedom and *P* value noted<br>*Give P values as exact values whenever suitable.*
☒	☐	For Bayesian analysis, information on the choice of priors and Markov chain Monte Carlo settings
☒	☐	For hierarchical and complex designs, identification of the appropriate level for tests and full reporting of outcomes
☒	☐	Estimates of effect sizes (e.g. Cohen's *d*, Pearson's *r*), indicating how they were calculated

Our web collection on statistics for biologists contains articles on many of the points above.

Software and code

Policy information about availability of computer code

Data collection	LC-MS data was collected on a Thermo Orbitrap Eclipse Tribrid using Xcalibur software (v4.1 or 4.2).<br>MALDI-MSI data was collected on a Bruker timsTOF fleX MALDI-QTOF, or Bruker timsTOF fleX MALDI-2 QTOF, or Bruker SolariX dual-source 7T MALDI-FTICR with SCiLS Lab (Bruker Corporation, versions 2021a, 2021c, 2023c, 2024a, or 2024b). Immunohistochemistry images for Colon 2 were collected with an Aperio AT2 Digital Pathology scanner (Leica Biosystems).
Data analysis	Unmodified proteomic data was analyzed with Byonic (Protein Metrics, version 4.5.2). Glycoproteomic data was also analyzed with Byonic as well as O-Pair with MetaMorpheus (version 1.0.2 or 1.0.3). All reported search results were manually verified using Excel and XCalibur (ThermoFisher Scientific, version 4.1). MALDI-MSI data was analyzed with SCiLS Lab (Bruker Corporation, version 2024b Pro).

For manuscripts utilizing custom algorithms or software that are central to the research but not yet described in published literature, software must be made available to editors and reviewers. We strongly encourage code deposition in a community repository (e.g. GitHub). See the Nature Portfolio guidelines for submitting code & software for further information.

Data

Policy information about availability of data

All manuscripts must include a data availability statement. This statement should provide the following information, where applicable:

- Accession codes, unique identifiers, or web links for publicly available datasets
- A description of any restrictions on data availability
- For clinical datasets or third party data, please ensure that the statement adheres to our policy

The MALDI-MSI and LC-MS proteomics data and search algorithm outputs have been deposited to the ProteomeXchange Consortium via the PRIDE partner repository with the dataset identifier PXD055865. All other data is provided in the Supplementary Data files as indicated in the text. Source data are provided with this paper.

Research involving human participants, their data, or biological material

Policy information about studies with human participants or human data. See also policy information about sex, gender (identity/presentation), and sexual orientation and race, ethnicity and racism.

Reporting on sex and gender	n/a
Reporting on race, ethnicity, or other socially relevant groupings	n/a
Population characteristics	n/a
Recruitment	n/a
Ethics oversight	Tissue samples were obtained from the Medical University of South Carolina's Hollings Cancer Center Biorepository or through Stanford University. No identifiable patient information was provided to the investigators, and the Institutional Review Boards at the Medical University of South Carolina (protocol 17669) or Stanford University (protocol IRB-46646) approved the use of all tissues.

Note that full information on the approval of the study protocol must also be provided in the manuscript.

Field-specific reporting

Please select the one below that is the best fit for your research. If you are not sure, read the appropriate sections before making your selection.

- [x] Life sciences
- [ ] Behavioural & social sciences
- [ ] Ecological, evolutionary & environmental sciences

For a reference copy of the document with all sections, see nature.com/documents/nr-reporting-summary-flat.pdf

Life sciences study design

All studies must disclose on these points even when the disclosure is negative.

Sample size	No sample size calculations were made; the number of samples analyzed was based on availability of archival mucinous tumors.
Data exclusions	No data were excluded from this study
Replication	MS analyses were performed once due to (a) sample availability and (b) the vast amount of data generated in each sample and the onus of manual validation.
Randomization	This is not relevant as the purpose of this proof-of-concept work was to develop a new workflow, not to test biological hypotheses.
Blinding	Blinding was not necessary as this was proof-of-concept work to develop a new workflow, not to test biological hypotheses.

Reporting for specific materials, systems and methods

We require information from authors about some types of materials, experimental systems and methods used in many studies. Here, indicate whether each material, system or method listed is relevant to your study. If you are not sure if a list item applies to your research, read the appropriate section before selecting a response.

Materials & experimental systems

n/a	Involved in the study
☐	☒ Antibodies
☒	☐ Eukaryotic cell lines
☒	☐ Palaeontology and archaeology
☒	☐ Animals and other organisms
☒	☐ Clinical data
☒	☐ Dual use research of concern
☒	☐ Plants

Methods

n/a	Involved in the study
☒	☐ ChIP-seq
☒	☐ Flow cytometry
☒	☐ MRI-based neuroimaging

Antibodies

Antibodies used	Rabbit recombinant anti-MUC1 [clone EP1024Y] (Abcam, cat. no. ab218998); Goat anti-Rabbit HRP (Vector Laboratories, MP-7451-15)
Validation	Both antibodies have been extensively validated for specific target binding by the listed manufacturers.

Plants

Seed stocks	n/a
Novel plant genotypes	n/a
Authentication	n/a
