# Supplementary figures and images for: Glycosite mapping and in situ mass spectrometry imaging of MUC2 glycopeptides via on-slide mucinase digestion (part 2 of 2)
Source: Nat Commun. 2026 May 7;17:6125. doi: 10.1038/s41467-026-72853-3 (PMC13357551; doi:10.1038/s41467-026-72853-3)

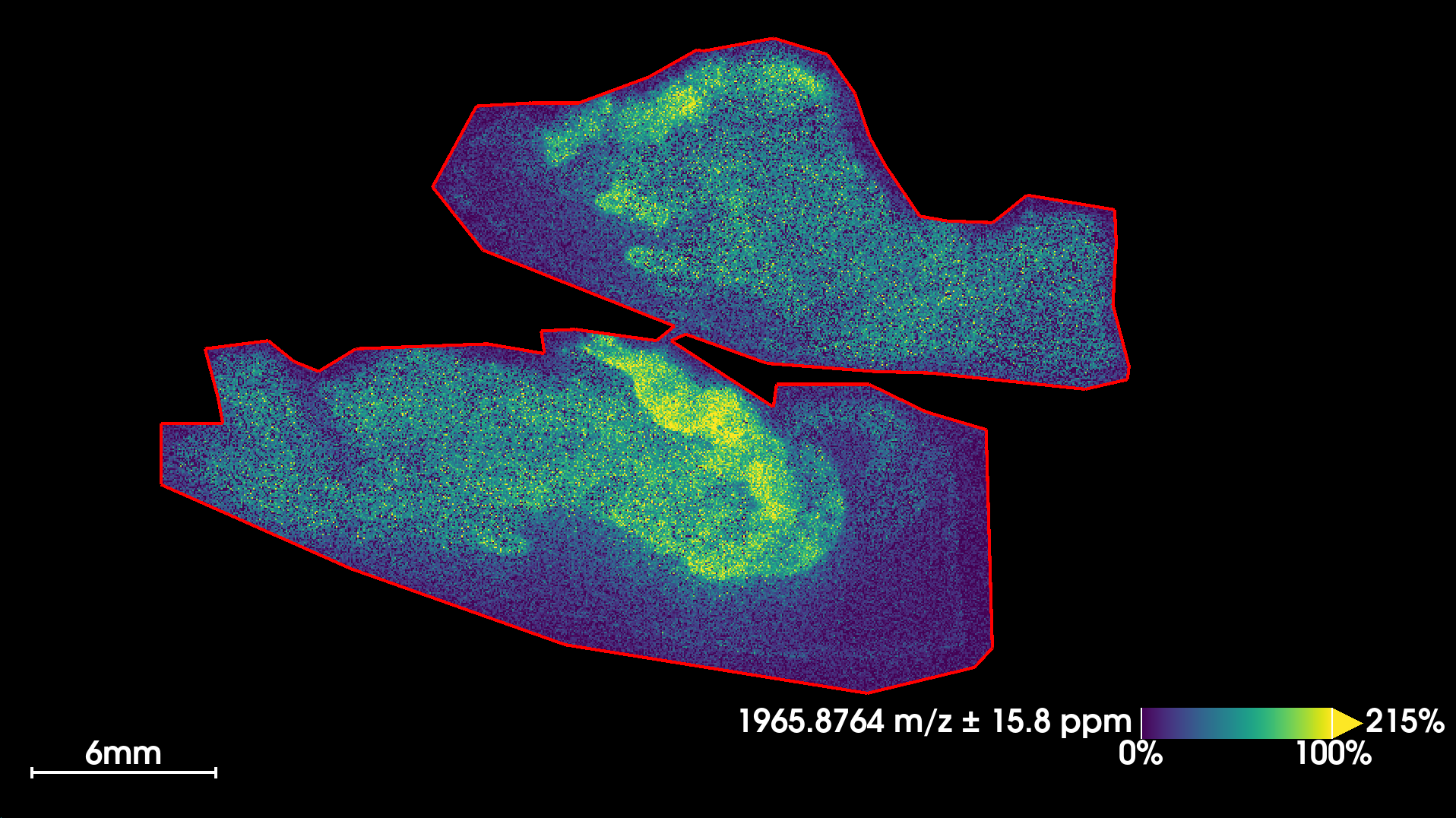

Supplement: Supplementary file 8 — Source Data 2 [file 41467_2026_72853_MOESM8_ESM.zip › Source Data MALDI Images/Supplementary Figure 14/1965.8764 mz ┬▒ 31.1 mDa.png]

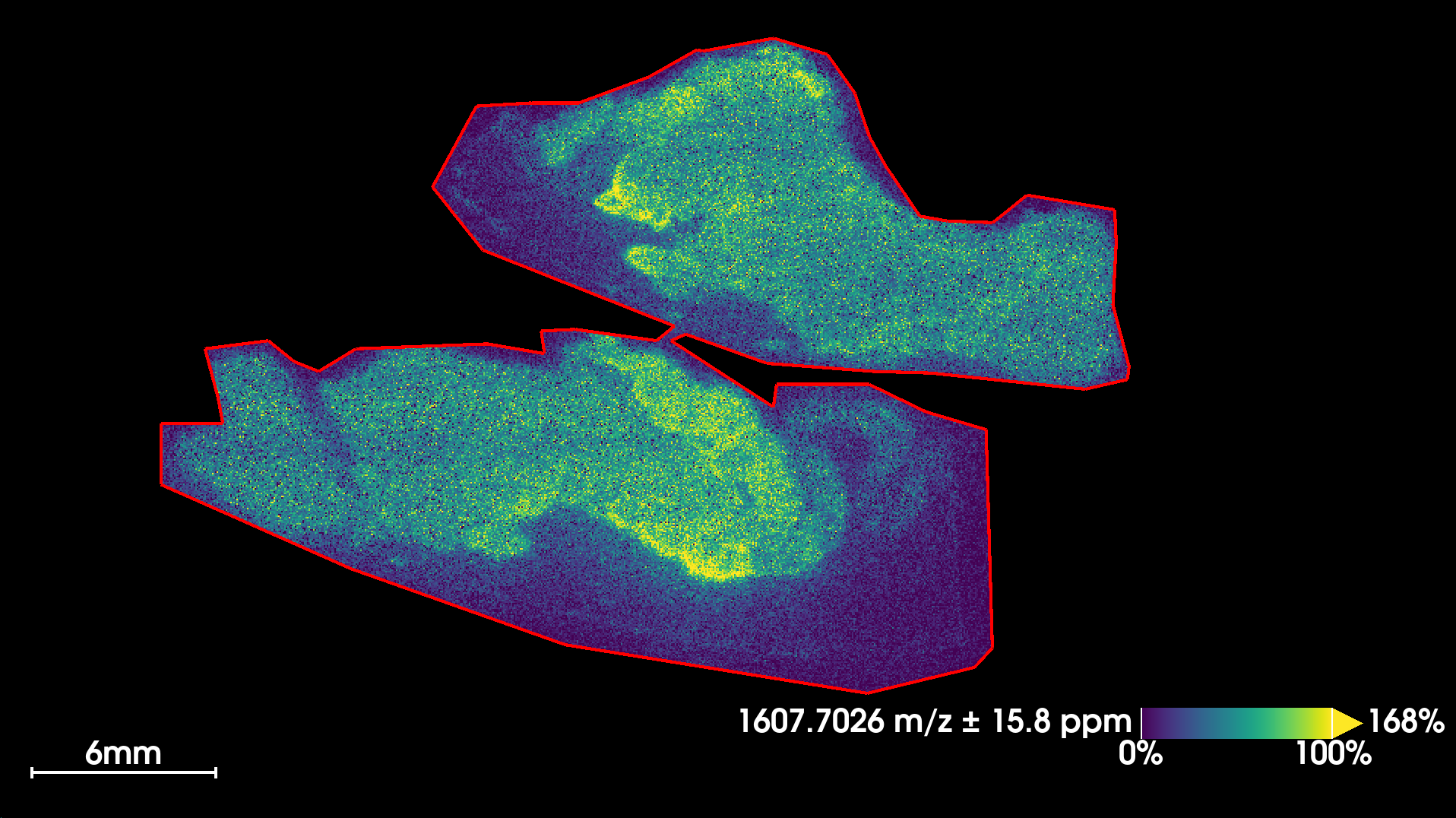

Supplement: Supplementary file 8 — Source Data 2 [file 41467_2026_72853_MOESM8_ESM.zip › Source Data MALDI Images/Supplementary Figure 14/1607.7026 mz ┬▒ 25.5 mDa.png]

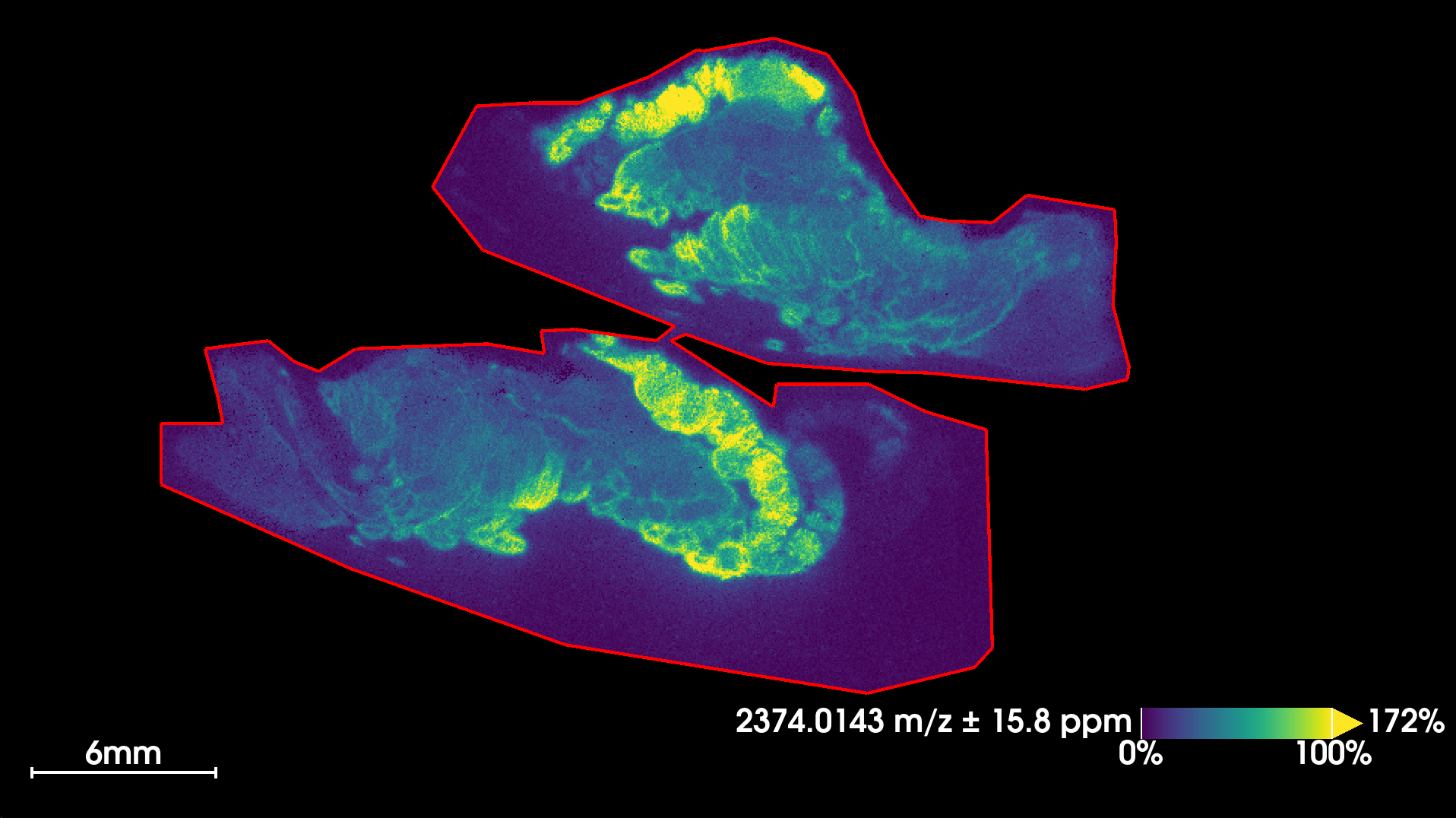

Supplement: Supplementary file 8 — Source Data 2 [file 41467_2026_72853_MOESM8_ESM.zip › Source Data MALDI Images/Supplementary Figure 14/2374.0143 mz ┬▒ 37.6 mDa.png]

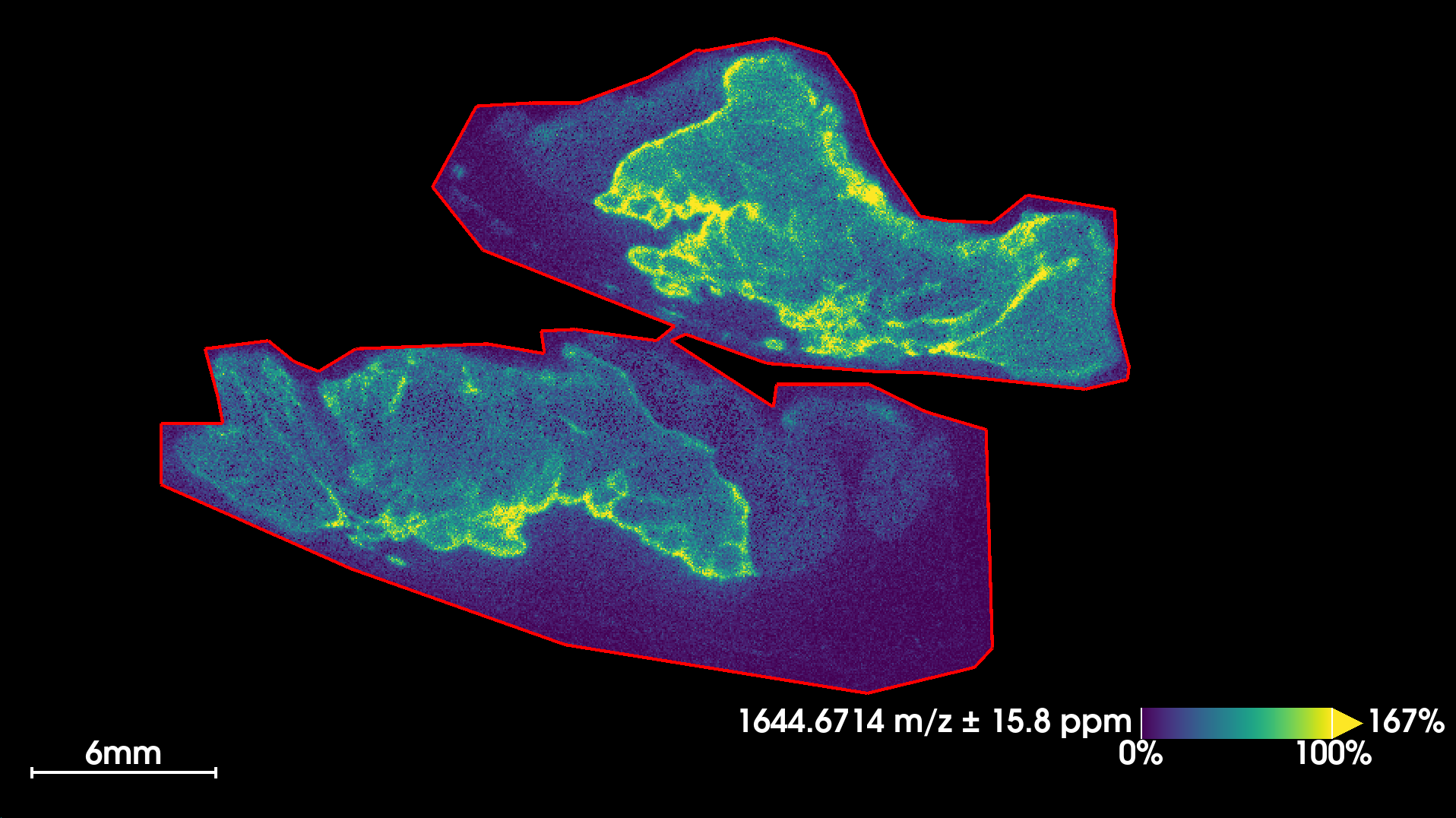

Supplement: Supplementary file 8 — Source Data 2 [file 41467_2026_72853_MOESM8_ESM.zip › Source Data MALDI Images/Supplementary Figure 14/1644.6714 mz ┬▒ 26.1 mDa.png]

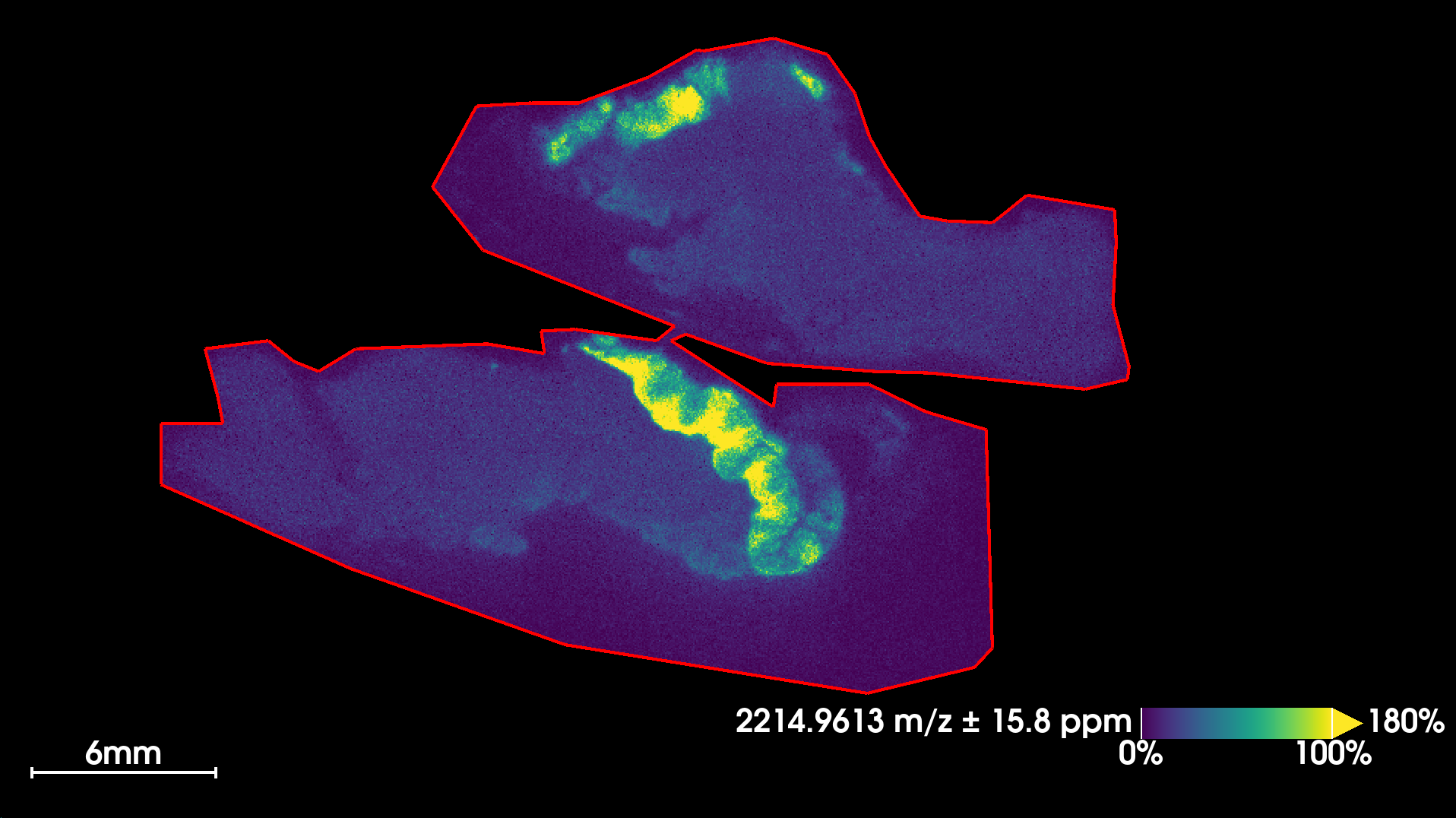

Supplement: Supplementary file 8 — Source Data 2 [file 41467_2026_72853_MOESM8_ESM.zip › Source Data MALDI Images/Supplementary Figure 14/2214.9613 mz ┬▒ 35.1 mDa.png]

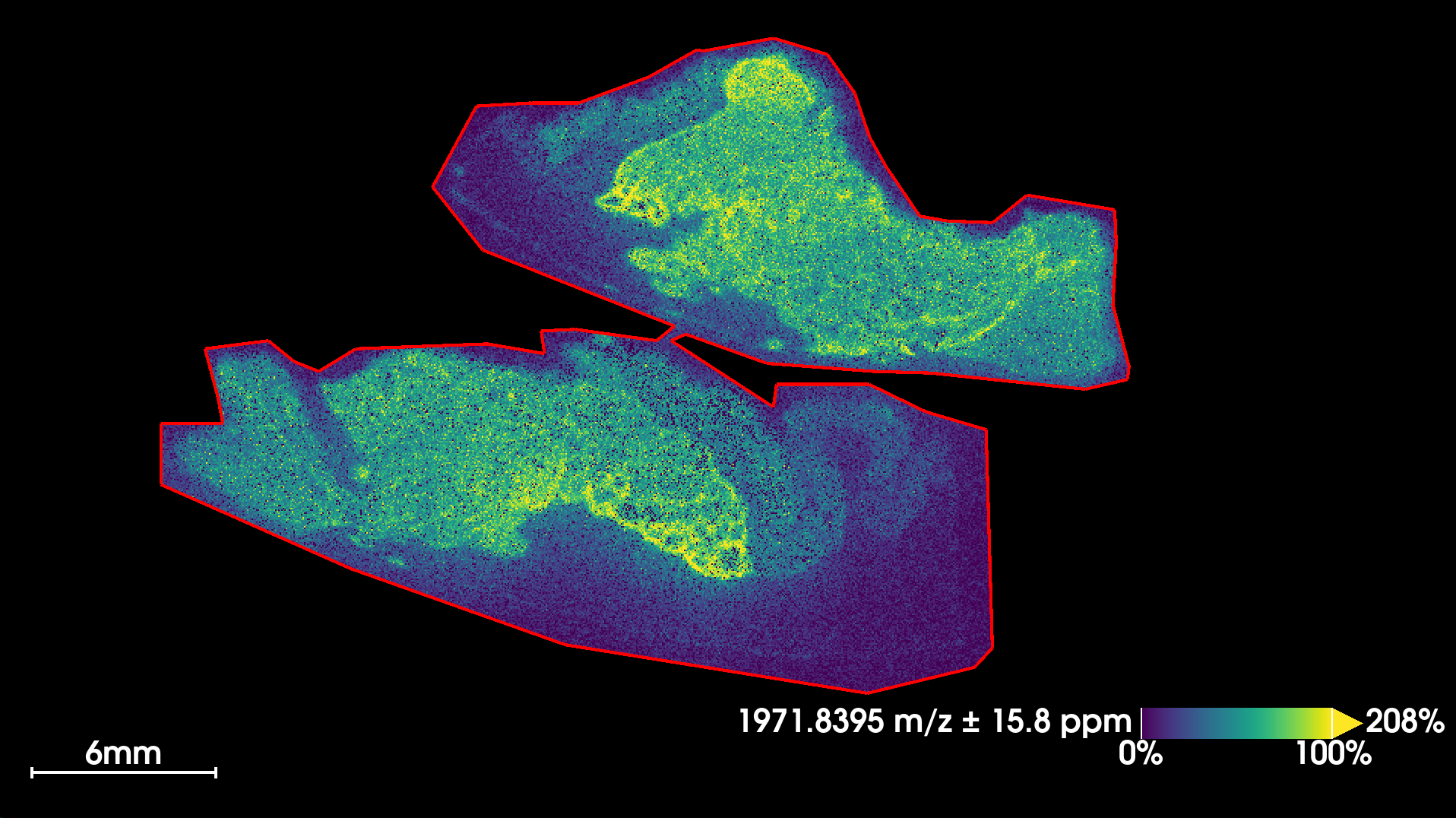

Supplement: Supplementary file 8 — Source Data 2 [file 41467_2026_72853_MOESM8_ESM.zip › Source Data MALDI Images/Supplementary Figure 14/1971.8395 mz ┬▒ 31.2 mDa.png]

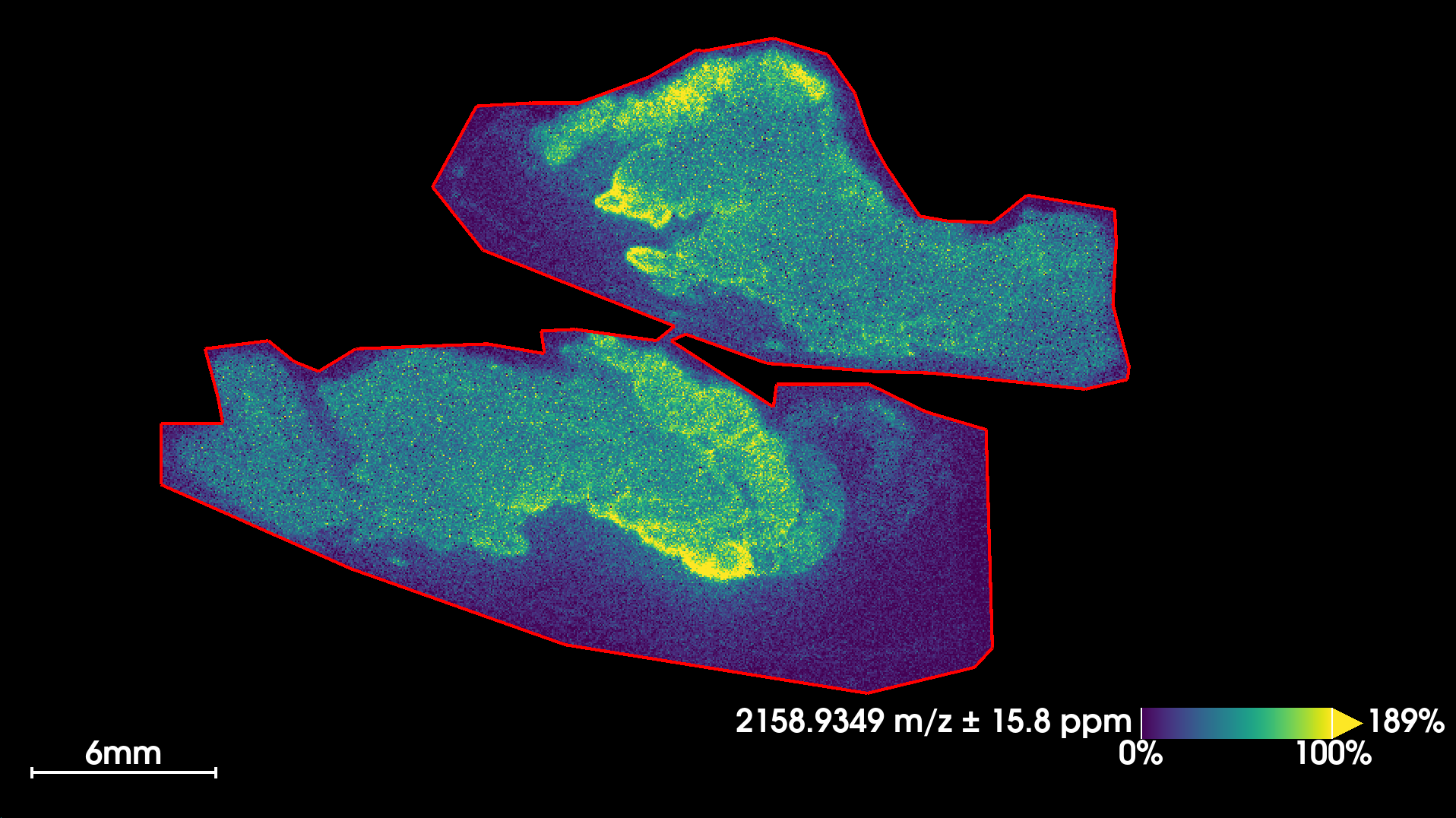

Supplement: Supplementary file 8 — Source Data 2 [file 41467_2026_72853_MOESM8_ESM.zip › Source Data MALDI Images/Supplementary Figure 14/2158.9349 mz ┬▒ 34.2 mDa.png]

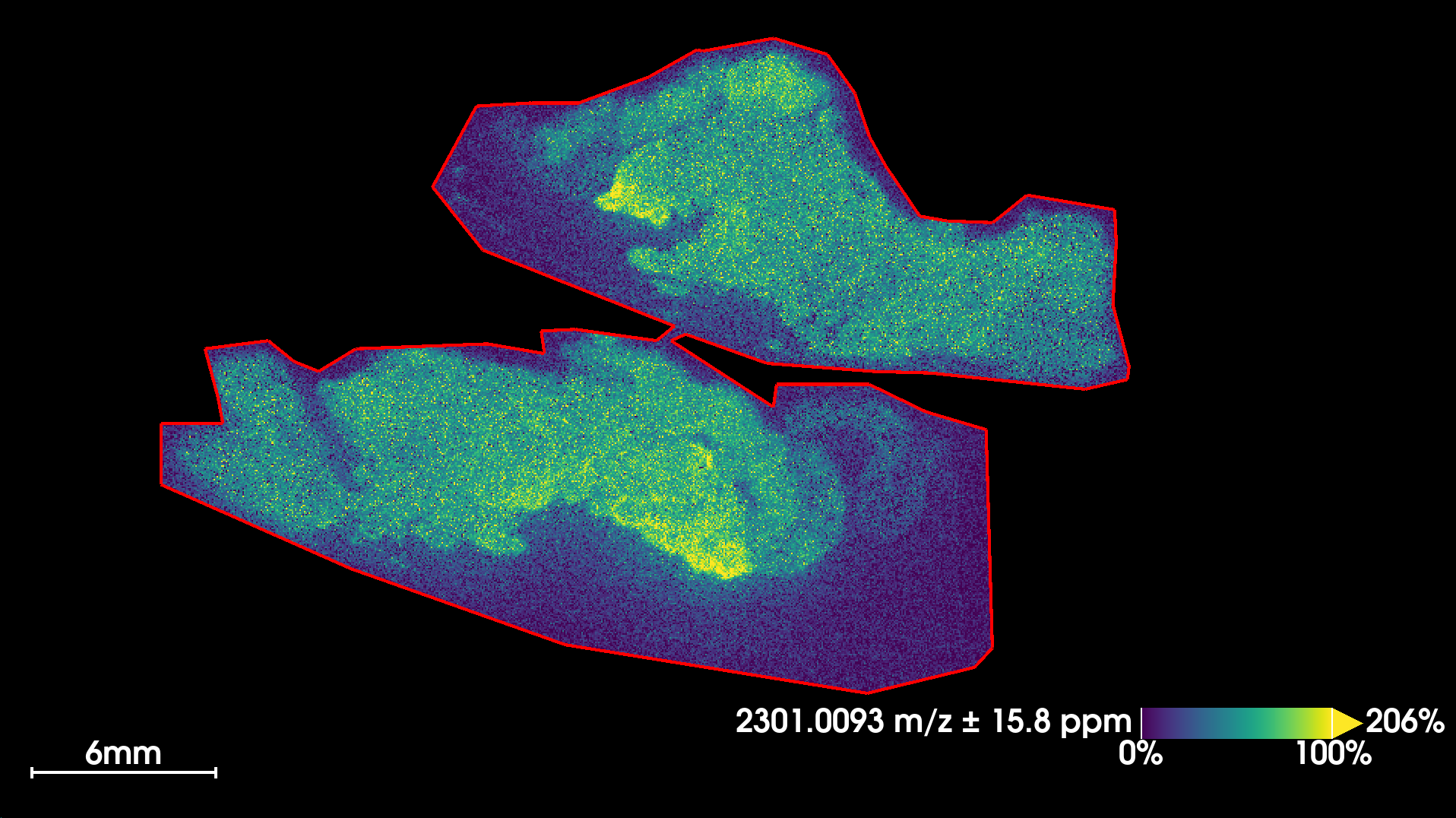

Supplement: Supplementary file 8 — Source Data 2 [file 41467_2026_72853_MOESM8_ESM.zip › Source Data MALDI Images/Supplementary Figure 14/2301.0093 mz ┬▒ 36.5 mDa.png]

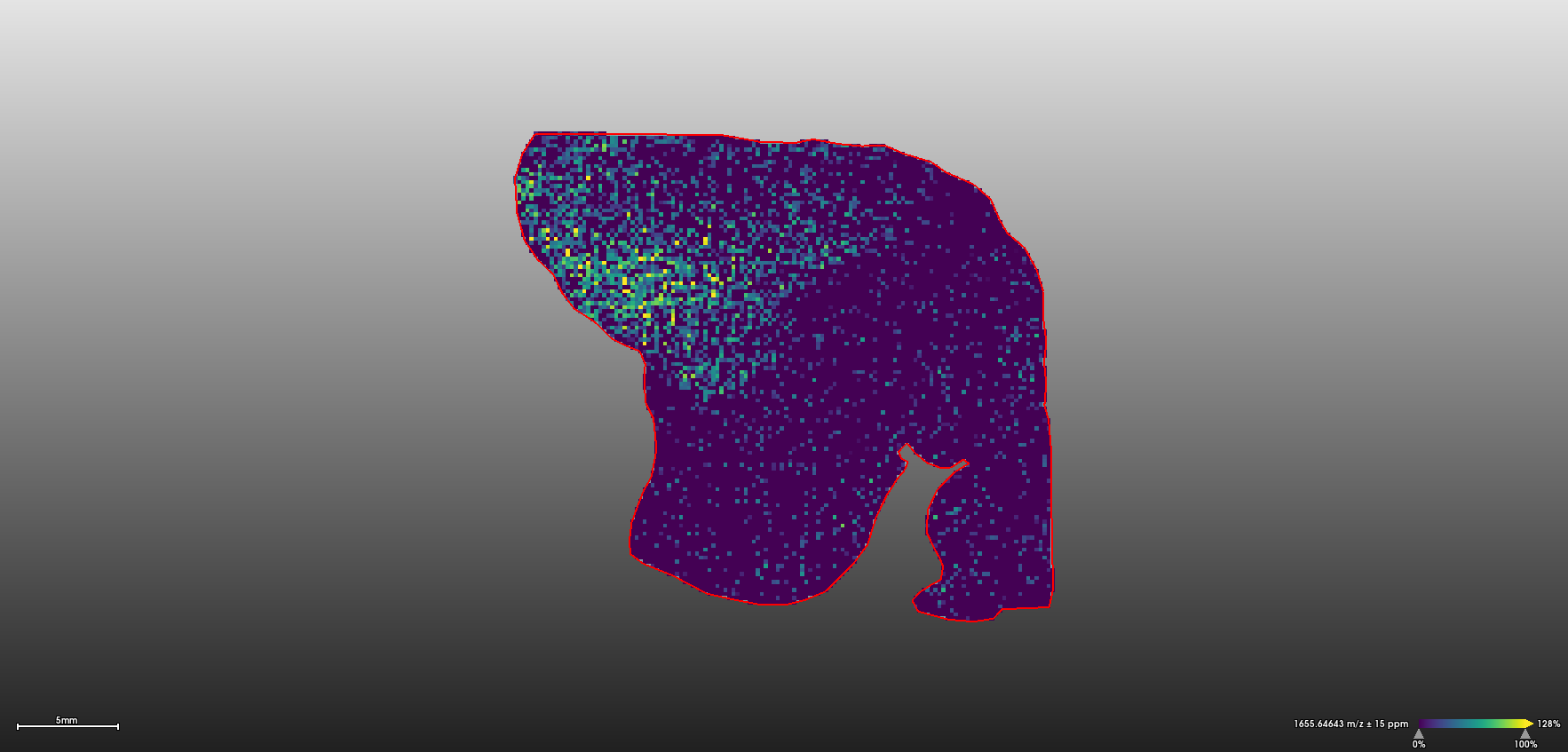

Supplement: Supplementary file 8 — Source Data 2 [file 41467_2026_72853_MOESM8_ESM.zip › Source Data MALDI Images/Supplementary Figure 13/20240531_TPSPPTT+H2N3_Colon1b.png]

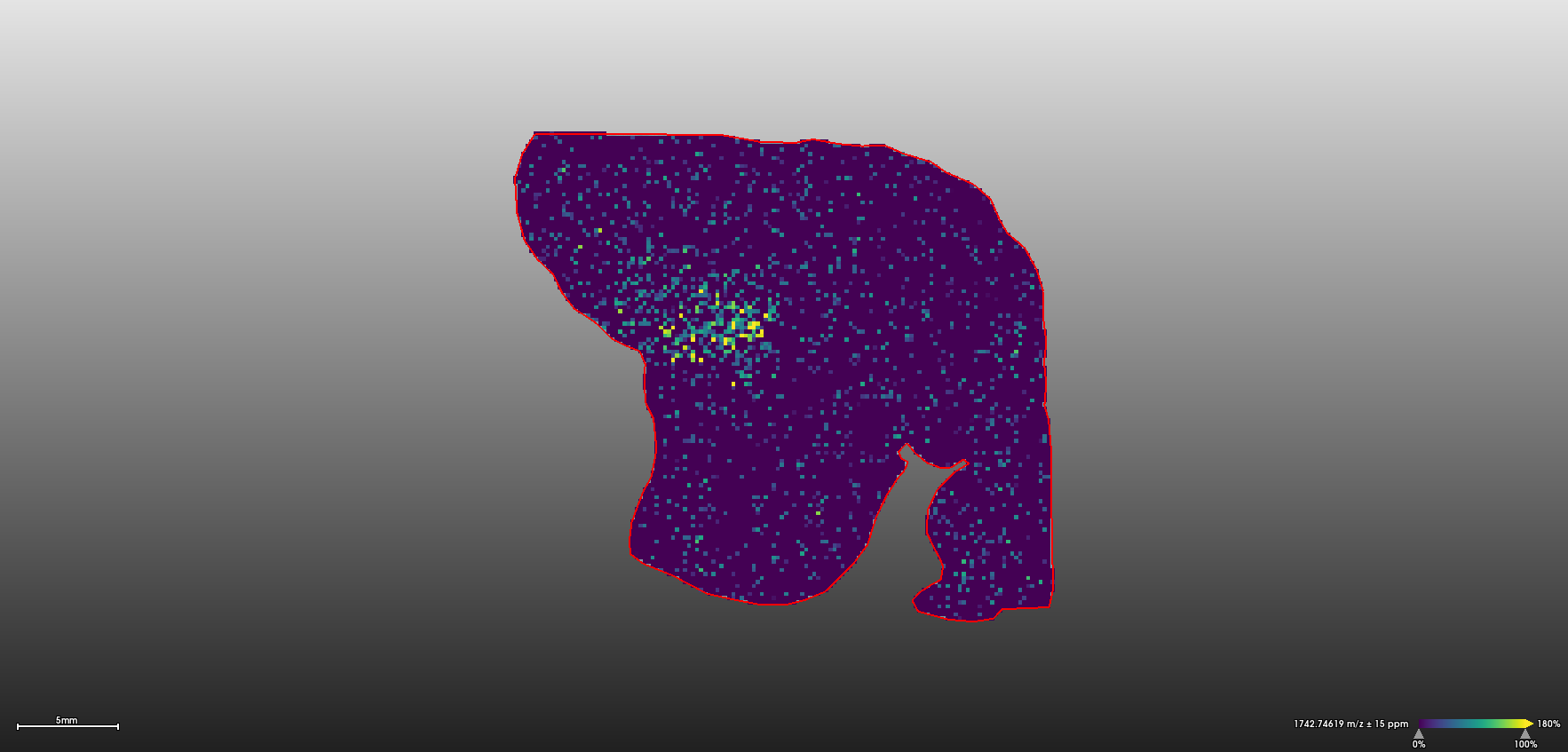

Supplement: Supplementary file 8 — Source Data 2 [file 41467_2026_72853_MOESM8_ESM.zip › Source Data MALDI Images/Supplementary Figure 13/20240627_TLTPITT+H1N4_Colon1b.png]

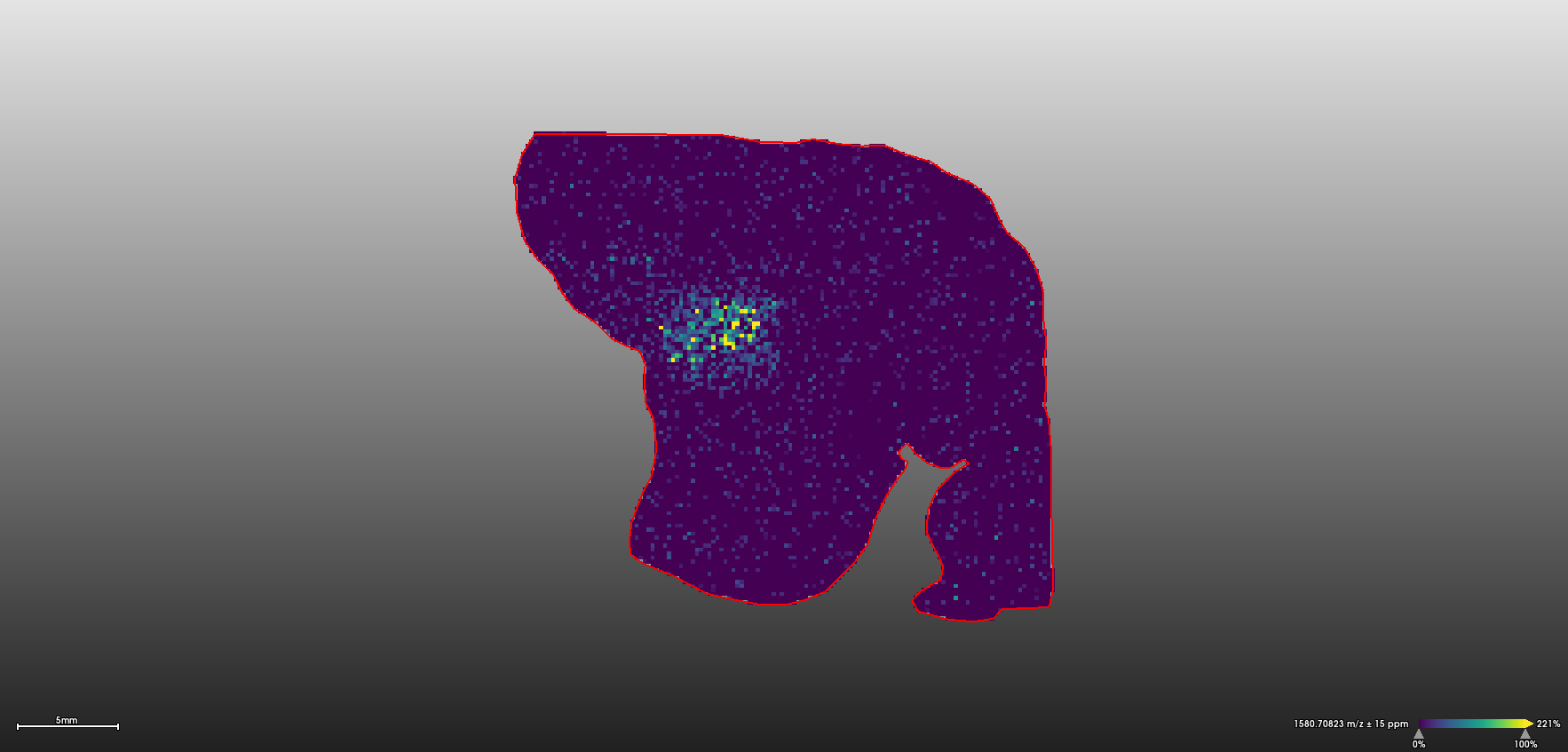

Supplement: Supplementary file 8 — Source Data 2 [file 41467_2026_72853_MOESM8_ESM.zip › Source Data MALDI Images/Supplementary Figure 13/20240627_TLTPITT+N4_Colon1b.png]

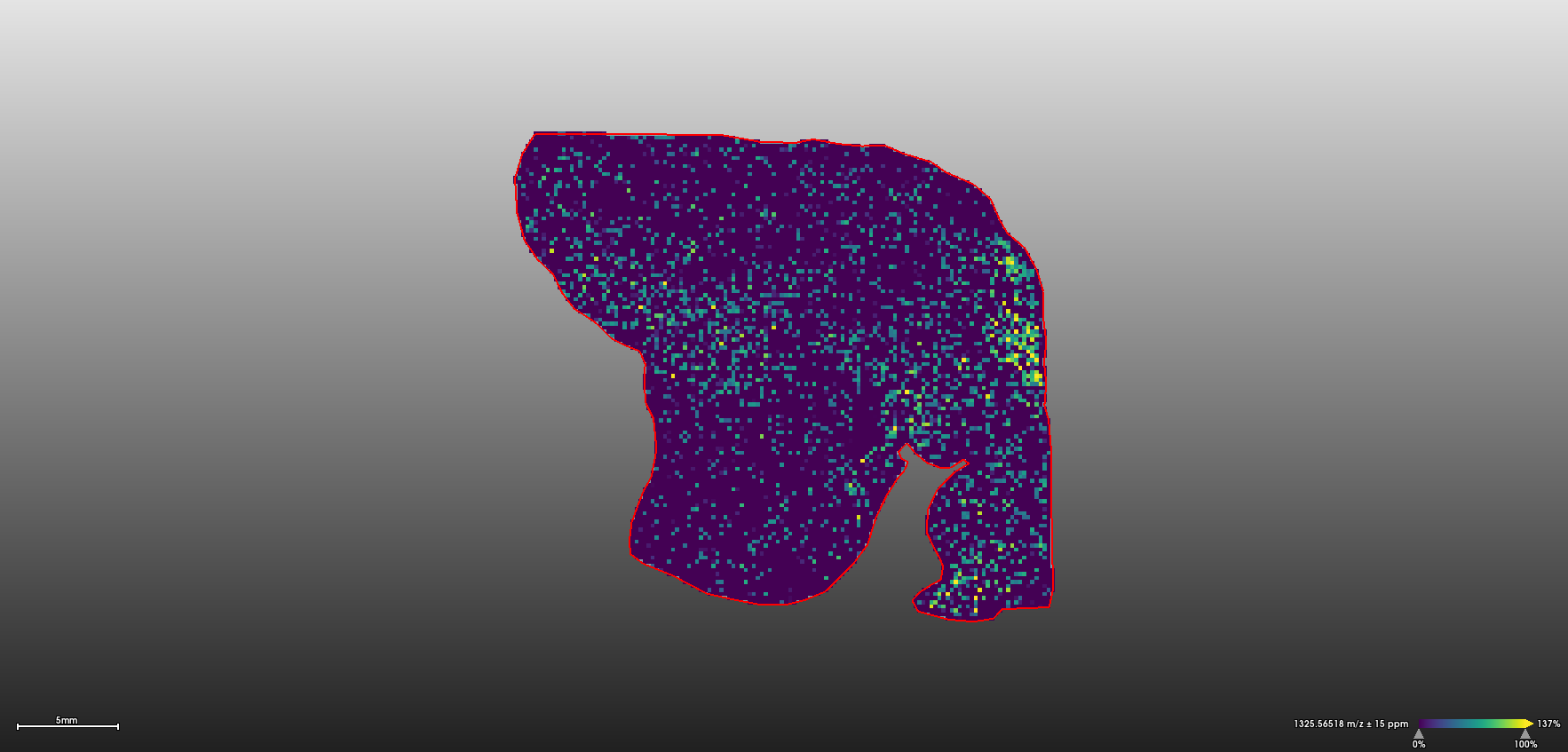

Supplement: Supplementary file 8 — Source Data 2 [file 41467_2026_72853_MOESM8_ESM.zip › Source Data MALDI Images/Supplementary Figure 13/20240627_TPITT+H1N3_Colon1b.png]

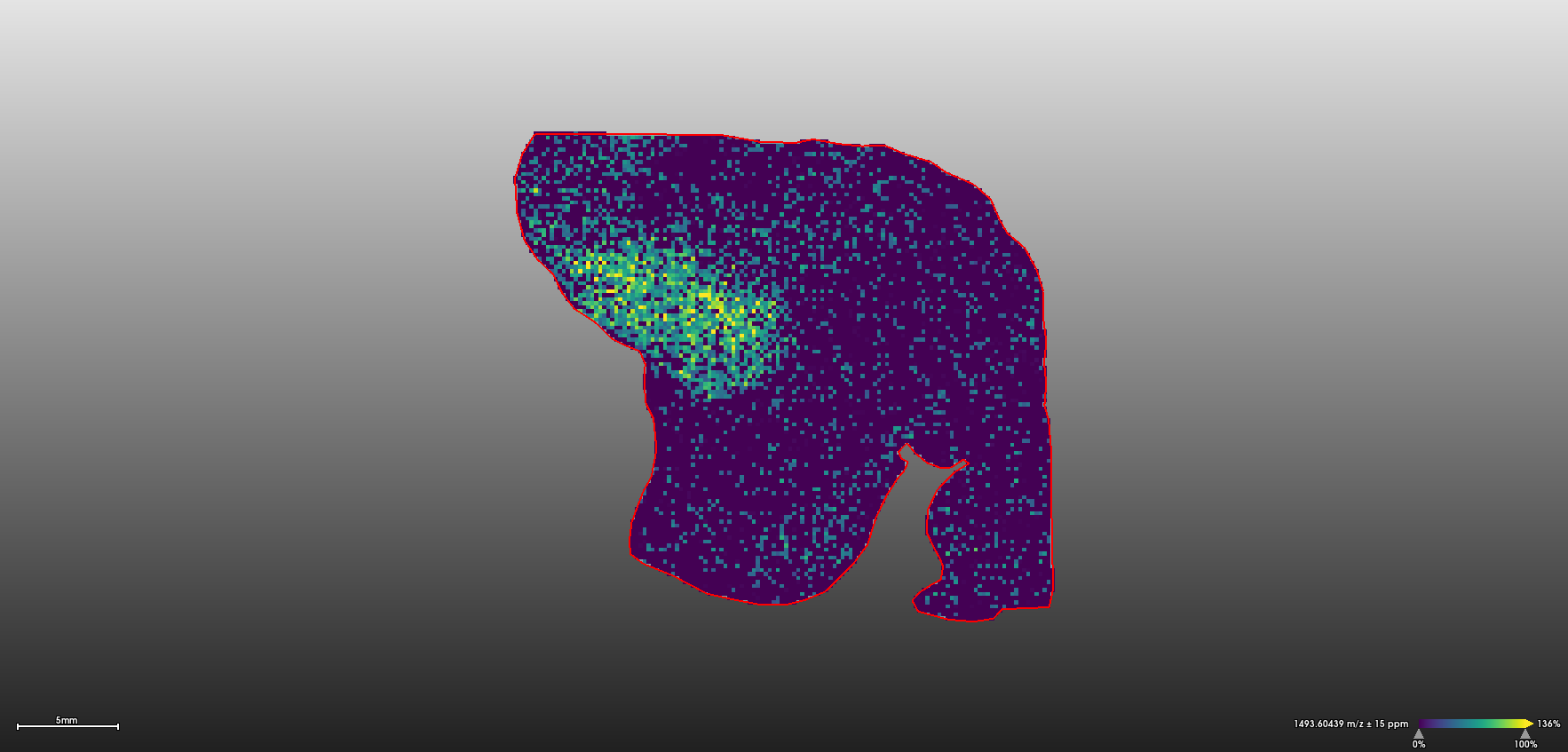

Supplement: Supplementary file 8 — Source Data 2 [file 41467_2026_72853_MOESM8_ESM.zip › Source Data MALDI Images/Supplementary Figure 13/20240531_TPSPPTT+H1N3_Colon1b.png]

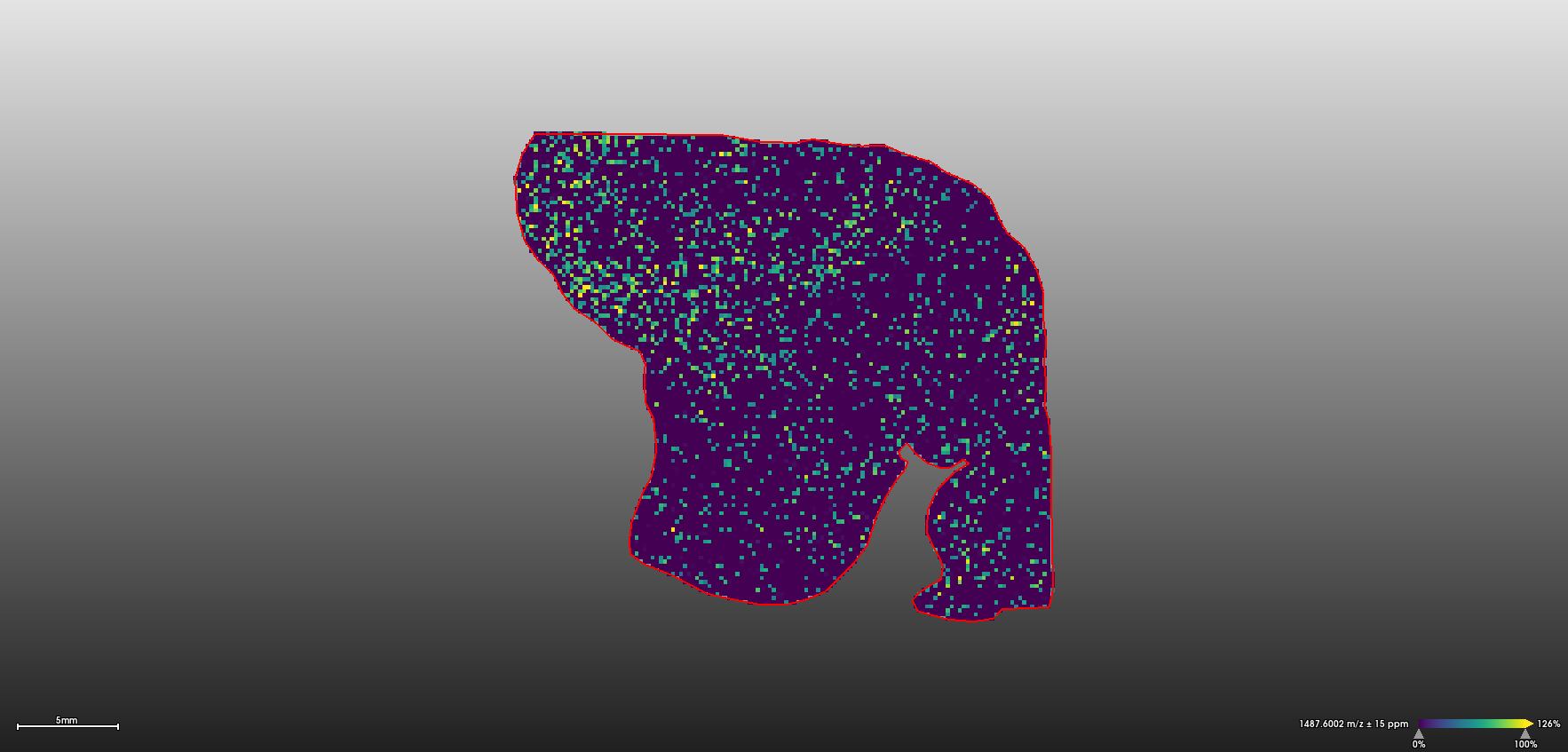

Supplement: Supplementary file 8 — Source Data 2 [file 41467_2026_72853_MOESM8_ESM.zip › Source Data MALDI Images/Supplementary Figure 13/20240627_TPITT+H2N3_Colon1b.png]

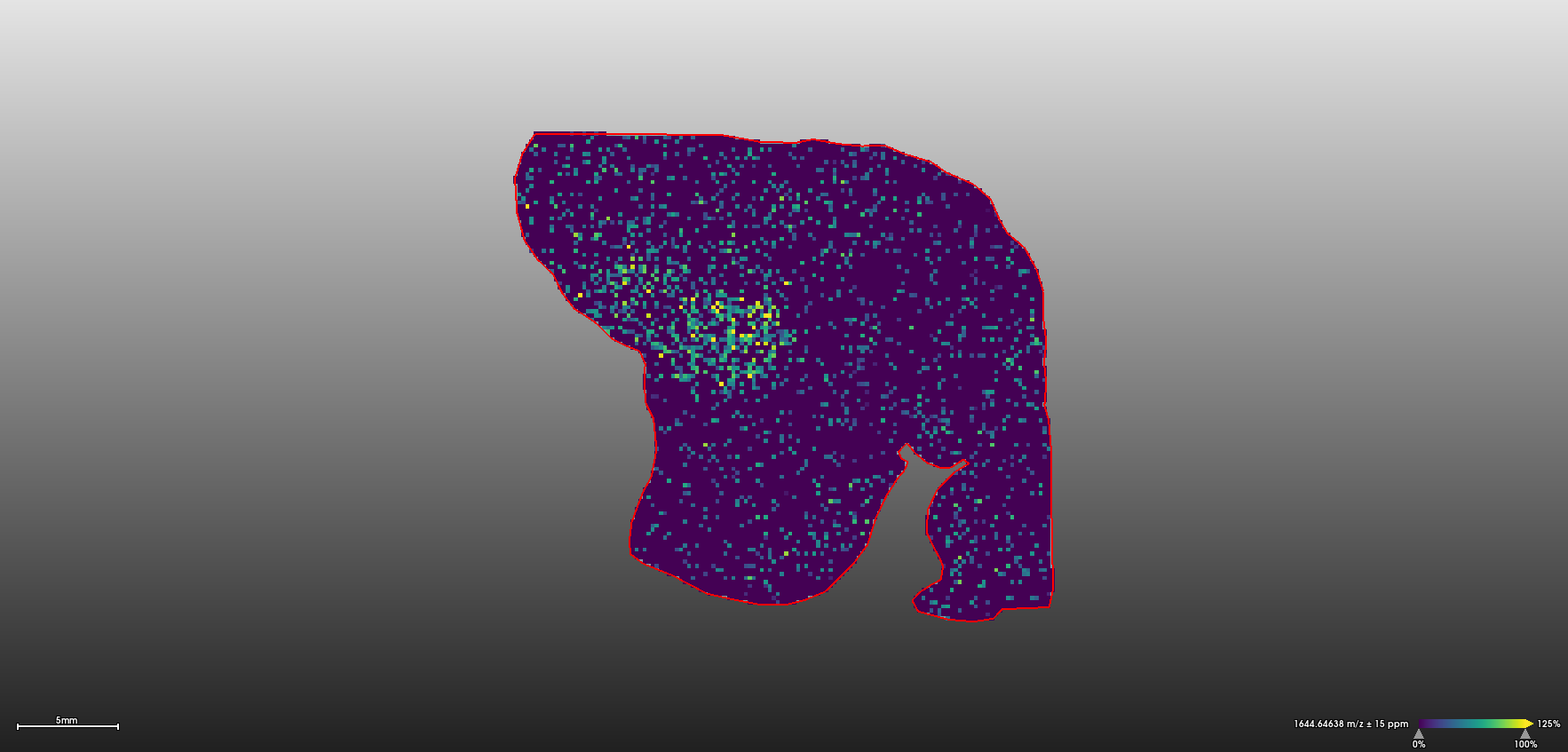

Supplement: Supplementary file 8 — Source Data 2 [file 41467_2026_72853_MOESM8_ESM.zip › Source Data MALDI Images/Supplementary Figure 13/20240531_TQTPTT+H1N4_Colon1b.png]

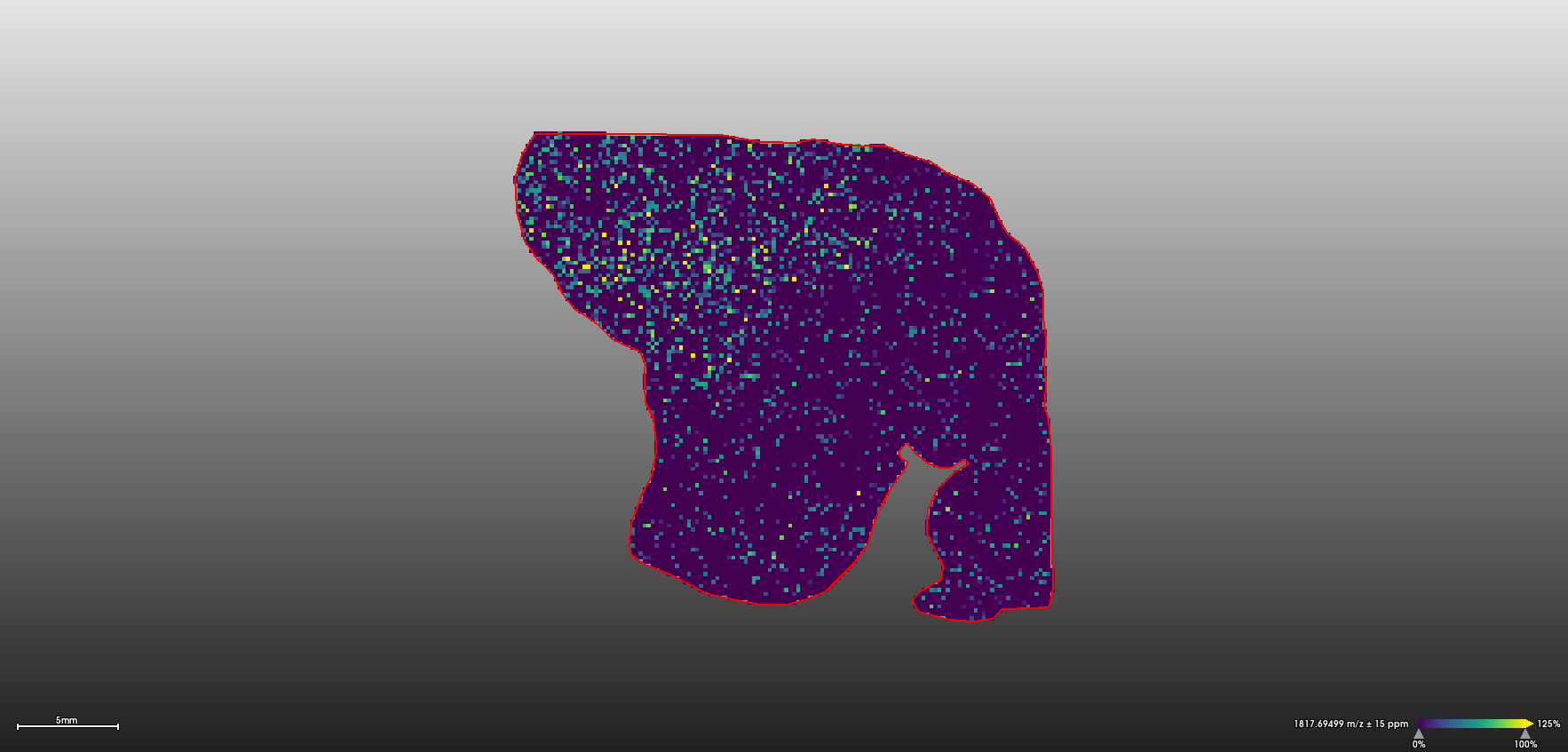

Supplement: Supplementary file 8 — Source Data 2 [file 41467_2026_72853_MOESM8_ESM.zip › Source Data MALDI Images/Supplementary Figure 13/20240627_TPSPPTT+H3N3_Colon1b.png]

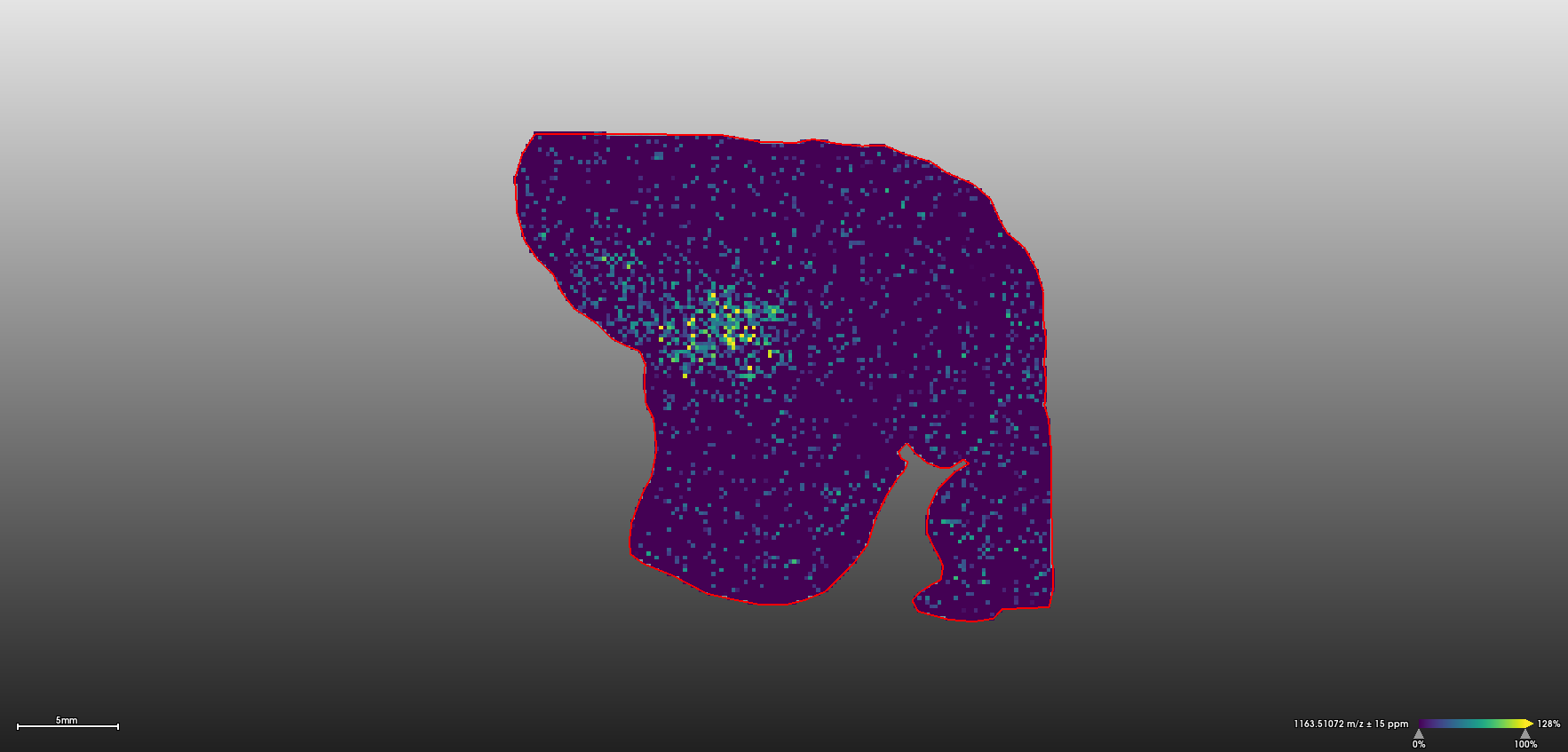

Supplement: Supplementary file 8 — Source Data 2 [file 41467_2026_72853_MOESM8_ESM.zip › Source Data MALDI Images/Supplementary Figure 13/20240627_TPITT+N3_Colon1b.png]

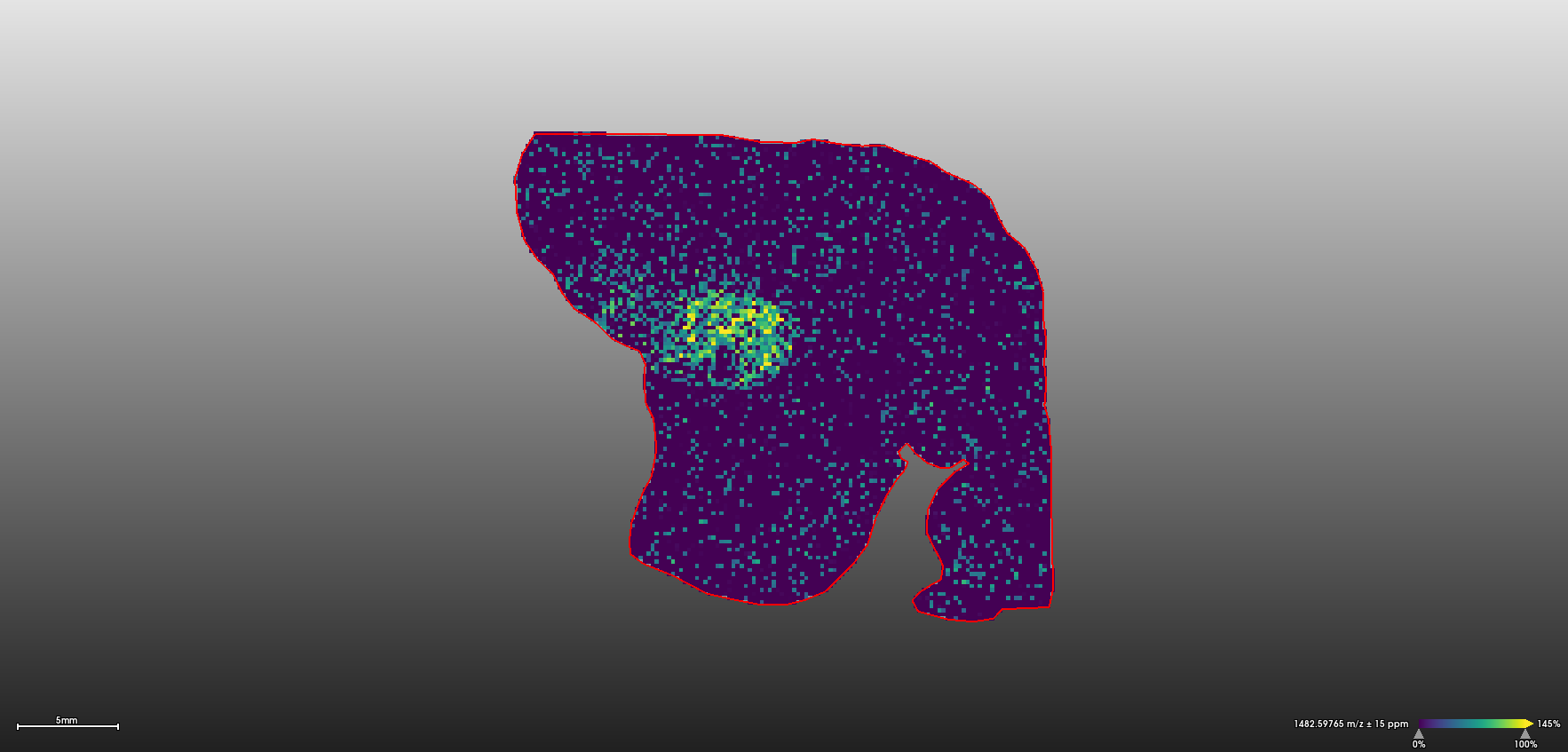

Supplement: Supplementary file 8 — Source Data 2 [file 41467_2026_72853_MOESM8_ESM.zip › Source Data MALDI Images/Supplementary Figure 13/20240531_TQTPTT+N4_Colon1b.png]

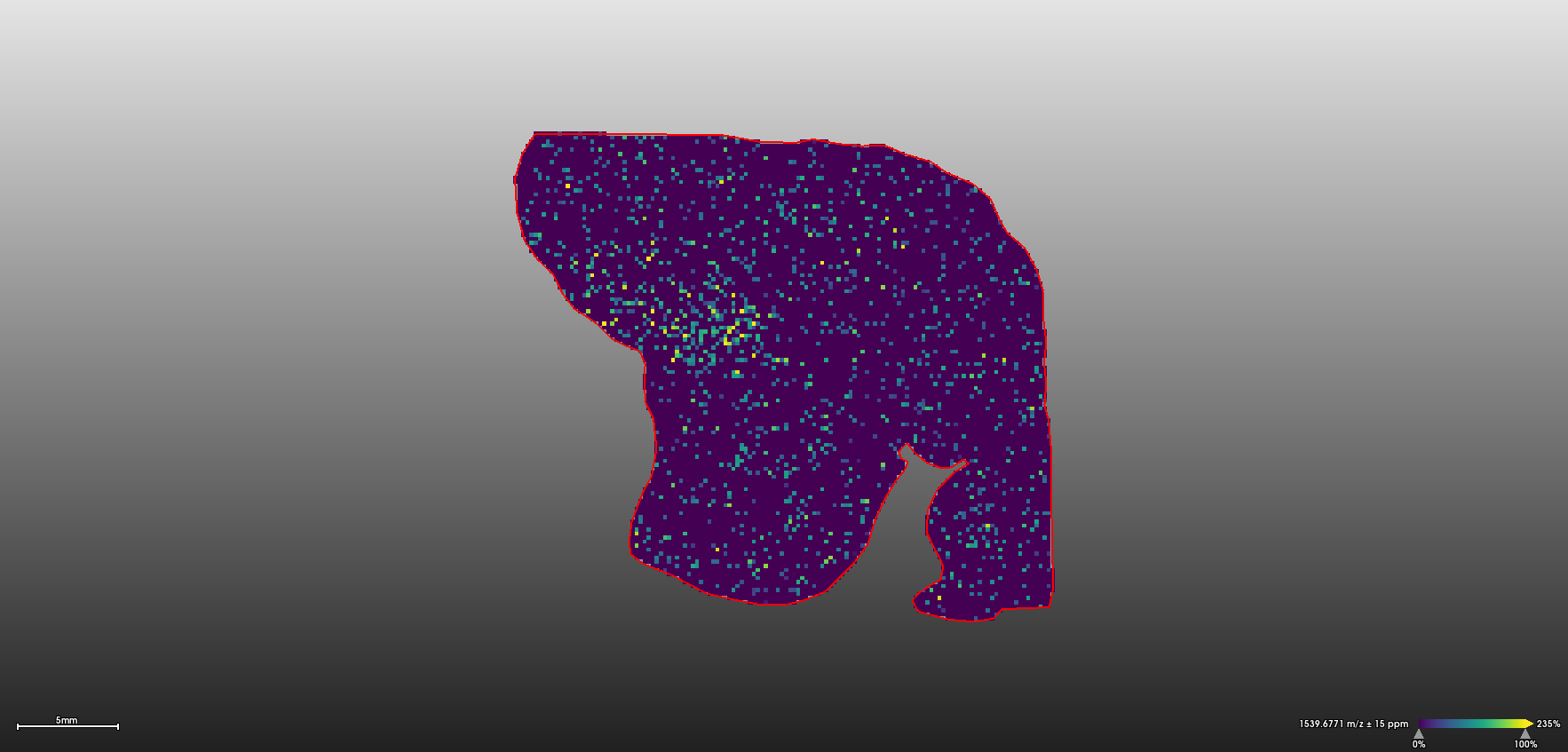

Supplement: Supplementary file 8 — Source Data 2 [file 41467_2026_72853_MOESM8_ESM.zip › Source Data MALDI Images/Supplementary Figure 13/20240627_TLTPITT+H1N3_Colon1b.png]

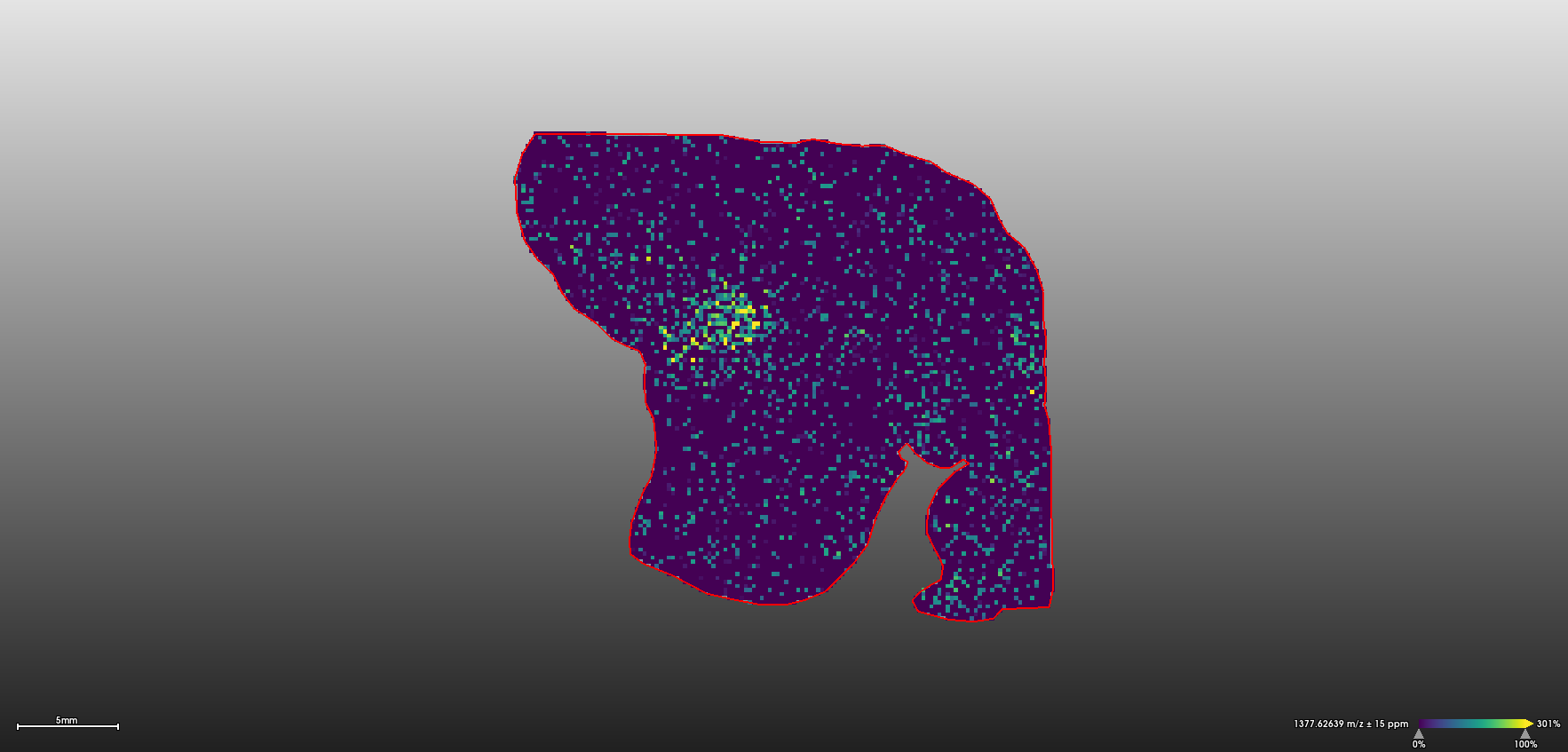

Supplement: Supplementary file 8 — Source Data 2 [file 41467_2026_72853_MOESM8_ESM.zip › Source Data MALDI Images/Supplementary Figure 13/20240627_TLTPITT+N3_Colon1b.png]

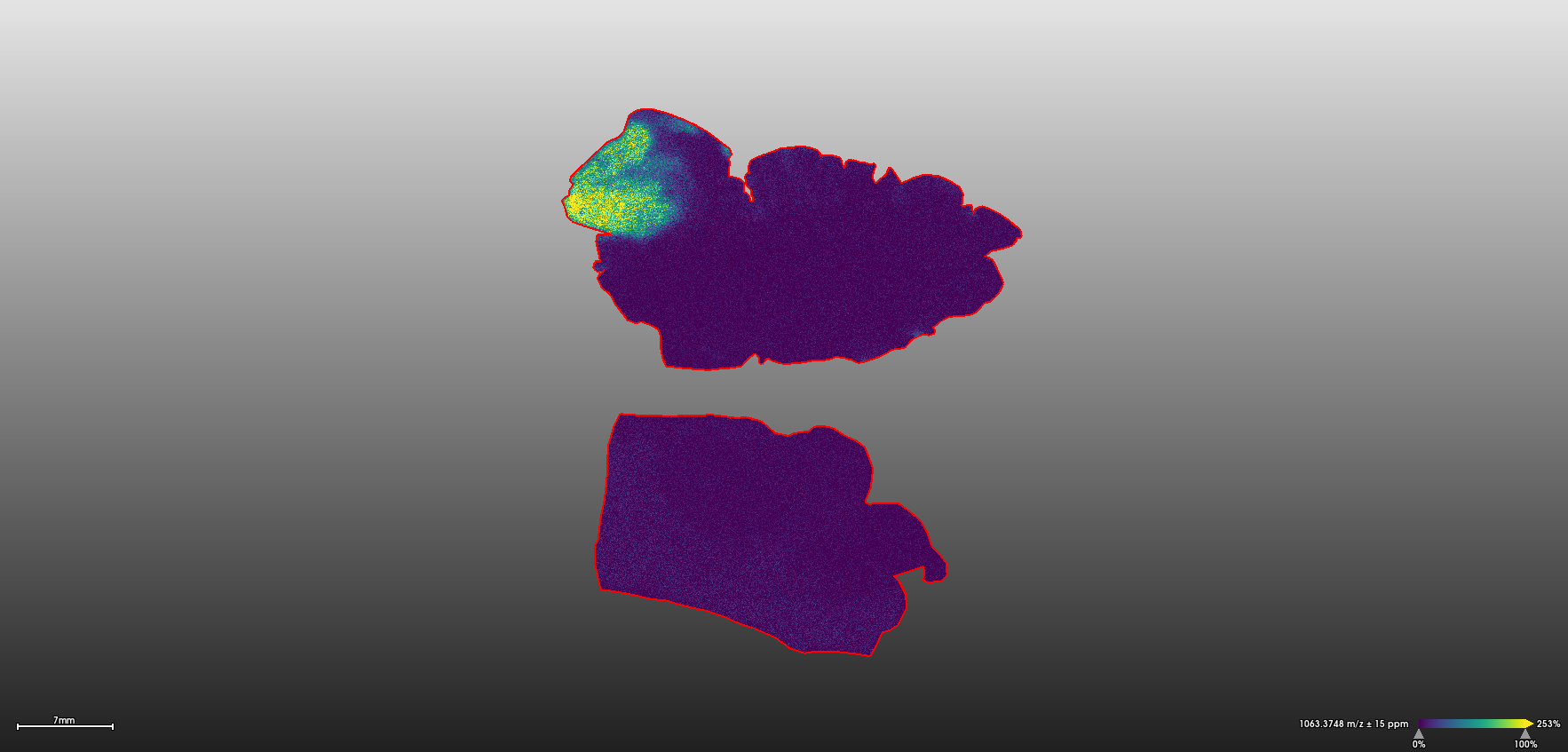

Supplement: Supplementary file 8 — Source Data 2 [file 41467_2026_72853_MOESM8_ESM.zip › Source Data MALDI Images/Figure 1/20240531_mz1063_Eso_Image.png]

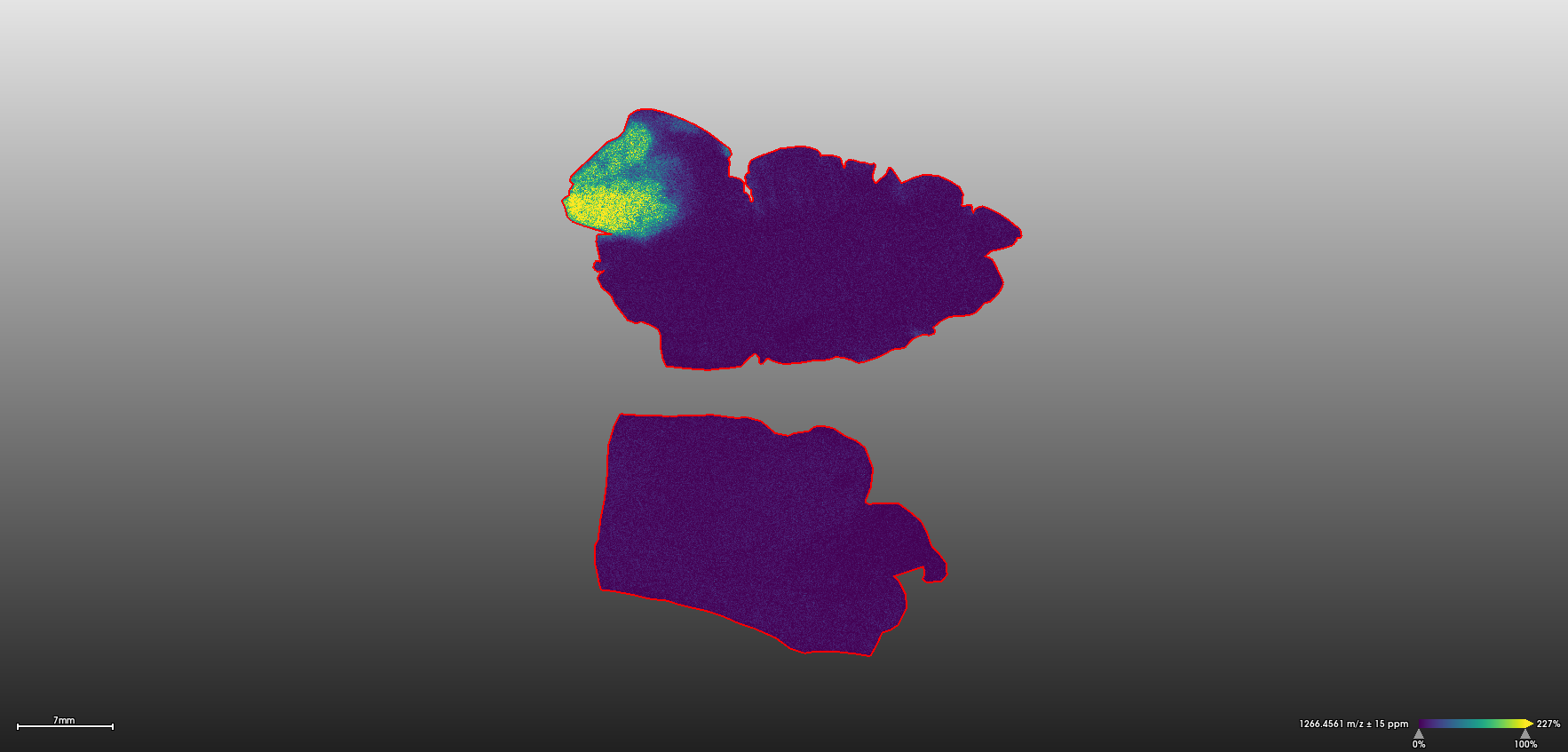

Supplement: Supplementary file 8 — Source Data 2 [file 41467_2026_72853_MOESM8_ESM.zip › Source Data MALDI Images/Figure 1/20240614_mz1266_Eso_Image.png]

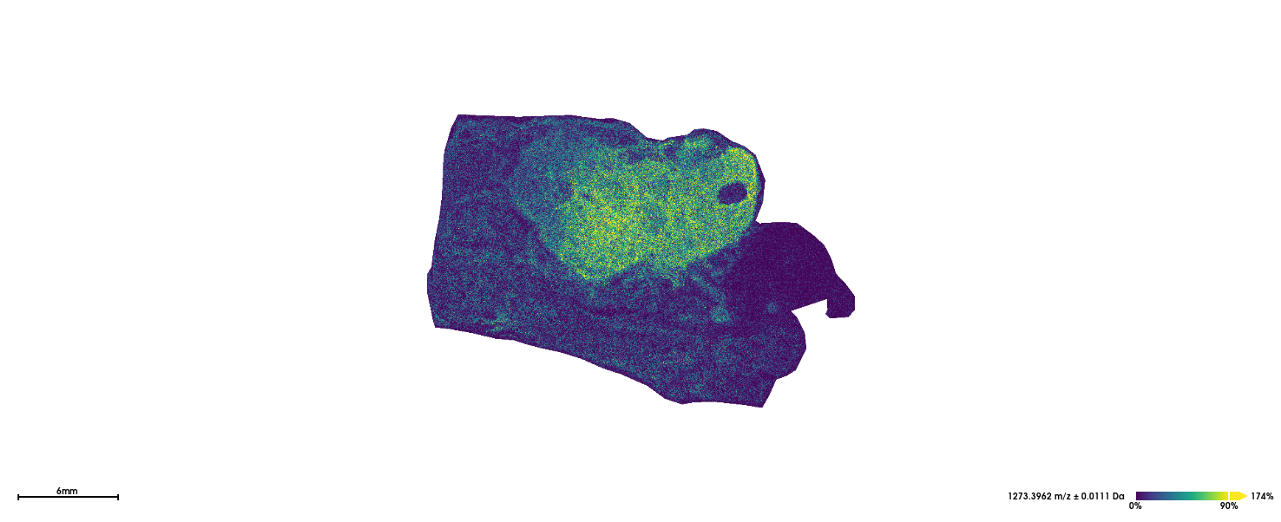

Supplement: Supplementary file 8 — Source Data 2 [file 41467_2026_72853_MOESM8_ESM.zip › Source Data MALDI Images/Figure 1/20260124_mz1273_SG_Image.png]

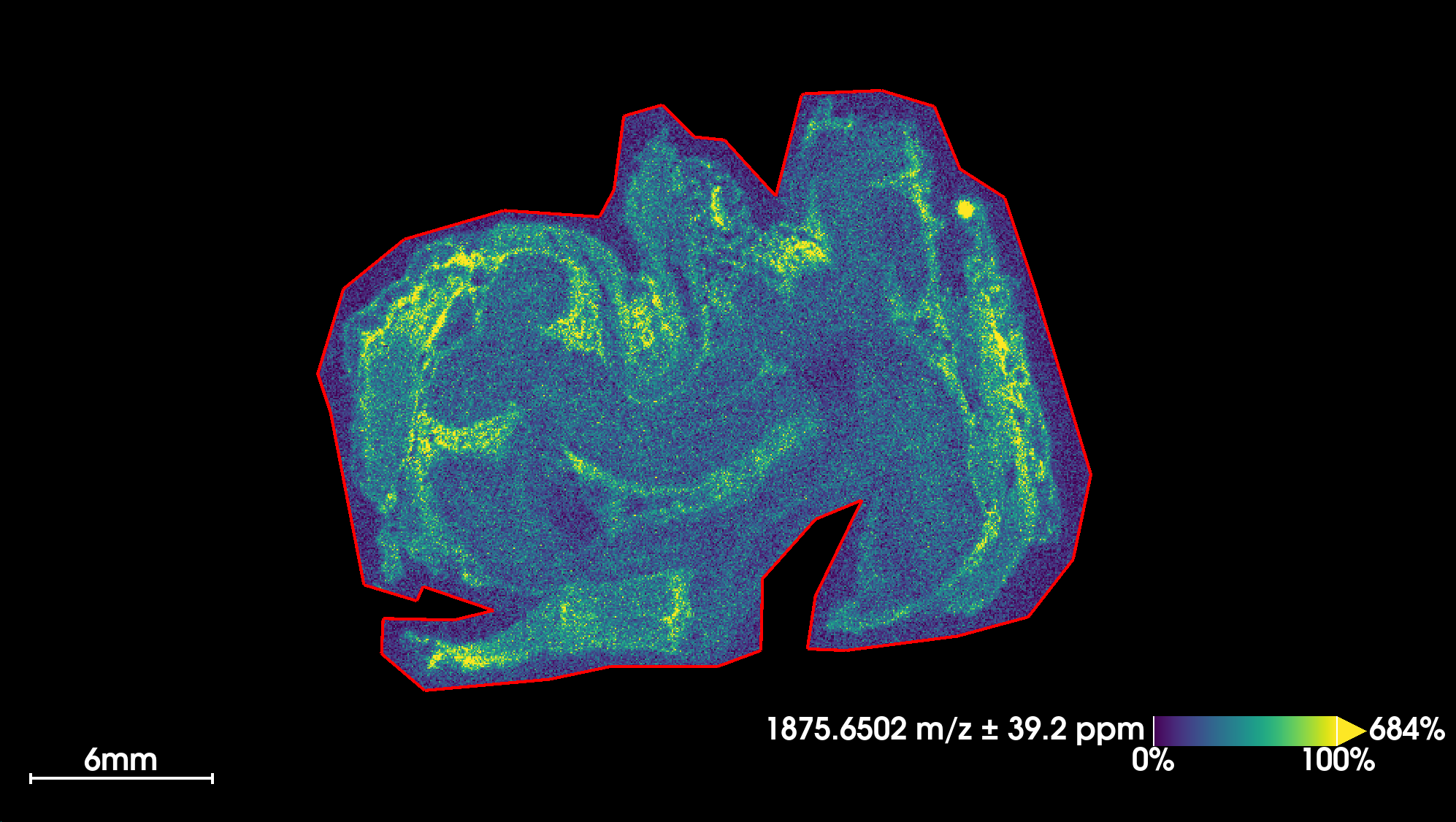

Supplement: Supplementary file 8 — Source Data 2 [file 41467_2026_72853_MOESM8_ESM.zip › Source Data MALDI Images/Figure 1/20250822_mz1875_HC_Image.png]

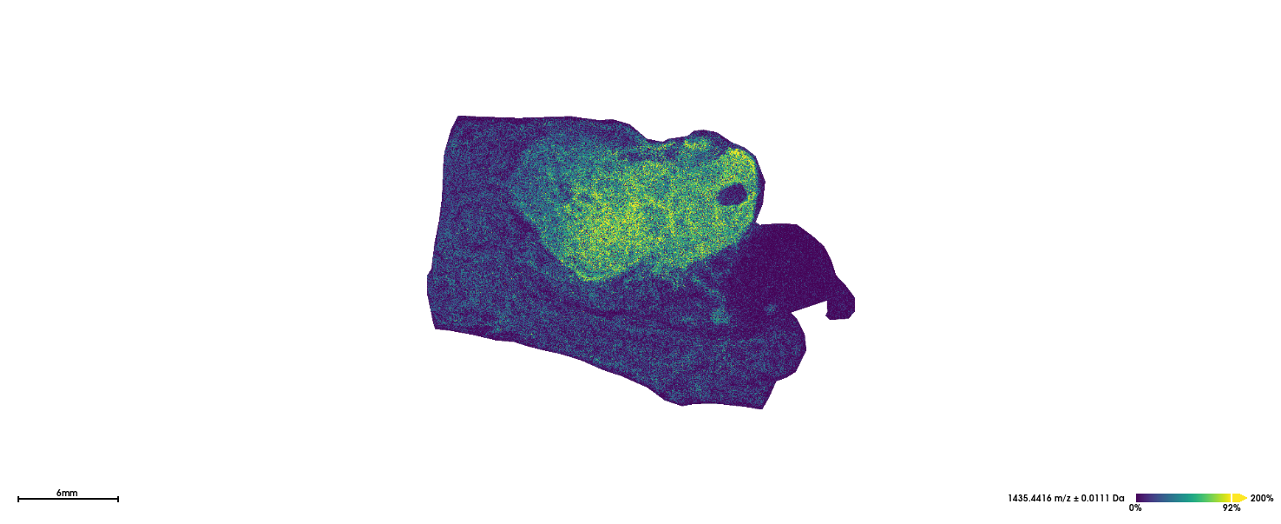

Supplement: Supplementary file 8 — Source Data 2 [file 41467_2026_72853_MOESM8_ESM.zip › Source Data MALDI Images/Figure 1/20260124_mz1435_SG_Image.png]

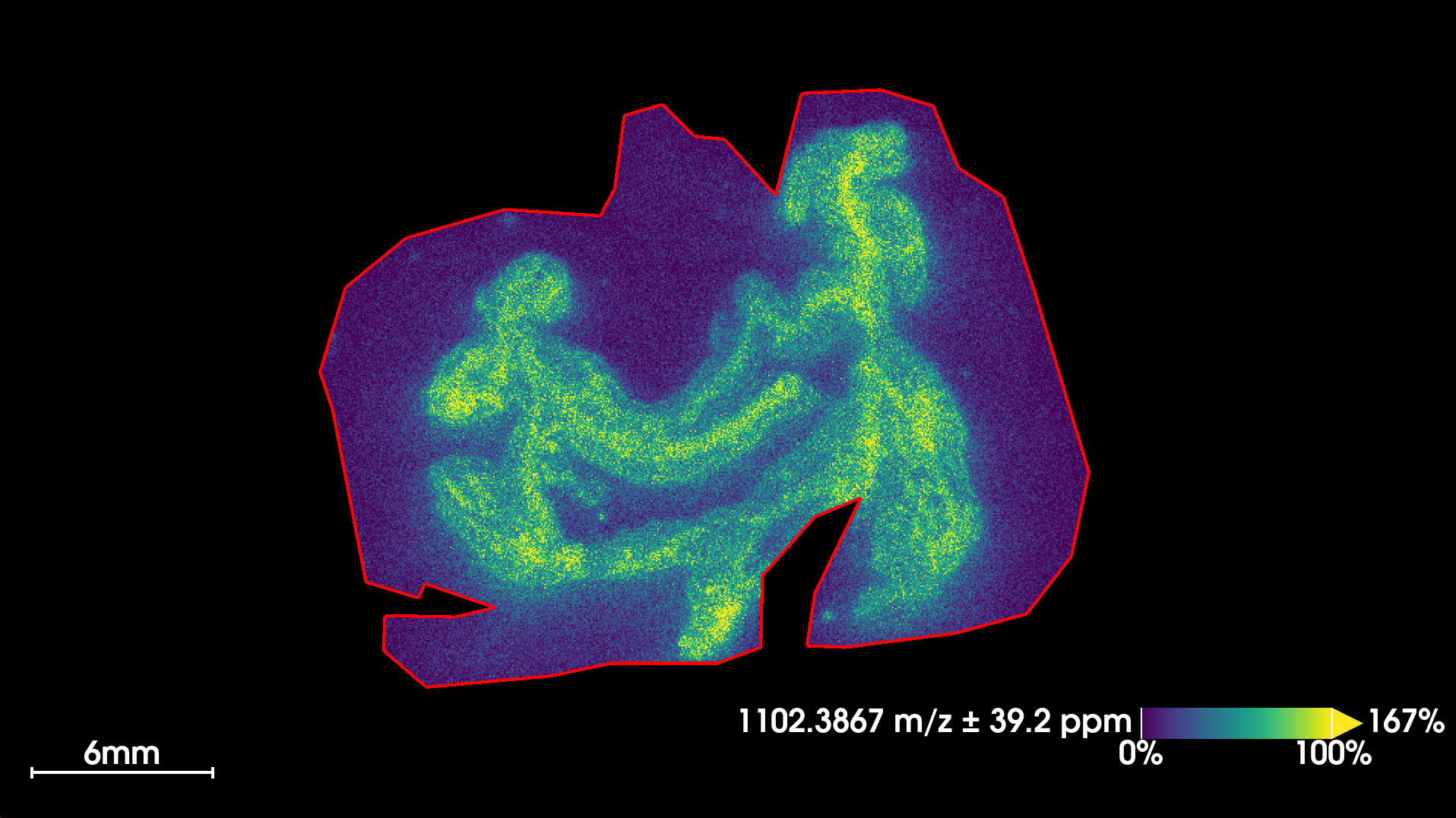

Supplement: Supplementary file 8 — Source Data 2 [file 41467_2026_72853_MOESM8_ESM.zip › Source Data MALDI Images/Figure 1/20250822_mz1102_HC_Image.png]

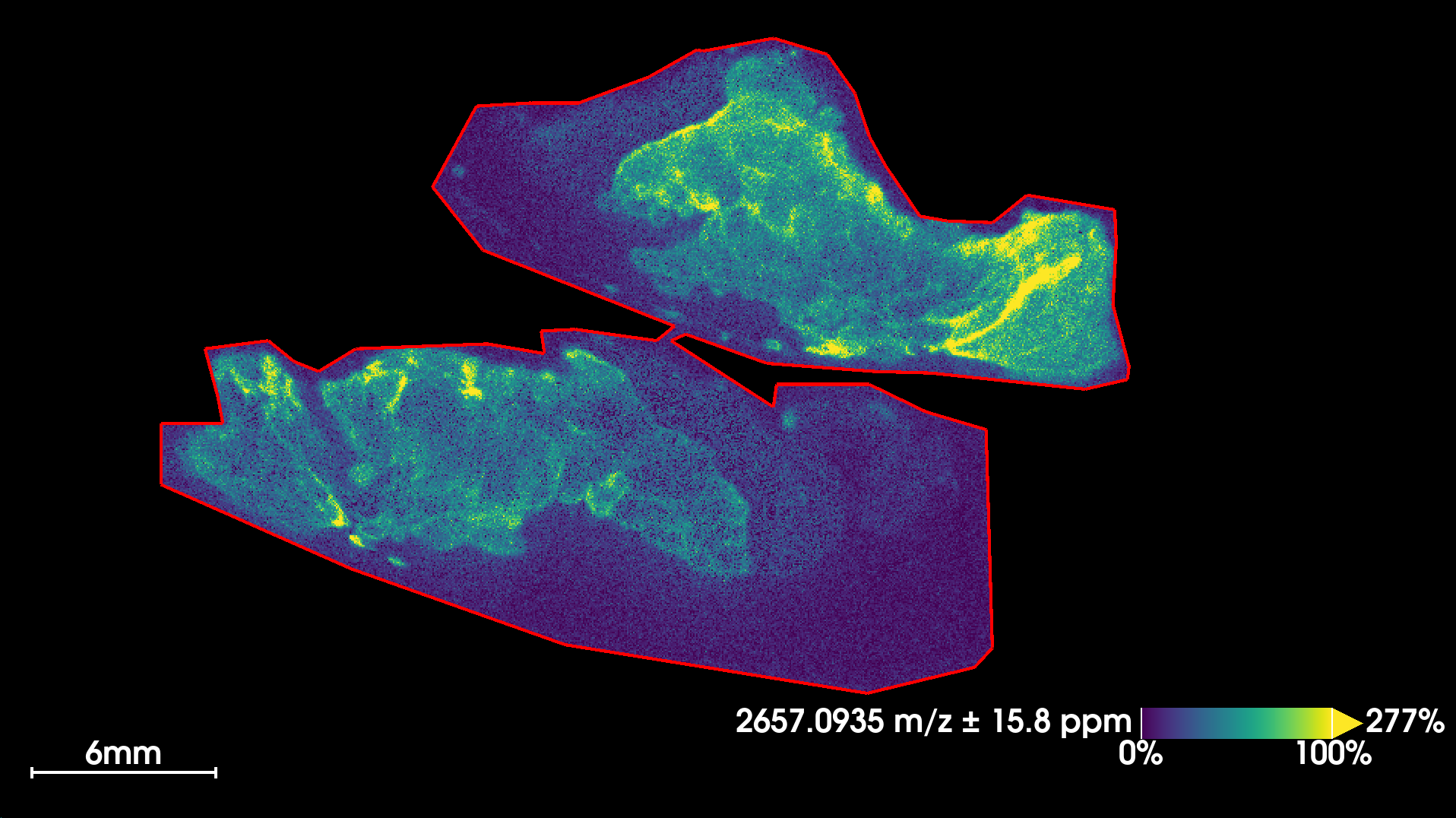

Supplement: Supplementary file 8 — Source Data 2 [file 41467_2026_72853_MOESM8_ESM.zip › Source Data MALDI Images/Figure 1/20250822_mz2657_Colon2_Image.png]

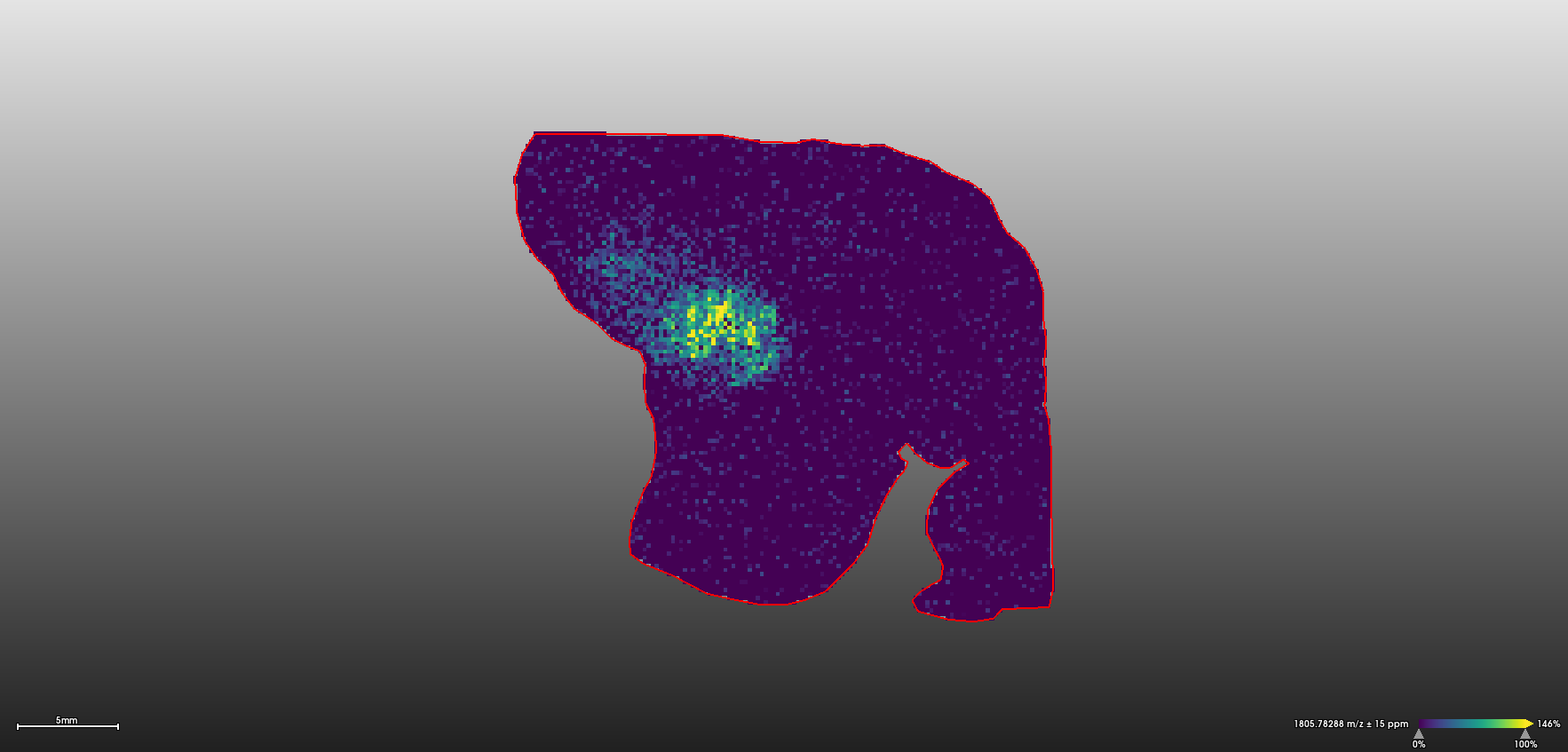

Supplement: Supplementary file 8 — Source Data 2 [file 41467_2026_72853_MOESM8_ESM.zip › Source Data MALDI Images/Figure 1/20240531_mz1805_Colon1b_Image.png]

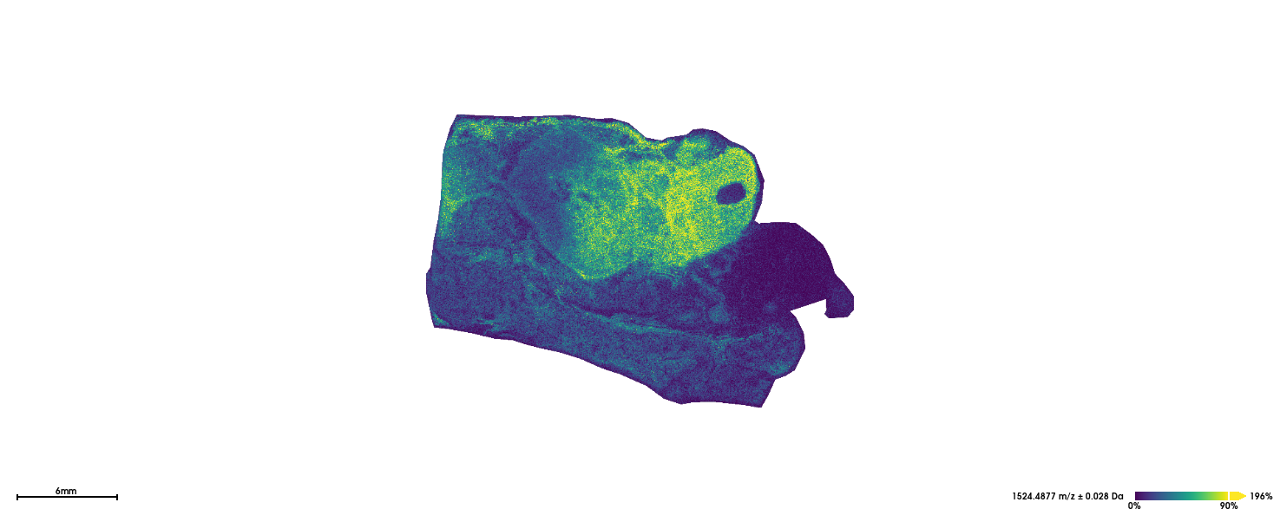

Supplement: Supplementary file 8 — Source Data 2 [file 41467_2026_72853_MOESM8_ESM.zip › Source Data MALDI Images/Figure 1/20260124_mz1524_SG_Image.png]

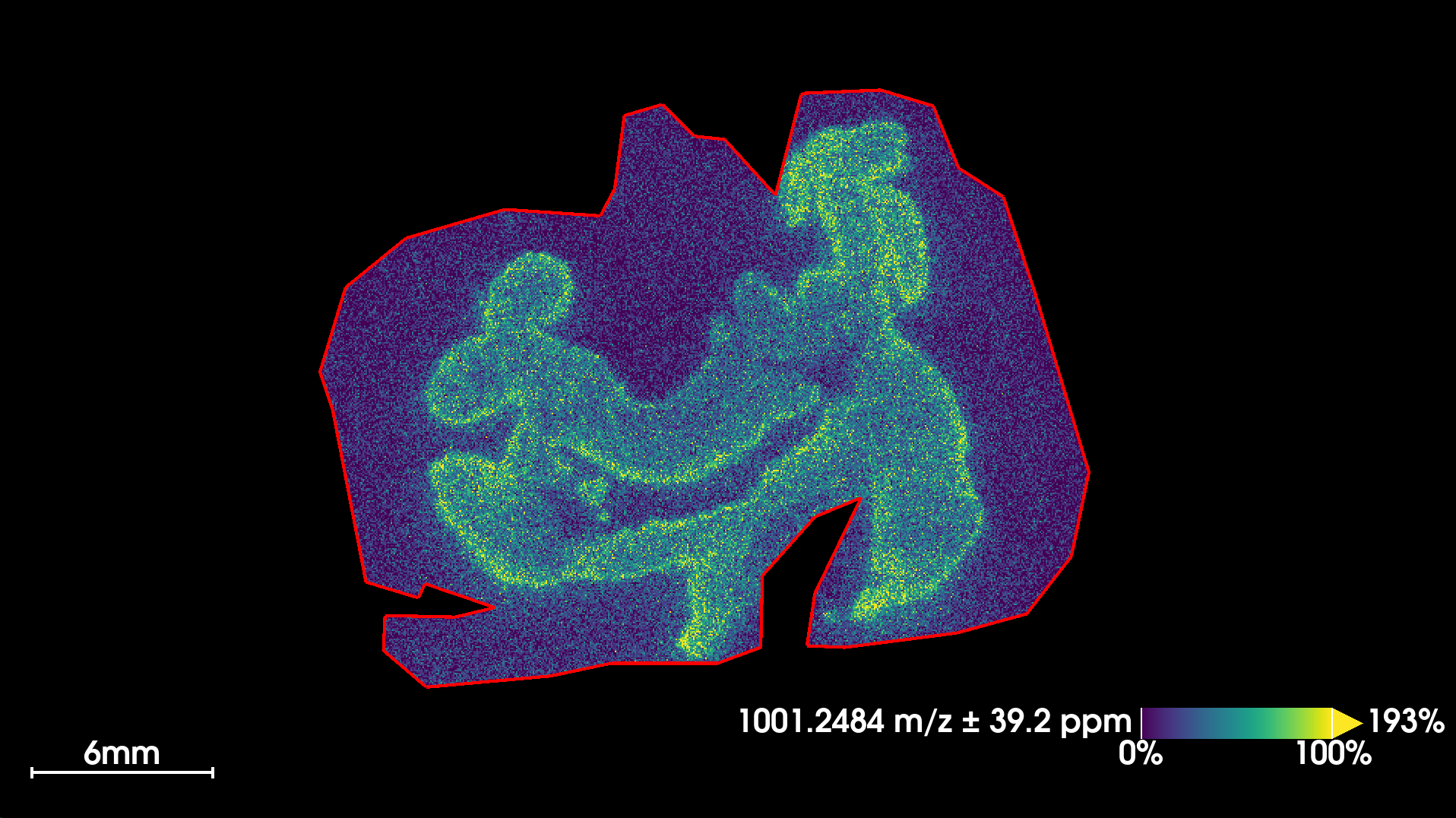

Supplement: Supplementary file 8 — Source Data 2 [file 41467_2026_72853_MOESM8_ESM.zip › Source Data MALDI Images/Figure 1/20250822_mz1001_HC_Image.png]

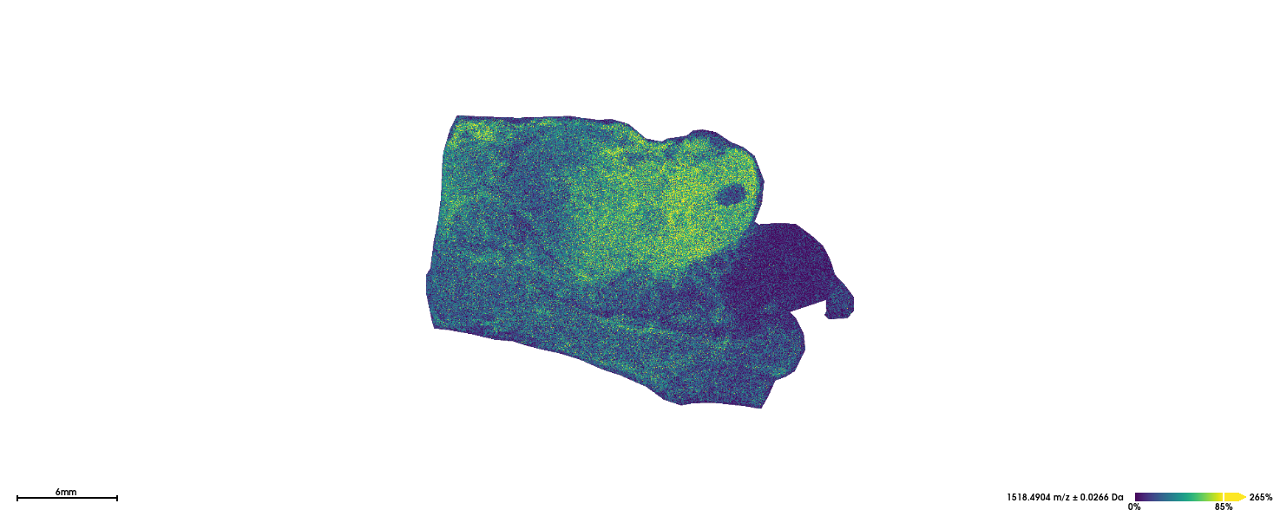

Supplement: Supplementary file 8 — Source Data 2 [file 41467_2026_72853_MOESM8_ESM.zip › Source Data MALDI Images/Figure 1/20260124_mz1518_SG_Image.png]

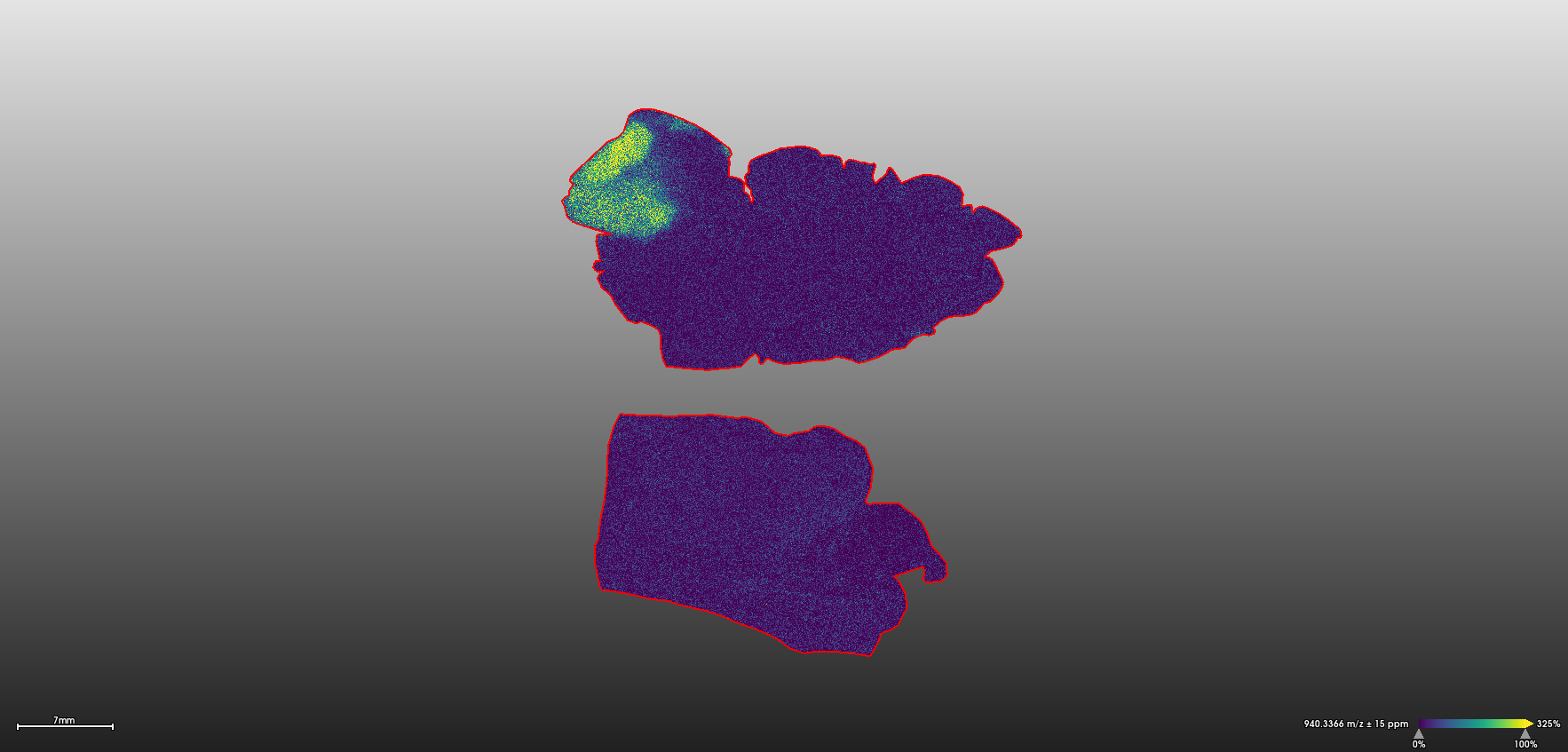

Supplement: Supplementary file 8 — Source Data 2 [file 41467_2026_72853_MOESM8_ESM.zip › Source Data MALDI Images/Figure 1/20240531_mz940_Eso_Image.png]

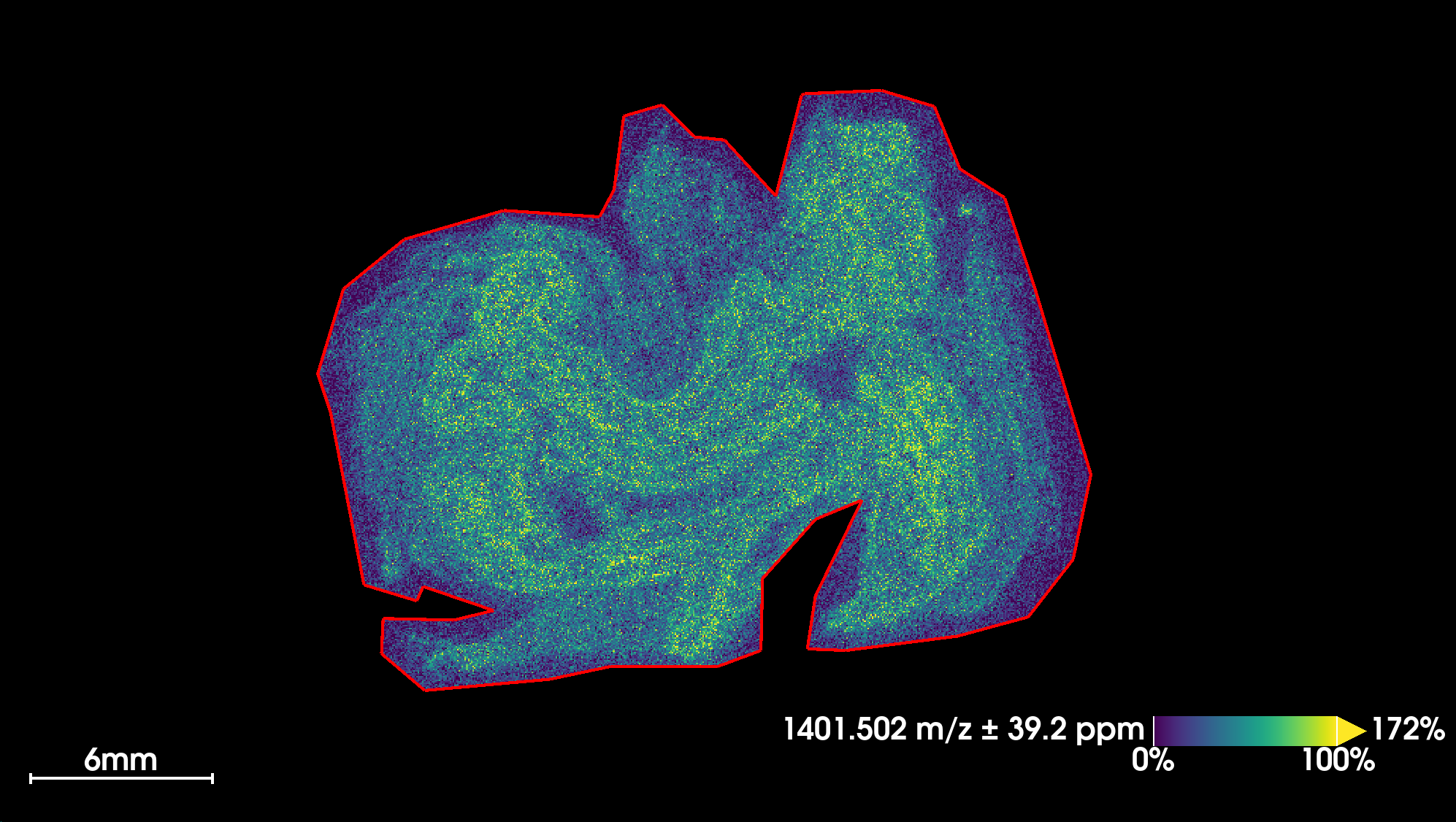

Supplement: Supplementary file 8 — Source Data 2 [file 41467_2026_72853_MOESM8_ESM.zip › Source Data MALDI Images/Figure 1/20250822_mz1401_HC_Image.png]

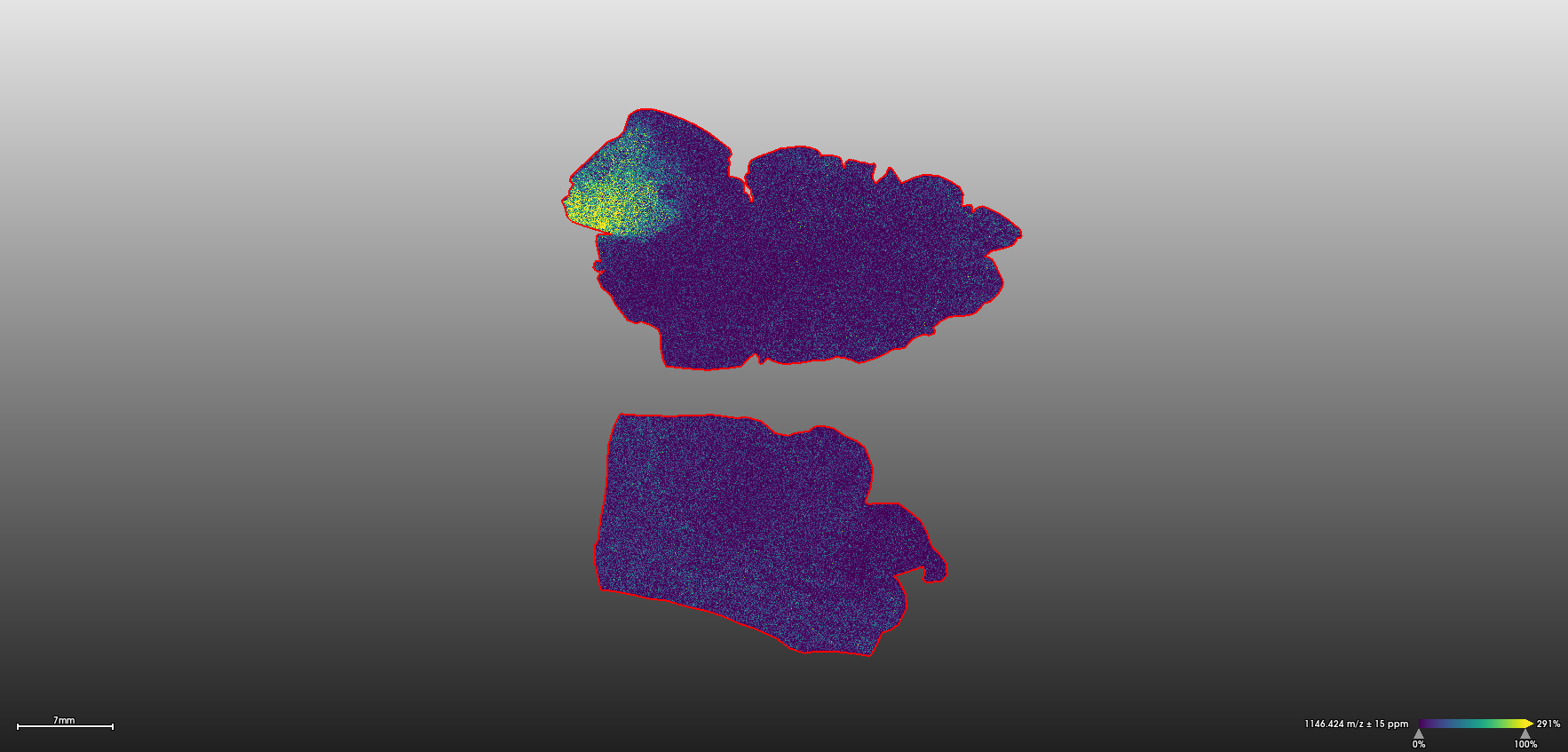

Supplement: Supplementary file 8 — Source Data 2 [file 41467_2026_72853_MOESM8_ESM.zip › Source Data MALDI Images/Figure 1/20240614_mz1146_Eso_Image.png]

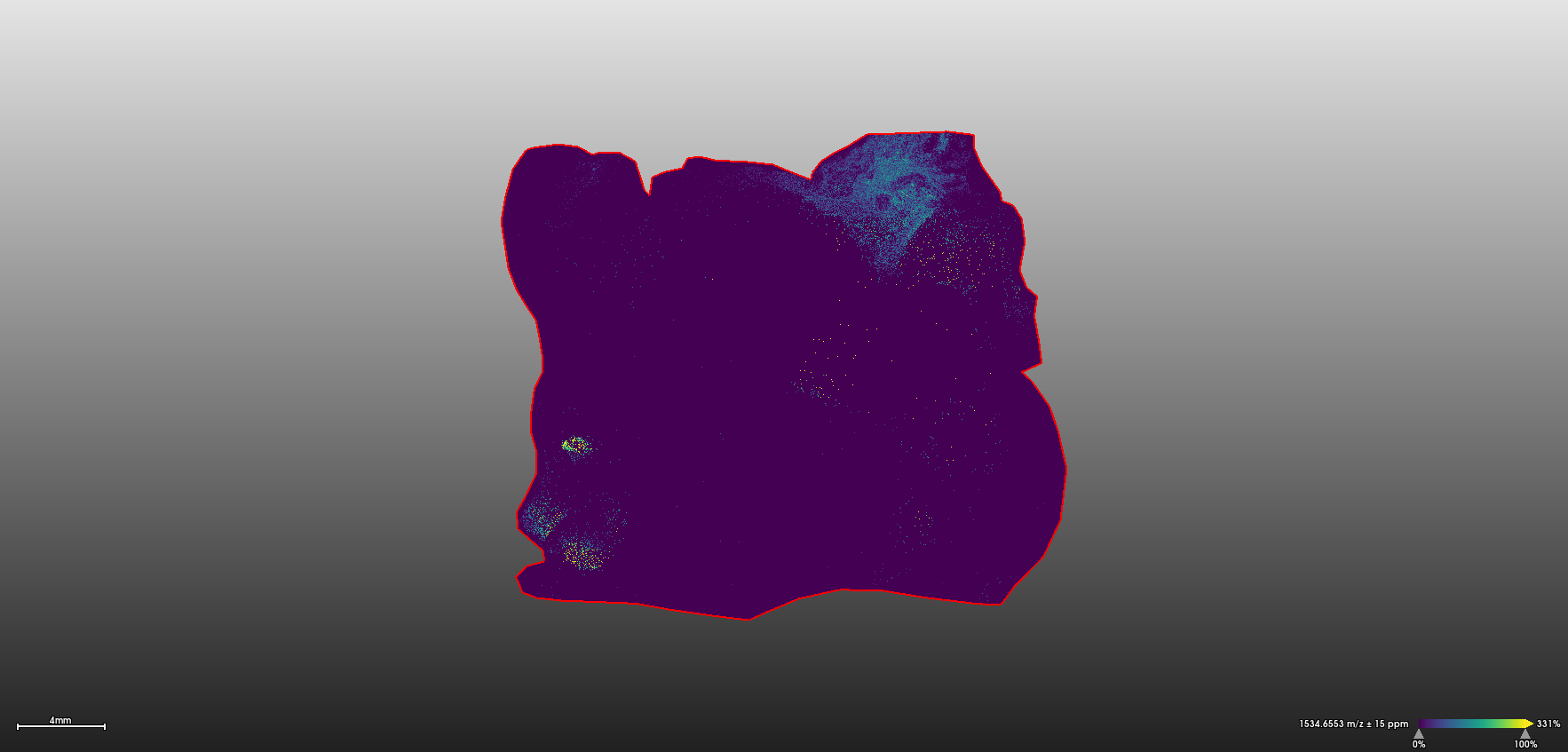

Supplement: Supplementary file 8 — Source Data 2 [file 41467_2026_72853_MOESM8_ESM.zip › Source Data MALDI Images/Supplementary Figure 11/20240531_TPSPPTT+N4_Colon1a.png]

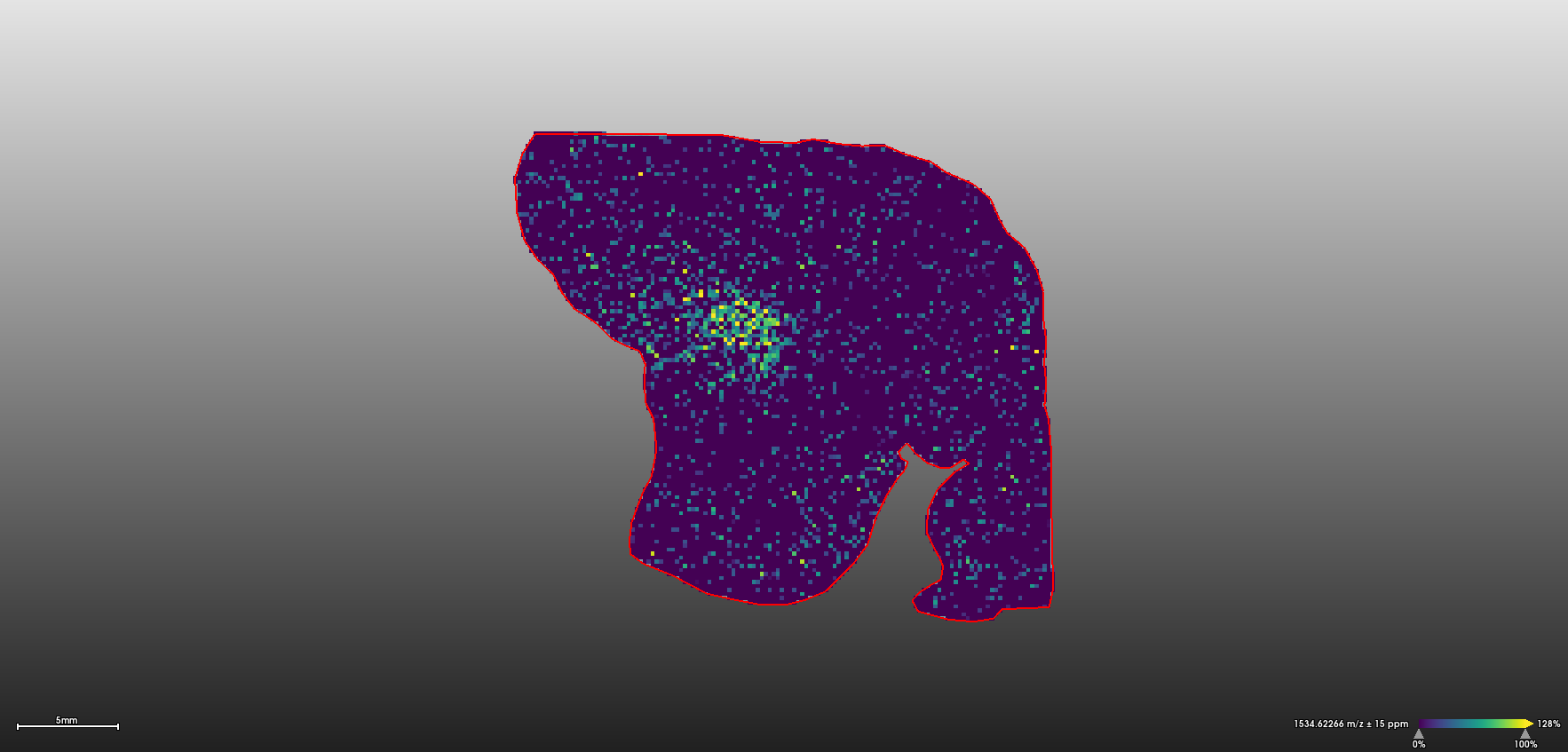

Supplement: Supplementary file 8 — Source Data 2 [file 41467_2026_72853_MOESM8_ESM.zip › Source Data MALDI Images/Supplementary Figure 11/20240531_TPSPPTT+N4_Colon1b.png]

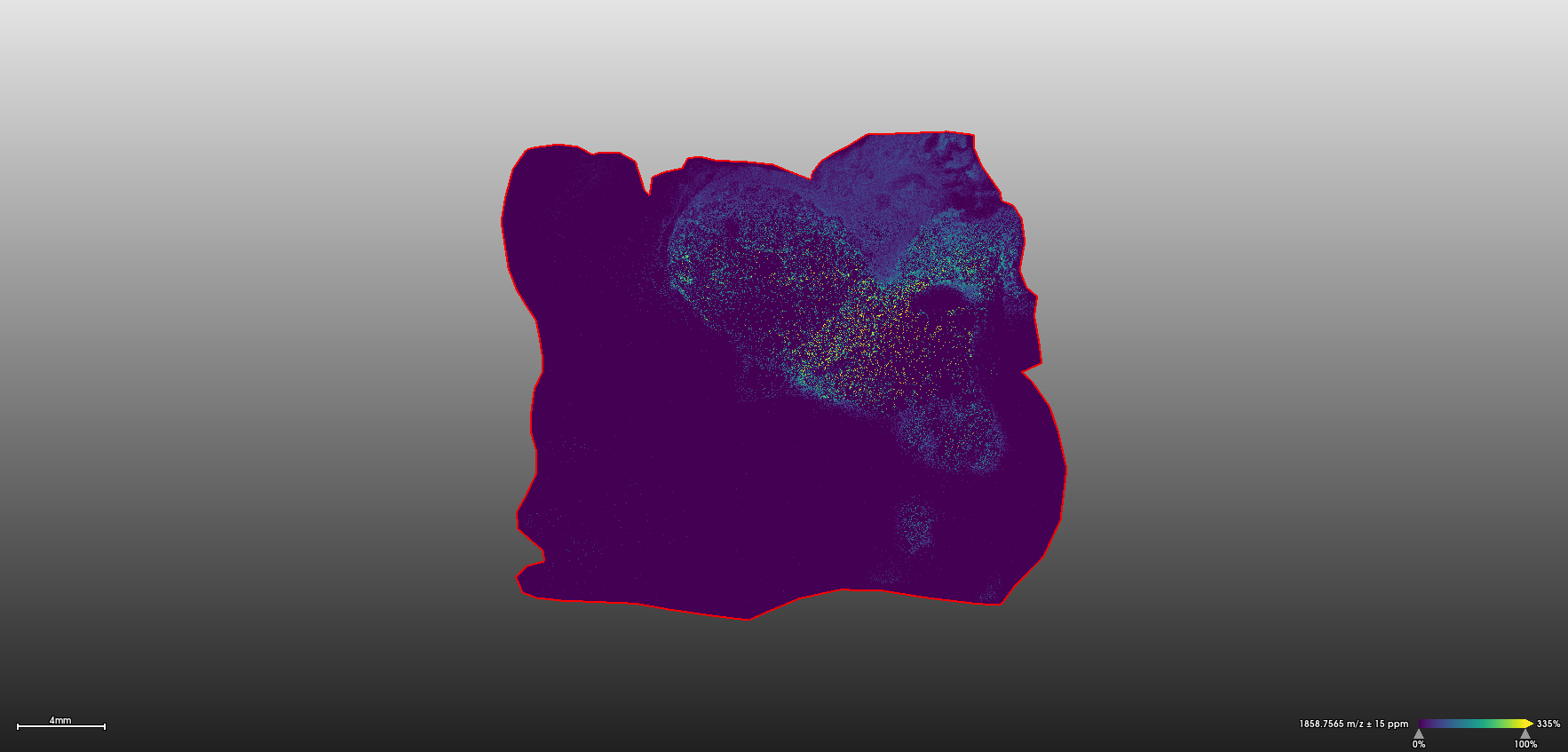

Supplement: Supplementary file 8 — Source Data 2 [file 41467_2026_72853_MOESM8_ESM.zip › Source Data MALDI Images/Supplementary Figure 11/20240627_TPSPPTT+H2N4_Colon1a.png]

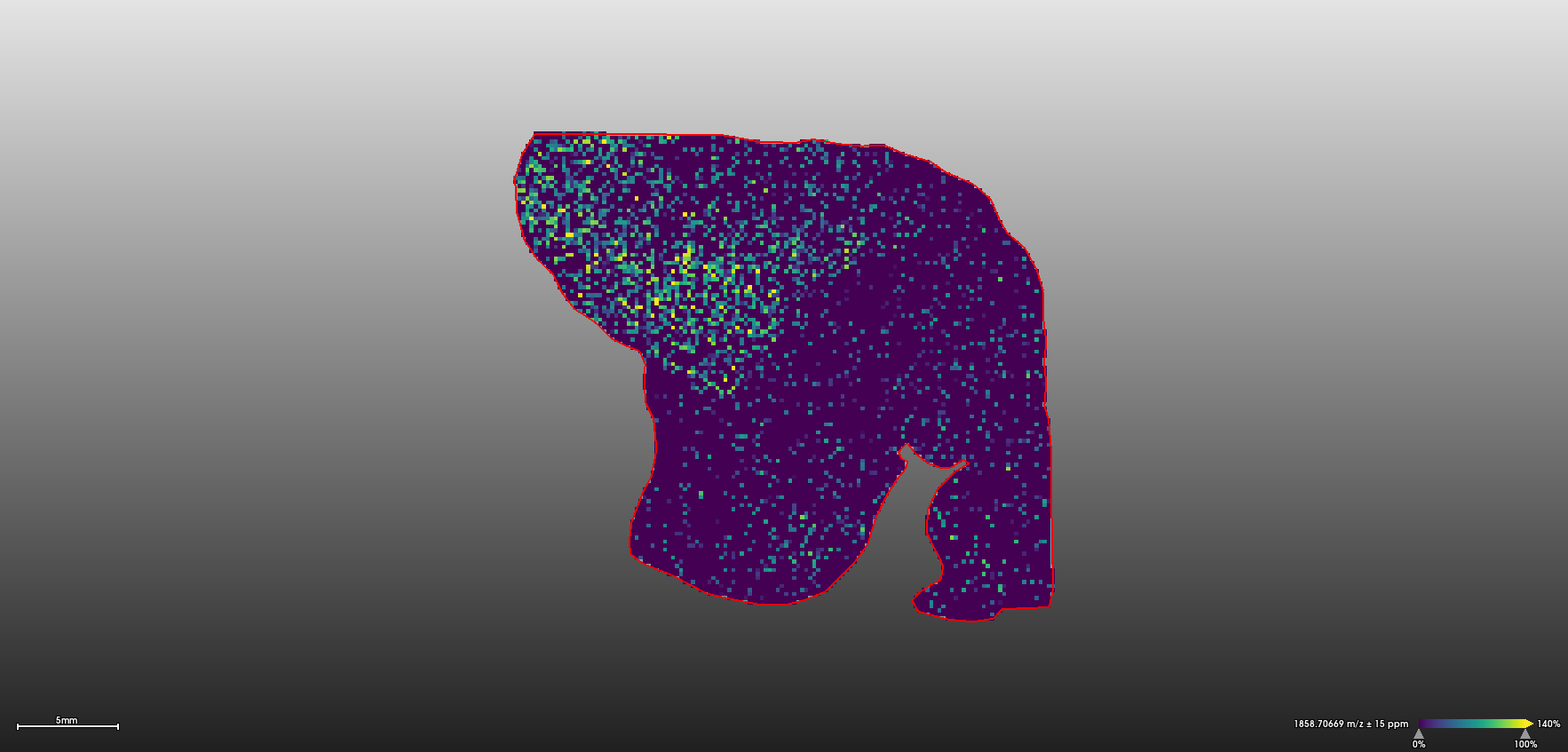

Supplement: Supplementary file 8 — Source Data 2 [file 41467_2026_72853_MOESM8_ESM.zip › Source Data MALDI Images/Supplementary Figure 11/20240627_TPSPPTT+H2N4_Colon1b.png]

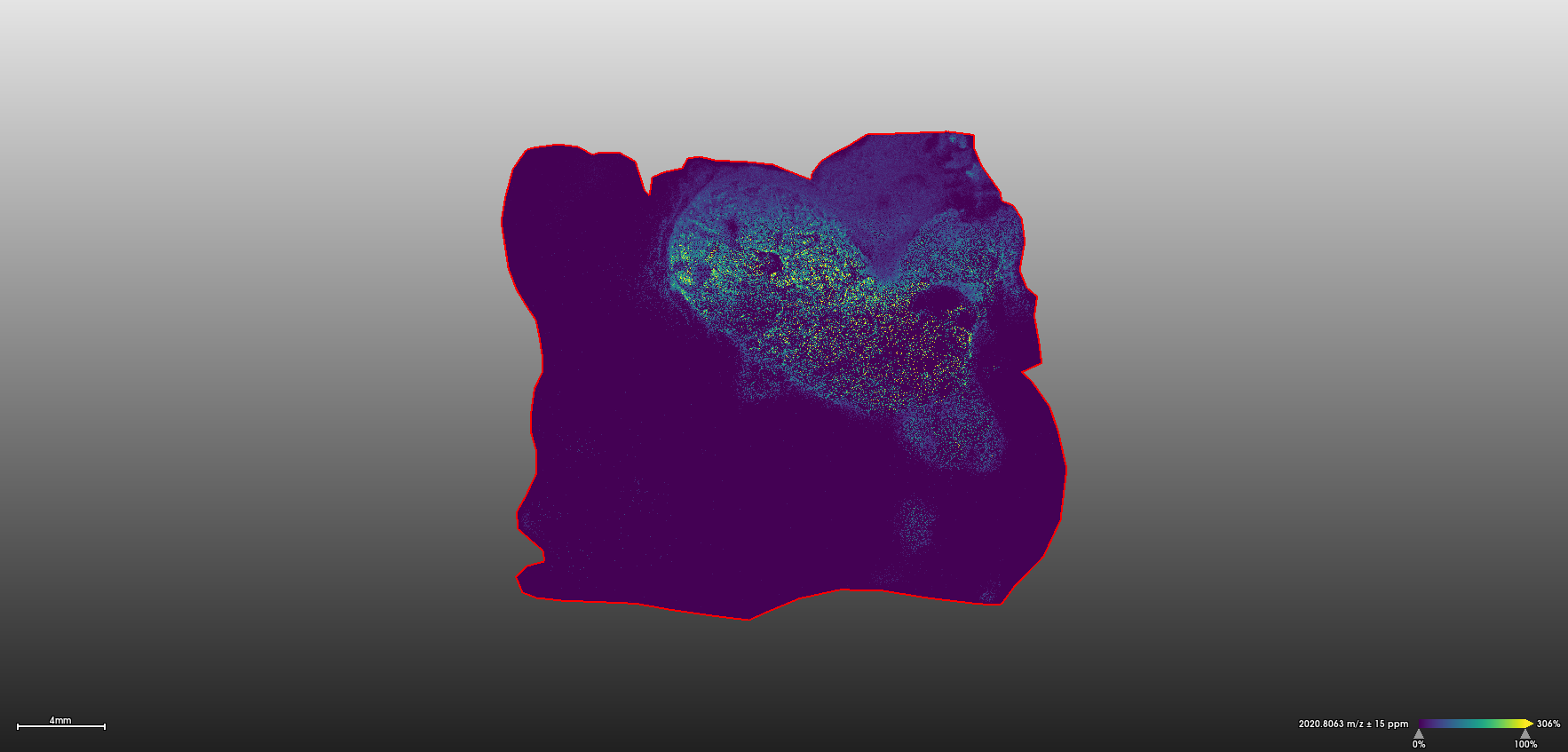

Supplement: Supplementary file 8 — Source Data 2 [file 41467_2026_72853_MOESM8_ESM.zip › Source Data MALDI Images/Supplementary Figure 11/20240627_TPSPPTT+H3N4_Colon1a.png]

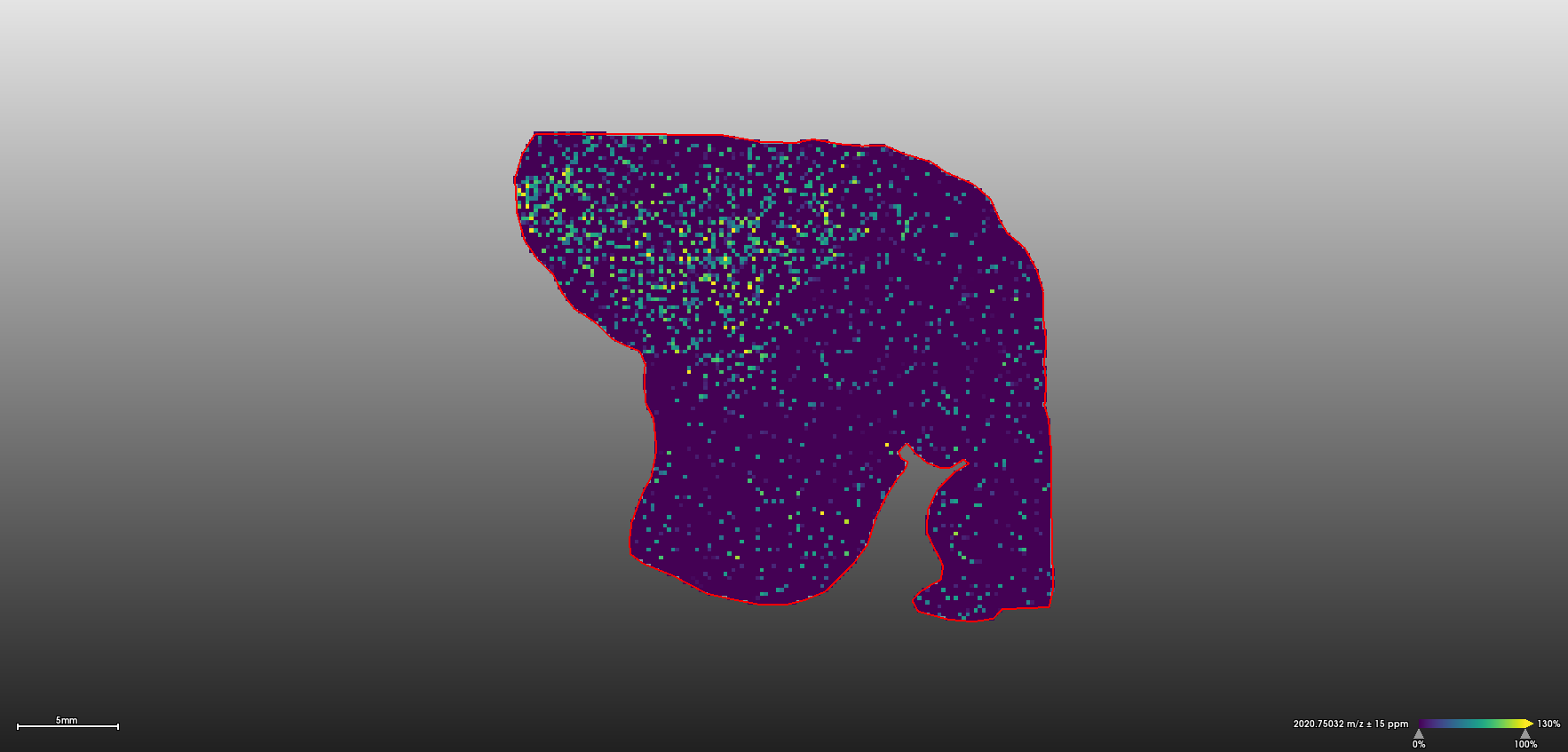

Supplement: Supplementary file 8 — Source Data 2 [file 41467_2026_72853_MOESM8_ESM.zip › Source Data MALDI Images/Supplementary Figure 11/20240627_TPSPPTT+H3N4_Colon1b.png]

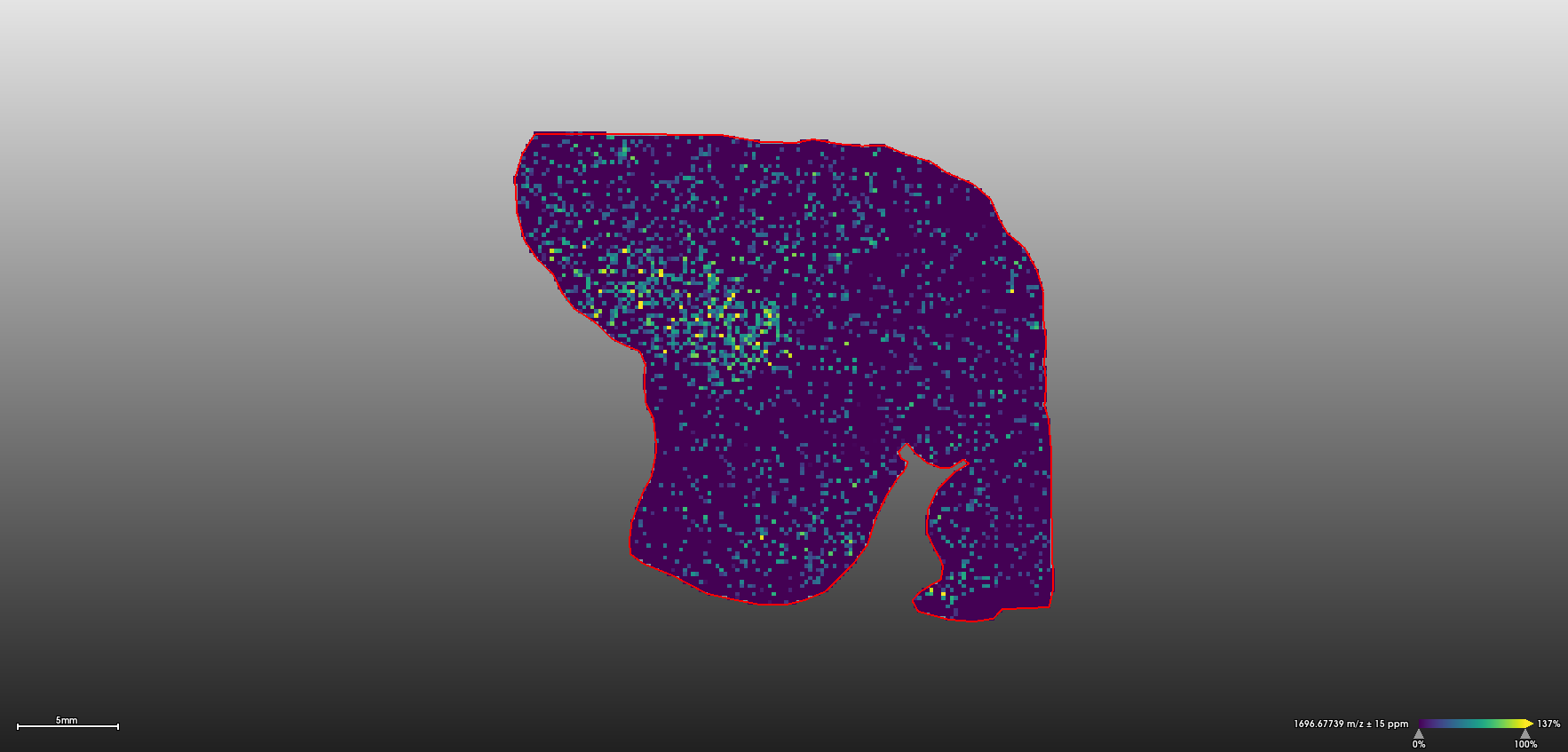

Supplement: Supplementary file 8 — Source Data 2 [file 41467_2026_72853_MOESM8_ESM.zip › Source Data MALDI Images/Supplementary Figure 11/20240531_TPSPPTT+H1N4_Colon1b.png]

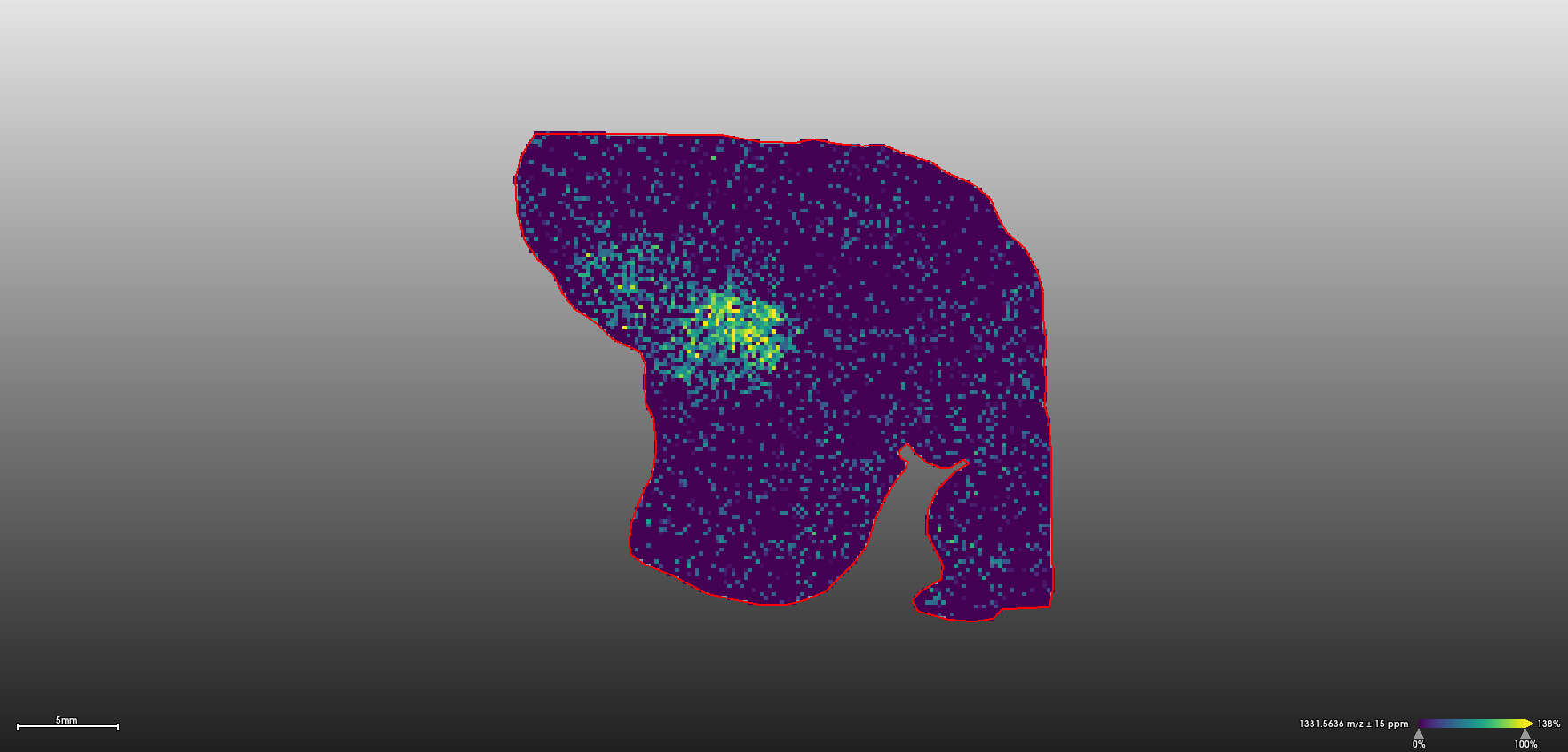

Supplement: Supplementary file 8 — Source Data 2 [file 41467_2026_72853_MOESM8_ESM.zip › Source Data MALDI Images/Supplementary Figure 11/20240531_TPSPPTT+N3_Colon1b.png]

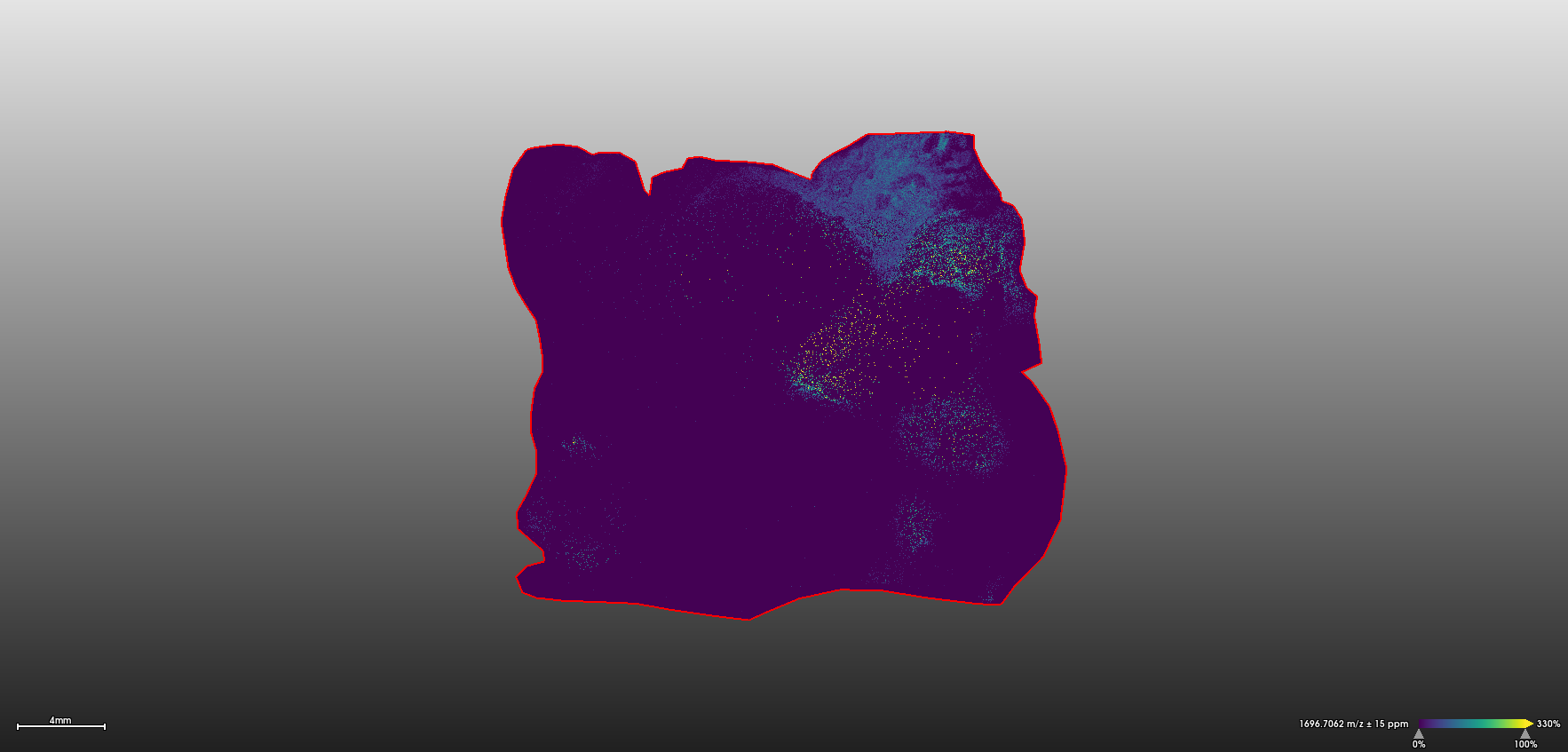

Supplement: Supplementary file 8 — Source Data 2 [file 41467_2026_72853_MOESM8_ESM.zip › Source Data MALDI Images/Supplementary Figure 11/20240531_TPSPPTT+H1N4_Colon1a.png]

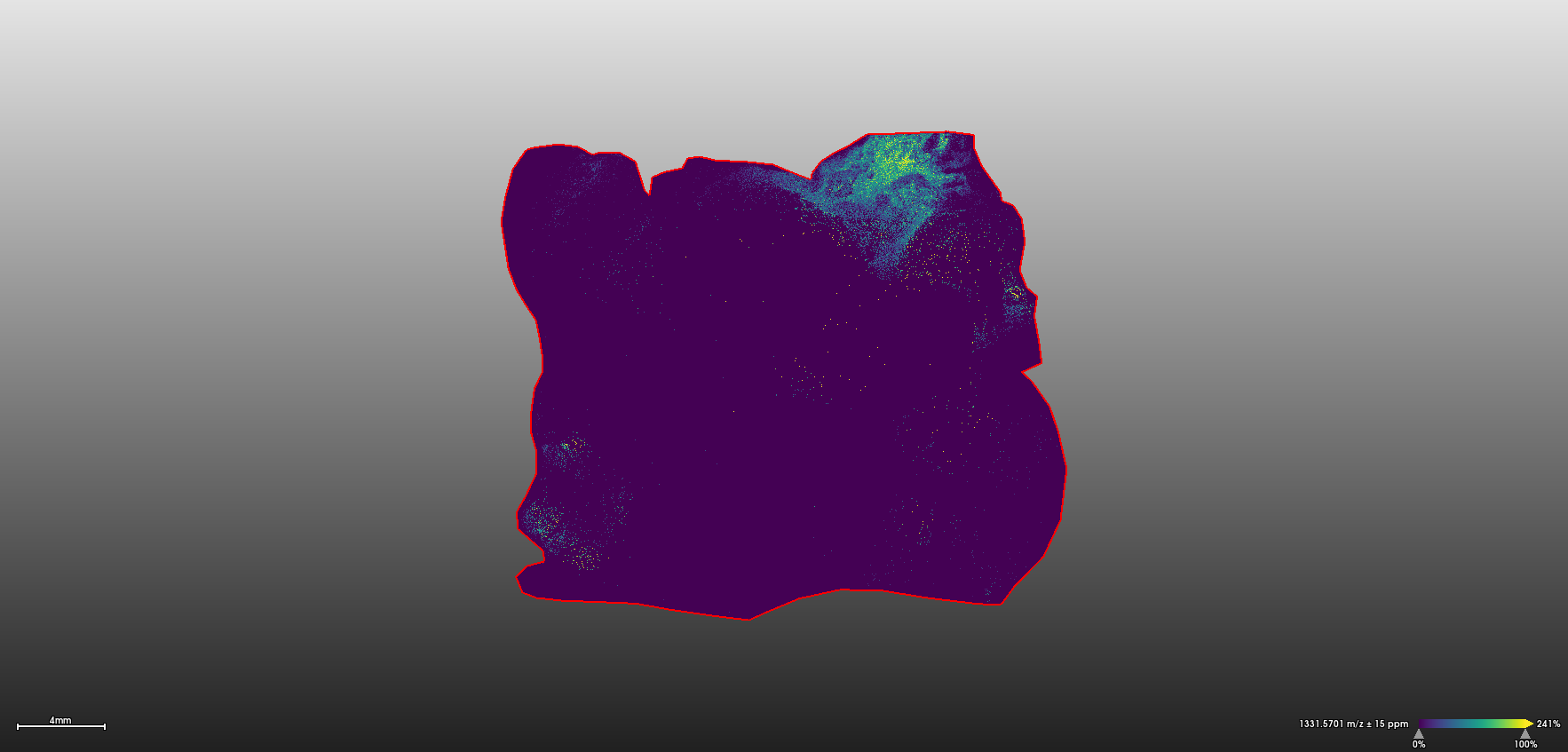

Supplement: Supplementary file 8 — Source Data 2 [file 41467_2026_72853_MOESM8_ESM.zip › Source Data MALDI Images/Supplementary Figure 11/20240531_TPSPPTT+N3_Colon1a.png]

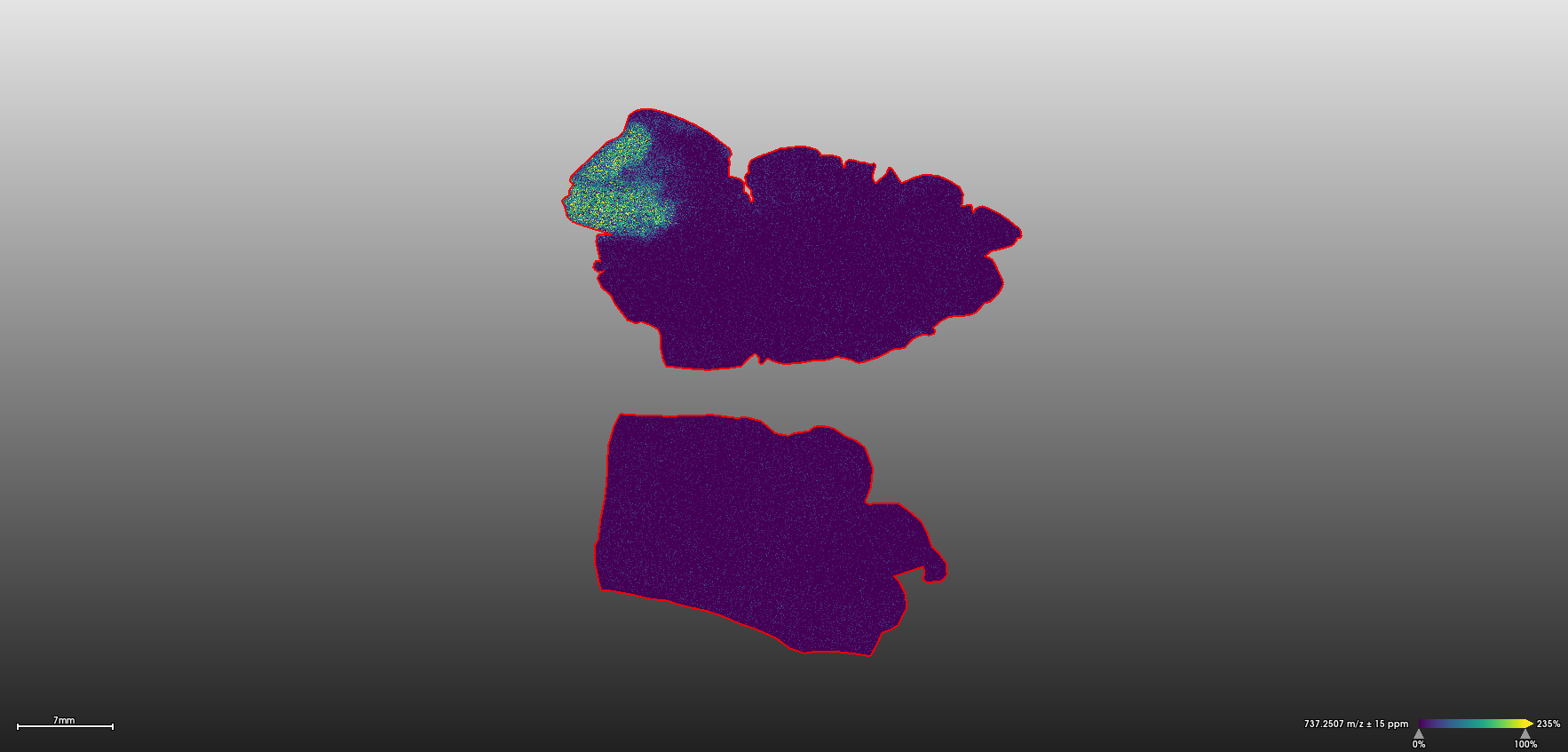

Supplement: Supplementary file 8 — Source Data 2 [file 41467_2026_72853_MOESM8_ESM.zip › Source Data MALDI Images/Supplementary Figure 32/20240614_737_Eso_Image.png]

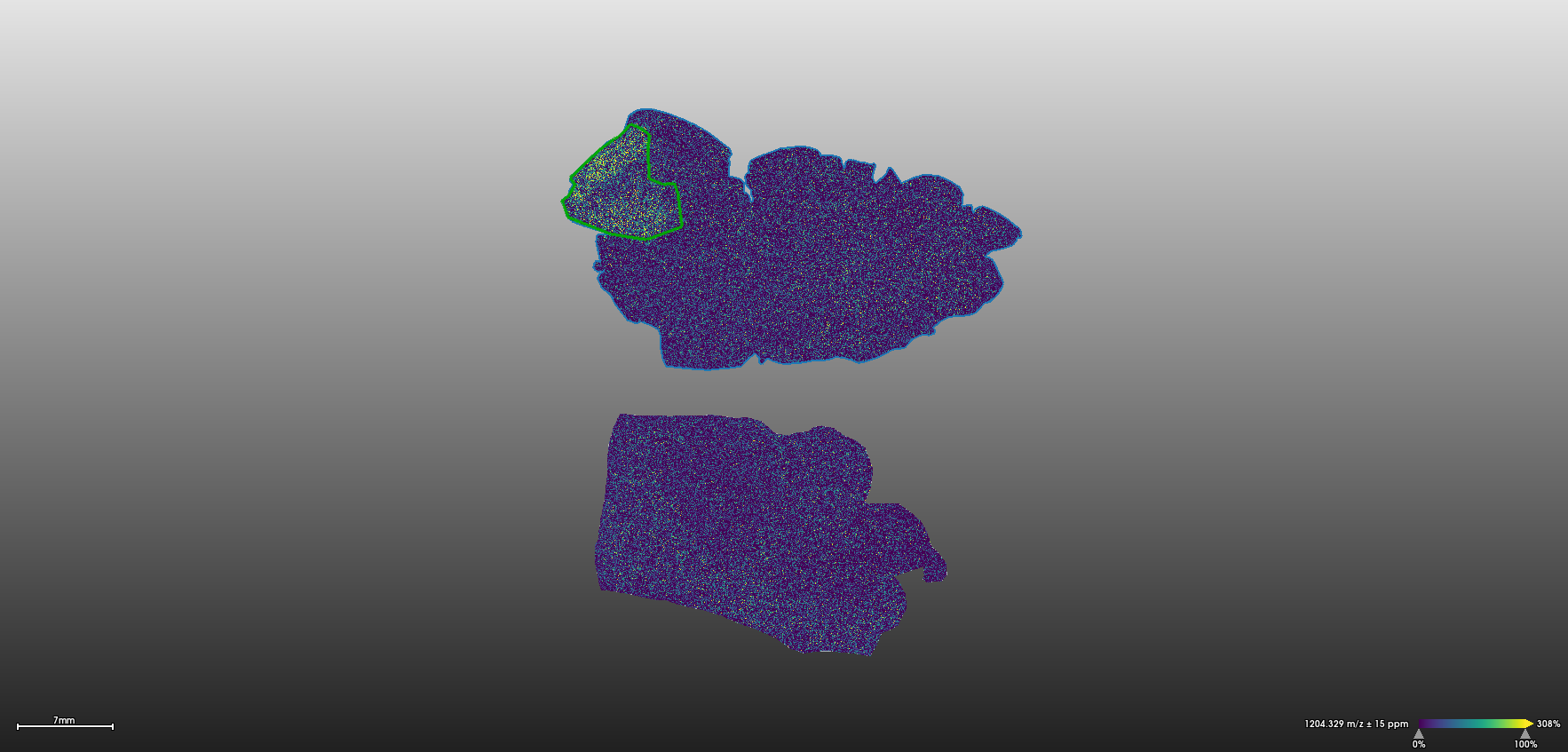

Supplement: Supplementary file 8 — Source Data 2 [file 41467_2026_72853_MOESM8_ESM.zip › Source Data MALDI Images/Supplementary Figure 32/20240627_1204_Eso_Image.png]

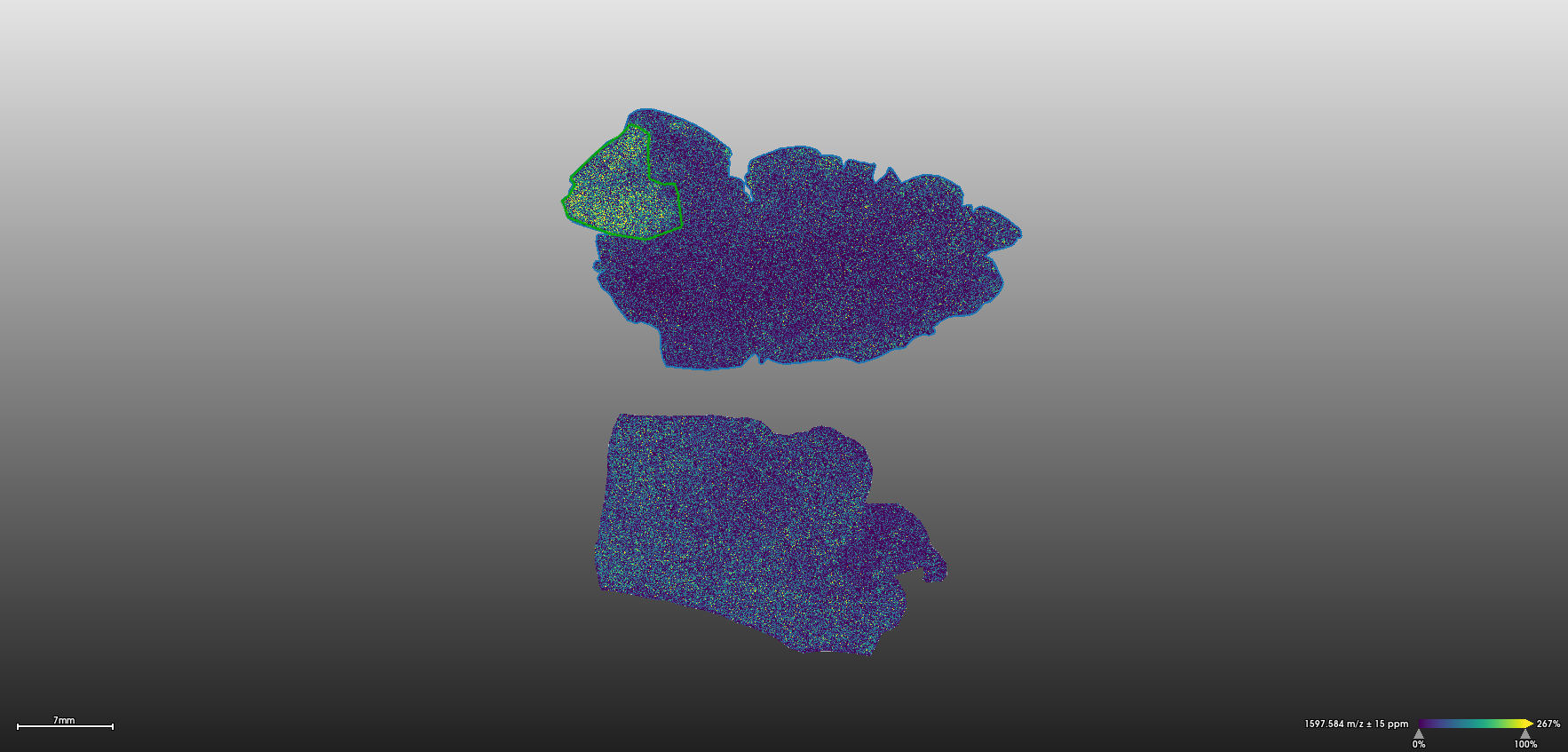

Supplement: Supplementary file 8 — Source Data 2 [file 41467_2026_72853_MOESM8_ESM.zip › Source Data MALDI Images/Supplementary Figure 32/20240627_1597_Eso_Image.png]

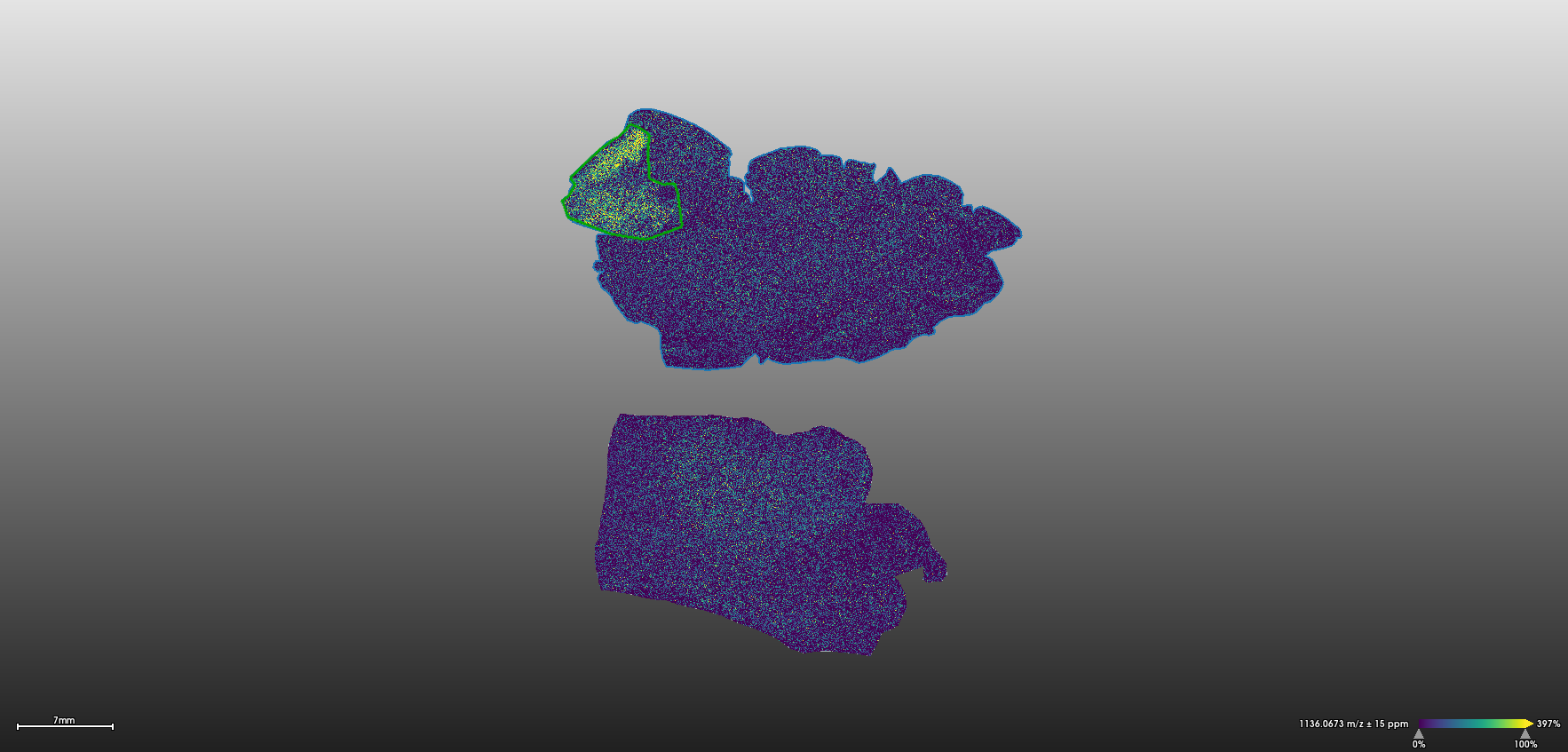

Supplement: Supplementary file 8 — Source Data 2 [file 41467_2026_72853_MOESM8_ESM.zip › Source Data MALDI Images/Supplementary Figure 32/20240627_1136_Eso_Image.png]

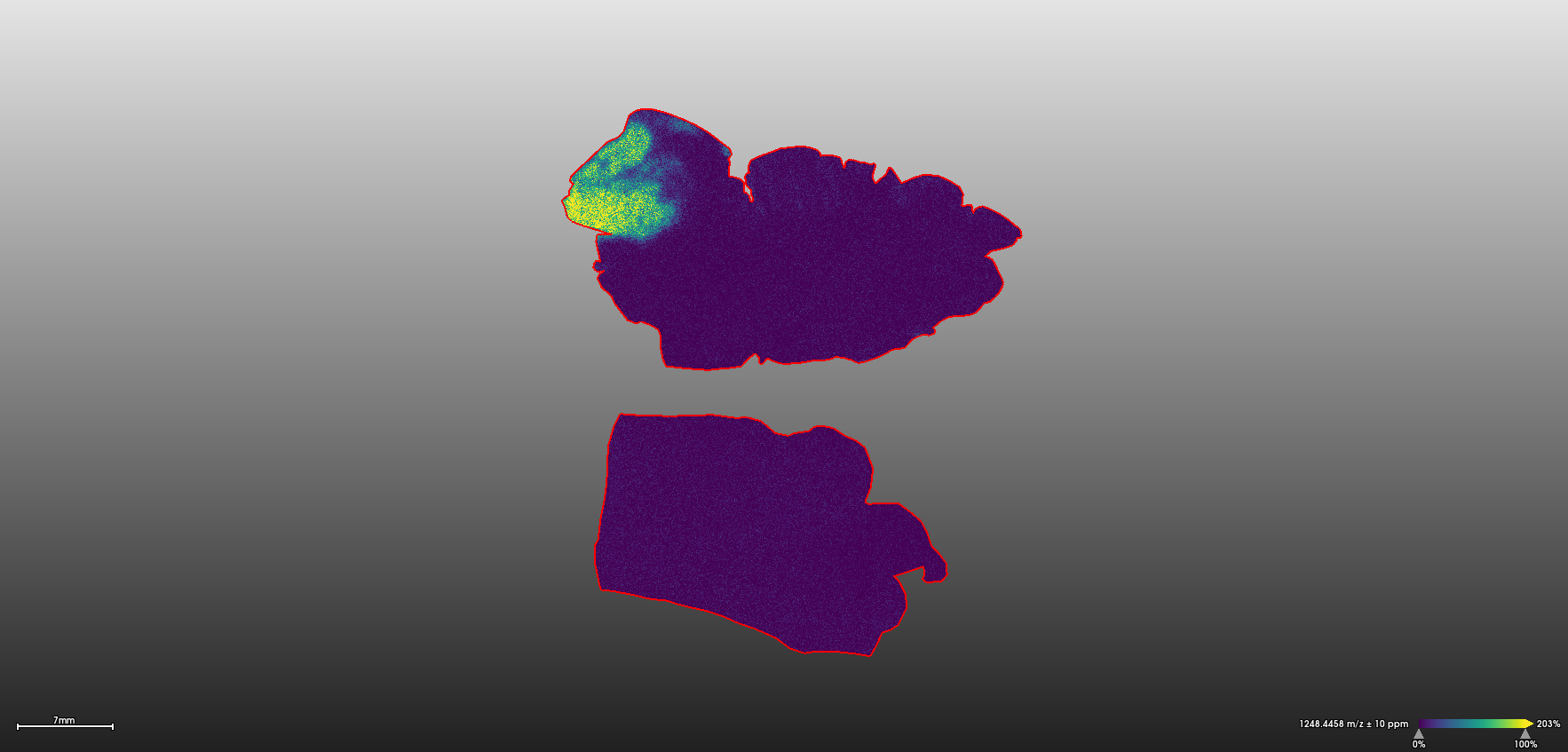

Supplement: Supplementary file 8 — Source Data 2 [file 41467_2026_72853_MOESM8_ESM.zip › Source Data MALDI Images/Supplementary Figure 32/20240524_1248_Eso_Image.png]

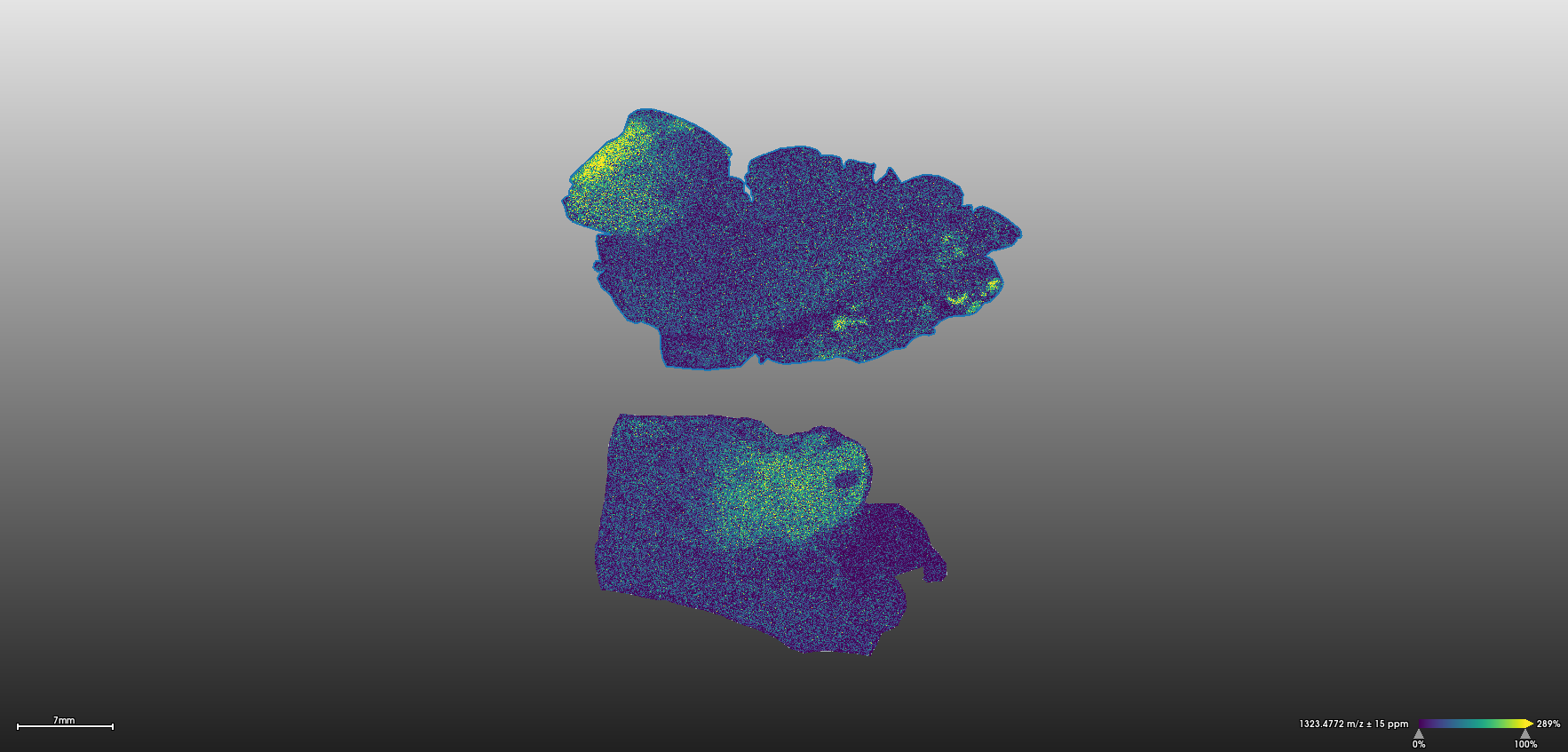

Supplement: Supplementary file 8 — Source Data 2 [file 41467_2026_72853_MOESM8_ESM.zip › Source Data MALDI Images/Supplementary Figure 32/20240627_1323_Eso_Image.png]

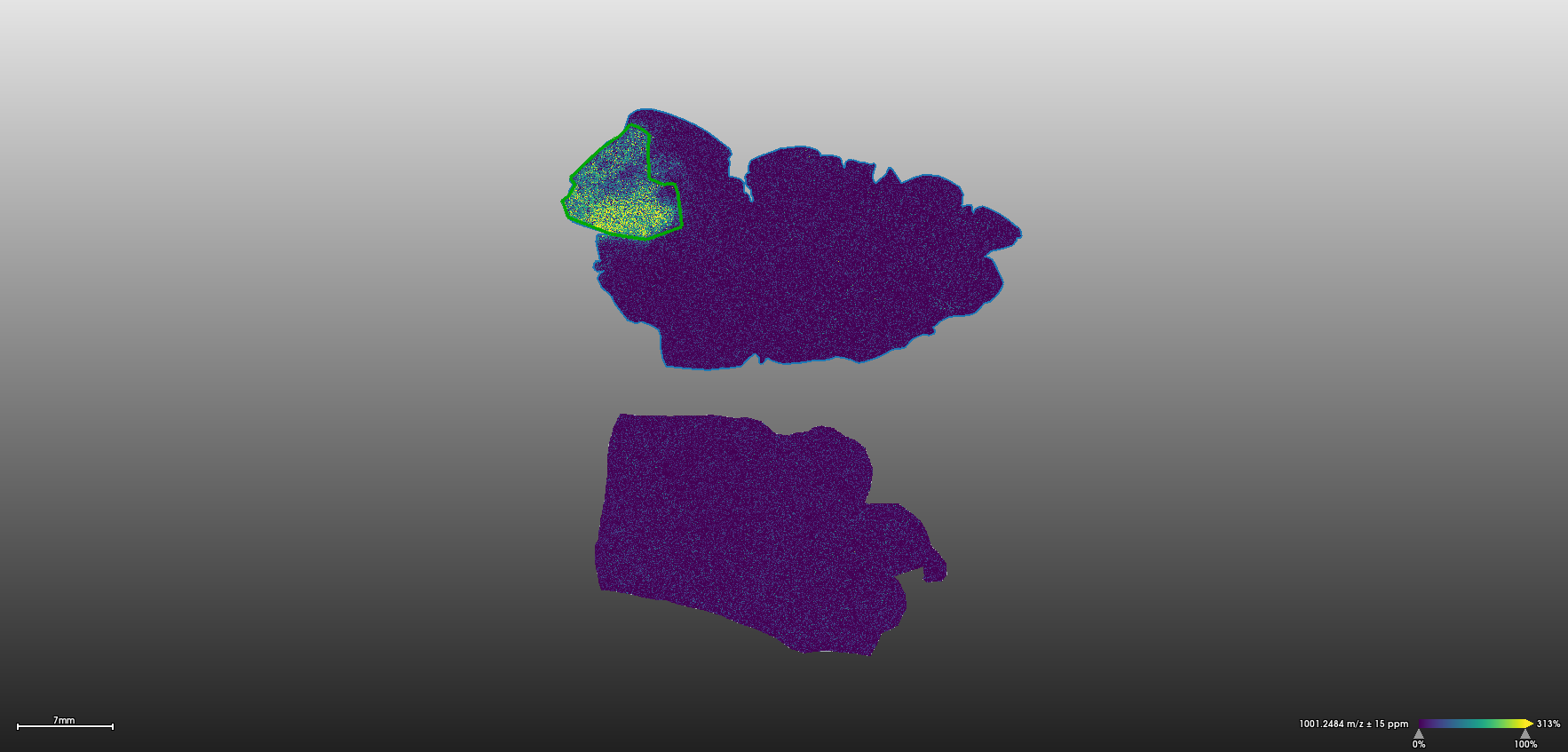

Supplement: Supplementary file 8 — Source Data 2 [file 41467_2026_72853_MOESM8_ESM.zip › Source Data MALDI Images/Supplementary Figure 32/20240627_1001_Eso_Image.png]

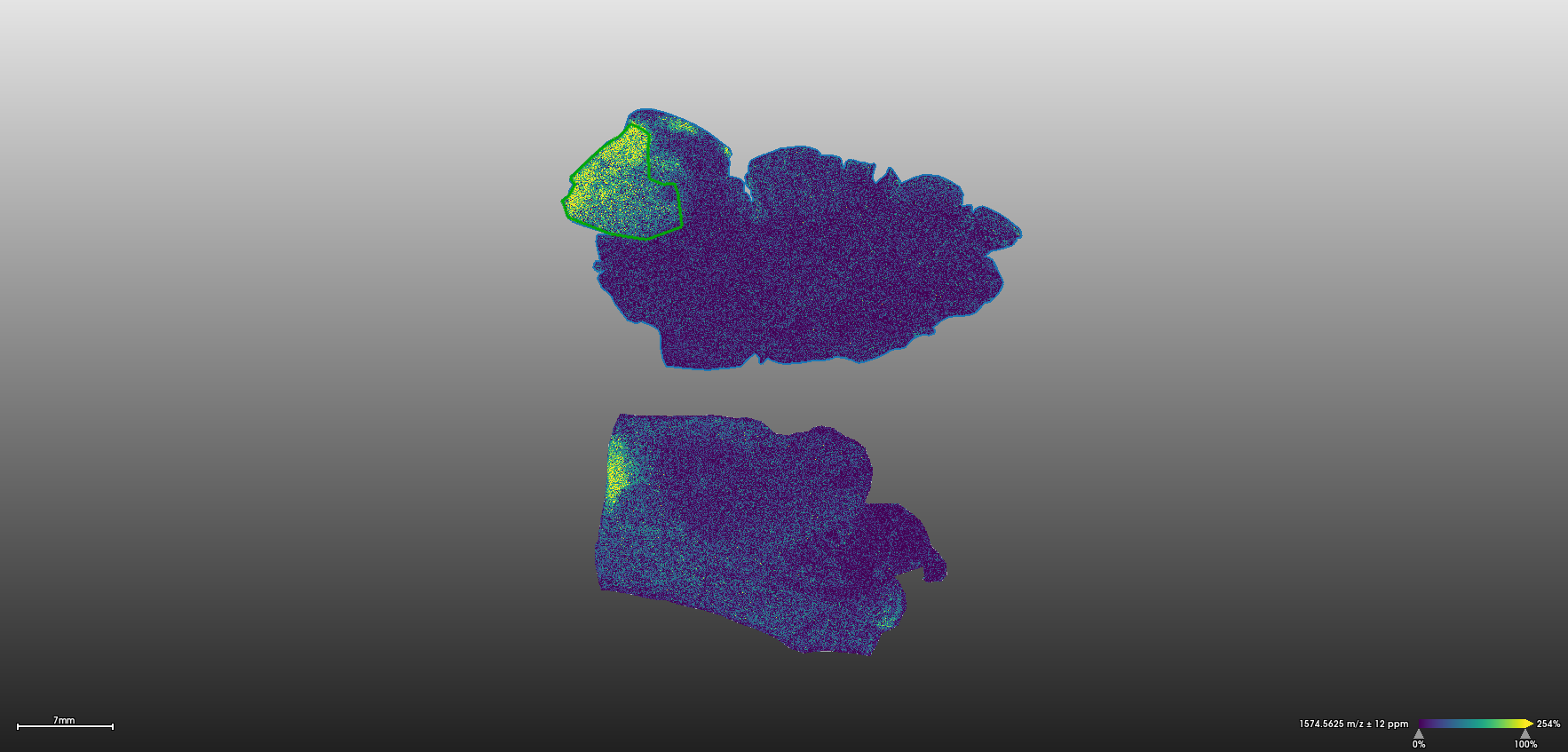

Supplement: Supplementary file 8 — Source Data 2 [file 41467_2026_72853_MOESM8_ESM.zip › Source Data MALDI Images/Supplementary Figure 32/20240627_1574_Eso_Image.png]

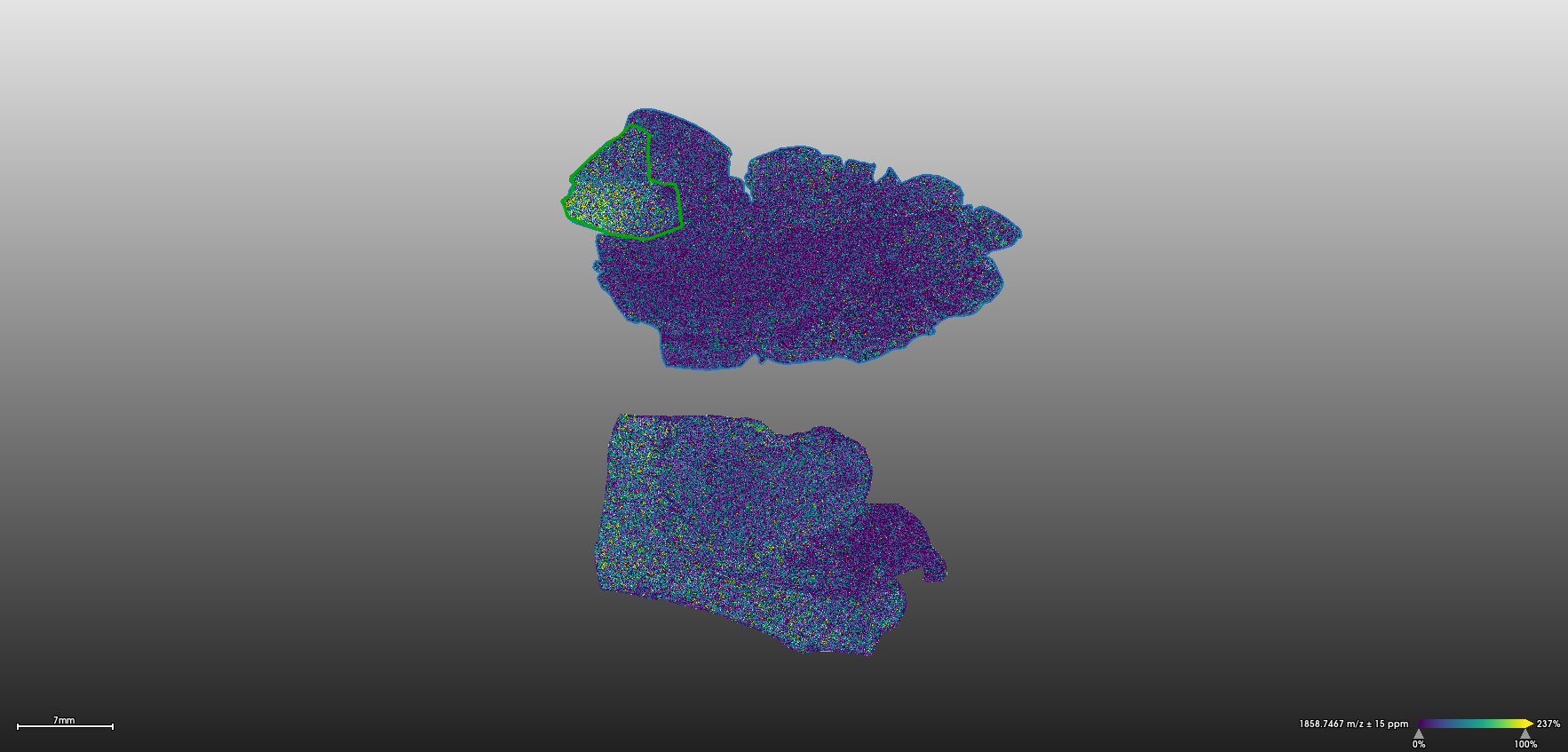

Supplement: Supplementary file 8 — Source Data 2 [file 41467_2026_72853_MOESM8_ESM.zip › Source Data MALDI Images/Supplementary Figure 32/20240627_1858_Eso_Image.png]

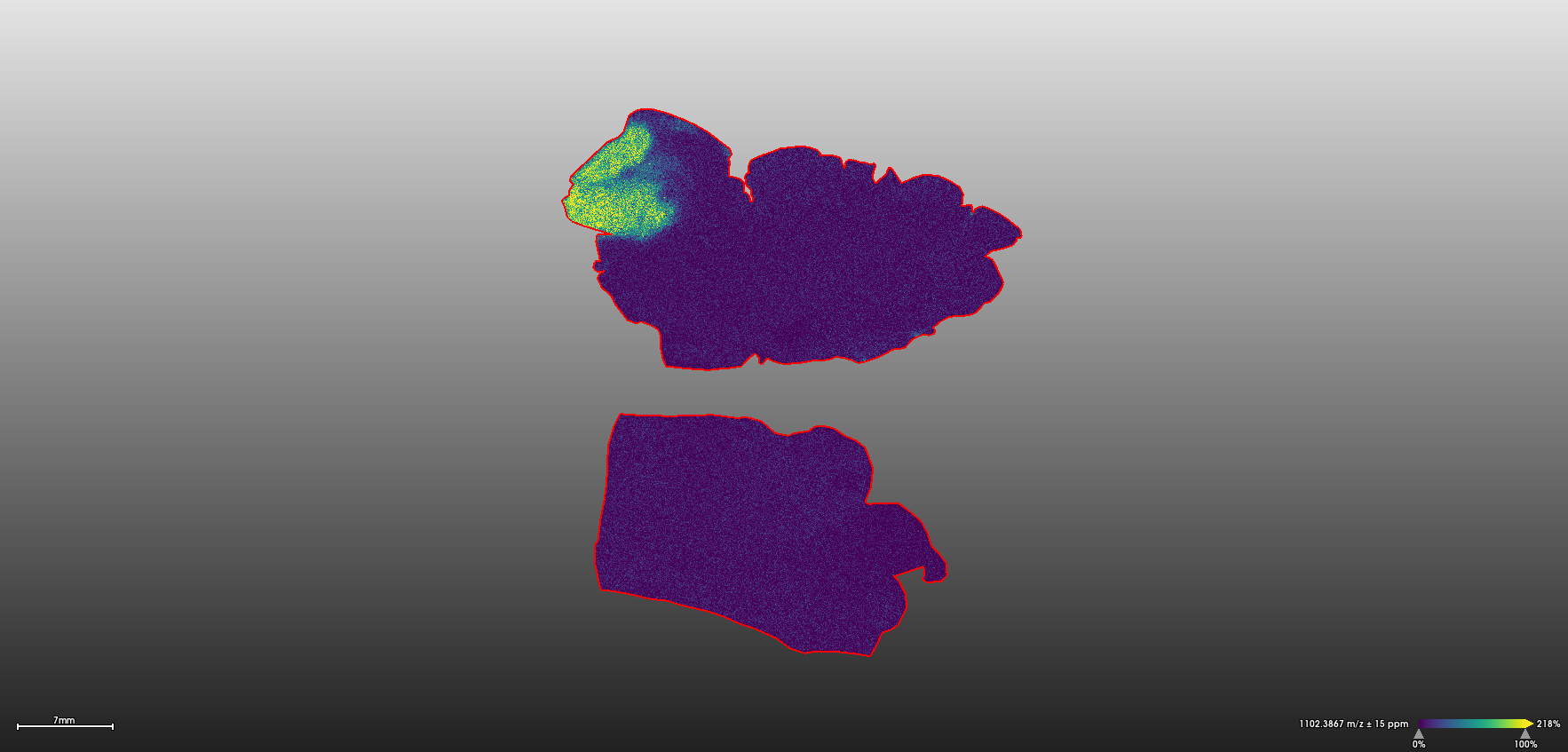

Supplement: Supplementary file 8 — Source Data 2 [file 41467_2026_72853_MOESM8_ESM.zip › Source Data MALDI Images/Supplementary Figure 32/20240614_1102_Eso_Image.png]

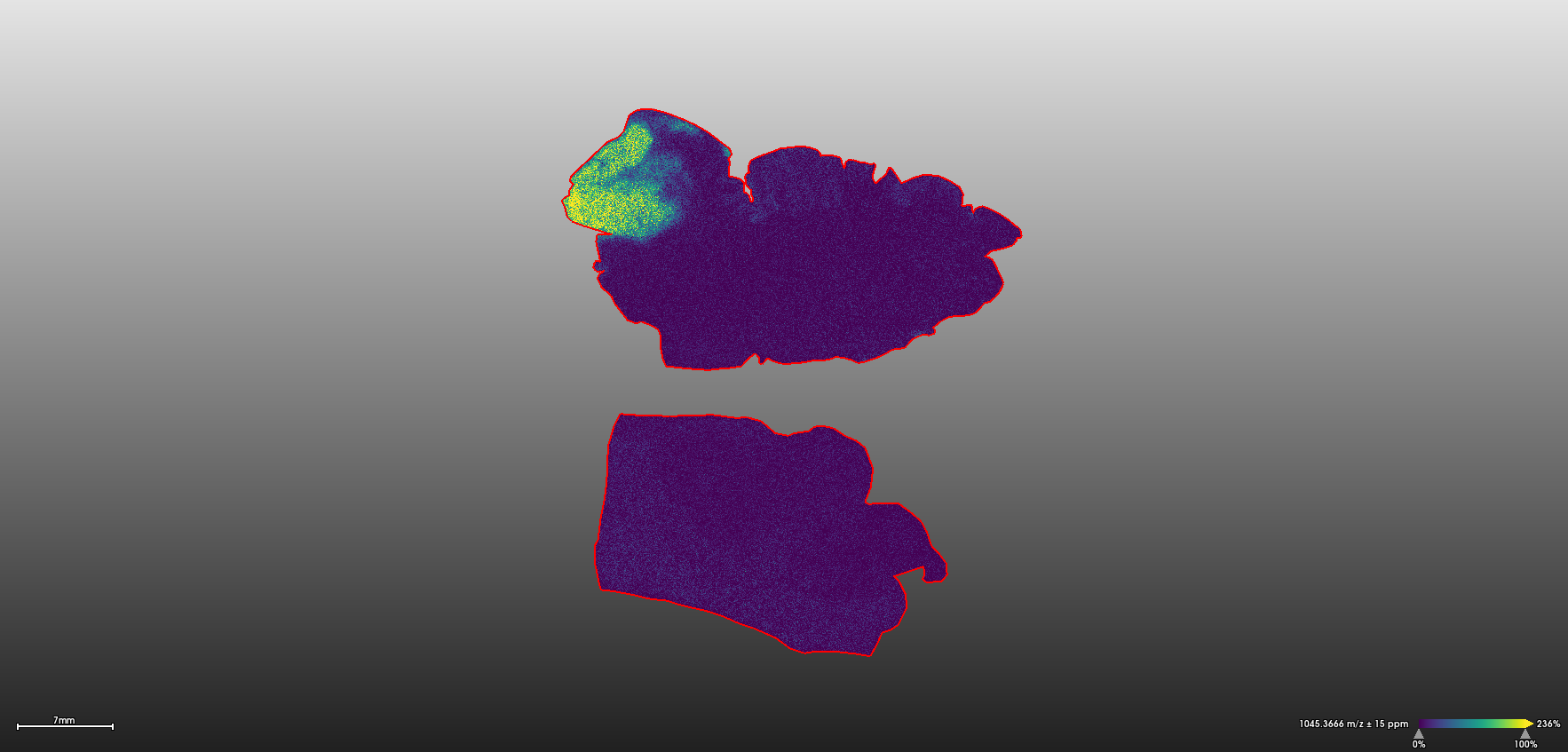

Supplement: Supplementary file 8 — Source Data 2 [file 41467_2026_72853_MOESM8_ESM.zip › Source Data MALDI Images/Supplementary Figure 32/20240614_1045_Eso_Image.png]

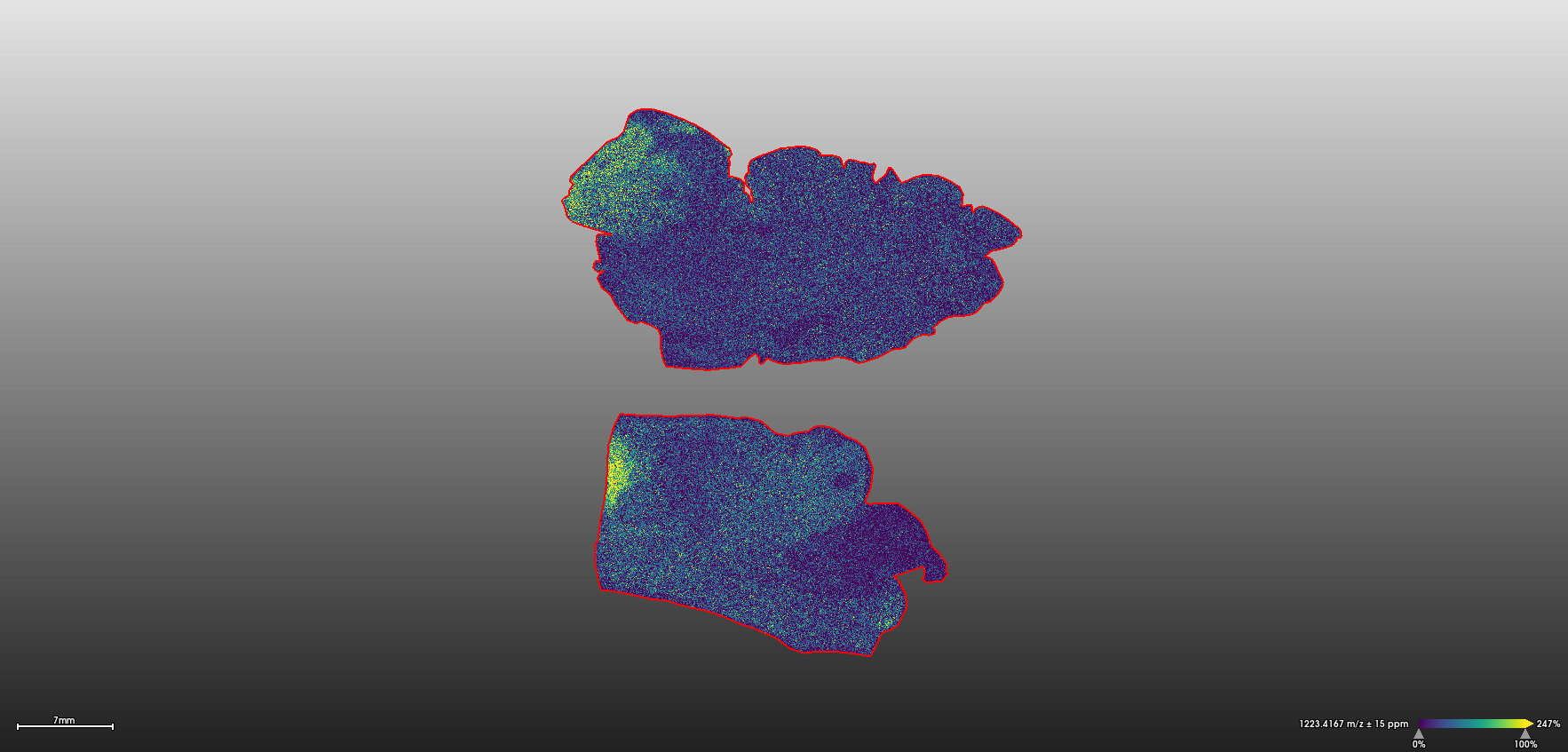

Supplement: Supplementary file 8 — Source Data 2 [file 41467_2026_72853_MOESM8_ESM.zip › Source Data MALDI Images/Supplementary Figure 32/20240614_1223_Eso_Image.png]

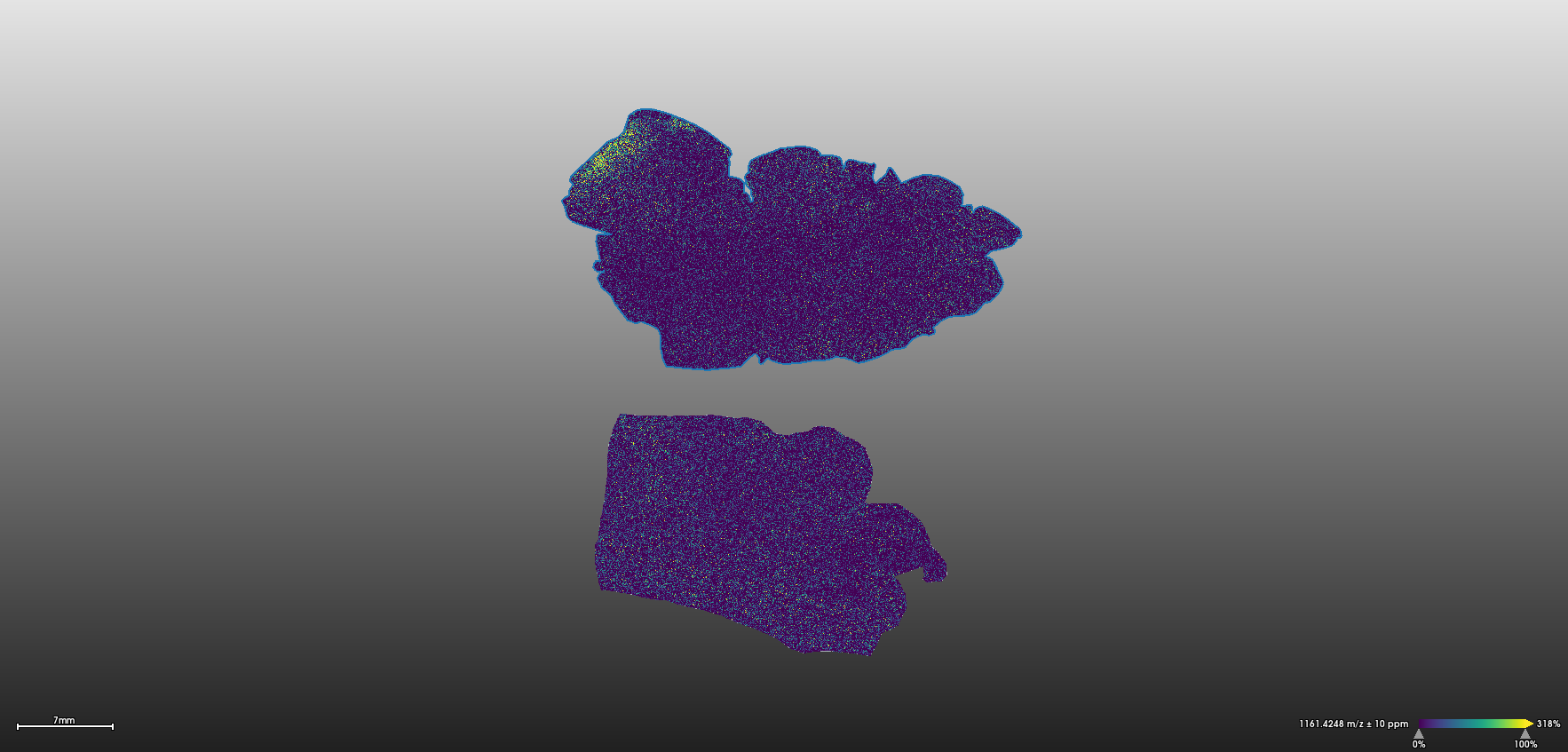

Supplement: Supplementary file 8 — Source Data 2 [file 41467_2026_72853_MOESM8_ESM.zip › Source Data MALDI Images/Supplementary Figure 32/20240627_1161_Eso_Image.png]

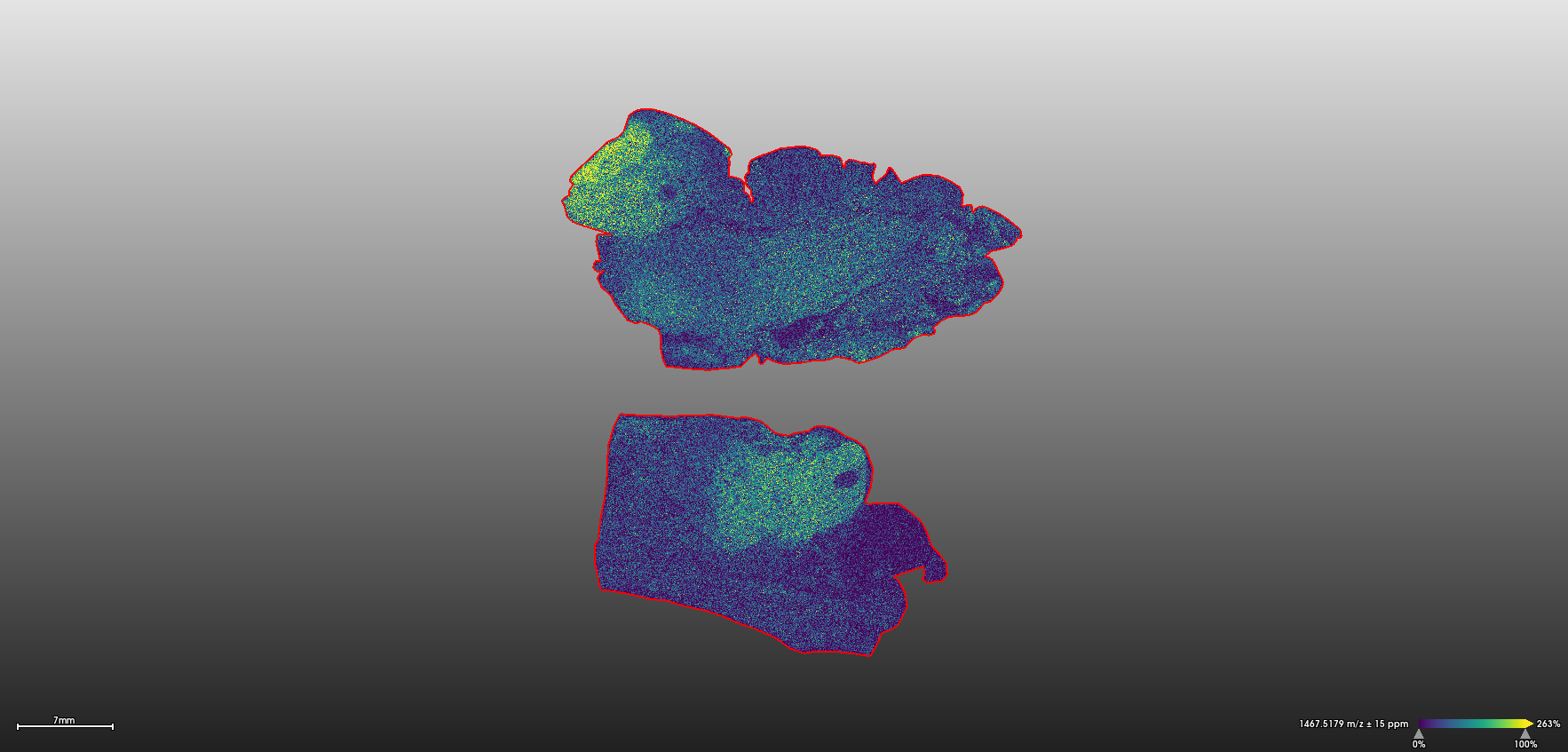

Supplement: Supplementary file 8 — Source Data 2 [file 41467_2026_72853_MOESM8_ESM.zip › Source Data MALDI Images/Supplementary Figure 32/20240614_1467_Eso_Image.png]

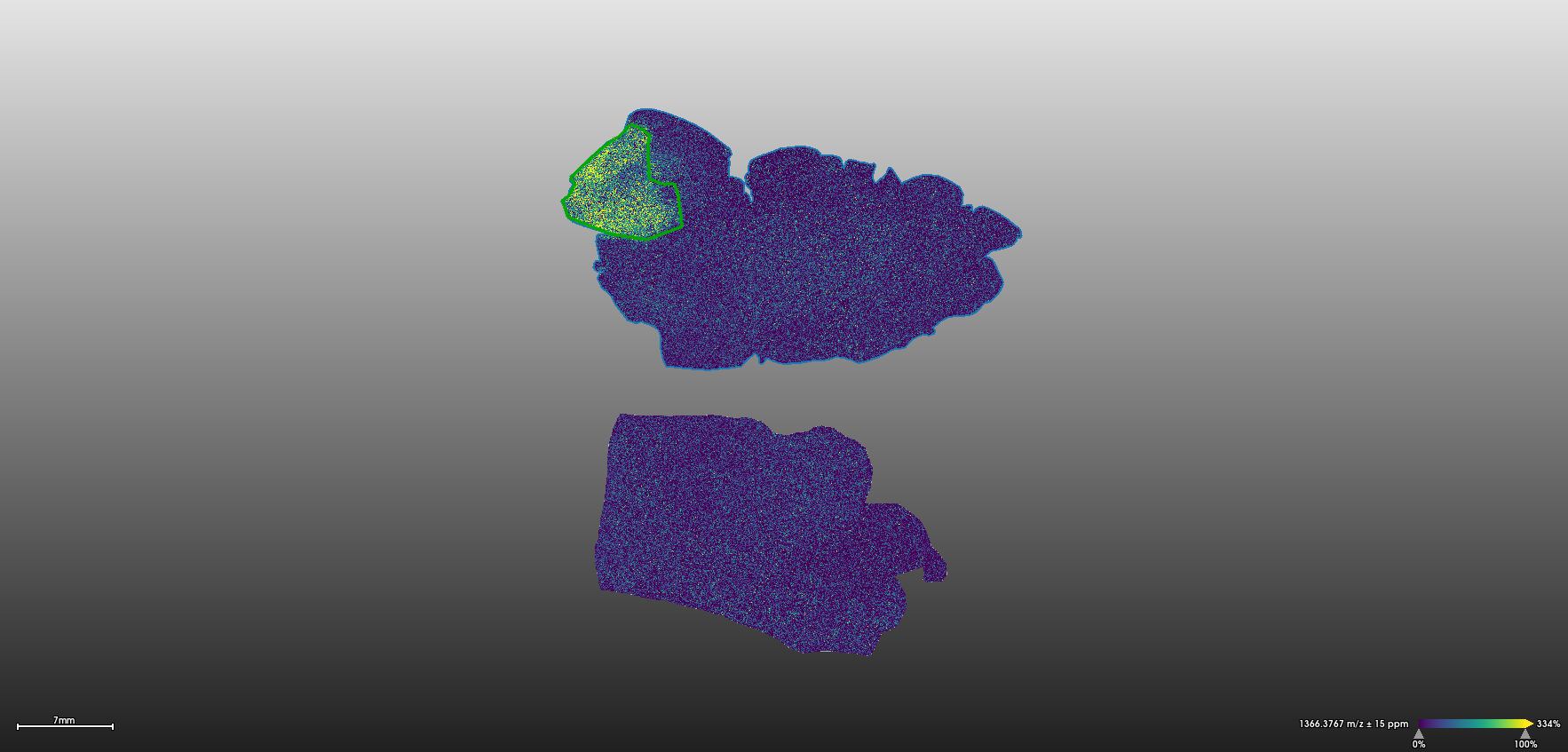

Supplement: Supplementary file 8 — Source Data 2 [file 41467_2026_72853_MOESM8_ESM.zip › Source Data MALDI Images/Supplementary Figure 32/20240627_1366_Eso_Image.png]

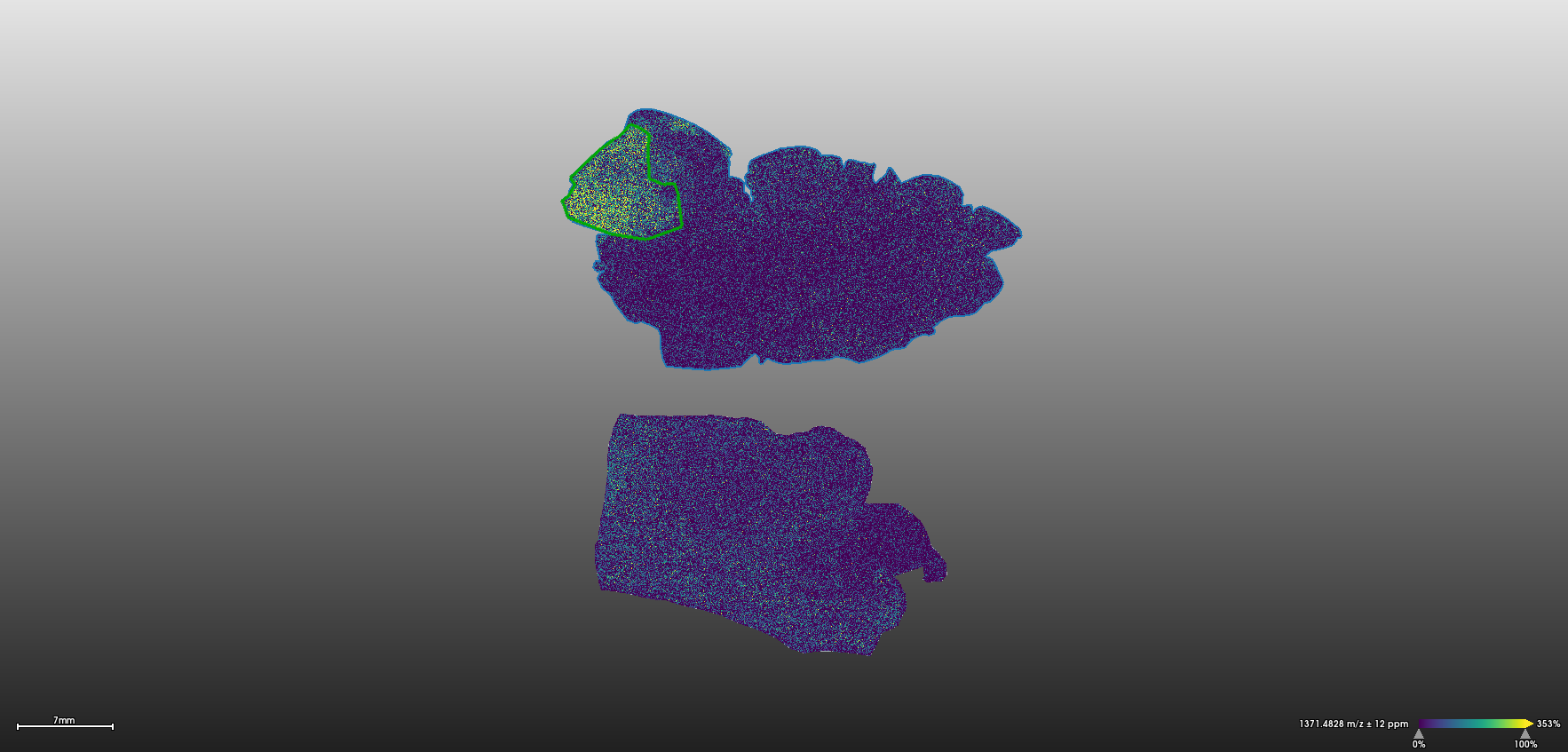

Supplement: Supplementary file 8 — Source Data 2 [file 41467_2026_72853_MOESM8_ESM.zip › Source Data MALDI Images/Supplementary Figure 32/20240627_1371_Eso_Image.png]

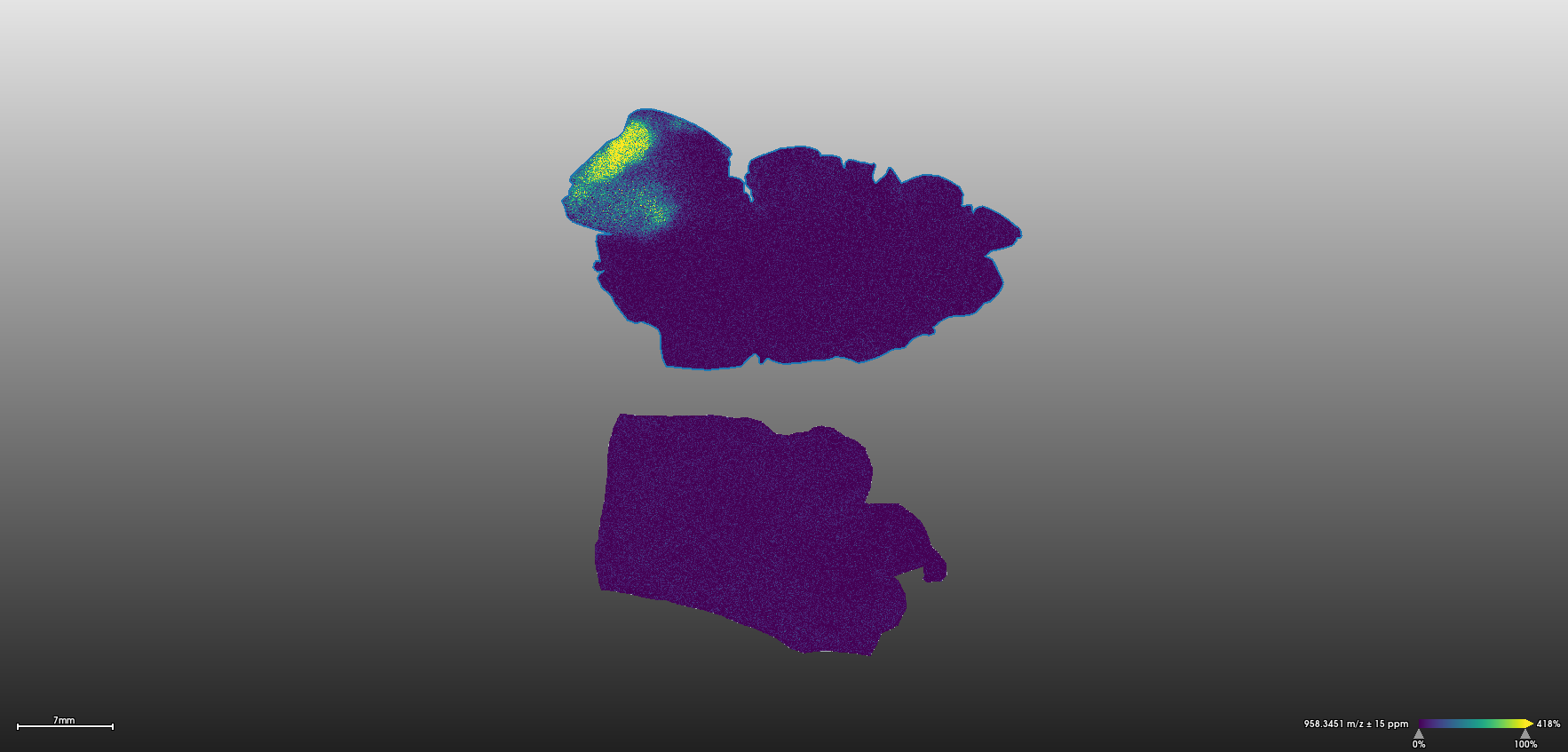

Supplement: Supplementary file 8 — Source Data 2 [file 41467_2026_72853_MOESM8_ESM.zip › Source Data MALDI Images/Supplementary Figure 32/20240627_958_Eso_Image.png]

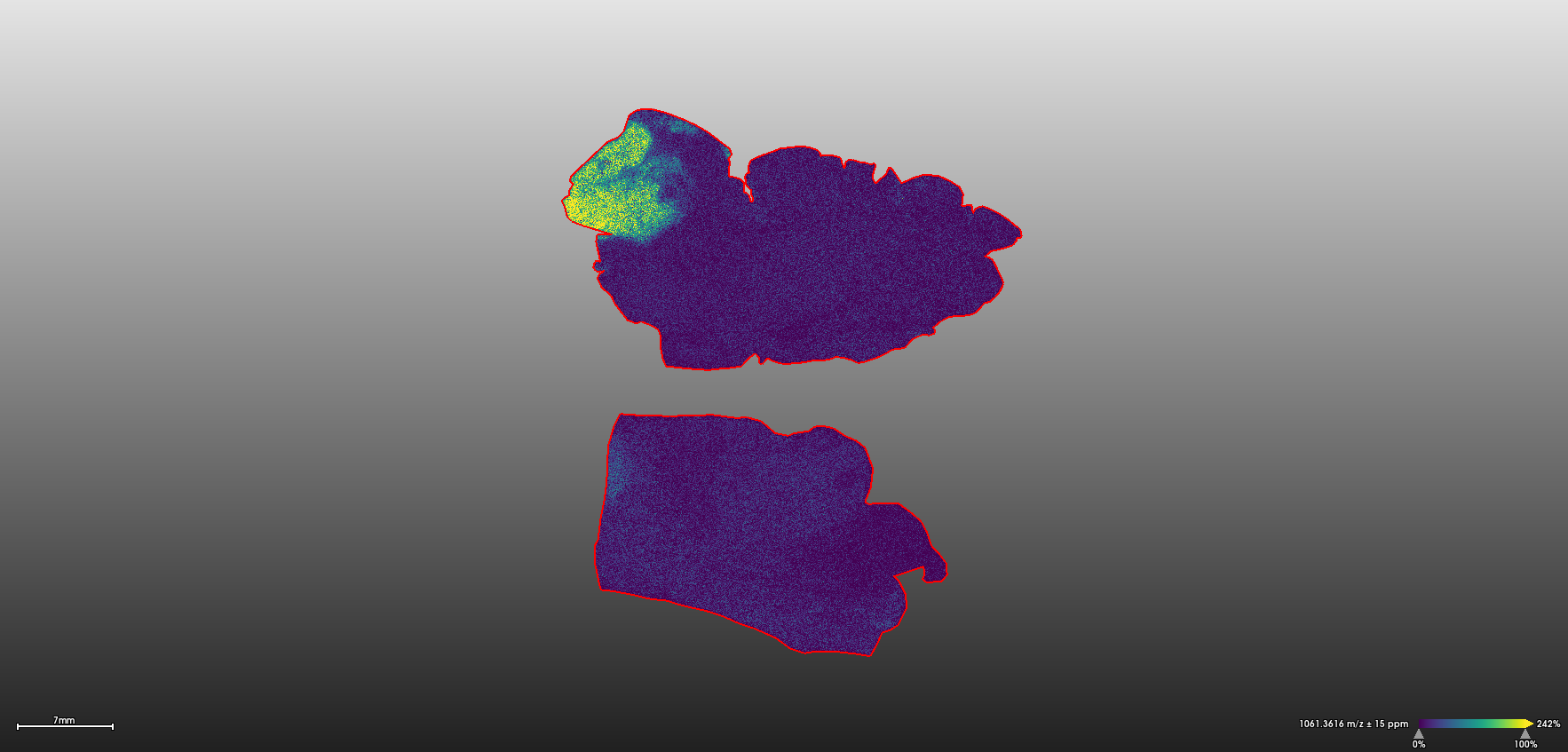

Supplement: Supplementary file 8 — Source Data 2 [file 41467_2026_72853_MOESM8_ESM.zip › Source Data MALDI Images/Supplementary Figure 32/20240531_1061_Eso_Image.png]

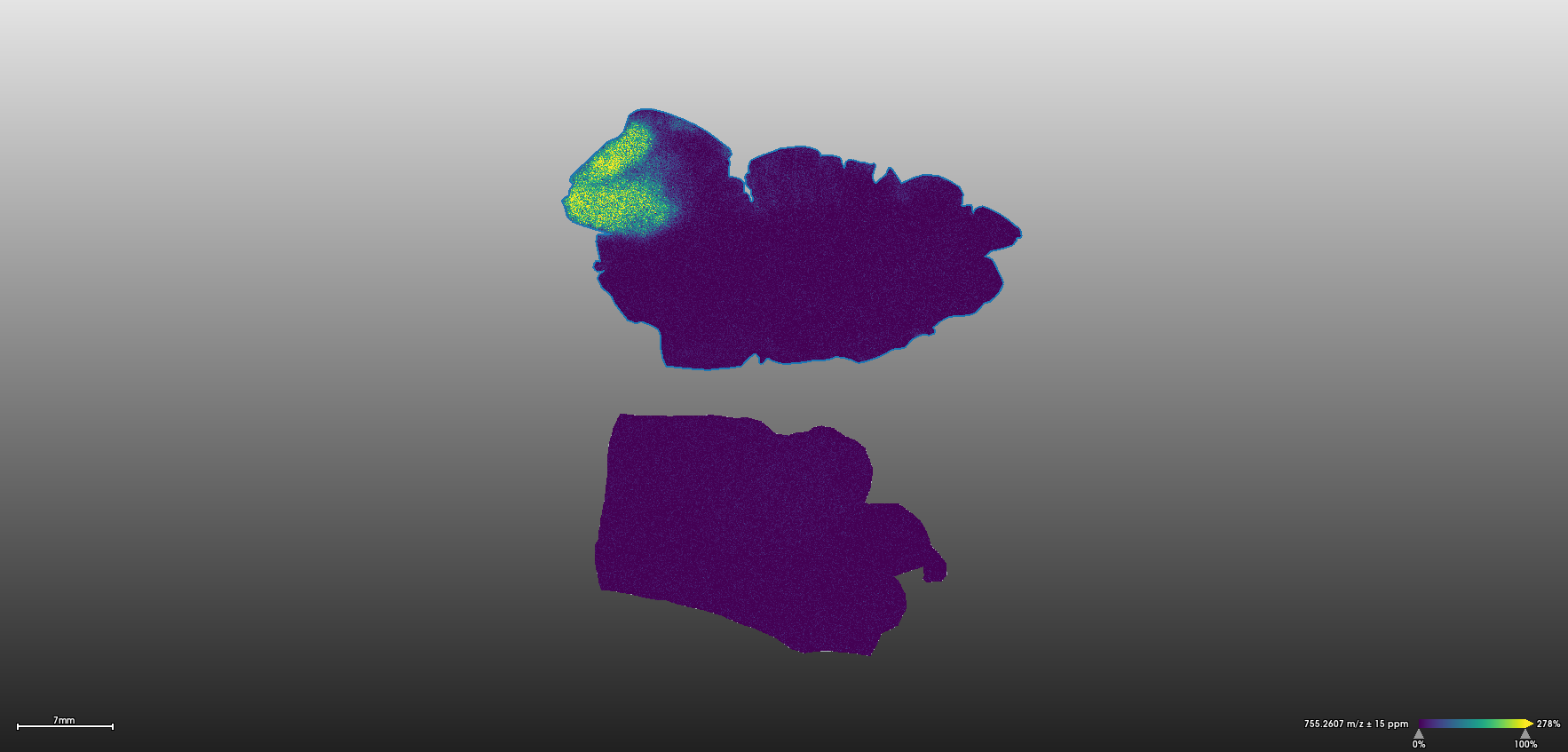

Supplement: Supplementary file 8 — Source Data 2 [file 41467_2026_72853_MOESM8_ESM.zip › Source Data MALDI Images/Supplementary Figure 32/20240627_755_Eso_Image.png]

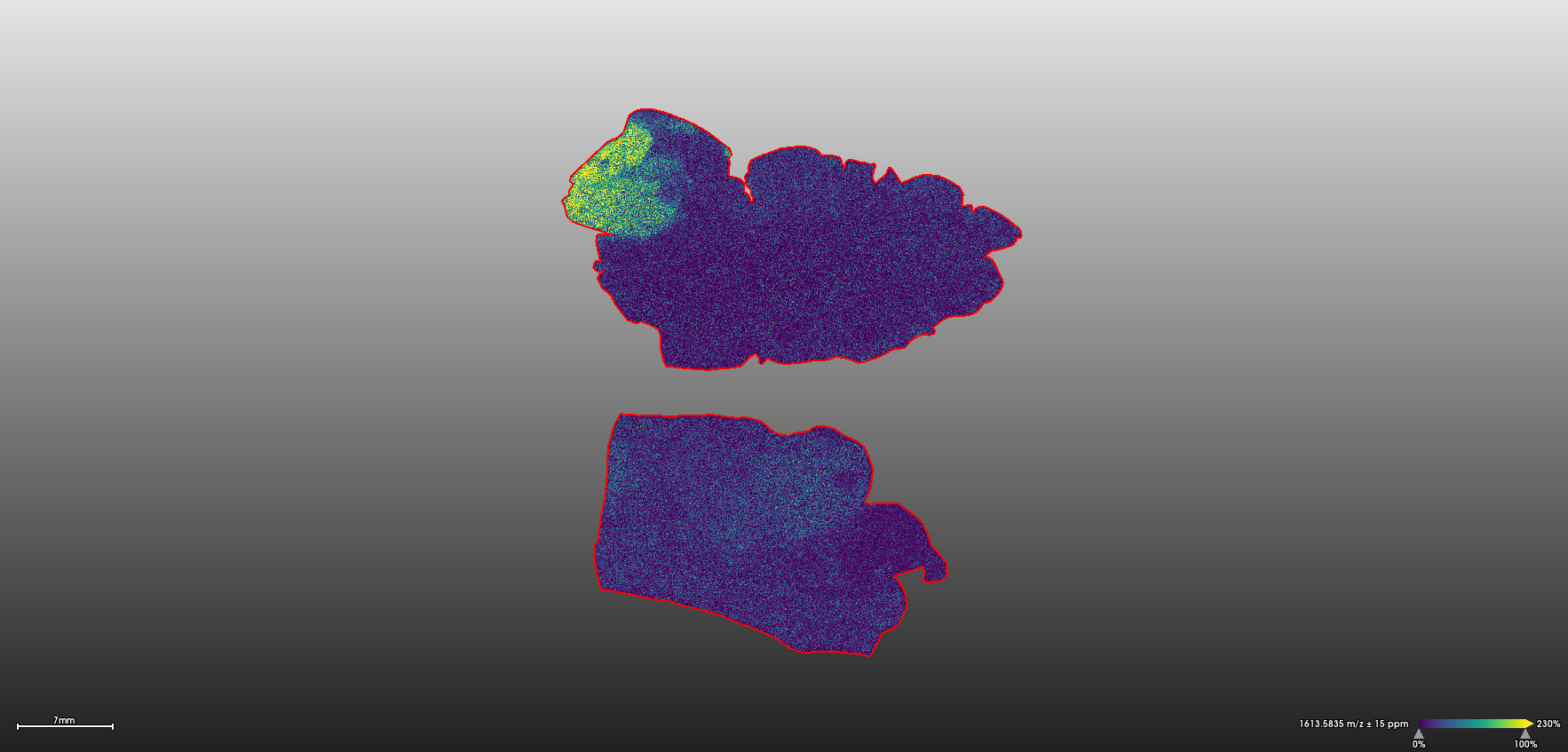

Supplement: Supplementary file 8 — Source Data 2 [file 41467_2026_72853_MOESM8_ESM.zip › Source Data MALDI Images/Supplementary Figure 32/20240614_1613_Eso_Image.png]

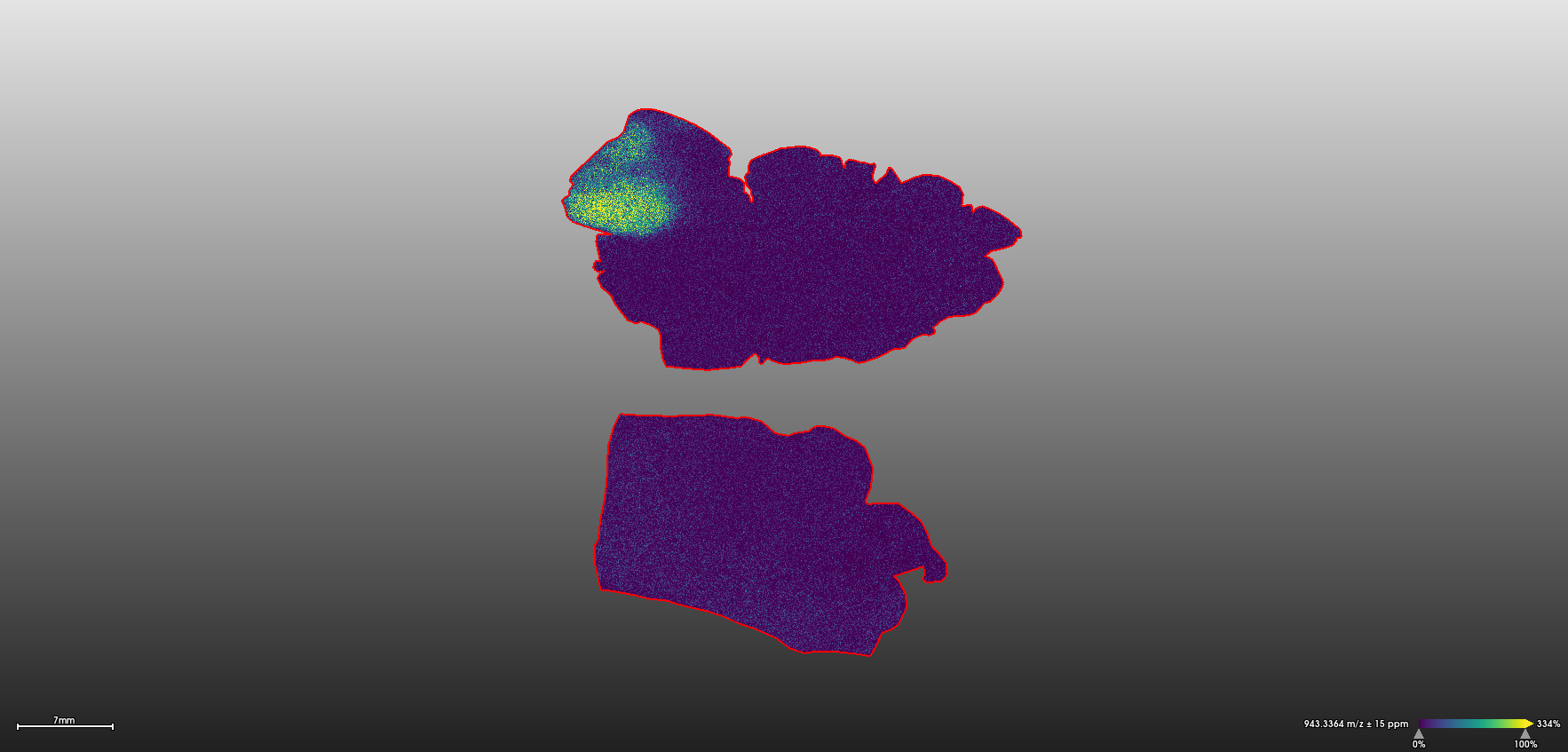

Supplement: Supplementary file 8 — Source Data 2 [file 41467_2026_72853_MOESM8_ESM.zip › Source Data MALDI Images/Supplementary Figure 32/20240531_943_Eso_Image.png]

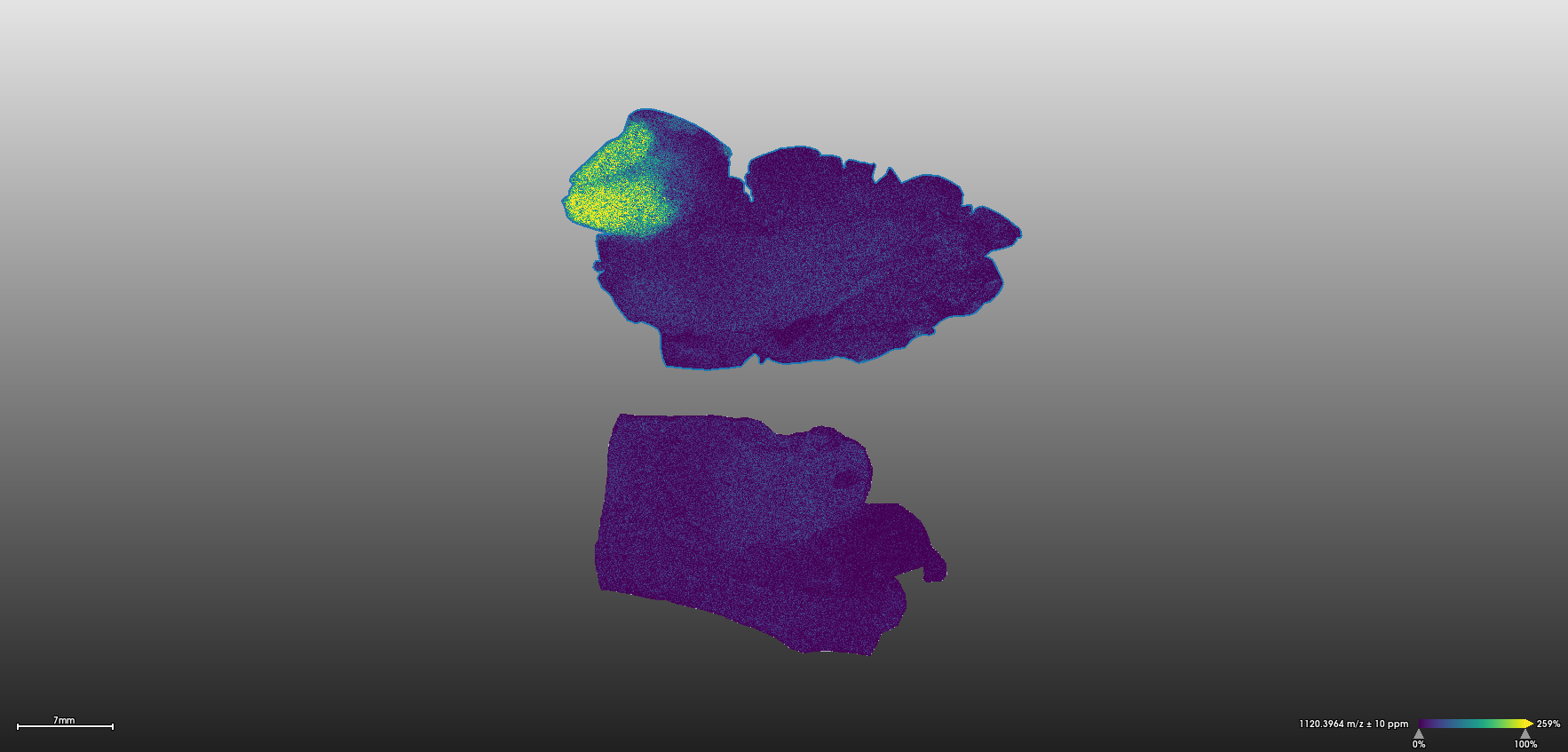

Supplement: Supplementary file 8 — Source Data 2 [file 41467_2026_72853_MOESM8_ESM.zip › Source Data MALDI Images/Supplementary Figure 32/20240627_1120_Eso_Image.png]

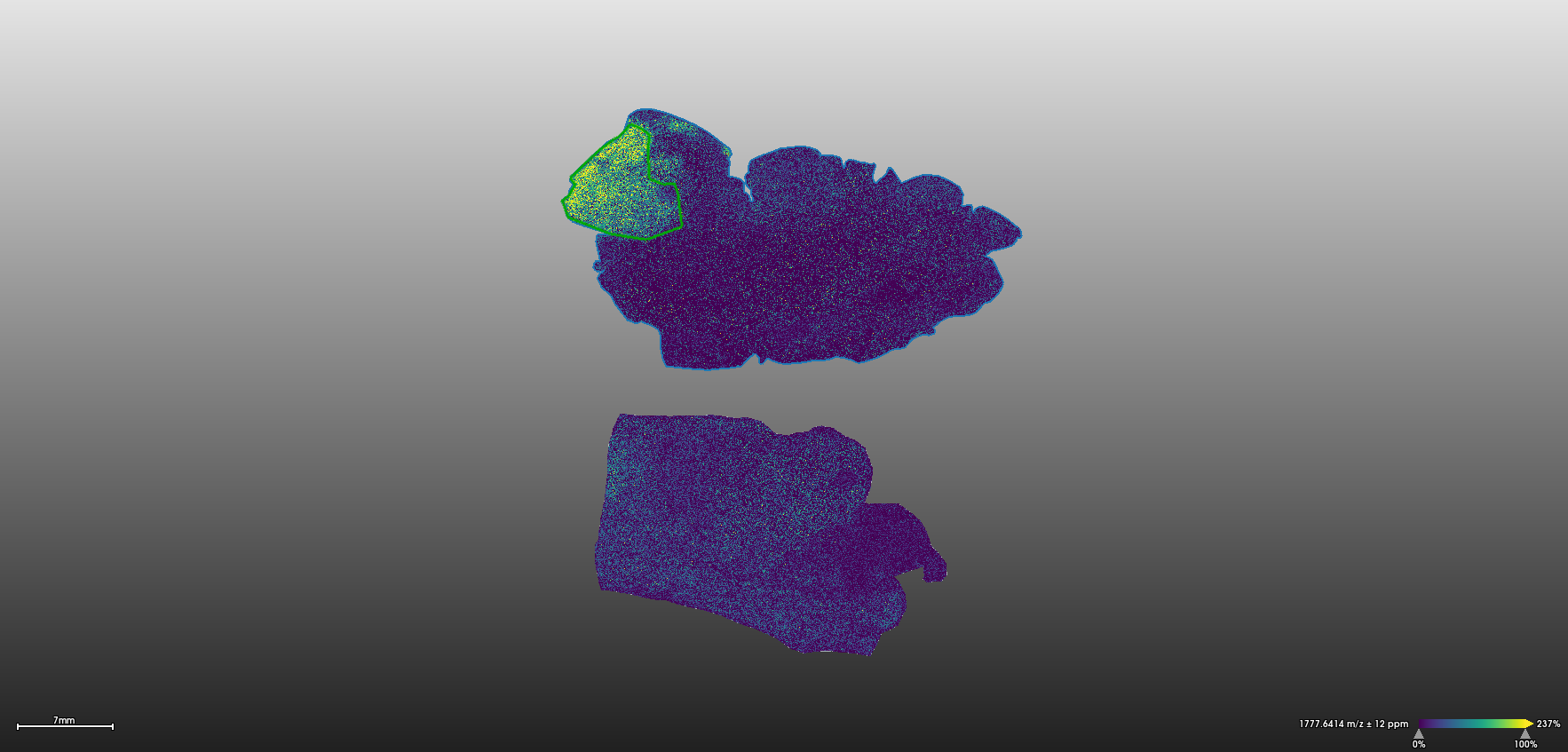

Supplement: Supplementary file 8 — Source Data 2 [file 41467_2026_72853_MOESM8_ESM.zip › Source Data MALDI Images/Supplementary Figure 32/20240627_1777_Eso_Image.png]

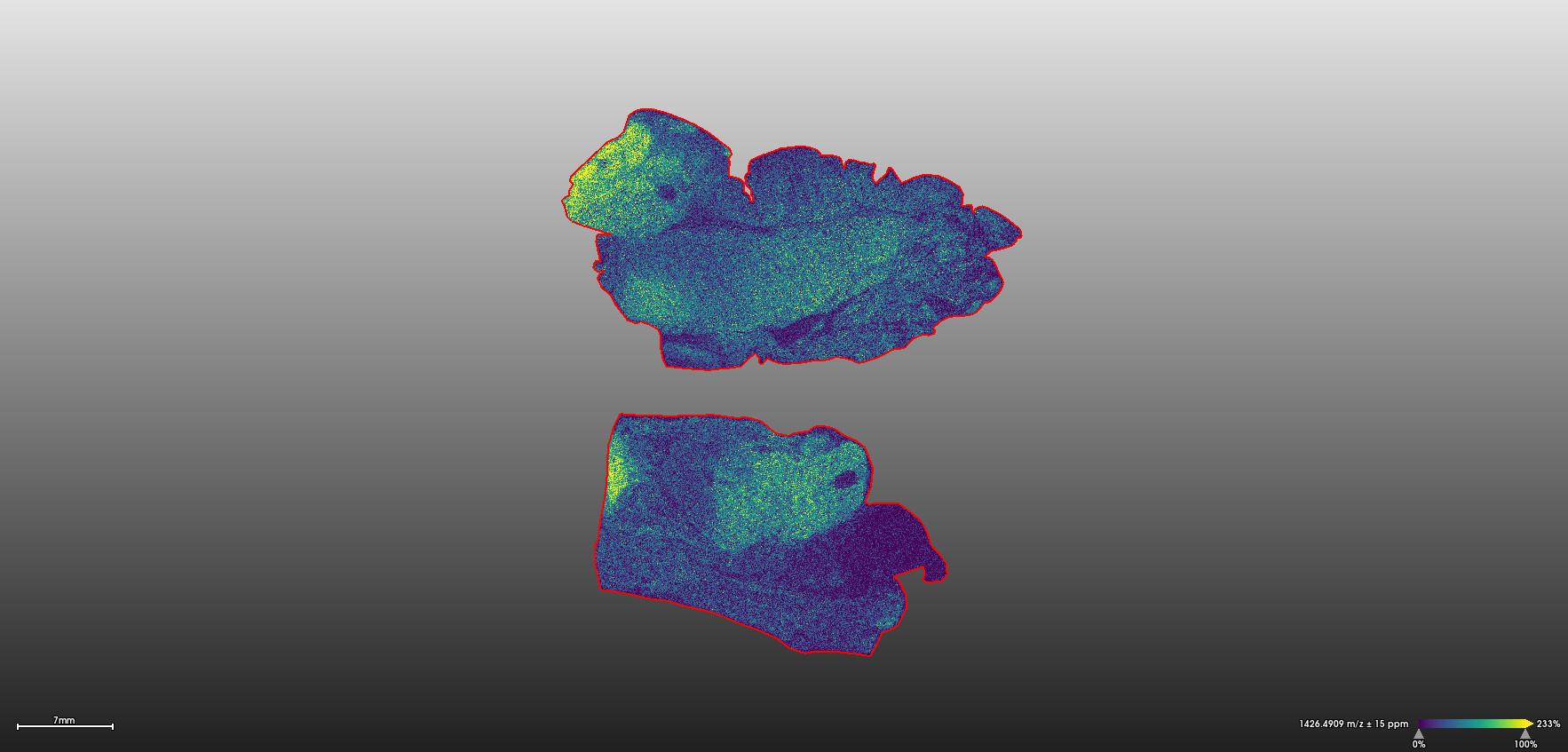

Supplement: Supplementary file 8 — Source Data 2 [file 41467_2026_72853_MOESM8_ESM.zip › Source Data MALDI Images/Supplementary Figure 32/20240614_1426_Eso_Image.png]

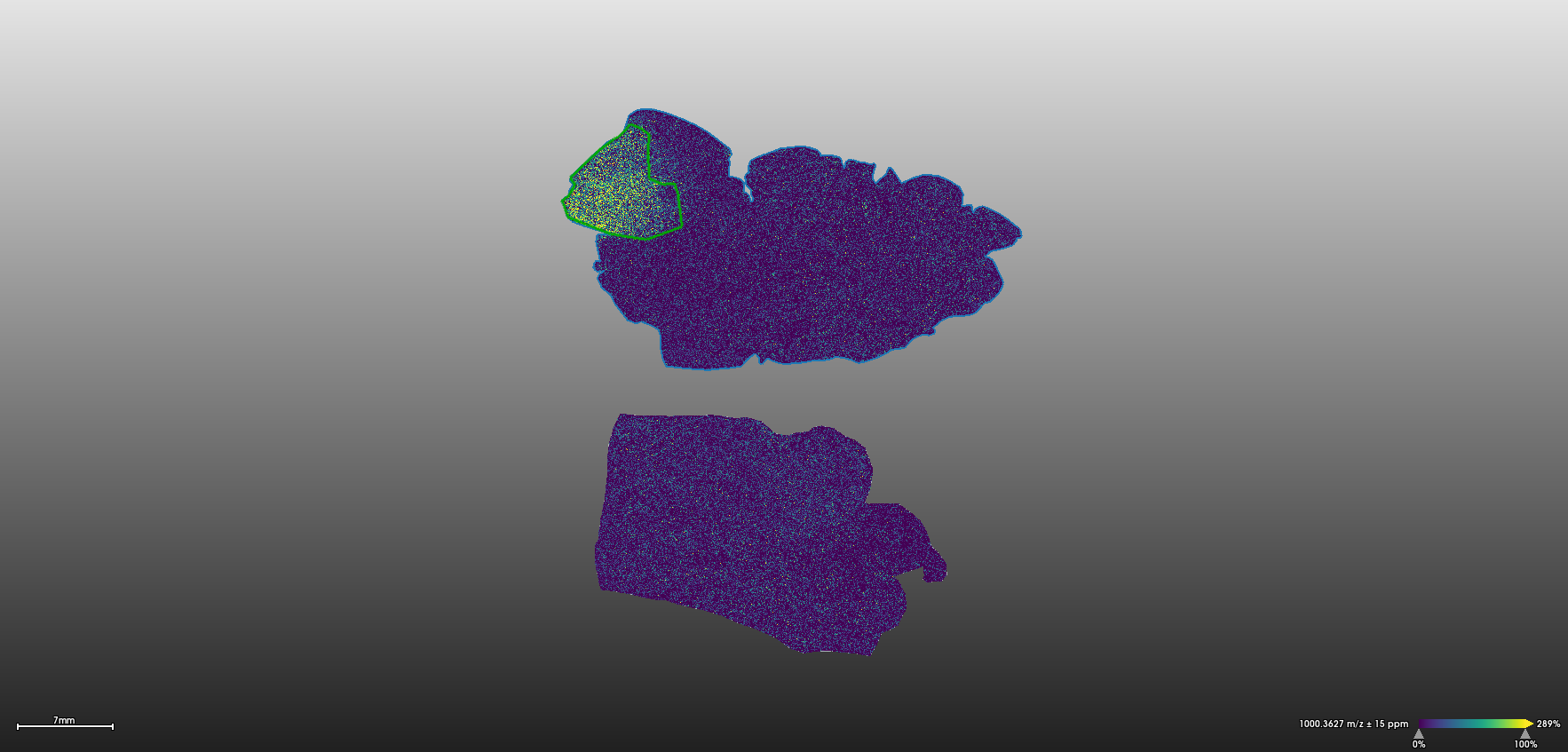

Supplement: Supplementary file 8 — Source Data 2 [file 41467_2026_72853_MOESM8_ESM.zip › Source Data MALDI Images/Supplementary Figure 32/20240627_1000_Eso_Image.png]

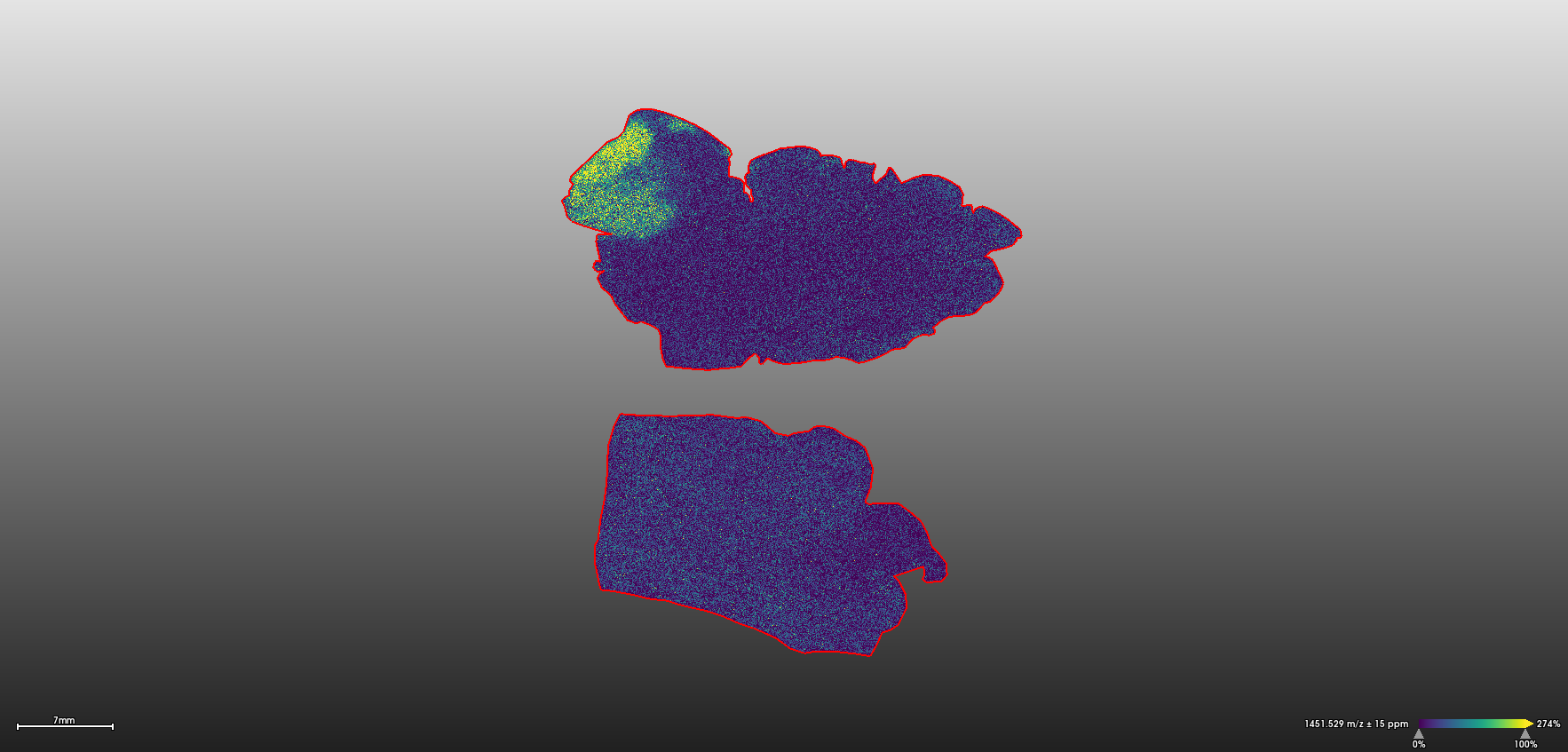

Supplement: Supplementary file 8 — Source Data 2 [file 41467_2026_72853_MOESM8_ESM.zip › Source Data MALDI Images/Supplementary Figure 32/20240614_1451_Eso_Image.png]

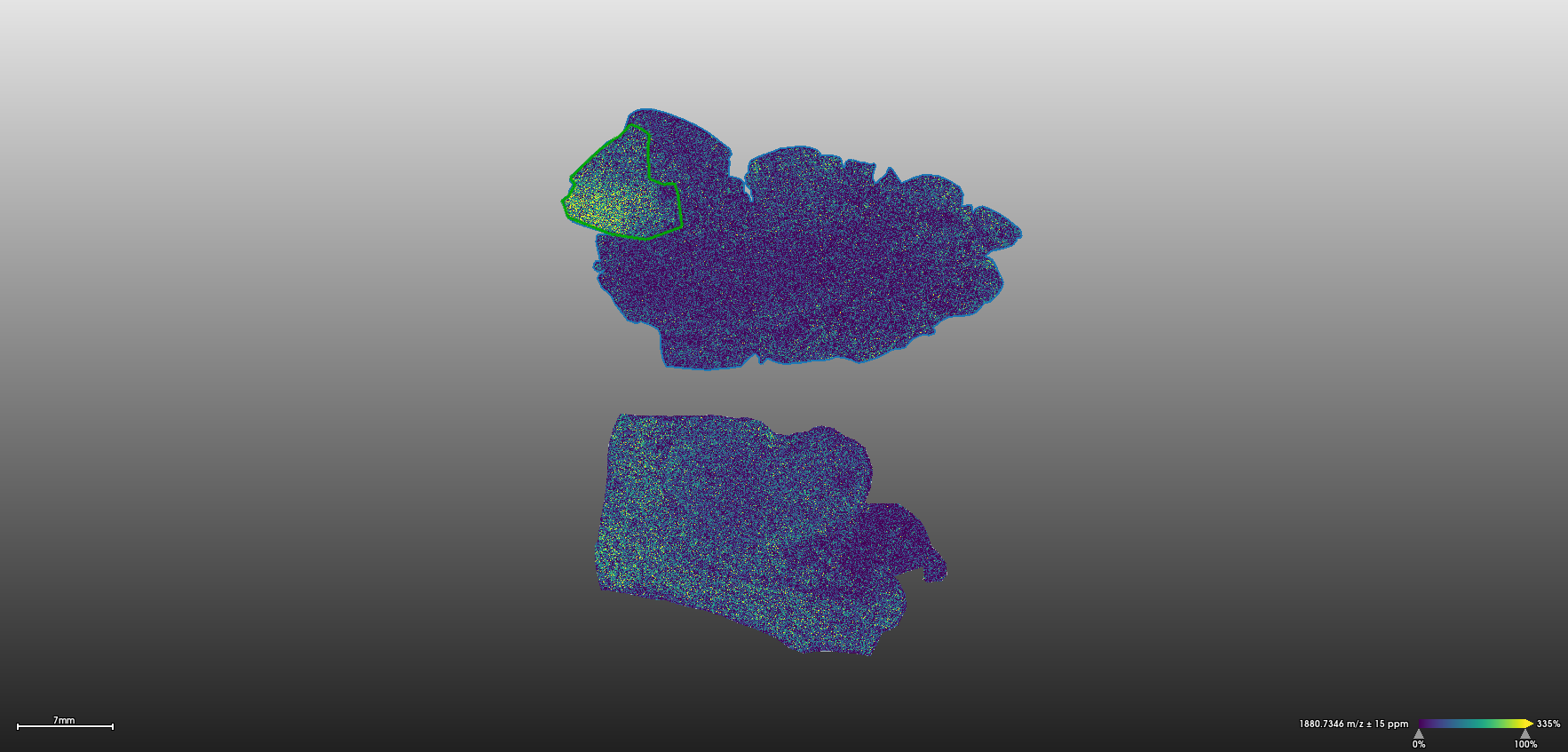

Supplement: Supplementary file 8 — Source Data 2 [file 41467_2026_72853_MOESM8_ESM.zip › Source Data MALDI Images/Supplementary Figure 32/20240627_1880_Eso_Image.png]

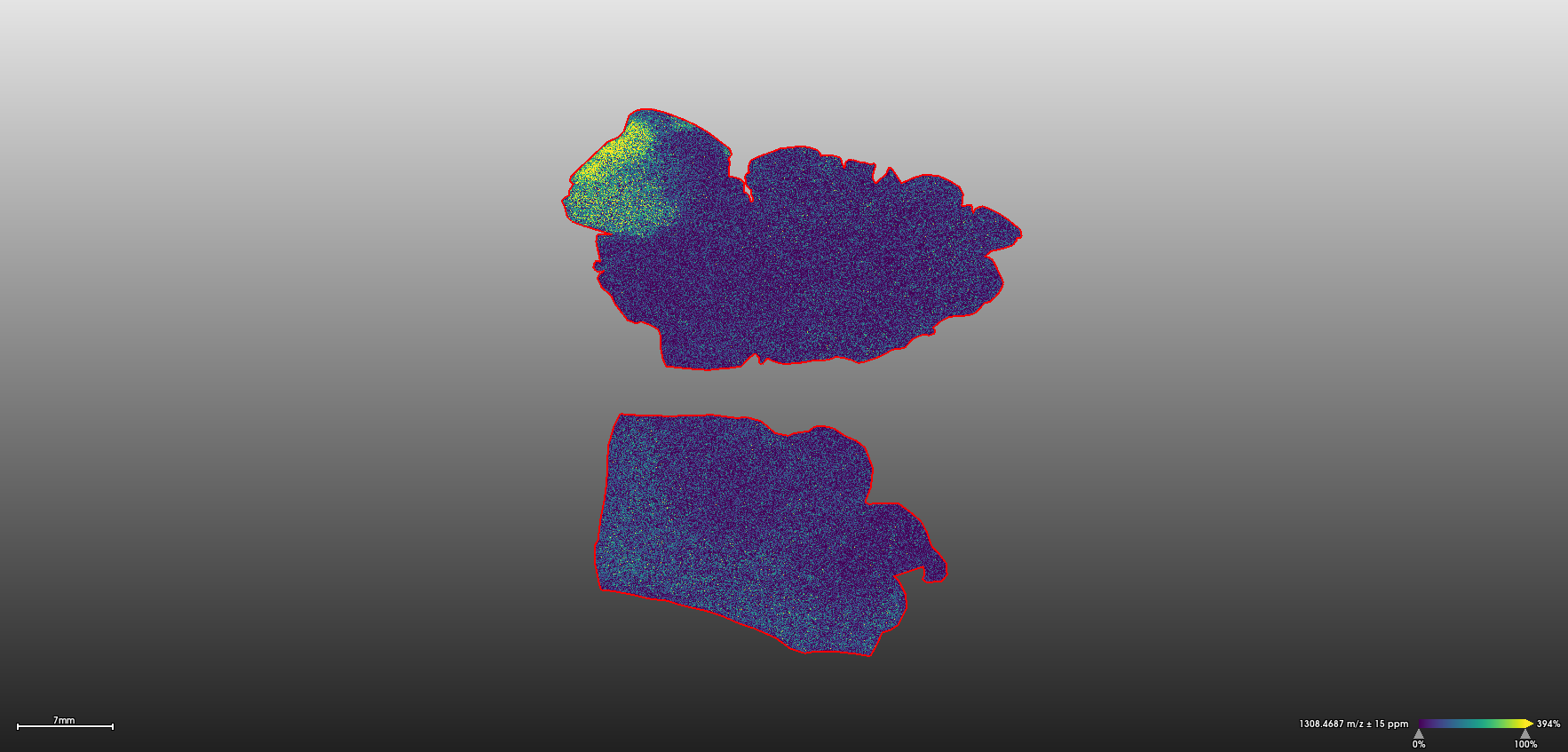

Supplement: Supplementary file 8 — Source Data 2 [file 41467_2026_72853_MOESM8_ESM.zip › Source Data MALDI Images/Supplementary Figure 32/20240614_1308_Eso_Image.png]

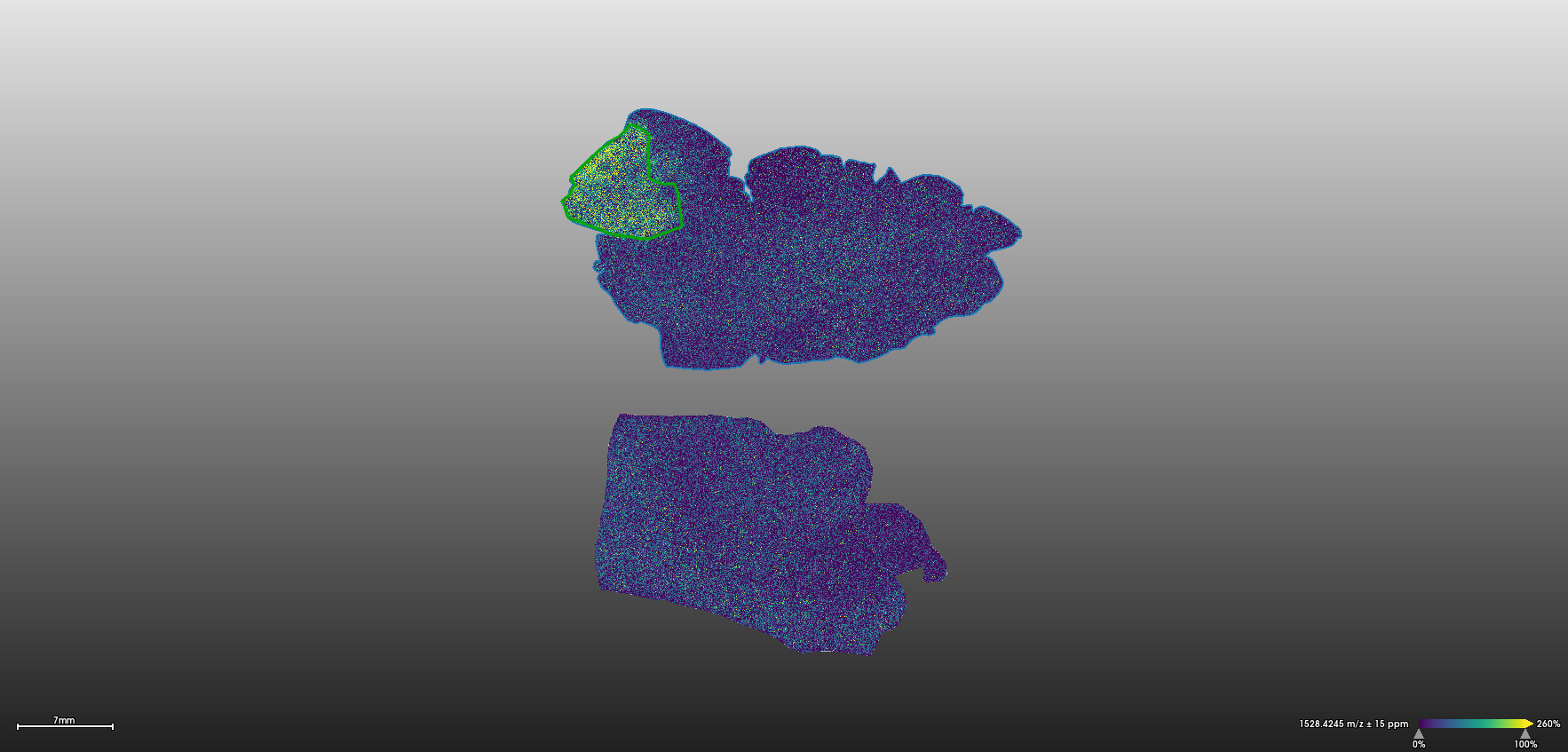

Supplement: Supplementary file 8 — Source Data 2 [file 41467_2026_72853_MOESM8_ESM.zip › Source Data MALDI Images/Supplementary Figure 32/20240627_1528_Eso_Image.png]

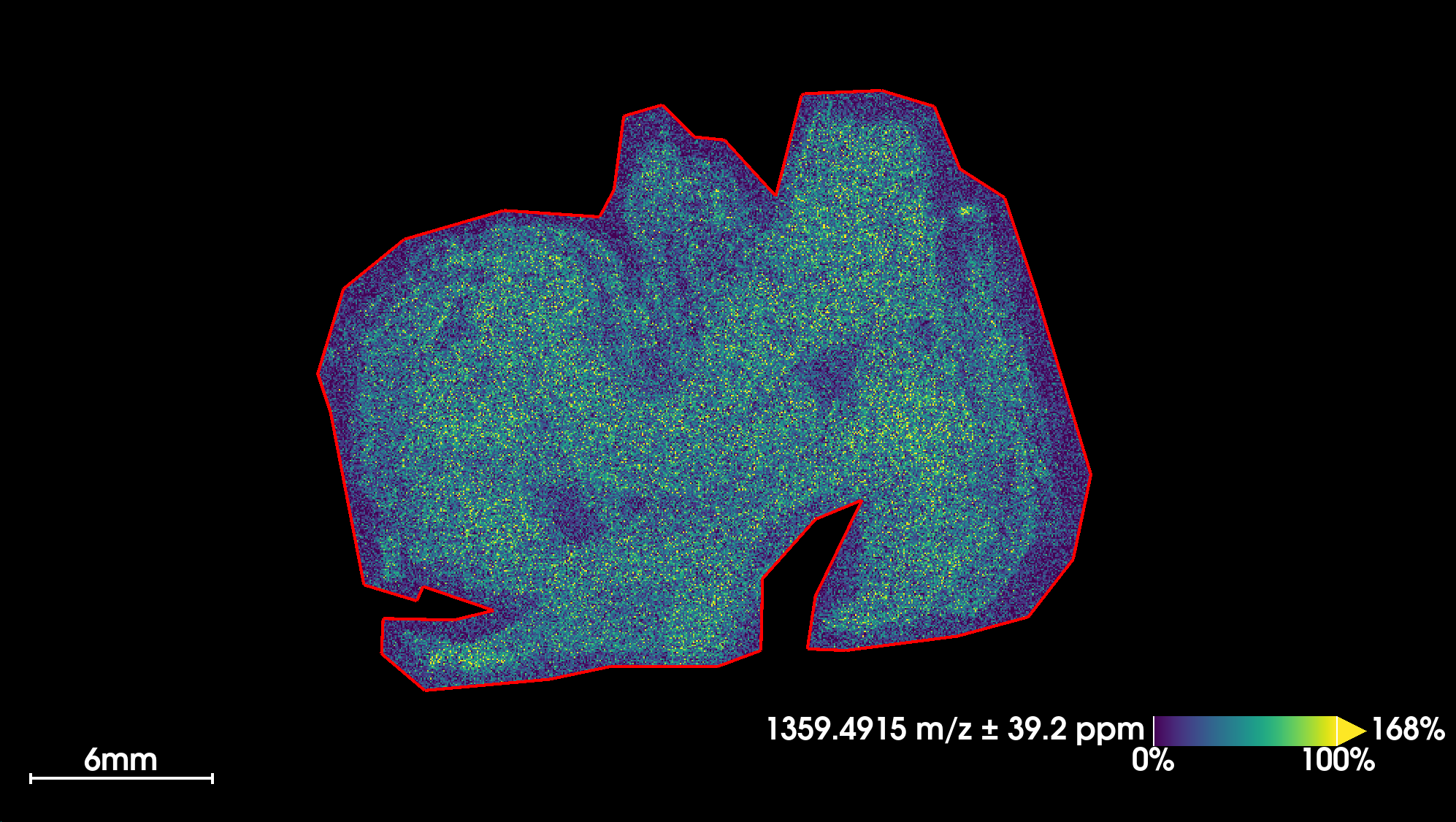

Supplement: Supplementary file 8 — Source Data 2 [file 41467_2026_72853_MOESM8_ESM.zip › Source Data MALDI Images/Supplementary Figure 8/1359.4915 mz ┬▒ 53.2 mDa.png]

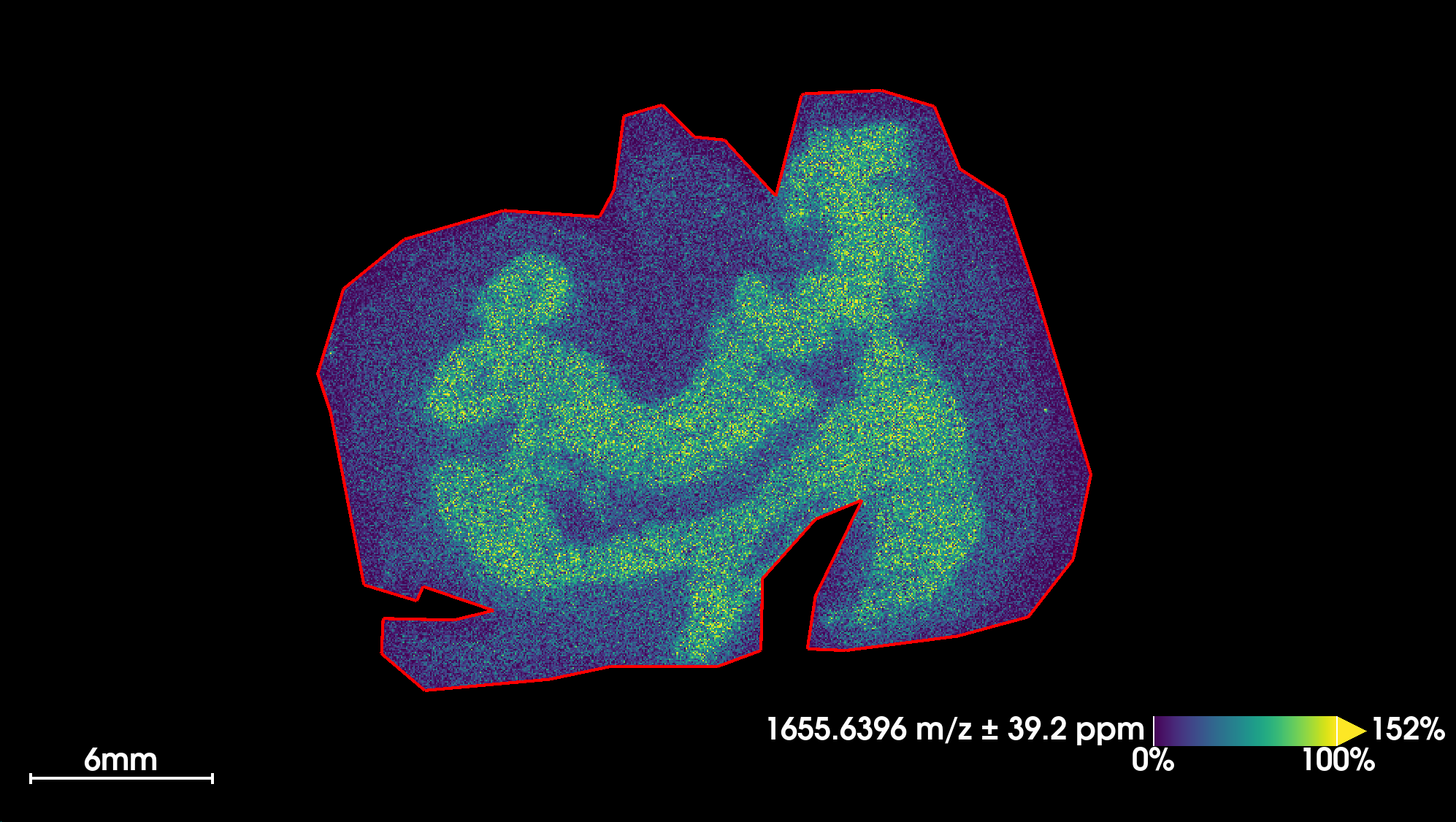

Supplement: Supplementary file 8 — Source Data 2 [file 41467_2026_72853_MOESM8_ESM.zip › Source Data MALDI Images/Supplementary Figure 8/1655.6396 mz ┬▒ 64.8 mDa.png]

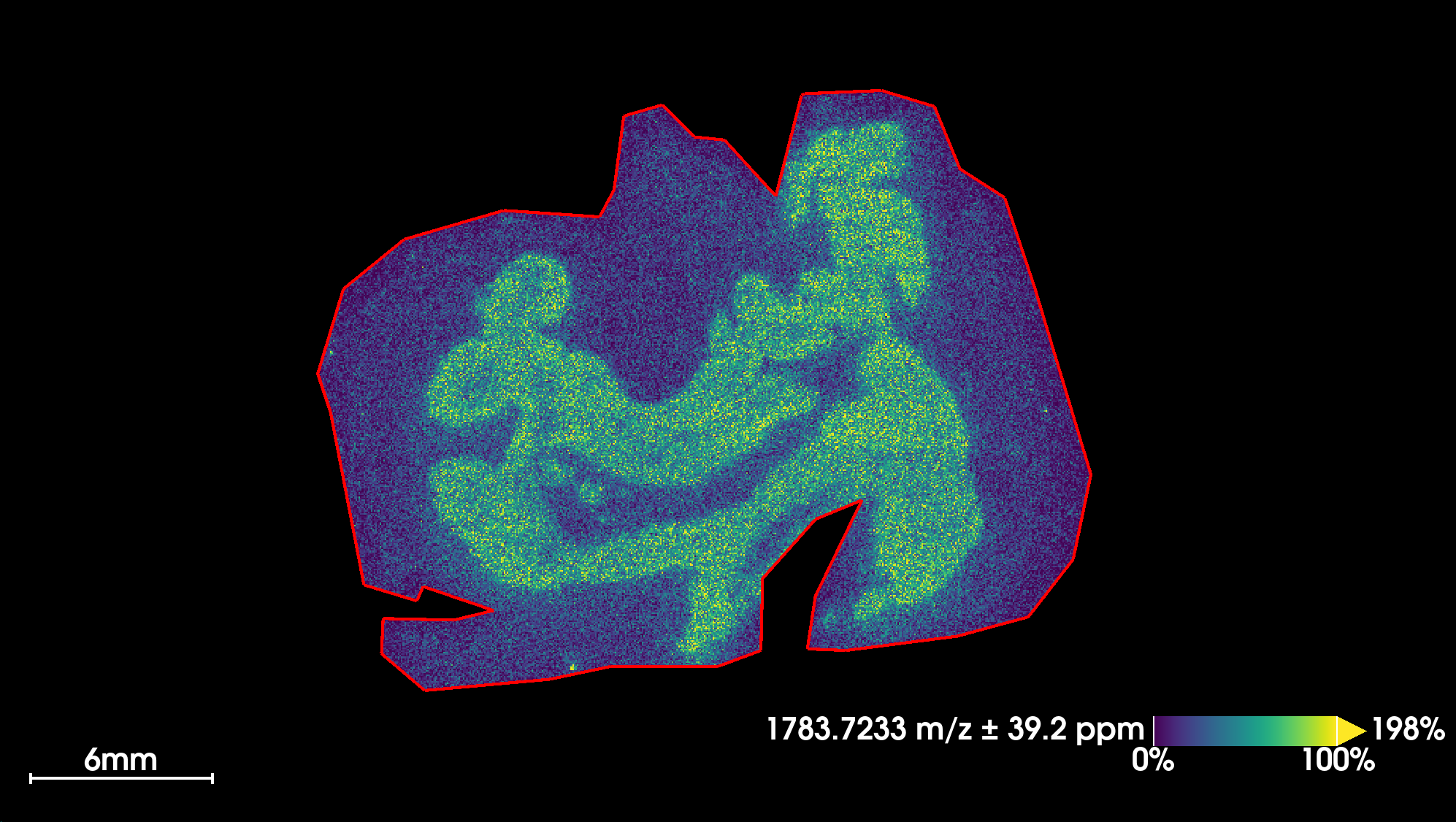

Supplement: Supplementary file 8 — Source Data 2 [file 41467_2026_72853_MOESM8_ESM.zip › Source Data MALDI Images/Supplementary Figure 8/1783.7233 mz ┬▒ 69.8 mDa.png]

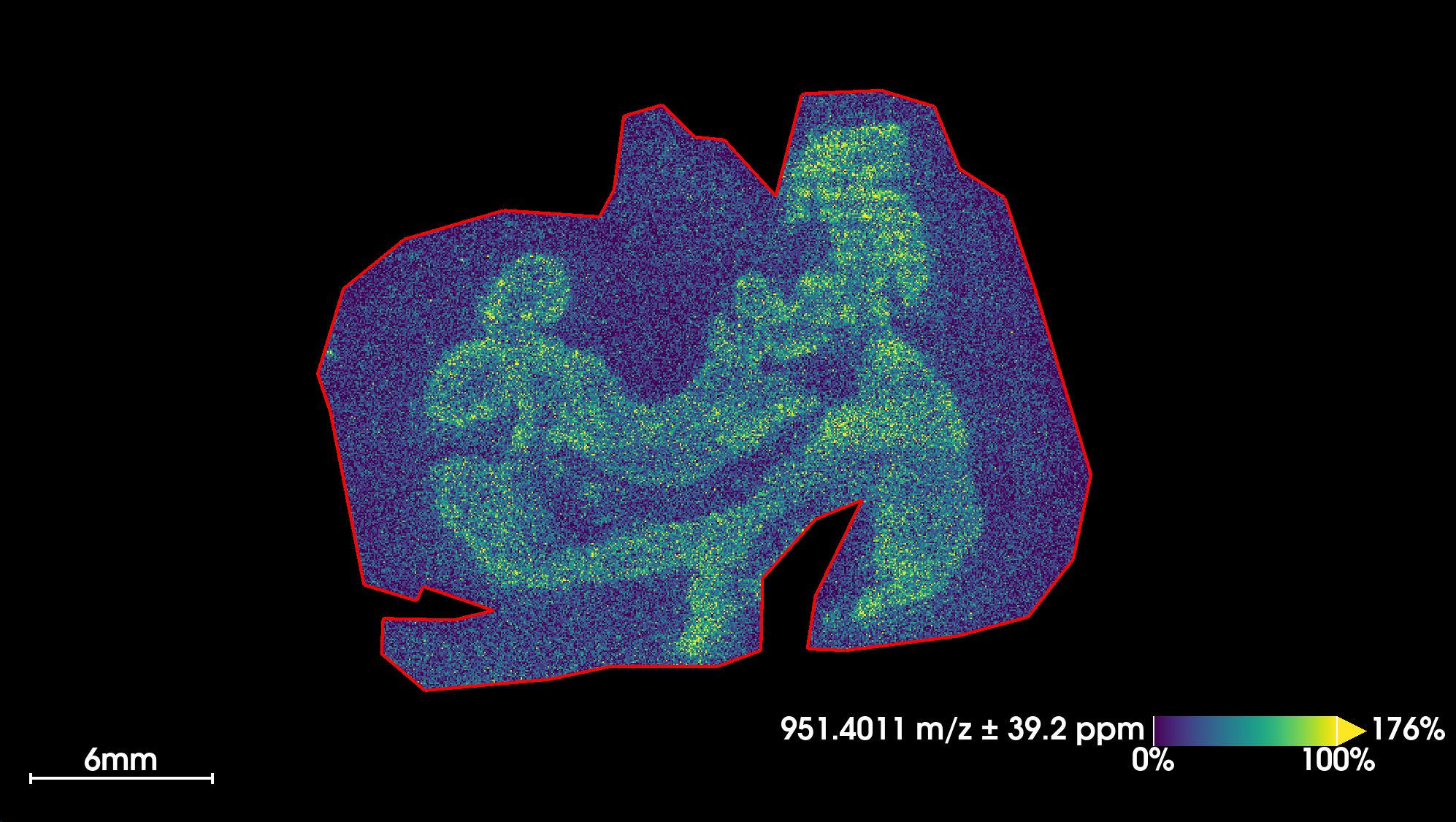

Supplement: Supplementary file 8 — Source Data 2 [file 41467_2026_72853_MOESM8_ESM.zip › Source Data MALDI Images/Supplementary Figure 8/951.4011 mz ┬▒ 37.3 mDa.png]

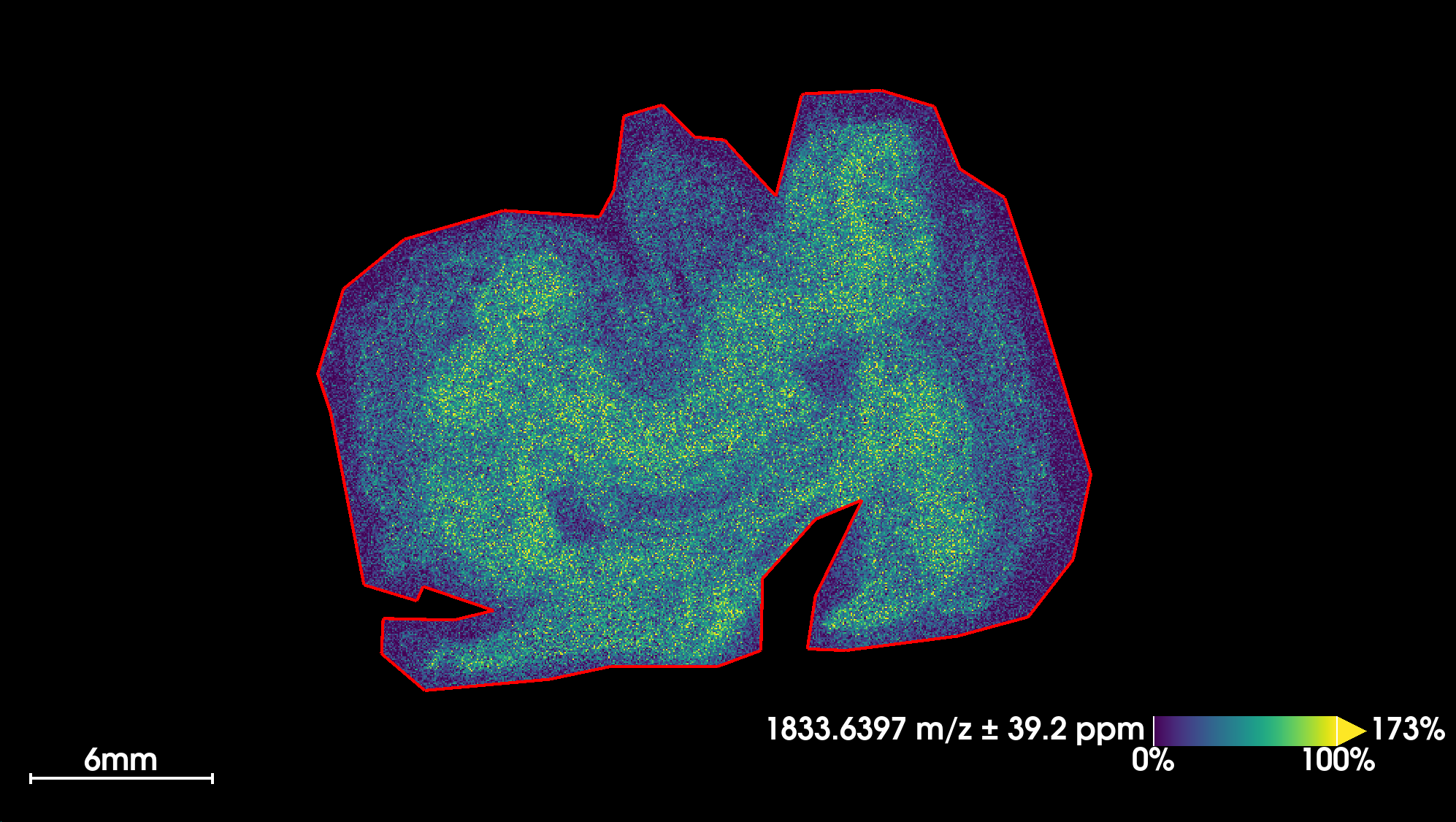

Supplement: Supplementary file 8 — Source Data 2 [file 41467_2026_72853_MOESM8_ESM.zip › Source Data MALDI Images/Supplementary Figure 8/1833.6397 mz ┬▒ 71.8 mDa.png]

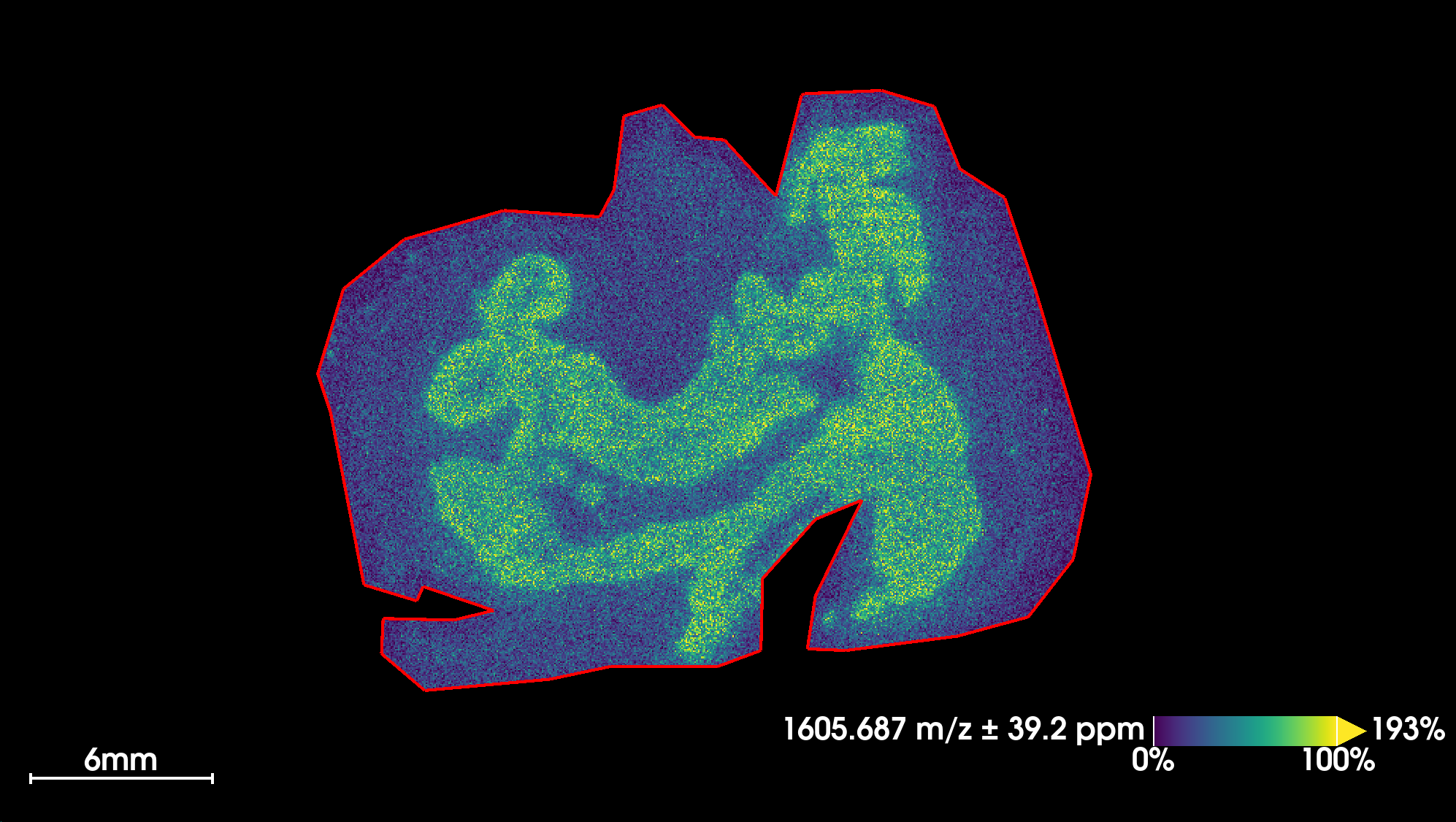

Supplement: Supplementary file 8 — Source Data 2 [file 41467_2026_72853_MOESM8_ESM.zip › Source Data MALDI Images/Supplementary Figure 8/1605.687 mz ┬▒ 62.9 mDa.png]

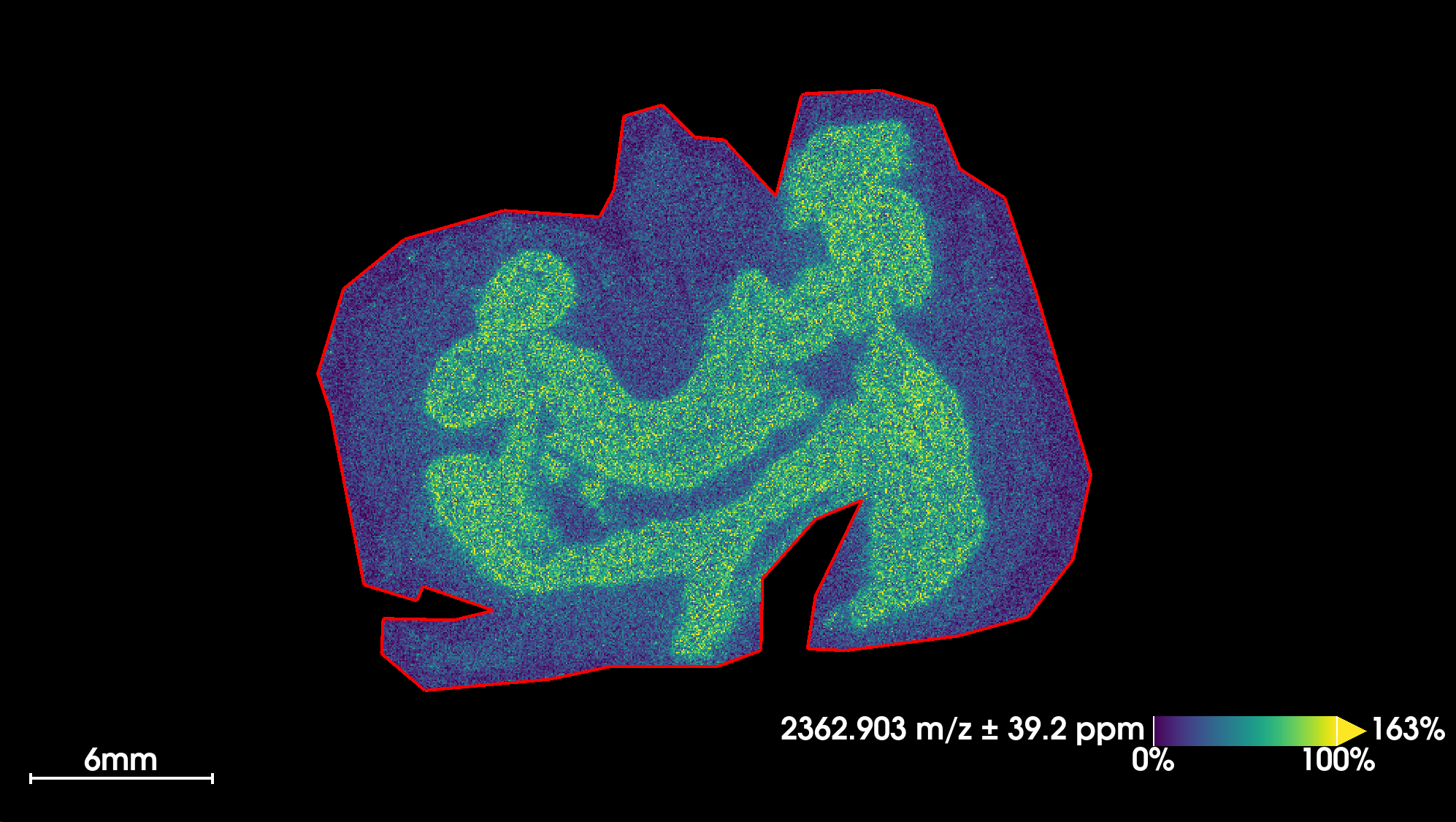

Supplement: Supplementary file 8 — Source Data 2 [file 41467_2026_72853_MOESM8_ESM.zip › Source Data MALDI Images/Supplementary Figure 8/2362.903 mz ┬▒ 92.5 mDa.png]

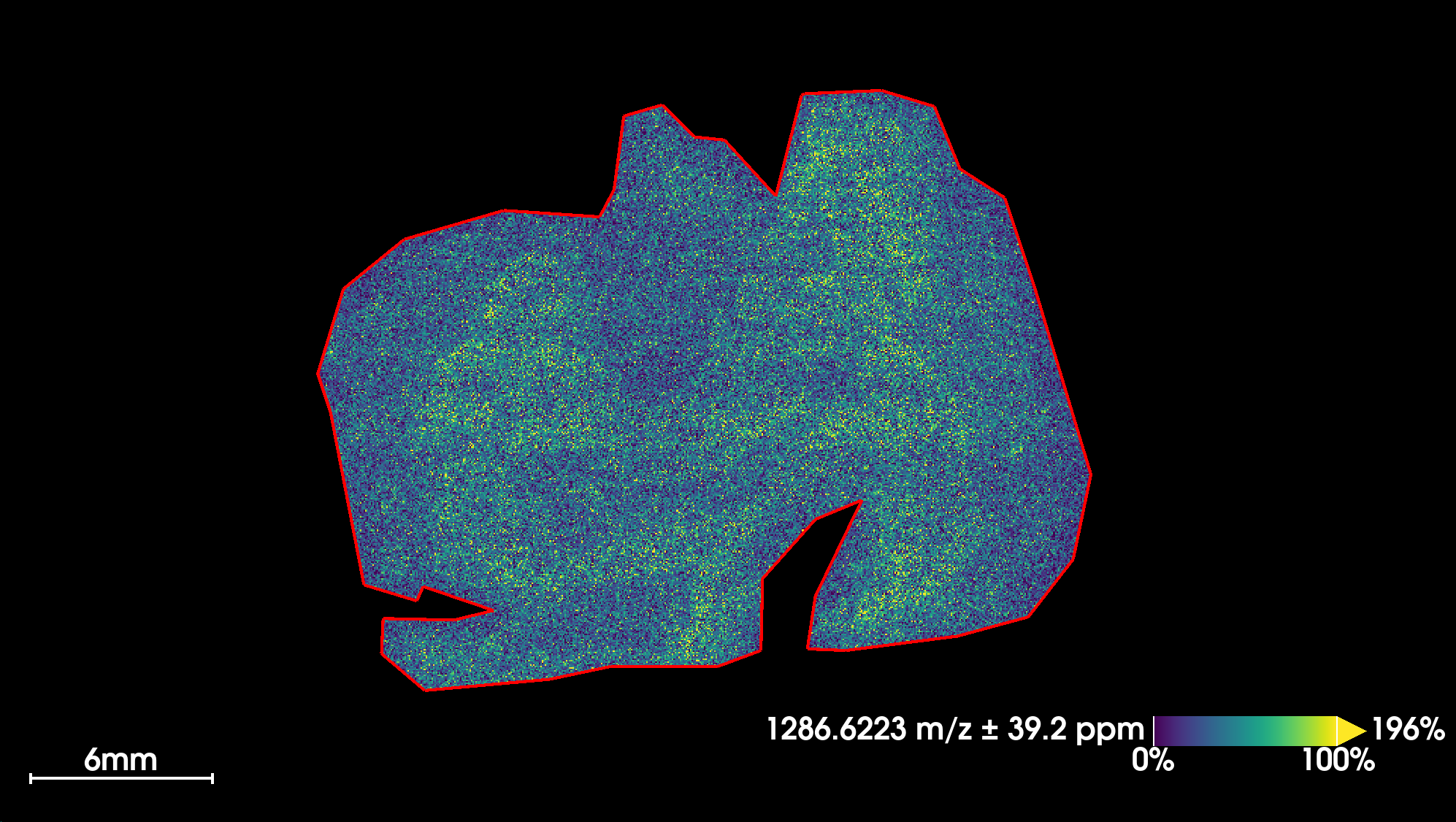

Supplement: Supplementary file 8 — Source Data 2 [file 41467_2026_72853_MOESM8_ESM.zip › Source Data MALDI Images/Supplementary Figure 8/1286.6223 mz ┬▒ 50.4 mDa.png]

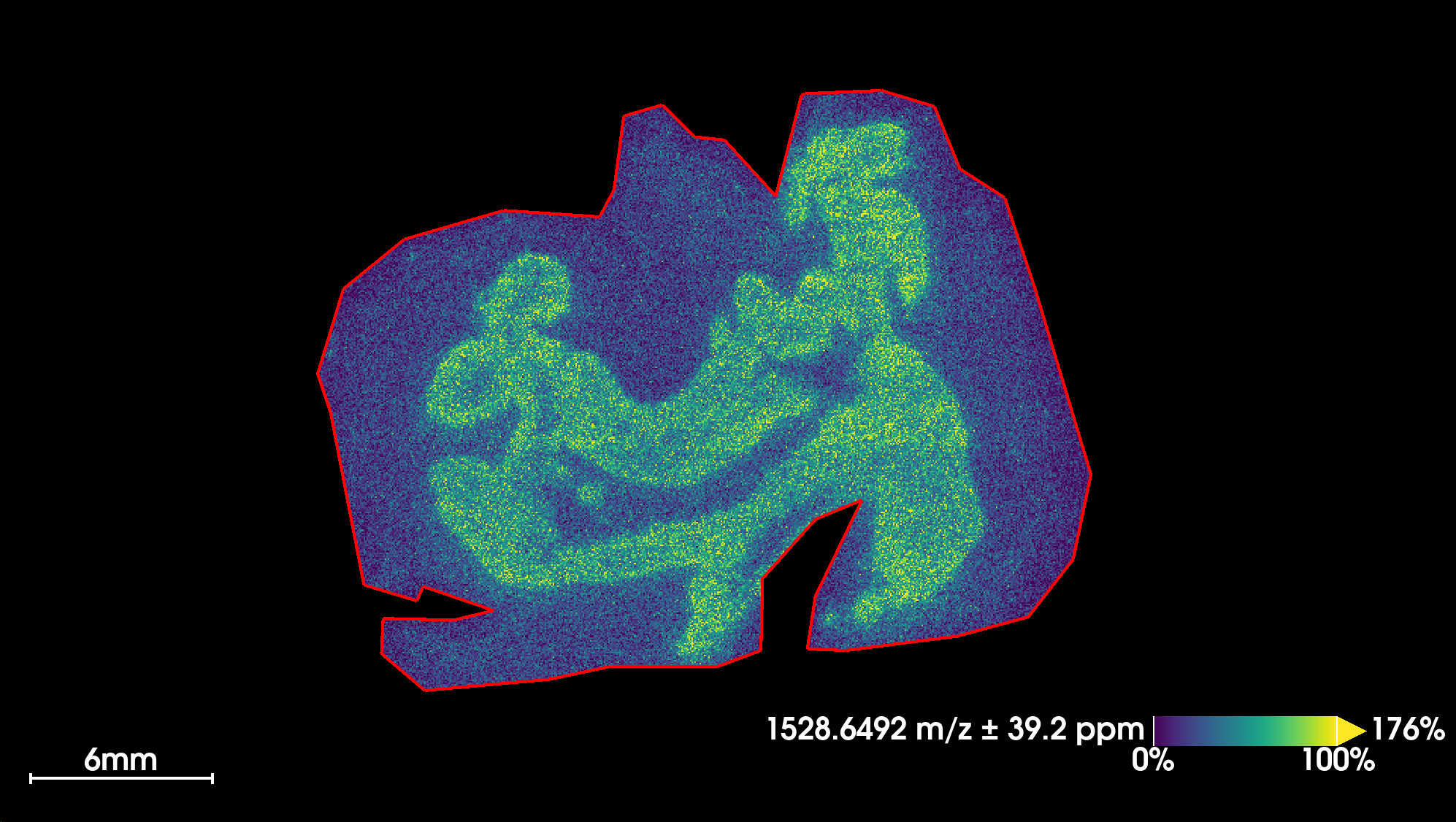

Supplement: Supplementary file 8 — Source Data 2 [file 41467_2026_72853_MOESM8_ESM.zip › Source Data MALDI Images/Supplementary Figure 8/1528.6492 mz ┬▒ 59.9 mDa.png]

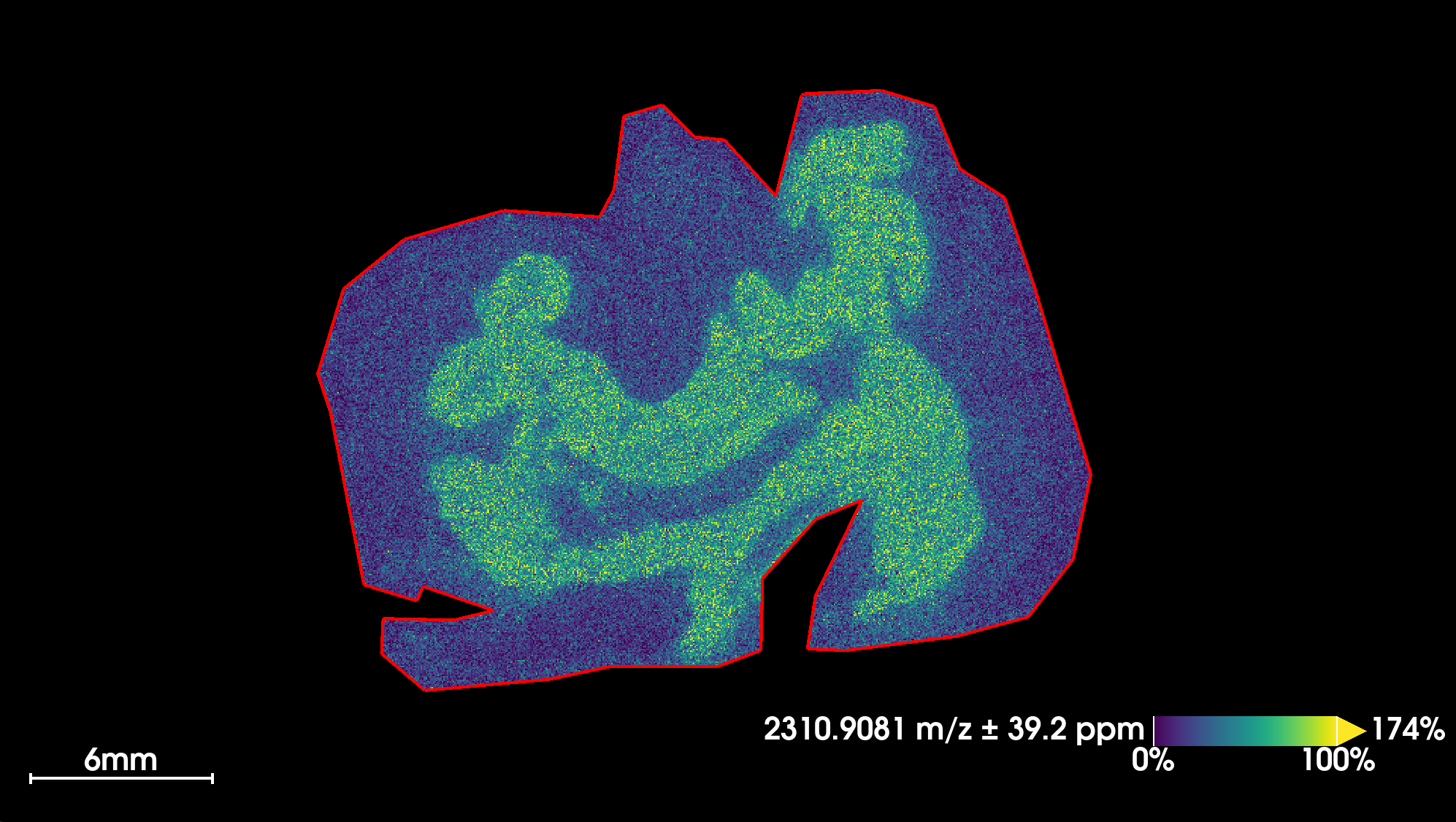

Supplement: Supplementary file 8 — Source Data 2 [file 41467_2026_72853_MOESM8_ESM.zip › Source Data MALDI Images/Supplementary Figure 8/2310.9081 mz ┬▒ 90.5 mDa.png]

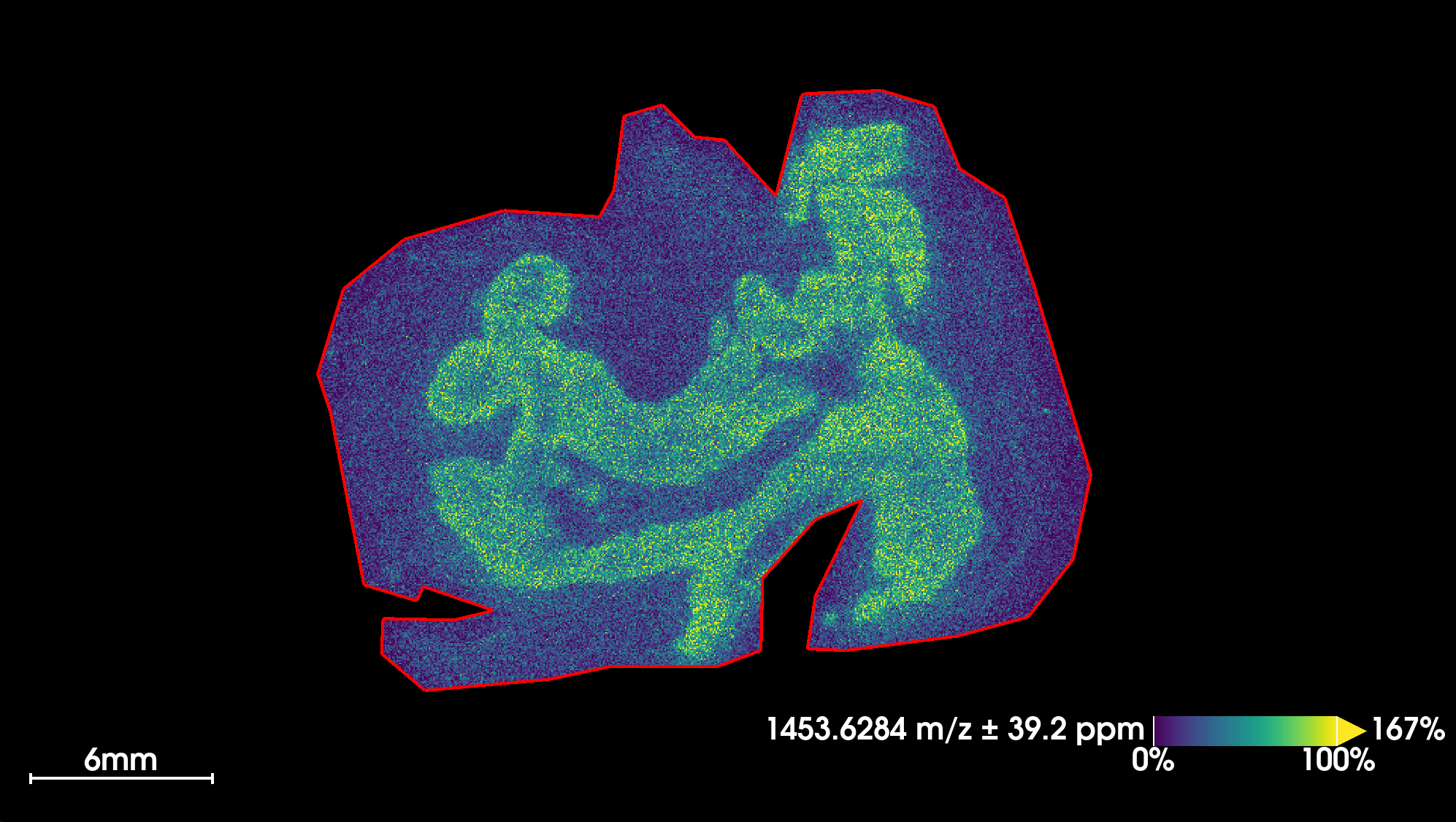

Supplement: Supplementary file 8 — Source Data 2 [file 41467_2026_72853_MOESM8_ESM.zip › Source Data MALDI Images/Supplementary Figure 8/1453.6284 mz ┬▒ 56.9 mDa.png]

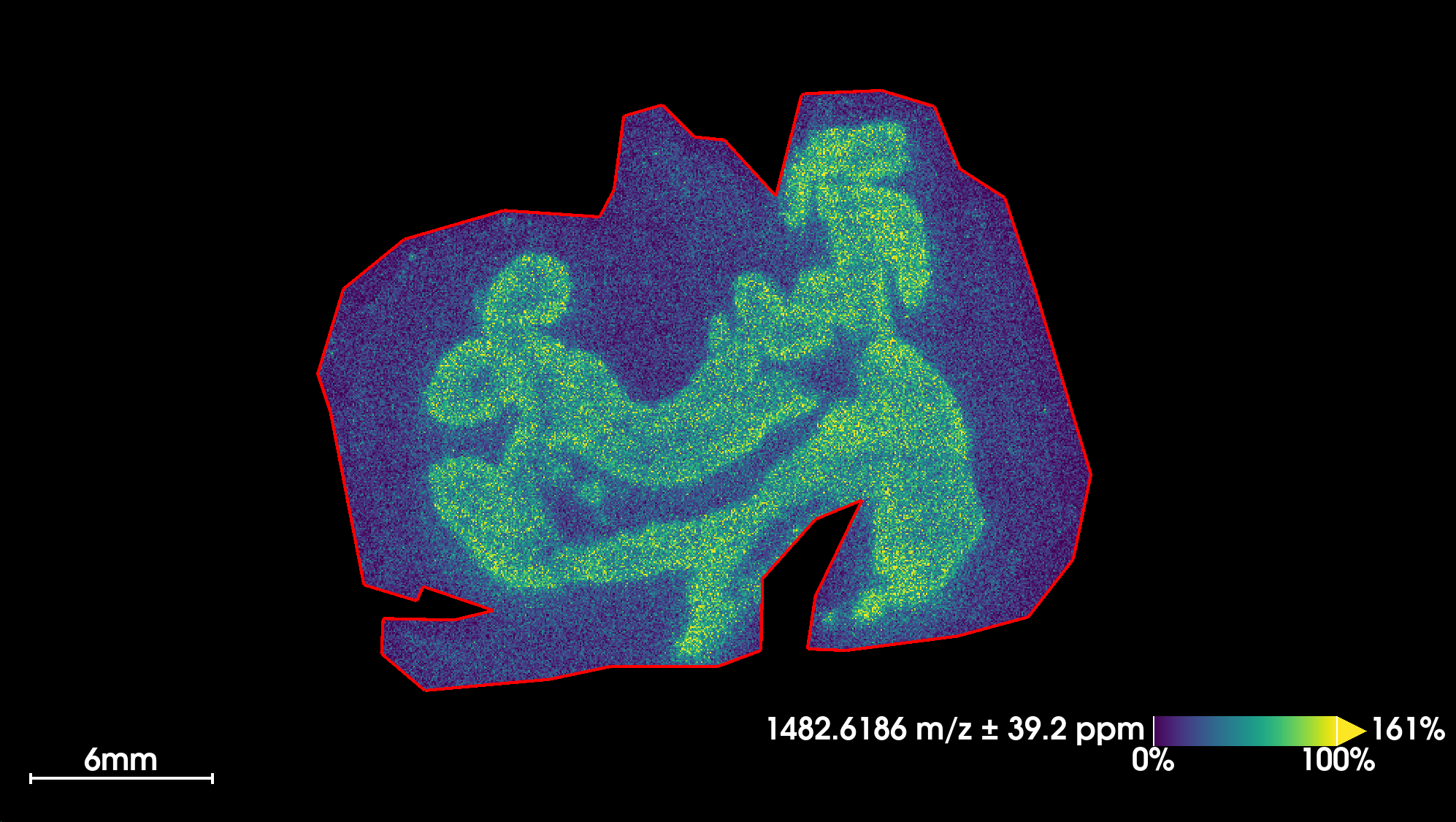

Supplement: Supplementary file 8 — Source Data 2 [file 41467_2026_72853_MOESM8_ESM.zip › Source Data MALDI Images/Supplementary Figure 8/1482.6186 mz ┬▒ 58.1 mDa.png]
