# Supplementary figures and images for: Glycosite mapping and in situ mass spectrometry imaging of MUC2 glycopeptides via on-slide mucinase digestion (part 1 of 2)
Source: Nat Commun. 2026 May 7;17:6125. doi: 10.1038/s41467-026-72853-3 (PMC13357551; doi:10.1038/s41467-026-72853-3)

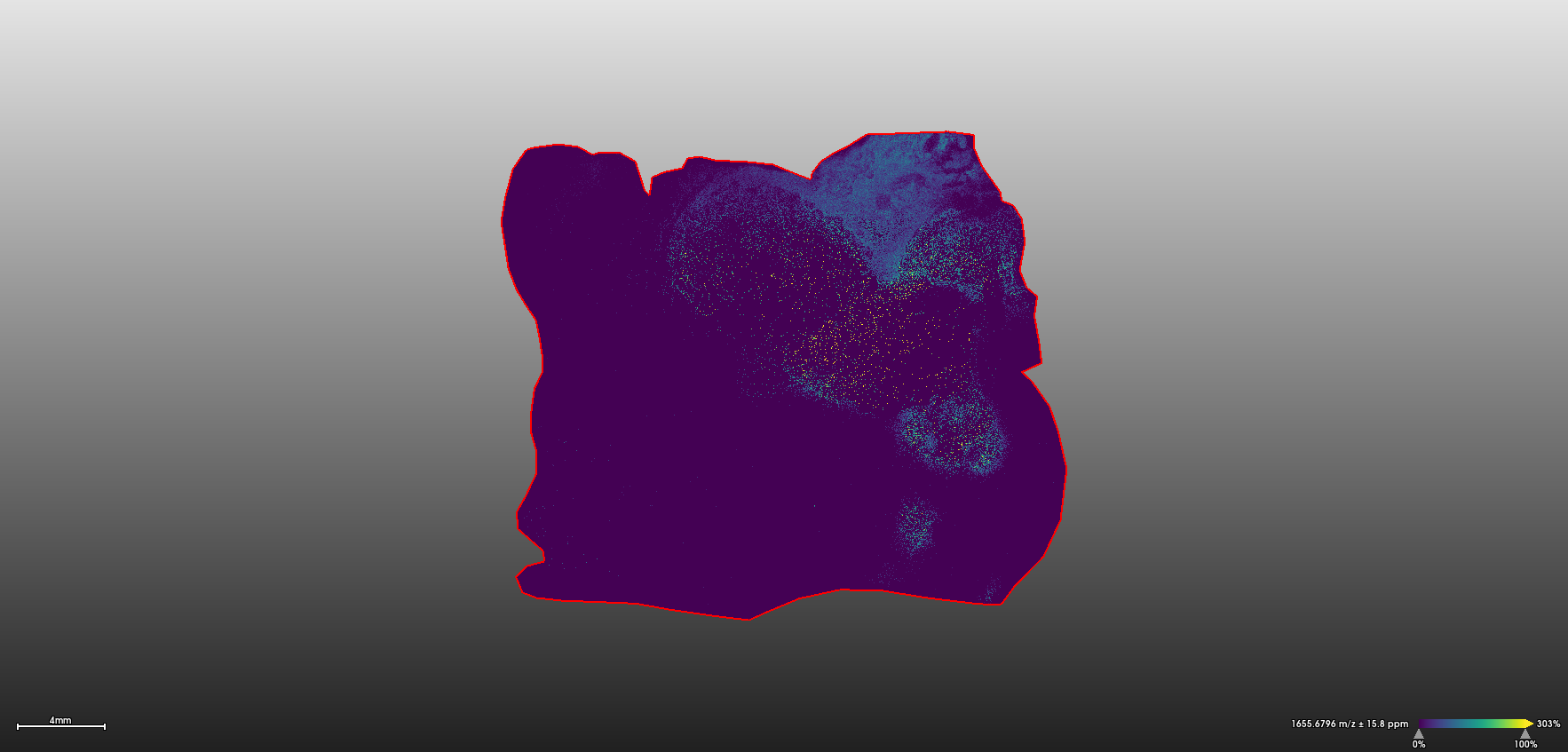

Supplement: Supplementary file 8 — Source Data 2 [file 41467_2026_72853_MOESM8_ESM.zip › Source Data MALDI Images/Supplementary Figure 12/20240531_TPSPPTT+H2N3_Colon1a.png]

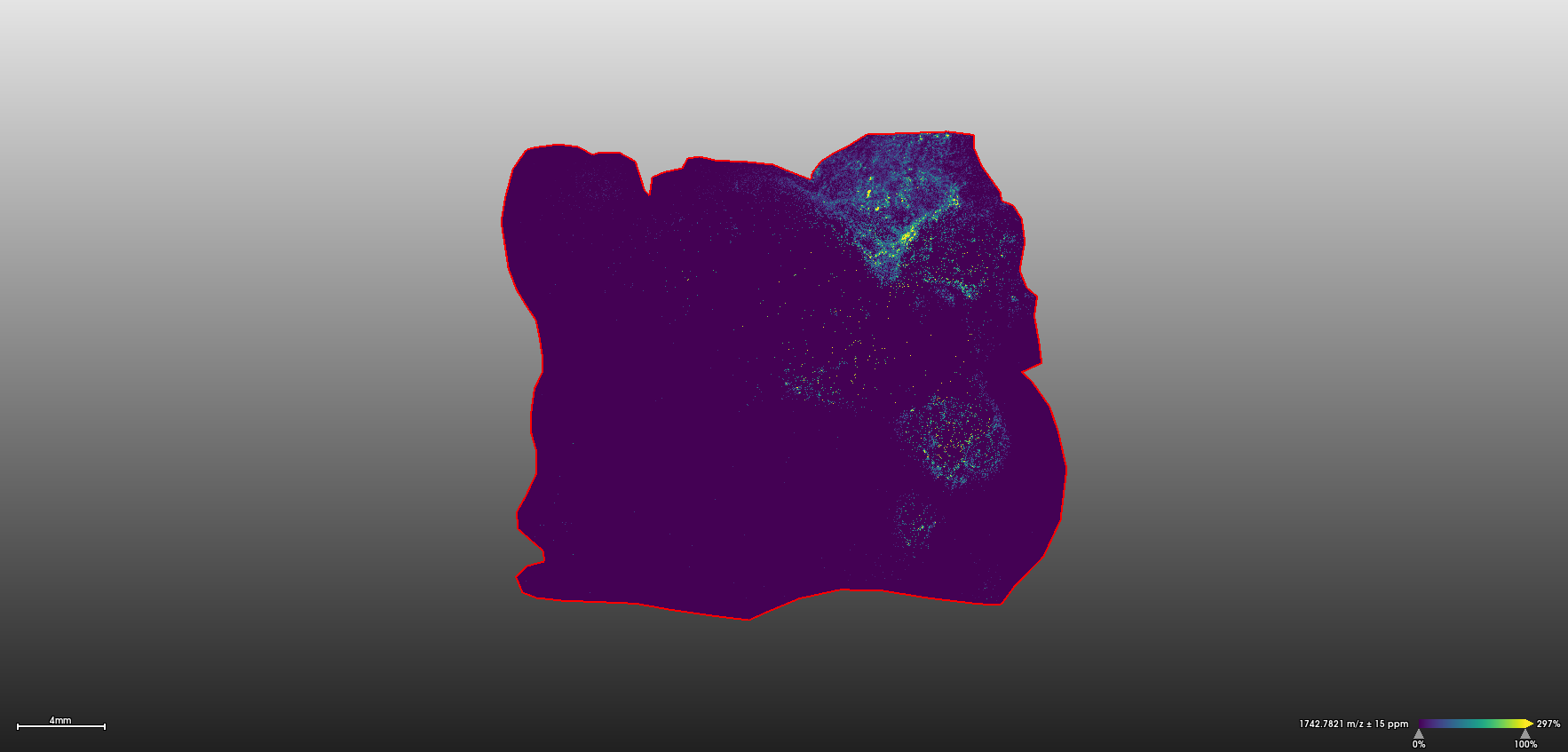

Supplement: Supplementary file 8 — Source Data 2 [file 41467_2026_72853_MOESM8_ESM.zip › Source Data MALDI Images/Supplementary Figure 12/20240627_TLTPITT+H1N4_Colon1a.png]

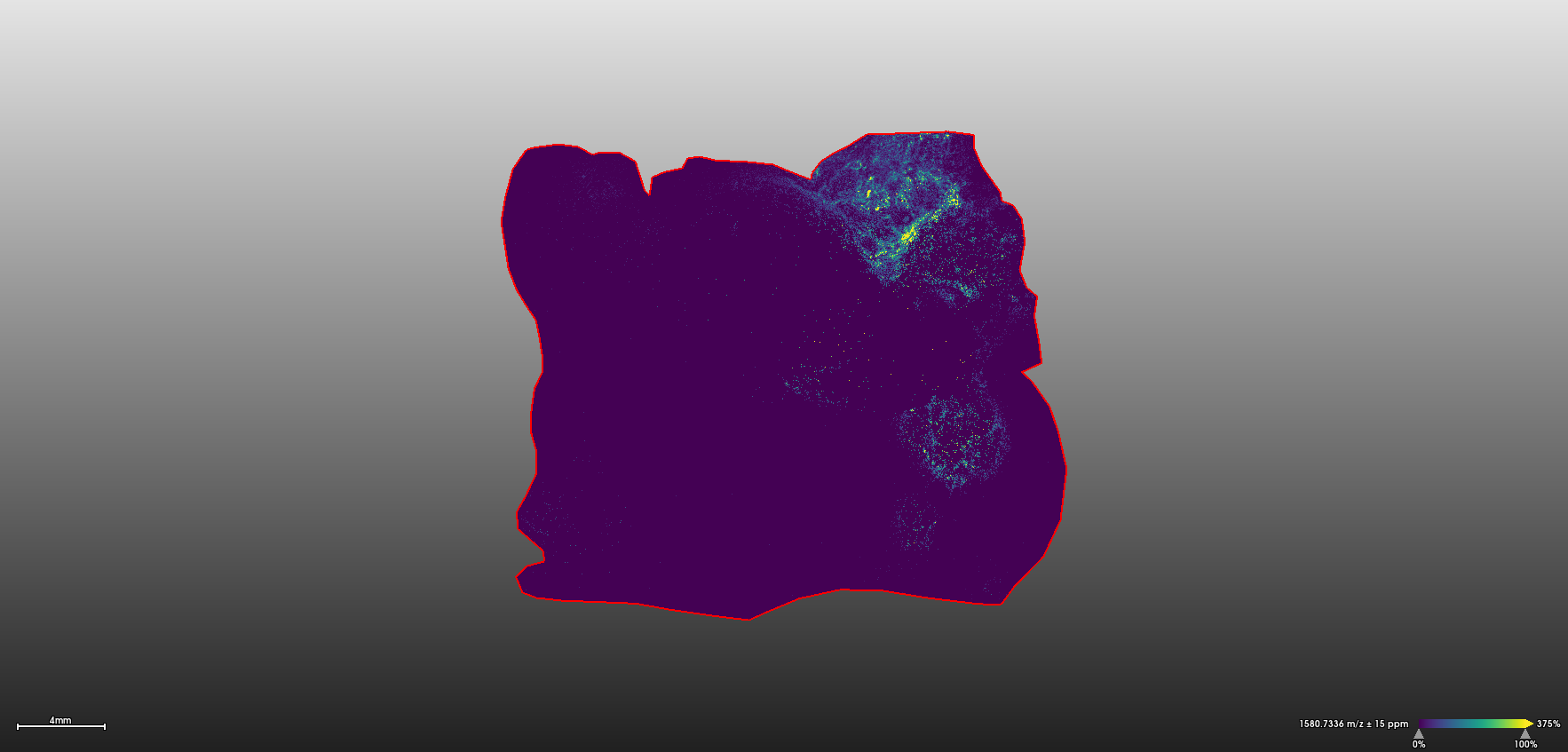

Supplement: Supplementary file 8 — Source Data 2 [file 41467_2026_72853_MOESM8_ESM.zip › Source Data MALDI Images/Supplementary Figure 12/20240627_TLTPITT+N4_Colon1a.png]

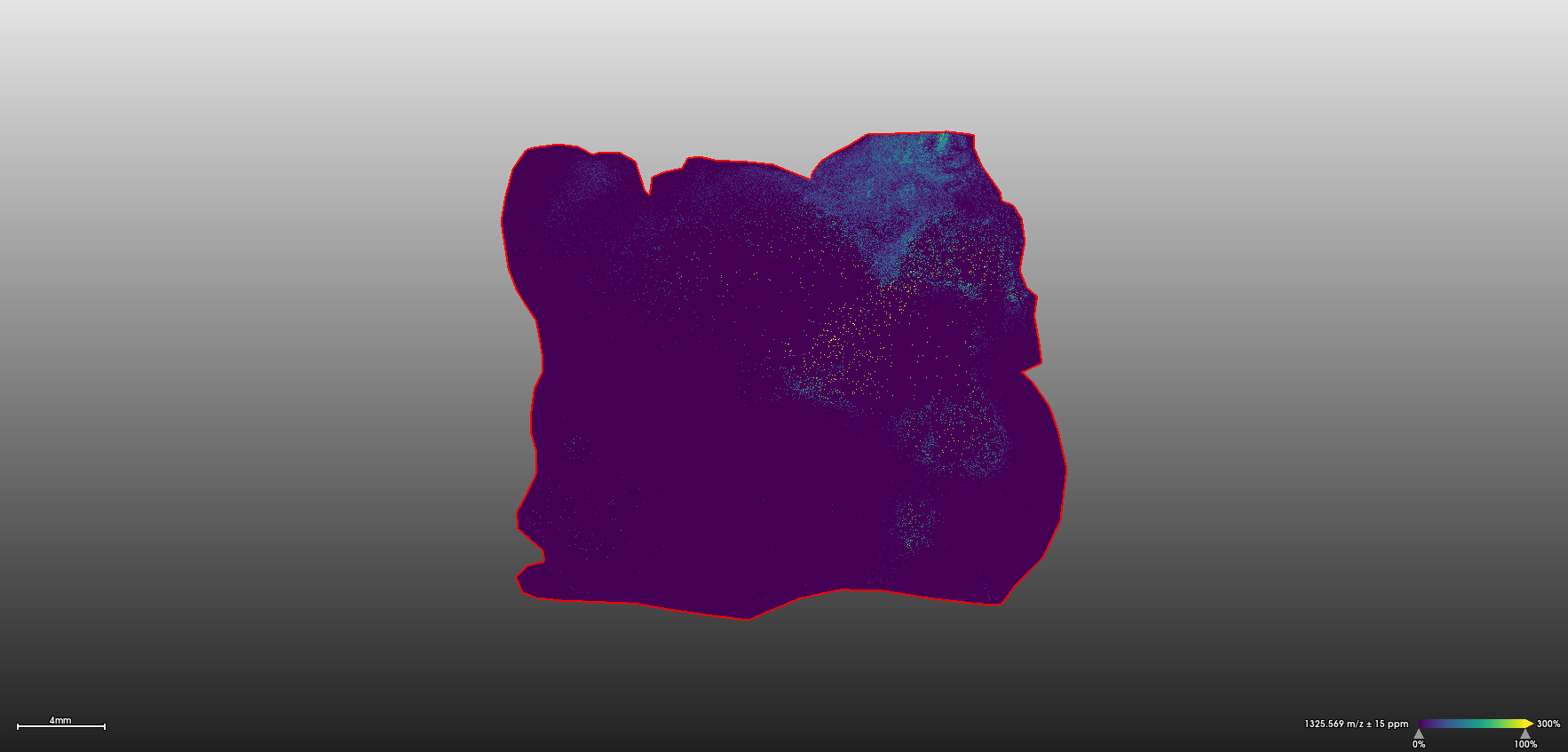

Supplement: Supplementary file 8 — Source Data 2 [file 41467_2026_72853_MOESM8_ESM.zip › Source Data MALDI Images/Supplementary Figure 12/20240627_TPITT+H1N3_Colon1a.png]

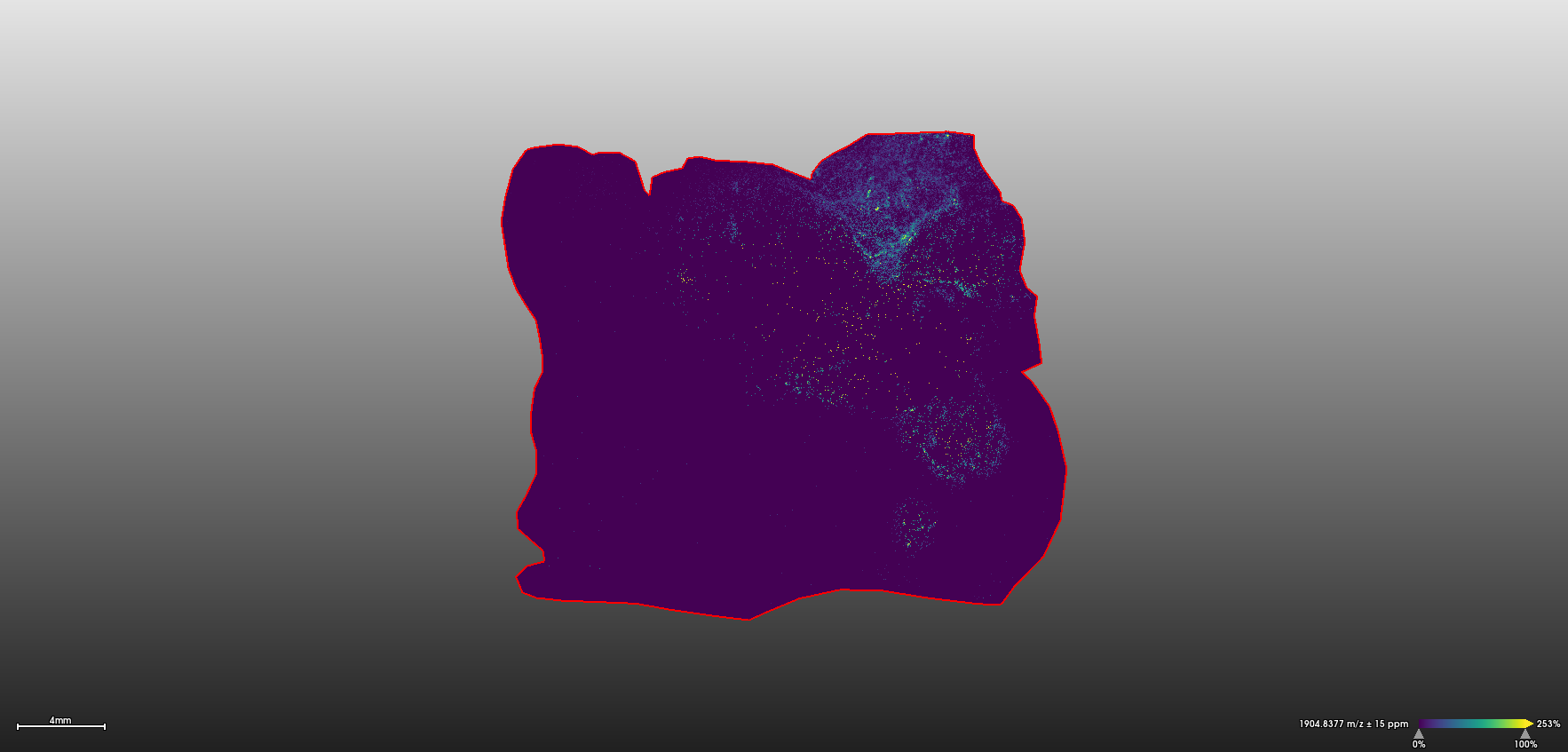

Supplement: Supplementary file 8 — Source Data 2 [file 41467_2026_72853_MOESM8_ESM.zip › Source Data MALDI Images/Supplementary Figure 12/20240627_TLTPITT+H2N4_Colon1a.png]

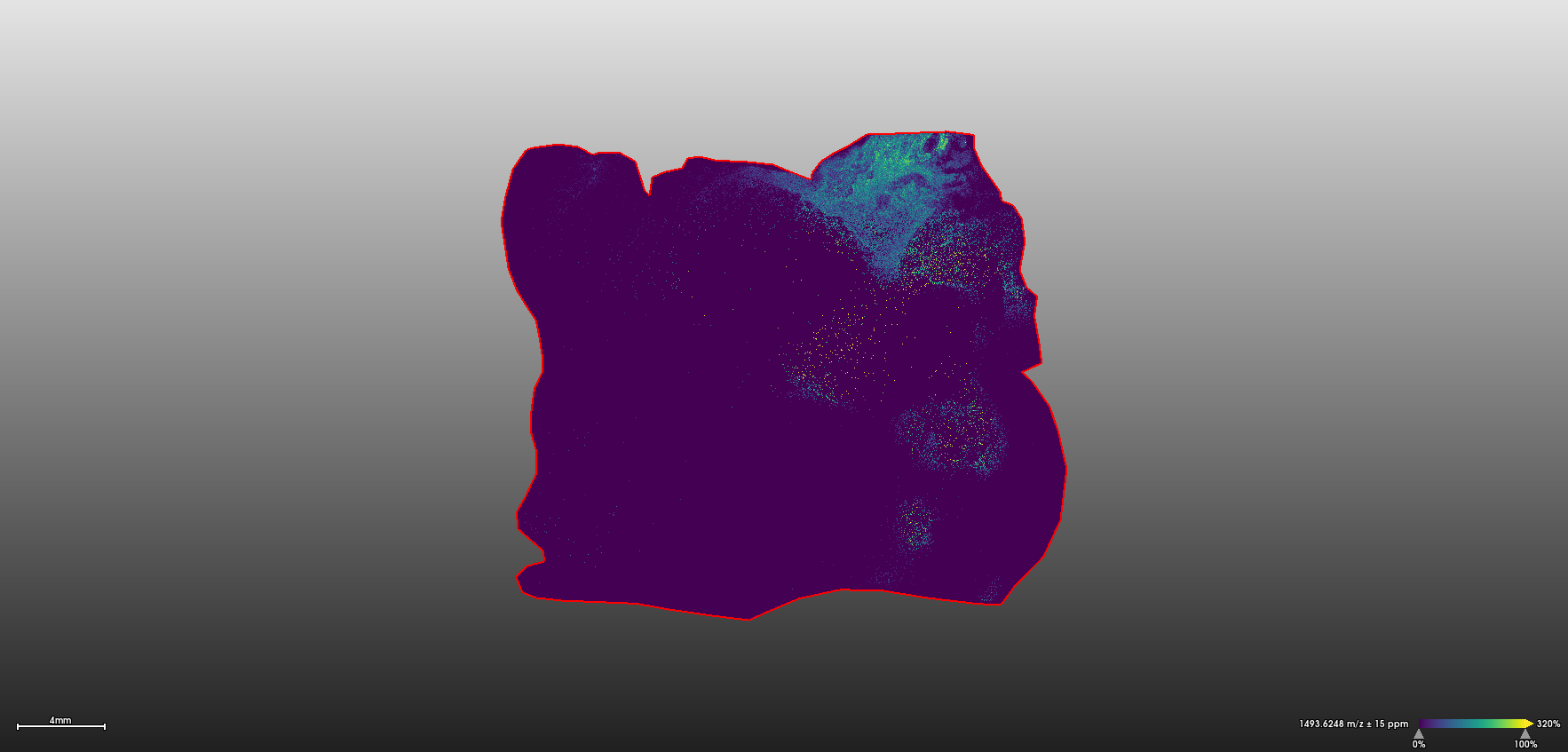

Supplement: Supplementary file 8 — Source Data 2 [file 41467_2026_72853_MOESM8_ESM.zip › Source Data MALDI Images/Supplementary Figure 12/20240531_TPSPPTT+H1N3_Colon1a.png]

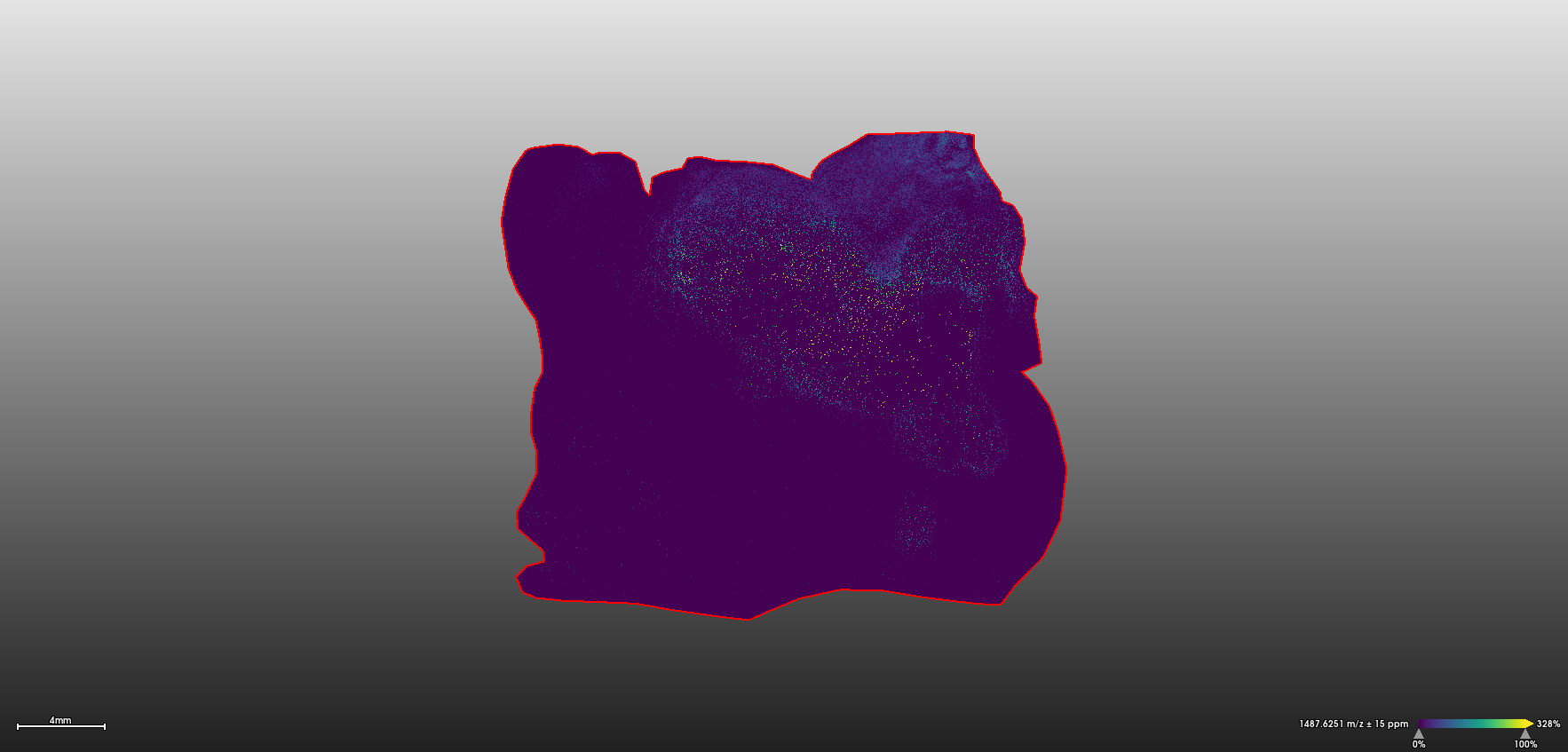

Supplement: Supplementary file 8 — Source Data 2 [file 41467_2026_72853_MOESM8_ESM.zip › Source Data MALDI Images/Supplementary Figure 12/20240627_TPITT+H2N3_Colon1a.png]

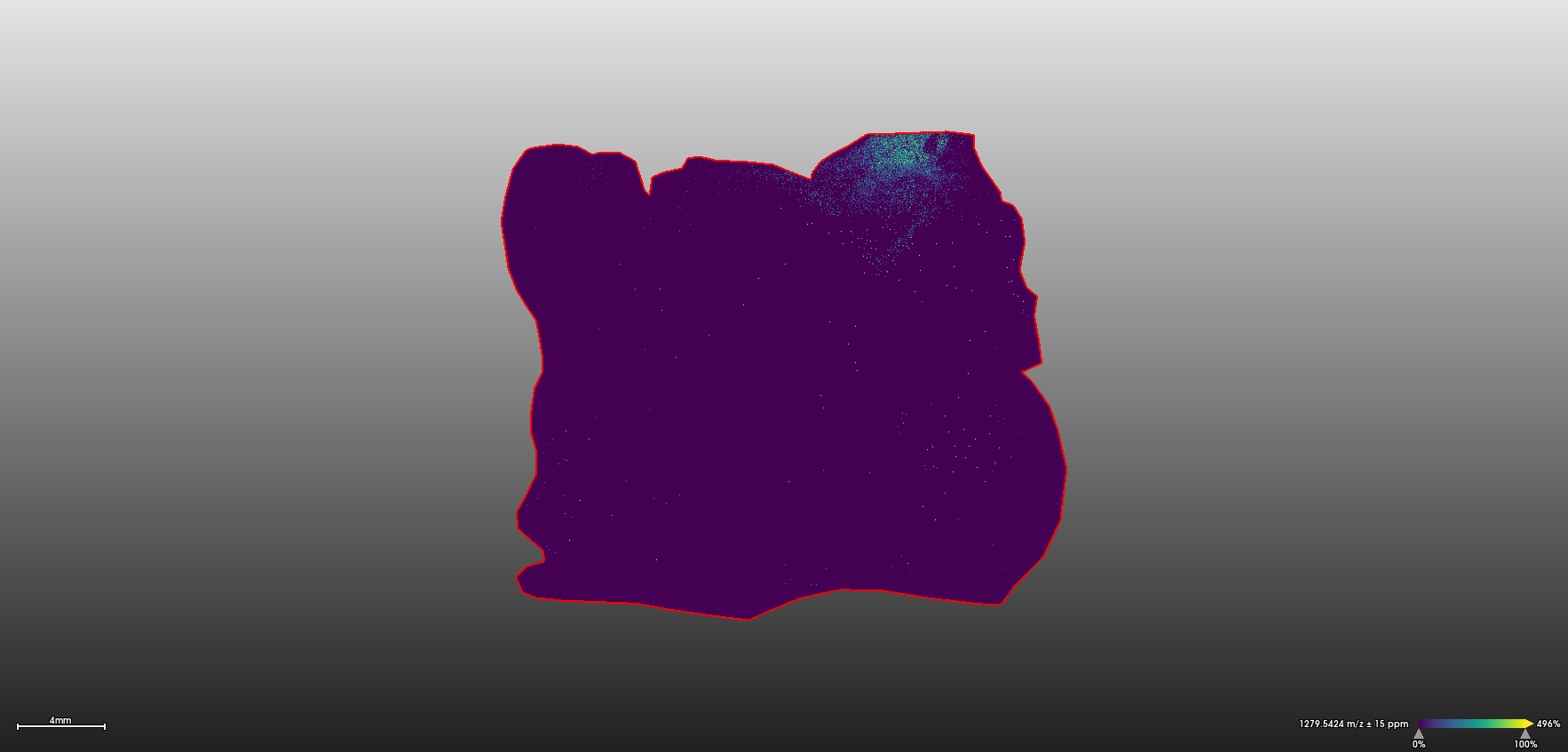

Supplement: Supplementary file 8 — Source Data 2 [file 41467_2026_72853_MOESM8_ESM.zip › Source Data MALDI Images/Supplementary Figure 12/20240531_TQTPTT+N3_Colon1a.png]

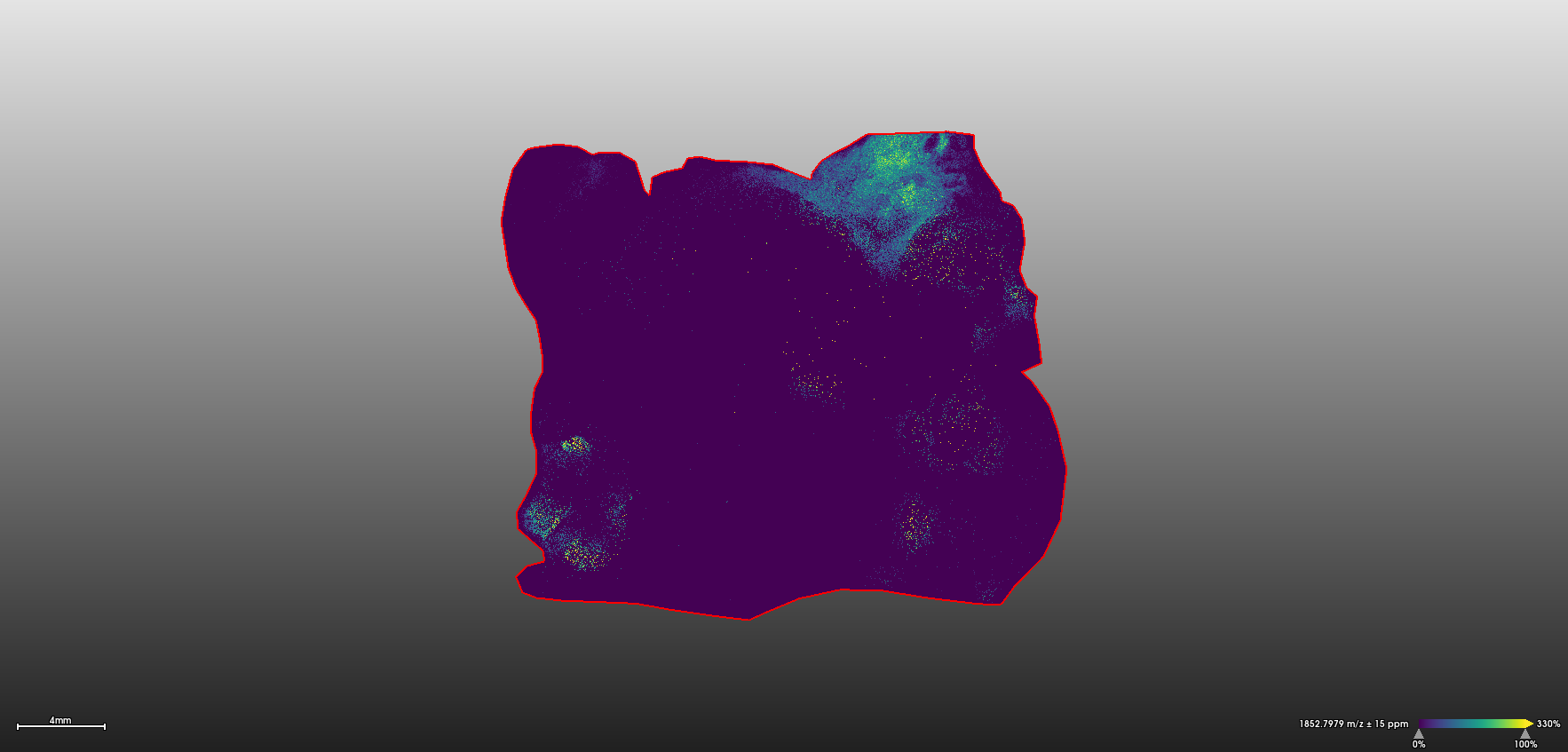

Supplement: Supplementary file 8 — Source Data 2 [file 41467_2026_72853_MOESM8_ESM.zip › Source Data MALDI Images/Supplementary Figure 12/20240531_TTTPPPTT+N5_Colon1a.png]

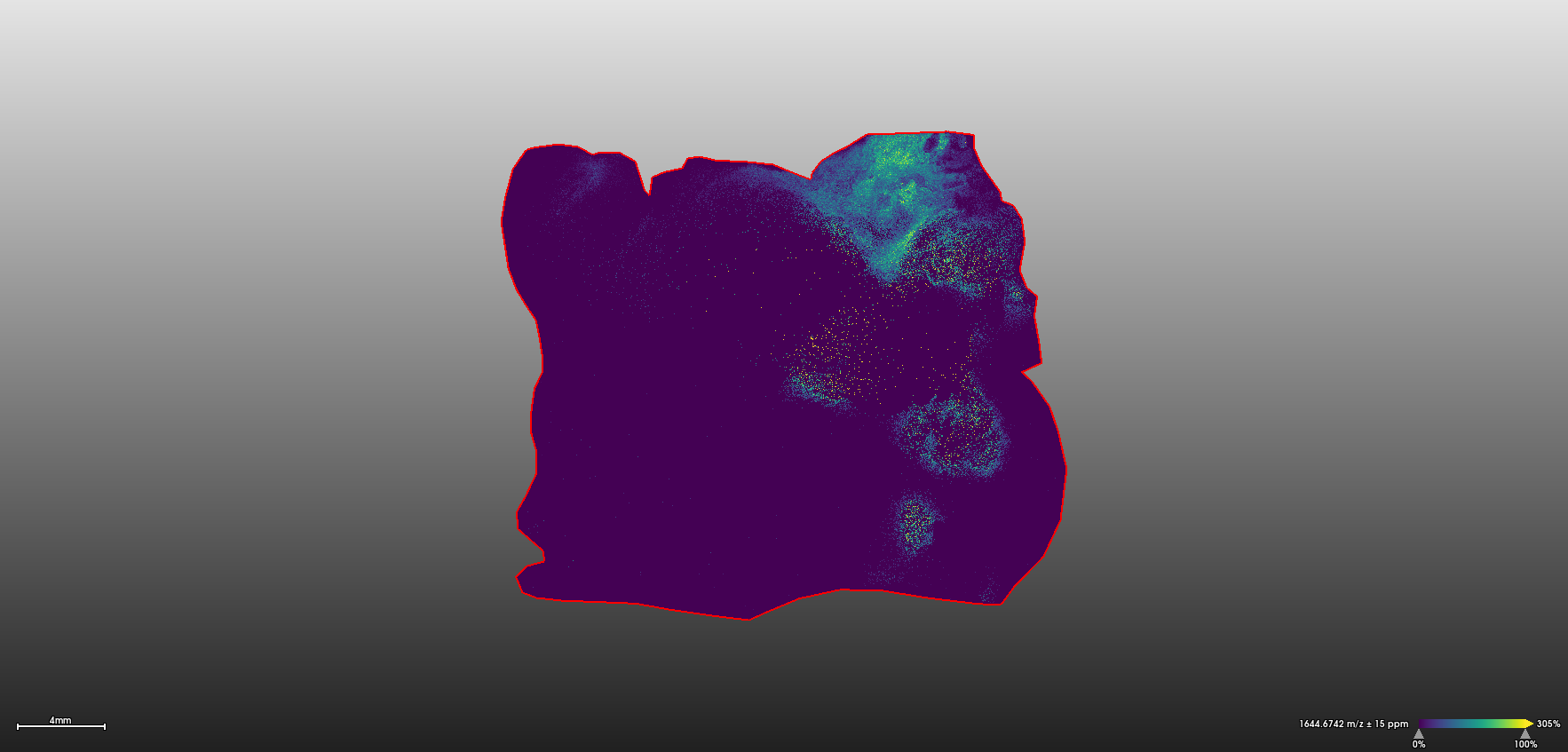

Supplement: Supplementary file 8 — Source Data 2 [file 41467_2026_72853_MOESM8_ESM.zip › Source Data MALDI Images/Supplementary Figure 12/20240531_TQTPTT+H1N4_Colon1a.png]

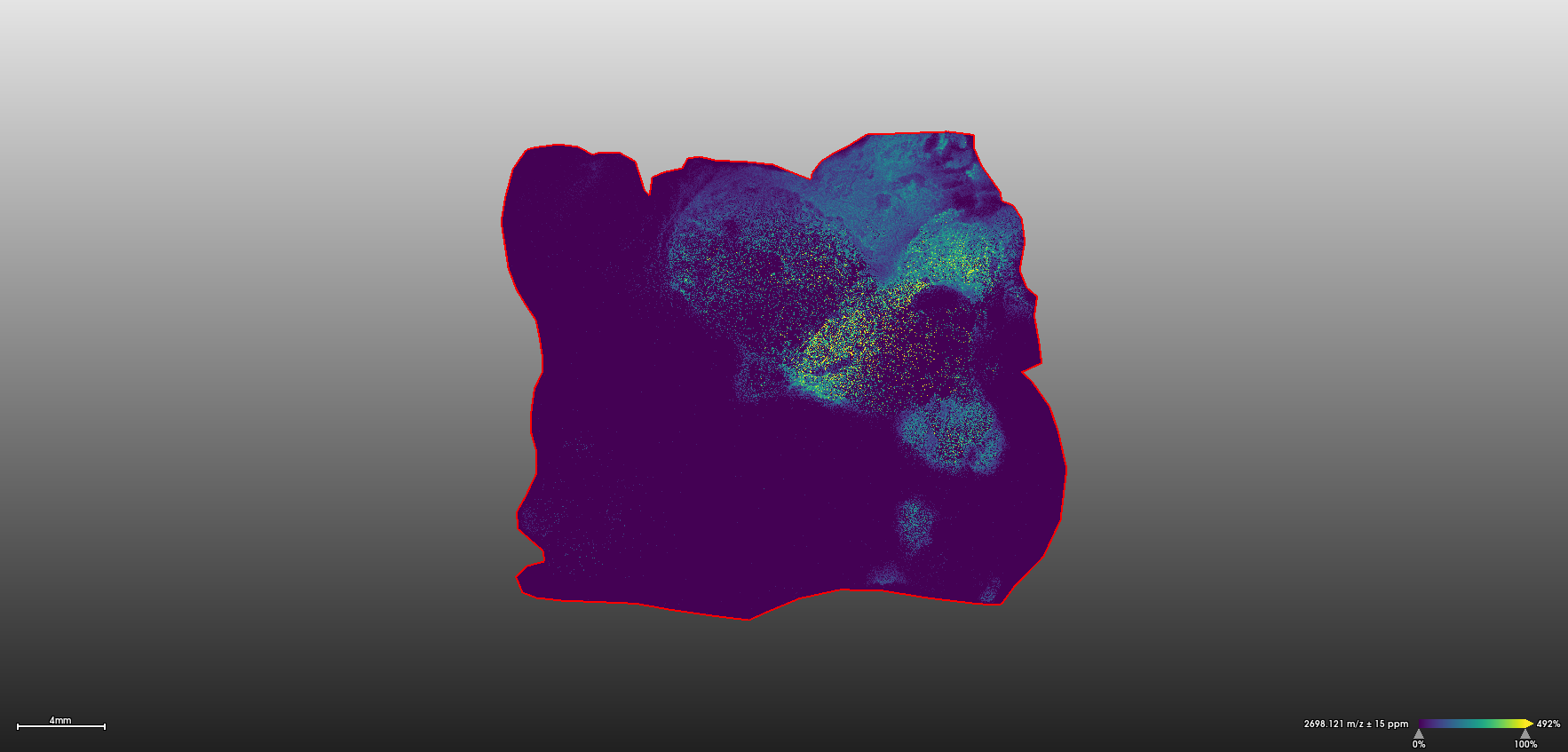

Supplement: Supplementary file 8 — Source Data 2 [file 41467_2026_72853_MOESM8_ESM.zip › Source Data MALDI Images/Supplementary Figure 12/20240524_TVTPTPTPTG+H3N6_Colon1a.png]

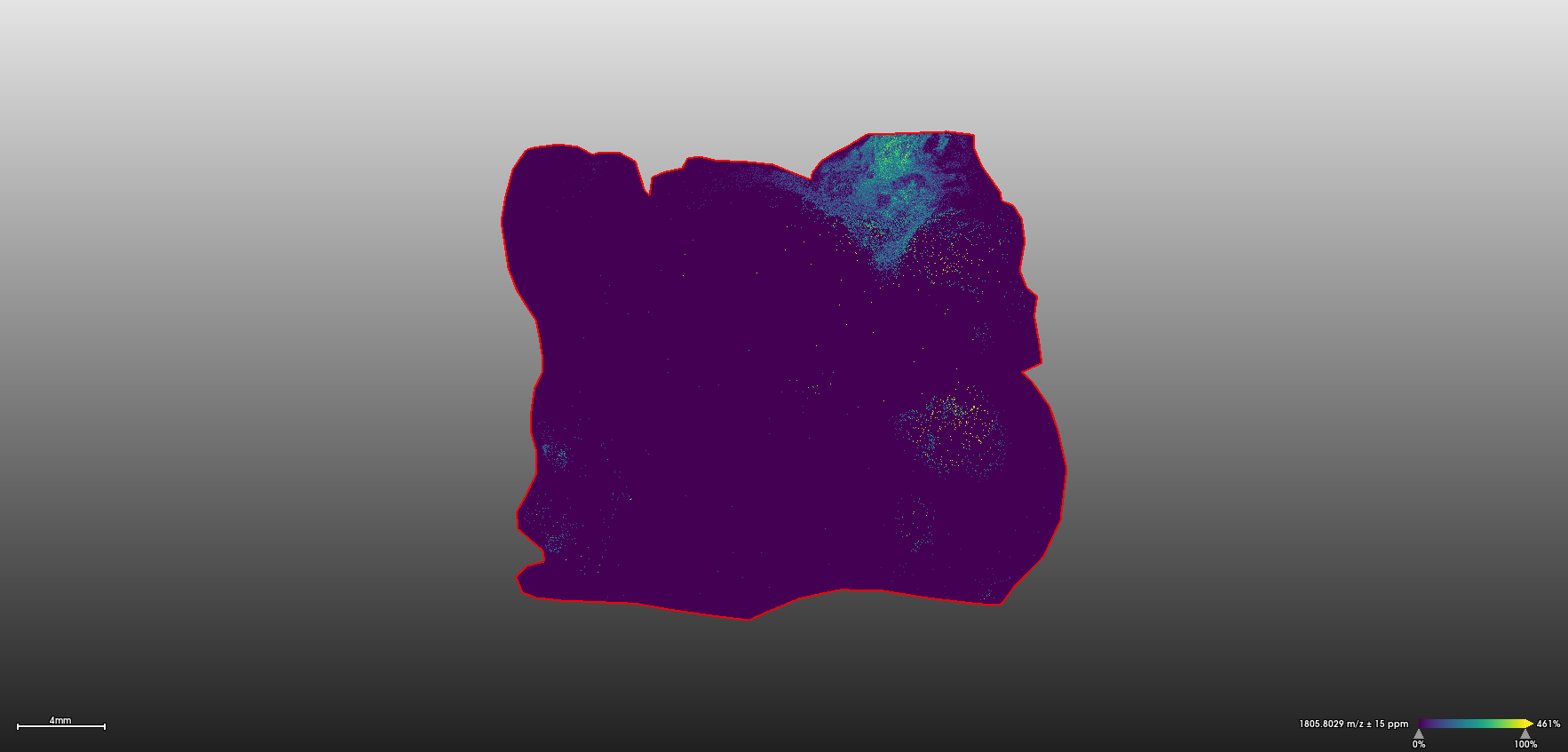

Supplement: Supplementary file 8 — Source Data 2 [file 41467_2026_72853_MOESM8_ESM.zip › Source Data MALDI Images/Supplementary Figure 12/20240524_TVTPTPTPTG+N4_Colon1a.png]

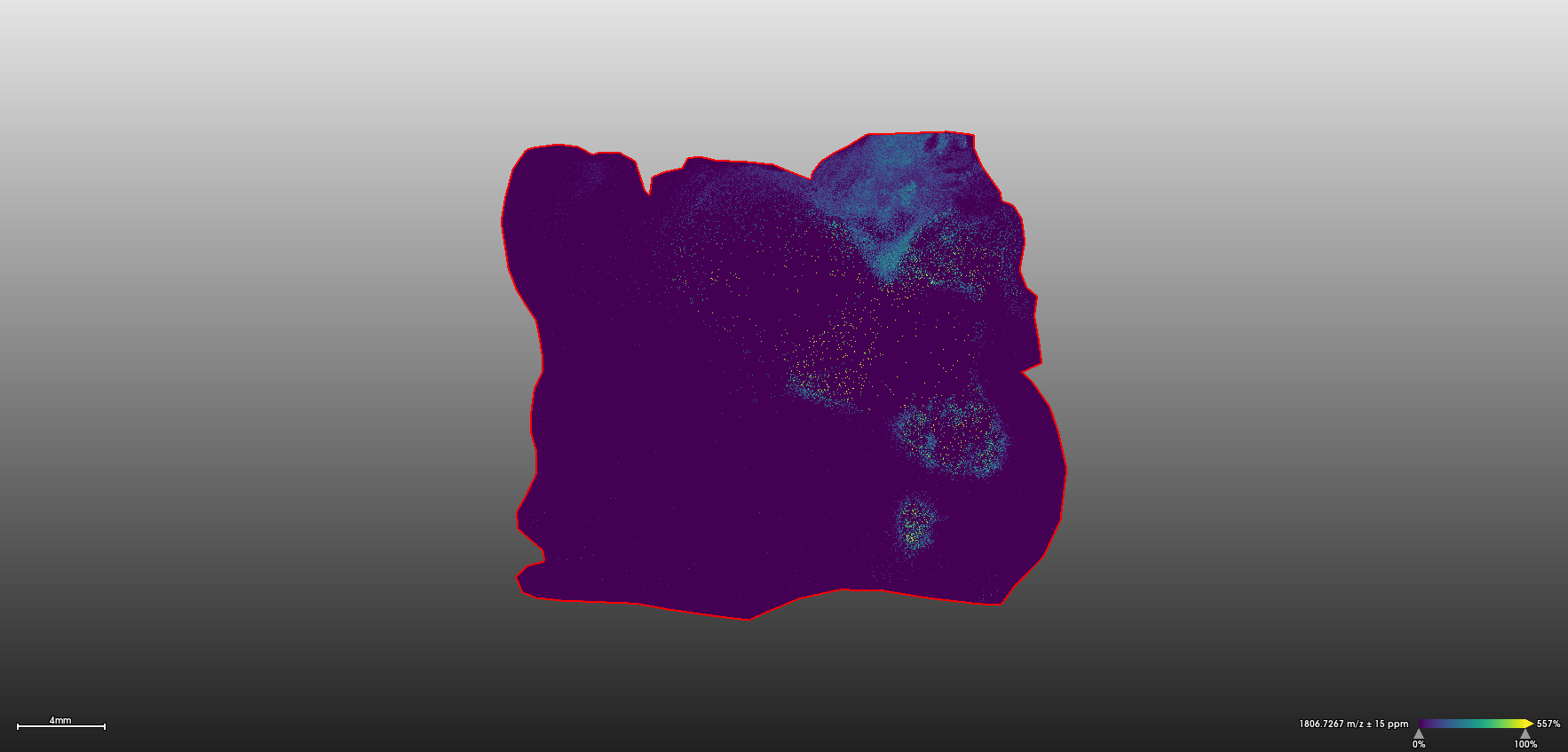

Supplement: Supplementary file 8 — Source Data 2 [file 41467_2026_72853_MOESM8_ESM.zip › Source Data MALDI Images/Supplementary Figure 12/20240531_TQTPTT+H2N4_Colon1a.png]

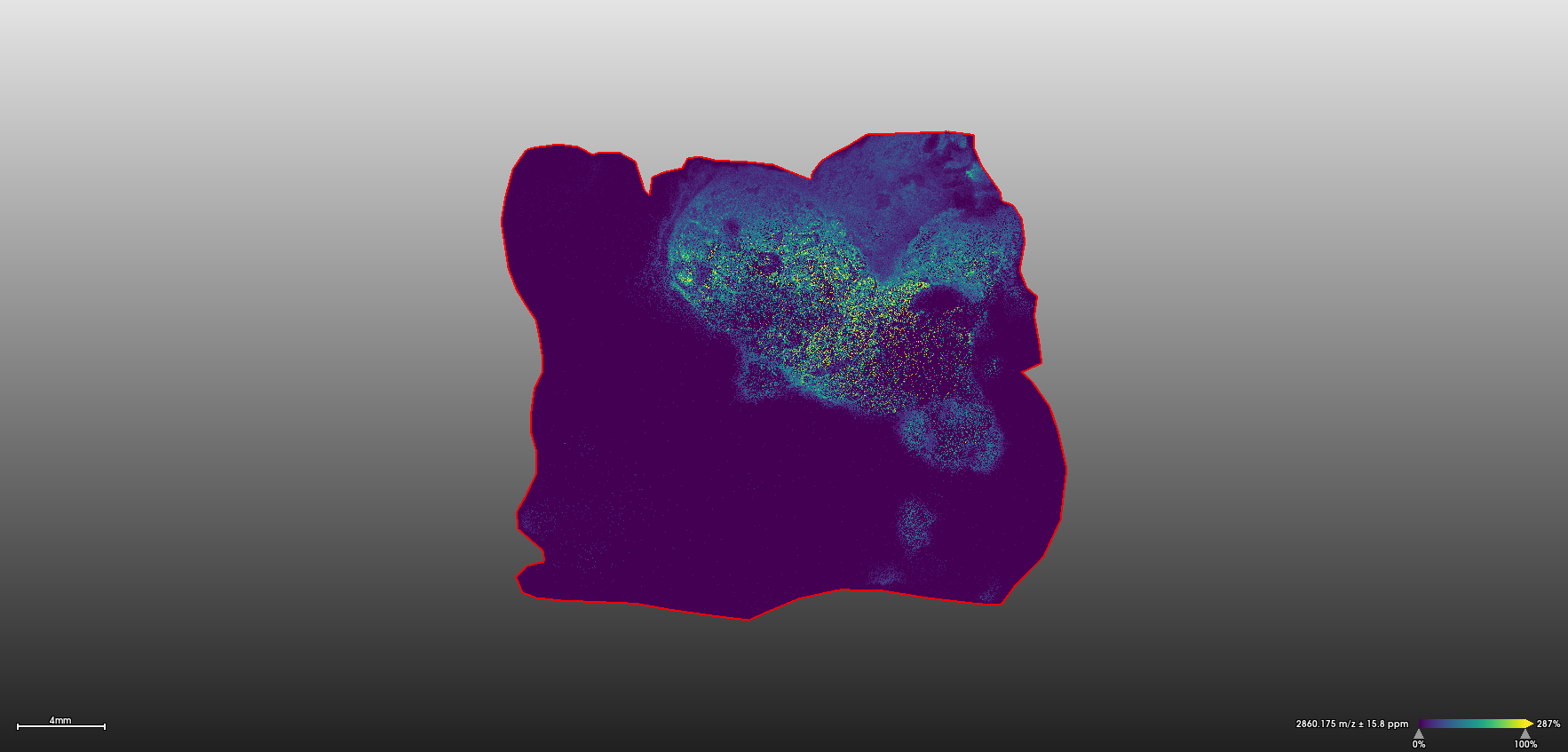

Supplement: Supplementary file 8 — Source Data 2 [file 41467_2026_72853_MOESM8_ESM.zip › Source Data MALDI Images/Supplementary Figure 12/20240524_TVTPTPTPTG+H4N6_Colon1a.png]

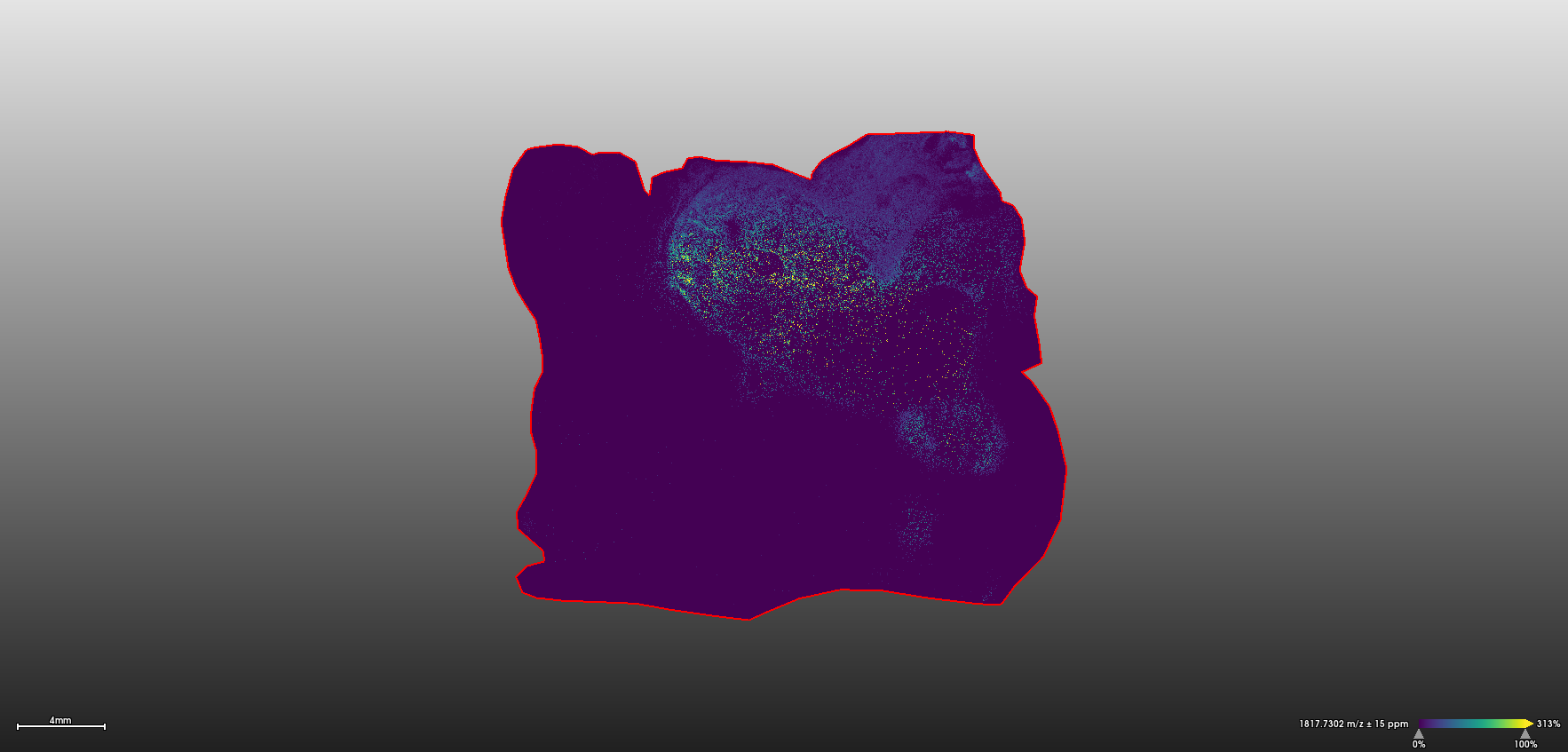

Supplement: Supplementary file 8 — Source Data 2 [file 41467_2026_72853_MOESM8_ESM.zip › Source Data MALDI Images/Supplementary Figure 12/20240627_TPSPPTT+H3N3_Colon1a.png]

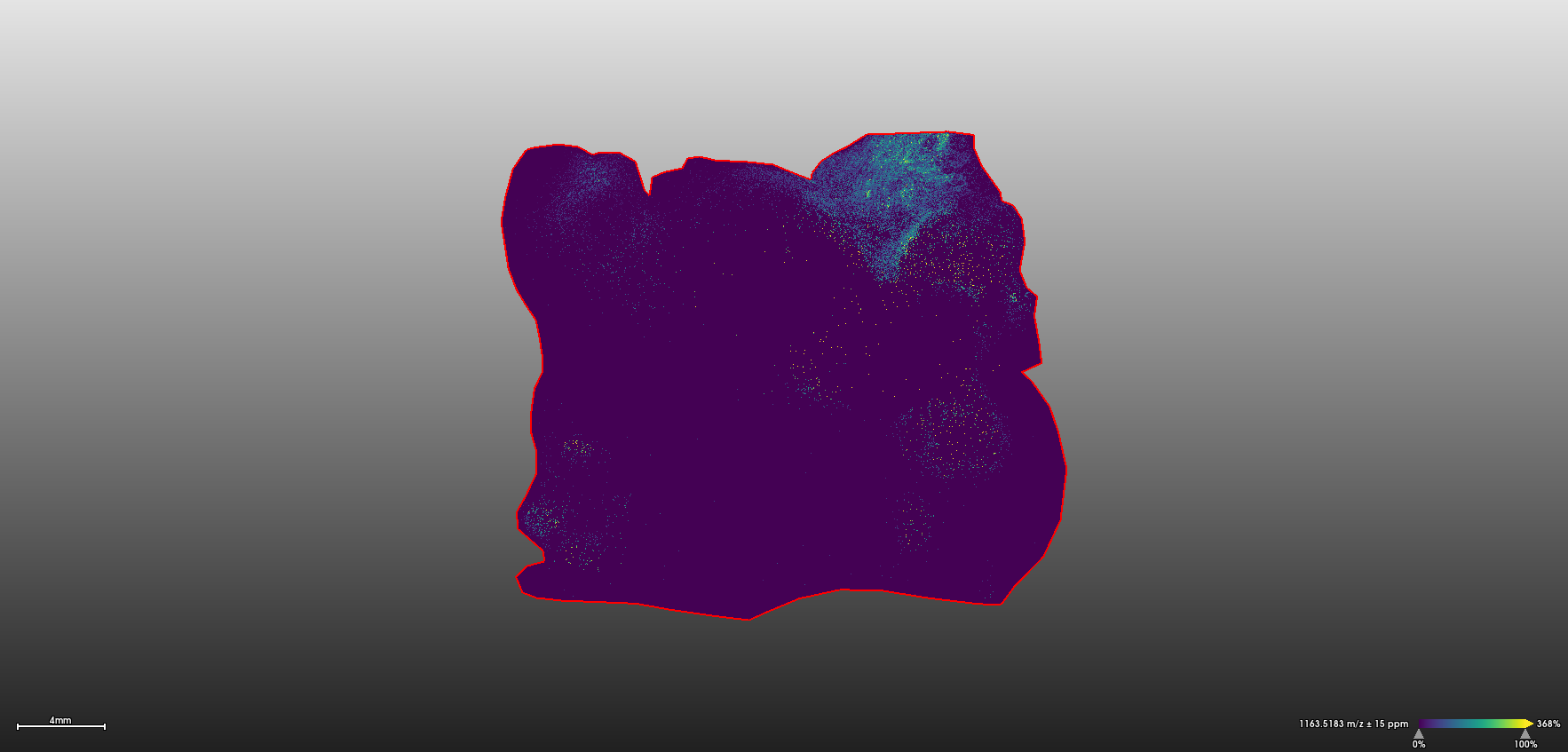

Supplement: Supplementary file 8 — Source Data 2 [file 41467_2026_72853_MOESM8_ESM.zip › Source Data MALDI Images/Supplementary Figure 12/20240627_TPITT+N3_Colon1a.png]

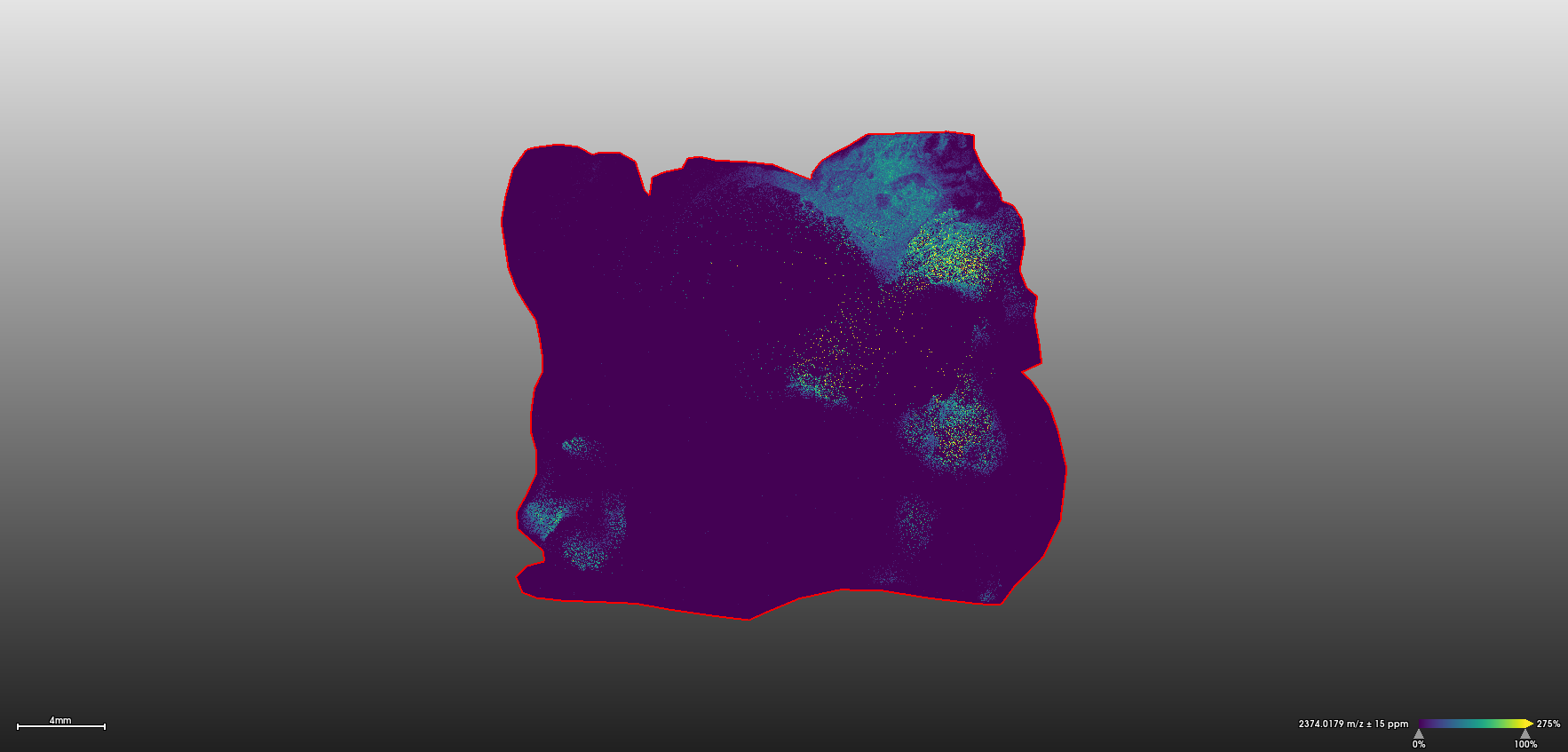

Supplement: Supplementary file 8 — Source Data 2 [file 41467_2026_72853_MOESM8_ESM.zip › Source Data MALDI Images/Supplementary Figure 12/20240524_TVTPTPTPTG+H1N6_Colon1a.png]

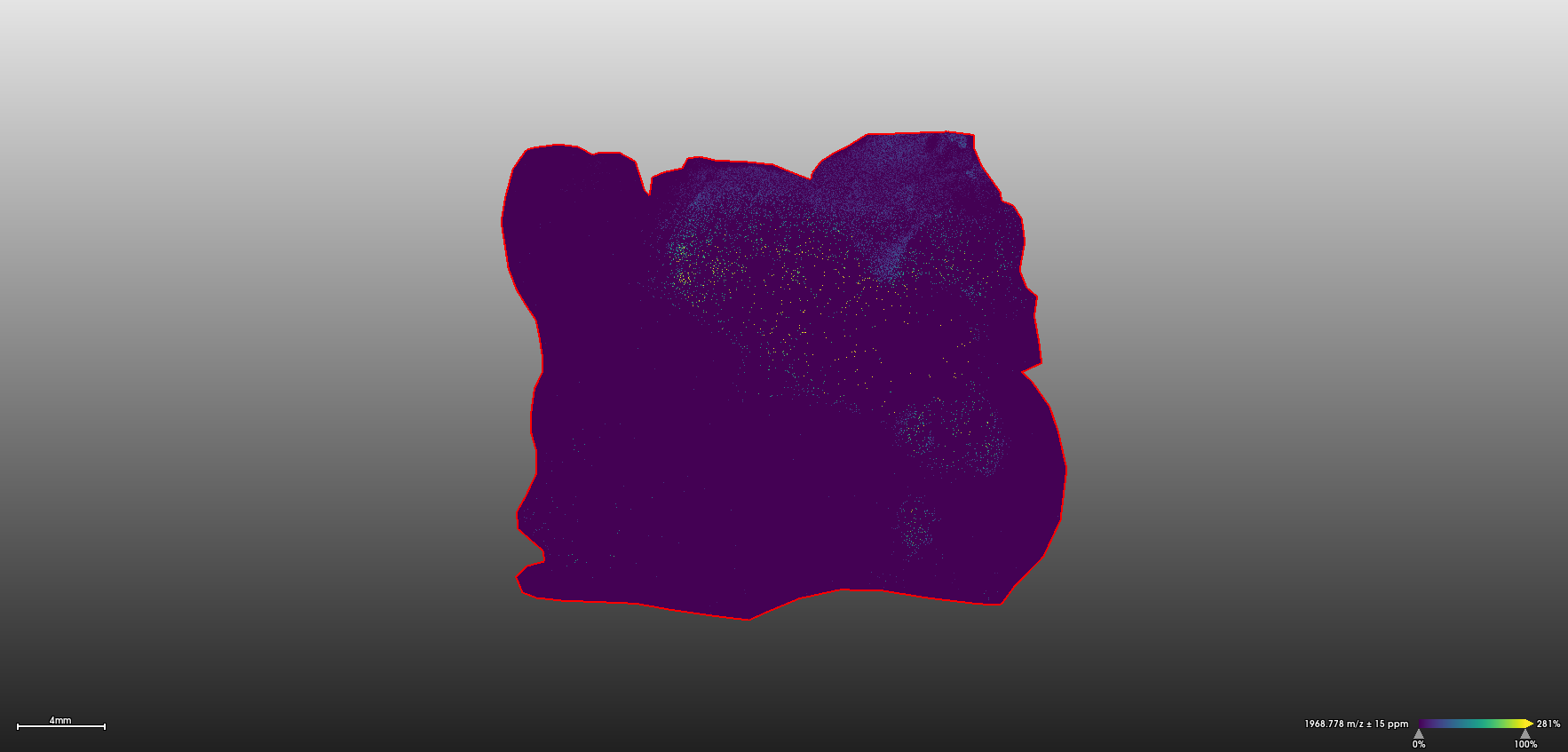

Supplement: Supplementary file 8 — Source Data 2 [file 41467_2026_72853_MOESM8_ESM.zip › Source Data MALDI Images/Supplementary Figure 12/20240531_TQTPTT+H3N4_Colon1a.png]

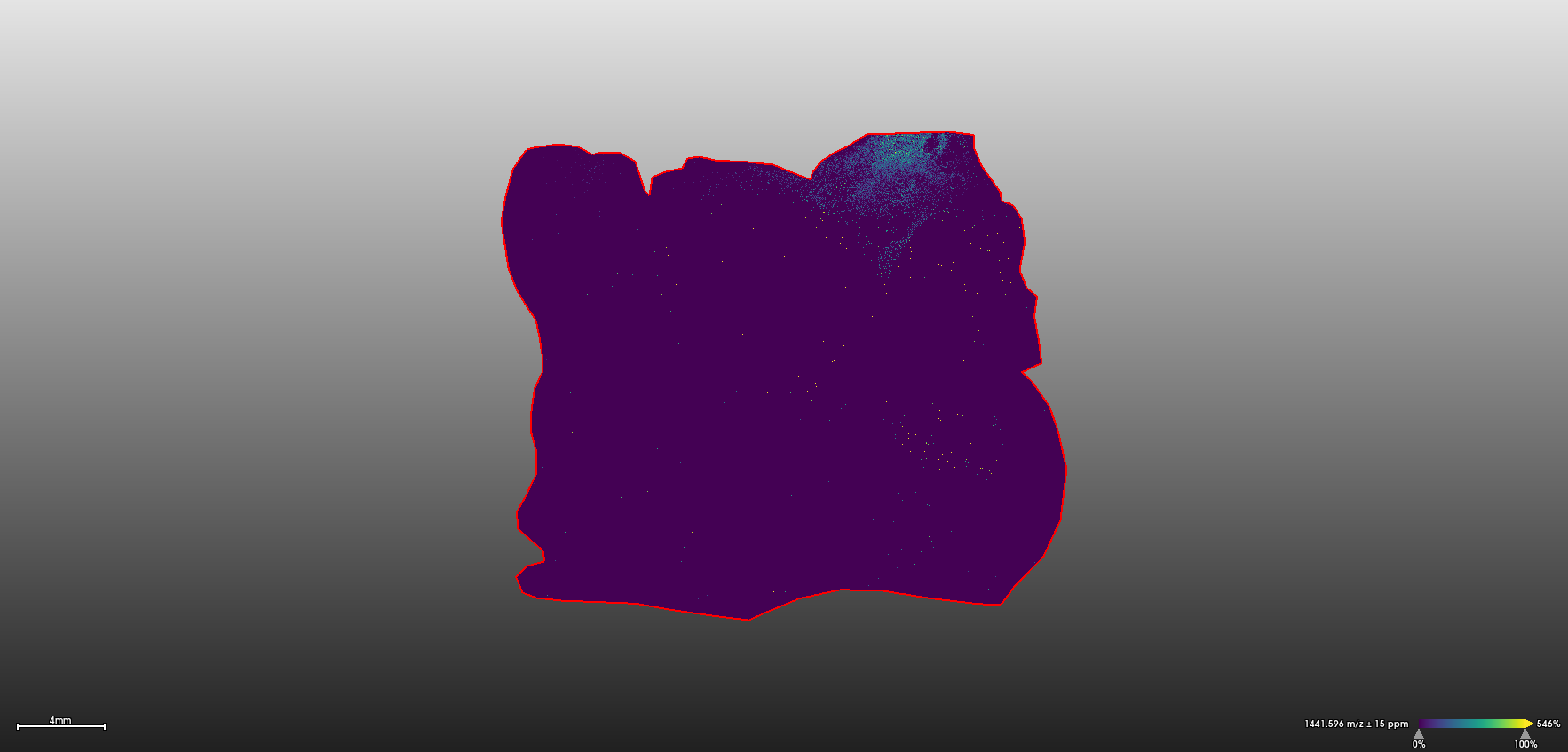

Supplement: Supplementary file 8 — Source Data 2 [file 41467_2026_72853_MOESM8_ESM.zip › Source Data MALDI Images/Supplementary Figure 12/20240531_TQTPTT+H1N3_Colon1a.png]

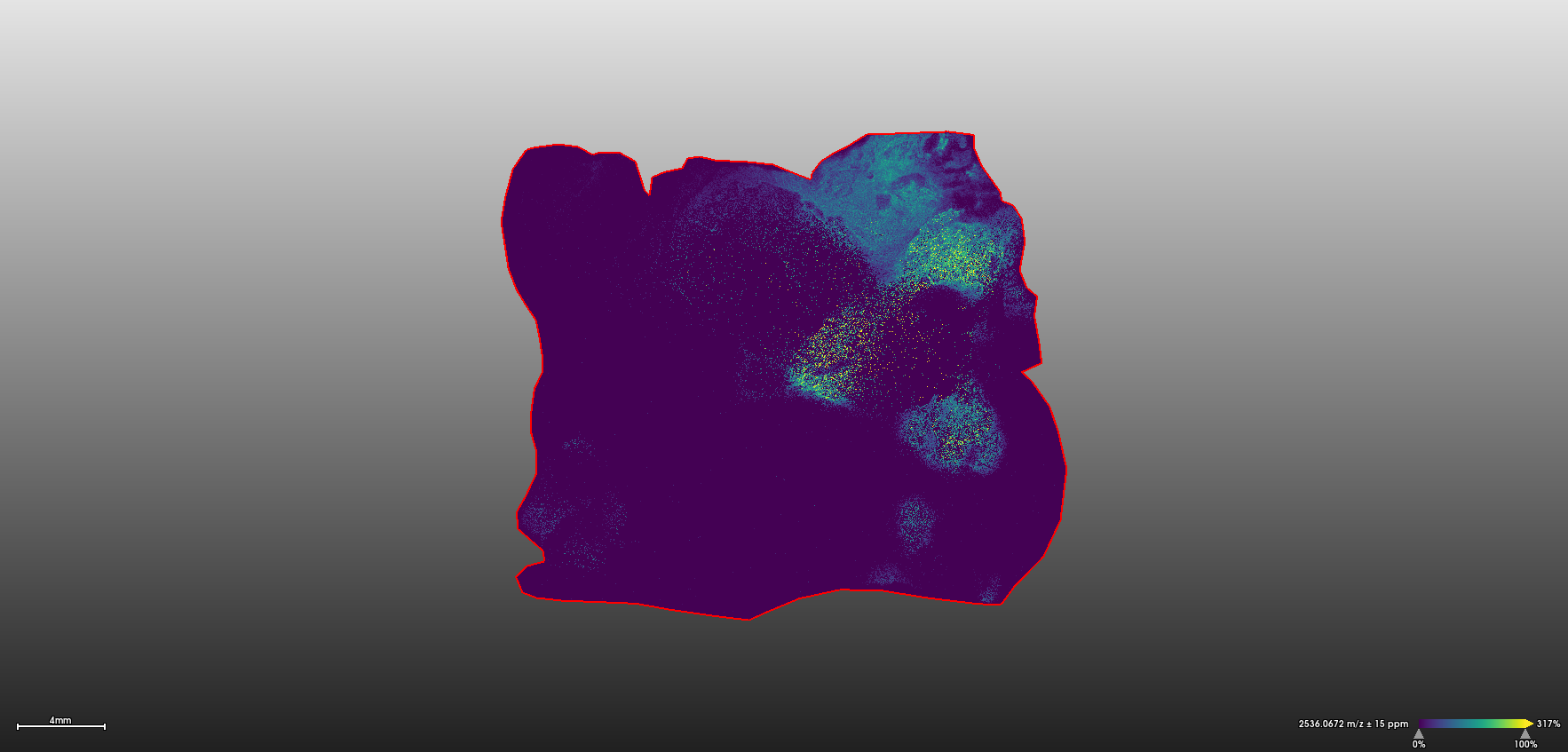

Supplement: Supplementary file 8 — Source Data 2 [file 41467_2026_72853_MOESM8_ESM.zip › Source Data MALDI Images/Supplementary Figure 12/20240524_TVTPTPTPTG+H2N6_Colon1a.png]

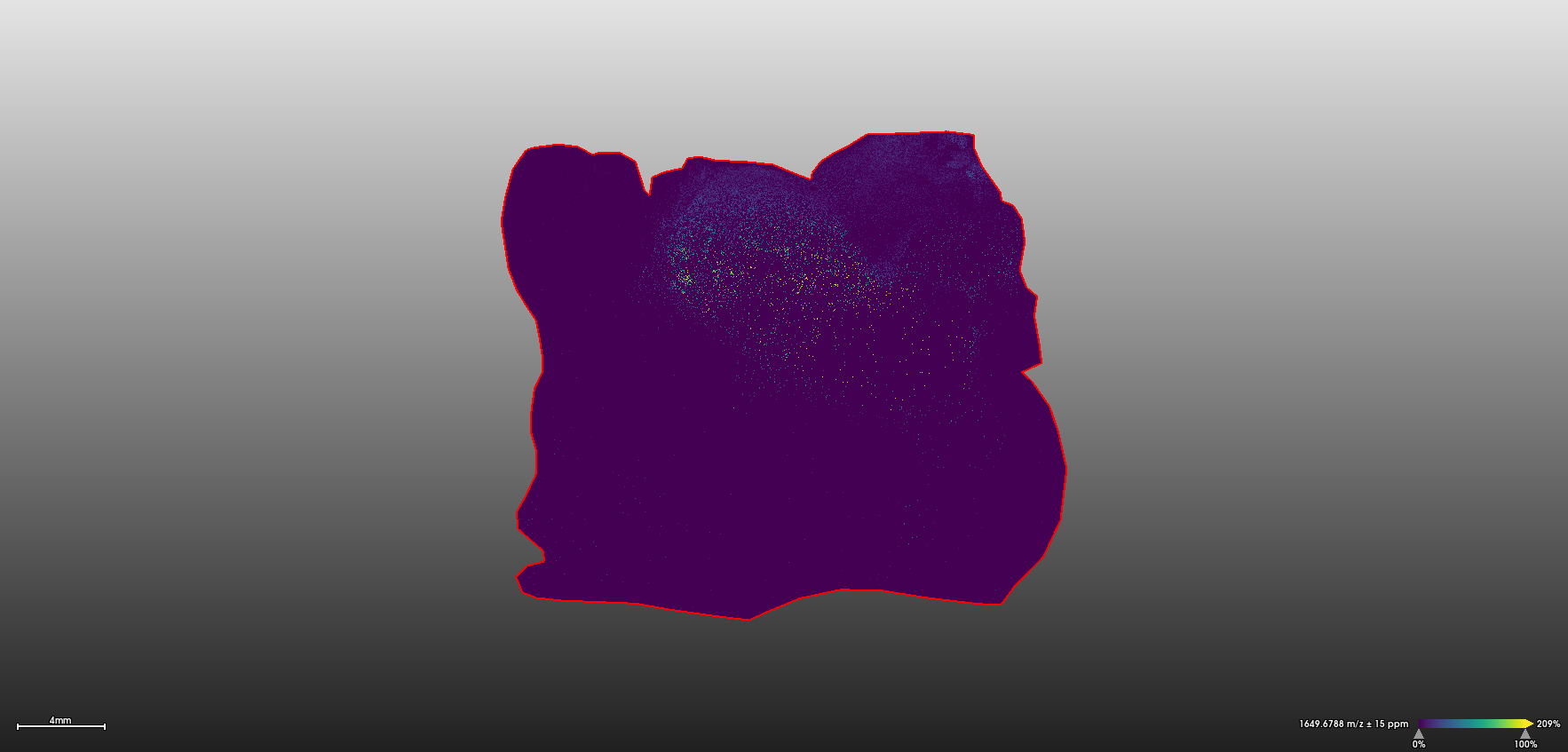

Supplement: Supplementary file 8 — Source Data 2 [file 41467_2026_72853_MOESM8_ESM.zip › Source Data MALDI Images/Supplementary Figure 12/20240627_TPITT+H3N3_Colon1a.png]

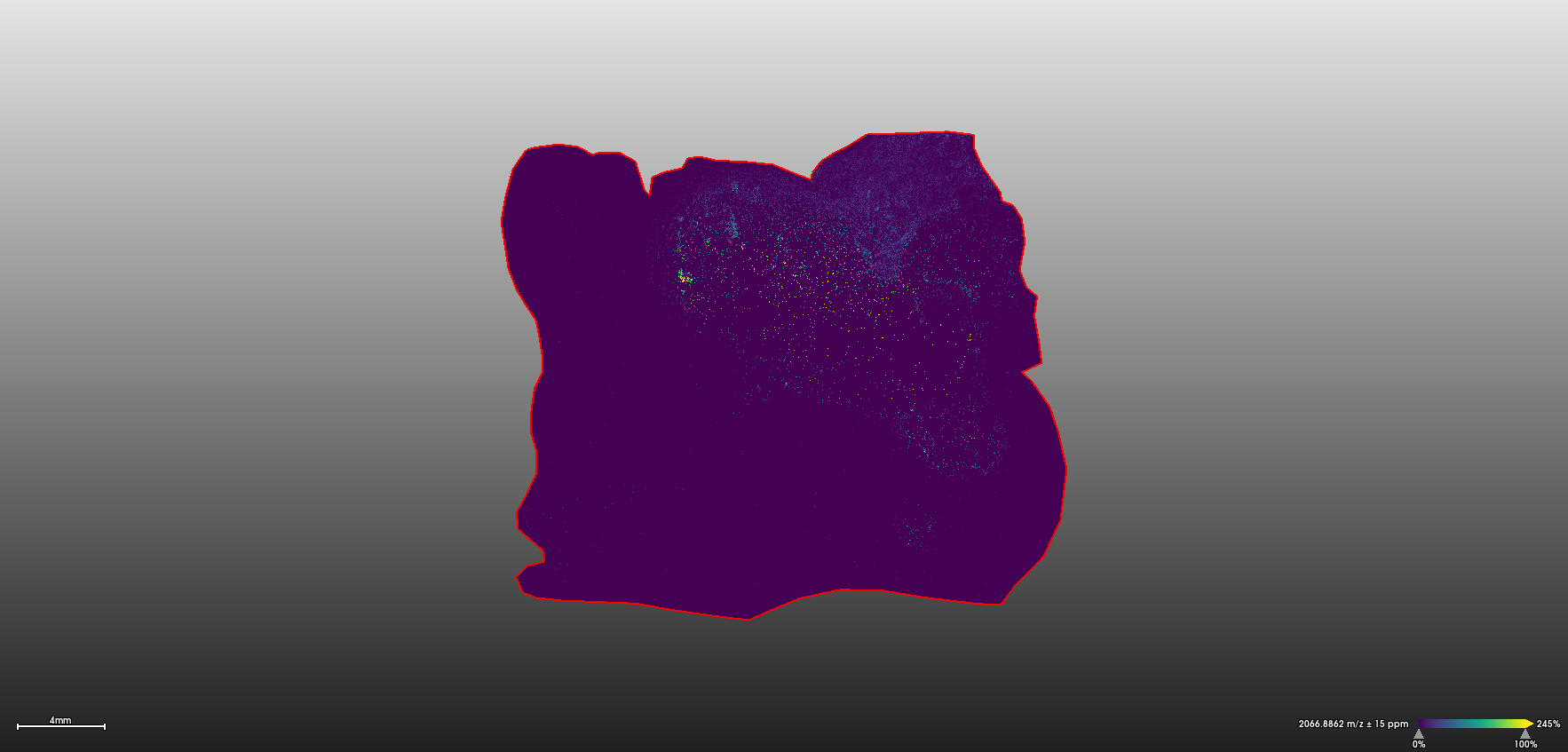

Supplement: Supplementary file 8 — Source Data 2 [file 41467_2026_72853_MOESM8_ESM.zip › Source Data MALDI Images/Supplementary Figure 12/20240627_TLTPITT+H3N4_Colon1a.png]

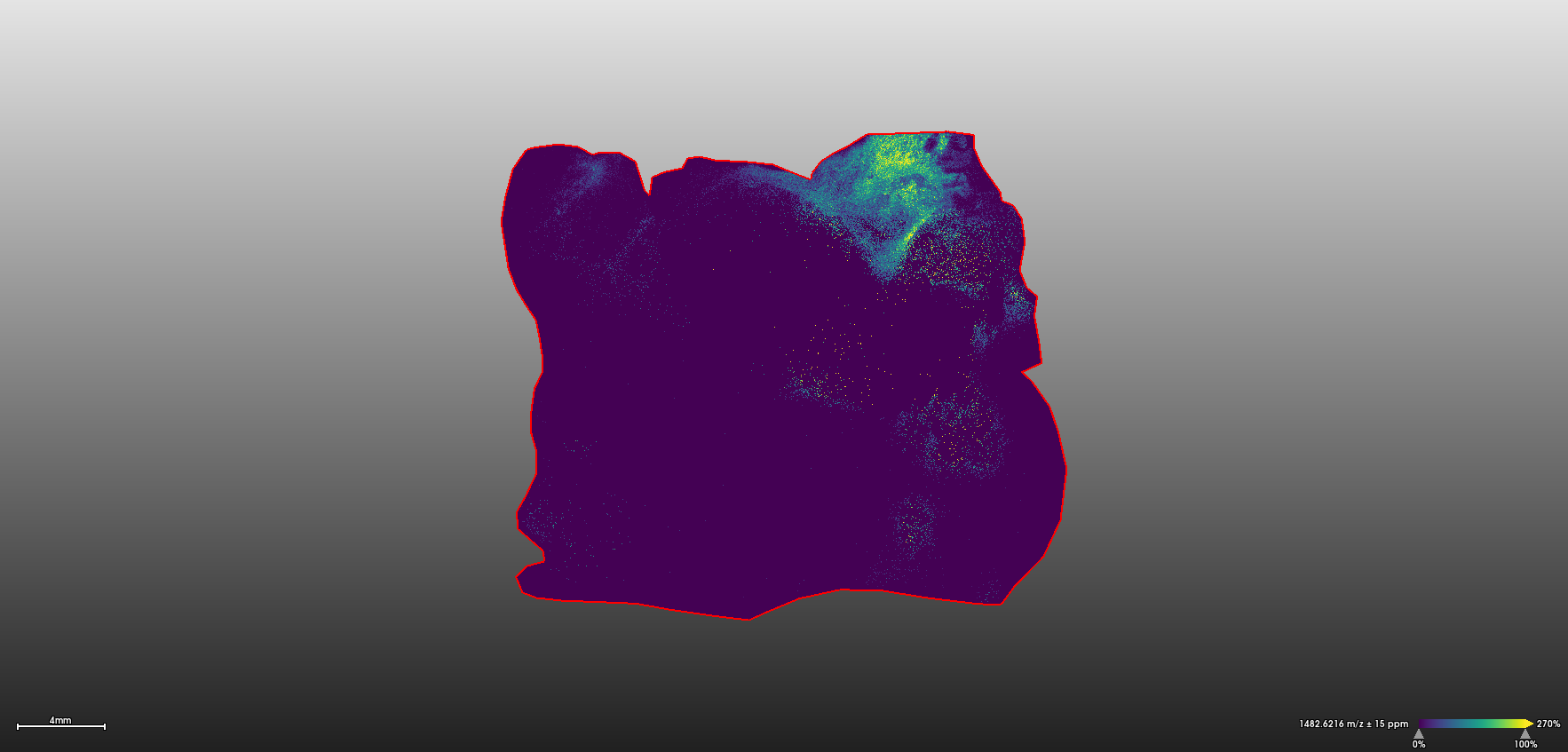

Supplement: Supplementary file 8 — Source Data 2 [file 41467_2026_72853_MOESM8_ESM.zip › Source Data MALDI Images/Supplementary Figure 12/20240531_TQTPTT+N4_Colon1a.png]

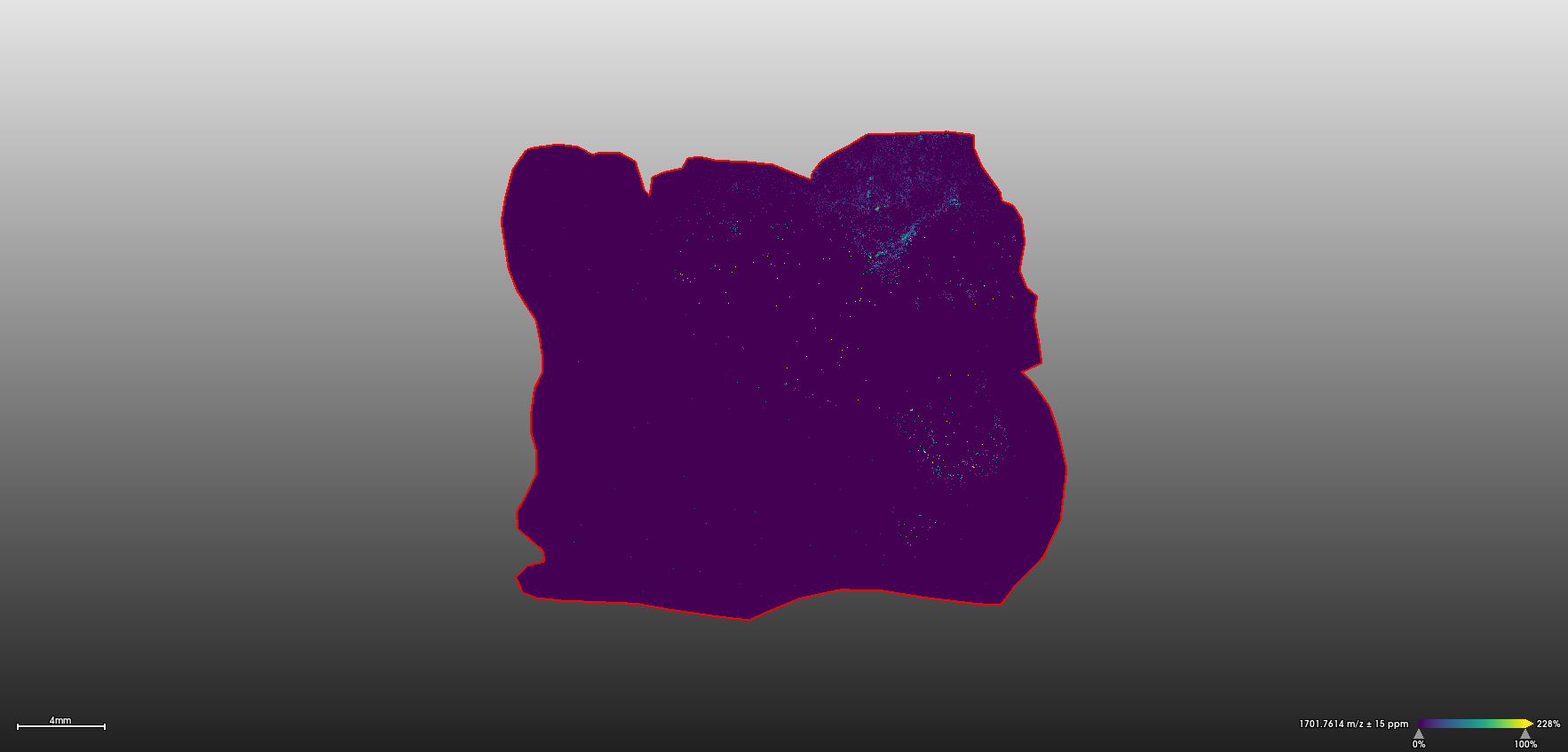

Supplement: Supplementary file 8 — Source Data 2 [file 41467_2026_72853_MOESM8_ESM.zip › Source Data MALDI Images/Supplementary Figure 12/20240627_TLTPITT+H2N3_Colon1a.png]

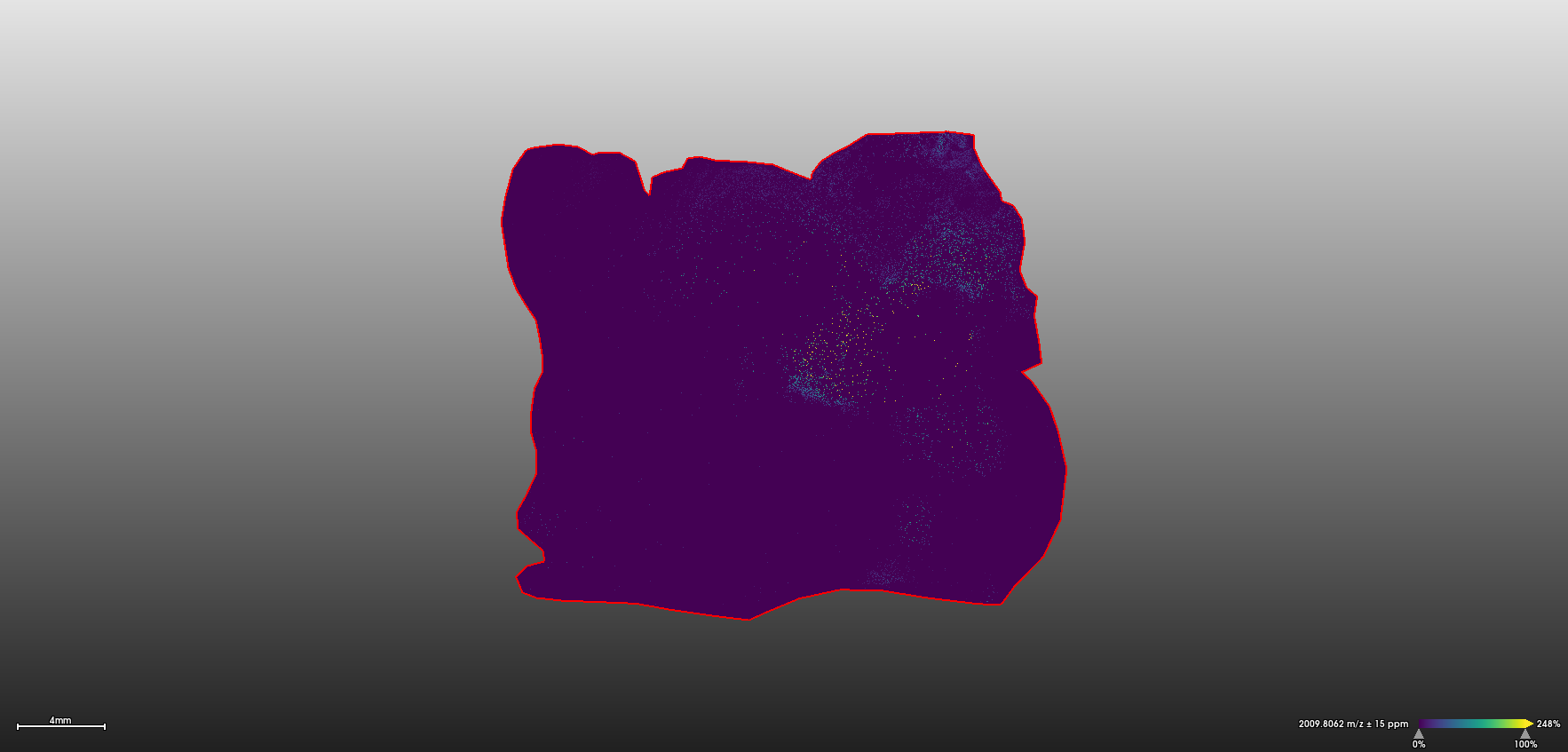

Supplement: Supplementary file 8 — Source Data 2 [file 41467_2026_72853_MOESM8_ESM.zip › Source Data MALDI Images/Supplementary Figure 12/20240531_TQTPTT+H2N5_Colon1a.png]

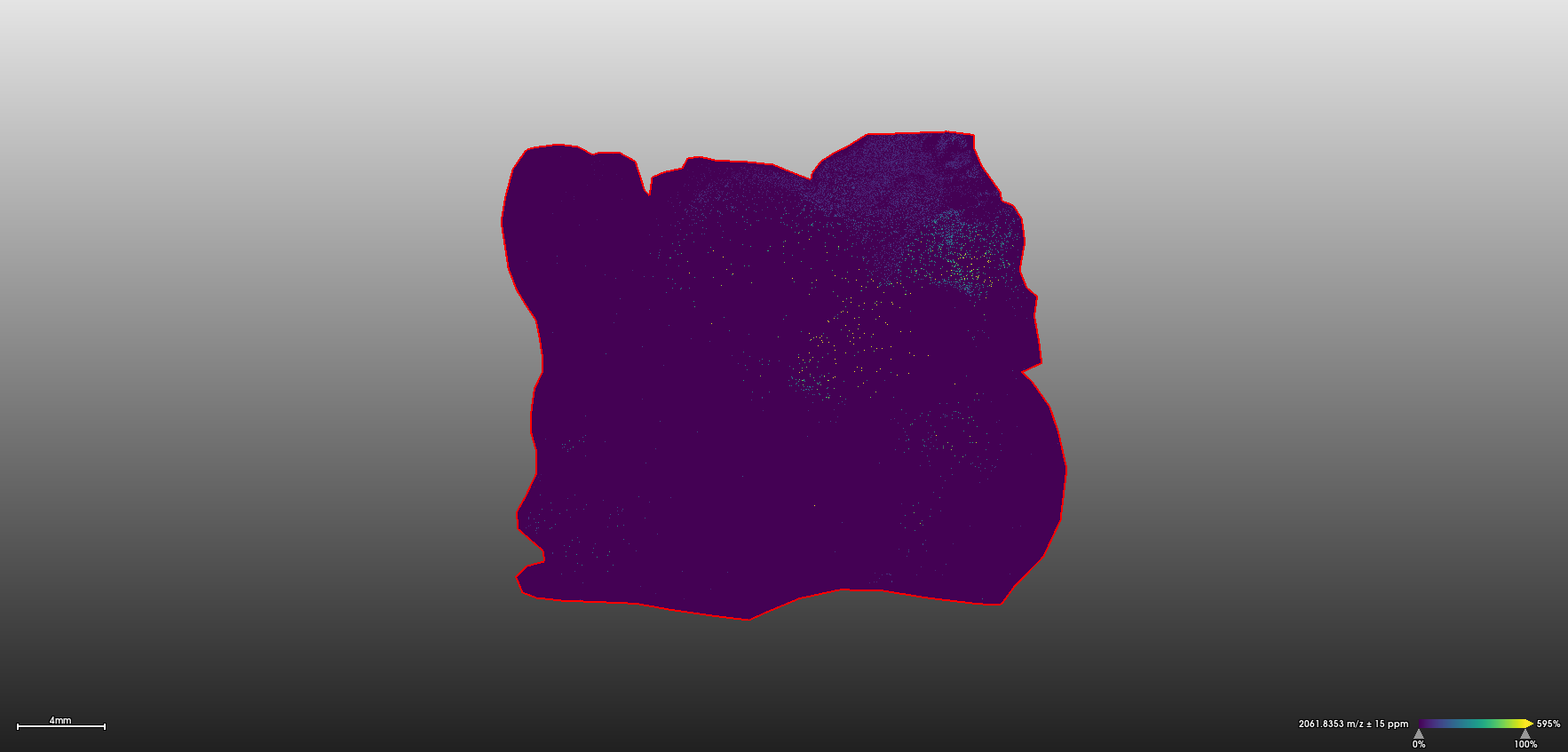

Supplement: Supplementary file 8 — Source Data 2 [file 41467_2026_72853_MOESM8_ESM.zip › Source Data MALDI Images/Supplementary Figure 12/20240627_TPSPPTT+H2N5_Colon1a.png]

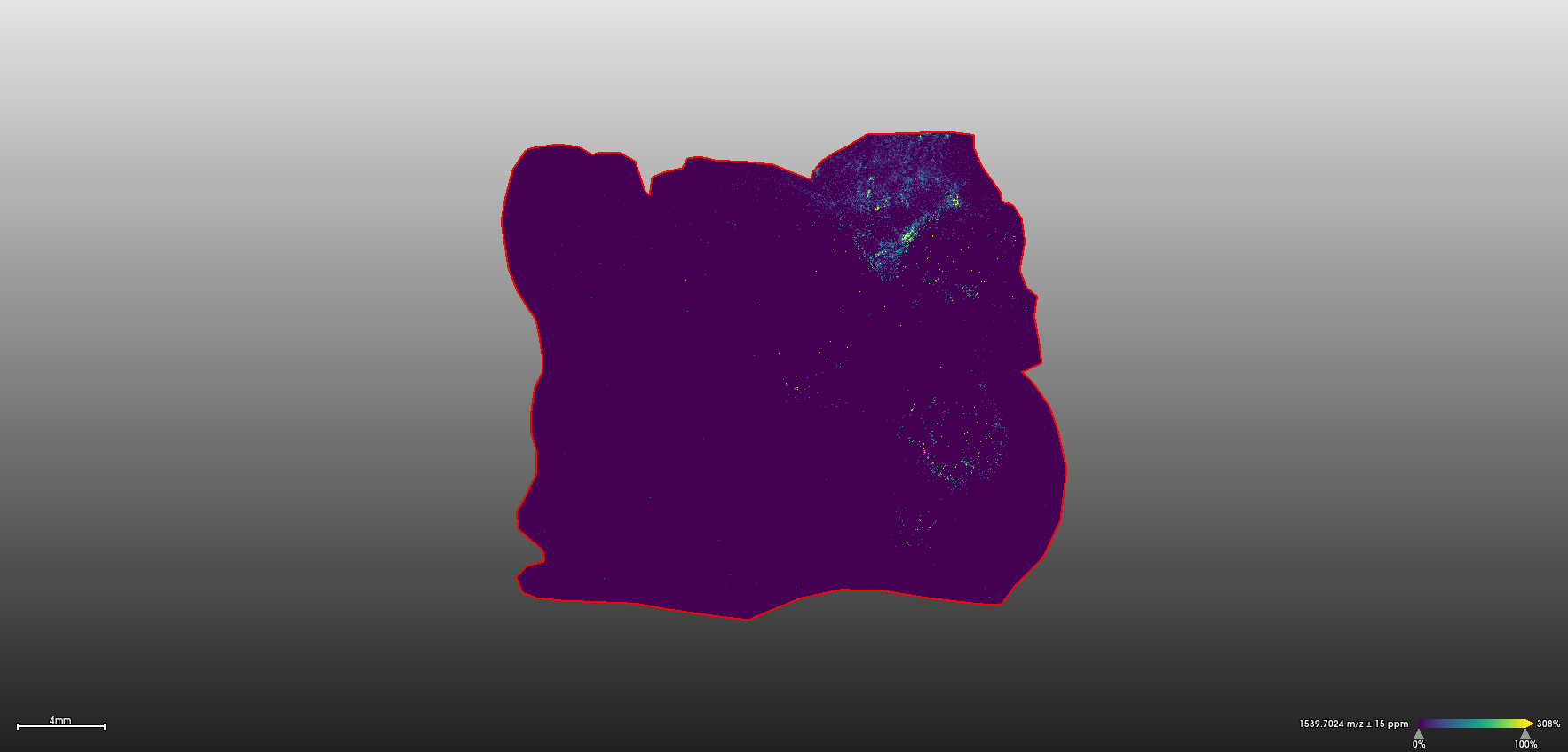

Supplement: Supplementary file 8 — Source Data 2 [file 41467_2026_72853_MOESM8_ESM.zip › Source Data MALDI Images/Supplementary Figure 12/20240627_TLTPITT+H1N3_Colon1a.png]

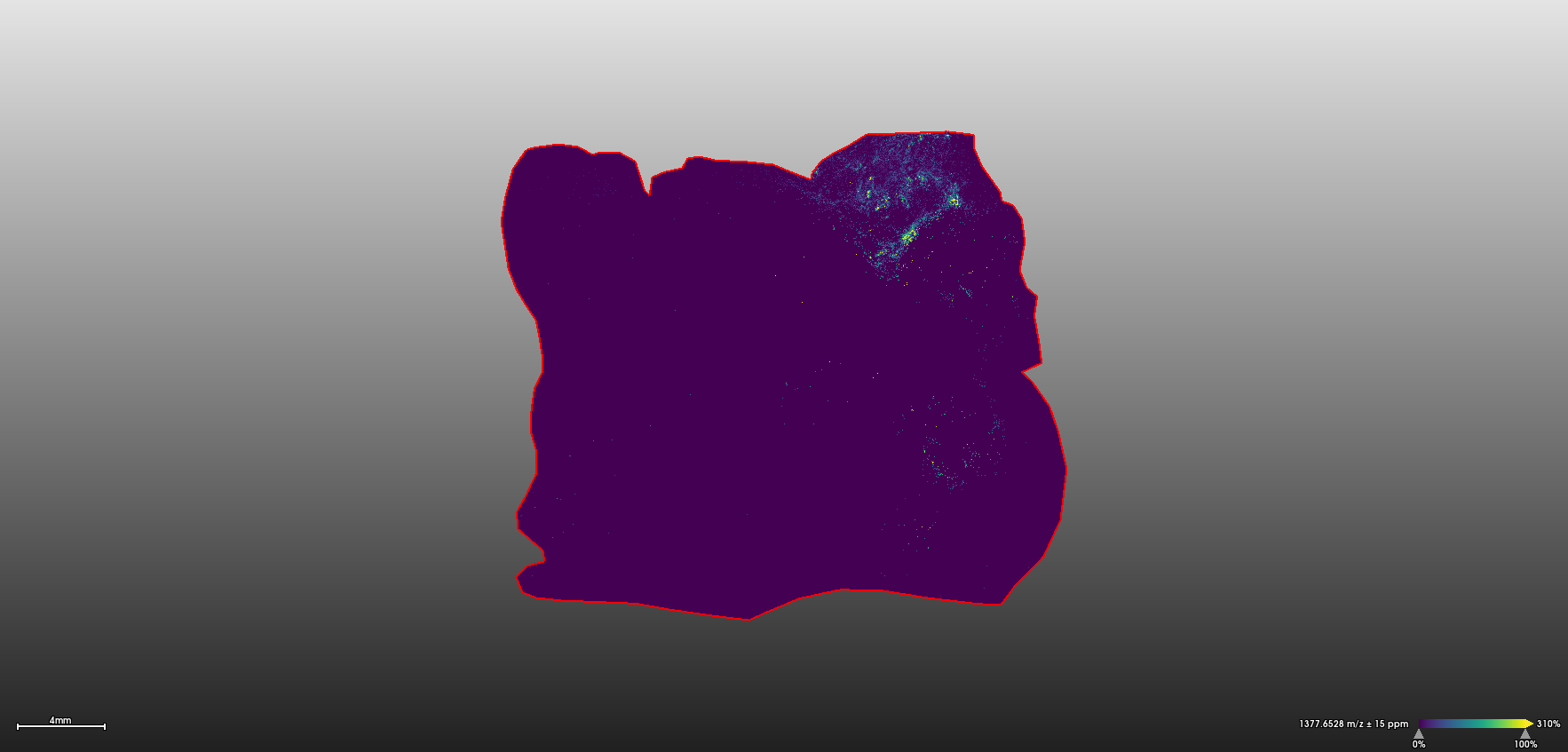

Supplement: Supplementary file 8 — Source Data 2 [file 41467_2026_72853_MOESM8_ESM.zip › Source Data MALDI Images/Supplementary Figure 12/20240627_TLTPITT+N3_Colon1a.png]

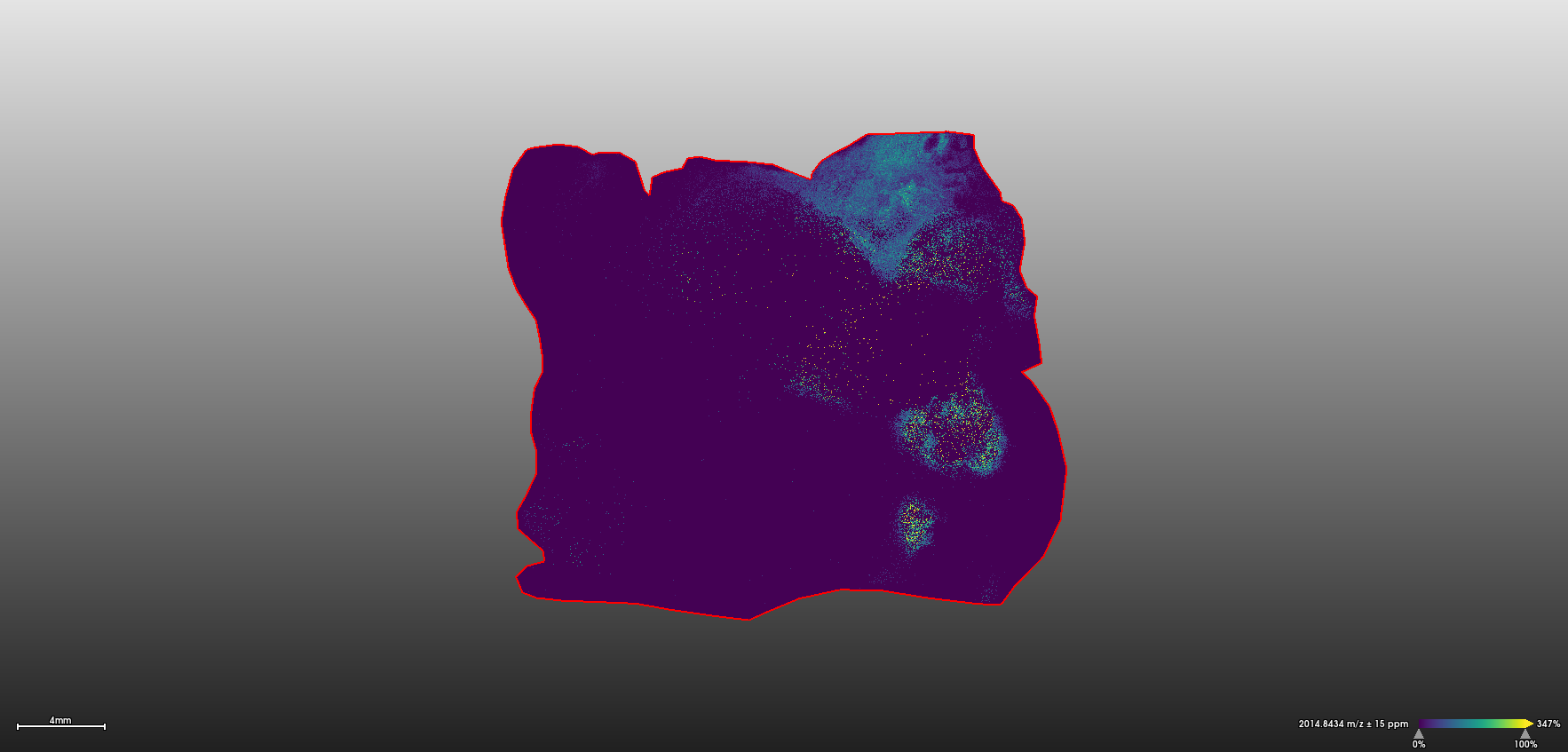

Supplement: Supplementary file 8 — Source Data 2 [file 41467_2026_72853_MOESM8_ESM.zip › Source Data MALDI Images/Supplementary Figure 12/20240531_TTTPPPTT+H1N5_Colon1a.png]

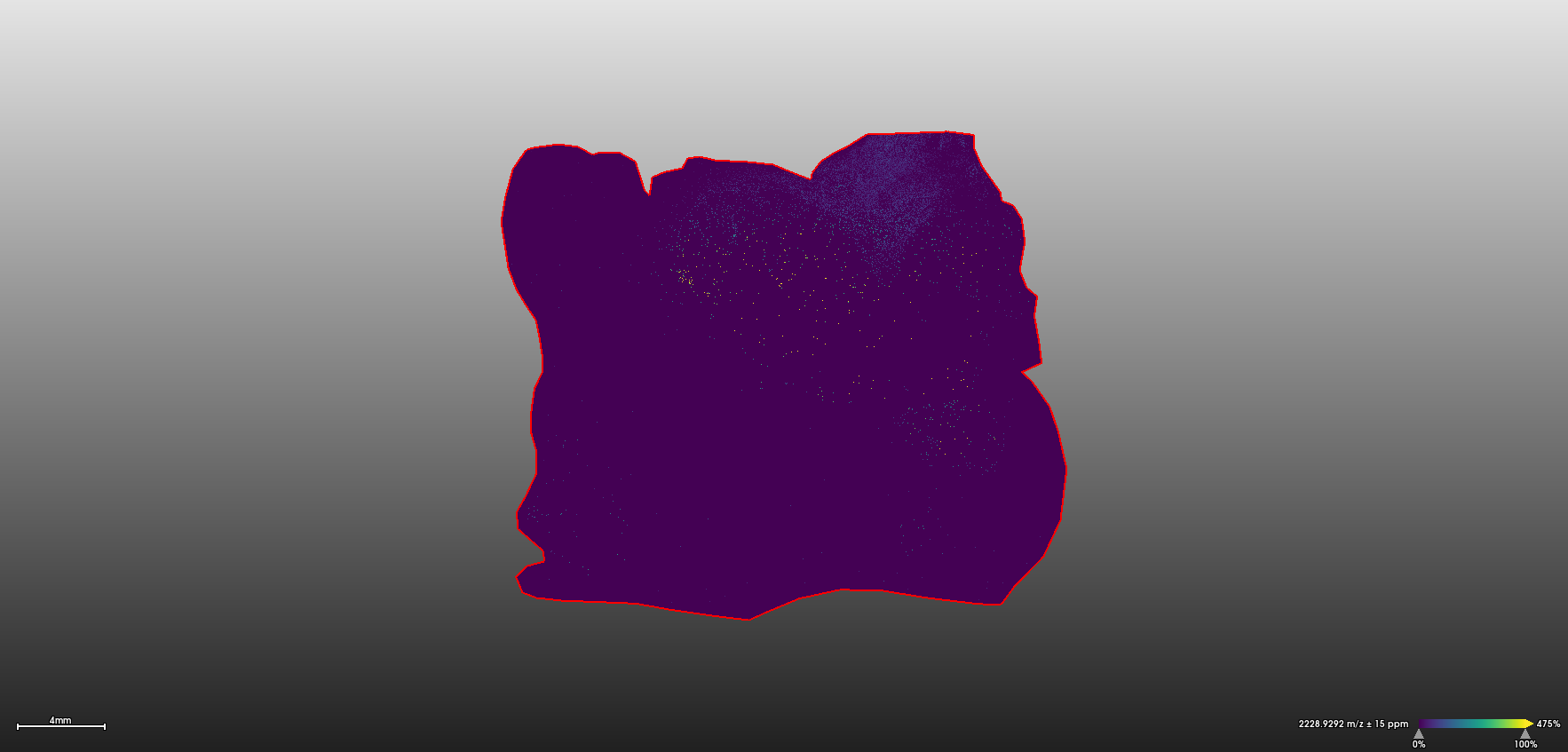

Supplement: Supplementary file 8 — Source Data 2 [file 41467_2026_72853_MOESM8_ESM.zip › Source Data MALDI Images/Supplementary Figure 12/20240627_TLTPITT+H4N4_Colon1a.png]

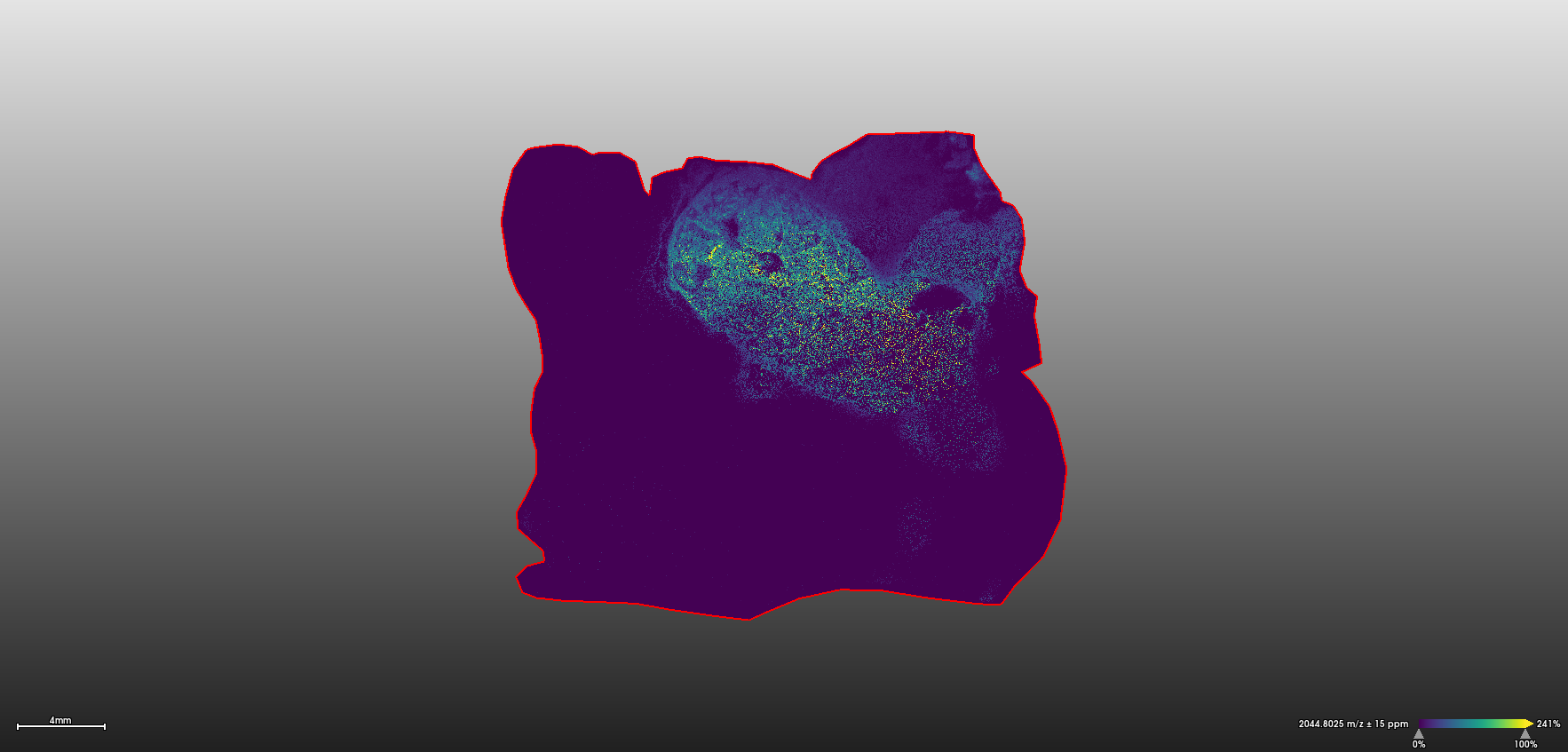

Supplement: Supplementary file 8 — Source Data 2 [file 41467_2026_72853_MOESM8_ESM.zip › Source Data MALDI Images/Supplementary Figure 12/20240531_TPPTF+H4N4_Colon1a.png]

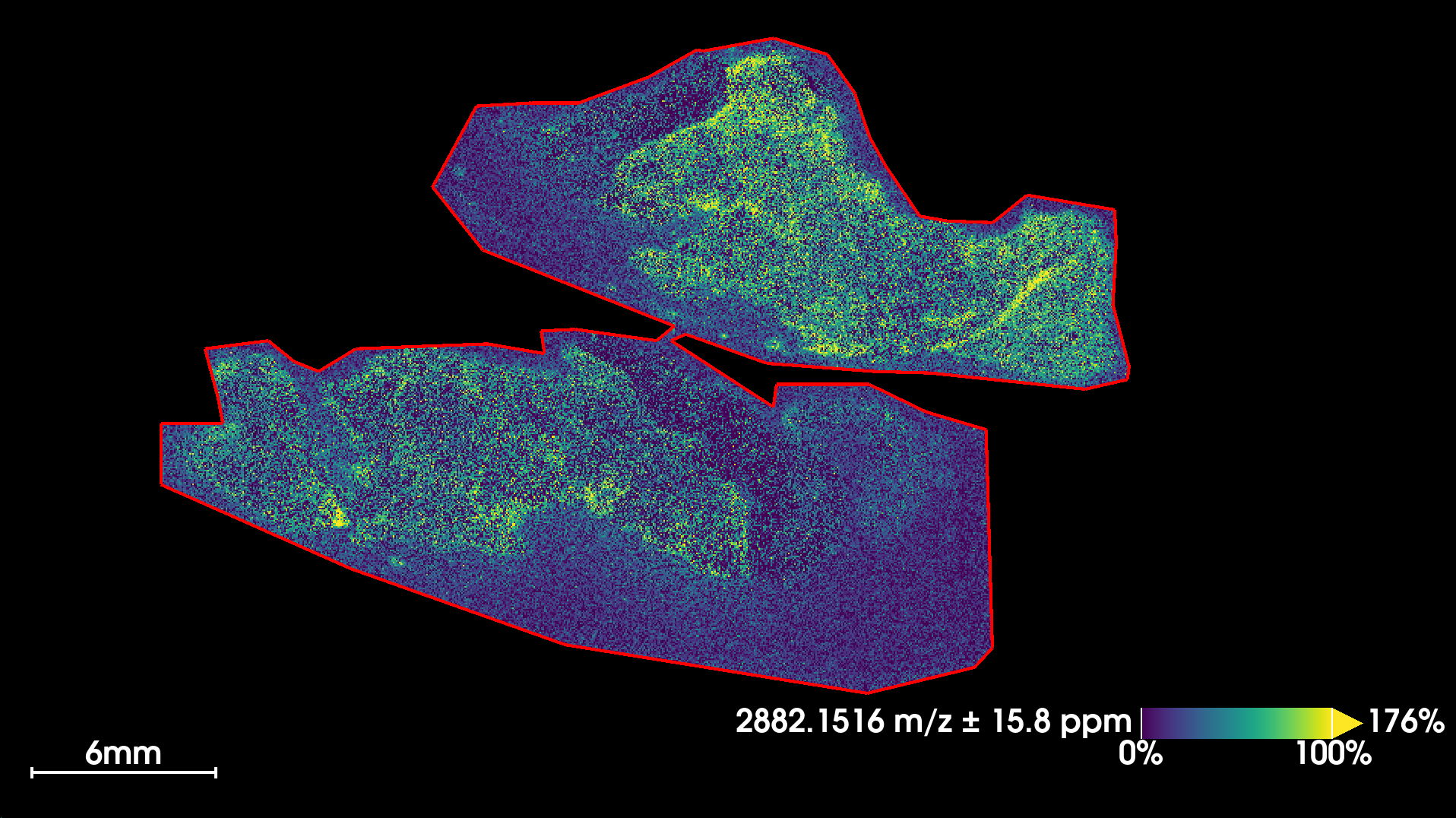

Supplement: Supplementary file 8 — Source Data 2 [file 41467_2026_72853_MOESM8_ESM.zip › Source Data MALDI Images/Supplementary Figure 15/20250822_mz2882_Colon2.png]

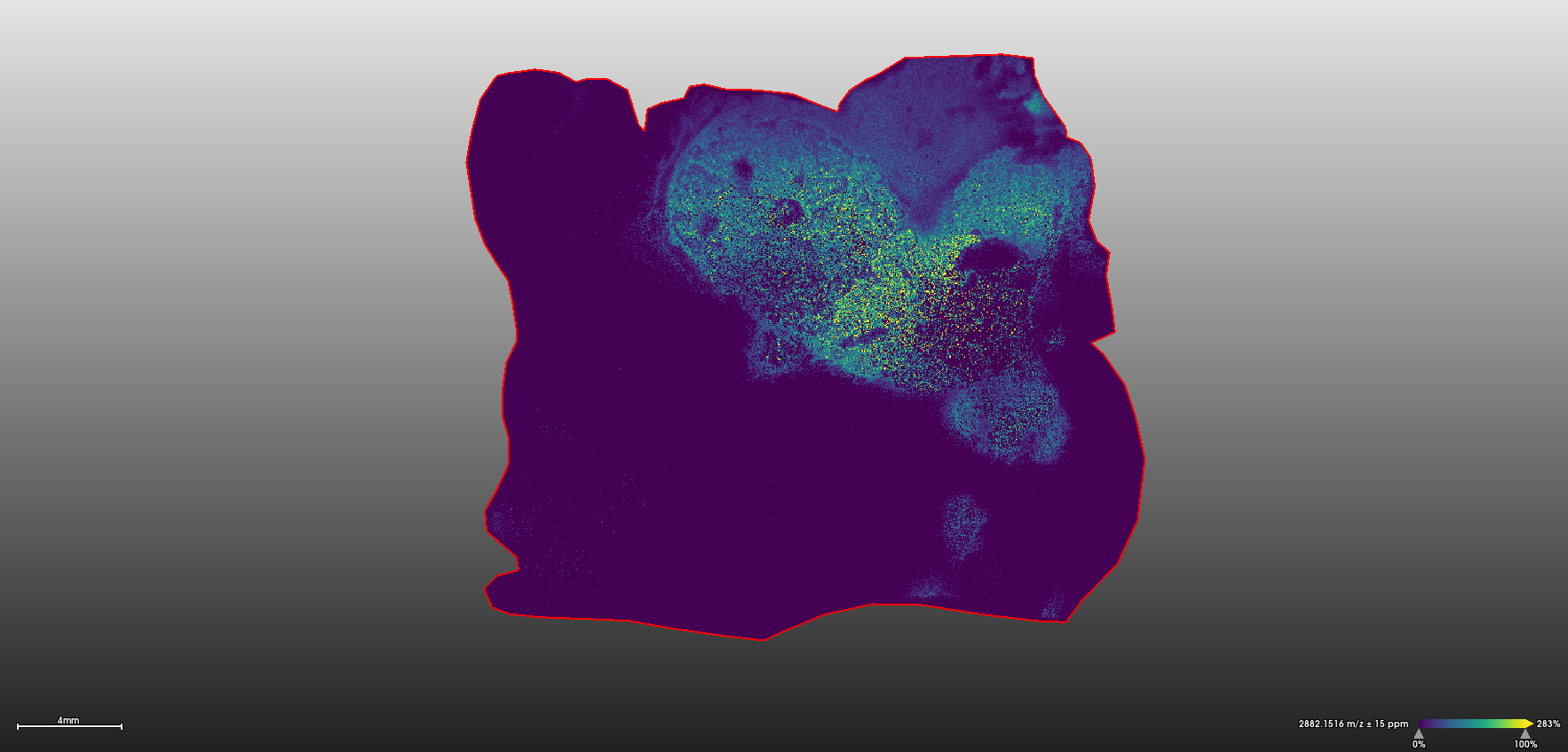

Supplement: Supplementary file 8 — Source Data 2 [file 41467_2026_72853_MOESM8_ESM.zip › Source Data MALDI Images/Supplementary Figure 15/20240524_mz2882_Colon1a.png]

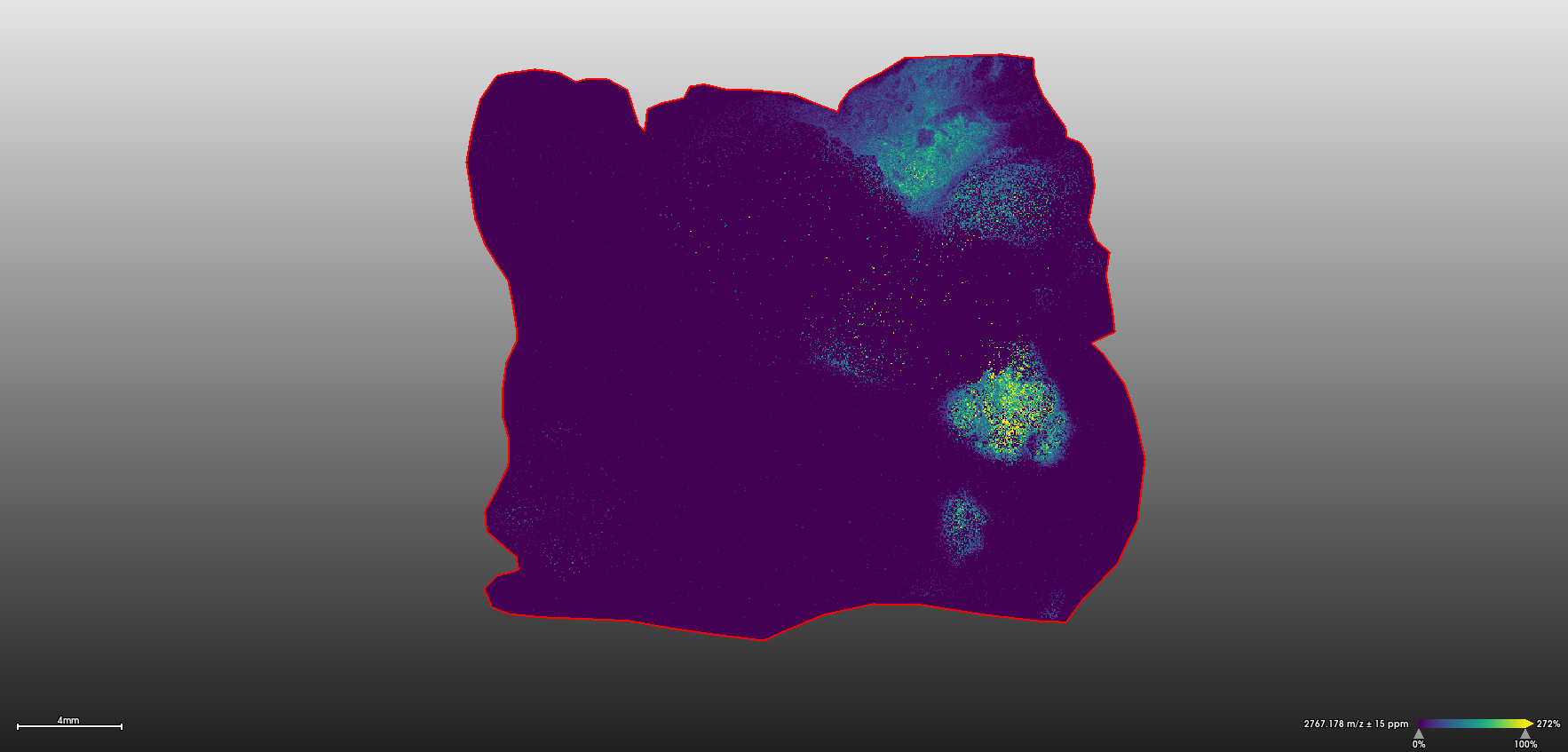

Supplement: Supplementary file 8 — Source Data 2 [file 41467_2026_72853_MOESM8_ESM.zip › Source Data MALDI Images/Supplementary Figure 15/20240524_mz2767_Colon1a.png]

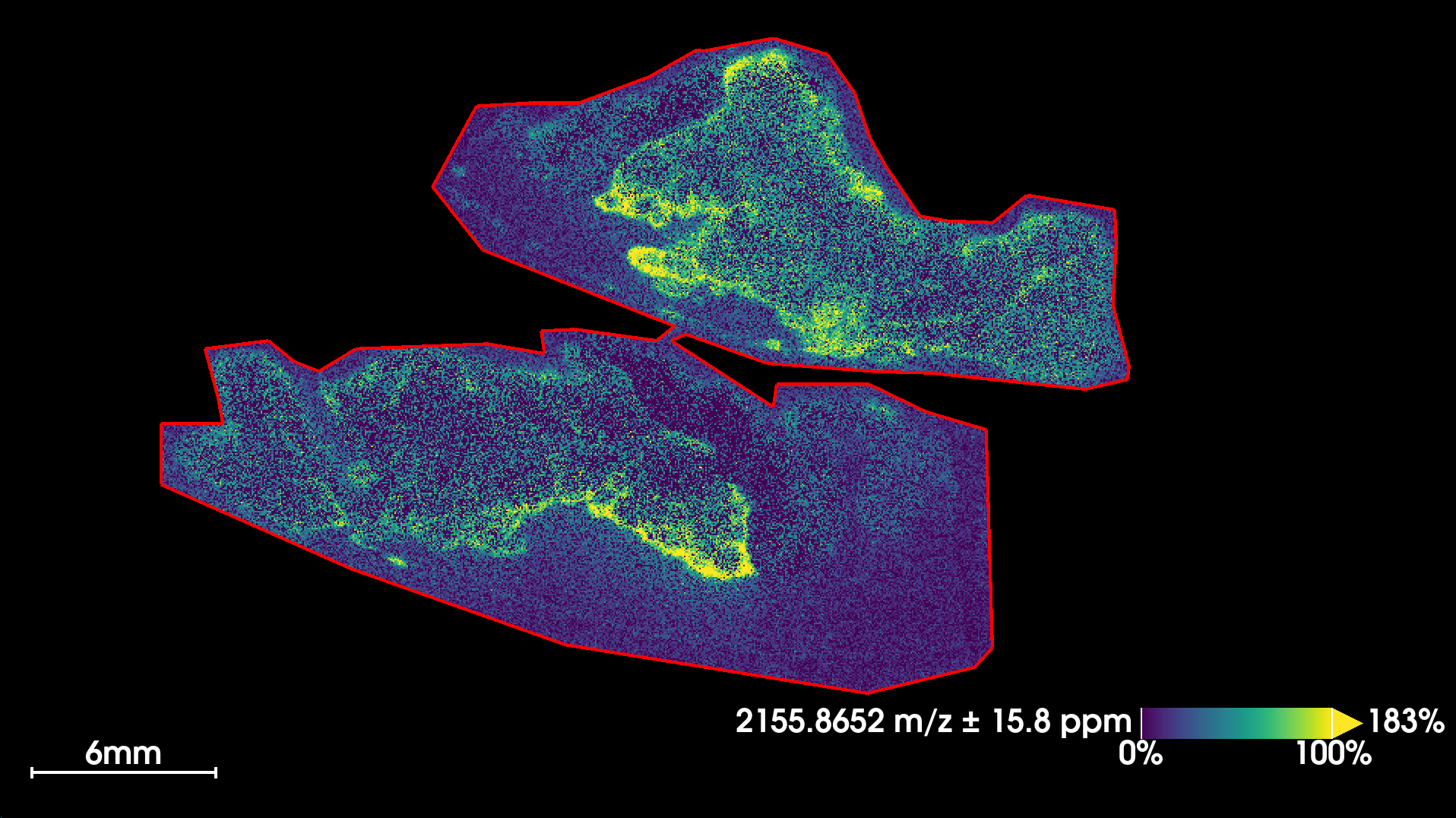

Supplement: Supplementary file 8 — Source Data 2 [file 41467_2026_72853_MOESM8_ESM.zip › Source Data MALDI Images/Supplementary Figure 15/20250822_mz2155_Colon2.png]

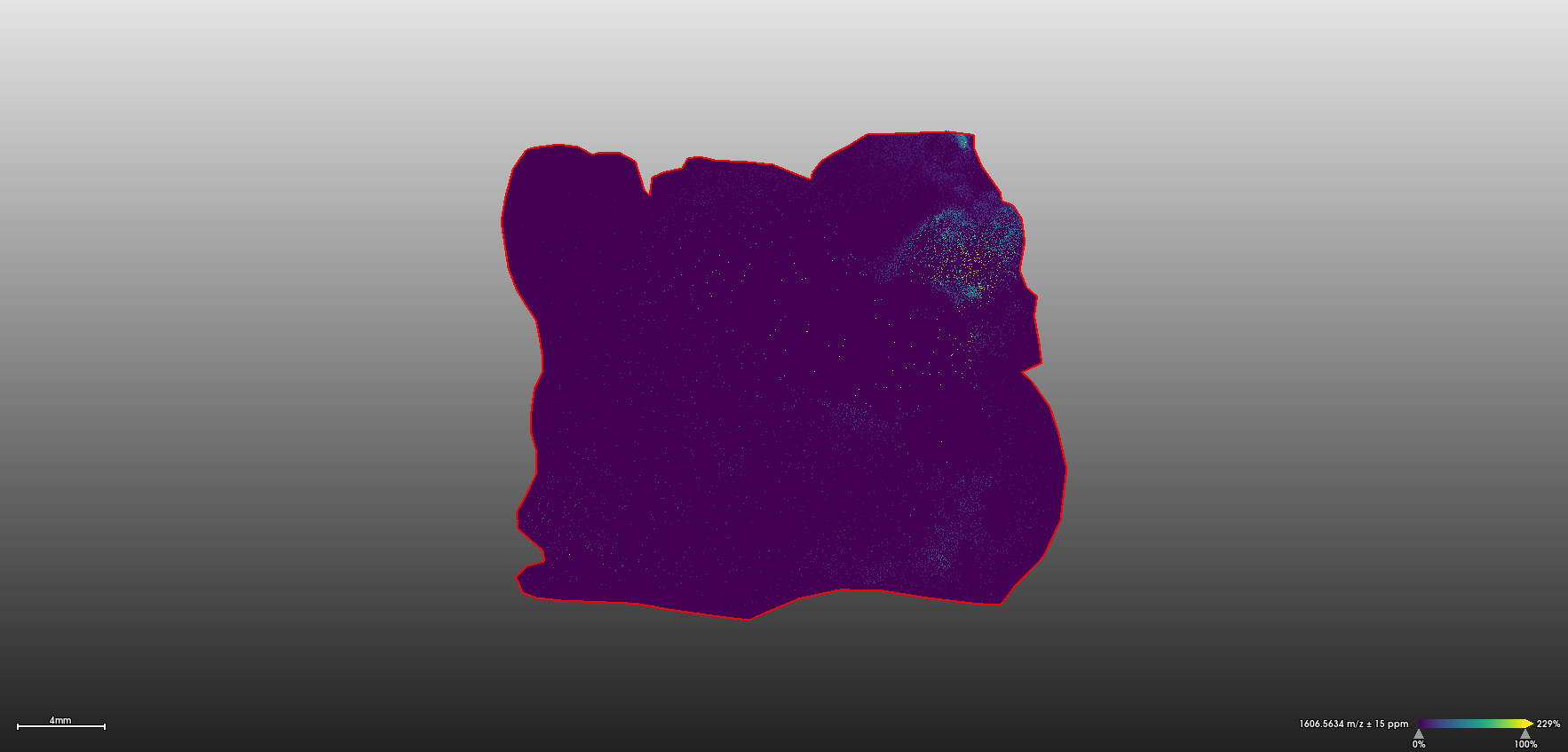

Supplement: Supplementary file 8 — Source Data 2 [file 41467_2026_72853_MOESM8_ESM.zip › Source Data MALDI Images/Supplementary Figure 15/20240531_mz1606_Colon1a.png]

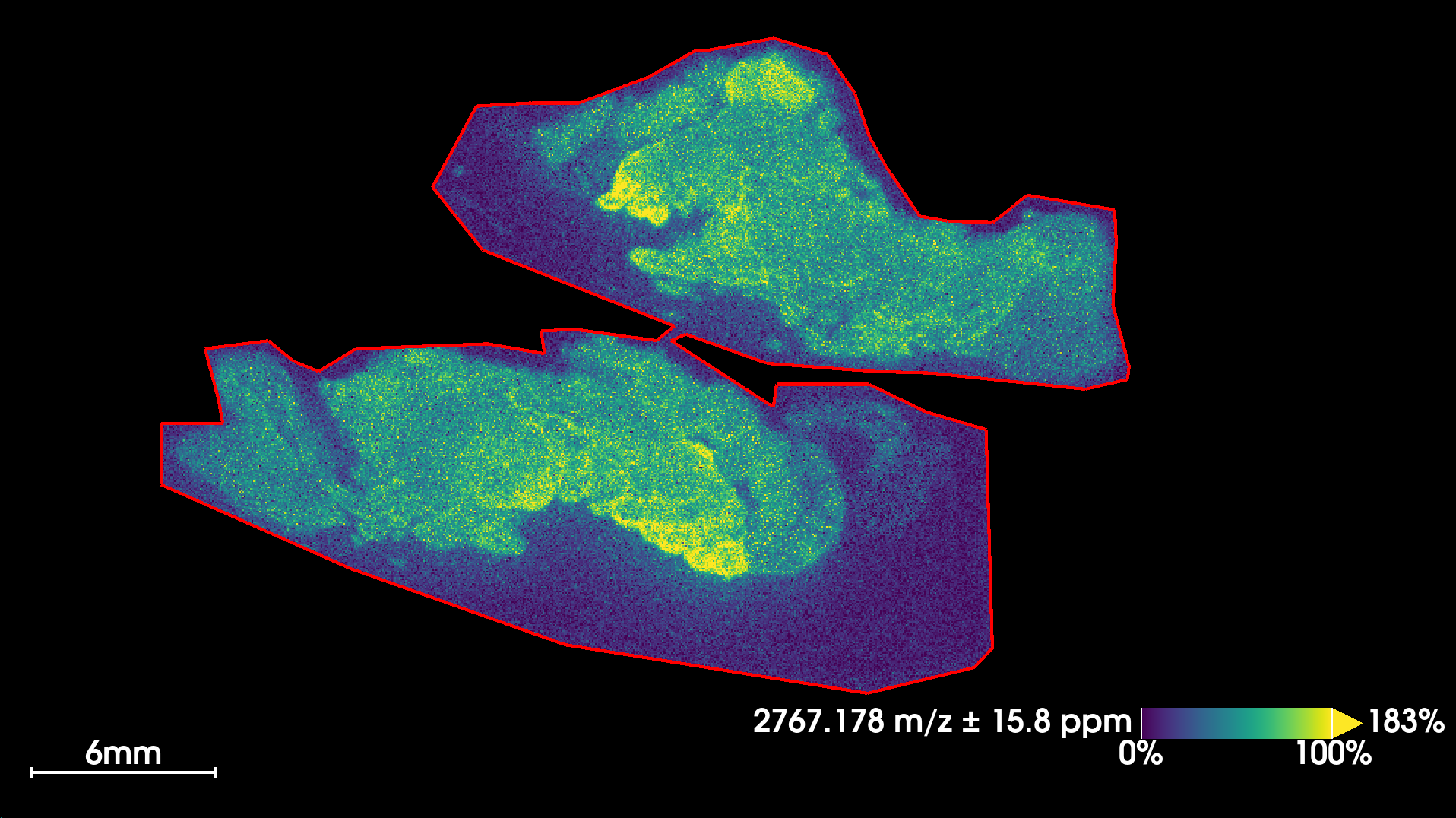

Supplement: Supplementary file 8 — Source Data 2 [file 41467_2026_72853_MOESM8_ESM.zip › Source Data MALDI Images/Supplementary Figure 15/20250822_mz2767_Colon2.png]

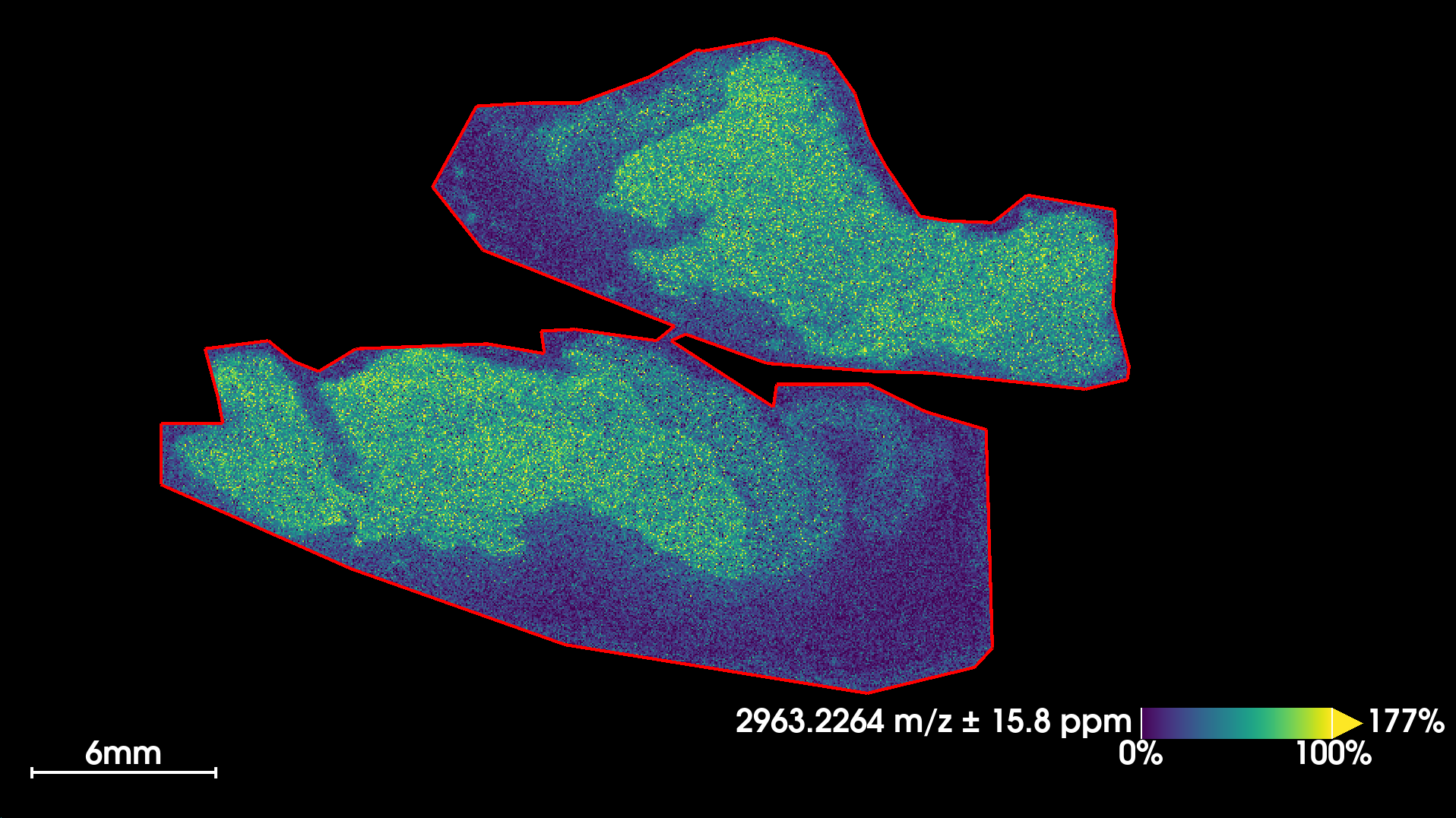

Supplement: Supplementary file 8 — Source Data 2 [file 41467_2026_72853_MOESM8_ESM.zip › Source Data MALDI Images/Supplementary Figure 15/20250822_mz2963_Colon2.png]

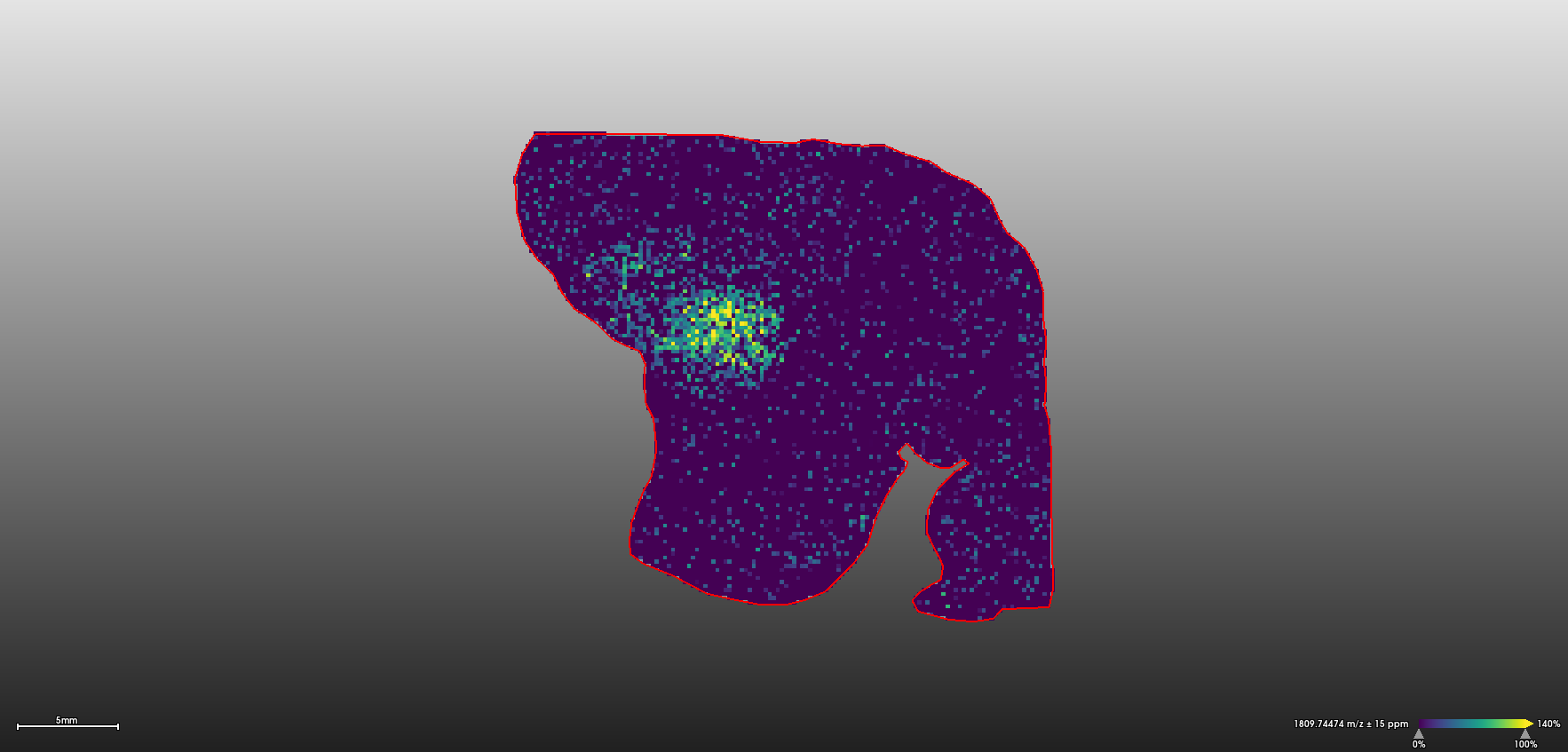

Supplement: Supplementary file 8 — Source Data 2 [file 41467_2026_72853_MOESM8_ESM.zip › Source Data MALDI Images/Supplementary Figure 15/20240531_mz1809_Colon1b.png]

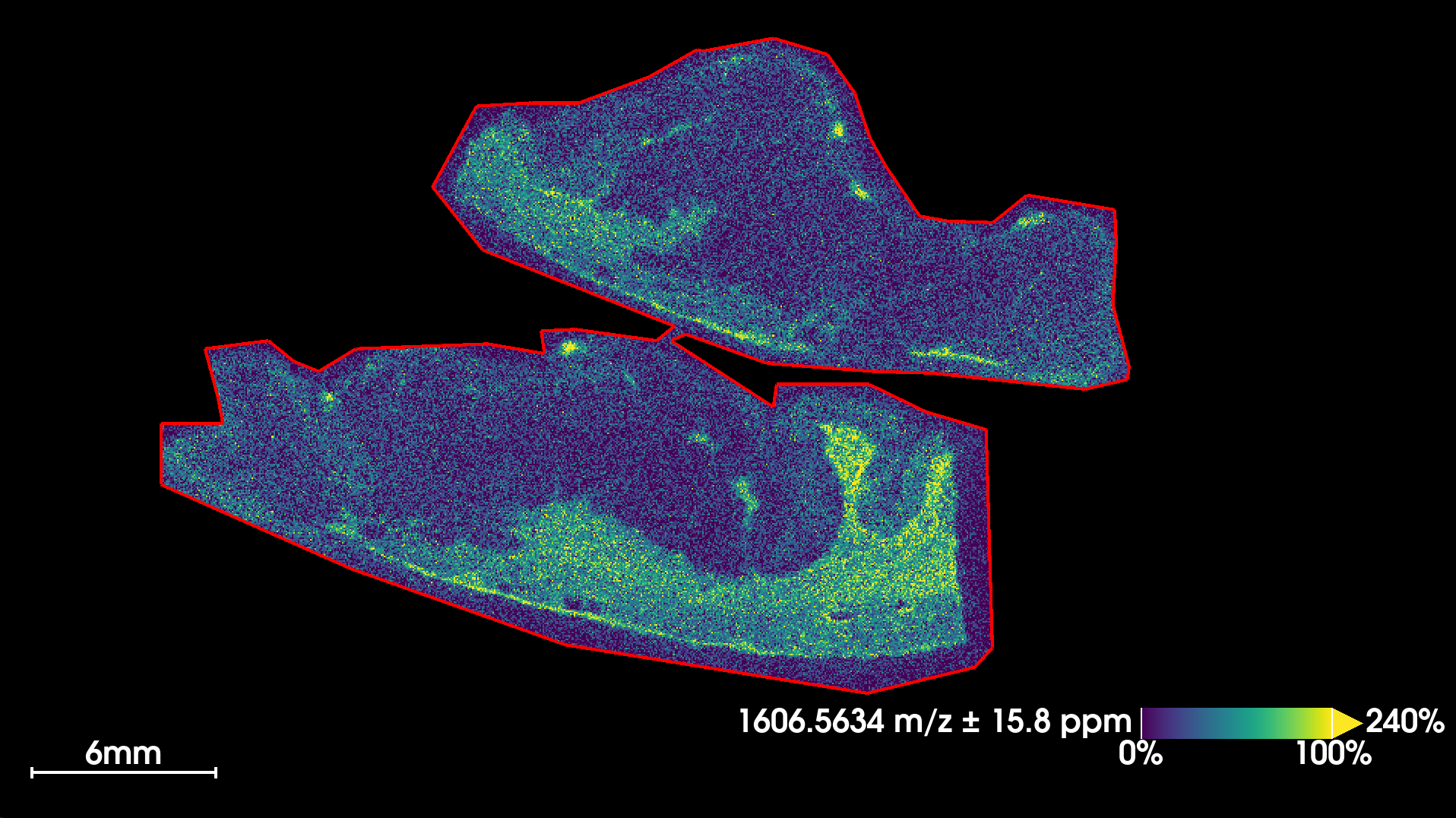

Supplement: Supplementary file 8 — Source Data 2 [file 41467_2026_72853_MOESM8_ESM.zip › Source Data MALDI Images/Supplementary Figure 15/20250822_mz1606_Colon2.png]

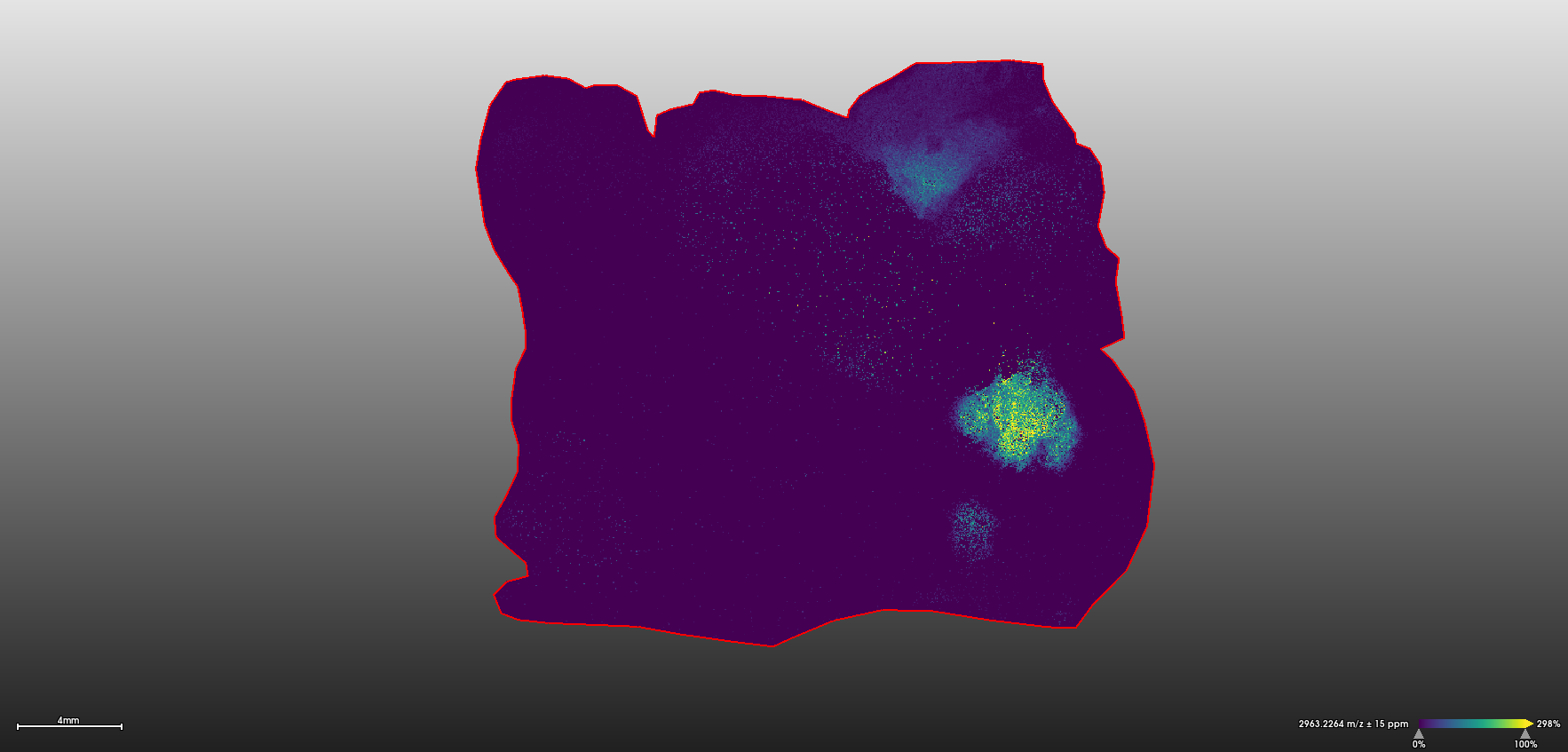

Supplement: Supplementary file 8 — Source Data 2 [file 41467_2026_72853_MOESM8_ESM.zip › Source Data MALDI Images/Supplementary Figure 15/20240524_mz2963_Colon1a.png]

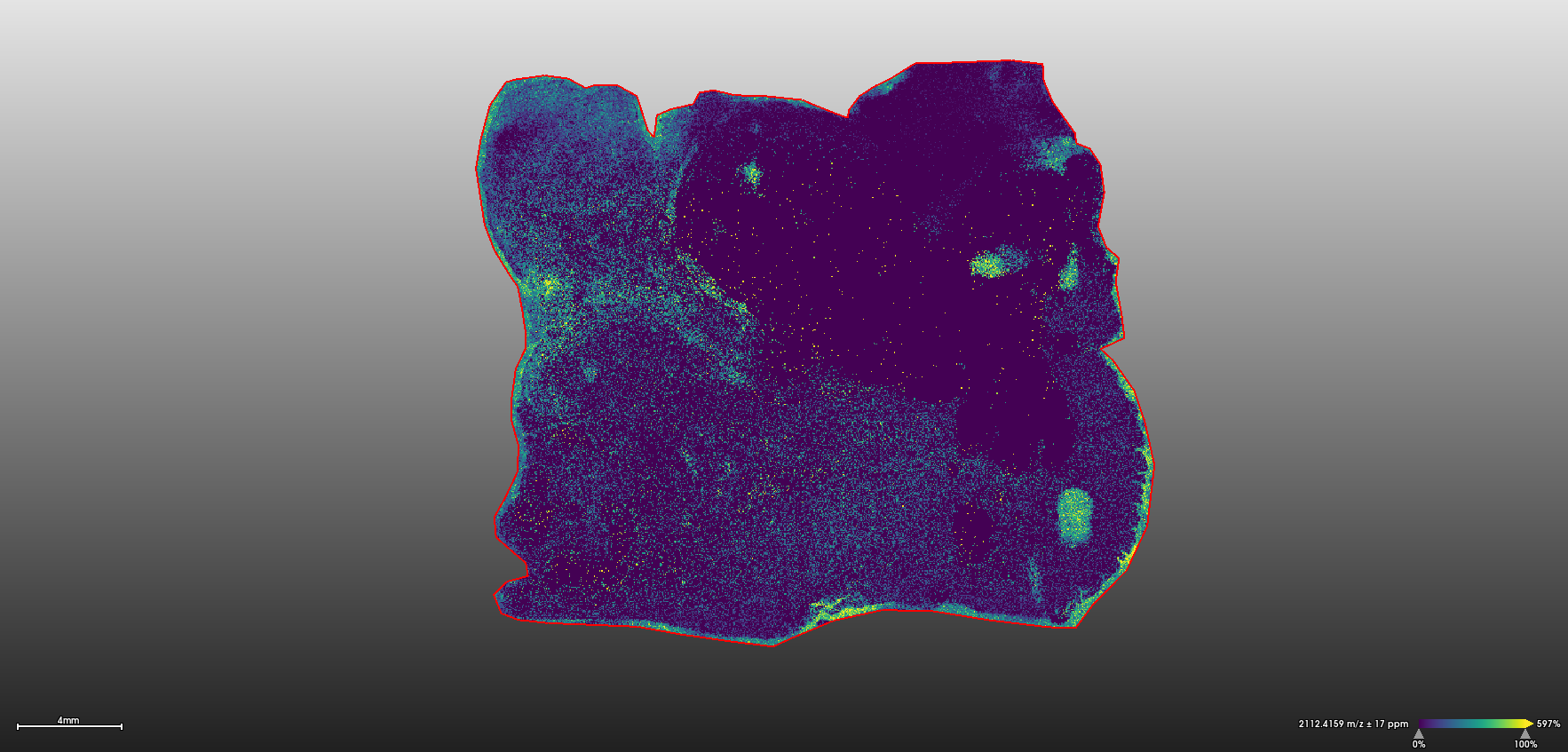

Supplement: Supplementary file 8 — Source Data 2 [file 41467_2026_72853_MOESM8_ESM.zip › Source Data MALDI Images/Supplementary Figure 15/20240524_mz2112_Colon1a.png]

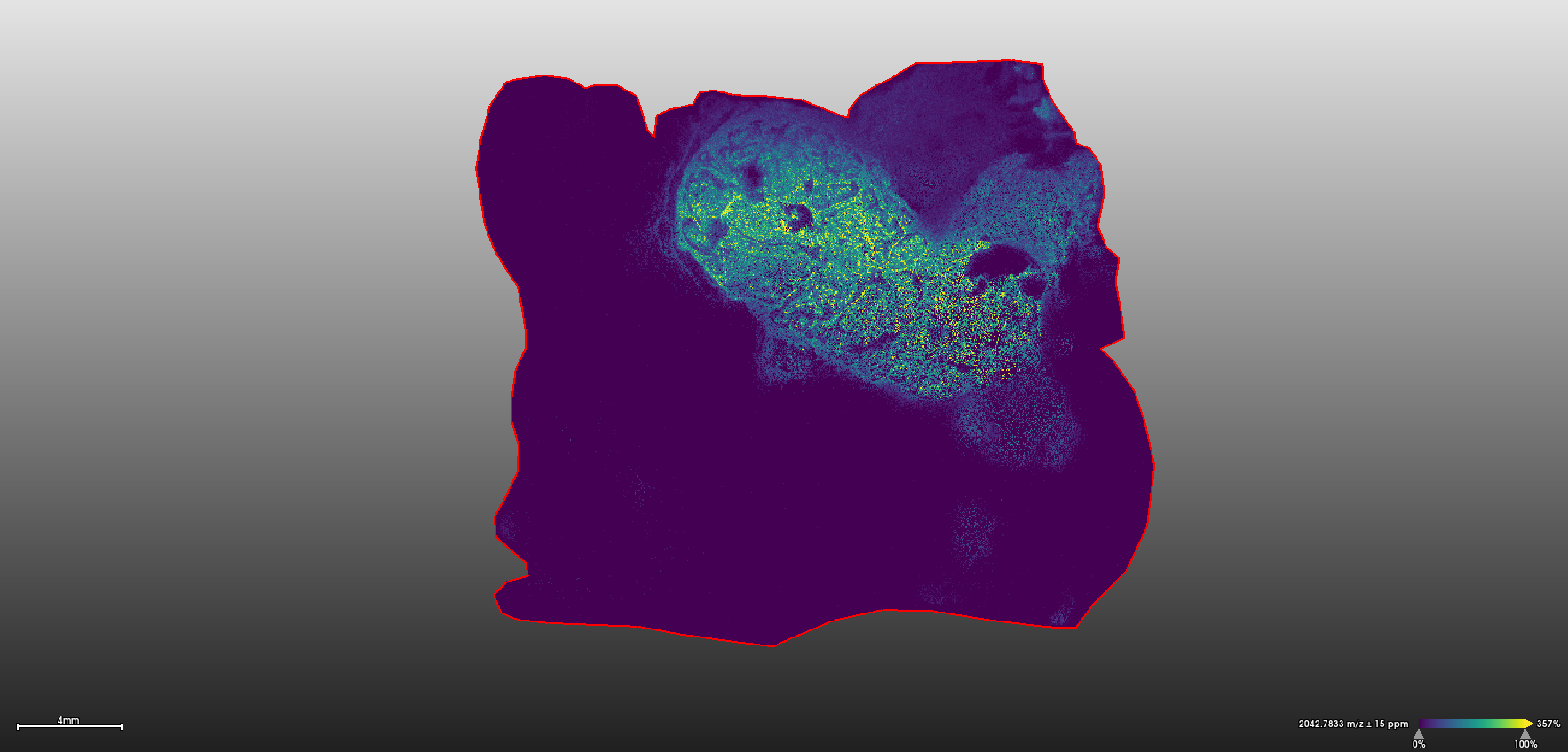

Supplement: Supplementary file 8 — Source Data 2 [file 41467_2026_72853_MOESM8_ESM.zip › Source Data MALDI Images/Supplementary Figure 15/20240524_mz2042_Colon1a.png]

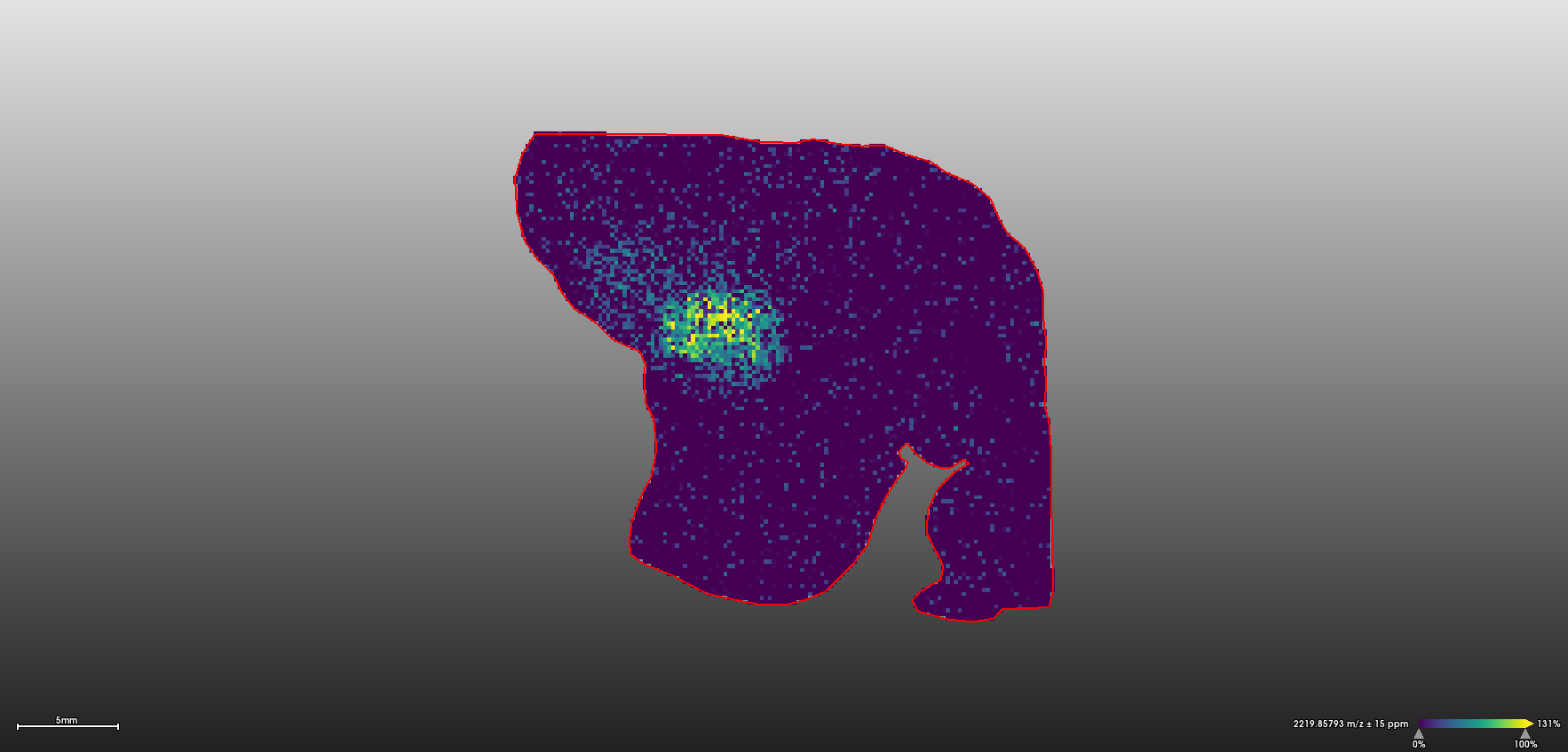

Supplement: Supplementary file 8 — Source Data 2 [file 41467_2026_72853_MOESM8_ESM.zip › Source Data MALDI Images/Supplementary Figure 15/20240627_mz2219_Colon1b.png]

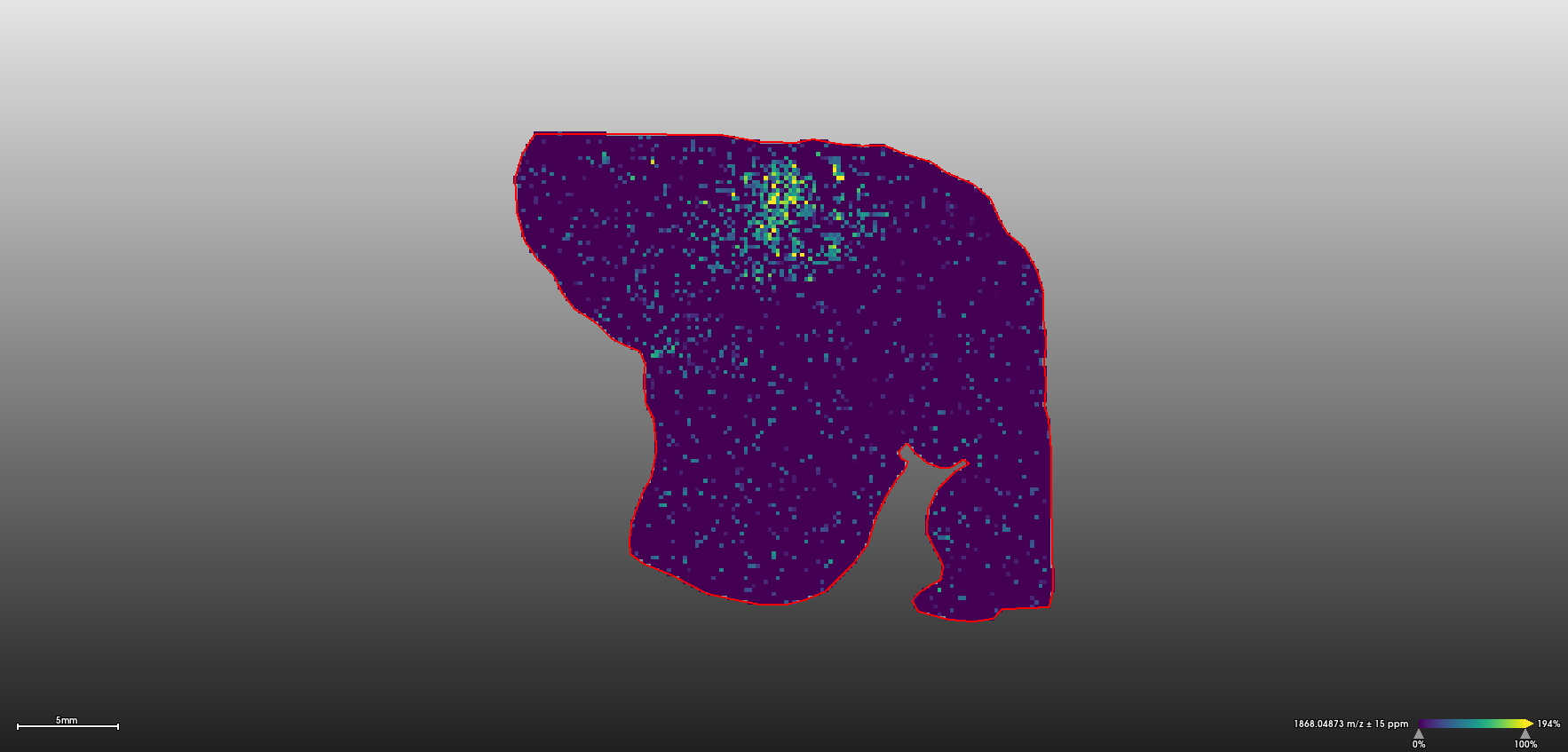

Supplement: Supplementary file 8 — Source Data 2 [file 41467_2026_72853_MOESM8_ESM.zip › Source Data MALDI Images/Supplementary Figure 15/20240531_mz1868_Colon1b.png]

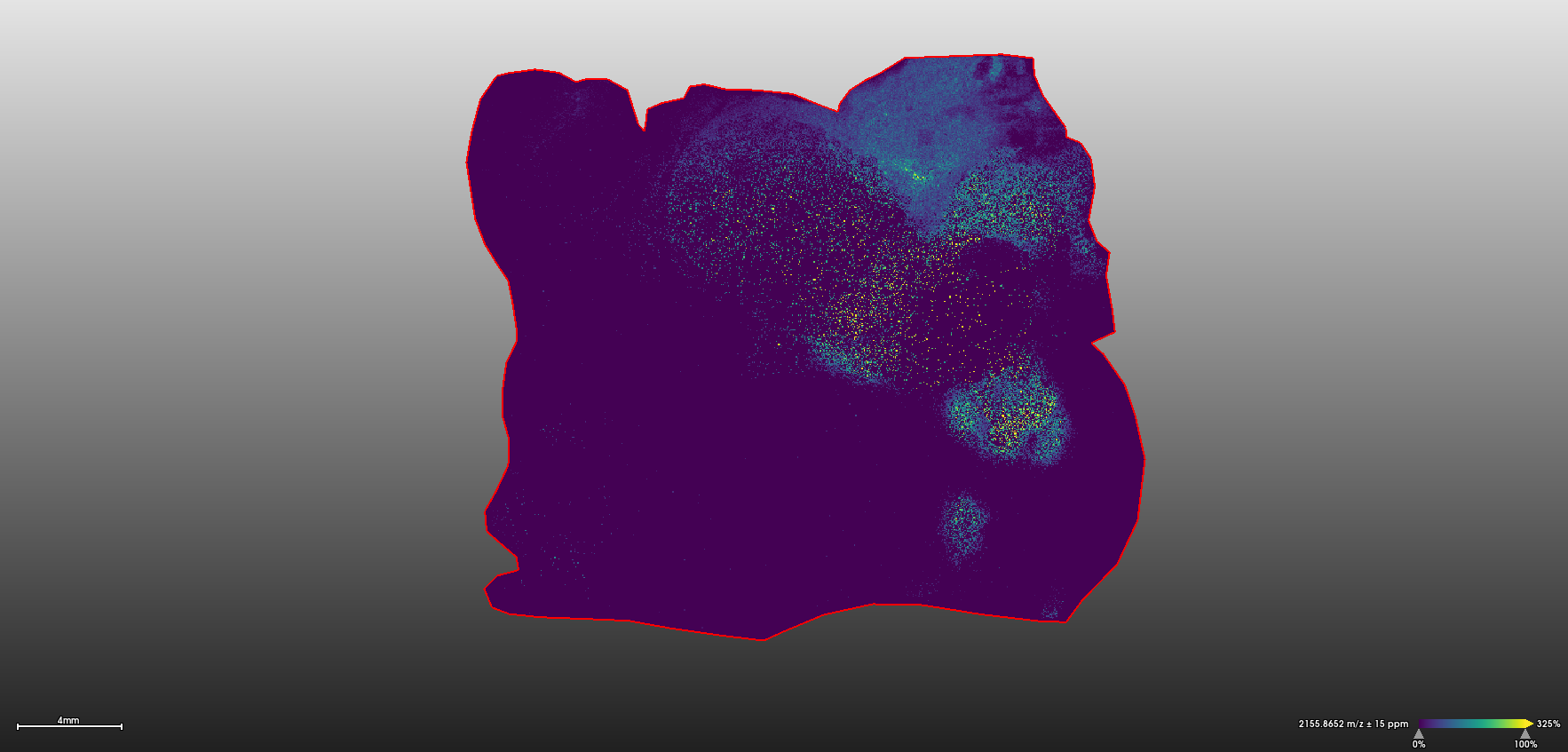

Supplement: Supplementary file 8 — Source Data 2 [file 41467_2026_72853_MOESM8_ESM.zip › Source Data MALDI Images/Supplementary Figure 15/20240524_mz2155_Colon1a.png]

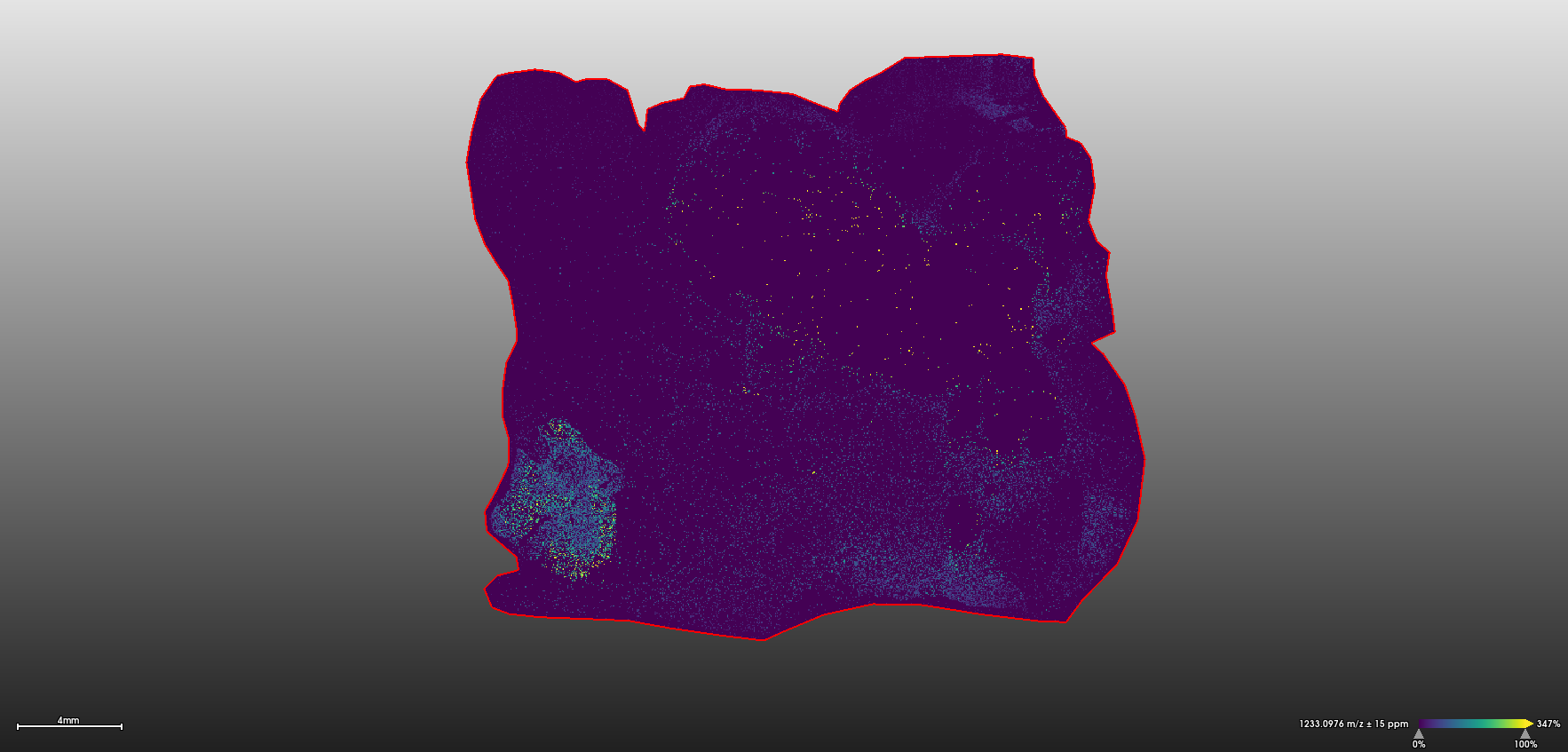

Supplement: Supplementary file 8 — Source Data 2 [file 41467_2026_72853_MOESM8_ESM.zip › Source Data MALDI Images/Supplementary Figure 15/20240524_mz1233_Colon1a.png]

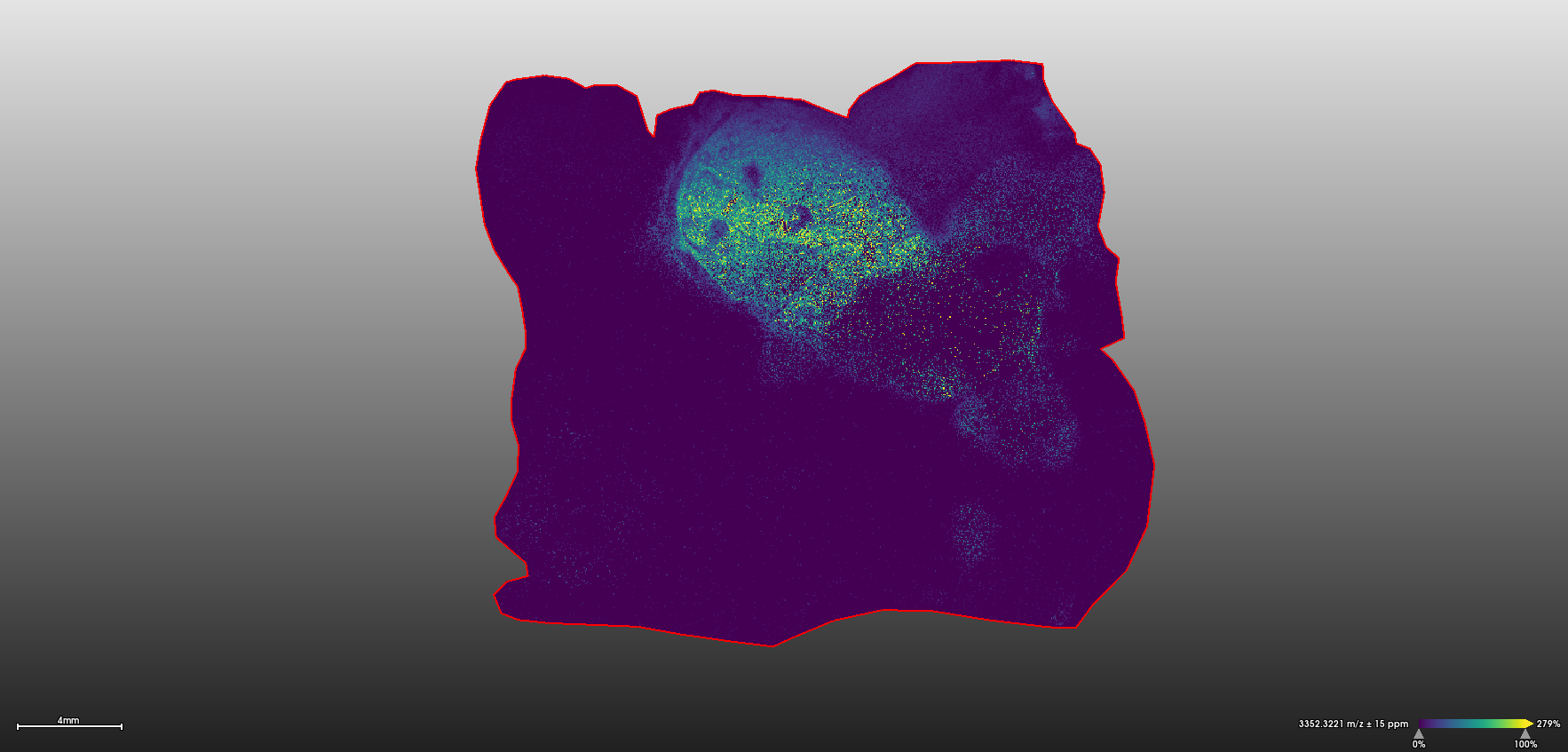

Supplement: Supplementary file 8 — Source Data 2 [file 41467_2026_72853_MOESM8_ESM.zip › Source Data MALDI Images/Supplementary Figure 15/20240524_mz3352_Colon1a.png]

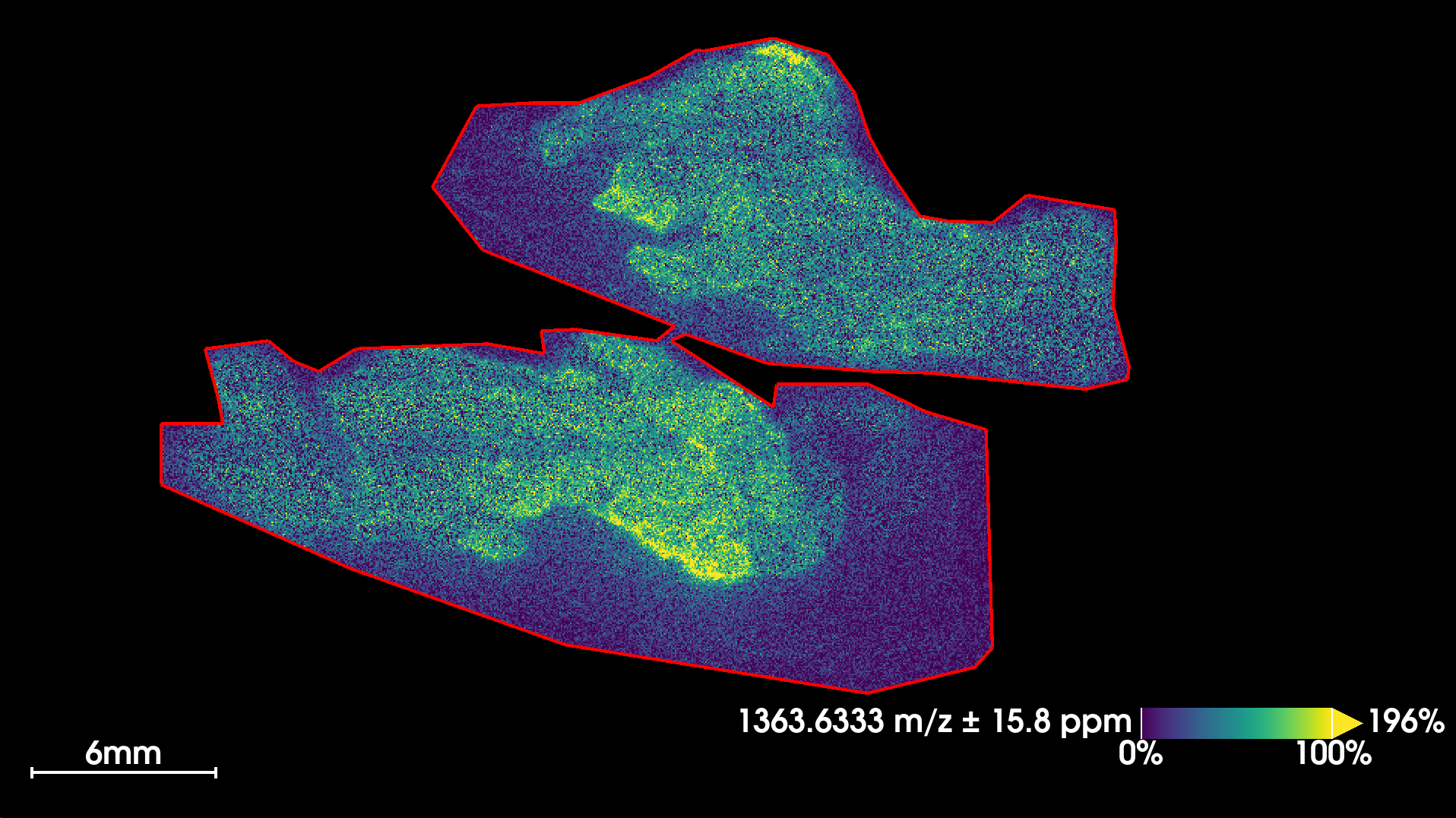

Supplement: Supplementary file 8 — Source Data 2 [file 41467_2026_72853_MOESM8_ESM.zip › Source Data MALDI Images/Supplementary Figure 14/1363.6333 mz ┬▒ 21.6 mDa.png]

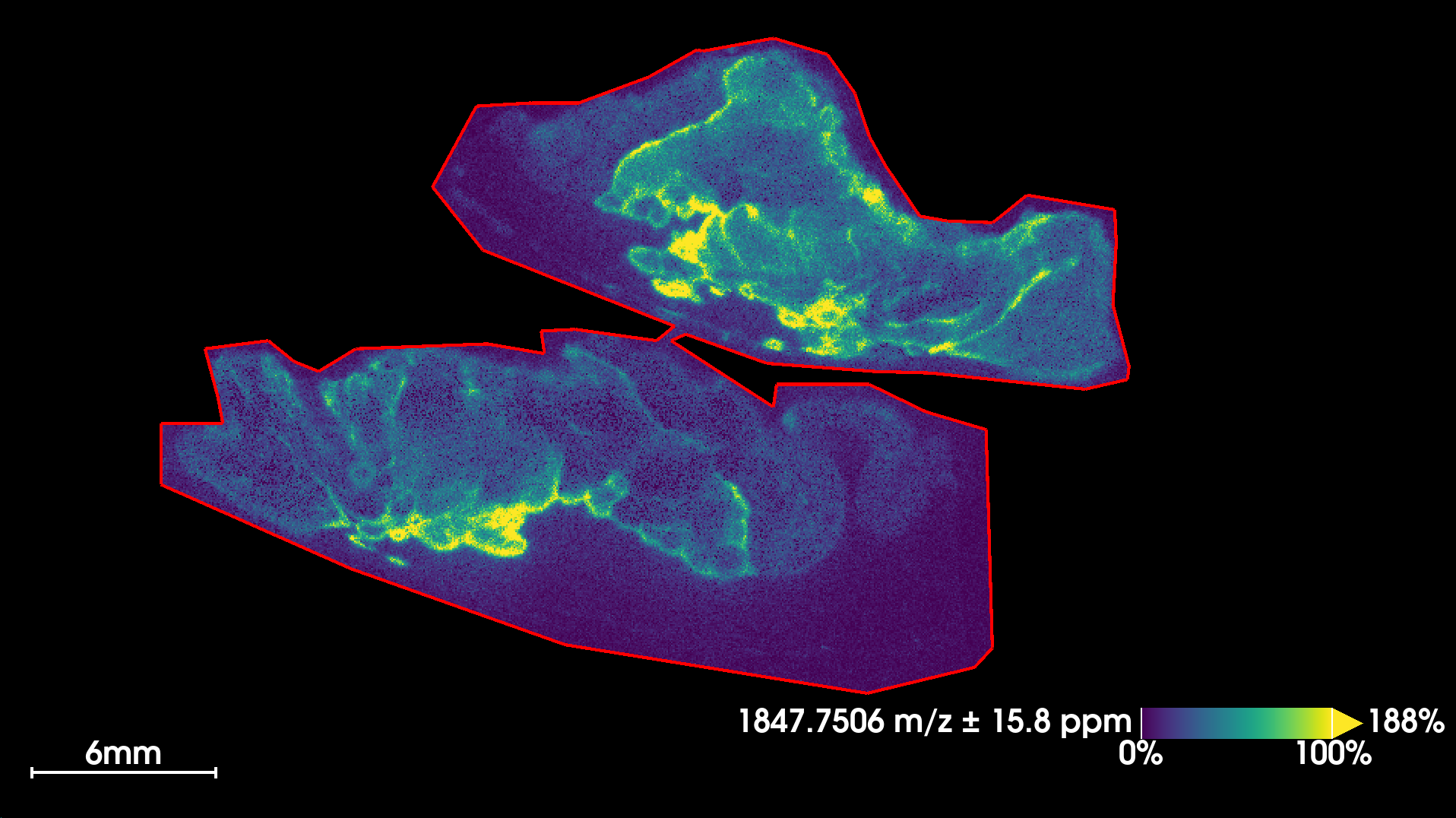

Supplement: Supplementary file 8 — Source Data 2 [file 41467_2026_72853_MOESM8_ESM.zip › Source Data MALDI Images/Supplementary Figure 14/1847.7506 mz ┬▒ 29.3 mDa.png]

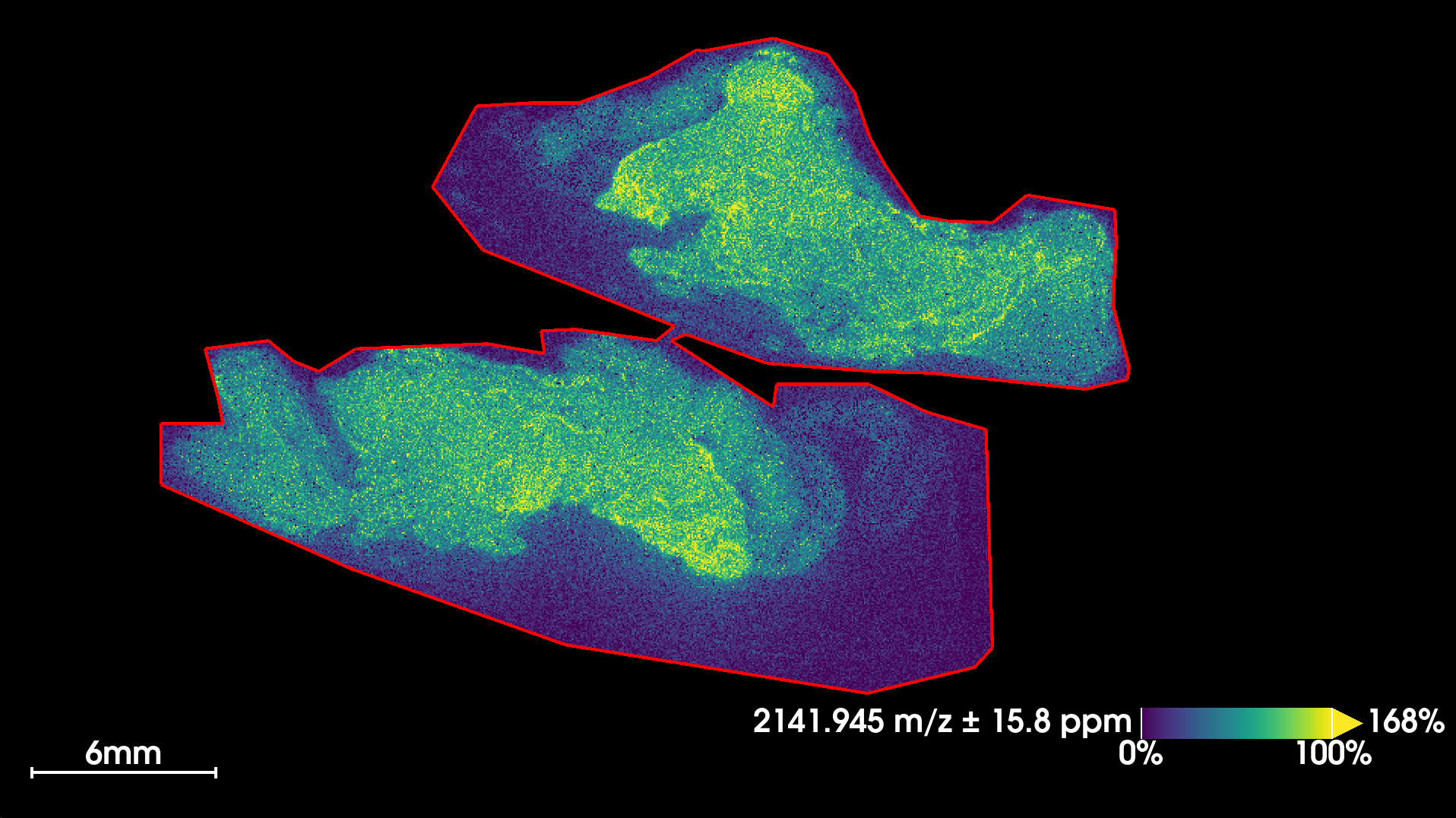

Supplement: Supplementary file 8 — Source Data 2 [file 41467_2026_72853_MOESM8_ESM.zip › Source Data MALDI Images/Supplementary Figure 14/2141.945 mz ┬▒ 33.9 mDa.png]

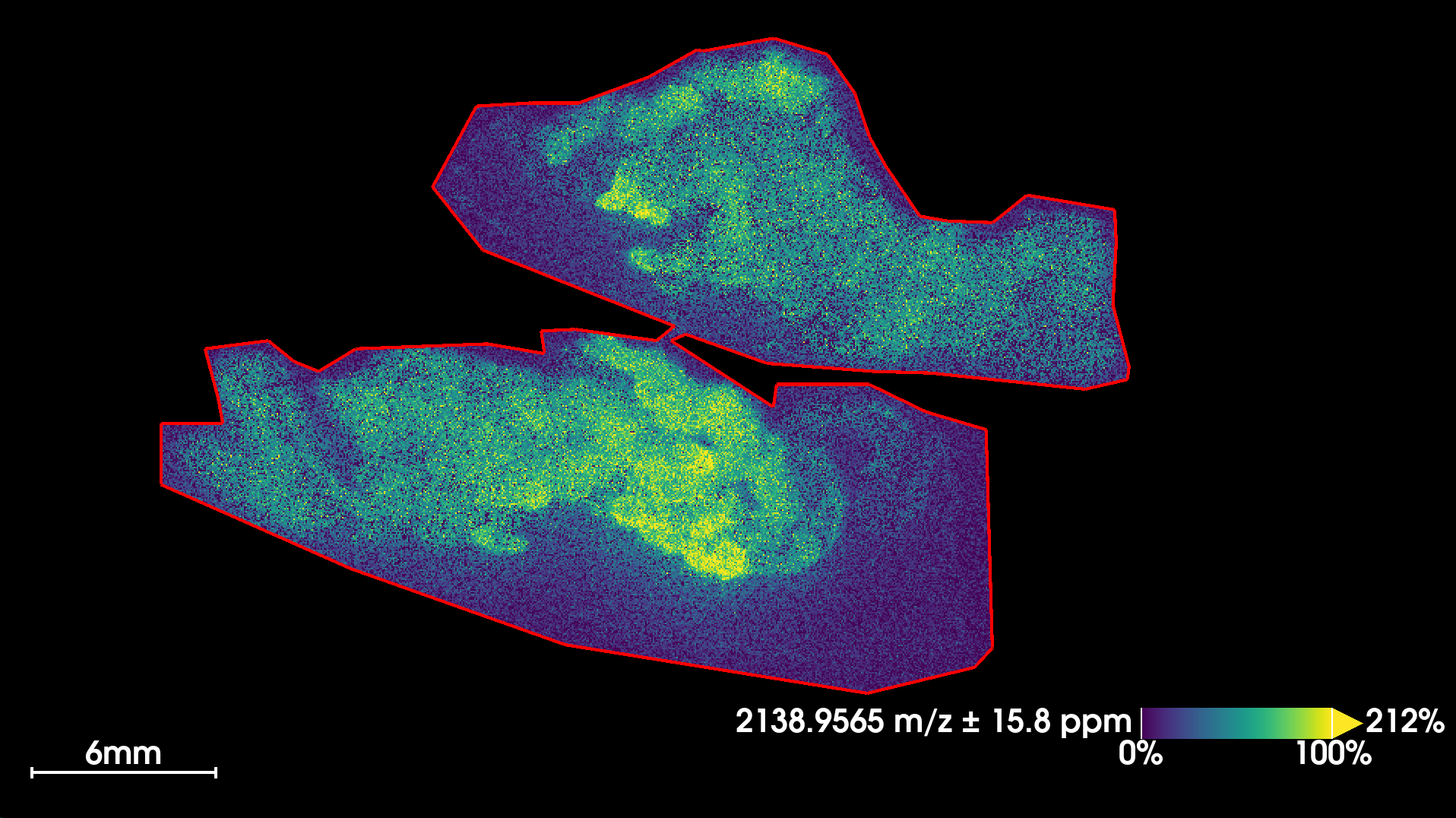

Supplement: Supplementary file 8 — Source Data 2 [file 41467_2026_72853_MOESM8_ESM.zip › Source Data MALDI Images/Supplementary Figure 14/2138.9565 mz ┬▒ 33.9 mDa.png]

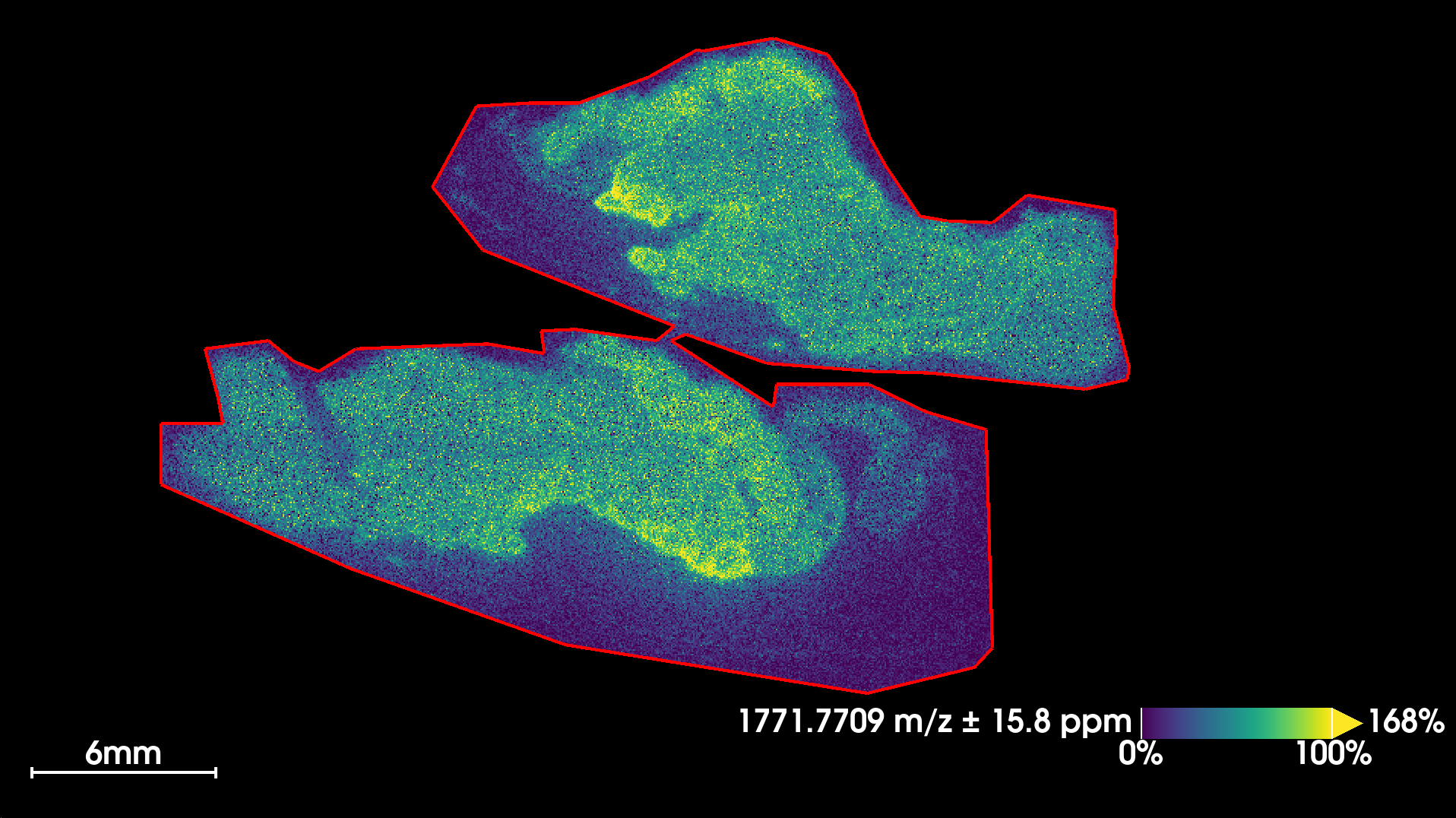

Supplement: Supplementary file 8 — Source Data 2 [file 41467_2026_72853_MOESM8_ESM.zip › Source Data MALDI Images/Supplementary Figure 14/1771.7709 mz ┬▒ 28.1 mDa.png]

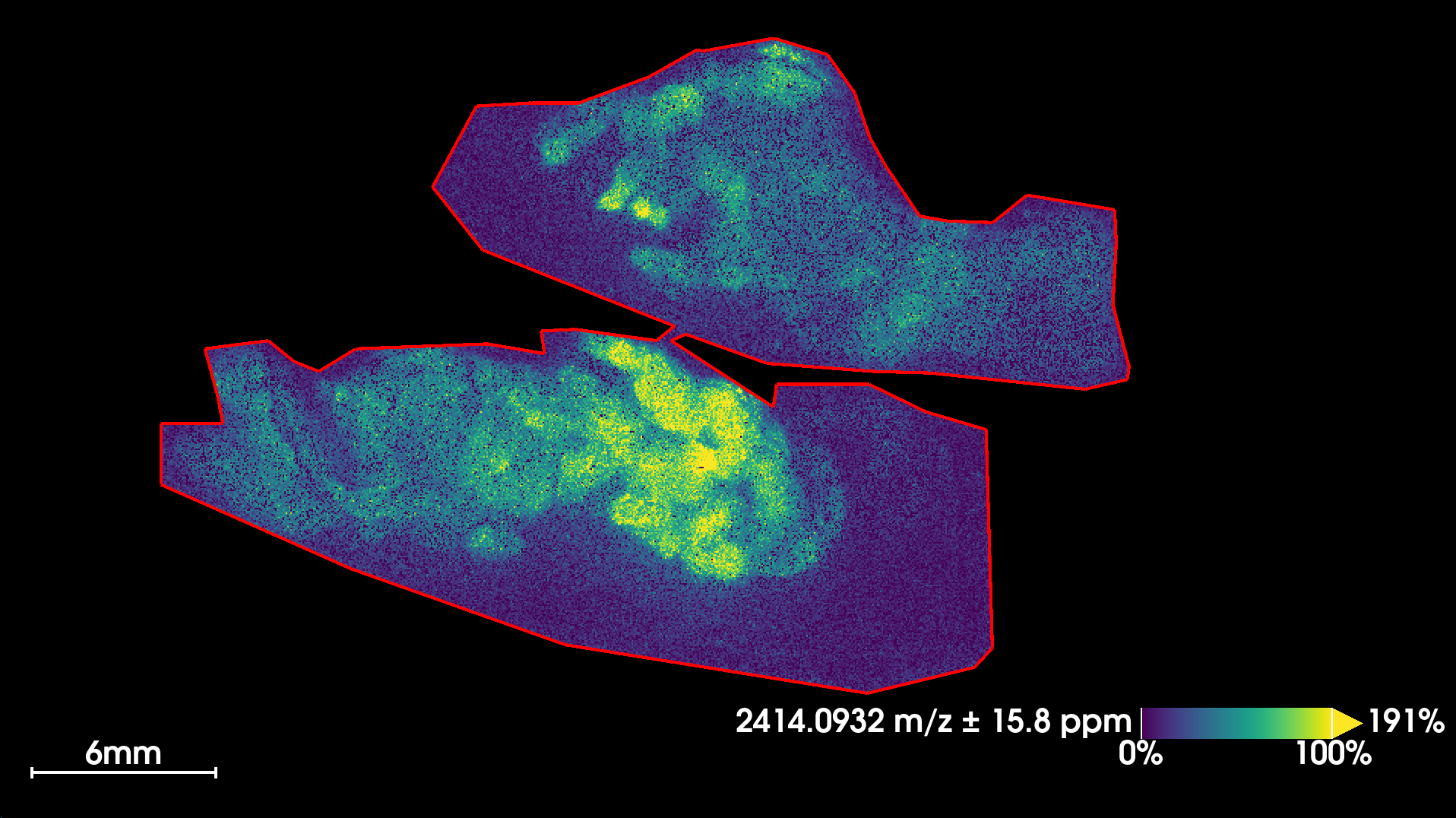

Supplement: Supplementary file 8 — Source Data 2 [file 41467_2026_72853_MOESM8_ESM.zip › Source Data MALDI Images/Supplementary Figure 14/2414.0932 mz ┬▒ 38.2 mDa.png]

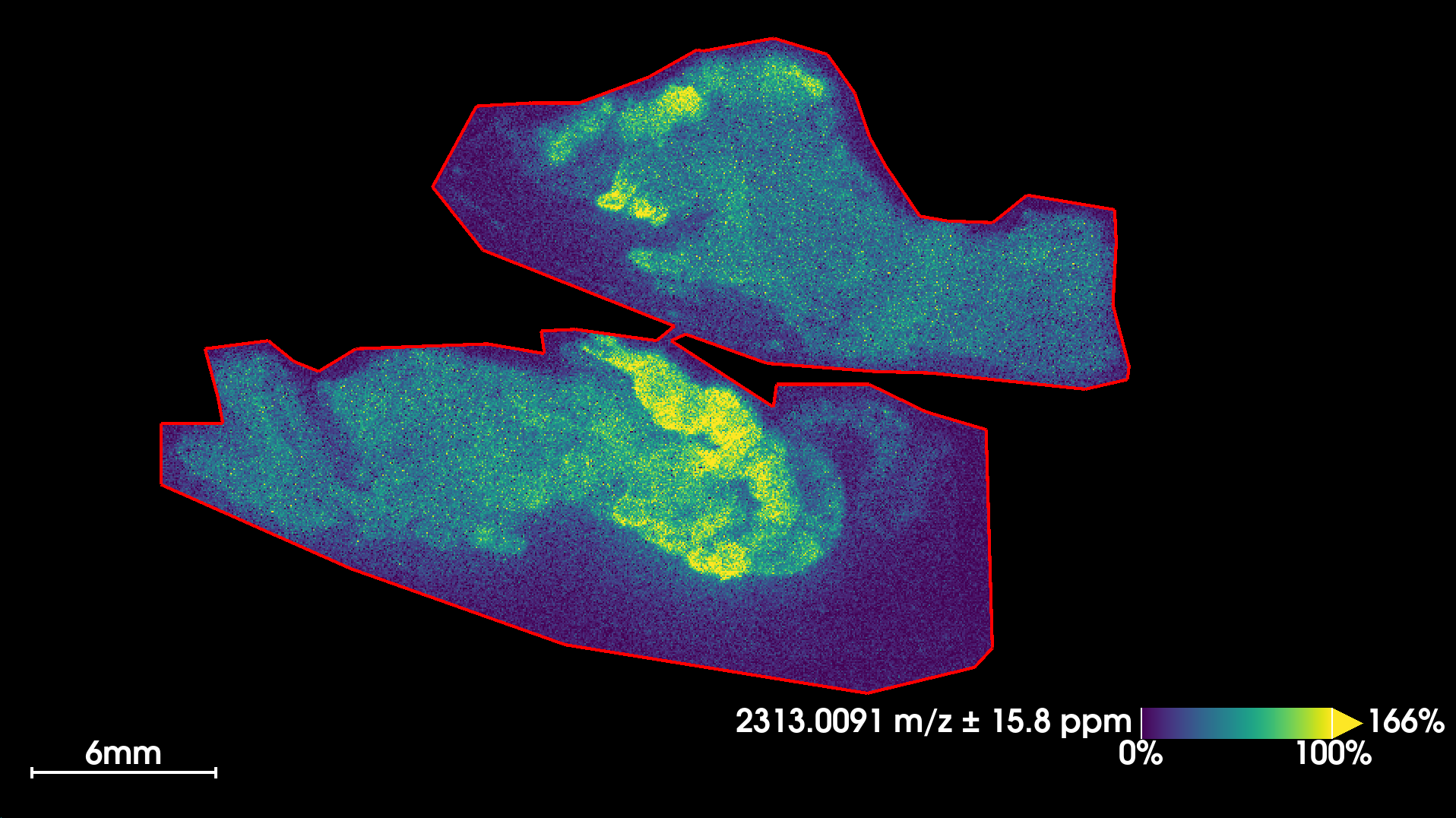

Supplement: Supplementary file 8 — Source Data 2 [file 41467_2026_72853_MOESM8_ESM.zip › Source Data MALDI Images/Supplementary Figure 14/2313.0091 mz ┬▒ 36.6 mDa.png]

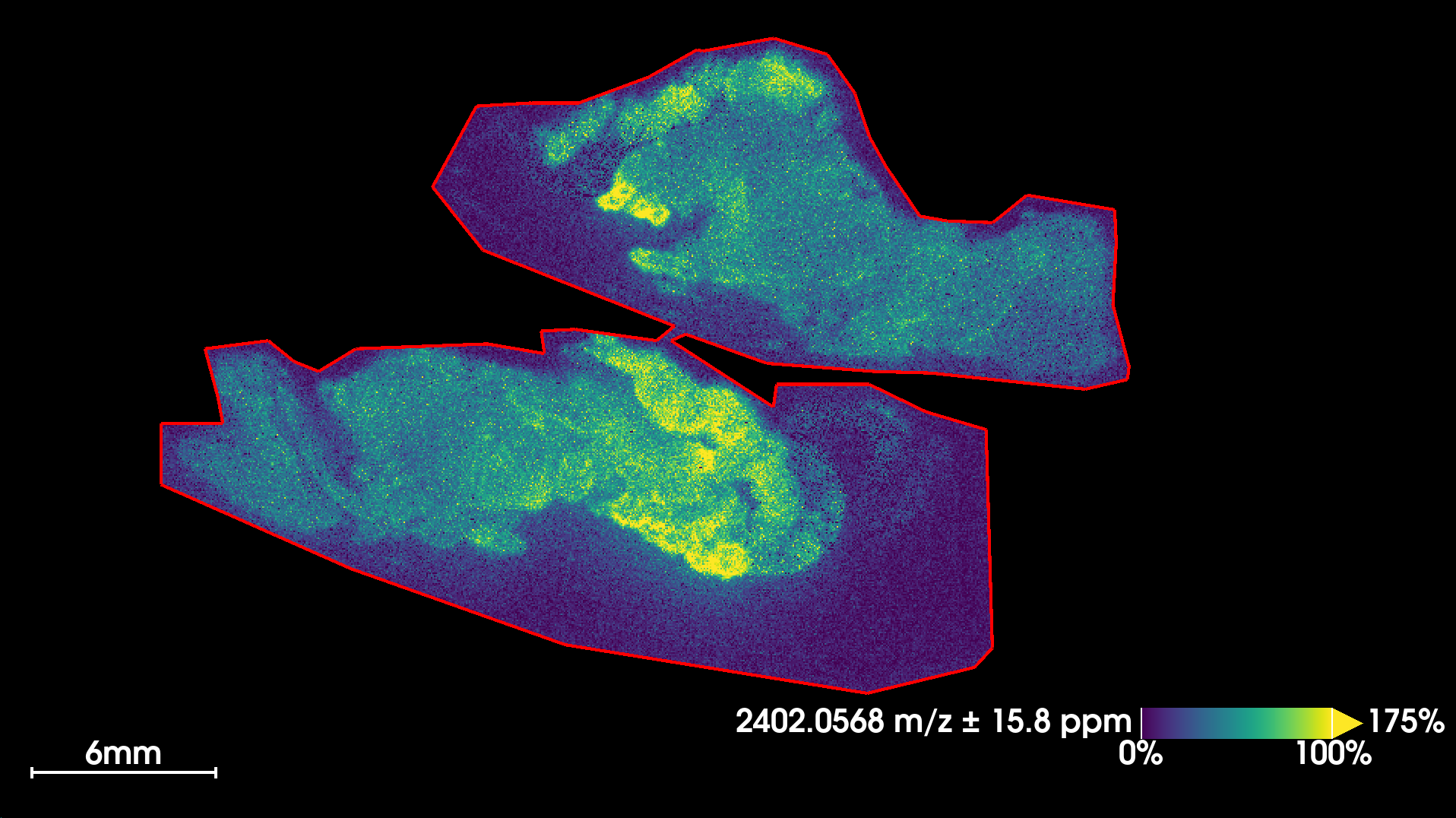

Supplement: Supplementary file 8 — Source Data 2 [file 41467_2026_72853_MOESM8_ESM.zip › Source Data MALDI Images/Supplementary Figure 14/2402.0568 mz ┬▒ 38.1 mDa.png]

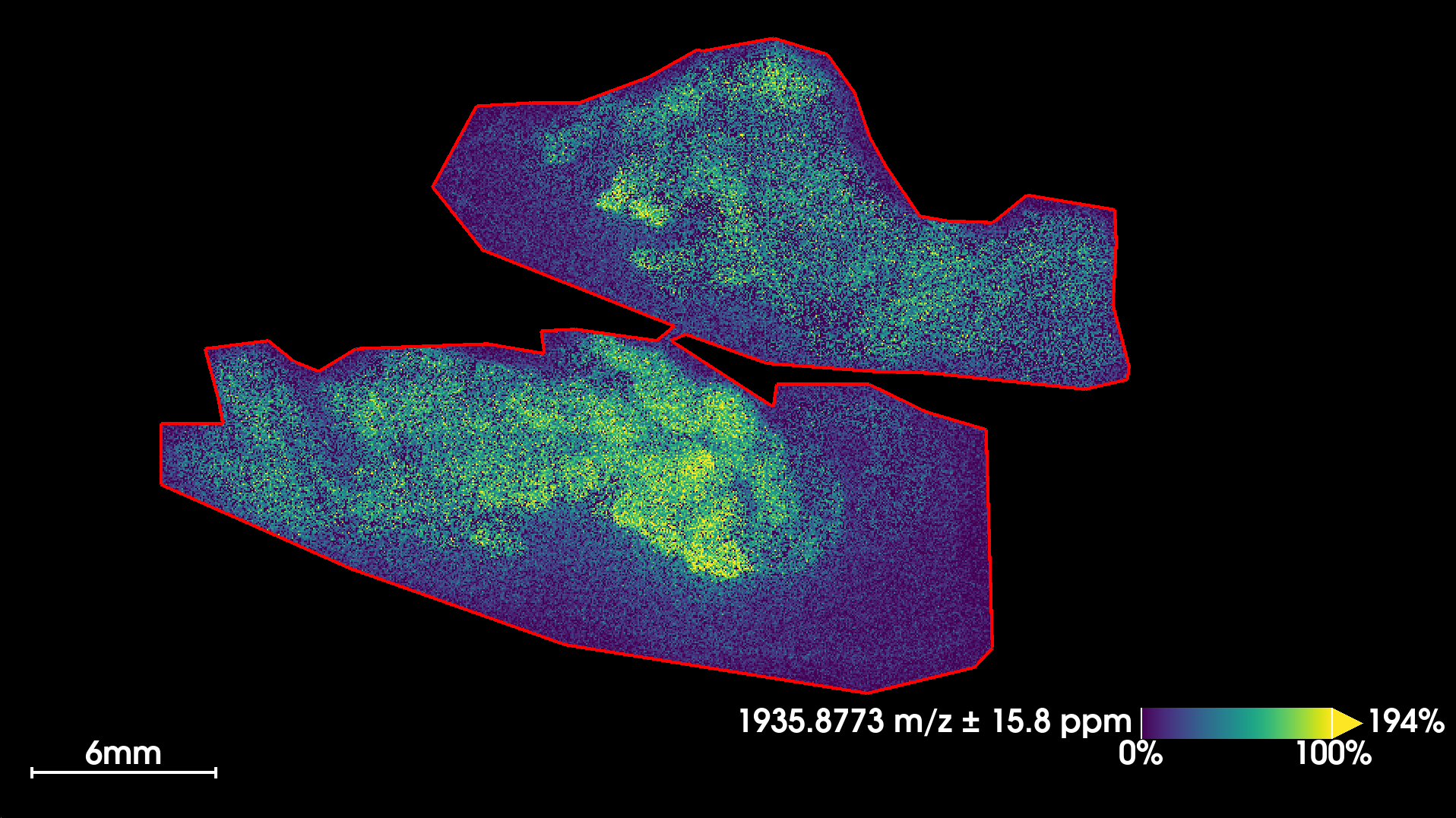

Supplement: Supplementary file 8 — Source Data 2 [file 41467_2026_72853_MOESM8_ESM.zip › Source Data MALDI Images/Supplementary Figure 14/1935.8773 mz ┬▒ 30.7 mDa.png]

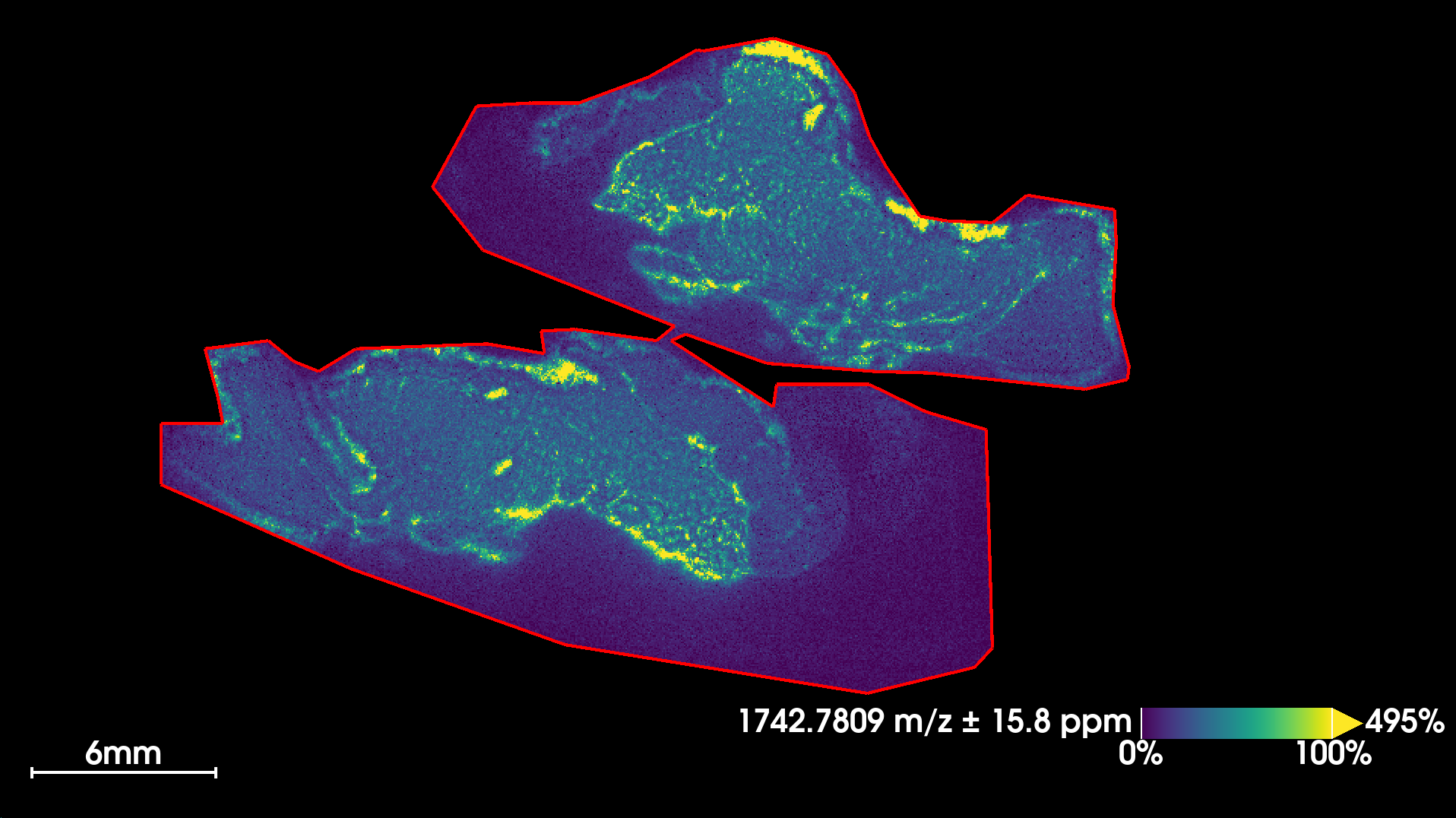

Supplement: Supplementary file 8 — Source Data 2 [file 41467_2026_72853_MOESM8_ESM.zip › Source Data MALDI Images/Supplementary Figure 14/1742.7809 mz ┬▒ 27.6 mDa.png]

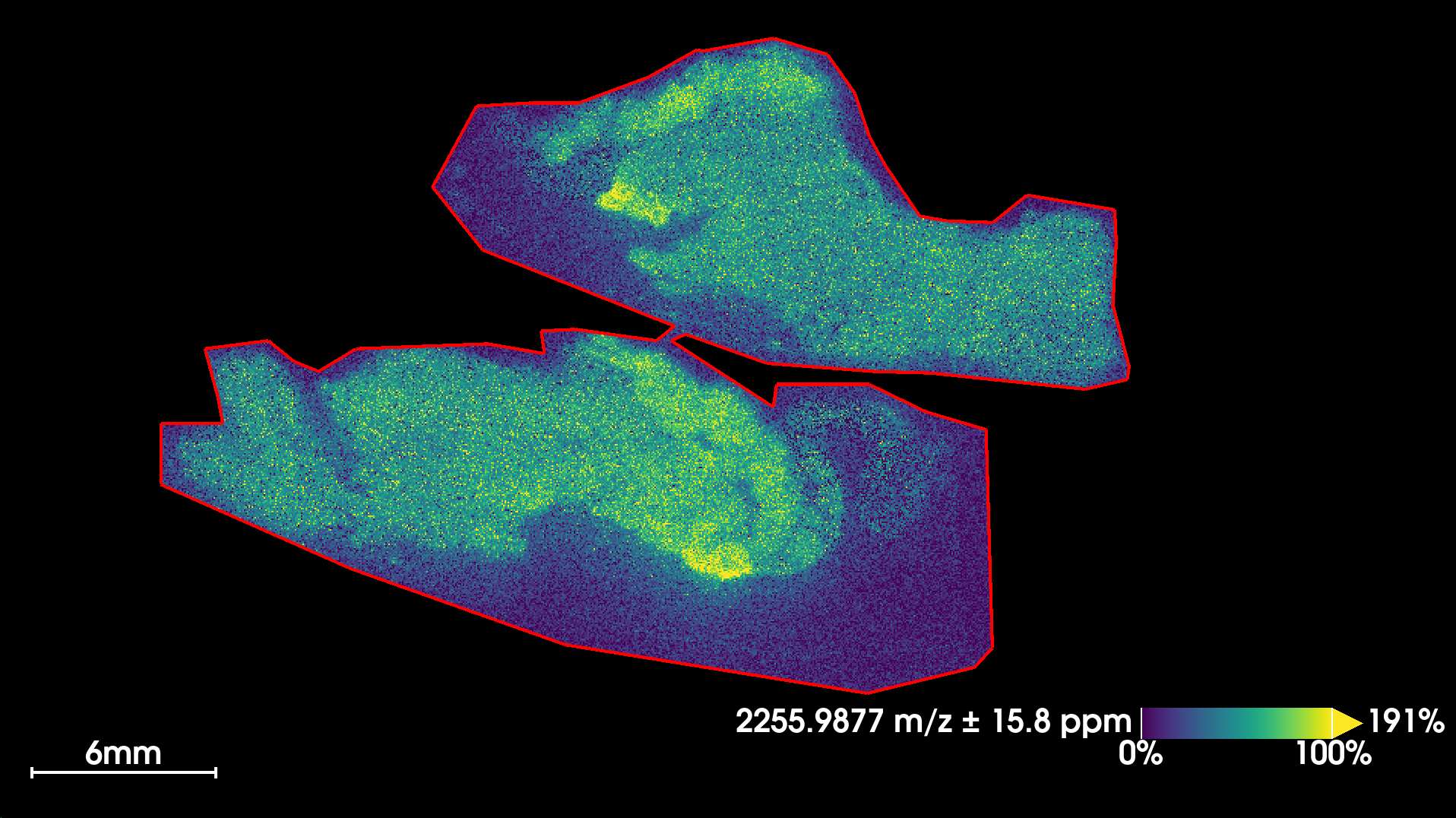

Supplement: Supplementary file 8 — Source Data 2 [file 41467_2026_72853_MOESM8_ESM.zip › Source Data MALDI Images/Supplementary Figure 14/2255.9877 mz ┬▒ 35.7 mDa.png]

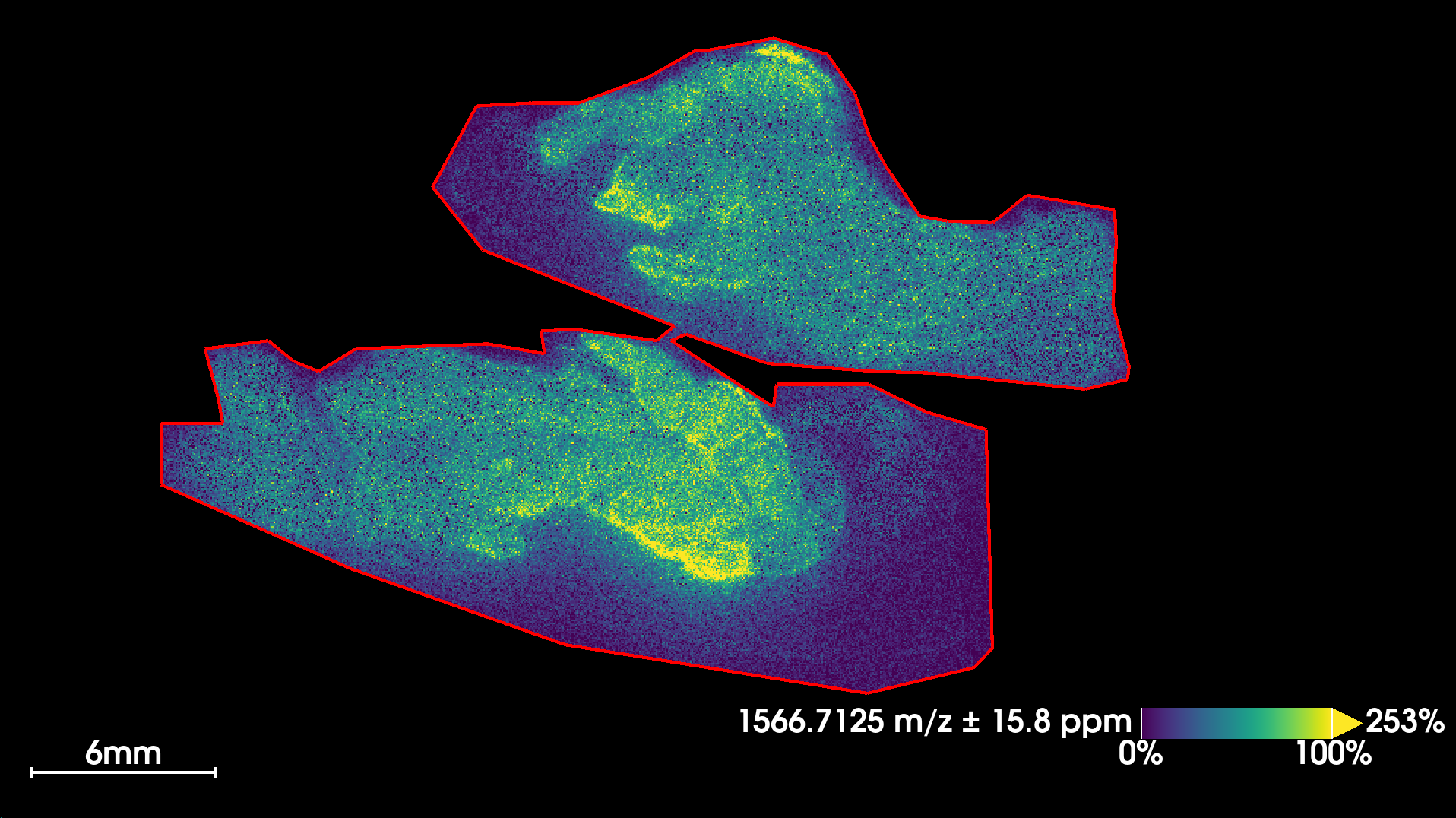

Supplement: Supplementary file 8 — Source Data 2 [file 41467_2026_72853_MOESM8_ESM.zip › Source Data MALDI Images/Supplementary Figure 14/1566.7125 mz ┬▒ 24.8 mDa.png]

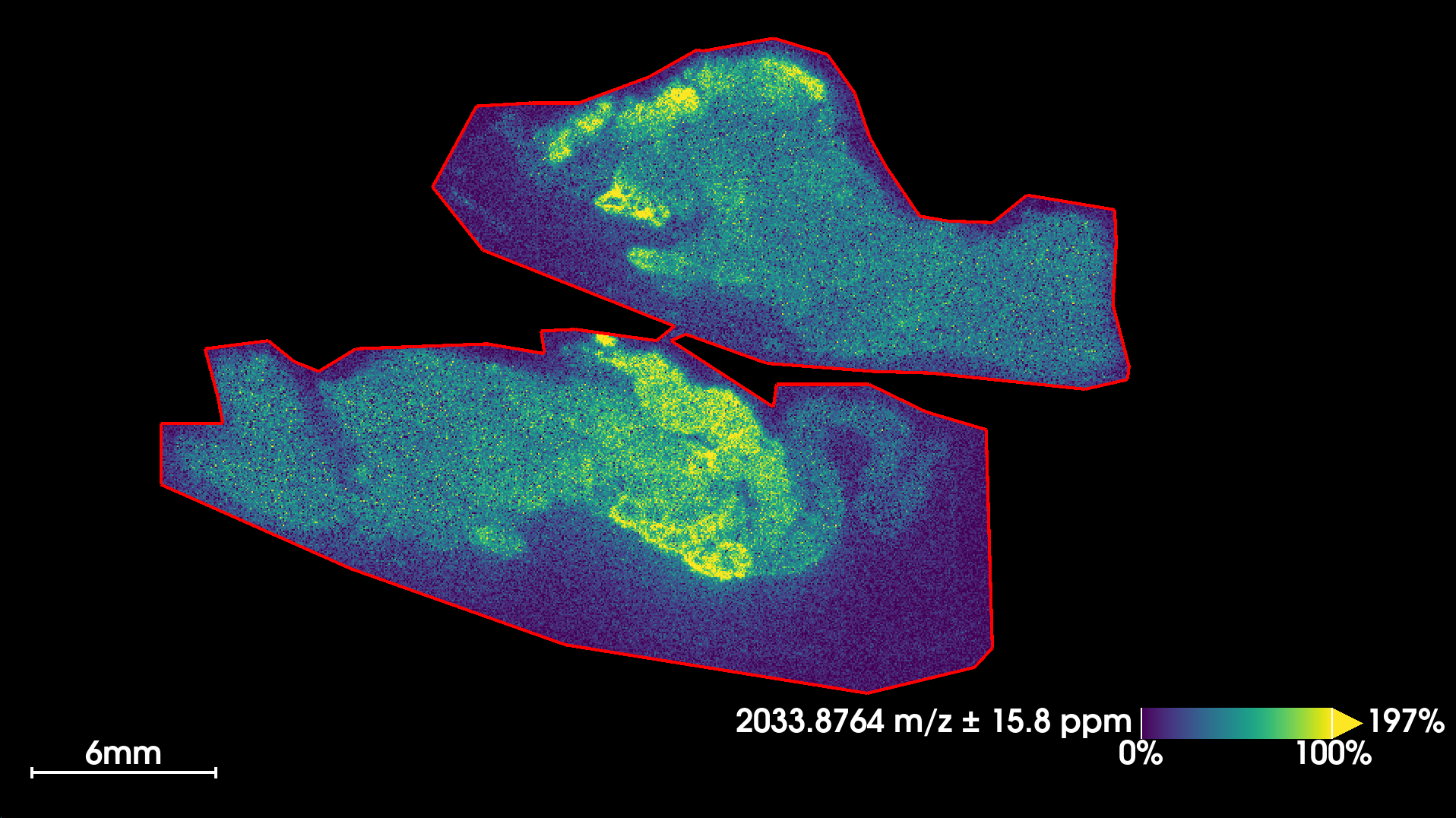

Supplement: Supplementary file 8 — Source Data 2 [file 41467_2026_72853_MOESM8_ESM.zip › Source Data MALDI Images/Supplementary Figure 14/2033.8764 mz ┬▒ 32.2 mDa.png]

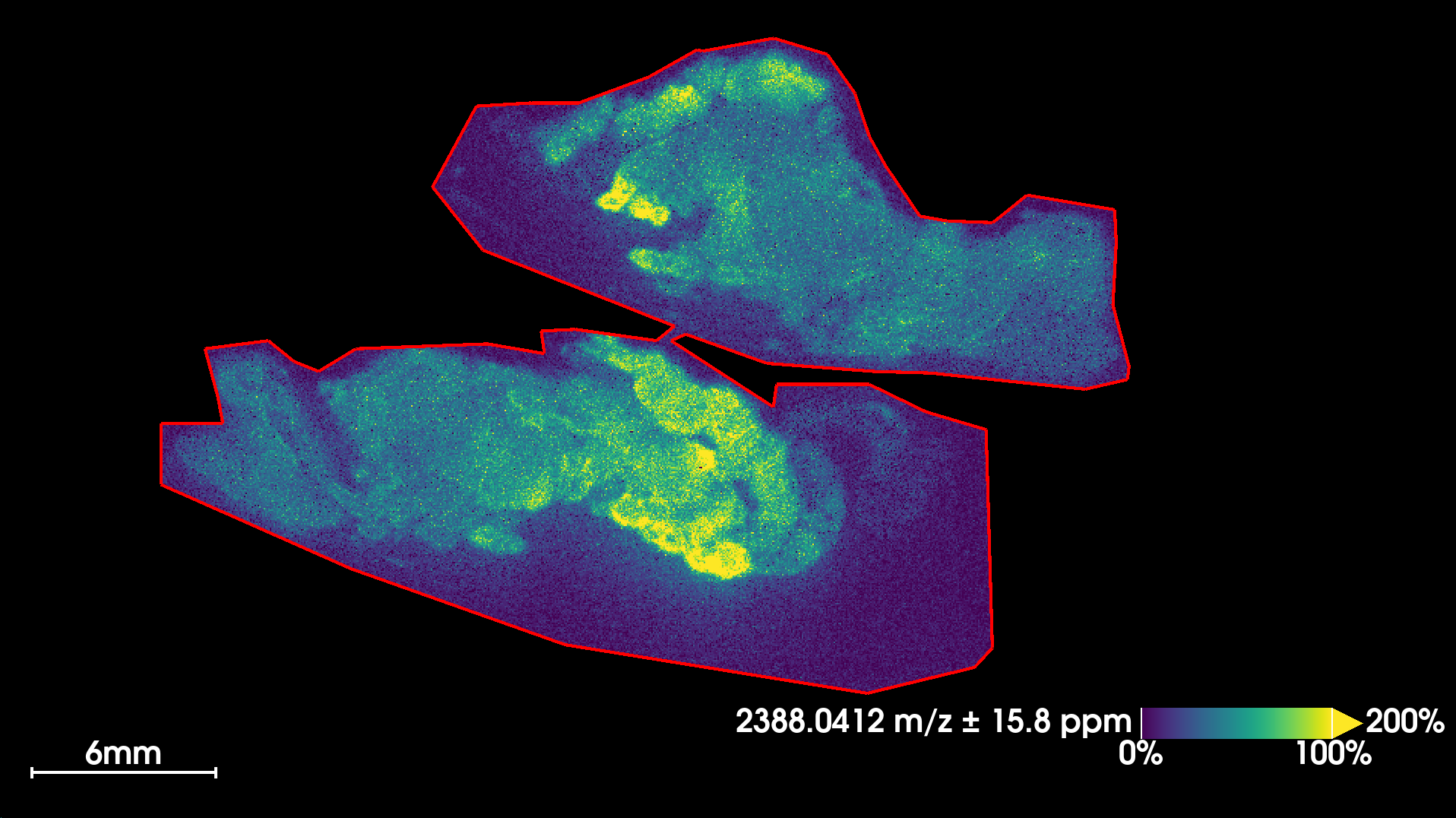

Supplement: Supplementary file 8 — Source Data 2 [file 41467_2026_72853_MOESM8_ESM.zip › Source Data MALDI Images/Supplementary Figure 14/2388.0412 mz ┬▒ 37.8 mDa.png]

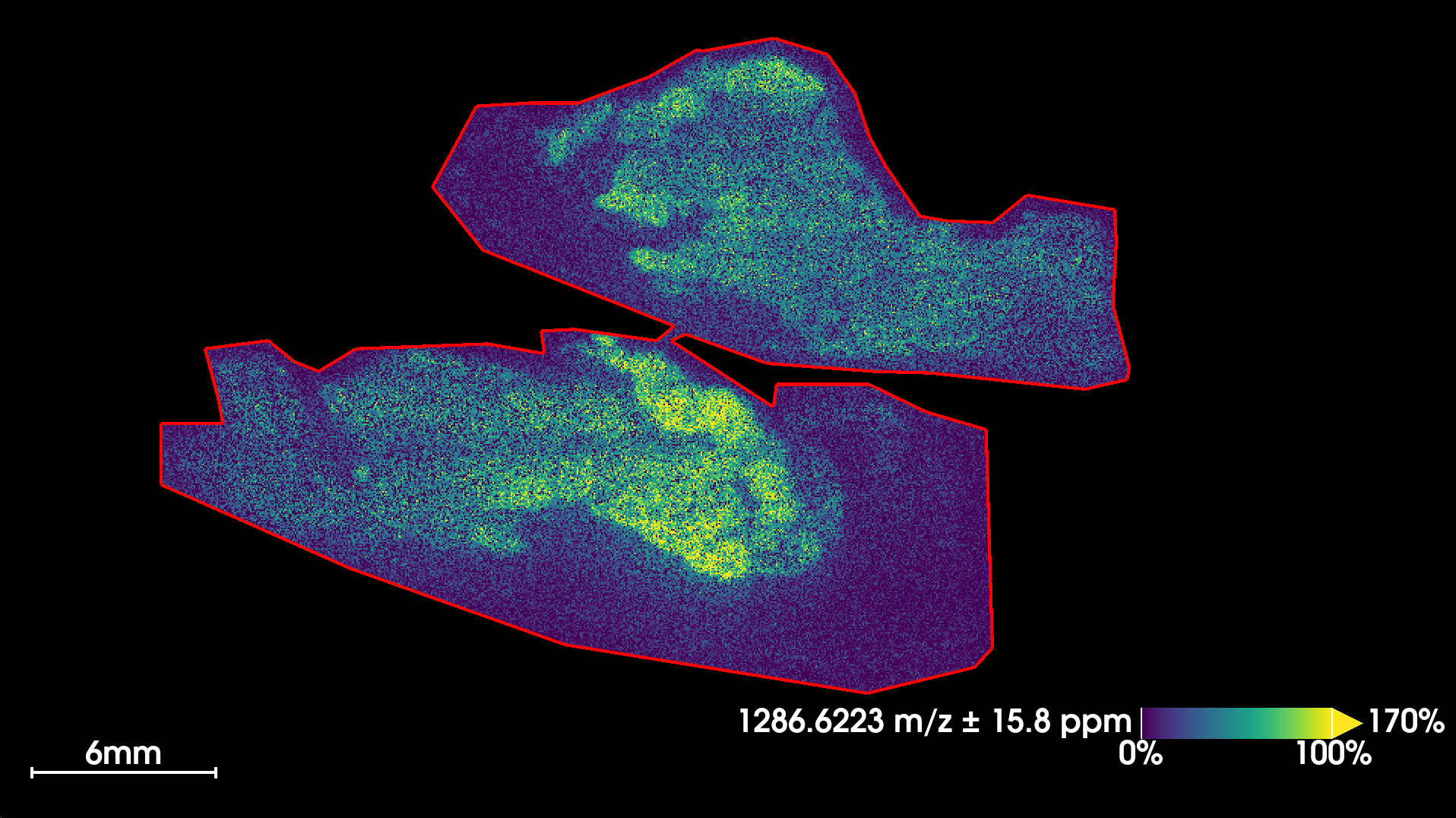

Supplement: Supplementary file 8 — Source Data 2 [file 41467_2026_72853_MOESM8_ESM.zip › Source Data MALDI Images/Supplementary Figure 14/1286.6223 mz ┬▒ 20.4 mDa.png]

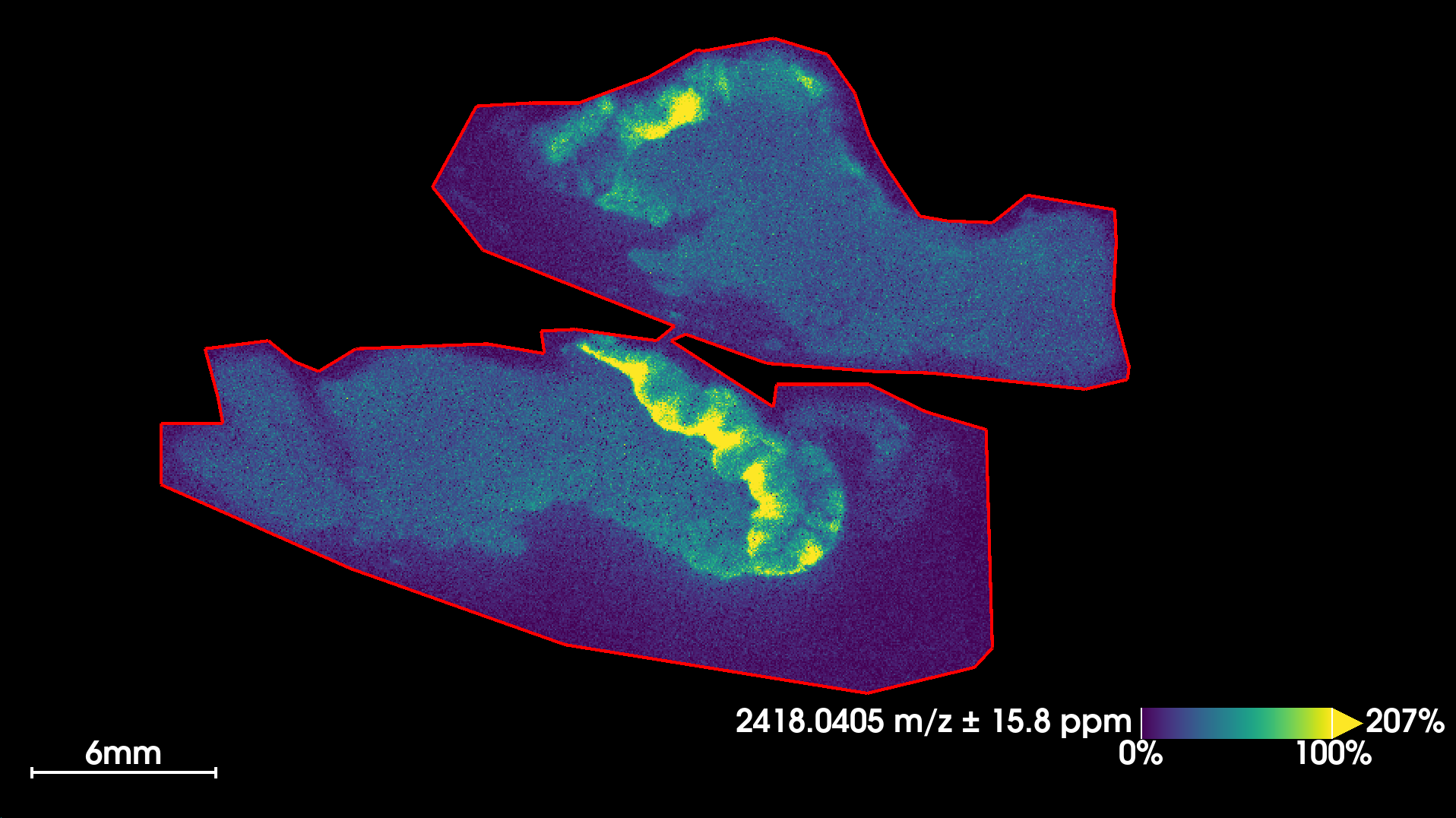

Supplement: Supplementary file 8 — Source Data 2 [file 41467_2026_72853_MOESM8_ESM.zip › Source Data MALDI Images/Supplementary Figure 14/2418.0405 mz ┬▒ 38.3 mDa.png]

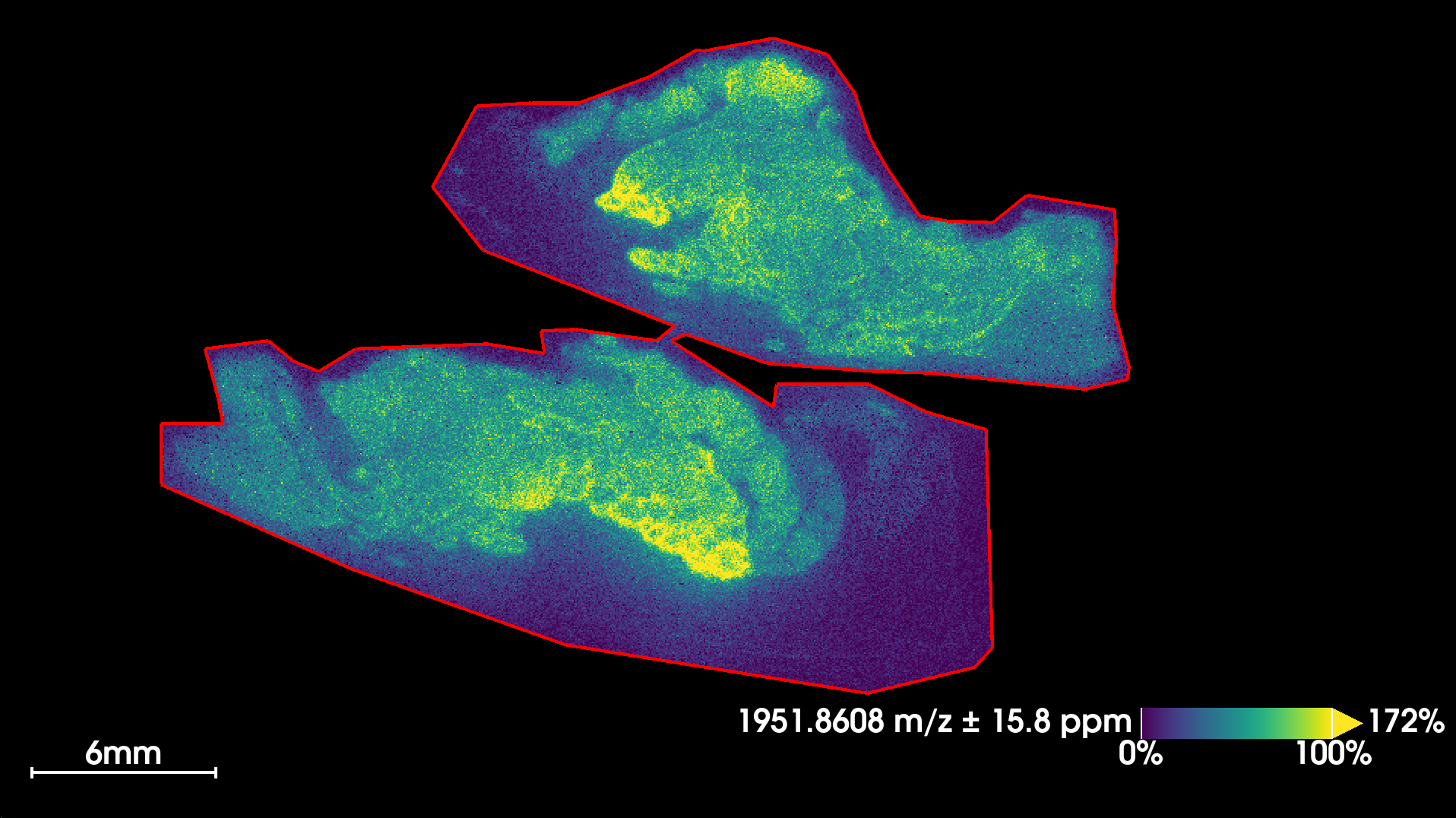

Supplement: Supplementary file 8 — Source Data 2 [file 41467_2026_72853_MOESM8_ESM.zip › Source Data MALDI Images/Supplementary Figure 14/1951.8608 mz ┬▒ 30.9 mDa.png]

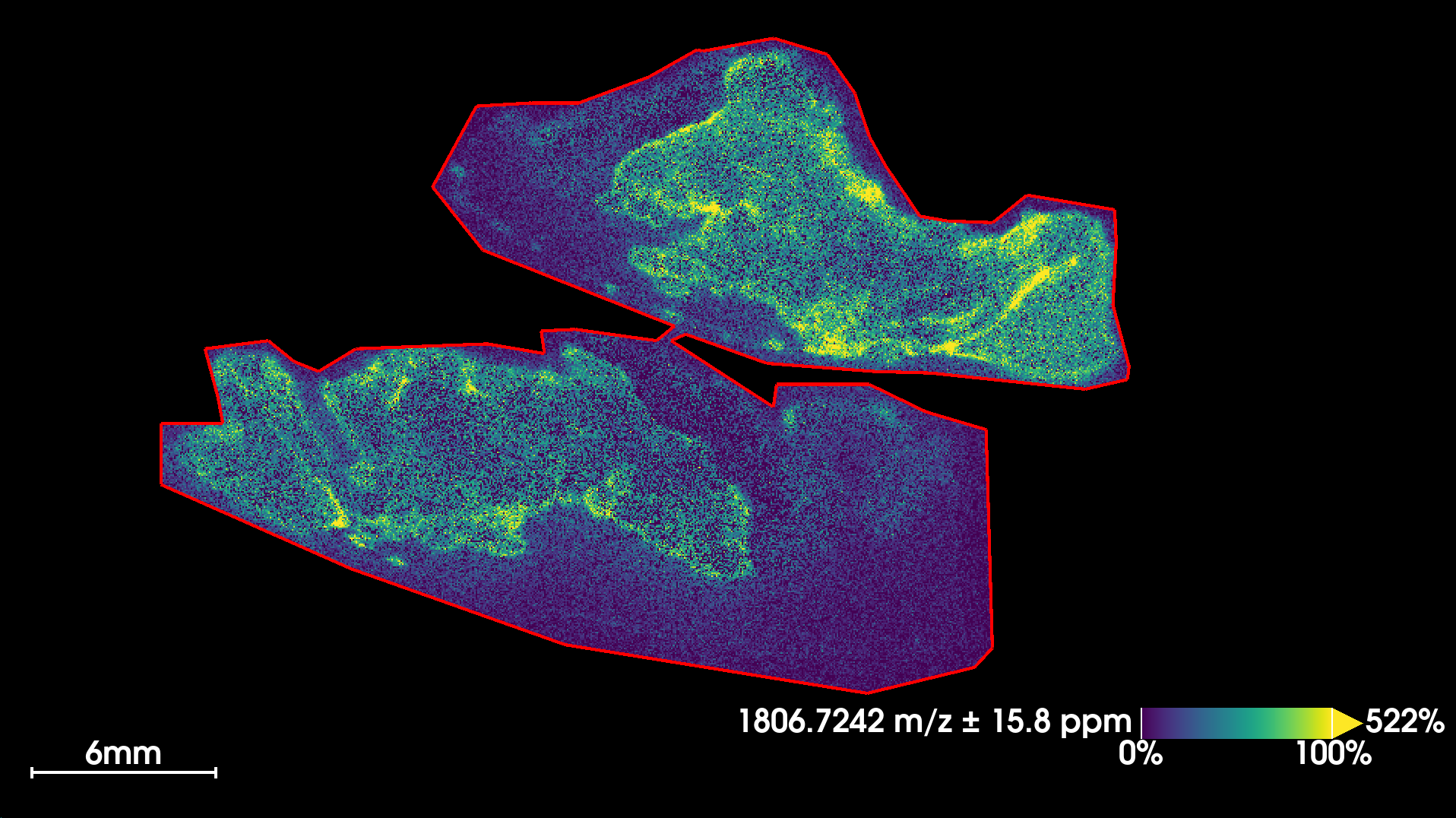

Supplement: Supplementary file 8 — Source Data 2 [file 41467_2026_72853_MOESM8_ESM.zip › Source Data MALDI Images/Supplementary Figure 14/1806.7242 mz ┬▒ 28.6 mDa.png]

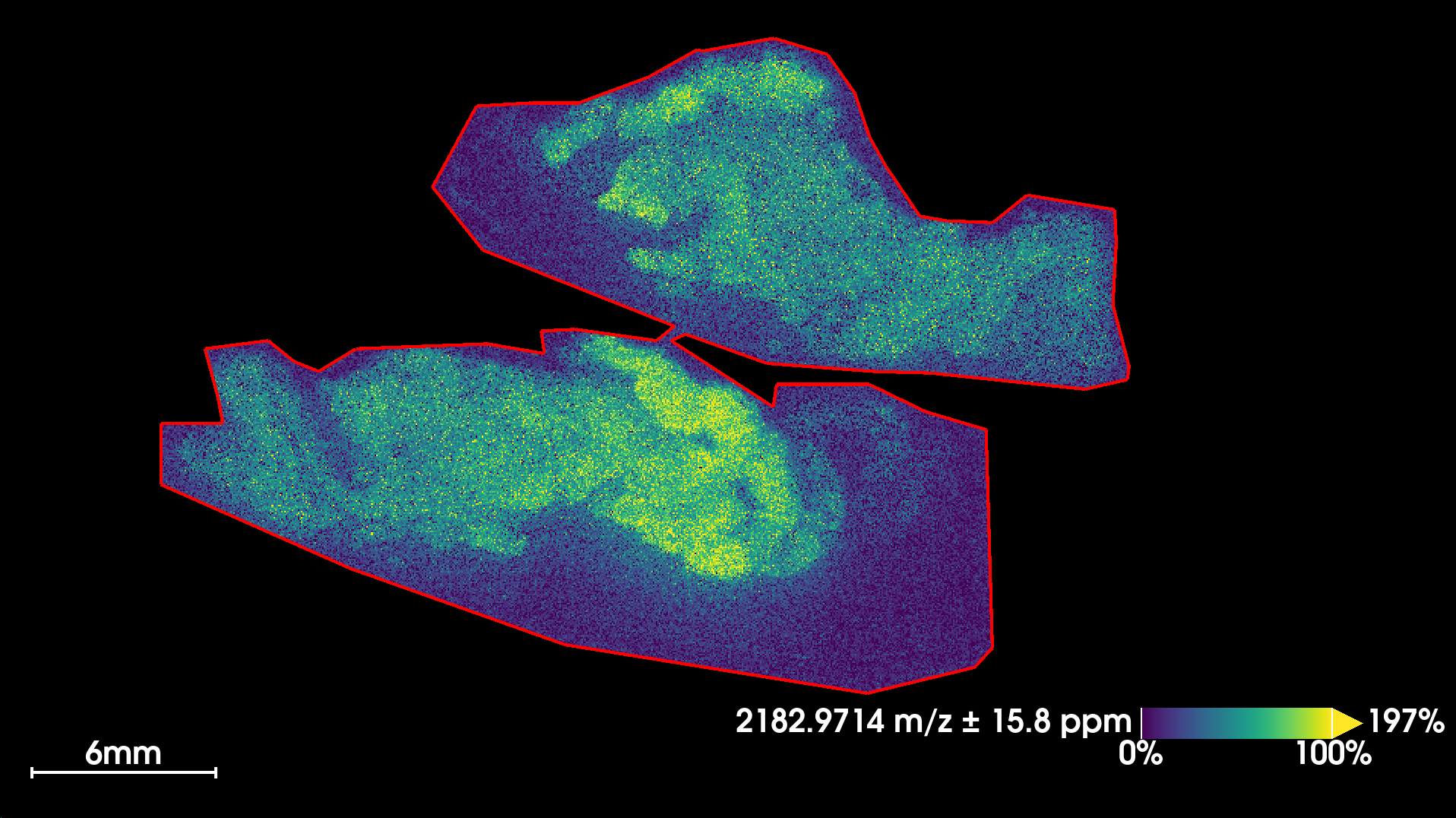

Supplement: Supplementary file 8 — Source Data 2 [file 41467_2026_72853_MOESM8_ESM.zip › Source Data MALDI Images/Supplementary Figure 14/2182.9714 mz ┬▒ 34.6 mDa.png]

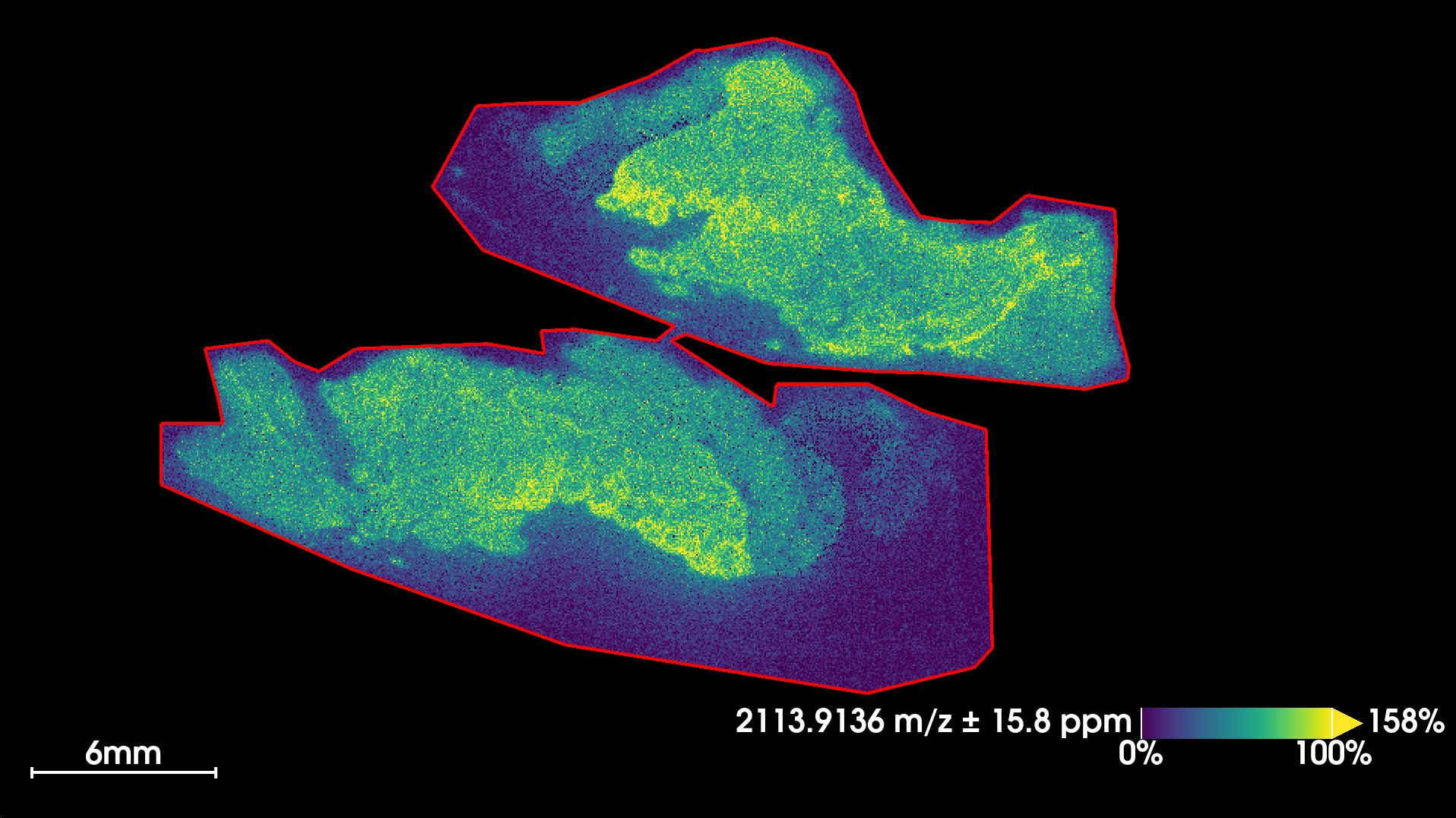

Supplement: Supplementary file 8 — Source Data 2 [file 41467_2026_72853_MOESM8_ESM.zip › Source Data MALDI Images/Supplementary Figure 14/2113.9136 mz ┬▒ 33.5 mDa.png]

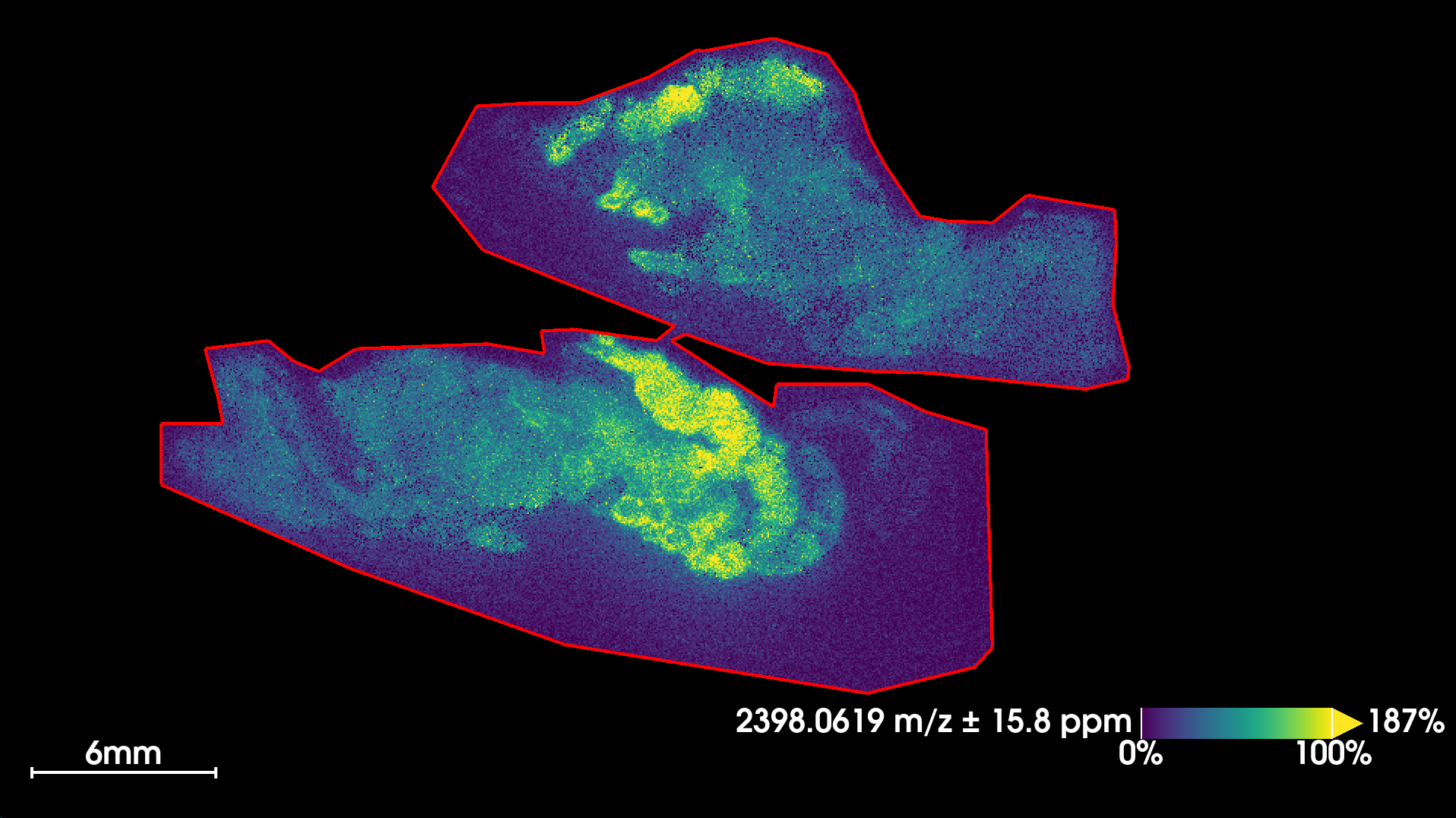

Supplement: Supplementary file 8 — Source Data 2 [file 41467_2026_72853_MOESM8_ESM.zip › Source Data MALDI Images/Supplementary Figure 14/2398.0619 mz ┬▒ 38 mDa.png]

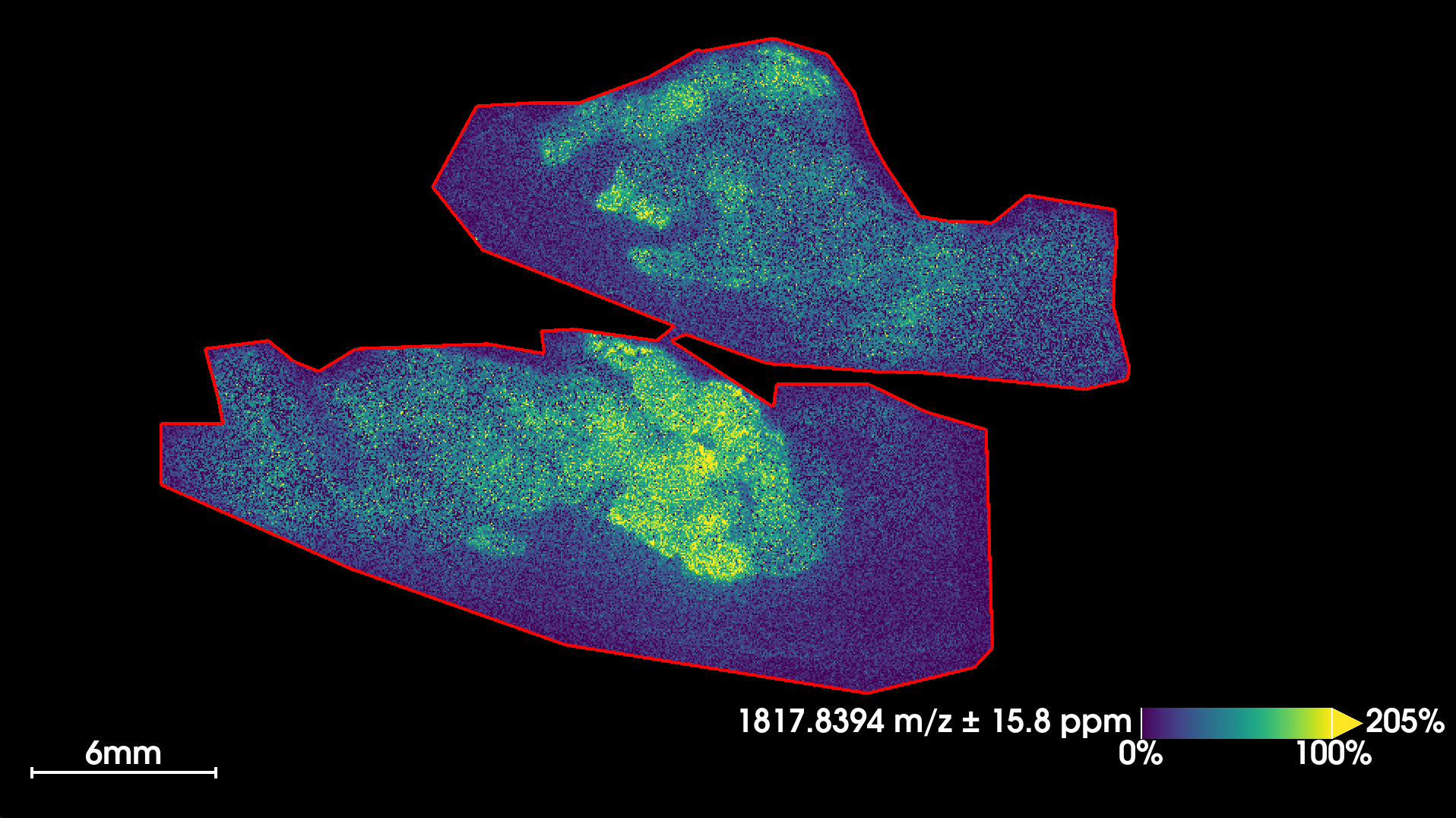

Supplement: Supplementary file 8 — Source Data 2 [file 41467_2026_72853_MOESM8_ESM.zip › Source Data MALDI Images/Supplementary Figure 14/1817.8394 mz ┬▒ 28.8 mDa.png]

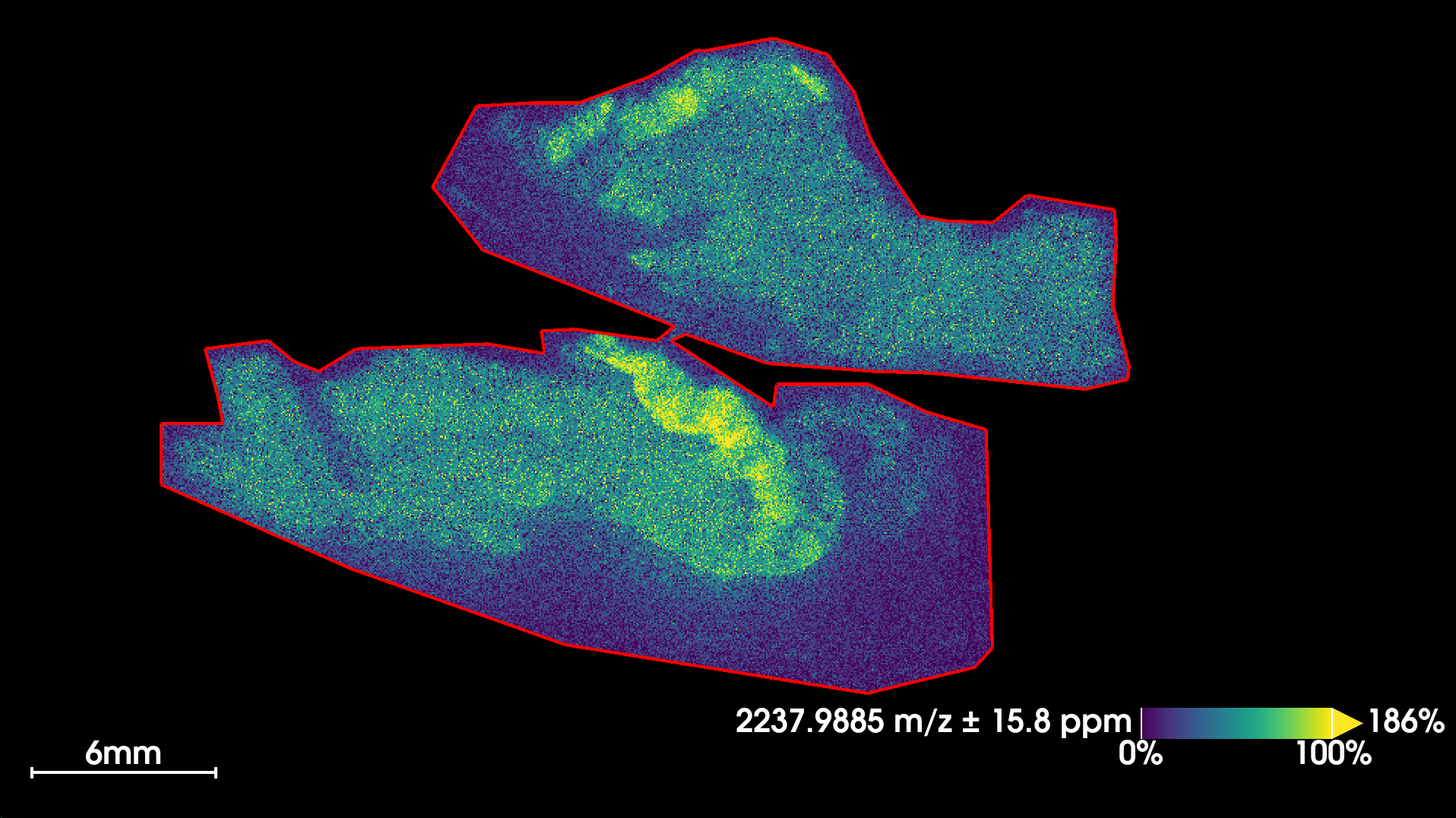

Supplement: Supplementary file 8 — Source Data 2 [file 41467_2026_72853_MOESM8_ESM.zip › Source Data MALDI Images/Supplementary Figure 14/2237.9885 mz ┬▒ 35.5 mDa.png]

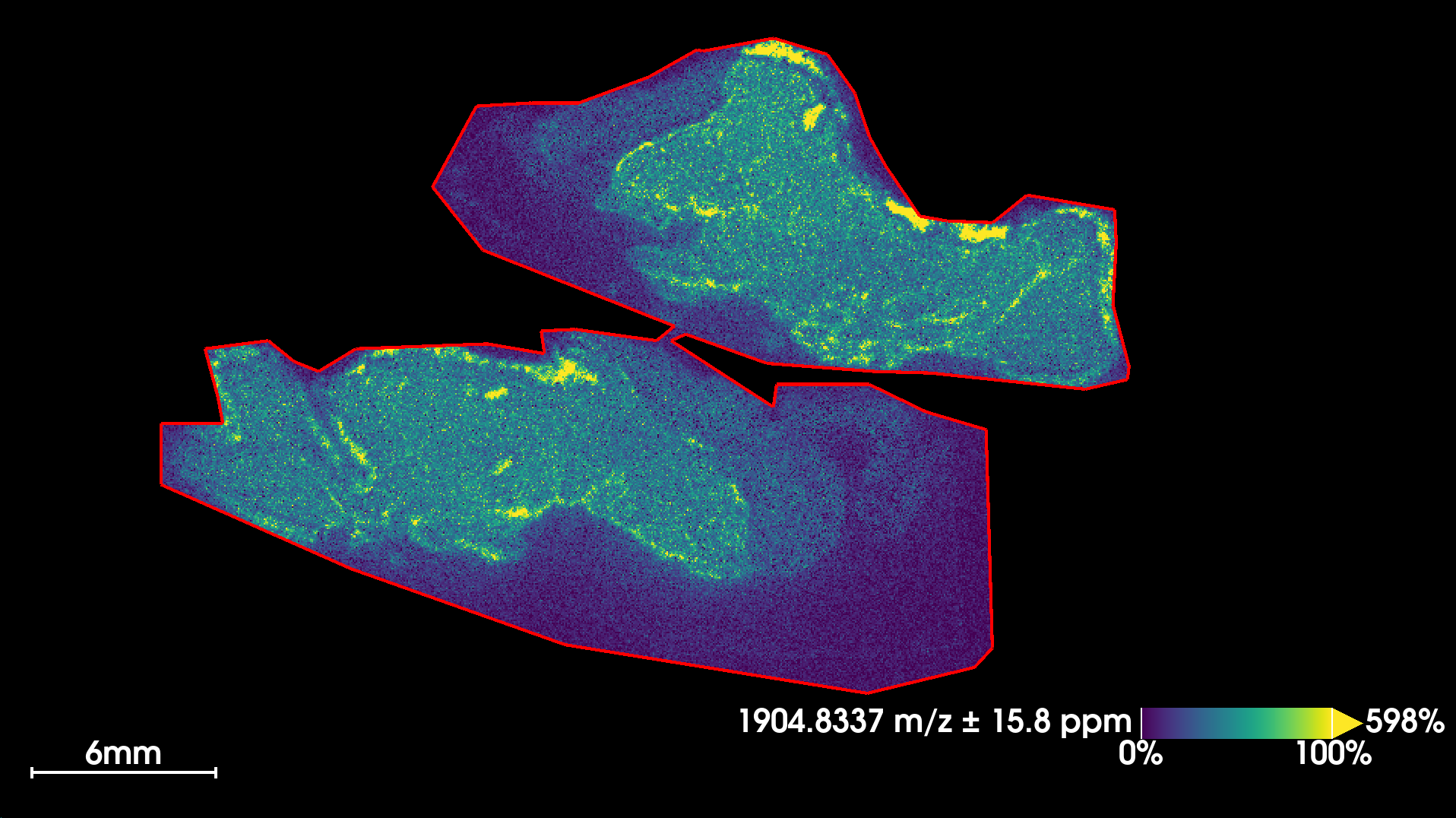

Supplement: Supplementary file 8 — Source Data 2 [file 41467_2026_72853_MOESM8_ESM.zip › Source Data MALDI Images/Supplementary Figure 14/1904.8337 mz ┬▒ 30.2 mDa.png]

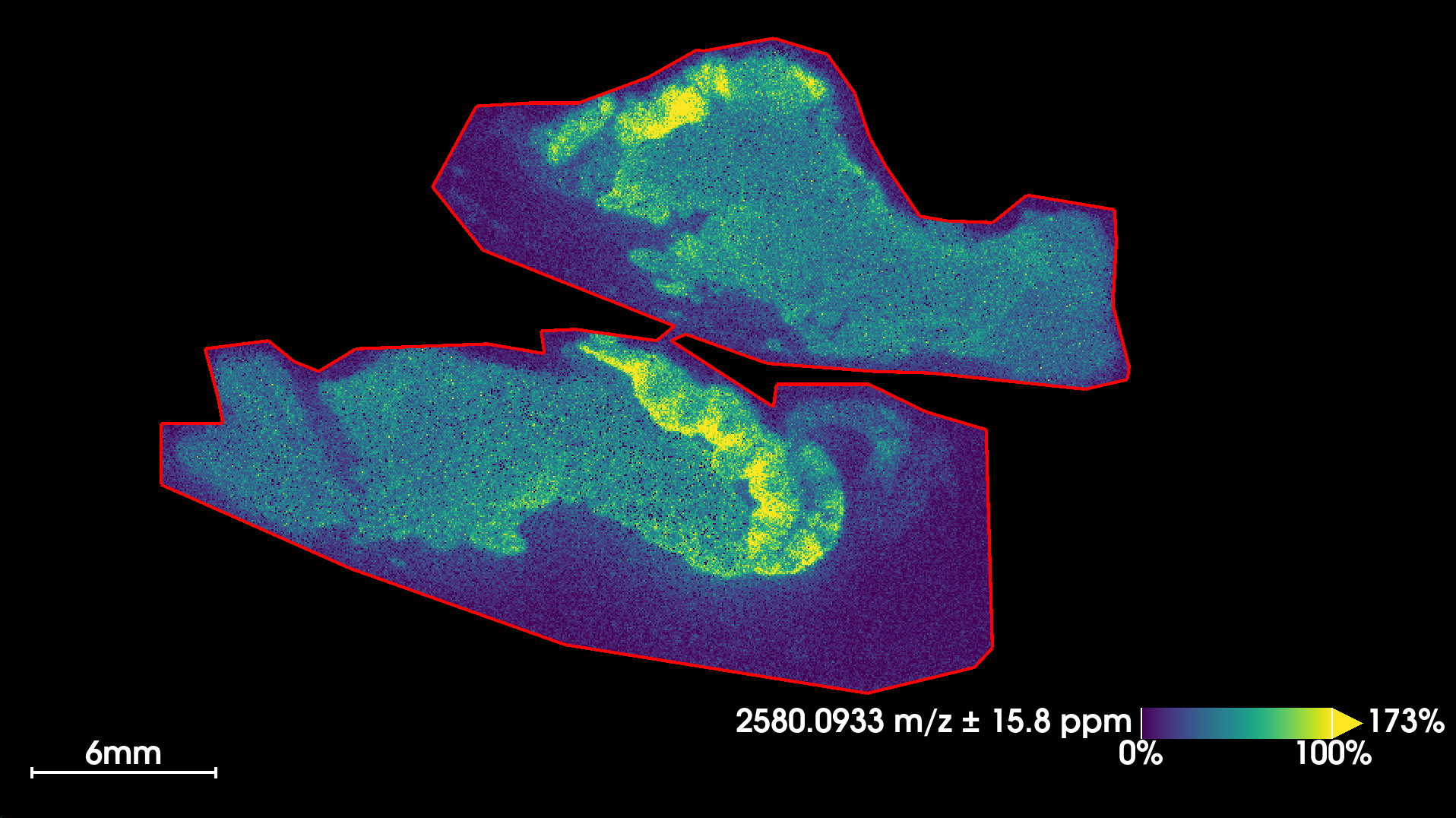

Supplement: Supplementary file 8 — Source Data 2 [file 41467_2026_72853_MOESM8_ESM.zip › Source Data MALDI Images/Supplementary Figure 14/2580.0933 mz ┬▒ 40.9 mDa.png]

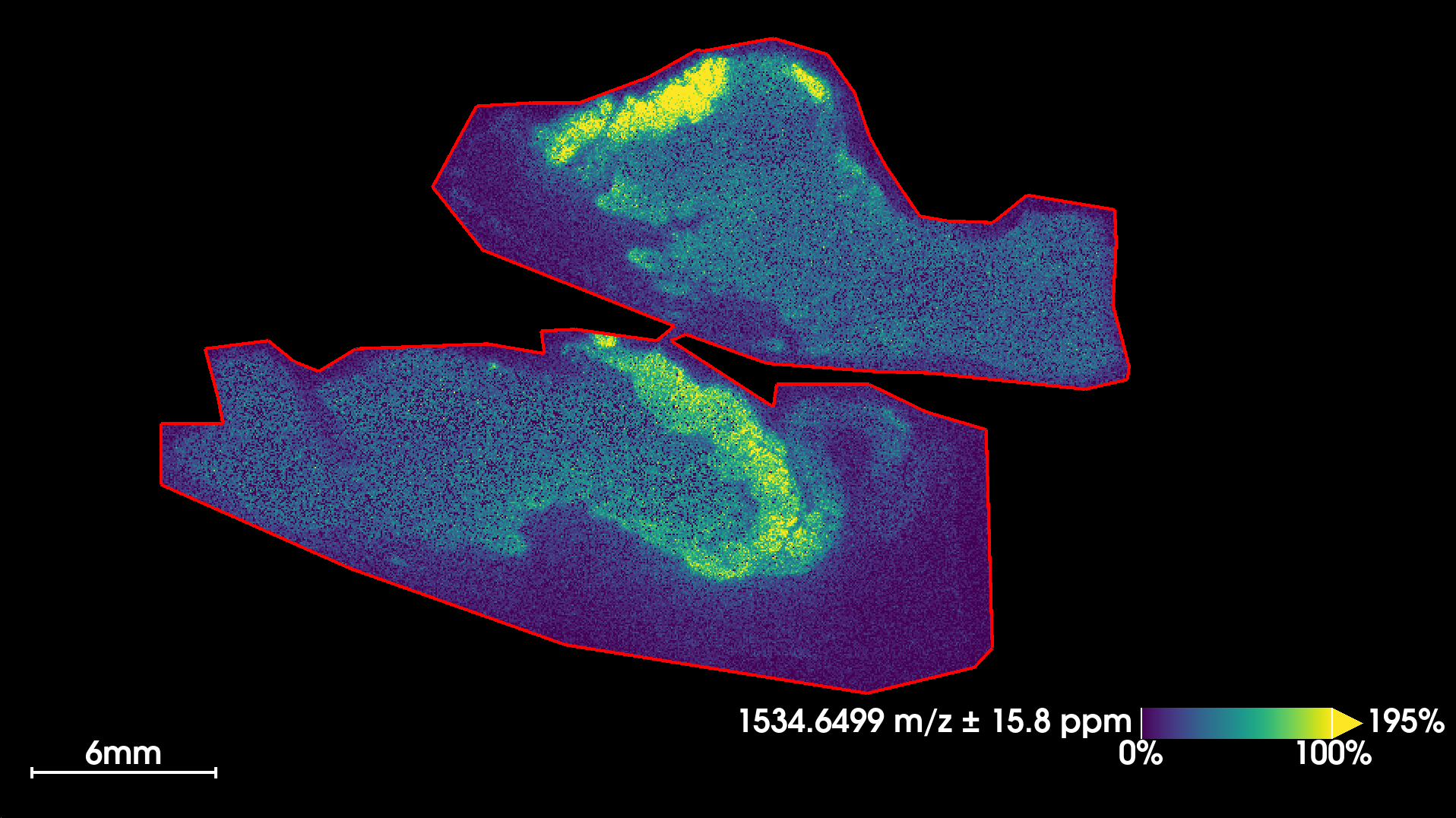

Supplement: Supplementary file 8 — Source Data 2 [file 41467_2026_72853_MOESM8_ESM.zip › Source Data MALDI Images/Supplementary Figure 14/1534.6499 mz ┬▒ 24.3 mDa.png]

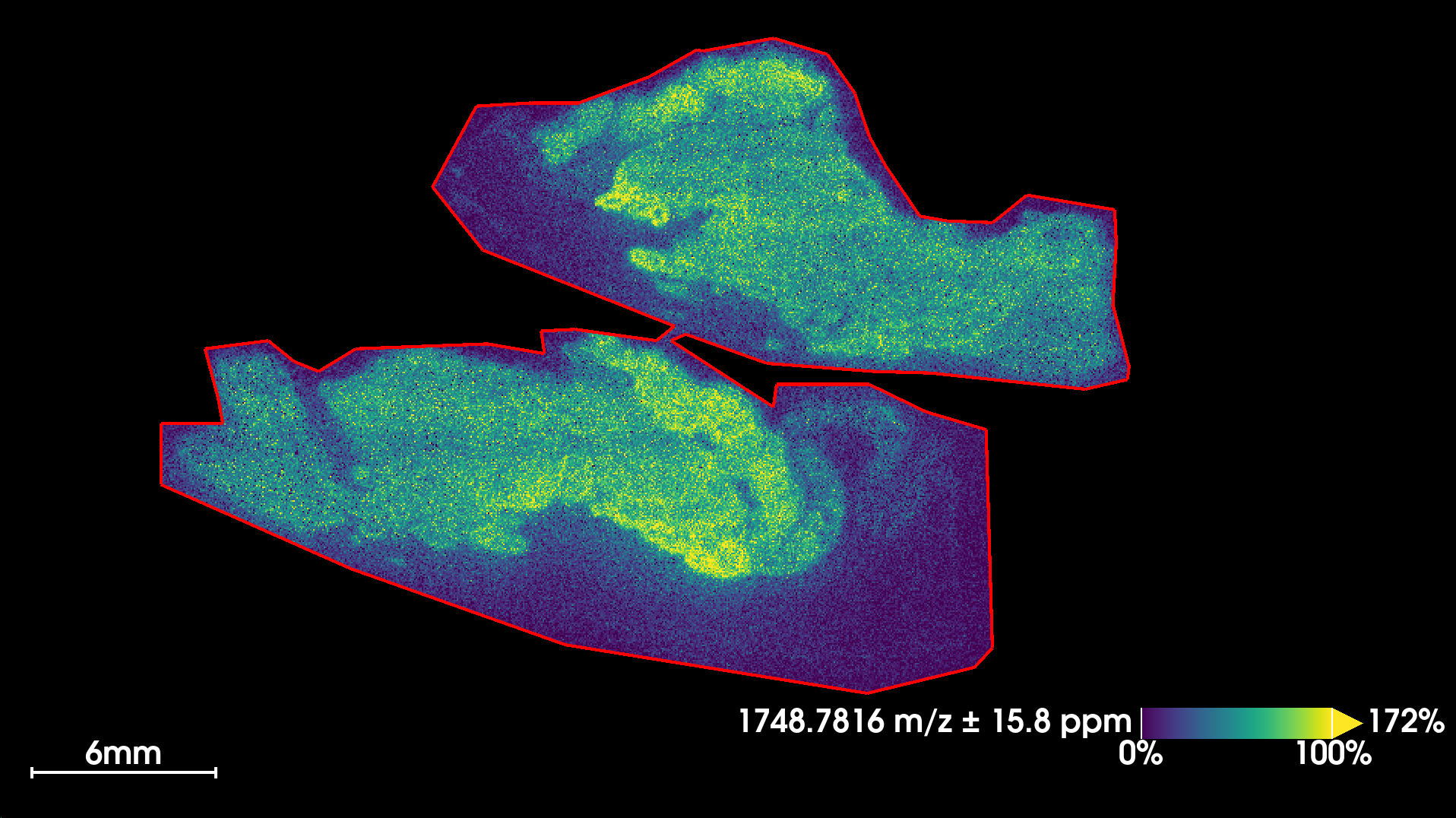

Supplement: Supplementary file 8 — Source Data 2 [file 41467_2026_72853_MOESM8_ESM.zip › Source Data MALDI Images/Supplementary Figure 14/1748.7816 mz ┬▒ 27.7 mDa.png]

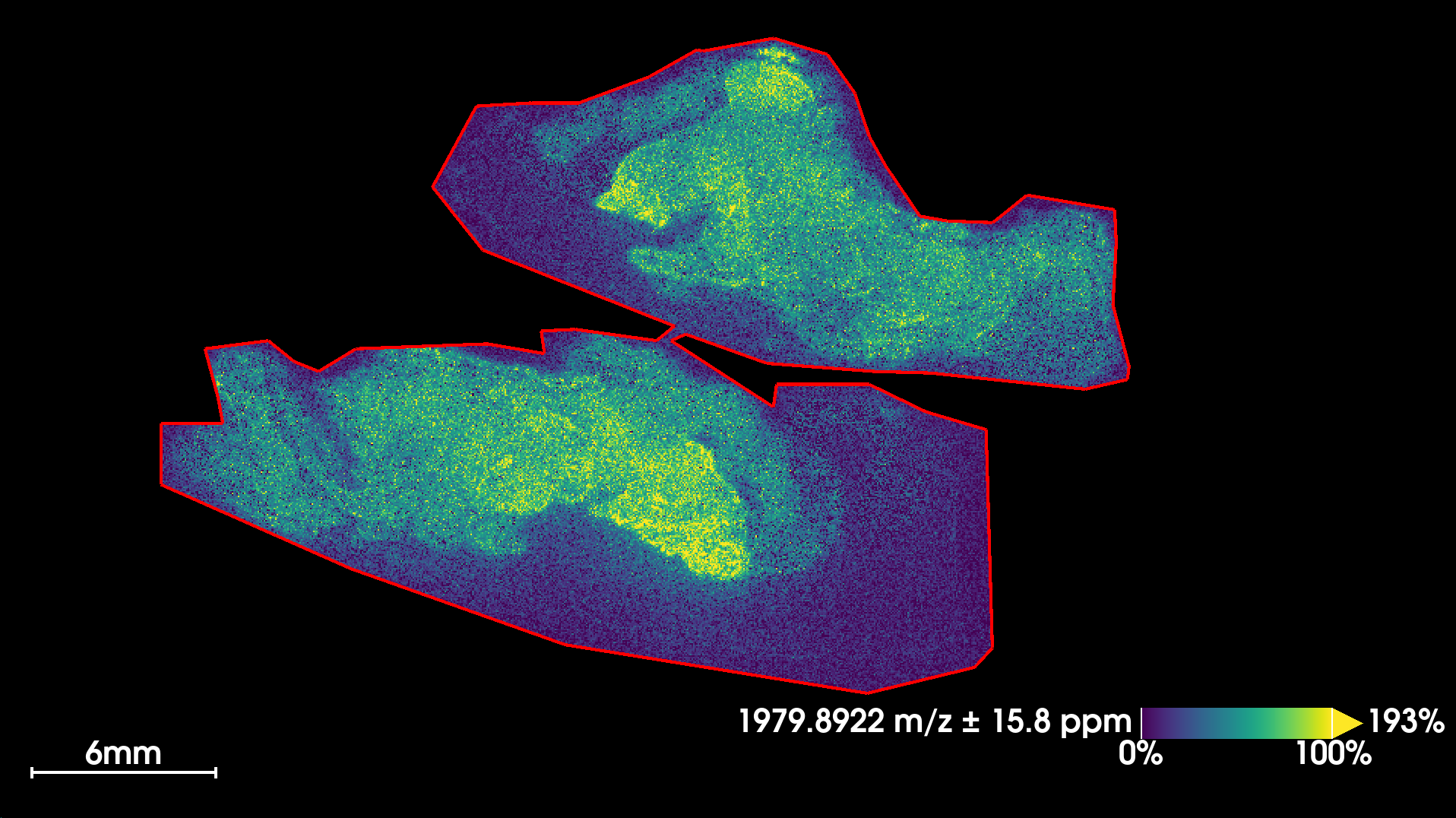

Supplement: Supplementary file 8 — Source Data 2 [file 41467_2026_72853_MOESM8_ESM.zip › Source Data MALDI Images/Supplementary Figure 14/1979.8922 mz ┬▒ 31.4 mDa.png]

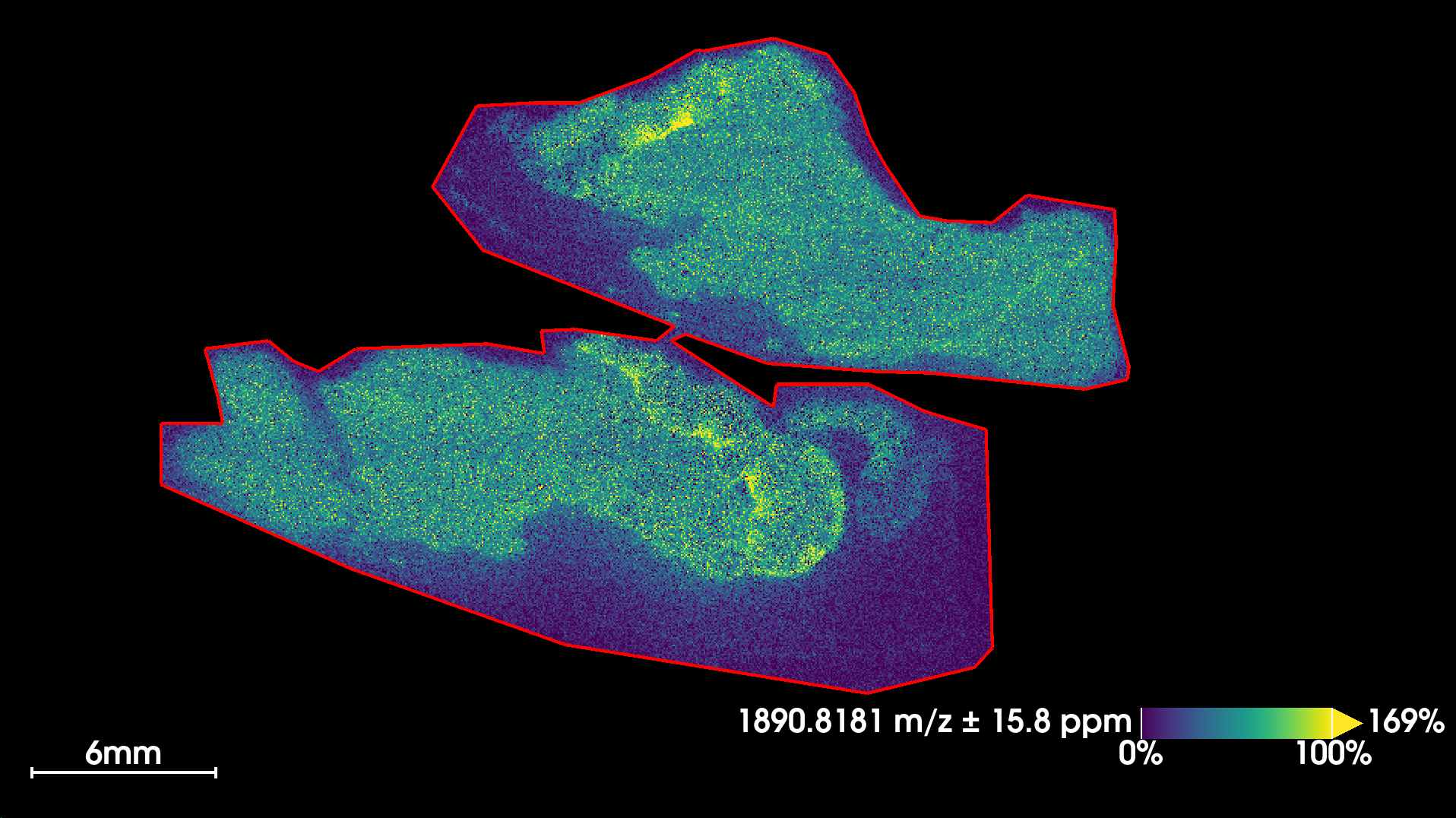

Supplement: Supplementary file 8 — Source Data 2 [file 41467_2026_72853_MOESM8_ESM.zip › Source Data MALDI Images/Supplementary Figure 14/1890.8181 mz ┬▒ 30 mDa.png]

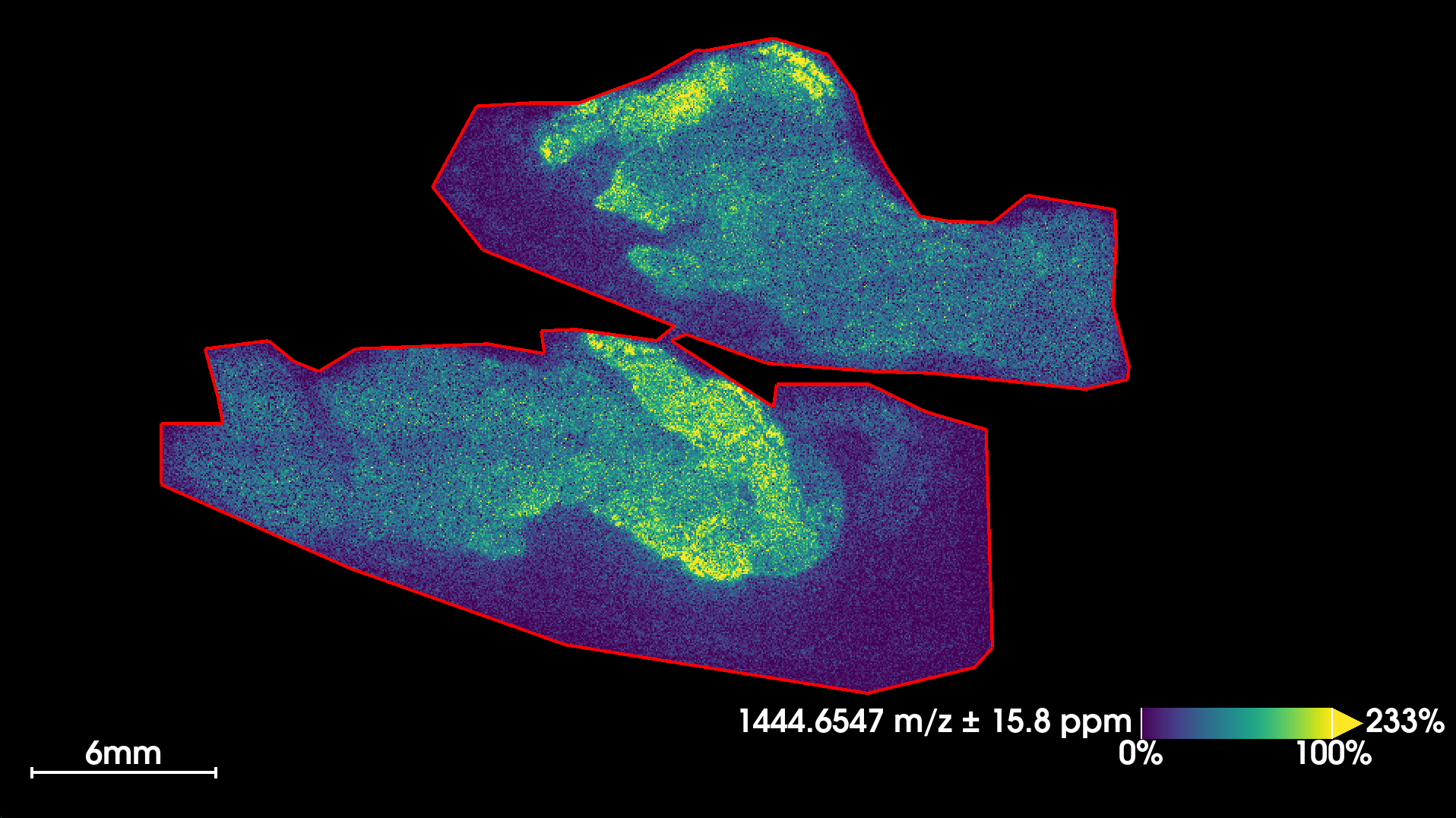

Supplement: Supplementary file 8 — Source Data 2 [file 41467_2026_72853_MOESM8_ESM.zip › Source Data MALDI Images/Supplementary Figure 14/1444.6547 mz ┬▒ 22.9 mDa.png]

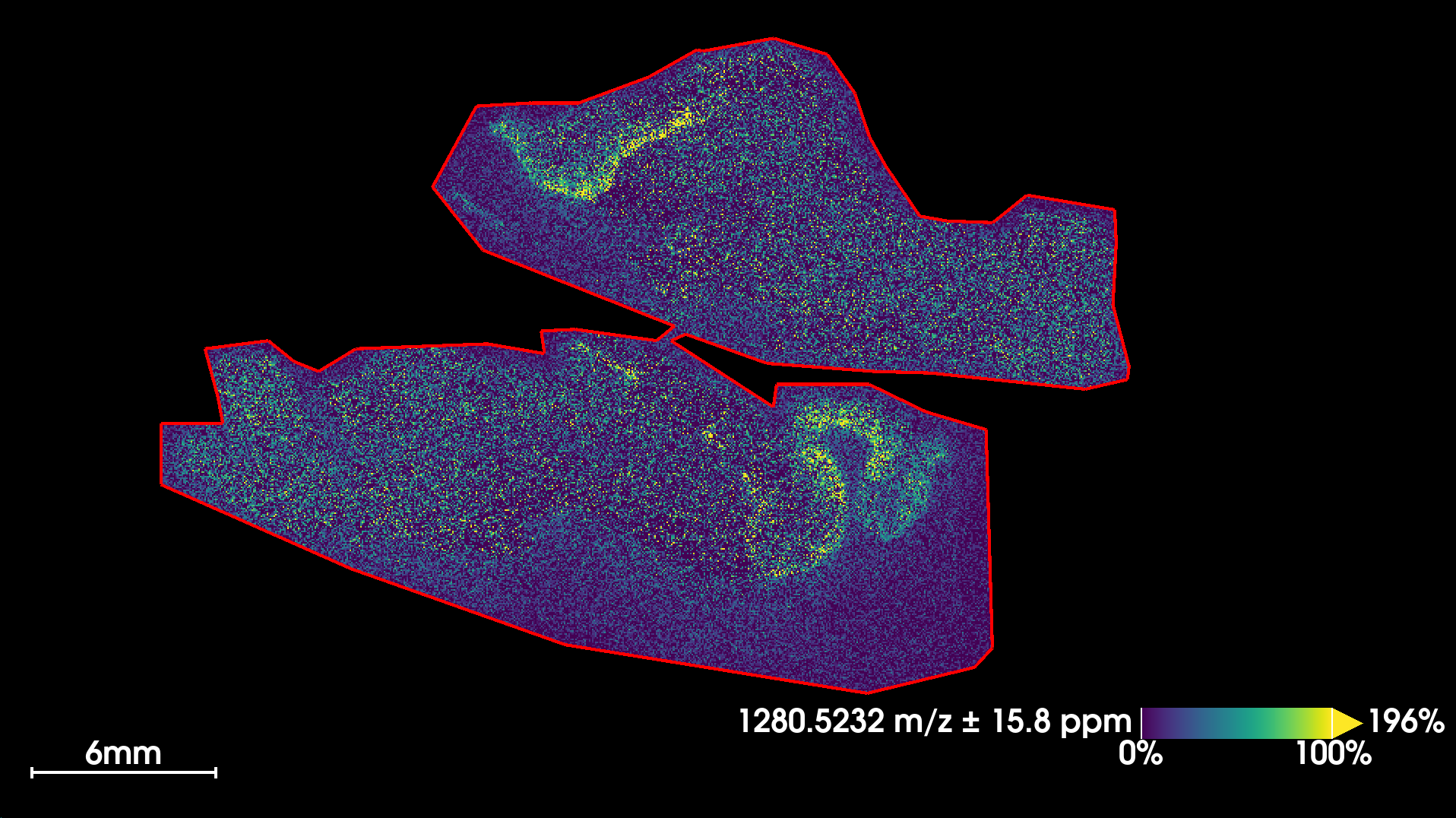

Supplement: Supplementary file 8 — Source Data 2 [file 41467_2026_72853_MOESM8_ESM.zip › Source Data MALDI Images/Supplementary Figure 14/1280.5232 mz ┬▒ 20.3 mDa.png]

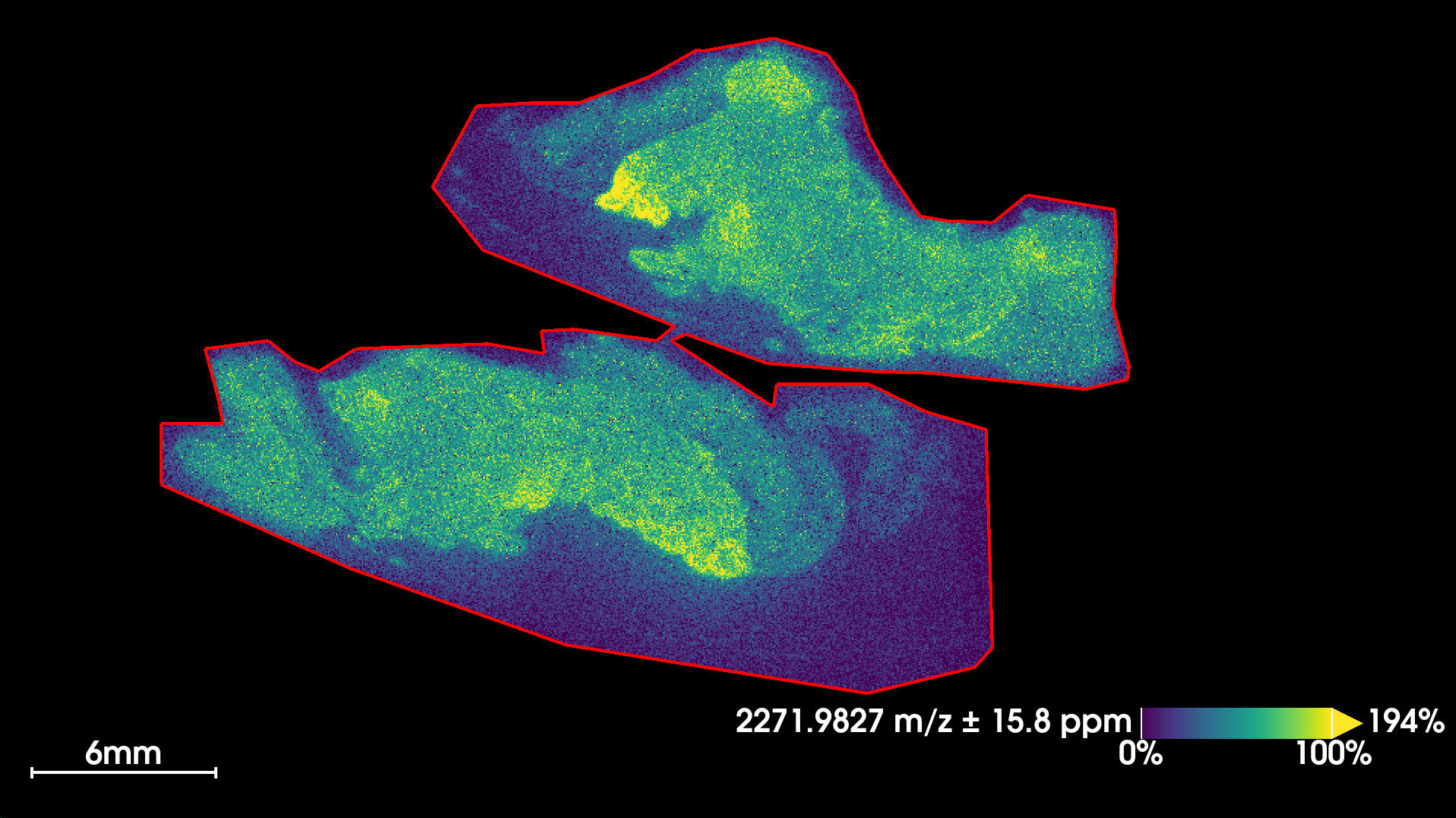

Supplement: Supplementary file 8 — Source Data 2 [file 41467_2026_72853_MOESM8_ESM.zip › Source Data MALDI Images/Supplementary Figure 14/2271.9827 mz ┬▒ 36 mDa.png]

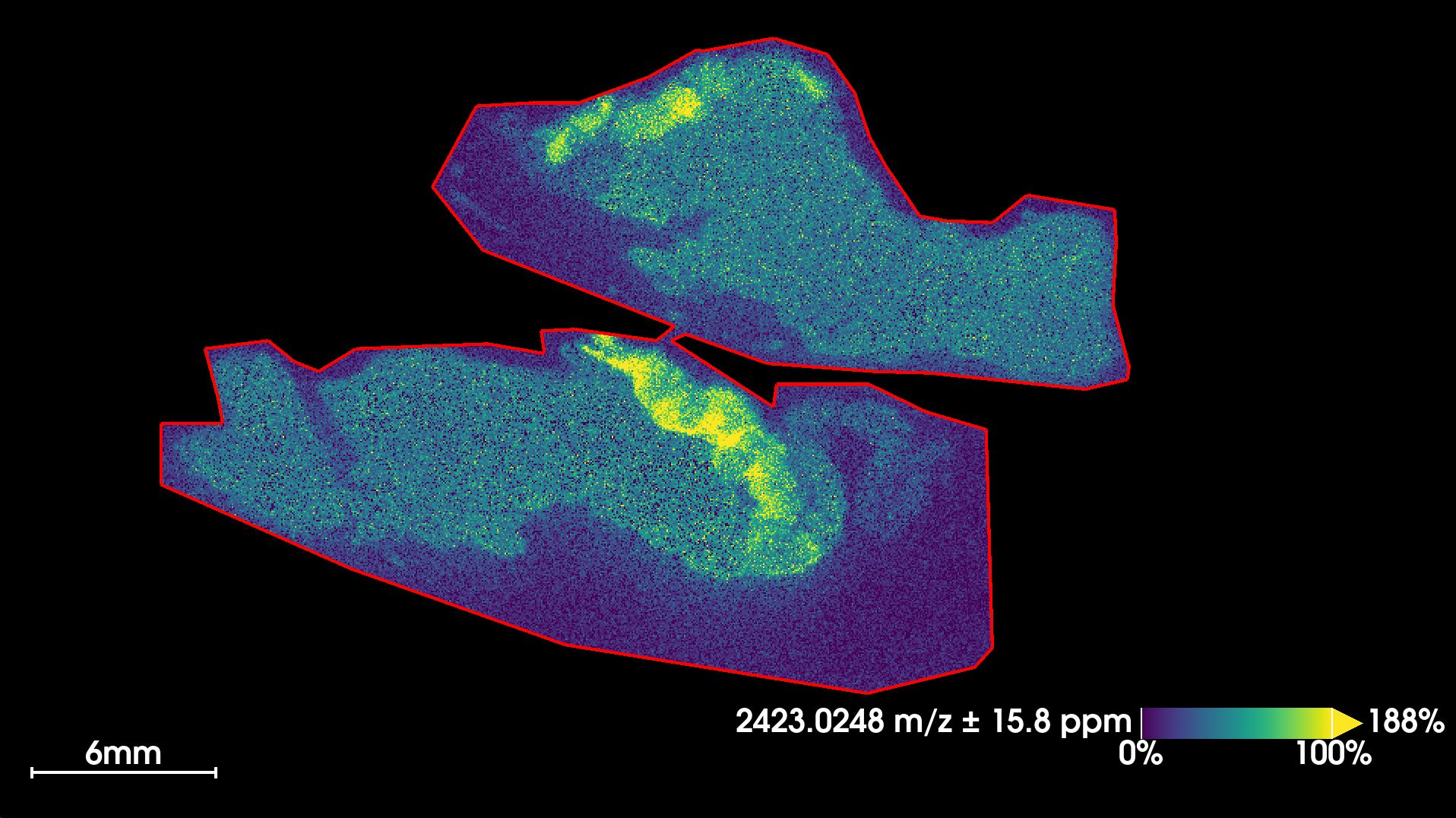

Supplement: Supplementary file 8 — Source Data 2 [file 41467_2026_72853_MOESM8_ESM.zip › Source Data MALDI Images/Supplementary Figure 14/2423.0248 mz ┬▒ 38.4 mDa.png]

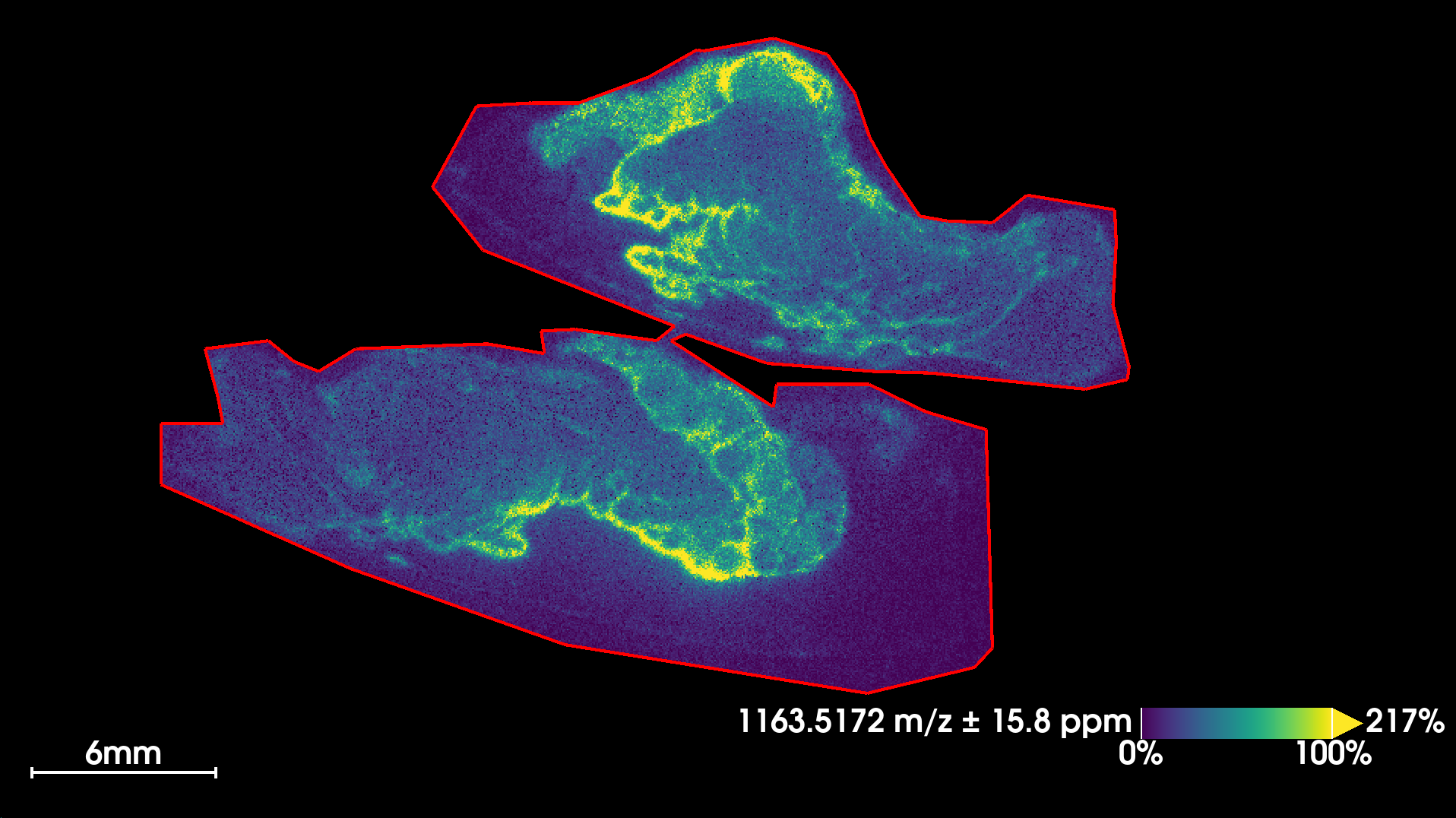

Supplement: Supplementary file 8 — Source Data 2 [file 41467_2026_72853_MOESM8_ESM.zip › Source Data MALDI Images/Supplementary Figure 14/1163.5172 mz ┬▒ 18.4 mDa.png]

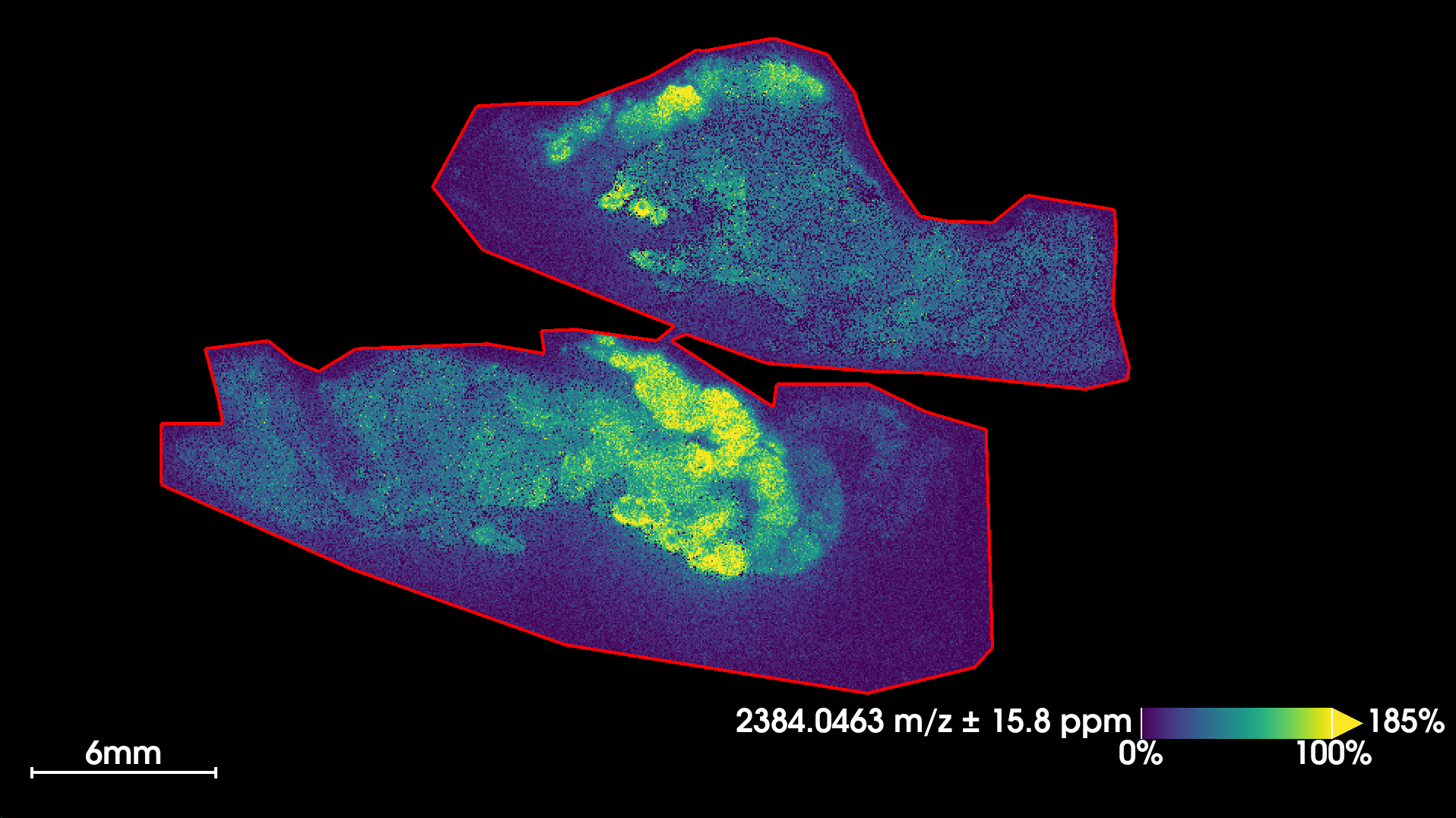

Supplement: Supplementary file 8 — Source Data 2 [file 41467_2026_72853_MOESM8_ESM.zip › Source Data MALDI Images/Supplementary Figure 14/2384.0463 mz ┬▒ 37.8 mDa.png]

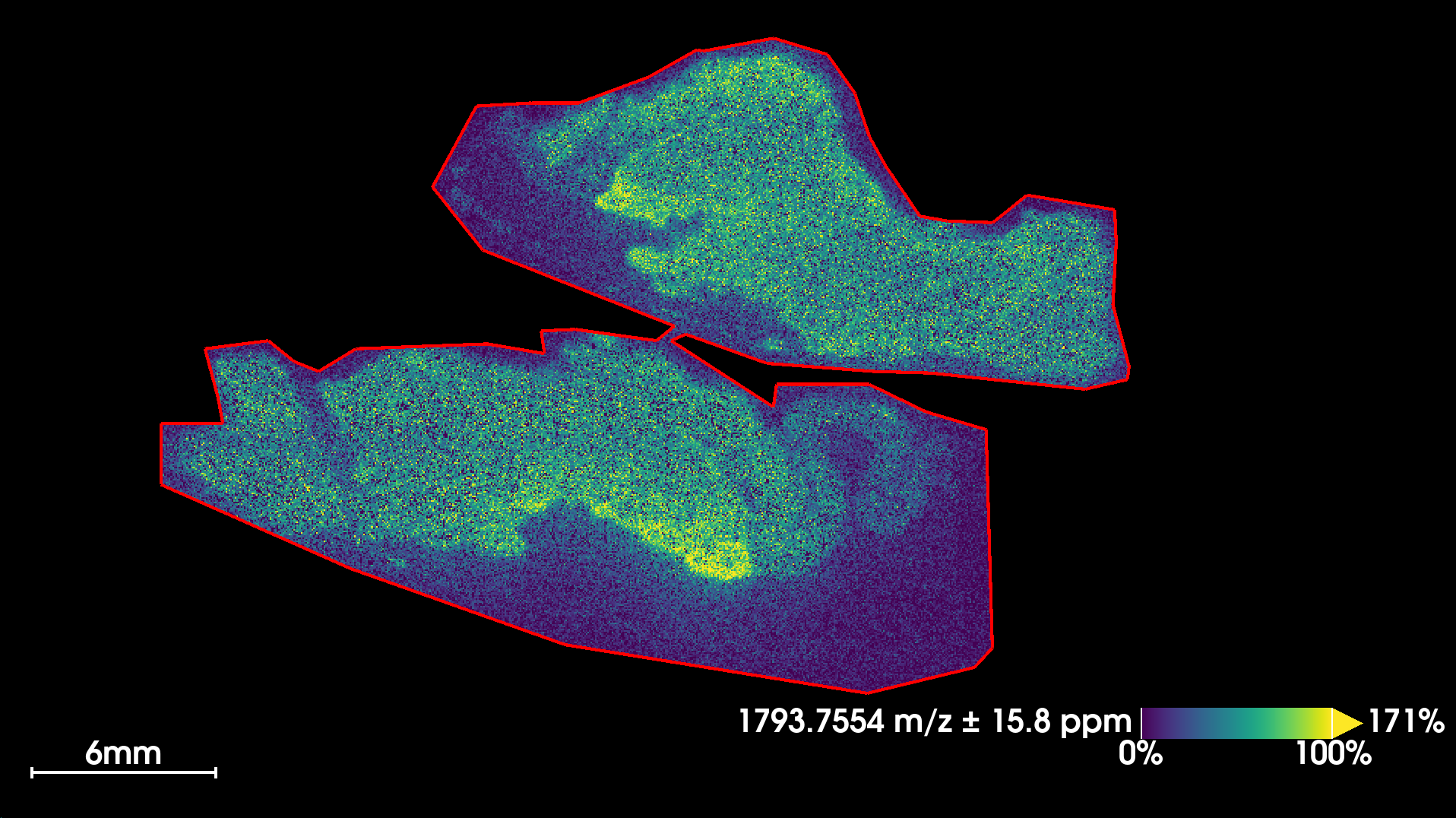

Supplement: Supplementary file 8 — Source Data 2 [file 41467_2026_72853_MOESM8_ESM.zip › Source Data MALDI Images/Supplementary Figure 14/1793.7554 mz ┬▒ 28.4 mDa.png]

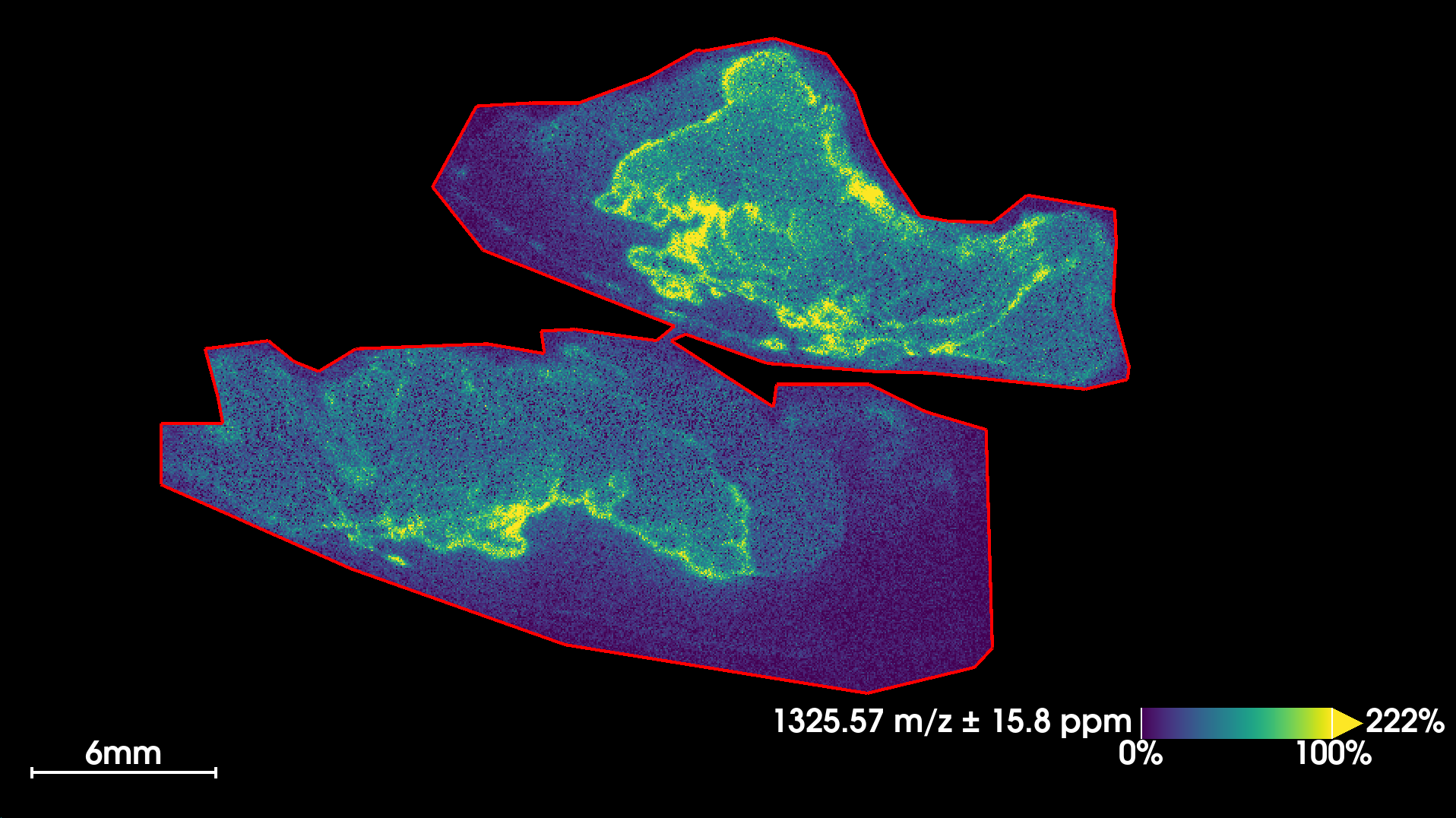

Supplement: Supplementary file 8 — Source Data 2 [file 41467_2026_72853_MOESM8_ESM.zip › Source Data MALDI Images/Supplementary Figure 14/1325.57 mz ┬▒ 21 mDa.png]

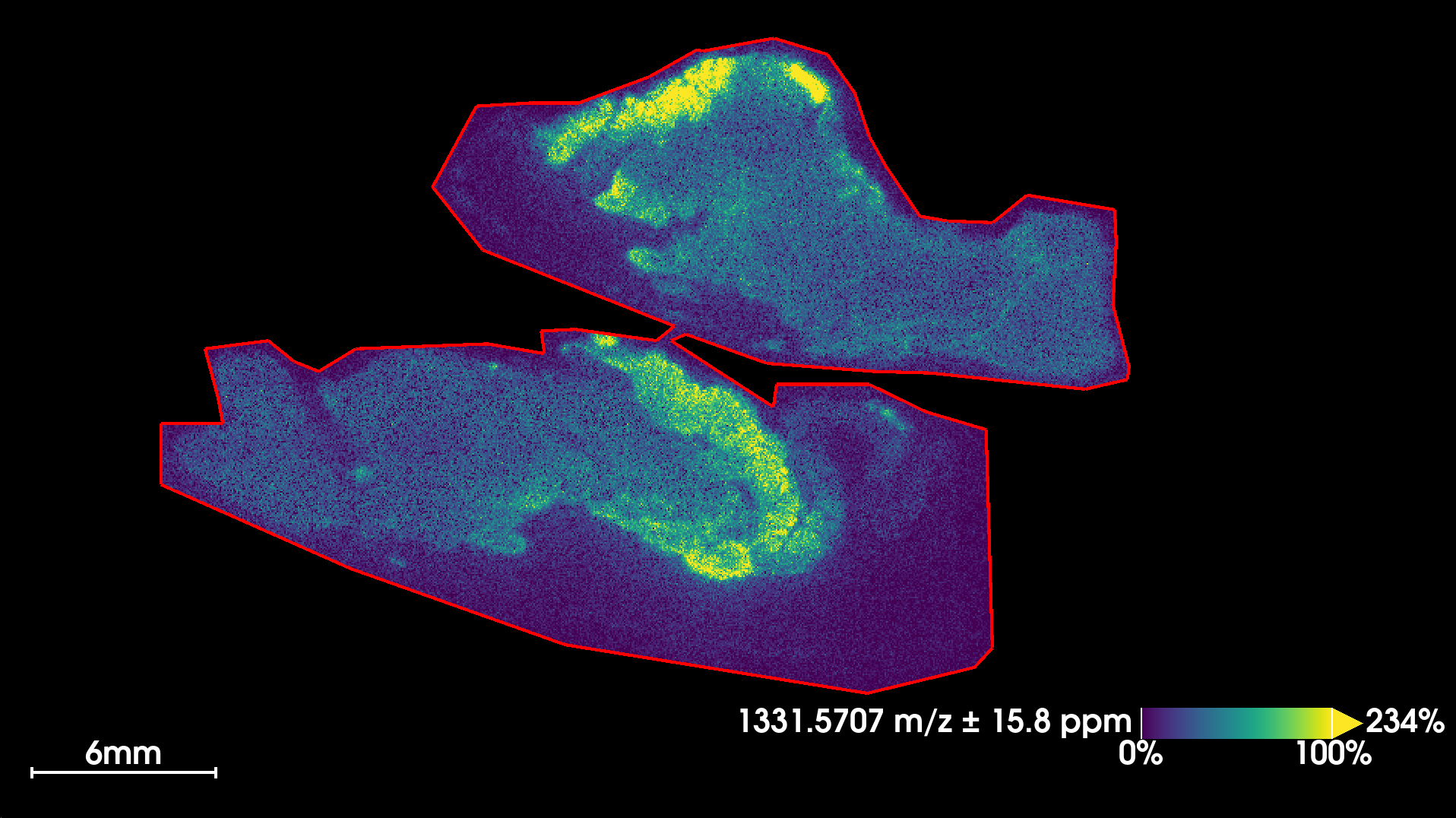

Supplement: Supplementary file 8 — Source Data 2 [file 41467_2026_72853_MOESM8_ESM.zip › Source Data MALDI Images/Supplementary Figure 14/1331.5707 mz ┬▒ 21.1 mDa.png]

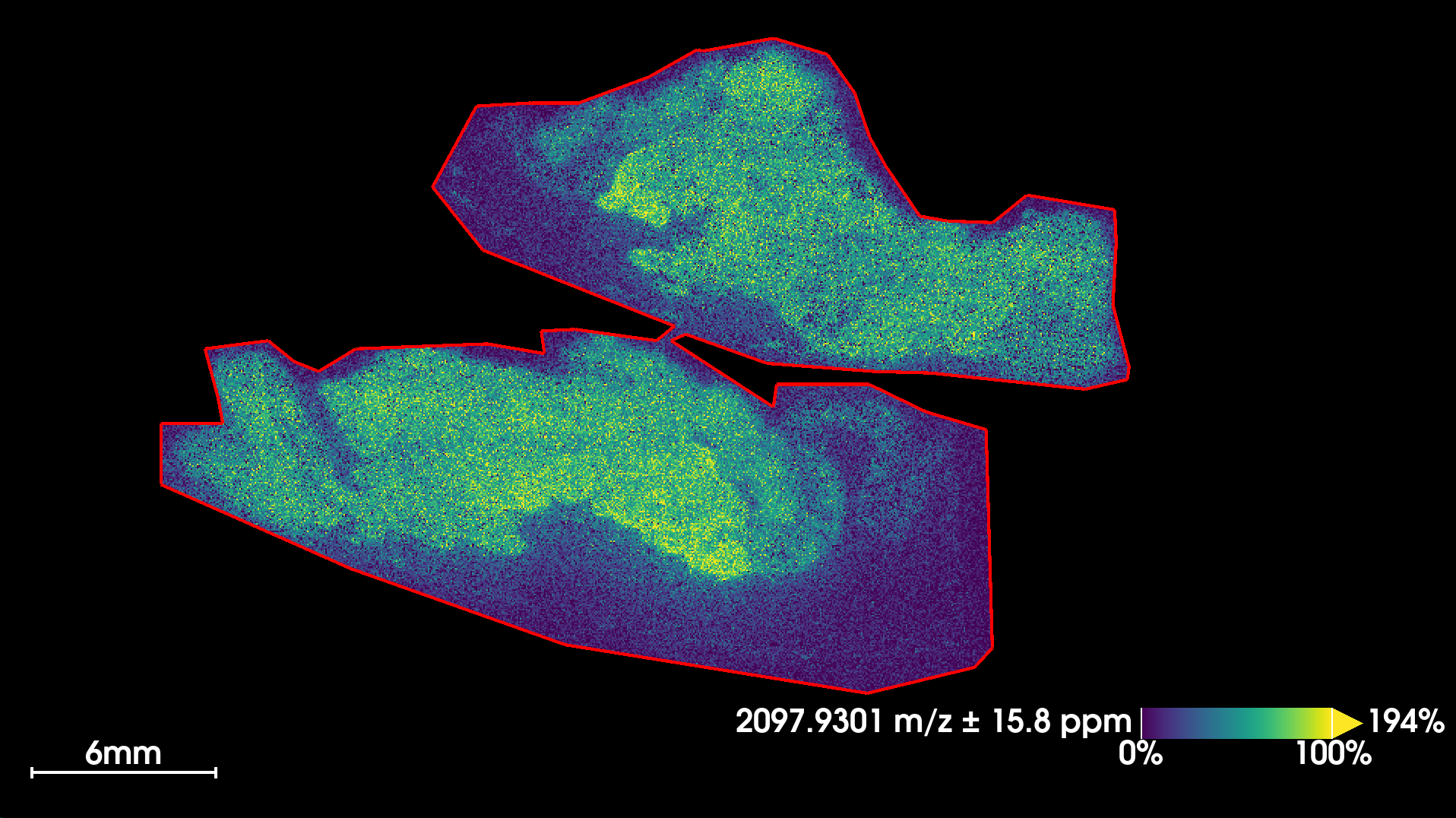

Supplement: Supplementary file 8 — Source Data 2 [file 41467_2026_72853_MOESM8_ESM.zip › Source Data MALDI Images/Supplementary Figure 14/2097.9301 mz ┬▒ 33.2 mDa.png]

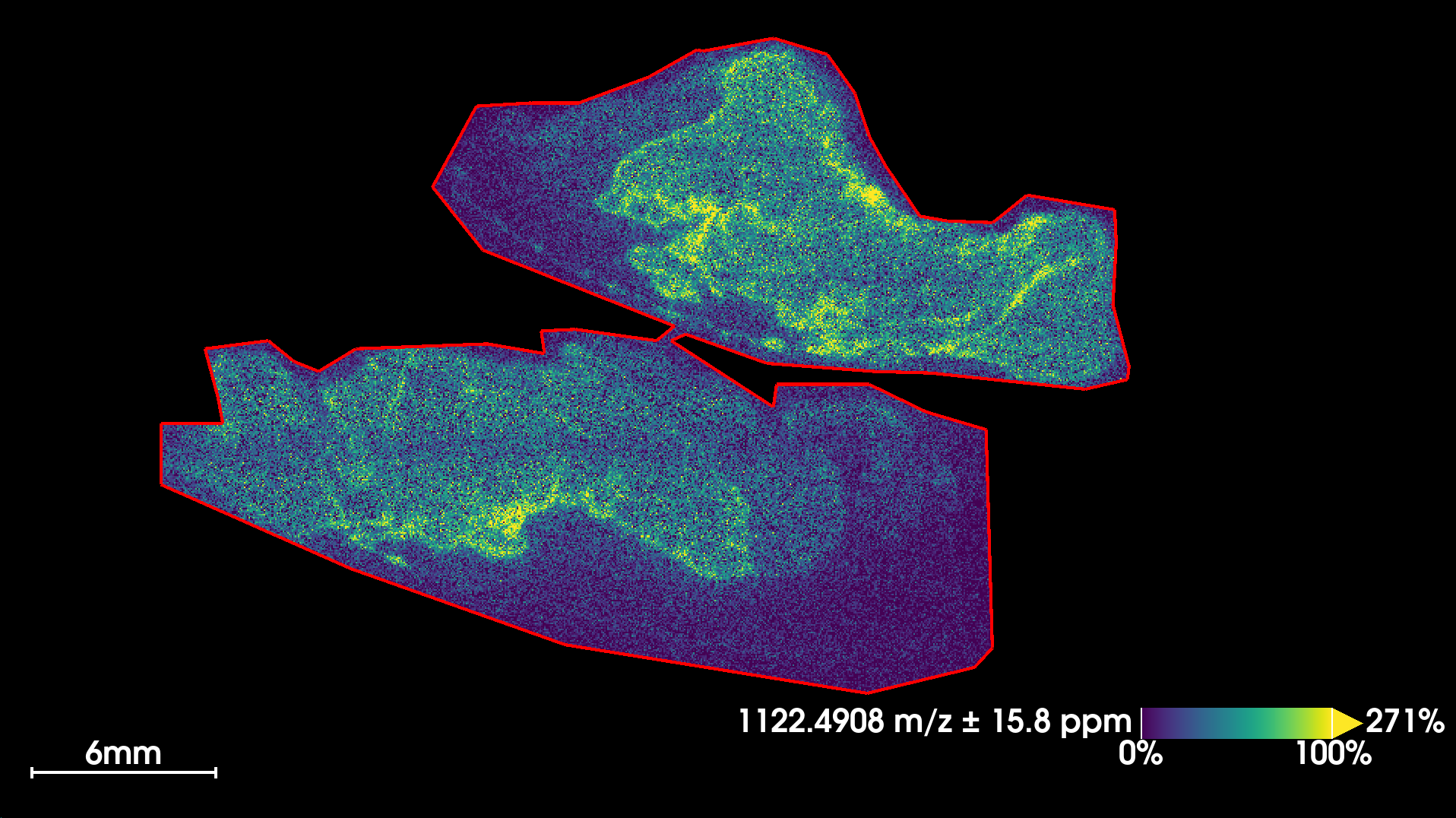

Supplement: Supplementary file 8 — Source Data 2 [file 41467_2026_72853_MOESM8_ESM.zip › Source Data MALDI Images/Supplementary Figure 14/1122.4908 mz ┬▒ 17.8 mDa.png]

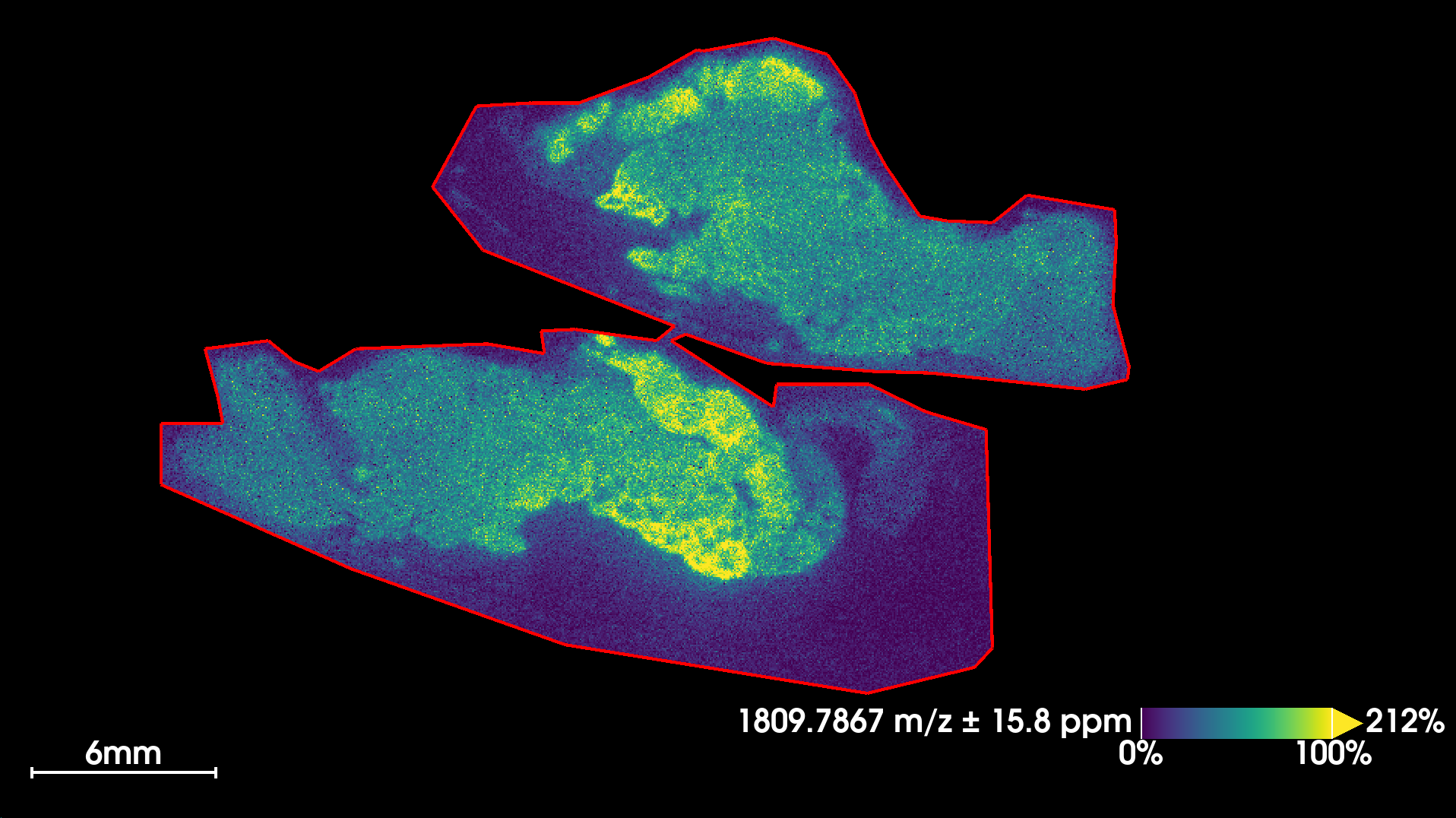

Supplement: Supplementary file 8 — Source Data 2 [file 41467_2026_72853_MOESM8_ESM.zip › Source Data MALDI Images/Supplementary Figure 14/1809.7867 mz ┬▒ 28.7 mDa.png]

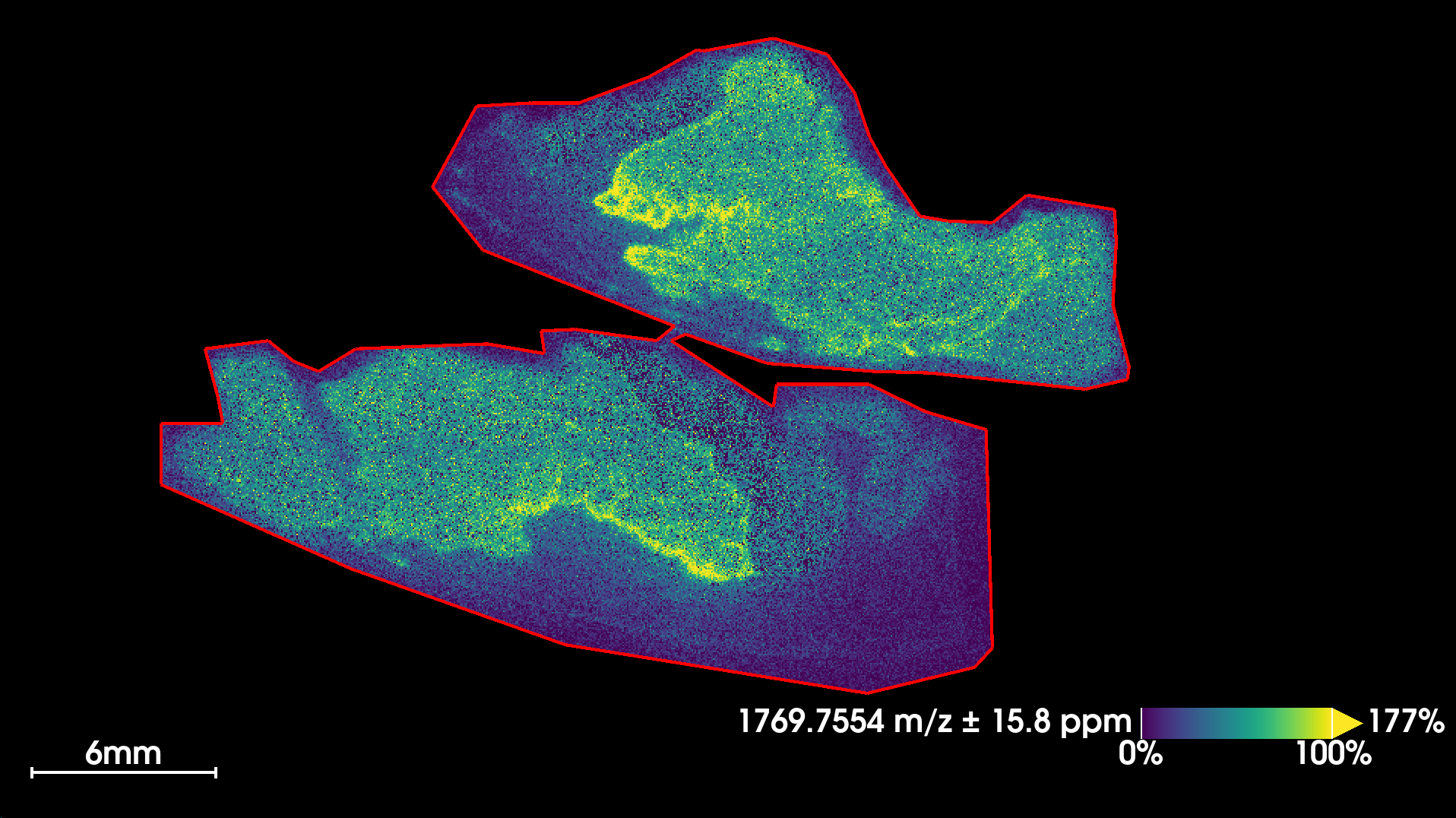

Supplement: Supplementary file 8 — Source Data 2 [file 41467_2026_72853_MOESM8_ESM.zip › Source Data MALDI Images/Supplementary Figure 14/1769.7554 mz ┬▒ 28 mDa.png]

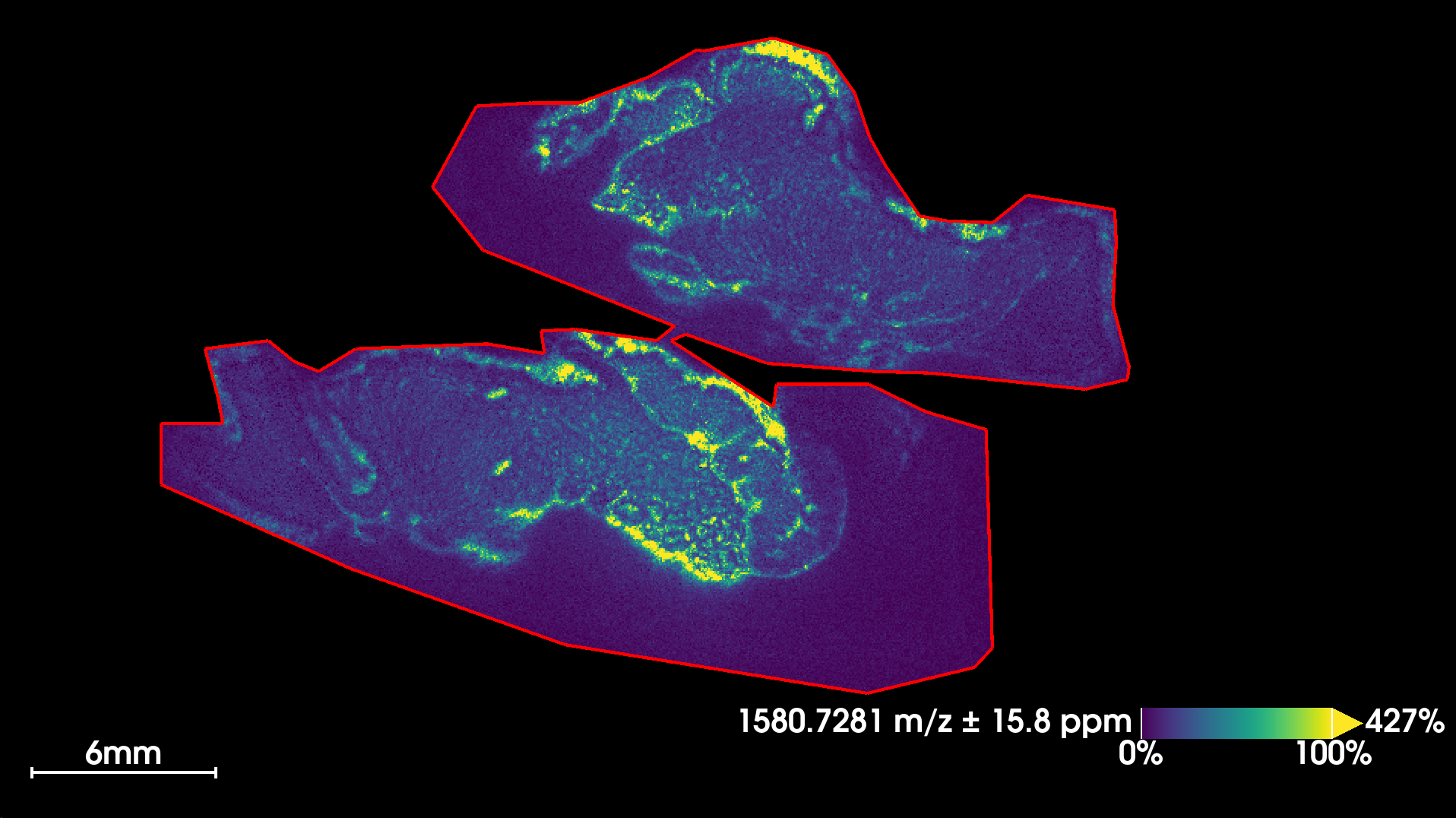

Supplement: Supplementary file 8 — Source Data 2 [file 41467_2026_72853_MOESM8_ESM.zip › Source Data MALDI Images/Supplementary Figure 14/1580.7281 mz ┬▒ 25 mDa.png]

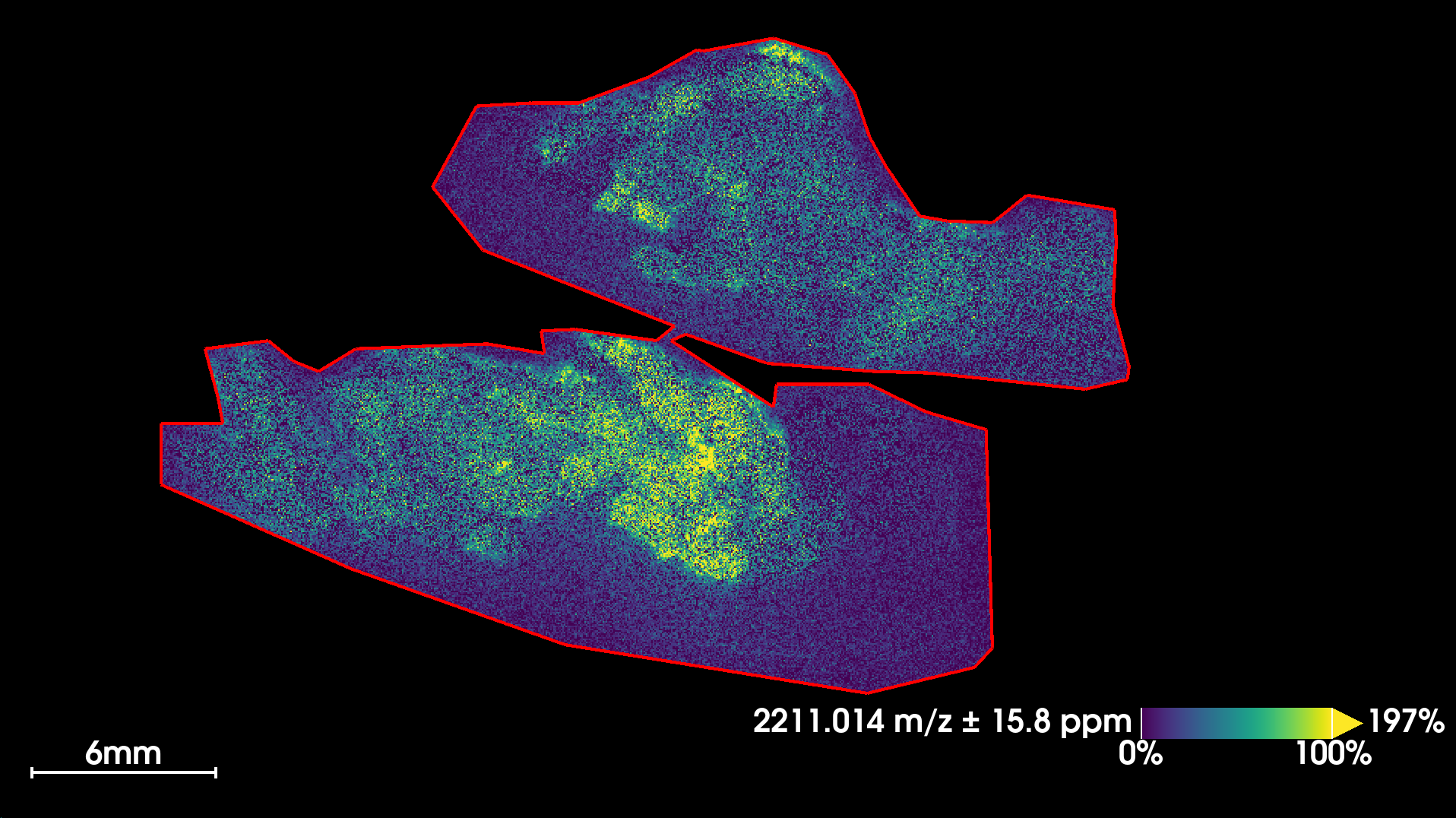

Supplement: Supplementary file 8 — Source Data 2 [file 41467_2026_72853_MOESM8_ESM.zip › Source Data MALDI Images/Supplementary Figure 14/2211.014 mz ┬▒ 35 mDa.png]

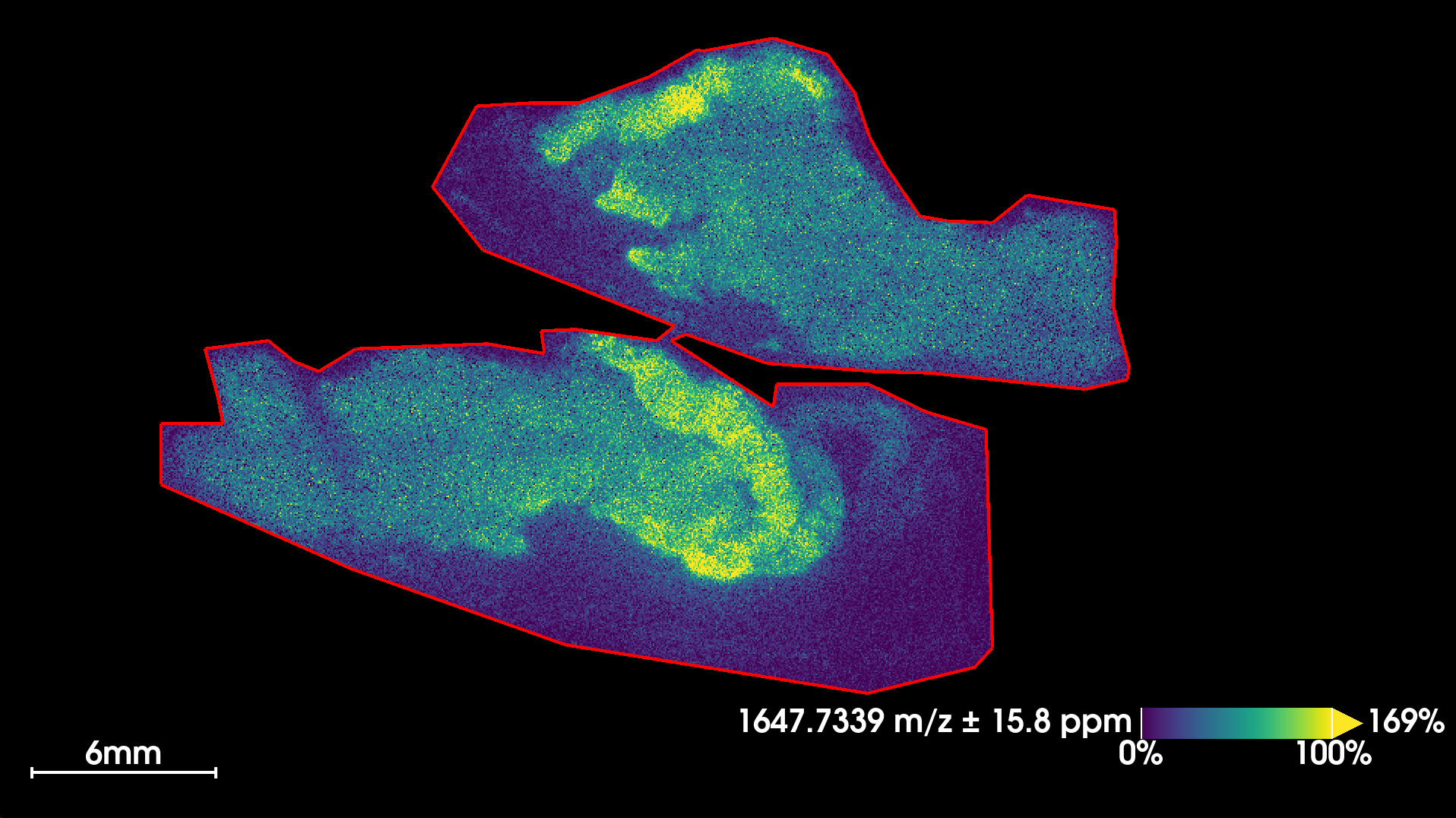

Supplement: Supplementary file 8 — Source Data 2 [file 41467_2026_72853_MOESM8_ESM.zip › Source Data MALDI Images/Supplementary Figure 14/1647.7339 mz ┬▒ 26.1 mDa.png]

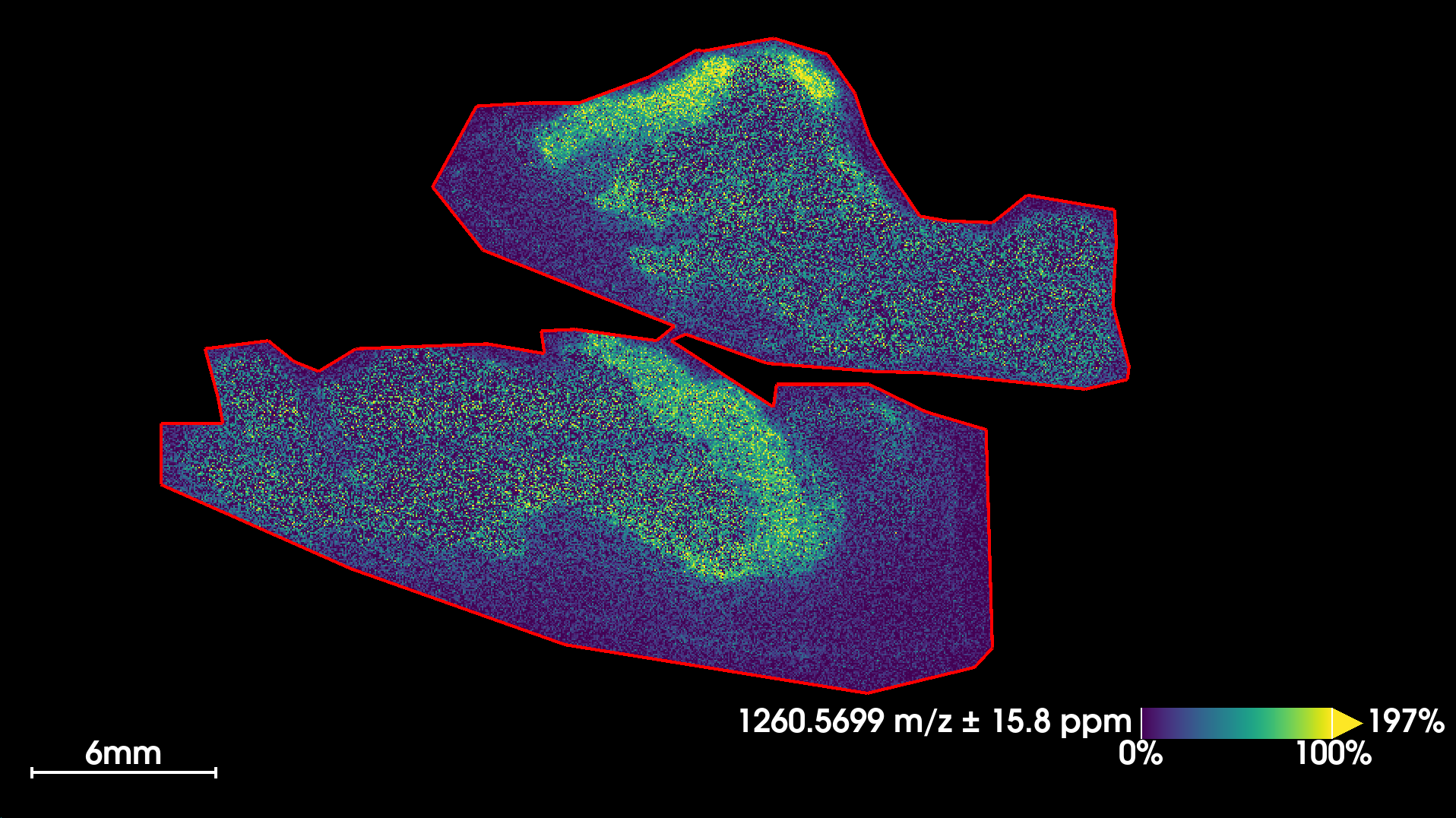

Supplement: Supplementary file 8 — Source Data 2 [file 41467_2026_72853_MOESM8_ESM.zip › Source Data MALDI Images/Supplementary Figure 14/1260.5699 mz ┬▒ 20 mDa.png]

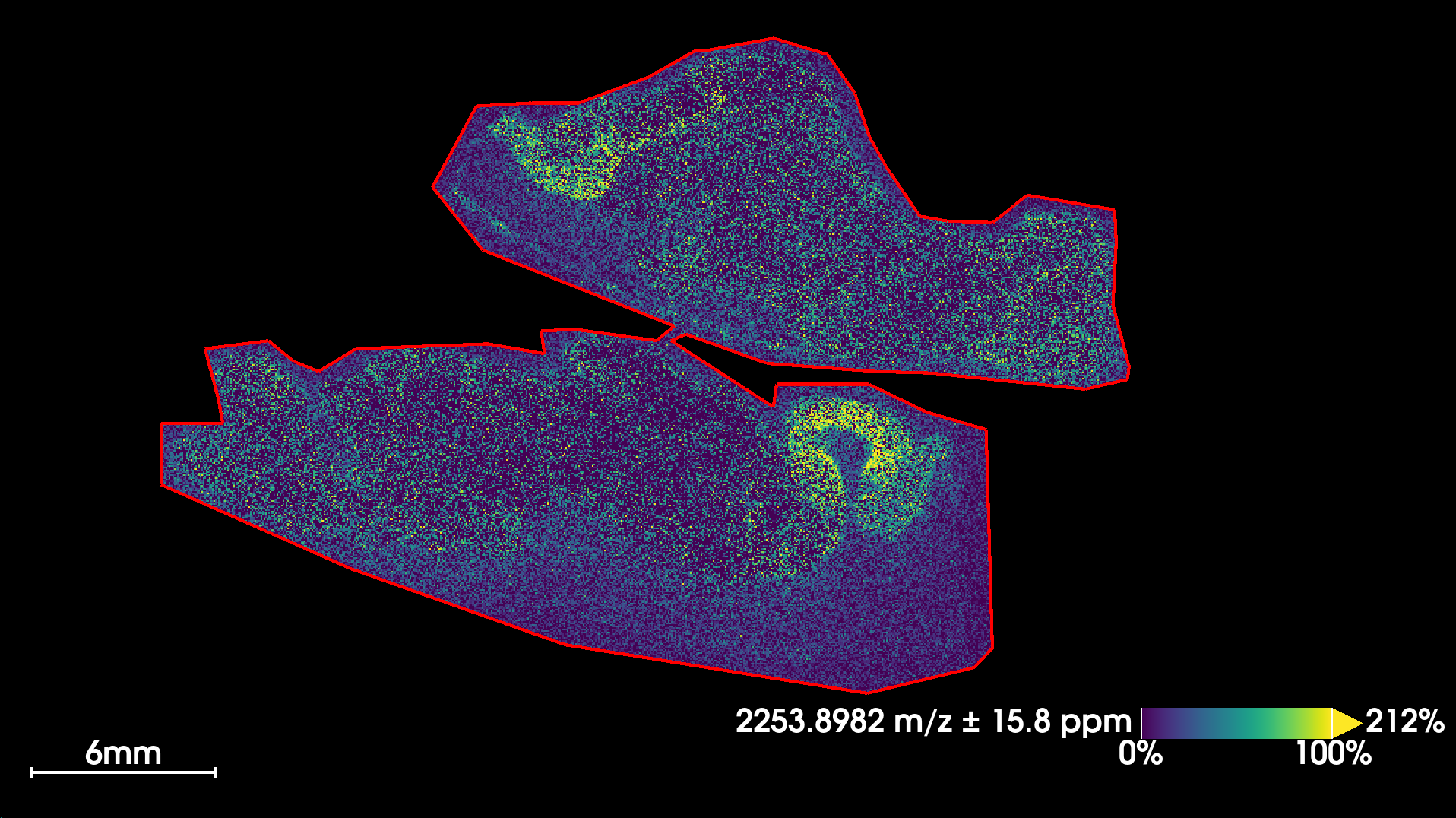

Supplement: Supplementary file 8 — Source Data 2 [file 41467_2026_72853_MOESM8_ESM.zip › Source Data MALDI Images/Supplementary Figure 14/2253.8982 mz ┬▒ 35.7 mDa.png]

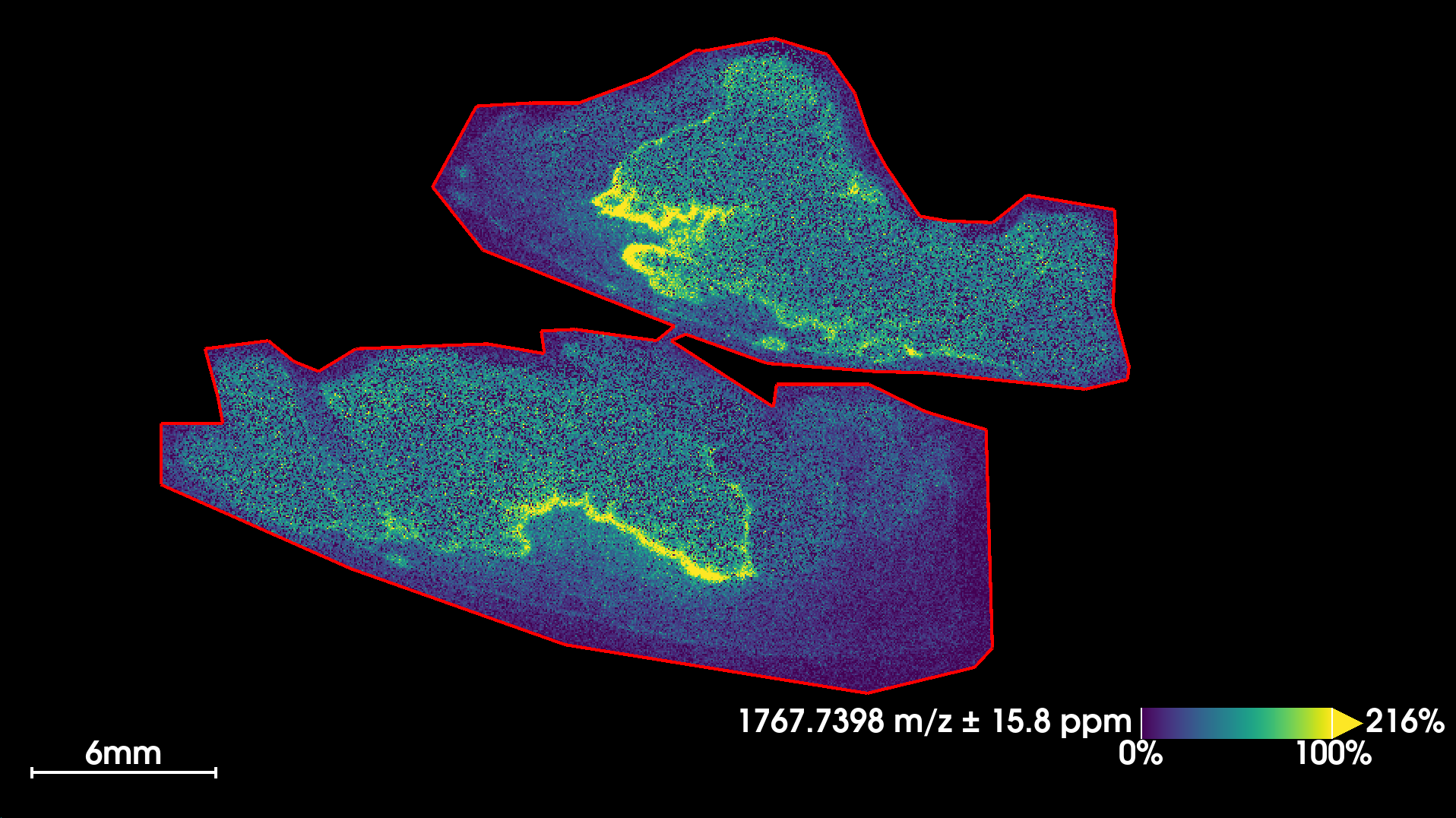

Supplement: Supplementary file 8 — Source Data 2 [file 41467_2026_72853_MOESM8_ESM.zip › Source Data MALDI Images/Supplementary Figure 14/1767.7398 mz ┬▒ 28 mDa.png]

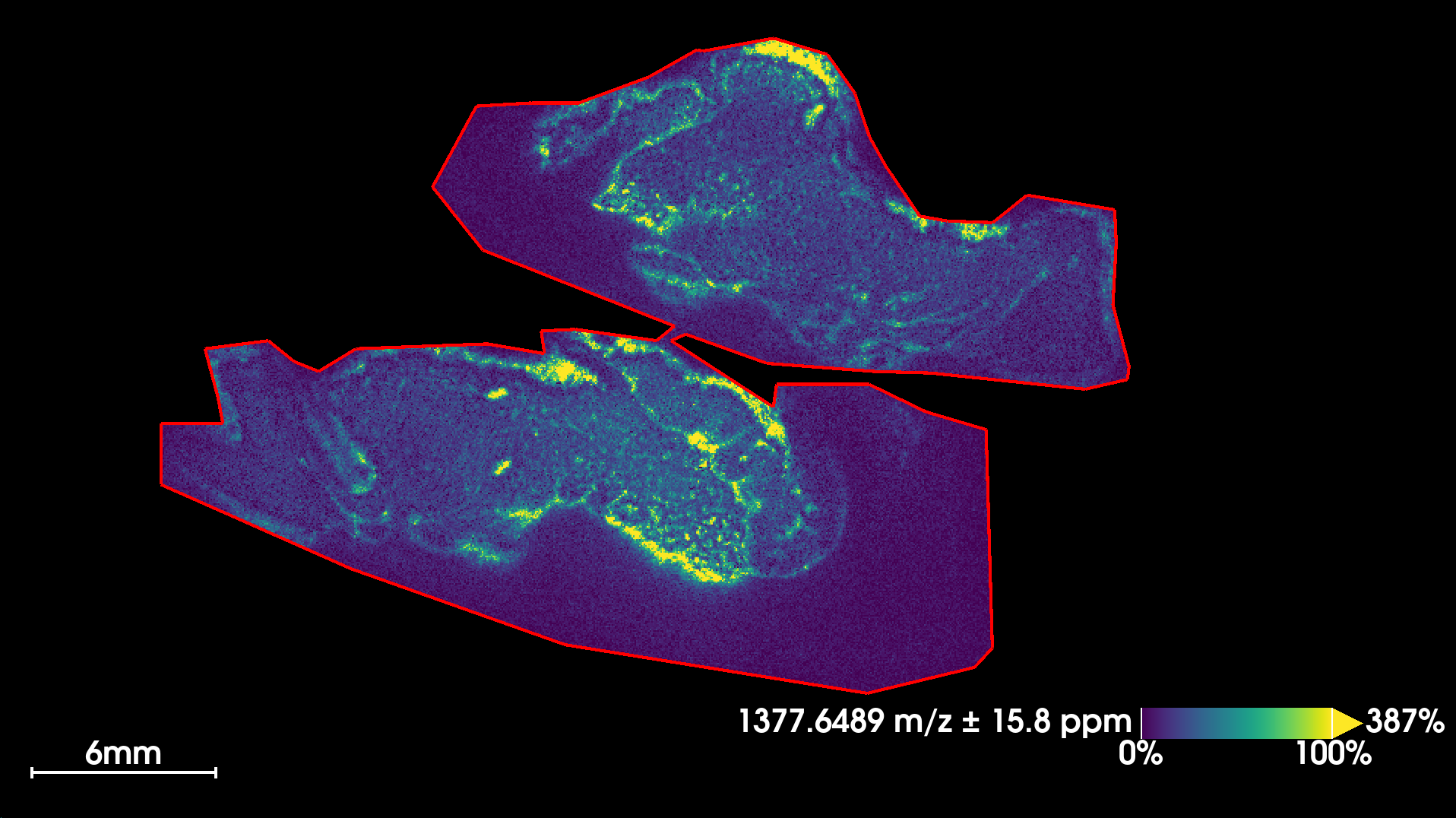

Supplement: Supplementary file 8 — Source Data 2 [file 41467_2026_72853_MOESM8_ESM.zip › Source Data MALDI Images/Supplementary Figure 14/1377.6489 mz ┬▒ 21.8 mDa.png]

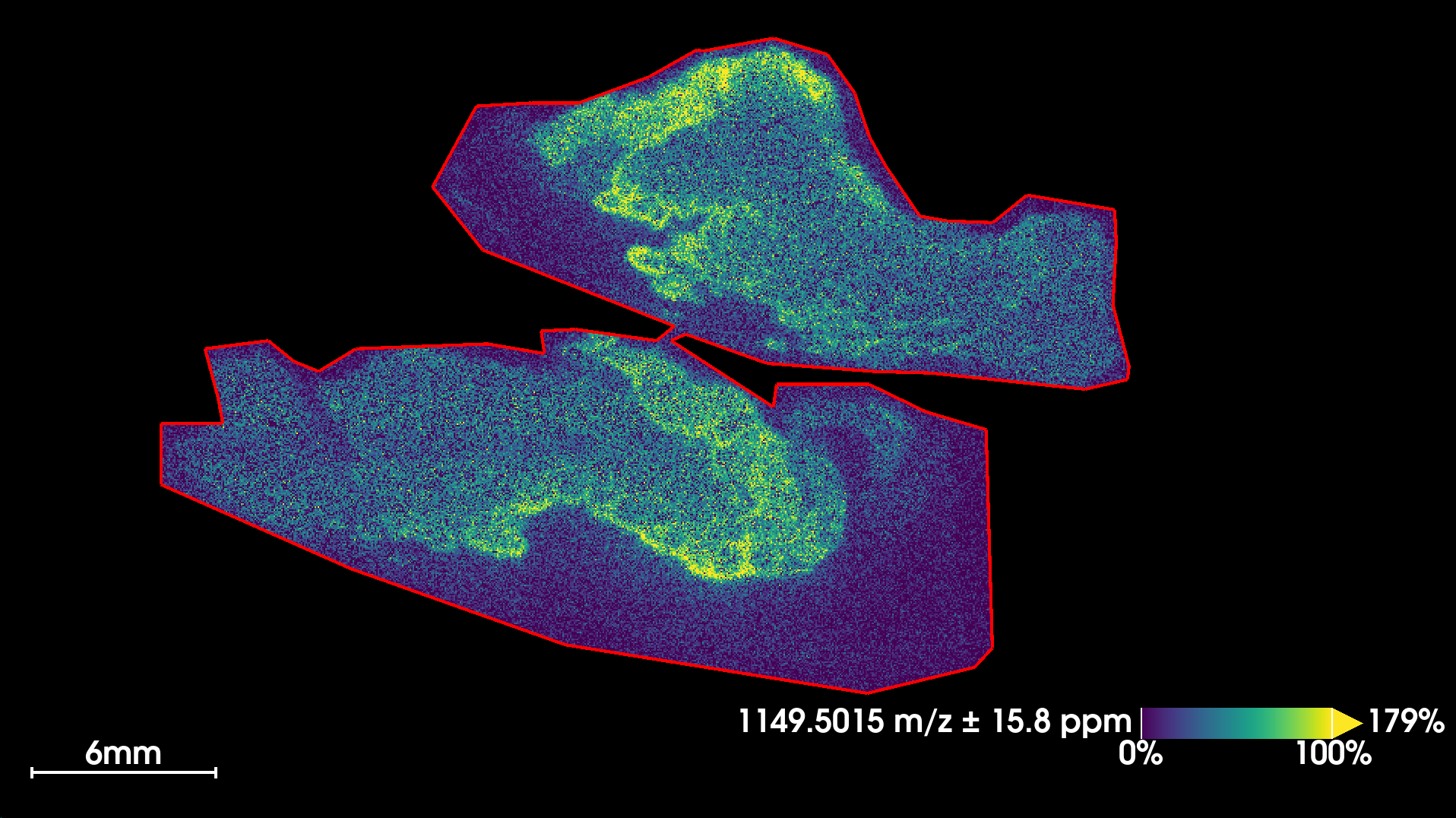

Supplement: Supplementary file 8 — Source Data 2 [file 41467_2026_72853_MOESM8_ESM.zip › Source Data MALDI Images/Supplementary Figure 14/1149.5015 mz ┬▒ 18.2 mDa.png]

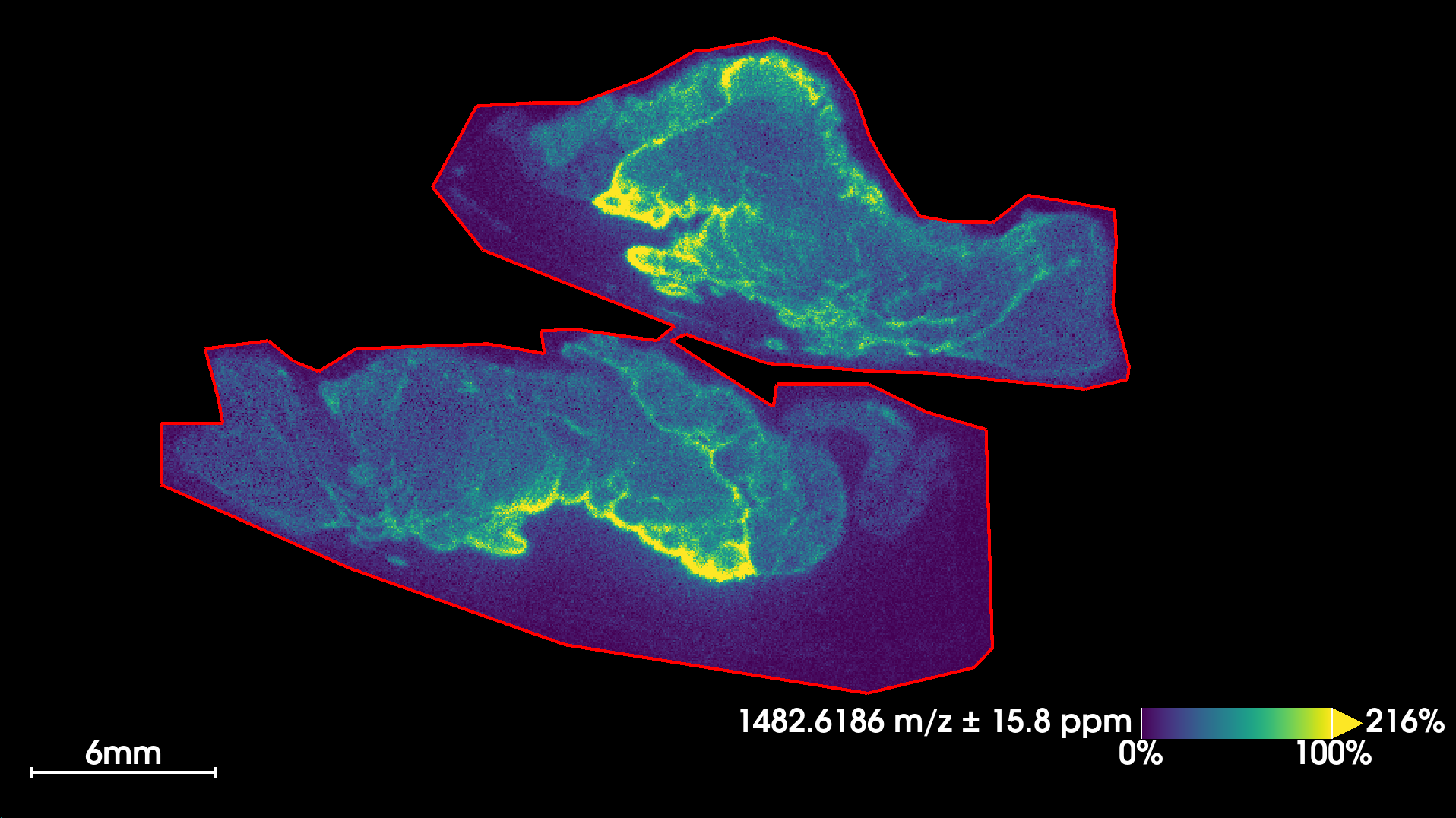

Supplement: Supplementary file 8 — Source Data 2 [file 41467_2026_72853_MOESM8_ESM.zip › Source Data MALDI Images/Supplementary Figure 14/1482.6186 mz ┬▒ 23.5 mDa.png]

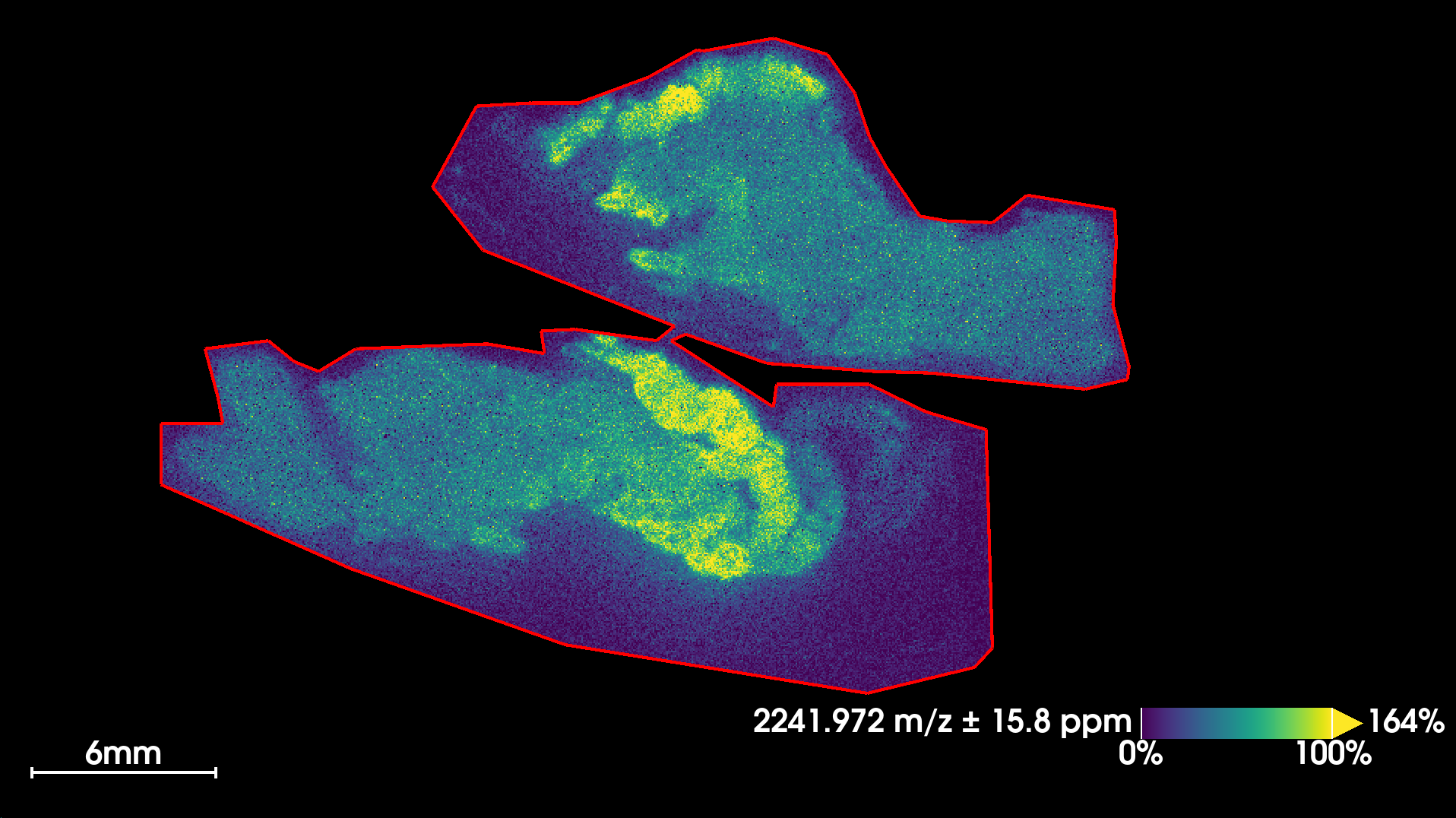

Supplement: Supplementary file 8 — Source Data 2 [file 41467_2026_72853_MOESM8_ESM.zip › Source Data MALDI Images/Supplementary Figure 14/2241.972 mz ┬▒ 35.5 mDa.png]
