# Supplementary material for: Glycosite mapping and in situ mass spectrometry imaging of MUC2 glycopeptides via on-slide mucinase digestion
Source: Nat Commun. 2026 May 7;17:6125. doi: 10.1038/s41467-026-72853-3 (PMC13357551; doi:10.1038/s41467-026-72853-3)
Supplement: Supplementary file 6 — Transparent Peer Review file [file 41467_2026_72853_MOESM6_ESM.pdf]

nature portfolio

Peer Review File

Glycosite Mapping and in situ Mass Spectrometry Imaging of MUC2 Glycopeptides via On-slide Mucinase Digestion

Corresponding Author: Professor Stacy Malaker

This file contains all reviewer reports in order by version, followed by all author rebuttals in order by version.

Version 0:

Reviewer comments:

Reviewer #1

(Remarks to the Author)
The manuscript by Malaker et al. elegantly demonstrates the potential of MALDI imaging to spatially visualize O-glycopeptides on histology sections. Combined with LC-MS analysis the approach can be used to identify the modified protein and residues observed by the MALDI imaging. The study focusses on a small sample set of mucinous tumors originating from different tissue types and proposes that these tumors produce large amounts of secreted mucins with truncated O-glycans. This finding provides the first in situ evidence of altered O-glycosylation in cancer tissue, with a specific focus on the MUC2 mucin. In addition, the authors were able to determine the density of O-glycosylation of the mucin domain of MUC2 and the potential first observation of acetylation of a GalNAc residue. Overall, the work is thorough and well-presented but lacks essential details/data. To strengthen their conclusions, the authors should incorporate more details on selected aspects of the manuscript as detailed below:

General comments:
- The authors draw conclusion based on a limited sample size (of one section per tissue) that would require more rigorous analysis to support, for example the conclusions based on the tissue protein identifications on page 11 claiming tumor specific proteins can't be made without replicates and quantification.
- The authors are in the assumption that the truncated O-glycans are due to the cancerous tissue origin. The length of O-glycans on MUC2 in the digestive tract is much longer (4 – 6) than that observed by the authors. Would it be possible that the observed signal is originated from intracellular not fully matured MUC2? In addition, StcE is limited in its ability to act on more complex/extended polysaccharides, could this also contribute to the observation. As there isn't much benign epithelial cells remaining in the tissue section so the lack of these O-glycopeptides isn't surprising as there will be little to no MUC2 expression. Ideally the authors would perform similar analysis on control intestinal tissue to establish the specific nature of these region with shortened O-glycans on MUC2.
- The authors state that they compare LC-MS O-glycopeptide analysis between tumor and non-tumor tissue, but the results aren't described or included in the manuscript. This data should be included to support the claims. Referring to lack of limited signal in the MALDI-MSI data is not sufficient.
- Some inconsistencies occur concerning the cleavage site preference of StcE as stated below as minor comments as well. Various none "T*X/T" peptides are identified or with glycan modifications at the P1 position. This variation might occur, and activity is likely more dependent on the presence of a O-glycan, but the authors also use the consensus motif as an argument for the absence of selected membrane mucins.
- Considering the presentation of the acetylated residues, please annotate spectra accordingly with observed mass of key/highlighted ions or provide fragment table. In addition, can the authors provide any insight on the relative abundance of these modifications, and potential biological implications. (Data is missing in the supplemental tables)
- Healthy human tissue expresses mainly core-3 glycans on MUC2 in the colon, while mainly core-1 structures were identified. Is this a common feature for mucinous tumors, or a limitation of StcE to act on core-3 modified structures.

Minor comments:
Lines 48 – 48: N-glycan extension mainly occurs in the Golgi
Lines 153 – 155: Colon tumor 1 section originates from the same patient as colon tumor 2 and MALDI-MSI data were obtained on a different instrument with different resolutions. Can the authors comment on variation in the patient and the impact of the resolution on the results.
Lines 176 – 178: Where is the data for the non-tumorous tissue

Lines 183 – 184: The statements made by the authors contributing to the increased search space are valid. Except for the non-specific enzymatic cleavage, as StcE in combination with trypsin would have a distinct preference n-term S/T and c-term K/R would this setting improve the results? In the method section none of the glycopeptides searches was performed using semi-specific settings, although each peptide should start with T/S + O-glycans. This would likely result in more false positive results. How do the authors justify their search strategy.
Lines 219 – 221: How do the authors envision that IMPa would improve this? Wouldn't be the main issue be the overwhelming amount of signal from the tryptic peptides suppressing the O-glycopeptide signal?
Lines 247 – 249: The initial StcE consensus sequence was established based on a limited set of glycosylated substrates. Based on the presented data this strong preference for T in the P2 is not as prevalent as claimed by the authors in previous work. Would the main reason for not identifying any glycopeptides for MUC1 be do the varying in abundance between transmembrane and secreted mucins.
Lines 253 – 254: The MUC12 peptide for example is T/SH or H/TT neither matching StcE consensus sequence (T/S)X/(T/S)
Lines 260 – 262: MUC5B isn't present in the supplementary data table 1 or 2. The total intensity of MUC2 and MUC5AC at protein level indicate a large variation in abundance. Is this a limitation in the sensitivity of the method.
Lines 269 - 278: It's unclear in supplementary table which tab contains the identifications matched to the MALDI-MSI data?
Lines 281 - 286: m/z ranges don't match with the method section.
Line: 309: Should refer to supplementary figure 13
Lines 316 – 320: Clarify that the 92% assumes that the peptides originating from each repeated sequence were identified or highlight shared peptides in the figure 3a.
Lines 326 - 327: Remove significantly.
Lines 352 – 353: Consider removing this statement, or move to discussion.
Lines 364 – 377: Data missing from supplementary table 2.
Lines 415 – 417: Based on the specified search settings how where these identified?
Lines 514 - 516: The images clearly show a difference in pixel resolution between colon 2 and the other tissues. Please correct statement or clarify.
Lines 637 – 642: Which data are collected using ITMS MS2? Provide details concerning FDR strategy and threshold.
Lines 659 – 661: Indicate which peptides were identified using this method and considered based on what criteria.

Supplementary table 2: Report probability score.

Reviewer #2

(Remarks to the Author)
In this study, the authors developed a workflow combining on-tissue mucinase StcE digestion with MALDI mass spectrometry imaging (MALDI-MSI) and LC-MS to spatially map mucin O-glycopeptides in tumor tissues. This approach revealed distinct MUC2 glycoform distributions within colorectal mucinous adenocarcinomas and achieved near-complete sequence coverage of MUC2 mucin domains. Additionally, the authors identified endogenous O-acetylated GalNAc, a novel glycan modification. Their findings demonstrate the potential of this technique to advance glycoproteomic studies in cancer and other fields. While technically advanced, the work is highly specialized and contains numerous areas where explanations are insufficient. Further development and clarification will be necessary for publication in a journal that also targets readers outside this specific field.

Major points:
1. In Figure 1, the intent behind showing N-glycan imaging should be clarified. Is the purpose simply to demonstrate that the N-glycan processing required for imaging O-glycopeptides worked effectively? If so, this should be clearly stated. This content might be more suitable in a supplementary figure.

1. Line 298-
It would be helpful for readers of Nature Communications if the Introduction included definitions of the T antigen and Tn antigen, as well as the fact that these are dominantly detected by mass spectrometry from glycoproteins. This background information would aid reader understanding.

3. In Figure 2, images of two peptides, TPSPPTT and TVTPTPTPTG, are shown. The authors only mention TPSPPTT, but is the statement intended to apply to both glycopeptides? If so, this should be clearly indicated.

4. Line 327
The statement that glycan structures were “more diverse” on peptide sequences from the second PTS domain (Fig. 3c) should be supported by quantitative evidence. Please consider including a measure of diversity, such as the number of distinct glycoforms or another appropriate metric.

5. Line 37-
Sialic acids are known to be susceptible to hydrolytic cleavage under acidic (low pH) conditions. Could the unexpectedly low sialic acid content in the other samples be due to acid-induced loss during sample preparation? In the salivary gland tumor sample, where sialic acid levels were extremely high, some may have remained detectable despite such degradation — but this may not be the case for the other samples.

6. Line 469-
The importance of sample size is clear, but the authors should also discuss the advantages of using fresh or unfixed samples over paraformaldehyde-fixed (PFF) tissues. Unfixed samples better preserve protein integrity and modifications, which can improve glycoproteomic analysis. Addressing this would strengthen the manuscript.

7. Line 498
If staining and coverslip removal were performed before MSI, this process should be illustrated in Figures 1A and 2A as well.

8. In this paper, “m/z” is written after the numerical values, but it should be placed before the numbers (e.g., m/z 300–3500).

9. Line 637-
It is unclear whether these settings (formaldehyde-related modifications such as lysine crosslinks and dehydration, and allowing more missed cleavages) were applied to the PFF samples. Please clarify.

10. Line 661
Could the authors clarify how they assigned peptides that are shared among multiple proteins to a single protein? Specifically, what criteria or rules were applied to resolve ambiguous peptide-to-protein mappings? Providing this information would improve the clarity and reliability of the protein assignments. Or were there no ambiguous peptide-to-protein mappings observed?

Other concerns:
11. Line 120- etc.
The authors use the term “high spatial resolution,” but this term is ambiguous. Instead, they should specify the actual resolution, for example “tens of micrometers resolution”.

12. Line 129
The use of "However" in the sentence seems somewhat inappropriate. The statement about analytes typically being identified only by their intact precursor mass in MALDI-MSI does not directly contrast or contradict the preceding hypothesis, but rather provides additional context or explanation.

13. A general comment applicable to all figures, including supplementary ones: according to international conventions, the symbol “m/z” should be consistently italicized throughout the manuscript.

14. Line 161-
“Nearly all ions detected in high abundance outside of the tumor boundaries were residual N-glycans,”

15. Line 309
Figure 13 is referred to, but it should be Supplementary Figure 13.

16. Line 372
The same asterisk annotation used in Fig. 4 should also be included in Supplementary Figure 22.

17. Line 434-
If the authors wish to emphasize that the number is “many,” the term is somewhat ambiguous. It would be helpful to provide an estimate or approximate cleaved patterns by non-expected enzymes to support this statement.

18. Line 484
It seems that “μM” was used to describe tissue thickness, which is likely a typo (μm).
In addition, information about the conductive slide glass for MALDI-MSI should be included here.

19. Line 656-
The authors should report the approximate number of ions manually searched for MSI based on the LC-MS results. Disclosing the workload involved in this approach is important for scientific publications aiming for reproducibility.

Reviewer #3

(Remarks to the Author)
This manuscript reports on proof-of-concept experiments carrying out spatial analysis of O-linked glycoproteins on mucinous carcinomas. The authors have looked at three different mucinous carcinomas. Being a proof-of-concept paper, the samples numbers are extremely low, 2 colorectal samples (from the same patient), one salivary and one oesophageal.
The decision to use mucinous carcinomas for the analysis O-linked glycans may have resulted in technical problems due to the large amount of secreted mucins. As mucins are upregulated in many non-mucinous carcinomas, including colorectal, these may have been a better choice.

There are a number of issues that elicit concern regarding the methodology:

• After treatment to remove N-linked glycans, the sections were treated with the mucinase, StcE. The step is vital to allow release of mucin O-linked glycopeptides but no analysis of the efficacy of the StcE was carried out and so the ability of this purified enzyme to cut at the specific mucin site after the tissue was treated in the way described, was not determined. Moreover, although MUC1 was detected in both colon samples, no MUC1 glycopeptides were identified. Each tandem repeat of MUC1 has 2 potential StcE. T*S and S*T. These sites are missing the intermediate amino acid as in S/T*-X-S/T and so as described by Malaker et al 2019 the cutting efficiency of StcE is reduced (* equal glycosylated aa for StcE cleavage). This may be an indication that the cutting efficiency of the StcE used in the experiments nor/or the condition of material is not optimal, as previous studies have shown that StcE can cut MUC1.
• No MUC1, was not seen in the salivary or oesophageal tumour or normal which is unusual.
• Mucinous colorectal carcinomas have been shown previously to express MUC2 and 94% to express MUC5AC (see Walsh et al 2013, Modern Path, 26,1642) but no MU5AC was found in the colorectal samples.
• The O-linked glycans found in the colon carcinoma samples were mainly of GalNAc (Tn) and GalNAc-Gal (T). Very little sialylated glycans or other glycan cores were found. This profile is definitely unusual as sialylated glycans are known to be present, as are extended cores. Given these results and the lack of testing of the StcE enzyme, the authors should use the same technique on cell line pellets and compare these with their previous datasets.
• The oesophageal tumours had a very similar profile to the colon tumour, although the salivary tumour had more sialic acid containing glycans. The small numbers of sialylated glycansin the colon samples is very odd. Very little has been published on the O-glycans of salivary tumours.
• The lack of tissue for any kind of validation is of concern. Perhaps it would have been sensible to make sure samples were used where there was sufficient tissue for validation.
• The symbols in Figure 2 need sorting out.

The results of the glycan analysis and mapping is very dependent on the specificity and efficacy of StcE. Therefore, this must be taken into account when drawing conclusions. The small number of tumours of course causes concern over how representative the results are. However, it is recognised that setting up this technology is not easy, and the authors should be commended for this.

This manuscript was co-review with an early career researcher

Reviewer #4

(Remarks to the Author)
I co-reviewed this manuscript with one of the reviewers who provided the listed reports. This is part of the Nature Communications initiative to facilitate training in peer review and to provide appropriate recognition for Early Career Researchers who co-review manuscripts.

Version 1:

Reviewer comments:

Reviewer #1

(Remarks to the Author)
Overall, the authors have successfully addressed my previous questions and revised the manuscript accordingly. I am satisfied with these changes. I have just one final comment regarding the current version: the newly included data for the additional tumor and healthy tissue samples have not been deposited to PRIDE, which is essential for publication.

Reviewer #2

(Remarks to the Author)
The authors have thoroughly addressed Reviewer #2’s concerns, particularly by improving clarity for a broader readership, refining figure presentation, and explicitly defining analytical assumptions. The revised manuscript is substantially strengthened and now well-aligned with the scope and audience of Nature Communications.

Reviewer #3

(Remarks to the Author)
I am delighted that the authors were able to include two additional samples; as they say this further highlights the utility of this approach.

My concern regarding mucinous carcinomas is simply that spatial analyses (ie IHC) with these tumours can be challenging (with respect to tumour cell specificity) owing to the extracellular pooling of mucous. However I understand the logic in wanting samples containing an abundance of mucous to enable the testing of the methods, the emphasis of the paper.

The suggestion with the StcE is not that I am unaware of StcE or unaware of the association of the author with this enzyme, it is simply that in my experience it can be pertinent to ensure quality of each batch of agent when the agent is critical for the work, especially when using a new method. For example QC-ing new methods on cell pellets where known glycoproteins

are expressed would a good control - and perhaps something to consider for the future.

Thank you for the very clear explanation regarding MUC1, MUC2 and MUC5AC expression, and also the excellent defence of the lack of sialylation owing to technicalities.

Reviewer #4

(Remarks to the Author)
I co-reviewed this manuscript with one of the reviewers who provided the listed reports. This is part of the Nature Communications initiative to facilitate training in peer review and to provide appropriate recognition for Early Career Researchers who co-review manuscripts.

Open Access This Peer Review File is licensed under a Creative Commons Attribution 4.0 International License, which permits use, sharing, adaptation, distribution and reproduction in any medium or format, as long as you give appropriate credit to the original author(s) and the source, provide a link to the Creative Commons license, and indicate if changes were made.
In cases where reviewers are anonymous, credit should be given to 'Anonymous Referee' and the source.
The images or other third party material in this Peer Review File are included in the article’s Creative Commons license, unless indicated otherwise in a credit line to the material. If material is not included in the article’s Creative Commons license and your intended use is not permitted by statutory regulation or exceeds the permitted use, you will need to obtain permission directly from the copyright holder.
To view a copy of this license, visit https://creativecommons.org/licenses/by/4.0/

REVIEWER COMMENTS

Reviewer #1 (Remarks to the Author):
The manuscript by Malaker et al. elegantly demonstrates the potential of MALDI imaging to spatially visualize O-glycopeptides on histology sections. Combined with LC-MS analysis the approach can be used to identify the modified protein and residues observed by the MALDI imaging. The study focusses on a small sample set of mucinous tumors originating from different tissue types and proposes that these tumors produce large amounts of secreted mucins with truncated O-glycans. This finding provides the first in situ evidence of altered O-glycosylation in cancer tissue, with a specific focus on the MUC2 mucin. In addition, the authors were able to determine the density of O-glycosylation of the mucin domain of MUC2 and the potential first observation of acetylation of a GalNAc residue. Overall, the work is thorough and well-presented but lacks essential details/data.

We thank the reviewer for their kind comments and hope that our revisions adequately address their concerns. Our revised manuscript includes data for two additional samples: a third colorectal carcinoma (different donor) and a section of healthy colon tissue. For clarity, we have updated the naming to Colons 1a & 1b (representing the original two colon tumors resected from the same patient) and Colon 2 (new data).

To strengthen their conclusions, the authors should incorporate more details on selected aspects of the manuscript as detailed below:

General comments:
- The authors draw conclusion based on a limited sample size (of one section per tissue) that would require more rigorous analysis to support, for example the conclusions based on the tissue protein identifications on page 11 claiming tumor specific proteins can't be made without replicates and quantification.

We agree with the reviewer that additional samples and more rigorous quantification are needed to support this statement. We have removed Supplementary Table 1 listing unmodified proteins shared between samples and the following text from the manuscript:

"**Using the unmodified peptide data, we identified 7 proteins that were exclusively identified in the tumor regions of each tissue (Supplementary Table 1). Previous studies have linked aberrant expression of many of these proteins to malignant processes. For example, upregulation of acid ceramidase (Q13510) has been observed in a variety of different cancer types, and in some instances, drug-induced downregulation of this protein was shown to enhance the efficacy of chemotherapy *in vitro* and in mouse models.[45] Another protein in this category, latent-transforming growth factor beta-binding protein 2 (LTBP2, Q14767), interacts with various ECM proteins in elastic fiber-rich tissues,[46] and its expression was previously linked to worse patient outcomes in head and neck squamous cell carcinoma.[47] Many more samples must be analyzed before making any biological conclusions, but the identification of cancer-associated proteins with the expected expression patterns demonstrates the potential depth of information provided with our workflow.**"

Additionally, to ensure the robustness of our technique, we added additional tumor and healthy tissue slices to the manuscript. We believe this bolsters our proof-of-concept work but agree that many more samples would be required for translational applications or statements about tumor specificity.

- The authors are in the assumption that the truncated O-glycans are due to the cancerous tissue origin. The length of O-glycans on MUC2 in the digestive tract is much longer (4 – 6) than that observed by the authors. Would it be possible that the observed signal is originated from intracellular not fully matured MUC2?

We agree with the reviewer that we cannot fully exclude the possibility of an intracellular origin for the MUC2 and have included the following in the revised manuscript:

"**Because data for Colon 2 and the Healthy Colon were acquired on the same instrument, we compared the relative abundance of MUC2 glycoforms in these tissues by normalizing the MS signal. Although MUC2 glycopeptides with truncated glycosylation were detected in the Healthy Colon, signal for these**

ions was negligible when directly compared to their intensity in Colon 2 (Fig. 2d). This disparity suggests that truncated, non-sialylated O-glycoforms of MUC2 are overexpressed in the colorectal tumors compared to healthy tissue. In the future, integrating our results into a multi-omic analyses of an expanded patient cohort could potentially link specific, overexpressed MUC2 O-glycoforms involved in carcinogenic processes in mucinous colorectal tumors. O-glycopeptide epitopes might then serve as diagnostic or prognostic biomarkers and possibly therapeutic targets. However, we should note that we cannot fully rule out the possibility that a portion of the detected signal corresponds to immature, Golgi-associated MUC2."

As mentioned in the text, we believe that a cancerous origin is more likely than immature glycans due to the well-documented relationship between O-glycan truncation and cancer (Stowell SR *et al. Annual Review Pathology: Mechanisms Disease* **10**, 473-510 (2015)). The relative paucity of truncated O-glycoforms in the Healthy Colon tissue further supports this notion. Figure 2d in the revised manuscript shows the drastic differences in expression of truncated MUC2 O-glycoforms:

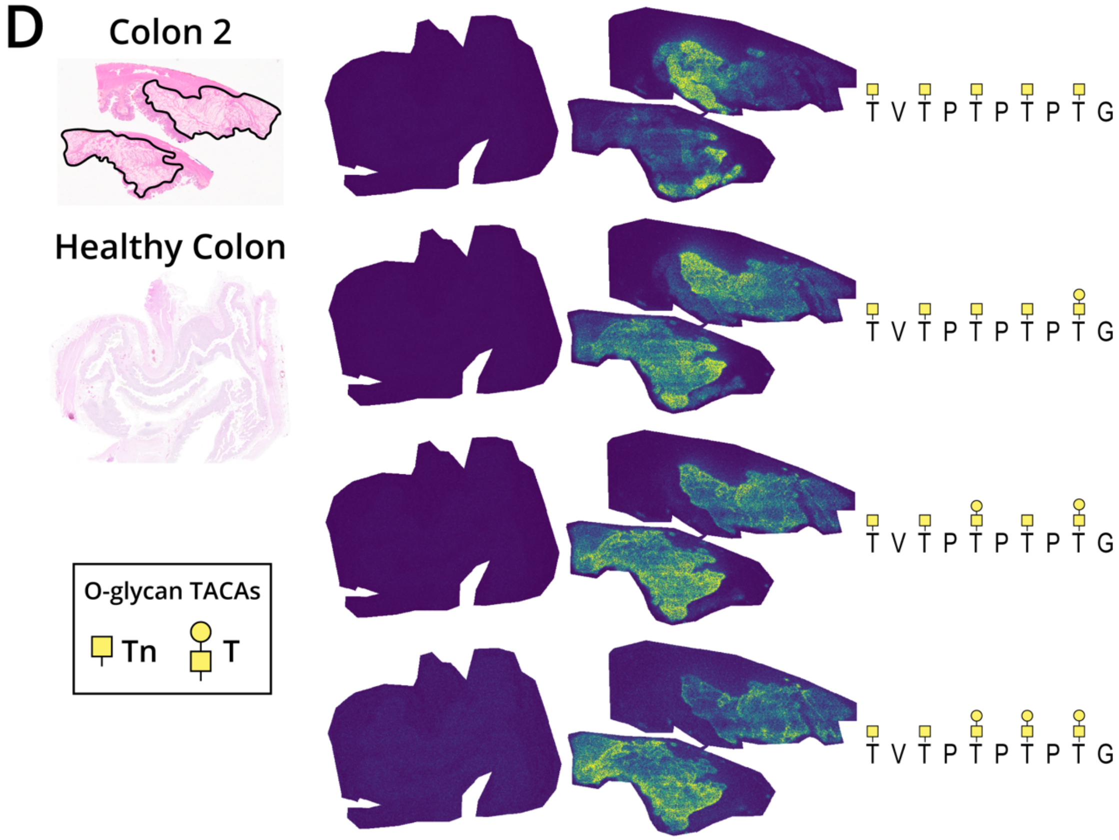

In addition, StcE is limited in its ability to act on more complex/extended polysaccharides, could this also contribute to the observation.

We concede that other work has found that StcE does not efficiently cleave sialyl-Tn (STn) nor core 3 O-glycan structures (Nason, R., *et al. Nat Commun* **12**, 4070 (2021)). However, we respectfully disagree with the reviewer's suggestion that StcE has only limited activity on complex/extended glycans more generally. In the same previously referenced work, Nason et al. also found that StcE efficiently digested mucin reporter sequences decorated with core 2 structures, consistent with results documented in the original publication detailing StcE's preferred cleavage motif (Malaker SA, *et al. Proc Natl Acad Sci USA* 116(15), 7278-7287 (2019)).

Abundant dipeptides decorated with extended polysaccharides were detected in the esophageal tissue section as well as in the non-cancerous Healthy Colon tissue, which we have detailed in the revised manuscript:

“By manually extracting and analyzing spectra with intense HexNAc fingerprint ions, we identified multiple dipeptides with the sequence TI/TL or IT/LT. These dipeptides were highly abundant and decorated with a variety of structures, including H2N2F1 and H2N3F2 (Supplementary Figs. 33a-b).”

“O-glycopeptides decorated with di- and tri-acetylated Neu5Ac on more extended core structures were highly abundant in the Healthy Colon tissue (Supplementary Figs. 21, 22f, & 25). Previous work characterizing human MUC2-derived O-glycans determined that many glycans with the same composition (minus Neu5Ac O-acetylation) were core 3 (Supplementary Fig. 21).[60] On the other hand, assays using StcE to digest mucin tandem repeat reporters with defined O-glycans found that core 3 structures effectively blocked proteolysis.[56] However, efficient cleavage of core 3-decorated mucin reporters was achieved at higher concentrations (approximately 1 μg/mL) in this same study.[56] Given that we saturated each tissue surface with much more concentrated StcE (approximately 50 μg/mL), it seems likely that any core 3-decorated mucins would be cleaved under these conditions. Future work should integrate in-depth glycomic analyses on the same samples for confident structural assignments.”

As there isn’t much benign epithelial cells remaining in the tissue section so the lack of these O-glycopeptides isn’t surprising as there will be little to no MUC2 expression. Ideally the authors would perform similar analysis on control intestinal tissue to establish the specific nature of these region with shortened O-glycans on MUC2.

As discussed above, we have now integrated additional data derived from non-cancerous colon tissue in our revised manuscript. Our MALDI-MSI data show that MUC2 glycoforms with truncated O-glycans are present in non-cancerous tissue, but at significantly lower relative levels with diffuse expression across the tissue. In fact, when normalized to the intensity of Colon 2, signal in the Healthy Colon is nearly imperceptible (see Figure 2d attached in response to a comment above). This is in contrast to all three colorectal tumor tissues, in which we observe abundant Tn- and T-decorated MUC2 with stratified localization patterns. Figure 2b-d in the revised manuscript shows these patterns for the backbone sequence TVTPTPTPTG. Figure 2d is shown above, and Figures 2b and 2c are shown here:

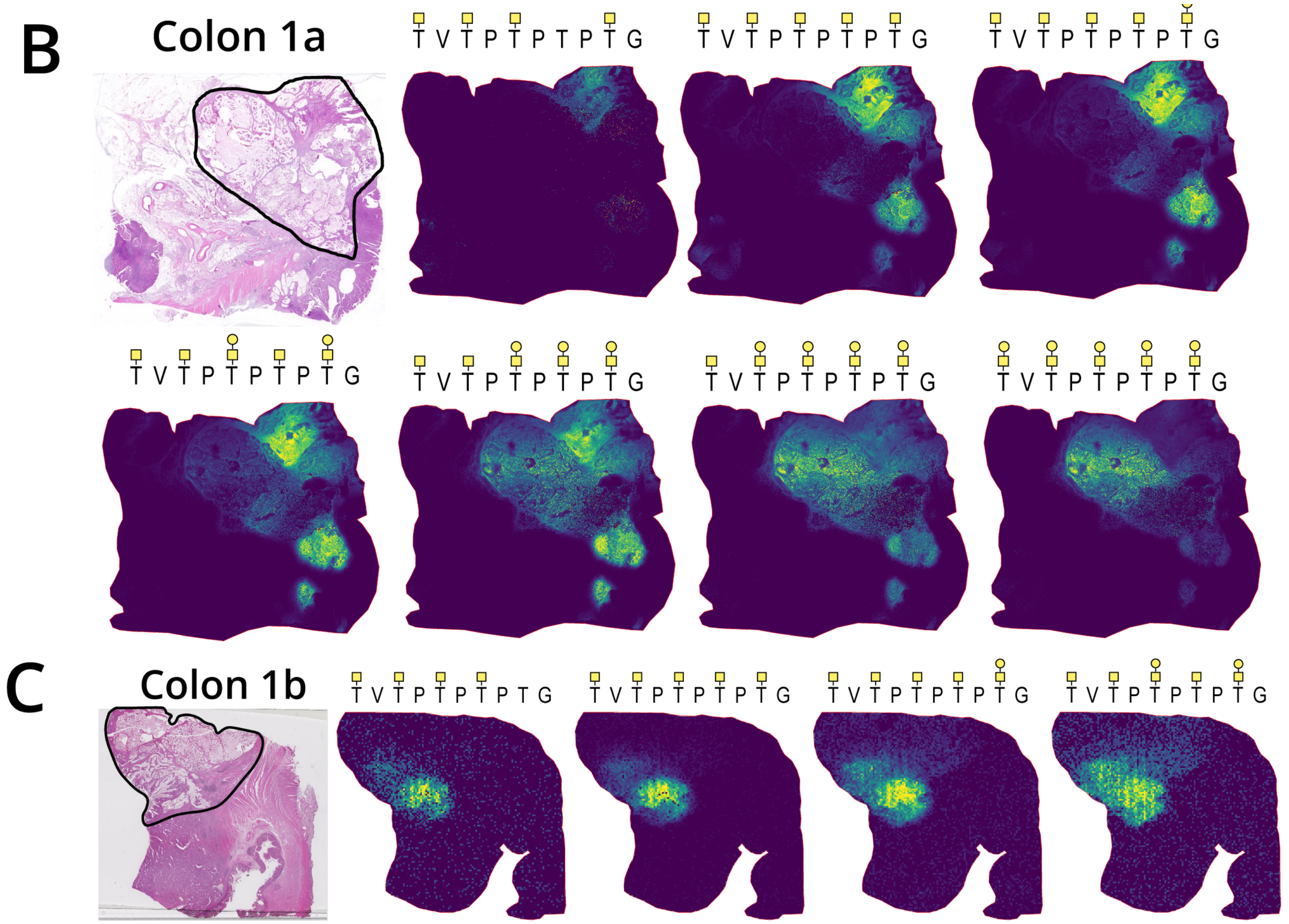

To accommodate the normalized images for Colon 2 and the Healthy Colon, we moved the images for the backbone sequence TPSPPTT to the supplement, as this sequence displays the same glycoform-dependent localization as for TVTPTPTPTG. Our revised figure also includes a small legend labeling the Tn and T antigens.

- The authors state that they compare LC-MS O-glycopeptide analysis between tumor and non-tumor tissue, but the results aren’t described or included in the manuscript. This data should be included to support the claims. Referring to lack of limited signal in the MALDI-MSI data is not sufficient.

We apologize for the confusion. “Non-tumor” tissue originally referred to the regions within the same section that are outside of the defined tumor boundaries (detailed in Supplementary Figures 1-6, 8). We updated the previous text to instead use the term “tumor-adjacent” tissue for clarity. As stated in response to the previous question, our revisions to the manuscript include integration of data from healthy colon tissue for comparison to tumor sections.

- Some inconsistencies occur concerning the cleavage site preference of StcE as stated below as minor comments as well. Various none “T*X/T” peptides are identified or with glycan modifications at the P1 position. This variation might occur, and activity is likely more dependent on the presence of a O-glycan, but the authors also use the consensus motif as an argument for the absence of selected membrane mucins.

We thank the reviewer for their thoroughness and raising an important point. Although StcE prefers to cleave within the “T*X/T” motif, it can cleave between adjacent Ser/Thr residues in the absence of its typical motif as

long as the P1 Ser/Thr is glycosylated. This behavior is described in Supplementary Figure S7 of Malaker *et al.* *PNAS* 2019, which describes the proteolytic activity of StcE in much greater detail. We would like to emphasize that non-canonical StcE cleavage is possible but not frequent.

It's also possible that these non-canonical termini represent proteolytic cleavage by endogenous proteases in each tissue. Non-specific cleavage was especially prominent for the proteoglycan versican in our glycoproteomic analysis of the salivary gland tumor, which we discussed in the main text:

"**Interestingly, many versican peptide sequences corresponded to non-StcE proteolytic cleavage at the N-terminus (n = 25, 9.8%), C-terminus (n = 110, 43.3%), or both (n = 9, 3.5%) (Supplementary Data 2). We detected only low levels of non-specific cleavage in other samples, suggesting degradation of versican by endogenous enzymes prior to tissue preservation.[1] Unmodified peptide searches identified multiple proteases and peptidases in this tissue (Supplementary Table 3). Whether O-glycosylation influences versican proteolysis and tumor ECM remodeling should be explored in greater detail.**"

- Considering the presentation of the acetylated residues, please annotate spectra accordingly with observed mass of key/highlighted ions or provide fragment table. In addition, can the authors provide any insight on the relative abundance of these modifications, and potential biological implications. (Data is missing in the supplemental tables)

We respectfully direct the reviewer to Figure 4 in the main text and supplementary figures S29 & S30 for annotated MS2 spectra of glycopeptides containing acetylated glycans. We have also added Supplementary Tables 1 and 2 listing the theoretical and observed glycan fingerprint ion masses along with calculated error for each, reproduced here:

Supplementary Table 1. Theoretical monoisotopic masses and subsequently detected exact masses of glycan fingerprint ions following higher energy collisional dissociation (HCD) of the sequence TPITT + N3Ac$_2$A1 shown in Figure 4a and Supplementary Figs. 29a-b.

Glycan Composition	Chemical Formula	Calculated Monoisotopic Mass	Detected Exact Mass	Error (ppm)
HexNAc-$C_2H_6O_3$	$[C_6H_8NO_2]^+$	126.0550	126.0543	-5.74
HexNAc-CH_6O_3	$[C_7H_8NO_2]^+$	138.0550	138.0544	-4.48
HexNAc-$C_2H_4O_2$	$[C_6H_{10}NO_3]^+$	144.0655	144.0652	-2.18
HexNAc-2H_2O	$[C_8H_{10}NO_3]^+$	168.0655	168.0647	-4.96
HexNAc-H_2O	$[C_8H_{12}NO_4]^+$	186.0761	186.0759	-1.25
HexNAc	$[C_8H_{14}NO_5]^+$	204.0867	204.0865	-0.74
Neu5Ac-4H_2O-Ac-CO	$[C_8H_8NO_2]^+$	150.0551	150.0534	-11.22
Neu5Ac-3H_2O-Ac-NH_3-CO	$[C_8H_7O_3]^+$	151.0392	151.0386	-4.38
Neu5Ac-4H_2O-Ac	$[C_9H_8NO_3]^+$	178.0500	178.0494	-3.61
Neu5Ac-3H_2O-Ac-NH_3	$[C_9H_7O_4]^+$	179.0341	179.0334	-3.84
Neu5Ac-3H_2O-Ac-CH_3	$[C_8H_7NO_4]^+$	182.0449	182.0444	-2.75
Neu5Ac-4H_2O-CO	$[C_{10}H_{10}NO_3]^+$	192.0657	192.0643	-7.13
Neu5Ac-3H_2O-Ac	$[C_9H_{10}NO_4]^+$	196.0605	196.0603	-1.34
Neu5Ac-4H_2O	$[C_{11}H_{10}NO_4]^+$	220.0606	220.0613	3.36
Neu5Ac-3H_2O	$[C_{11}H_{12}NO_5]^+$	238.0711	238.0704	-2.86
Neu5Ac-2H_2O	$[C_{11}H_{14}NO_6]^+$	256.0816	256.0798	-7.02
Neu5Ac-H_2O	$[C_{11}H_{16}NO_7]^+$	274.0921	Not Detected	n/a
Neu5Ac	$[C_{11}H_{18}NO_8]^+$	292.1027	Not Detected	n/a
AcNeu5Ac-3H_2O	$[C_{13}H_{14}NO_6]^+$	280.0818	280.0815	-0.79
AcNeu5Ac-2H_2O	$[C_{13}H_{16}NO_7]^+$	298.0923	298.0914	-3.00

AcNeu5Ac-H_2O	$[C_{13}H_{18}NO_8]^+$	316.1028	316.1023	-1.49
AcNeu5Ac	$[C_{13}H_{20}NO_9]^+$	334.1133	Not Detected	n/a
Ac_2Neu5Ac-2H_2O	$[C_{15}H_{18}NO_8]^+$	340.1028	340.1055	7.76
Ac_2Neu5Ac-H_2O	$[C_{15}H_{20}NO_9]^+$	358.1133	358.1131	-0.73
Ac_2Neu5Ac	$[C_{15}H_{22}NO_{10}]^+$	376.1238	376.1210	-7.60

Supplementary Table 2. Theoretical monoisotopic masses and subsequently detected exact masses of glycan fingerprint ions following higher energy collisional dissociation (HCD) of the sequence TLTPITT + N3AcN1 shown in Figure 4b and Supplementary Fig. 30a-b.

Glycan Composition	Chemical Formula	Calculated Monoisotopic Mass	Detected Exact Mass	Error (ppm)
HexNAc-$C_2H_6O_3$	$[C_6H_8NO_2]^+$	126.0550	126.0545	-4.36
HexNAc-CH_6O_3	$[C_7H_8NO_2]^+$	138.0550	138.0545	-3.37
HexNAc-$C_2H_4O_2$	$[C_6H_{10}NO_3]^+$	144.0655	144.0651	-2.60
HexNAc-2H_2O	$[C_8H_{10}NO_3]^+$	168.0655	168.0652	-1.60
HexNAc-H_2O	$[C_8H_{12}NO_4]^+$	186.0761	186.0761	-0.19
HexNAc	$[C_8H_{14}NO_5]^+$	204.0867	204.0868	0.30
AcHexNAc-H_2O	$[C_{10}H_{14}NO_5]^+$	228.0868	228.0883	6.61
AcHexNAc	$[C_{10}H_{16}NO_6]^+$	246.0973	246.0975	0.97

In Supplemental Data 2, we added MS1 peak areas for all listed precursors to facilitate quantitative comparisons. Compared to the Healthy Colon tissue, in which Neu5Ac was most often tri-acetylated, all colorectal tumor samples featured only mono- and di-acetylated Neu5Ac. This is accompanied by the following text:

"**O-glycopeptides decorated with di- and tri-acetylated Neu5Ac on more extended core structures were highly abundant in the Healthy Colon tissue (Supplementary Figs. 21, 22f, & 25)...**

On the other hand, LC-MS analyses of Colons 1a/b and 2 revealed only low levels of mono- and di-O-acetylated Neu5Ac-containing glycans, consistent with downregulation of this modification with disease progression (Supplementary Figs. 26 and 27). In Colons 1a and 1b, such structures were identified in tumor and tumor-adjacent regions, while the same glycans were only detected in the tumor-adjacent tissue in Colon 2. The Ac_2Neu5Ac fingerprint ion signal (*m/z* 358.1131 and 376.1237) exceeded that of AcNeu5Ac (*m/z* 316.1026 and 334.1132) across Colon 1b and in the tumor-adjacent region of Colon 1a, with the opposite pattern observed in the tumor region (Supplementary Figs. 26, 27, and 28). Glycans containing AcNeu5Ac and Ac_2Neu5Ac could be localized on peptides from the second PTS domain of MUC2 (Fig. 4a, Supplementary Figs. 29a-b, Supplementary Table 1, and Supplementary Data 2)."

This observation is consistent with published literature linking loss of sialic acid acetylation to the development and progression of colorectal tumors (see Corfield AP, et al. *Glycoconjugate Journal* **16**, 307–317 (1999)).

- Healthy human tissue expresses mainly core-3 glycans on MUC2 in the colon, while mainly core-1 structures were identified. Is this a common feature for mucinous tumors, or a limitation of StcE to act on core-3 modified structures.

We thank the reviewer for raising an important point. StcE can accommodate core 3 structures in its cleavage motif, albeit to a much lesser degree than other structures, as demonstrated in Figure 5 in Nason R. et al. *Nat Commun* 2021. Notably, this work found that sequences from both PTS domains of MUC2 showed relatively high levels of core 1 O-glycans compared to reporters derived from other canonical mucins. Furthermore, the new data include O-glycopeptides decorated with putative core 3 structures, which were detected in high

abundance in the Healthy Colon tissue but not in the colorectal tumor sections (Supplementary Data 2), a result that we have discussed in the revised manuscript (also reprinted in response to a comment above):

"**O-glycopeptides decorated with di- and tri-acetylated Neu5Ac on more extended core structures were highly abundant in the Healthy Colon tissue (Supplementary Figs. 21, 22f, & 25). Previous work characterizing human MUC2-derived O-glycans determined that many glycans with the same composition (minus Neu5Ac O-acetylation) were core 3 (Supplementary Fig. 21).[60] On the other hand, assays using StcE to digest mucin tandem repeat reporters with defined O-glycans found that core 3 structures effectively blocked proteolysis.[56] However, efficient cleavage of core 3-decorated mucin reporters was achieved at higher concentrations (approximately 1 μg/mL) in this same study.[56] Given that we saturated each tissue surface with much more concentrated StcE (approximately 50 μg/mL), it seems likely that any core 3-decorated mucins would be cleaved under these conditions. Future work should integrate in-depth glycomic analyses on the same samples for confident structural assignments.**"

Taken together, the data supports the overexpression of T/Tn structures as a feature of these mucinous tumors rather than a result of limited StcE cleavage. Very little has been published on changes in the glycome in mucinous colorectal adenocarcinoma specifically, so it's unclear if our data is representative of tumors in this category.

Minor comments:

Lines 48 – 48: N-glycan extension mainly occurs in the Golgi

We thank the reviewer for their comment and have fixed this in the text.

Lines 153 – 155: Colon tumor 1 section originates from the same patient as colon tumor 2 and MALDI-MSI data were obtained on a different instrument with different resolutions. Can the authors comment on variation in the patient and the impact of the resolution on the results.

We appreciate the reviewer pointing out an important feature of our MALDI-MSI data. Our revised manuscript includes two additional samples: a colorectal tumor and a healthy colon section from two different donors. The data for Colon 2 & the Healthy Colon were collected on the same instrument. Because different instrument models were used to collect MALDI-MSI data from different samples, we have refrained from directly comparing ion intensities between tissues. Rather, we have chosen to focus on the relative abundance and spatial distribution of ions within each sample. In our opinion, reproducing the same glycoform-specific distribution of MUC2 glycopeptides in different colorectal tumors, using three different instruments, in two separate labs, underscores the adaptability of our approach to a variety of MALDI-MSI platforms with variable resolution. We added the following language to the discussion:

"**MALDI-MSI data was acquired in two different labs on three different instruments with varied resolution, demonstrating the robustness of this technique for imaging intact O-glycopeptides.**"

We would like to note that all LC-MS/MS data was collected using the same instrument. While the large overlap in glycopeptides identified in Colons 1a & 1b is not surprising, we did not expect such a high degree of similarity with Colon 2 (new data), which was resected from a different donor. Our revised manuscript details our findings regarding the overlap between the colorectal tumor tissues:

"**Overall, we observed a high degree of overlap in the glycopeptides identified in Colons 1a, 1b, and 2. We expected Colons 1a and 1b to be similar in composition as they were resected from the same patient. However, we were surprised by their overlap with Colon 2, which was from a second patient. Nearly 90% of the glycopeptides identified in Colon 2 were shared with Colons 1a and 1b, a result largely driven by the overlap of MUC2 glycoforms, which composed nearly all (approximately 97.1%) of the reported identifications in Colon 2. Furthermore, Colon 2 was prepared by a different lab and analyzed at a much later date than the other colorectal tumors, underscoring the high similarity between these samples as well as the robustness of our workflow.**"

Lines 176 – 178: Where is the data for the non-tumorous tissue

We thank the reviewer for their comment and have updated the phrasing used for clarity:

"**Tumor and tumor-adjacent regions within the same tissue section were then outlined using a hydrophobic barrier pen, enabling a comparison of the (glyco)proteome between malignant and neighboring tissues.**"

Lines 183 – 184: The statements made by the authors contributing to the increased search space are valid. Except for the non-specific enzymatic cleavage, as StcE in combination with trypsin would have a distinct preference n-term S/T and c-term K/R would this setting improve the results? In the method section none of the glycopeptides searches was performed using semi-specific settings, although each peptide should start with T/S + O-glycans. This would likely result in more false positive results. How do the authors justify their search strategy.

We appreciate the reviewer raising important questions related to the poor performance of existing proteomic software for the analysis of mucinous O-glycopeptides. In theory, searching data with semi-specific settings should prove optimal for samples treated with both StcE and trypsin. However, empirically, we found that non-specific settings outperformed semi-specific searches. Many mucin domains, including those of MUC2, have very few Arg or Lys residues. As a result, the majority of MUC2 O-glycopeptides derived from StcE cleavage at both termini. Although StcE strongly prefers to cleave within the T*XT motif, previous work has shown that it can cleave at slightly different sites, as described above. We suspect that the dominance of StcE cleavage at both termini as well as its slightly permissive cleavage motif are the primary reasons why non-specific searches performed optimally. We would like to highlight the fact that the backbone sequence and glycan localization of all reported glycopeptides were manually verified, thereby eliminating the risk of false positives in our reported results.

Lines 219 – 221: How do the authors envision that IMPa would improve this? Wouldn't be the main issue be the overwhelming amount of signal from the tryptic peptides suppressing the O-glycopeptide signal?

Digestion with an O-glycoprotease like IMPa would ensure backbone cleavage near O-glycosites, likely enhancing the generation of glycopeptides that are small enough for ionization and detection via LC-MS/MS. As the reviewer notes, it is quite possible that even then, the signal from tryptic peptides may overwhelm that of the resultant O-glycopeptides if the latter are particularly low in abundance. In our experience, the PHEA-based enrichment described in this manuscript typically removes enough unmodified peptides to facilitate confident detection and identification of glycopeptides from complex samples.

Although this portion of the manuscript has been moved to the supplement during revisions, we have added the following text:

"**Cleavage proximal to O-glycosites by IMPa and similar enzymes could produce smaller glycopeptides more likely to be ionized in LC-MS, therefore improving their identification. However, it's possible that unmodified tryptic peptides could still suppress their detection during MS acquisition, a potential challenge that may or may not be alleviated via downstream enrichment techniques like the PHEA-based approached described in this work.**"

Lines 247 – 249: The initial StcE consensus sequence was established based on a limited set of glycosylated substrates. Based on the presented data this strong preference for T in the P2 is not as prevalent as claimed by the authors in previous work. Would the main reason for not identifying any glycopeptides for MUC1 be do the varying in abundance between transmembrane and secreted mucins.

We concede that the initial characterization of StcE's cleavage relied on a set of purified recombinant mucins, which does not necessarily capture the complexity of the tissue microenvironment. Please refer to our response to the next comment for discussion of StcE's cleavage motif. We would also like to point out that the number of identified glycopeptides with non-specific C-termini (i.e., residue other than Ser or Thr as second-to-last residue) was very small, with the exception of the salivary gland tumor in which nearly half of the glycopeptides in this tissue had non-specific C-termini (which we attribute to other protease activity). In all other tumor samples, less than 5% of all identified glycopeptides exhibited non-specific C-terminal cleavage, and approximately 12% of the glycopeptides fell into this category in the Healthy Colon tissue. Based on this information, we respectfully uphold that StcE has a strong preference for a glycosylated Ser or Thr in the P2 position. Notably, many of the non-specific C-termini glycopeptides across all tissues were derived from versican, suggesting an alternative explanation for this protein specifically. We suspect that these non-specific cleavage events are the result of endogenous protease activity prior to tissue preservation, which we have discussed in the main text:

"**Various cancers are associated with versican overexpression,[65] and cleavage of the core protein by tissue-resident and tumor-associated proteases may contribute to tissue invasion and metastasis.[66] Core 1 and core 2 structures with and without sialylation were localized on versican glycopeptides, although most were decorated with only T antigens (Supplementary Fig. 22e). Surprisingly, very few versican glycopeptides displayed the Tn antigen, in stark contrast to MUC2 results in the other tumors (Supplementary Figs. S22a-b). Interestingly, many versican peptide sequences corresponded to non-StcE proteolytic cleavage at the N-terminus (n = 25, 9.8%), C-terminus (n = 110, 43.3%), or both (n = 9, 3.5%) (Supplementary Data 2). We detected only low levels of non-specific cleavage in other samples, suggesting degradation of versican by endogenous enzymes prior to tissue preservation.[1] Unmodified peptide searches identified multiple proteases and peptidases in this tissue (Supplementary Table 3). Whether O-glycosylation influences versican proteolysis and tumor ECM remodeling should be explored in greater detail.**"

We believe that the lack of MUC1 glycopeptides in our results likely stems from its relative abundance compared to MUC2 in the colorectal cancer samples. This could be further compounded by the lack of canonical StcE motifs in the tandem repeats of MUC1, thereby restricting its cleavage to regions outside of the repeat domain. We have added a clarifying text to the revised manuscript:

"**Although MUC1 contains very few putative StcE cleavage sites in its tandem repeats, StcE-sensitive sequences can be found outside this region. Because there are only single copies of the potential glycopeptides produced by StcE, they are less likely to produce sufficient signal for detection in LC-MS. Therefore, we suspect the lack of MUC1 glycopeptides from Colons 1a and 1b stems from its relatively low abundance compared to MUC2 in these samples.**

Even though we were unable to detect MUC1 via LC-MS in Colon 2 and the Healthy Colon, immunohistological staining of MUC1 in these samples revealed weak expression in both tissues (Supplementary Figs. 32a-b). In contrast, staining with the catalytically inactive StcE^{E447D}, which binds any mucin domain-containing proteins in tissue, revealed an inverse expression pattern (Supplementary Figs. 32a-b). While MUC1 staining was strongest at the outer edges of the mucosa, StcE^{E447D} staining was detected throughout the mucosa and submucosa (Supplementary Figs. 32a-b). These data suggest StcE used to prepare LC-MS samples could not favorably bind to MUC1, in contrast to previous work demonstrating StcE's activity on MUC1.[39,61] This observation might be explained by unfavorable binding in a complex tissue or that our current antigen retrieval step does not adequately expose the MUC1 backbone for binding and cleavage by StcE."

Lines 253 – 254: The MUC12 peptide for example is T/SH or H/TT neither matching StcE consensus sequence (T/S)X/(T/S)

The glycopeptide in question has the backbone sequence SHSRPGSTH, with the glycan N1 localized to both Thr and the first Ser. In full-length MUC12, this sequence is both preceded and flanked by two Thr residues. If we assume the P2 Thr upstream of the N-terminus is glycosylated, both termini of the detected glycopeptide are consistent with StcE's preferred cleavage motif (glycosylation indicated by *, motif sequence bolded):

T*T/S*HSRPGS**T*H/T**T

In this case, the P1 Thr at the N-terminus and the C-terminal His are the “X” residues of the motif.

Lines 260 – 262: MUC5B isn’t present in the supplementary data table 1 or 2.

We thank the reviewer for identifying this oversight. A portion of the unmodified tryptic peptide data for the esophageal tumor sample was mistakenly removed when compiling results in Supplemental Data 1. We have corrected this error in the updated spreadsheet. MUC5B is not in Supplementary Table 2 (which details glycoproteomic results) since we did not identify any glycopeptides from this mucin, as noted in the main text:

“**We identified a single unmodified peptide from MUC5B, a secreted, gel-forming airway mucin. The backbone sequence of MUC5B contains numerous Ser and Thr within TXT motifs, so the relative paucity of MUC5B glycopeptides in the esophageal data was surprising. Like MUC2, this secreted mucin assembles into complex, higher-order structures that may render the protein backbone inaccessible to StcE**.”

The total intensity of MUC2 and MUC5AC at protein level indicate a large variation in abundance. Is this a limitation in the sensitivity of the method.

We believe that the variation in the relative intensity of MUC2 between samples more likely reflects size differences between each sample. Although the tumor regions of Colons 1a/b and 2 were roughly comparable in total area, the esophageal tumor region was much smaller, as noted in the text in the paragraph under the subheading LC-MS allows for detection of MUC2 and MUC5AC glycoforms in esophageal tumor:

“This tumor was notably smaller than those in the colorectal and salivary gland tissues, and as a result, the amount of recovered material and subsequent LC-MS HexNAc signal was lower in the esophageal samples compared to the others (Supplementary Fig. 11a).”

In the previous version of our manuscript, MUC5AC was only identified via unmodified tryptic peptides in the esophageal tumor, an expected result as this mucin is normally expressed in the respiratory and upper digestive tract. Excitingly, we detected multiple MUC5AC glycopeptides in the new Colon 2 data, consistent with reports of aberrant expression of this mucin in colorectal cancer (see Walsh MD et al. *Modern Pathology* 2013 and Betge J. et al. *Virchows Archiv* 2016) Our updated manuscript includes a description of these results:

“**MUC5AC, like MUC2, is a secreted, gel-forming mucin that is often aberrantly expressed in colorectal cancer. Normally, MUC5AC is only found in the respiratory and upper digestive tract, and its presence in colorectal cancer is associated with better patient outcomes.[24] We identified both unmodified tryptic peptides and glycopeptides from MUC5AC in Colon 2, but not Colons 1a or 1b. As with MUC2, the O-glycans we detected on MUC5AC glycopeptides were primarily truncated structures like the T and Tn antigens. Future work should investigate how MUC5AC glycosylation regulates its function and contribution to colon cancer.**”

Using these results from Colon 2, we were able to identify the same MUC5AC glycopeptides in the esophageal data. The relatively weak signal in this sample precluded their confident identification in our initial analysis.

Lines 269 - 278: It’s unclear in supplementary table which tab contains the identifications matched to the MALDI-MSI data?

We apologize for this oversight; our original supplemental tables did not include a separate tab for glycopeptides matched to MALDI-MSI data. The updated spreadsheets now include this information on the tab named “Matched to MALDI-MSI.”

Lines 281 - 286: m/z ranges don't match with the method section.

We thank the reviewer for their comment and have corrected the incorrect values listed in the methods section:

"The maximum *m/z* values used in our experiments were 4000 (Colon 1a), 2500 (Colon 1b), and 3000 (Colon 2, Healthy Colon, Esophagus & Salivary Gland)."

Line: 309: Should refer to supplementary figure 13

We thank the reviewer for their comment and have corrected the referenced figure number.

Lines 316 – 320: Clarify that the 92% assumes that the peptides originating from each repeated sequence were identified or highlight shared peptides in the figure 3a.

We thank the reviewer for their comment and have added clarifying language:

"**Because bottom-up proteomic methods cannot determine the exact origin of perfect repeat sequences from the same protein, our calculation assumes repeat glycopeptides were derived from all possible locations.**"

Lines 326 - 327: Remove significantly.

We thank the reviewer for their comment and have removed "significantly" from the text.

Lines 352 – 353: Consider removing this statement or move to discussion.

We thank the reviewer for their comment and have removed the following sentence:

"**Due to limited sample availability, we were unable to confirm this hypothesis by staining tissues with sialic acid-binding immunoglobulin-like lectins (Siglecs) or plant-derived lectin probes.**"

Lines 364 – 377: Data missing from supplementary table 2.

Glycopeptides containing acetylated Neu5Ac were included in the original version of supplemental table 2. However, the notation for different structures was inconsistent between tabs, which likely made them easy to miss in the spreadsheet. We apologize for the confusion and have updated the notation for acetylated structures to improve clarity and visibility in the revised supplemental file:

- Mono-acetylated Neu5Ac denoted as AcNeu5Ac
- Di-acetylated Neu5Ac denoted as Ac_2Neu5Ac
- Tri-acetylated Neu5Ac denoted as Ac_3Neu5Ac

Lines 415 – 417: Based on the specified search settings how where these identified?

We manually extracted spectra with intense HexNAc signal (i.e., m/z 204.0867) and determined their identity by hand. The main text has been edited for clarification:

"**Many glycopeptide species were too weak for MS2 selection and were identified by precursor mass and retention time (Supplementary Data 2). Despite strong HexNAc ion signal in the esophageal tumor**

(Supplementary Fig. 11a), relatively few spectra could be confidently assigned to glycopeptides from specific proteins using search algorithms. By manually extracting and analyzing spectra with intense HexNAc fingerprint ions, we identified multiple dipeptides with the sequence TI/TL or IT/LT."

Lines 514 - 516: The images clearly show a difference in pixel resolution between colon 2 and the other tissues. Please correct statement or clarify.

The reviewer is correct in noting the lower MALDI-MSI resolution of Colon 1b (formerly labeled Colon 2) with a pixel size of approximately 200 µm compared to tens of microns in images of other tissues. We believe this disparity highlights the sensitivity of our approach, which still discerned glycoform-specific expression patterns at a much lower resolution. Because of the long acquisition times required for ultra-high resolution MALDI-MSI analysis of entire tissue sections, rapid screening of tissues (the total MALDI-MSI acquisition time for Colon 1b was less than 1 minute) prior to full-length analyses can potentially increase the pace of future work using this approach.

We have added the following text to the manuscript:

"**Image resolution was in the range of 40-50 µm, except for Colon 1b, for which the pixel size was approximately 200 µm… The detection of distinct expression patterns in lower-resolution Colon 1b images highlights the sensitivity of our workflow, indicating tissues can be rapidly screened for such patterns in future work.**"

Lines 637 – 642: Which data are collected using ITMS MS2?

Empirically, our lab has found that ETD is effective on glycopeptides below approximately *m/z* 850, while supplemental activation (EThcD) improves spectral quality for glycan localization on larger precursors. After meeting the intensity threshold and glycan fingerprint ion requirements in HCD, glycopeptides in our analyses below *m/z* 850 were selected for ETD and detected in the ion trap, while EThcD was triggered for glycopeptides above *m/z* 850 with fragments detected in the orbitrap (FTMS). This is described in greater detail in the Materials & Methods section:

"**Detection of at least 3 HexNAc or Neu5Ac fingerprint ions (*m/z* 126.055, 138.055, 144.07, 168.065, 186.076, 204.086, 274.092, 292.103, and 316.102) present at ± 10 ppm and greater than 5% relative intensity triggered additional fragmentation on precursors below *m/z* 850 (with an intensity of at least 5e4) using electron transfer dissociation (ETD), and fragments were detected in the ion trap at a normal scan rate. For precursors between m/z 850-1300 (with an intensity over 5e5), detection of fingerprint ions triggered ETD with supplemental activation (EThcD) at 15% nCE, which was analyzed in the Orbitrap with a resolution of 7,500. Calibrated charge-dependent reaction times were used for ETD and EThcD fragmentation, with 200 ms maximum injection time and custom injection targets.**"

Provide details concerning FDR strategy and threshold.

The protein-level FDR cutoff was set to 1% for Byonic searches of unmodified peptide data; however, we note that for glycopeptide data this statistic is unreliable, hence our manual interpretation of all data. Regardless, this information has been added to the methods section:

"**Tryptic peptide *.raw files were searched against the human proteome with Byonic (Protein Metrics).69 Mass tolerances of 10 ppm, 20 ppm, and 0.3 Da were used for MS1, FTMS MS2, and ITMS MS2, respectively. Met oxidation was selected as a variable modification. The search was set to "fully specific" with proteolytic cleavage C-terminal to R and K, allowing 2 missed cleavages. The protein-level FDR was set to 1%.**"

Lines 659 – 661: Indicate which peptides were identified using this method and considered based on what criteria.

Regrettably, we did not track which glycopeptides were originally assigned by software or solely by manual analysis of LC-MS data, but we assure the reviewer that we are confident in our assignments regardless of how they were primarily identified. For manual assignment of glycopeptides, we first require that the MS1 precursor mass error is consistent with other glycopeptides identified in the same run. We then require near-complete backbone sequence coverage and the presence of the HexNAc fingerprint in the low-mass region in HCD spectra. Because we typically observe the naked peptide backbone sequence in HCD, the total glycan mass is readily obtained by subtraction of this mass from the intact precursor mass. Due the modular nature of glycans, we can then deduce the total glycan composition from this number. We then check for various glycan localizations in electron-based dissociation MS2s before using BLAST to assign the sequence to a specific protein. We assess the protein assignment based on our other results – if additional glycopeptides from a specific protein have already been identified, we are more confident in the BLAST results.

Supplementary table 2: Report probability score.

We thank the reviewer for their suggestion but respectfully disagree with the necessity of a probability score in this context. All spectral data for identifications listed in supplementary table 2 were manually reviewed for accuracy, thus we feel that reporting a probability score is inaccurate. Furthermore, this table includes glycopeptides identified via manual analysis that do not have a software-derived score available. Please refer to Chongsaritsinsuk et al. *Nat Commun* 14, 6169 (2023) for a detailed description of how our lab manually inspects all HCD, EThcD, and ETD spectra to confirm the backbone sequence and glycan localization of glycopeptides identified by search algorithms.

Reviewer #2 (Remarks to the Author):

In this study, the authors developed a workflow combining on-tissue mucinase StcE digestion with MALDI mass spectrometry imaging (MALDI-MSI) and LC-MS to spatially map mucin O-glycopeptides in tumor tissues. This approach revealed distinct MUC2 glycoform distributions within colorectal mucinous adenocarcinomas and achieved near-complete sequence coverage of MUC2 mucin domains. Additionally, the authors identified endogenous O-acetylated GalNAc, a novel glycan modification. Their findings demonstrate the potential of this technique to advance glycoproteomic studies in cancer and other fields. While technically advanced, the work is highly specialized and contains numerous areas where explanations are insufficient. Further development and clarification will be necessary for publication in a journal that also targets readers outside this specific field.

We thank the reviewer for their kind words and thoughtful feedback. We hope that our significant revisions alleviate any concerns about our work.

Major points:

1. In Figure 1, the intent behind showing N-glycan imaging should be clarified. Is the purpose simply to demonstrate that the N-glycan processing required for imaging O-glycopeptides worked effectively? If so, this should be clearly stated. This content might be more suitable in a supplementary figure.

We apologize for the lack of clarity. The purpose of showing the N-glycan heat maps in Figure 1 was to demonstrate our workflow's compatibility with additional enzymes to enable multimodal MALDI-MSI. We agree that the original manuscript was unclear on this point and have revised the text accordingly:

"Nearly all ions detected in the cancerous tissues in high abundance outside of the tumor boundaries were N-glycans, although we identified multiple N-glycans within the boundaries of the salivary gland tumor (Supplementary Fig. 7), showing the compatibility of our workflow with orthogonal enzymatic treatments for multi-omic imaging."

We also replaced the images in Figure 1F with non-N-glycan species, to prevent further confusion.

2. Line 298-It would be helpful for readers of Nature Communications if the Introduction included definitions of the T antigen and Tn antigen, as well as the fact that these are dominantly detected by mass spectrometry from glycoproteins. This background information would aid reader understanding.

We thank the reviewer for their helpful suggestions. We have updated the introduction to include the monosaccharide composition of the Tn and T antigens:

"**Specific O-glycan structures associated with cancer include the Tn (GalNAc or N1), sialyl-Tn (STn, GalNAc-Neu5Ac or N1A1), and Thomsen–Friedenreich (T, GalNAc-Gal or H1N1) antigens.**"

We have also added legends to Figure 2 and Supplementary Figures 20 & 21 for enhanced clarity:

Figure 2:

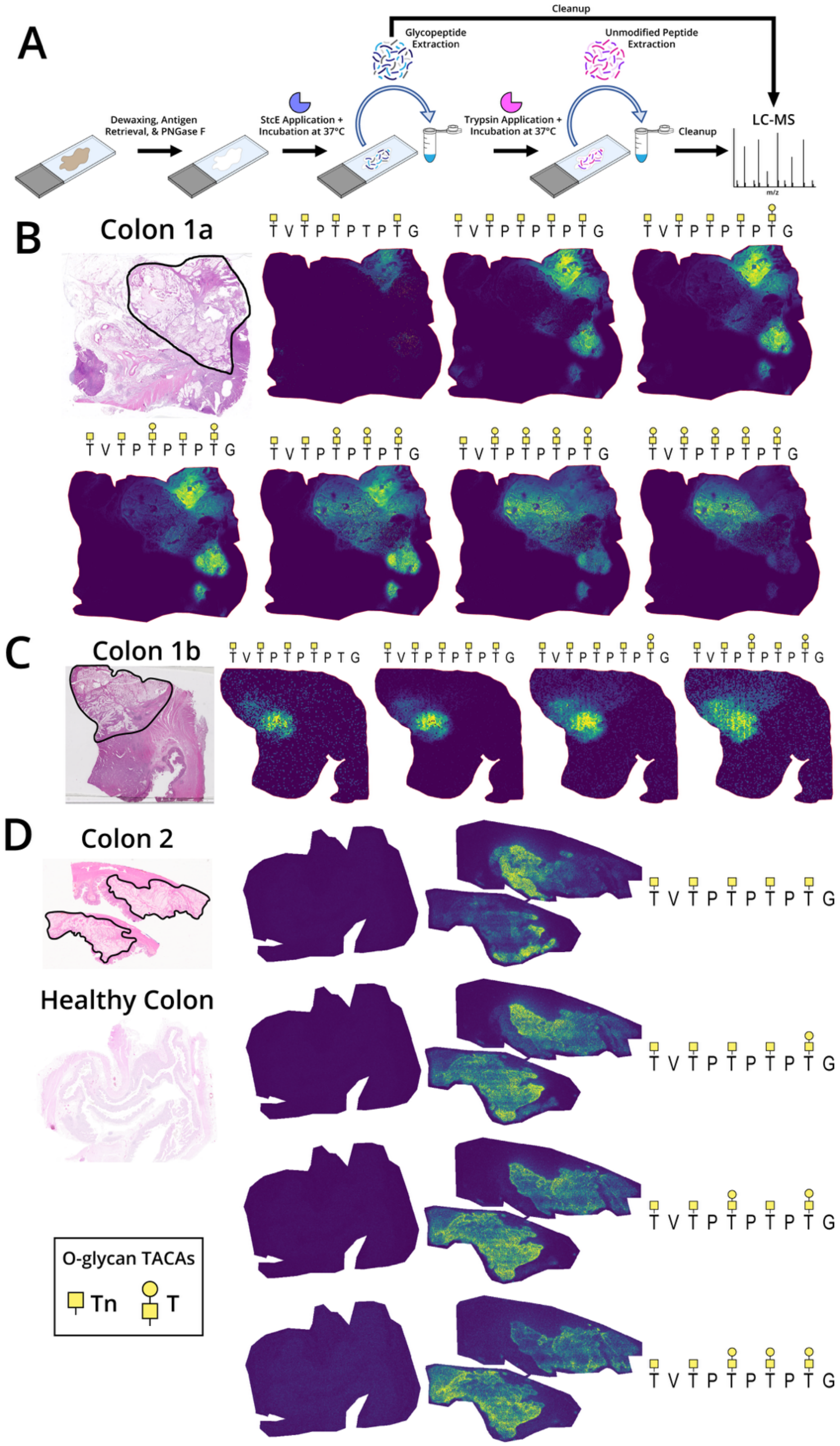

Figure 2. Identification of StcE-derived O-glycopeptides via LC-MS. (a) Serial tissue sections were dewaxed, antigen-retrieved, and PNGaseF-treated. Following rinse steps and on-slide digestion with sprayer-applied StcE, the tissue surface was saturated with pipette-applied StcE to maximize O-glycopeptide generation. After O-glycopeptide extraction, an on-slide trypsin digestion was performed, and unmodified peptides were collected and fractioned with a HILIC-based approach. All peptide samples were then analyzed with LC-MS. (b) The expression profile of MUC2 glycoforms of the backbone TVTPTPTPTG in Colon 1a varies based on relative amounts of the T and Tn antigens, with the high levels of the former displaying a more diffuse pattern and the latter more clustered to specific areas. The sequence TVTPTPTPTG is from the second PTS domain in MUC2. (c) MUC2 glycoforms in Colon 1b exhibit a similar spatial pattern as in Colon 1a. (d) The same MUC2 glycoforms

shown in (b) and (c) exhibit a similar expression pattern in Colon 2. When normalized to the same intensity as Colon 2, signal for these glycopeptides in the Healthy Colon is nearly undetectable, indicating highly elevated expression of MUC2 with truncated O-glycosylation in Colon 2.

Supplementary Figure 17:

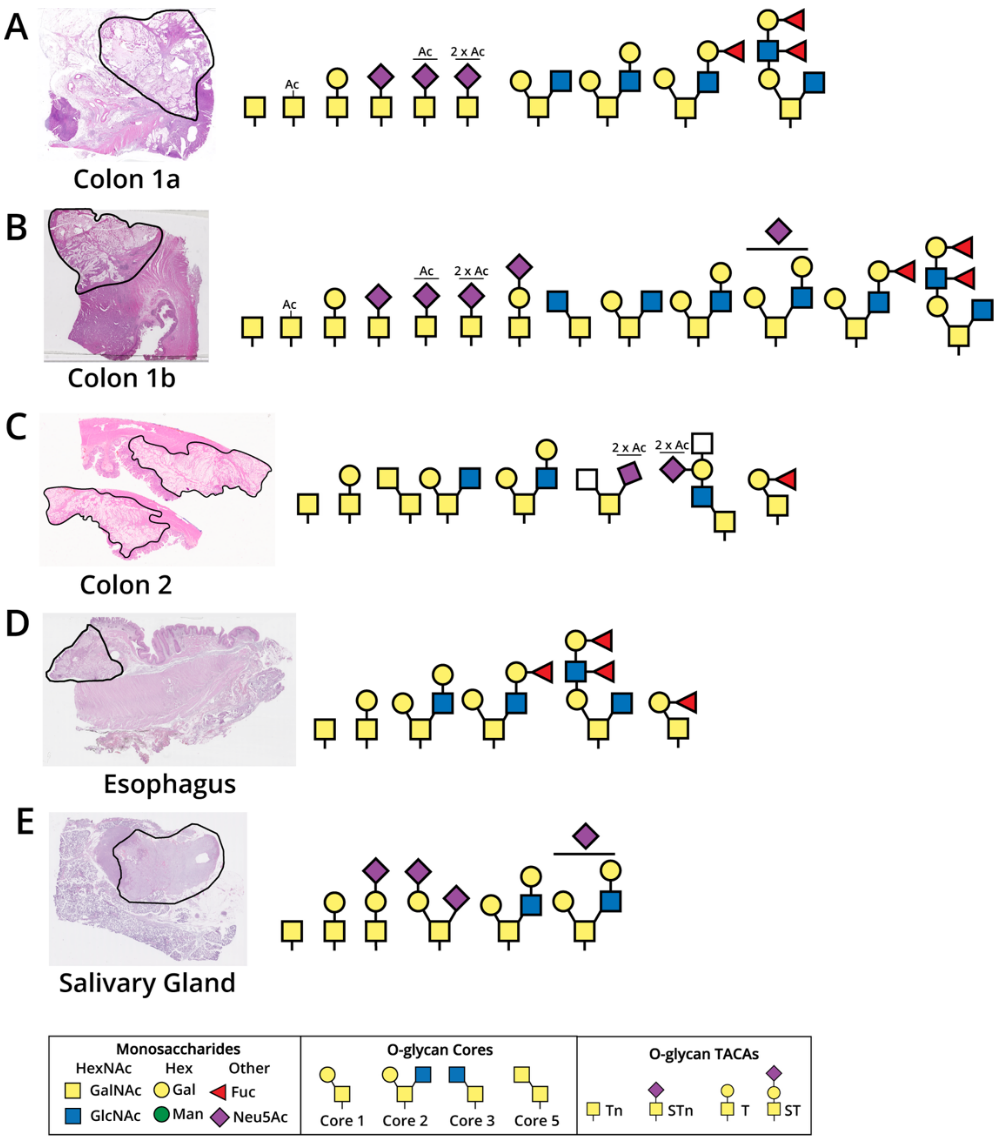

Supplementary Fig. 17. Glycan structures confidently localized in each tumor region (outlined in black) via LC-MS/MS. (A) From left to right: N1 (Tn), AcN1, H1N1 (T), N1A1 (sialyl-Tn), N1AcA1, $N1Ac_2A1$, H1N2, H2N2, H2N2F1, and H2N3F2 could be localized to specific sites on Colon 1a glycopeptides. (B) From left to right: N1 (Tn), AcN1, H1N1 (T), N1A1 (sialyl-Tn), N1AcA1, $N1Ac_2A1$, H1N1A1 (sialyl T), N2, H1N2, H2N2, H2N2A1, H2N2F1, and H2N3F2 could be localized to specific sites on Colon 1b glycopeptides. (C) From left to right: N1 (Tn), H1N1 (T), N2, H1N2, H2N2, $N2Ac_2A1$, $H2N3Ac_2A1$, and H1N1F1 could be localized to specific sites on Colon 2 glycopeptides. White squares indicate ambiguous HexNAc. (D) From left to right: N1 (Tn), H1N1 (T), H2N2, H2N2F1, H2N3F2, and H1N1F1 could be localized to specific sites on Esophagus glycopeptides. (E) From left to right: N1 (Tn), H1N1 (T), H1N1A1 (sialyl T), H1N1A2 (di-sialyl T), H2N2, and H2N2A1 could be localized to specific sites on Salivary Gland glycopeptides.

Supplementary Figure 18:

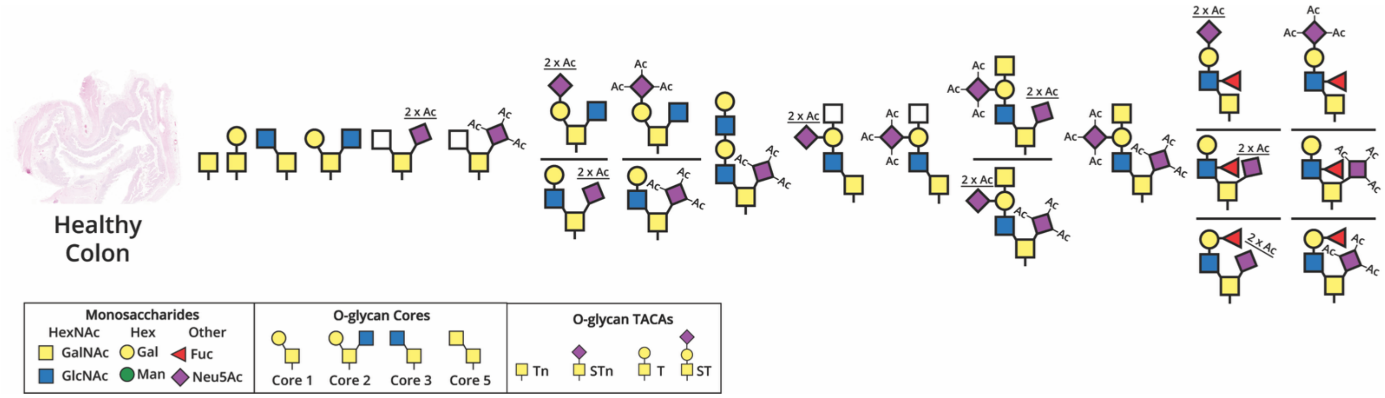

Supplementary Fig. 18. Glycan structures confidently localized on glycopeptides extracted from the Healthy Colon via LC-MS/MS. White squares indicate ambiguous HexNAcs. Vertically stacked glycans separated by lines indicate possible structural isomers based on monosaccharide composition. Ac, Acetyl. Proposed glycan structures are based on a previously published glycomic characterization of human intestinal MUC2-derived O-glycans.[5]

3. In Figure 2, images of two peptides, TPSPPTT and TVTPTPTPTG, are shown. The authors only mention TPSPPTT, but is the statement intended to apply to both glycopeptides? If so, this should be clearly indicated.'

We thank the reviewer for catching this oversight. Our revised version of Figure 2 includes images only for TVTPTPTPTG glycoforms (see above), and we have corrected the figure caption:

"**(b) The expression profile of MUC2 glycoforms of the backbone TVTPTPTPTG in Colon 1a varies based on relative amounts of the T and Tn antigens, with the high levels of the former displaying a more diffuse pattern and the latter more clustered to specific areas. The sequence TVTPTPTPTG is from the second PTS domain in MUC2.**"

4. Line 327-The statement that glycan structures were "more diverse" on peptide sequences from the second PTS domain (Fig. 3c) should be supported by quantitative evidence. Please consider including a measure of diversity, such as the number of distinct glycoforms or another appropriate metric.

We have edited the language to communicate that we identified extended glycan structures on the second PTS domain in addition to the T/Tn antigens:

"**Most of the MUC2 glycopeptides identified in the colorectal tumors were decorated with a combination of T and Tn antigens, although we detected evidence indicating the presence of core 2, fucosylated, and sialylated O-glycans that could not be localized to specific Ser or Thr residues (Supplementary Fig. 19a and Supplementary Data 2). Notably, we only detected evidence for a single sialylated O-glycopeptide in the first PTS domain, and more complex fucosylated and acetylated structures in PTS 2 (Supplementary Fig. 19c-d). In the first, shorter PTS domain, we confidently localized only Tn and T antigen structures, with the exception of one core 2 structure mapped to a single site (Fig. 3b). We localized these and additional, more extended O-glycan structures on peptide sequences from the second PTS domain (Fig. 3c).**"

Additionally, we have updated Supplementary Figure 16 with two additional panels to visualize the differences in the glycoforms identified in the two PTS domains of MUC2:

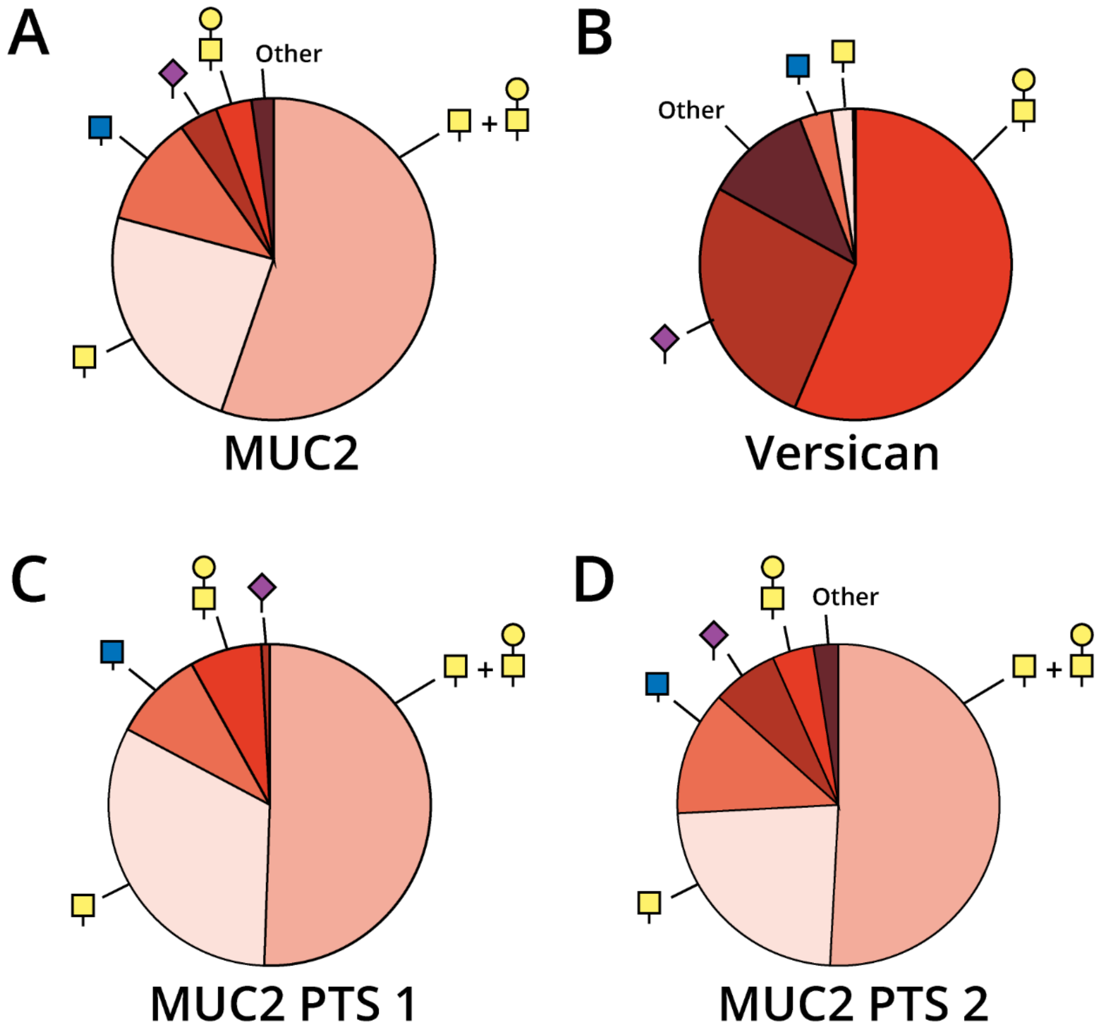

Supplementary Fig. 16. Pie charts showing distribution of identified glycoforms. All glycoforms recorded in Supplementary Data 2 for each protein or protein domain were binned into one of six categories (Tn-only, Tn + T, T-only, GlcNAc-containing, sialylated, and other), and the total number of sequences in each category were summed. (A) MUC2 glycopeptides (N=698) can be categorized into Tn+T (365, 52.3%), Tn-only (173, 24.8%), GlcNAc-containing (81, 11.6%), sialylated (35, 5.0%), T-only (32, 4.6%), and other (12, 1.7%) species. (B) Versican glycopeptides (N=251) can be categorized into T-only (142, 56.6%), sialylated (67, 26.7%), other (28, 11.2%), GlcNAc-containing (8, 3.2%), and Tn-containing (6, 2.4%) species. (C) MUC2 PTS1 glycopeptides (N=174) can be categorized into Tn+T (88, 50.6%), Tn-only (56, 32.2%), GlcNAc-containing (16, 9.2%), T-only (13, 7.4%), and sialylated (1, 0.6%) species. (D) MUC2 PTS 2 glycopeptides (N=515) can be categorized into Tn+T (263, 51.1%), Tn-only (120, 23.3%), GlcNAc-containing (64, 12.4%), sialylated (34, 6.6%), T-only (22, 4.3%), and other (12, 2.3%) species."

5. Line 37- Sialic acids are known to be susceptible to hydrolytic cleavage under acidic (low pH) conditions. Could the unexpectedly low sialic acid content in the other samples be due to acid-induced loss during sample preparation? In the salivary gland tumor sample, where sialic acid levels were extremely high, some may have remained detectable despite such degradation — but this may not be the case for the other samples.

We appreciate the reviewer's inquiry about the lack of sialylation in the gastrointestinal tumors, a feature that was initially concerning to us as well. As the reviewer recognizes, the same workflow applied to the salivary gland tumor resulted in abundant sialylated species. This, in addition to the detection of multiple sialylated species in the new data from the healthy colon tissue, indicates that the lack of sialic acid in the colorectal/esophageal tumors is unlikely to be caused by a technical issue. The main text includes discussion regarding this topic:

“In all tumor samples and the Healthy Colon, we identified glycopeptides decorated with a variety of O-glycan structures (Supplementary Figs. 20a-e & 21). In the colorectal and esophageal tumor data, the distribution of identified glycoforms largely resembled that of MUC2 (Supplementary Figs. 22a-d). Notably, Neu5Ac-containing glycans were not particularly abundant in the colon and esophageal tumors, evidenced by ion chromatograms for protonated Neu5Ac and its water loss at *m/z* 292.1027 and 274.0921, respectively (Supplementary Fig. 23). This result was surprising, as cancer is typically associated with hypersialylation of N- and O-glycans.[20] We did detect some sialyl T-containing MUC2 glycopeptides in the colorectal tumors with LC-MS and MALDI-MSI, although at a much lower intensity than non-sialylated versions of the same species (Supplementary Fig. 24 and Supplementary Data 2). Some data has suggested that StcE does not readily cleave mucins heavily decorated with STn,[56] however, the scarcity of sialyl-T and other Neu5Ac-containing structures in the MUC2 data suggests that StcE’s cleavage restrictions did not skew our glycoproteomic results.

Sialic acids are prone to in-source fragmentation (ISF) in both ESI and MALDI,[57] which might have contributed to poor detection of sialylated glycopeptides. However, the Neu5Ac fingerprint signal was robust in the salivary gland tumor LC-MS data (Supplementary Fig. 23), thus we concluded that the unexpectedly low sialic acid content in other samples likely was not a technical artifact. Regardless, future studies may benefit from an on-slide derivatization step during sample preparation, which can reduce sialic acid lability during ionization and even distinguish its different glycosidic linkages.[58]”

6. Line 469- The importance of sample size is clear, but the authors should also discuss the advantages of using fresh or unfixed samples over paraformaldehyde-fixed (PFF) tissues. Unfixed samples better preserve protein integrity and modifications, which can improve glycoproteomic analysis. Addressing this would strengthen the manuscript.

As the reviewer notes, unfixed samples are typically considered superior to FFPE tissue for protein integrity. We analyzed tissue that was readily available to us, however, if the workflow performed well for FFPE, we suspect that the results would be even better for unfixed or fresh-frozen tissue. We have added a sentence to the discussion highlighting the potential improvements that could be achieved with alternative preservation methods:

“**Additionally, future work should assess the performance of our workflow when applied to unfixed or fresh-frozen tissue. Samples prepared with these preservation techniques are generally considered superior to FFPE tissue in retaining protein and PTM integrity, thus, we would expect to observe an improved depth of analysis in tissues that were not fixed.**”

7. Line 498- If staining and coverslip removal were performed before MSI, this process should be illustrated in Figures 1A and 2A as well.

We thank the reviewer for catching our unintentional omission. For this work, a representative tissue section from each block was H&E stained for structural reference. We did not H&E stain the exact samples used for MALDI-MSI nor LC-MS, as our primary goal was to assess the performance of our coupled-MS workflow rather than to extract biological insights from our data. However, this process is often performed (and the stain washed out) prior to on-slide digestion or matrix application (in the case of MALDI-MSI). We do note that the MALDI-MSI instruments collect autofluorescence images of each tissue prior to laser application such that minor structural differences are accounted for during data analysis.

8. In this paper, “m/z” is written after the numerical values, but it should be placed before the numbers (e.g., m/z 300–3500).

We thank the reviewer for identifying this error and have corrected our manuscript accordingly.

9. Line 637- It is unclear whether these settings (formaldehyde-related modifications such as lysine crosslinks and dehydration and allowing more missed cleavages) were applied to the PFF samples. Please clarify.

We did not find evidence of significant FFPE-related modifications when optimizing our workflow, thus we did not include these in our search parameters in downstream analyses. Due to the relative paucity of Lys in mucin domains, we don't expect fixation to significantly alter our glycoproteomic results for data derived from StcE-only digests. Furthermore, we manually assessed many spectra with the most intense m/z 204 signal in each analysis. If crosslinking or other preservation-related artifacts were pervasive, we expect we would have detected it during this part of our analysis.

10. Line 661- Could the authors clarify how they assigned peptides that are shared among multiple proteins to a single protein? Specifically, what criteria or rules were applied to resolve ambiguous peptide-to-protein mappings? Providing this information would improve the clarity and reliability of the protein assignments. Or were there no ambiguous peptide-to-protein mappings observed?

Thank you for this astute point. For O-glycopeptides with sequences that mapped to more than one mucin, we assigned identifications based on what is known about StcE's cleavage preferences as well as the presence of tryptic peptides in the unmodified peptide samples. For example, the backbone sequence TPITT/TPLTT (Leu and Ile cannot be distinguished with the MS acquisition method used) maps to multiple proteins. Many of these proteins are intracellular and thus highly unlikely to carry extracellular O-GalNAc-type glycosylation. TPITT is repeated 69 times in the canonical MUC2 sequence and is preceded/flanked by residues that are consistent with the StcE cleavage motif.

On the other hand, TPLTT is also found in multiple proteins, including 3 times in the canonical MUC16 sequence, but the preceding and flanking residues adjacent to this sequence are not consistent with StcE's cleavage motif. TPLTT is also present in MUC17, with two instances of the sequence having correct preceding/flanking residues for StcE cleavage. However, neither MUC16 nor MUC17 were identified via tryptic peptides in the unmodified peptide samples. Taken together, we feel that assigning this sequence to MUC2 is not unreasonable, given the high abundance of MUC2 in these samples, the number of repeats in the MUC2 sequence, and the lack of evidence for the presence of alternative proteins.

We agree with the reviewer that this is pertinent information and have added a paragraph discussing these considerations to the main text:

"**Additionally, some backbone sequences we attributed to MUC2 are technically present in other proteins. We considered StcE's known cleavage preferences, total proteome data (from unmodified peptides), and cellular compartment annotations to make assignments in these cases. For example, because our MS acquisition method cannot distinguish Leu from Ile, the sequence TPITT could, in theory, be TPLTT. Both of these sequences map to numerous proteins, however, many of these can be excluded from consideration as they are intracellular proteins and thus highly unlikely to be glycosylated with O-GalNAc glycans. TPLTT is repeated three times in the canonical MUC16 sequence and twice in MUC17, but only in MUC17 is the TPLTT sequence flanked by residues consistent with StcE's cleavage motif. MUC17 was not identified in the unmodified tryptic data, however. On the other hand, MUC2 is highly abundant in tryptic data and the sequence TPITT is repeated 69 times in the canonical MUC2 sequence flanked by residues corresponding to StcE cleavage. Taken together, we felt confident assigning MUC2-derived TPITT as the identity of the glycopeptide.**"

Other concerns:

11. Line 120- the authors use the term "high spatial resolution," but this term is ambiguous. Instead, they should specify the actual resolution, for example "tens of micrometers resolution".

We apologize for the lack of clarity regarding MALDI-MSI data acquisition. While most of our imaging data was collected at a resolution of 40-50 µm, Colon 1b's pixel size was much larger than the rest at approximately 200 µm. We detailed our justification for this in a response to the previous reviewer, which we have copied here:

"The reviewer is correct in noting the lower MALDI-MSI resolution of Colon 1b (formerly labeled Colon 2) with a pixel size of approximately 200 µm compared to 40-50 µm in images of other tissues. We believe this disparity highlights the sensitivity of our approach, which still discerned glycoform-specific expression patterns at a much lower resolution. Because of the long acquisition times required for ultra-high resolution MALDI-MSI analysis of entire tissue sections, rapid screening of tissues (the total MALDI-MSI acquisition time for Colon 1b was less than 1 minute) prior to full-length analyses can potentially increase the pace of future work using this approach."

We have revised the manuscript with the language recommended by the reviewer:

"**Matrix-assisted laser-desorption mass spectrometry imaging (MALDI-MSI) is a technique that is commonly employed to analyze glycans within preserved biological tissues with high spatial resolution, often in the range of tens of micrometers.**[44]"

12. Line 129- The use of "However" in the sentence seems somewhat inappropriate. The statement about analytes typically being identified only by their intact precursor mass in MALDI-MSI does not directly contrast or contradict the preceding hypothesis, but rather provides additional context or explanation.

We thank the reviewer for their comment and have corrected the wording in the main text.

13. A general comment applicable to all figures, including supplementary ones: according to international conventions, the symbol "m/z" should be consistently italicized throughout the manuscript.

We thank the reviewer for their comment and have italicized all instances of *m/z*.

14. Line 161- "Nearly all ions detected in high abundance outside of the tumor boundaries were residual N-glycans,"

It looks like the reviewer's question/comment was not completed – we would be happy to address any issues with this line in future versions.

15. Line 309- Figure 13 is referred to, but it should be Supplementary Figure 13.

We thank the reviewer for their comment and have corrected the referenced figure.

16. Line 372- The same asterisk annotation used in Fig. 4 should also be included in Supplementary Figure 22.

The figure has been updated to include the annotated acetylated Neu5Ac structure.

17. Line 434-If the authors wish to emphasize that the number is "many," the term is somewhat ambiguous. It would be helpful to provide an estimate or approximate cleaved patterns by non-expected enzymes to support this statement.

We thank the reviewer for their comment and have added the requested numbers: "Interestingly, many versican peptide sequences corresponded to non-StcE proteolytic cleavage at the N-terminus (n = 25, 9.8%), C-terminus (n = 110, 43.3%), or both (n = 9, 3.5%) (Supplementary Data 2)."

18. Line 484It seems that “μM” was used to describe tissue thickness, which is likely a typo (μm).

We thank the reviewer for their comment and have corrected the text.

In addition, information about the conductive slide glass for MALDI-MSI should be included here.

We thank the reviewer for their comment and have specified our use of conductive ITO slides for MALDI-MSI experiments:

“**Tissues were sectioned onto ITO conductive slides at thickness of 5 μm for further sample preparation.**”

19. Line 656-The authors should report the approximate number of ions manually searched for MSI based on the LC-MS results. Disclosing the workload involved in this approach is important for scientific publications aiming for reproducibility.

We thank the reviewer for raising an important point and agree that the text should better describe the effort required for this work. We searched MALDI-MSI data for all masses corresponding to the mono-sodiated, singly charged *m/z* values for each glycopeptide identified via LC-MS. Note that the presence of isobaric glycopeptides reduced the total number of values searched for in MALDI-MSI files compared to the total number of glycopeptides reported in Supplemental Data 2. We estimate that the total number of masses searched for in MALDI-MSI data is approximately 300-400, although we would like to note that SCiLS Lab (Bruker software used to analyze imaging data) greatly reduces the workload at this step. Instead of having to manually search for each mass in the MALDI-MSI data, this program has the ability to compile images for all detected masses in a user-uploaded list of masses. We could readily convert all LC-MS masses to MALDI-MSI masses, upload the list, and then quickly visually inspect all MALDI images exported by the software.

This analytical approach is described in the Materials & Methods, and we have added the following text to the discussion:

“While this work represents a significant milestone in mucin glycobiology, we emphasize that in-depth characterization of full-length MUC2 and other canonical mucins are unlikely to become routine in the near future due to persistent technical obstacles. The software used in this work provided an extremely helpful starting point in our analyses, but the lack of search algorithms that can accurately and reliably localize multiple O-glycan structures on a given peptide remains the most significant bottleneck in such work. Manual assessment of all glycopeptide identifications in LC-MS data alone was an intensive, months-long effort. The presence of numerous possible O-glycosites, evidence for multiple glycoforms in the same or additional chromatographic peaks, and isobaric sequences from the PTS domains of MUC2 are just a few examples of mucin-related challenges we addressed. The dominance of truncated O-glycans in our data was a small mercy in this work, which would have been further complicated (and therefore slowed) by an abundance of extended structures.

Matching LC-MS identifications to MALDI-MSI data was another hurdle requiring significant trial and error on our part. Although vendor software facilitated extraction of ion intensity maps for a given list of precursor masses (see Materials & Methods), many of the exported images were simply noise. This necessitated visual inspection of all extracted images (approximately 300-400) to remove low-quality data. However, the detection of glycoform-specific expression patterns was a serendipitous result of this process, underscoring the importance of manually reviewing raw data to enhance the value of more sophisticated computational approaches.”

Reviewer #3 (Remarks to the Author):

This manuscript reports on proof-of-concept experiments carrying out spatial analysis of O-linked glycoproteins on mucinous carcinomas. The authors have looked at three different mucinous carcinomas. Being a proof-of-

concept paper, the samples numbers are extremely low, 2 colorectal samples (from the same patient), one salivary and one oesophageal.

We thank the reviewer for their extremely thorough feedback. We do recognize that the low number of samples limits our ability to draw any biological conclusions from our data, however, we would like to emphasize that our goal was to demonstrate the technical feasibility in this proof-of-concept work. In the revised manuscript, we have integrated data from two additional samples, another mucinous colorectal tumor (from a different donor) and a section of healthy colon tissue. With these two additional tissues, we hope we acquired sufficient data to demonstrate the utility of our approach.

The decision to use mucinous carcinomas for the analysis O-linked glycans may have resulted in technical problems due to the large amount of secreted mucins. As mucins are upregulated in many non-mucinous carcinomas, including colorectal, these may have been a better choice.

We thank the reviewer for highlighting the importance of sample selection. We would be happy to address the reviewer's specific concerns about our approach, but we are unclear as to what the reviewer means by "technical problems." We respectfully disagree with the reviewer regarding the use of mucinous carcinomas in this work. Because we used StcE, a mucin-specific protease, we knew that using samples with an abundance of mucus would maximize the potential recovery of glycopeptides for analysis. We would also like to point out that the salivary gland tumor was non-mucinous, and we detected an abundance of glycopeptides from the mucinous proteoglycan versican. That being said, in future work we hope to apply this workflow to characterize non-mucinous carcinomas and further optimize our approach as needed.

There are a number of issues that elicit concern regarding the methodology:

• After treatment to remove N-linked glycans, the sections were treated with the mucinase, StcE. The step is vital to allow release of mucin O-linked glycopeptides but no analysis of the efficacy of the StcE was carried out and so the ability of this purified enzyme to cut at the specific mucin site after the tissue was treated in the way described, was not determined. **(???)**

While we appreciate the reviewer's dedication to high-quality and thorough characterization of StcE, we respectfully point the reviewer to previous works that were dedicated to characterizing the activity and specificity of StcE:

Malaker SA, Pedram K, Ferracane MJ, Bensing BA, Krishnan V, Pett C, Yu J, Woods EC, Kramer JR, Westerlind U, Dorigo O, Bertozzi CR. The mucin-selective protease StcE enables molecular and functional analysis of human cancer-associated mucins. Proc Natl Acad Sci USA. 2019 Apr 9;116(15):7278-7287. doi: 10.1073/pnas.1813020116. Epub 2019 Mar 25. PMID: 30910957; PMCID: PMC6462054.

Shon DJ, Malaker SA, Pedram K, Yang E, Krishnan V, Dorigo O, Bertozzi CR. An enzymatic toolkit for selective proteolysis, detection, and visualization of mucin-domain glycoproteins. Proc Natl Acad Sci U S A. 2020 Sep 1;117(35):21299-21307. doi: 10.1073/pnas.2012196117. Epub 2020 Aug 17. PMID: 32817557; PMCID: PMC7474620.

Malaker, S.A., Riley, N.M., Shon, D.J. *et al.* Revealing the human mucinome. *Nat Commun* 13, 3542 (2022). https://doi.org/10.1038/s41467-022-31062-4

Additional work from our own lab and many others collectively demonstrates the robust and reproducible performance of StcE for mucin digestion. A small sampling of the published literature using StcE:

Nason, R., Büll, C., Konstantinidi, A. *et al.* Display of the human mucinome with defined O-glycans by gene engineered cells. *Nat Commun* 12, 4070 (2021). https://doi.org/10.1038/s41467-021-24366-4

Park, S., Colville, M.J., Paek, J.H. *et al.* Immunoengineering can overcome the glycocalyx armour of cancer cells. *Nat. Mater.* 23, 429–438 (2024). https://doi.org/10.1038/s41563-024-01808-0

Shi, S.M., Suh, R.J., Shon, D.J. *et al.* Glycocalyx dysregulation impairs blood–brain barrier in ageing and disease. *Nature* 639, 985–994 (2025). https://doi.org/10.1038/s41586-025-08589-9

Javitt G, Khmelnitsky L, Albert L, Bigman LS, Elad N, Morgenstern D, Ilani T, Levy Y, Diskin R, Fass D. Assembly Mechanism of Mucin and von Willebrand Factor Polymers. Cell. 2020 Oct 29;183(3):717-729.e16. doi: 10.1016/j.cell.2020.09.021. Epub 2020 Oct 7. PMID: 33031746; PMCID: PMC7599080.

Moreover, although MUC1 was detected in both colon samples, no MUC1 glycopeptides were identified. Each tandem repeat of MUC1 has 2 potential StcE. T*S and S*T. These sites are missing the intermediate amino acid as in S/T*-X-S/T and so as described by Malaker et al 2019 the cutting efficiency of StcE is reduced (* equal glycosylated aa for StcE cleavage). This may be an indication that the cutting efficiency of the StcE used in the experiments nor/or the condition of material is not optimal, as previous studies have shown that StcE can cut MUC1.

We respectfully disagree with the reviewer that the quality of the StcE and tissue samples was subpar. While MUC1 was detected via unmodified peptides, a low overall protein abundance likely means that the intensity of different glycopeptides was below the limit of detection. MUC1's tandem repeats do not contain canonical StcE cleavage motifs, although there are StcE cleavage sites outside of the tandem repeats. This is in line with work published by the Clausen group, which found that StcE did NOT cleave MUC1 tandem repeats (Nason, R., *et al.* *Nat Commun* **12**, 4070 (2021)), and thus digestion of MUC1 by StcE is likely driven by cleavage at sites outside of the tandem repeats.

The publication referenced by the reviewer demonstrated efficient cleavage of purified, recombinant PSGL-1 (not MUC1) at S/T*-S/T* *in the absence of* StcE's preferred motif. The samples we analyzed were highly complex with mucins containing StcE's preferred sequence. This, in conjunction with its overall low protein abundance, likely account for the lack of MUC1 O-glycopeptides in our data, as discussed in the main text:

"**MUC1 overexpression is estimated to occur in over half of all cancers,[15] and we detected this transmembrane mucin in Colons 1a & 2b as well as the esophageal tumor. Notably, we did not identify any glycopeptides from MUC1. StcE cleaves mucins at T/S*XT/S" motifs, in which a glycosylated Ser or Thr is followed by any amino acid (X) and then another Ser or Thr.[60] Cleavage occurs C-terminally to the "X" residue, and StcE can accommodate glycosylation at any position within the motif. Although MUC1 contains very few putative StcE cleavage sites in its tandem repeats, numerous StcE-sensitive sequences can be found outside this region. Therefore, we suspect the lack of MUC1 glycopeptides from Colons 1a and 1b stems from its relatively low abundance compared to MUC2 in these samples.**

Although we were unable to detect MUC1 via LC-MS in Colon 2 and the Healthy Colon, immunohistological staining of MUC1 in these samples revealed weak expression in both tissues (Supplementary Figs. 13a-b). In contrast, staining with the catalytically inactive StcE^{E447D}, which binds any mucin domain-containing proteins in tissue, revealed an inverse expression pattern (Supplementary Figs. 13a-b). While MUC1 staining was strongest at the outer edges of the mucosa, StcE^{E447D} staining was detected throughout the mucosa and submucosa (Supplementary Figs. 13a-b). These data suggest StcE used to prepare LC-MS samples could not favorably bind to MUC1, in contrast to previous work demonstrating StcE's activity on MUC1. This observation might be explained by unfavorable binding in a complex tissue or that our current antigen retrieval step does not adequately expose the MUC1 backbone for binding and cleavage by StcE."

Supplementary Figure 29:

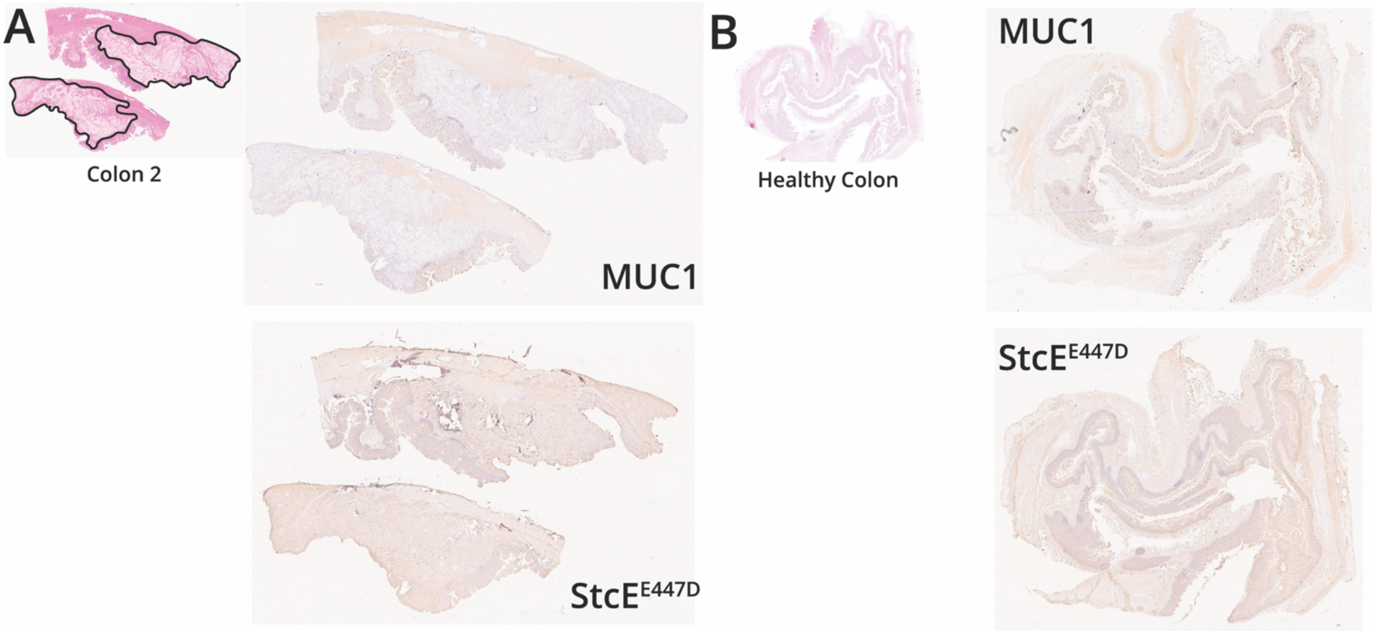

Supplementary Fig. 29. Anti-MUC1 immunohistochemistry and staining with catalytically inactive StcE^{E447D} of serial sections from (A) Colon 2 and (B) the Healthy Colon.

• No MUC1, was not seen in the salivary or oesophageal tumour or normal which is unusual.

Please refer to our response to the previous question.

• Mucinous colorectal carcinomas have been shown previously to express MUC2 and 94% to express MUC5AC (see Walsh et al 2013, Modern Path, 26,1642) but no MU5AC was found in the colorectal samples.

The publication referenced by the reviewer states "…321/649 (**49%**) cancers were classified as MUC5AC-positive." Therefore, the lack of MUC5AC detected in Colons 1a and 1b should not be a cause for concern. Furthermore, we detected both unmodified and glycosylated peptides from MUC5AC in the new data from Colon 2, demonstrating that the previous results were not due to technical issues with our workflow.

• The O-linked glycans found in the colon carcinoma samples were mainly of GalNAc (Tn) and GalNAc-Gal (T). Very little sialylated glycans or other glycan cores were found. This profile is definitely unusual as sialylated glycans are known to be present, as are extended cores. Given these results and the lack of testing of the StcE enzyme, the authors should use the same technique on cell line pellets and compare these with their previous datasets.

We respectfully disagree, again, with the lack of testing on StcE and point the reviewer to the corresponding author's body of work regarding StcE.

However, with regard to sialic acid, we agree that this is an important point. We were also very surprised by the lack of sialylated O-glycans in our initial analyses of the gastrointestinal tumors. As discussed in the main text, we believe the abundance of Neu5Ac-containing glycopeptides in the salivary gland tumor and in the newly added Healthy Colon tissue indicate that the lack of Neu5Ac is not a technical artifact, but rather a biological characteristic of the other tissues:

"**In all tumor samples and the Healthy Colon, we identified glycopeptides decorated with a variety of O-glycan structures (Supplementary Figs. 20a-e & 21). In the colorectal and esophageal tumor data, the distribution of identified glycoforms largely resembled that of MUC2 (Supplementary Figs. 22a-d). Notably, Neu5Ac-containing glycans were not particularly abundant in the colon and esophageal tumors, evidenced by ion chromatograms for protonated Neu5Ac and its water loss at *m/z* 292.1027 and 274.0921,**

respectively (Supplementary Fig. 23). This result was surprising, as cancer is typically associated with hypersialylation of N- and O-glycans.[20] We did detect some sialyl T-containing MUC2 glycopeptides in the colorectal tumors with LC-MS and MALDI-MSI, although at a much lower intensity than non-sialylated versions of the same species (Supplementary Fig. 24 and Supplementary Data 2). Some data has suggested that StcE does not readily cleave mucins heavily decorated with STn,[56] however, the scarcity of sialyl-T and other Neu5Ac-containing structures in the MUC2 data suggests that StcE's cleavage restrictions did not skew our glycoproteomic results.

Sialic acids are prone to in-source fragmentation (ISF) in both ESI and MALDI,[57] which might have contributed to poor detection of sialylated glycopeptides. However, the Neu5Ac fingerprint signal was robust in the salivary gland tumor (Supplementary Fig. 23), thus we concluded that the unexpectedly low sialic acid content in other samples likely was not a technical artifact. Regardless, future studies may benefit from an on-slide derivatization step during sample preparation, which can reduce sialic acid lability during ionization and even distinguish its different glycosidic linkages.[58]"

Further, the most abundant glycans identified in the newly added Healthy Colon tissue are more extended structures, most likely core 3 based on previous glycomic analyses of human intestine-derived MUC2. Taken together, we believe our data is sufficient to rule out technical limitations to explain the abundance of truncated, non-sialylated O-glycans in our results.

• The oesophageal tumours had a very similar profile to the colon tumour, although the salivary tumour had more sialic acid containing glycans. The small numbers of sialylated glycansin the colon samples is very odd. Very little has been published on the O-glycans of salivary tumours.

We were also surprised by the relative paucity of sialic acids in certain tumor samples, as mentioned above. We also concur that the literature on salivary tumor O-glycosylation is somewhat limited. Please see our previous response discussing the limited sialylation detected in the colon/esophageal tumors. We would also like to note that the colon/esophageal tumor glycoproteomic data was dominated by MUC2, but the salivary gland tumor glycoproteome was largely derived from versican.

• The lack of tissue for any kind of validation is of concern. Perhaps it would have been sensible to make sure samples were used where there was sufficient tissue for validation.

Unfortunately, there is nothing we can do to address this comment. We agree this would have been prudent; however, we are at the behest of collaborators that selected the tissue for us. In future studies, we will be sure to heed this advice.

• The symbols in Figure 2 need sorting out.

We apologize for the confusion in this figure and have added a panel labeling the Tn and T antigens in Figure 2.

The results of the glycan analysis and mapping is very dependent on the specificity and efficacy of StcE. Therefore, this must be taken into account when drawing conclusions.

Please refer to our previous response regarding the activity of StcE. We also have qualified our results with the following text:

"**Some data has suggested that StcE does not readily cleave mucins heavily decorated with STn,[56] however, the scarcity of sialyl-T and other Neu5Ac-containing structures in the MUC2 data suggests that StcE's cleavage restrictions did not skew our glycoproteomic results.**"

“These data suggest StcE used to prepare LC-MS samples could not favorably bind to MUC1, in contrast to previous work demonstrating StcE’s activity on MUC1. This observation might be explained by unfavorable binding in a complex tissue or that our current antigen retrieval step does not adequately expose the MUC1 backbone for binding and cleavage by StcE.”

“We identified a single unmodified peptide from MUC5B, a secreted, gel-forming airway mucin. The backbone sequence of MUC5B contains numerous Ser and Thr within TXT motifs, so the relative paucity of MUC5B glycopeptides in the esophageal data was surprising. Like MUC2, this secreted mucin assembles into complex, higher-order structures that may render the protein backbone inaccessible to StcE.[63]”

The small number of tumours of course causes concern over how representative the results are.

We agree with the reviewer that we cannot draw biological conclusions from our current dataset. However, with the two additional samples in the revised manuscript, we believe we have demonstrated the potential value and performance of our coupled-MS workflow to image intact O-glycopeptides in archival tissue. The glycoproteomic results and glycoform-dependent expression patterns originally recorded for Colons 1a and 1b were highly similar to our analyses for Colon 2, the new colorectal tumor tissue. We believe this finding in particular underscores the reproducibility of our method. Even though the total number of samples precludes any generalizations about this type of tumor, we are especially intrigued by the consistency of the MALDI-MSI spatial distributions across 3 tumors from two different patients, collected in two different laboratories. As a result, we look forward to sufficiently expanding our patient cohort to assess the prevalence of this pattern.

However, it is recognised that setting up this technology is not easy, and the authors should be commended for this.

We appreciate the reviewer’s recognition of the effort put into this work.

This manuscript was co-review with an early career researcher

Reviewer #4 (Remarks to the Author):

I co-reviewed this manuscript with one of the reviewers who provided the listed reports. This is part of the Nature Communications initiative to facilitate training in peer review and to provide appropriate recognition for Early Career Researchers who co-review manuscripts.

We thank the reviewer for taking the time to assess our manuscript and provide detailed feedback.

REVIEWERS' COMMENTS

Reviewer #1 (Remarks to the Author):

Overall, the authors have successfully addressed my previous questions and revised the manuscript accordingly. I am satisfied with these changes. I have just one final comment regarding the current version: the newly included data for the additional tumor and healthy tissue samples have not been deposited to PRIDE, which is essential for publication.

We thank the reviewer for their helpful feedback that significantly improved our revised manuscript. The newly included data has been deposited to PRIDE and made publicly available.

Reviewer #2 (Remarks to the Author):

The authors have thoroughly addressed Reviewer #2’s concerns, particularly by improving clarity for a broader readership, refining figure presentation, and explicitly defining analytical assumptions. The revised manuscript is substantially strengthened and now well-aligned with the scope and audience of Nature Communications.

We thank the reviewer for their helpful feedback that significantly improved our revised manuscript.

Reviewer #3 (Remarks to the Author):

I am delighted that the authors were able to include two additional samples; as they say this further highlights the utility of this approach.

My concern regarding mucinous carcinomas is simply that spatial analyses (ie IHC) with these tumours can be challenging (with respect to tumour cell specificity) owing to the extracellular pooling of mucous. However I understand the logic in wanting samples containing an abundance of mucous to enable the testing of the methods, the emphasis of the paper.

The suggestion with the StcE is not that I am unaware of StcE or unaware of the association of the author with this enzyme, it is simply that in my experience it can be pertinent to ensure quality of each batch of agent when the agent is critical for the work, especially when using a new method. For example QC-ing new methods on cell pellets where known glycoproteins are expressed would a good control - and perhaps something to consider for the future.

Thank you for the very clear explanation regarding MUC1, MUC2 and MUC5AC expression, and also the excellent defence of the lack of sialylation owing to technicalities.

We appreciate the reviewer’s feedback regarding QC of StcE. To date, all StcE (produced in-house) has been highly active, based on the success of unrelated experiments performed by other lab members. However, we agree that it would be wise to implement a standardized procedure to test each batch on a defined set of mucins.

Reviewer #4 (Remarks to the Author):

I co-reviewed this manuscript with one of the reviewers who provided the listed reports. This is part of the Nature Communications initiative to facilitate training in peer review and to provide appropriate recognition for Early Career Researchers who co-review manuscripts.

We appreciate the reviewer taking the time to provide helpful feedback on our manuscript.
